# 2023 ACVIM Forum Research Abstract Program

**DOI:** 10.1111/jvim.16913

**Published:** 2023-11-10

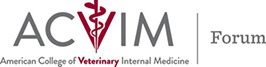

The American College of Veterinary Internal Medicine (ACVIM) Forum and the Journal of Veterinary Internal Medicine (JVIM) are not responsible for the content or dosage recommendations in the abstracts. The abstracts are not peer reviewed before publication. The opinions expressed in the abstracts are those of the author(s) and may not represent the views or position of the ACVIM. The authors are solely responsible for the content of the abstracts.

**2023 ACVIM Forum**

**June 14–October 31, 2023**

**Research Abstract Oral Program**

**Index of Abstracts**

**Table:** 

**WEDNESDAY, JUNE 14**			
Time	#	Presenting Author	Abstract Title
**CARDIOLOGY**			
3:45 PM	C01	Bruna Del Nero	Two‐dimensional Quantitative Methods for Evaluation of Mitral Regurgitation Severity in Canine Degenerative Mitral Valve Disease (ACVIM Resident Research Award Eligible & Cardiology Research Abstract Award Eligible)
4:00 PM	C02	Luke Dutton	Repeatability of Early versus Late Diastolic Left Atrium/Aorta in Cats with Hypertrophic Cardiomyopathy (ACVIM Resident Research Award Eligible & Cardiology Research Abstract Award Eligible)
4:15 PM	C03	Sarifa Lakhdhir	Two‐ and Three‐Dimensional Echocardiographic Assessment of Canine Left Ventricular Outflow Tract Velocity and Area (ACVIM Resident Research Award Eligible & Cardiology Research Abstract Award Eligible)
4:30 PM	C04	Luke Dutton	Significance of Degenerative Tricuspid Valve Disease in Dogs that have Undergone Mitral Valve Repair (ACVIM Resident Research Award Eligible & Cardiology Research Abstract Award Eligible)
**NEUROLOGY**			
3:45 PM	N01	Christopher Adams	Degradation of Midazolam over Time in a Simulated Home Environment (ACVIM Resident Research Award Eligible)
4:00 PM	N02	Sarvenaz Bagheri	Effect of Postoperative N‐Acetylcysteine on Canine Myelopathy Secondary to Intervertebral Disc Extrusion (ACVIM Resident Research Award Eligible)
4:15 PM	N03	Megan Lin	Clinical Characteristics Associated with Cervical Hydrated Nucleus Pulposus Extrusion in Canines (ACVIM Resident Research Award Eligible)
4:30 PM	N04	Wojciech Panek	The CCL2/CCR4 Axis in Regulatory T‐cell Trafficking to Canine Glioma ‐ A Novel Therapeutic Target (ACVIM Resident Research Award Eligible)
**ONCOLOGY**			
3:45 PM	O01	Katie Dittrich	Baseline Tumor Gene Expression Signatures Correlate with Chemoimmunotherapy Treatment Responsiveness in Canine B Cell Lymphoma (ACVIM Resident Research Award Eligible)
4:00 PM	O02	Regina Hayburn	The Immune Landscape of Canine Soft Tissue Sarcomas (ACVIM Resident Research Award Eligible)
4:15 PM	O03	Keira Sztukowski	Vincristine‐Induced Adverse Events as Related to Body Weight in Dogs Treated for Lymphoma (ACVIM Resident Research Award Eligible)
4:30 PM	O04	Skylar Sylvester	Tolerability and Efficacy of Adjuvant Temozolomide and Doxorubicin Combination Chemotherapy for Canine Splenic Hemangiosarcoma (ACVIM Resident Research Award Eligible)
**SMALL ANIMAL INTERNAL MEDICINE ‐ ENDOCRINOLOGY**			
3:45 PM	EN01	Frederik Allan	Expression of Steroidogenic Enzymes in Canine Functional and Non‐functional Adrenocortical Tumors (ACVIM Resident Research Award Eligible)
4:00 PM	EN02	Morgan Brown	The Impact of Single‐dose Trazodone on Endogenous Adrenocorticotropic Hormone and Cortisol Concentrations in Healthy Dogs (ACVIM Resident Research Award Eligible)
4:15 PM	EN03	Jessica Herman	Evaluating a Second‐generation Flash Glucose Monitoring System in Nondiabetic Dogs with Rapidly Induced Hypoglycemia (ACVIM Resident Research Award Eligible)
4:30 PM	EN04	Lauren Porter	Selective Osmotic Shock for the Isolation of Feline Pancreatic Islets of Langerhans (ACVIM Resident Research Award Eligible)
**SMALL ANIMAL INTERNAL MEDICINE ‐ GASTROENTEROLOGY**			
3:45 PM	GI01	Samantha Barth	ADAMTS13 Activity in Dogs with Chronic Enteropathies (ACVIM Resident Research Award Eligible)
4:00 PM	GI02	Petra Cerna	Clinicopathological Findings, Treatment and Outcome of 60 cats with Feline Gastrointestinal Eosinophilic Sclerosing Fibroplasia (ACVIM Resident Research Award Eligible)
4:15 PM	GI03	Mary Bollman	Management of Benign Esophageal Strictures in Dogs and Cats – Long‐Term Follow‐Up of 32 cases (ACVIM Resident Research Award Eligible)
4:30 PM	GI04	Chelsea Lim	Evaluation of KI‐67, Goblet Cell and MUC2 Mucin Expression in Canine Lymphoplasmacytic and Granulomatous Colitis (ACVIM Resident Research Award Eligible)
**THURSDAY, JUNE 15**			
Time		Presenting Author	Abstract Title
**CARDIOLOGY**			
10:00 AM	C05	Michelle Oranges	Prospective Characterization of Electrocardiographic, Echocardiographic, and Natriuretic Peptide Changes Following Radioiodine Therapy in Hyperthyroid Cats (ACVIM Resident Research Award Eligible & Cardiology Research Abstract Award Eligible)
10:15 AM	C06	Evan Ross	Utility of Radiographic Heart Size Measurements for Predicting Heart Failure in Dogs with Respiratory Distress (ACVIM Resident Research Award Eligible & Cardiology Research Abstract Award Eligible)
10:30 AM	C07	Graham Rossi	Laboratory and Clinical Findings in Cats Receiving Amiodarone for Treatment of Supraventricular and Ventricular Arrhythmias (ACVIM Resident Research Award Eligible & Cardiology Research Abstract Award Eligible)
10:45 AM	C08	Rachel Van Zile	Hemodynamic Responses to Alternative Right Ventricular Pacing Site measured in 8 Dogs with AV Block. (ACVIM Resident Research Award Eligible & Cardiology Research Abstract Award Eligible)
11:15 AM	C09	Kristen Verdoorn	Evaluation of Occult Gastrointestinal Bleeding in Canine Left‐Sided Congestive Heart Failure: A Pilot Study (ACVIM Resident Research Award Eligible & Cardiology Research Abstract Award Eligible)
11:45 AM	C11	Karsten Schober	Echocardiographic Assessment of Aortic Root Size in 220 Healthy Cats
12:00 PM	C12	Ryan Fries	Accuracy of Bionote Point‐of‐care Quantitative N‐terminal Pro‐brain Natriuretic Peptide Assay in Dyspneic Dogs and Cats
1:45 PM	C13	Kentaro Kurogochi	Mitral Valve Repair Outcomes in Different Dog Breeds
2:00 PM	C14	Emily Gavic	Clinical, Electrocardiographic, and Diagnostic Imaging Features and Outcomes in 23 Cats with Ventricular Preexcitation
2:15 PM	C15	Lauren Quigley	Hemodynamic, Echocardiographic, Electrocardiographic, and Behavioral Effects of Oral Trazodone in Healthy Dogs
2:30 PM	C16	Todd Sumerfield	Comparison of Two‐dimensional Transesophageal Echocardiographic Quantification of Mitral Valve Annular Area with Three‐dimensional Planimetric Imaging
3:00 PM	C17	Sonja Tjostheim	Effect of Amiodarone, Diltiazem, or Both on Heart Rate Control in Dogs with Atrial Fibrillation
3:15 PM	C18	Brianna Potter	Transpulmonary Stent Implantation in 60 dogs with Congenital Pulmonary Valve Stenosis
3:30 PM	C19	Rachel Melvin	Subclinical Cardiovascular Abnormalities in Apparently Healthy, Aged, Dogs Screened for a Large Randomized Clinical Trial
3:45 PM	C20	Chung‐Yao Yin	Clinical Findings, Outcomes and Risk Factors in 100 Dogs Hospitalized for Acute Cardiogenic Pulmonary Edema
4:30 PM	C21	Lara Barron	Development and Validation of a Machine Learning Algorithm to Detect Canine Heart Murmurs
4:45 PM	C22	MIN SU KIM	Use of a Newly Developed Vascular Occluder System for Closing Patent Ductus Arteriosus in Dog
5:00 PM	C23	Kentaro Kurogochi	Reperfusion Ventricular Fibrillation in Mitral Valve Repair: A Comparison of Blood Cardioplegia and Crystalloid Cardioplegia
5:15 PM	C24	Lauren Markovic	Transcatheter Occlusion of Patent Ductus Arteriosus: Multicenter Collaborative Study Across Pediatric and Veterinary Cardiology Centers
5:30 PM	C25	Marisa Ciccozzi	Amplatz Canine Duct Occluder for Patent Ductus Arteriosus Occlusion in Dogs: Outcomes and Complications
5:45 PM	C26	Erin McCarragher	Hemodynamic, Echocardiographic, and Sedative Effects of Oral Trazodone in Healthy Dogs
11:30 AM	C33	Shira Burg	Allosteric Inhibitors Targeting the Calmodulin‐pip2 Interface of SK4 K+ Channels for Atrial fibrillation Treatment
**NEUROLOGY**			
10:00 AM	N05	Ashley Potts	Kinematic Evaluation of Lumbosacral Vertebral Region in Large‐breed Dogs with and Without Degenerative Lumbosacral Stenosis (ACVIM Resident Research Award Eligible)
10:15 AM	N06	Aaron Rozental	Double‐blinded Crossover Trial of Adjunct Treatment with Cannabidiol in 51 Dogs with Refractory Idiopathic Epilepsy (ACVIM Resident Research Award Eligible)
10:30 AM	N07	Alexa Stephen	Evaluation of microRNA Dysregulation as a Prognostic Indicator in Dogs with Spinal Cord Injury (ACVIM Resident Research Award Eligible)
10:45 AM	N08	Go Togawa	Clinical Outcome in Paraplegic Dogs with Fibrocartilaginous Embolic Myelopathy or Acute Non‐compressive Nucleus Pulposus Extrusion (ACVIM Resident Research Award Eligible)
11:15 AM	N09	Cecilia‐Gabriella Danciu	Concurrent Conditions and Long‐term Outcome Associated with Canine Ischemic Stroke
11:30 AM	N10	Eileen Donoghue	Causal Agents and Imaging in 85 Dogs Diagnosed with Discospondylitis (2018–2022)
11:45 AM	N11	Nicole Block	Utility of Thoracic Radiography Before Advanced Imaging in Dogs with Presumed Intervertebral Disc Herniation
12:00 PM	N12	Devon Hague	Veterinary Students’ Perception of Their Knowledge and Comfort of Clinical Neurology Prior to Their Rotation
1:45 PM	N13	Leanne Jankelunas	Novel COL6A3 Frameshift Mutation Causes Ullrich‐Like Congenital Muscular Dystrophy In American Staffordshire Terrier Dogs
2:00 PM	N14	Melissa Lewis	Land Treadmill Gait Scoring Adapted for Underwater Treadmill in Dogs with Thoracolumbar Intervertebral Disc Extrusion
2:15 PM	N15	Theofanis Liatis	Idiopathic and Structural Episodic Non‐intentional Head Tremor Syndrome in Dogs: 100 Cases (2004–2022)
2:30 PM	N16	Kara Majors	Efficacy of High Dose Cytarabine in 62 Dogs with Severe Meningoencephalitis of Unknown Etiology (MUE)
**NUTRITION**			
4:30 PM	NM01	Aulus Carciofi	A High Starch Kibble Diet Favor Body Weight Control in Neutered Cats Fed ad libitum
4:45 PM	NM02	Itsuma Nagao	High‐Fat Diet Enhances Stemness and Compromises Intestinal Permeability in a Translational Canine Colonoid Models
5:00 PM	NM03	Caitlin Grant	Investigating Differences in Energy Density and Theoretical Calorie Intake of Canadian Raw Diets for Dogs
5:15 PM	NM04	Davide Stefanutti	Effect of Spirulina (Arthrospira platensis) Supplementation in Overweight Dogs Undergoing a Weight Loss Program
**SMALL ANIMAL INTERNAL MEDICINE ‐ ENDOCRINOLOGY**			
11:15 AM	EN05	Jessica Box	Effect of Sodium‐Glucose Cotransporter 2 Inhibitor, Canagliflozin, on Interstitial Glucose in Diabetic Insulin Treated Dogs
11:30 AM	EN06	Sophie Broughton	A Comparison of Biochemical Findings in Cats with Diabetes Mellitus Between Different Body Condition Categories
11:45 AM	EN07	Susan Carr	The Effect of Storage Conditions on Canine Free Thyroxine Measurement by Chemiluminescence and Bulk‐Acoustic‐Wave Assays
1:45 PM	EN09	Molly Bechtold	Untargeted Serum Metabolomic Profiles in Hyperthyroid Cats Before and After I‐131 Therapy
2:00 PM	EN11	Cody Brady	Analytic Performance of a Veterinary‐specific Biosensor‐based Assay for Canine Serum Cortisol Measurement
2:15 PM	EN12	Samuel Stewart	New Generation Device for Blood Glucose Monitoring in Cats
3:00 PM	EN13	Victoria Travail	Likelihood Assessment of Hypothyroidism in Dogs Treated for Hypothyroidism at Primary Care Practices
3:15 PM	EN14	Jonathon Williams	Concurrent Disorders of Cats with Diabetes Mellitus and Arterial Systolic Hypertension
3:30 PM	EN15	Shino Yoshida	Adrenal Incidentaloma: Epidemiology, Clinicopathological Evaluation, Vascular Invasion Pattern, and Histopathological Results
3:45 PM	EN16	Marit van den Berg	Whole Transcriptome Analysis of Canine Pheochromocytoma and Paraganglioma (ESVE Award Winner)
**SMALL ANIMAL INTERNAL MEDICINE ‐ GASTROENTEROLOGY**			
10:00 AM	GI05	Douglas Margarucci	Comparison of Gastroesophageal Reflux between Nasoesophageal and Nasogastric Tubes in Dogs Undergoing Enteral Nutrition (ACVIM Resident Research Award Eligible)
10:15 AM	GI06	Amanda Blake	Expanded Fecal Bile Acid Profile in Dogs with Chronic Enteropathy
10:30 AM	GI07	Chee‐Hoon Chang	Efficacy of Oral or Injectable Supplementation with Cobalamin in Cats with Hypocobalaminemia
10:45 AM	GI08	Chih‐Chun Chen	Evaluation of the Abundance of Ruminococcus gnavus in Dogs with Chronic Enteropathies
11:15 AM	GI09	Bruna Correa Lopes	Stability of Frozen and Lyophilized Clostridium hiranonis in Fecal Microbiota Transplantation Preparations
11:30 AM	GI10	Evan Cosgrove	Assessment of Mucosal Fibrosis and its Correlation with Disease Severity in Cats with Chronic Enteropathy
11:45 AM	GI11	Floris Dröes	Effect of Low‐Fat Diet on Serum Triglyceride and cPLI Concentrations in Dogs with Hypertriglyceridemic Pancreatitis
12:00 PM	GI12	Connie Rojas	Microbiome Responses to Oral Fecal Microbiota Transplantation in a Cohort of Domestic Dogs
**SMALL ANIMAL INTERNAL MEDICINE ‐ HAEMATOLOGY**			
4:30 PM	HM01	Kelly Chappell	Sample Collection Effect on Canine Fecal Occult Blood Testing and Utility of Hemoglobin Immunochemical Tests (ACVIM Resident Research Award Eligible)
4:45 PM	HM02	Antoine Duclos	Leukocyte and Platelet Ratios in Dogs Diagnosed with Non‐associative Immune‐mediated Hemolytic Anemia
5:00 PM	HM03	Francisco De Membiela	Neutrophil‐lymphocyte Ratio, Platelet‐lymphocyte Ratio and Mean Platelet Volume as Prognostic Biomarkers in Critically Ill Dogs
5:15 PM	HM04	Elizabeth Luciani	β1‐tubulin Mutations in Non‐Cavalier King Charles Spaniel Dogs with Macrothrombocytopenia (ACVIM Resident Research Award Eligible)
5:30 PM	HM05	Kimberly Yore	Red Blood Cell Indices Performance to Detect Reticulocytosis in Anemic and Non‐anemic Dogs in Taiwan
5:45 PM	HM06	Gina Dinallo	Heparin Induced VCM Tracing Changes Compared to Aptt and Anti‐xa Activity in ex‐vivo Canine Blood
**SMALL ANIMAL INTERNAL MEDICINE ‐ HEPATOLOGY**			
10:00 AM	HP01	Loni Albrecht	Hematological Parameters Do Not Correlate to Survival in Dogs with Multiple Acquired Portosystemic Shunts (ACVIM Resident Research Award Eligible)
10:15 AM	HP02	Andrea Johnston	Liver Shear Wave Elastography Validated by Hepatic Histology in Normal Cats
10:30 AM	HP03	Itsuma Nagao	Canine Patient‐derived Gallbladder Organoids for Personalized Gallbladder Mucocele Modeling and Drug Screening
10:45 AM	HP04	Adrian Tinoco Najera	Serial Quantification of Serum MicroRNA 122 in Dogs with Acute Liver Injury
**SMALL ANIMAL INTERNAL MEDICINE ‐ INFECTIOUS DISEASE**			
3:00 PM	ID01	Skyler Caldwell	Canine Cholecystitis Secondary to Hammondia spp. Tachyzoite Infection (ACVIM Resident Research Award Eligible)
3:15 PM	ID02	Jeff Bender	Detection of Carbapenemase‐producing E. coli through a Veterinary‐Public Health Surveillance Partnership — Minnesota, 2022
3:30 PM	ID03	Meg Nakazawa	Enterohemorrhagic Escherichia coli Reduces Mucus and Barrier Integrity in Translational Canine Colonoid‐Derived Monolayers
3:45 PM	ID04	Erin Lashnits	Flea‐borne Pathogens in Fleas from Naturally Infested Dogs and Cats in Private Homes in Florida
4:30 PM	ID05	Christian Leutenegger	Frequency of Intestinal Parasites in Dogs and Cats Identified by Molecular Diagnostics
4:45 PM	ID06	Beth Licitra	Sites of Feline Coronavirus Persistence in Cats
5:00 PM	ID07	JoAnn Morrison	Outcomes of Naturally Occurring Canine Heartworm Infection
5:15 PM	ID08	Rodolfo Oliveira Leal	Can We Predict Xanthinuria in Dogs with Leishmaniasis Undergoing Allopurinol Treatment? – a Retrospective Study
5:30 PM	ID09	Víctor Oppenheimer Lúgaro	Trends and Risk Factors for Multidrug‐Resistant Canine Urinary Tract Infections (2009–2013; 2017–2021)
5:45 PM	ID10	Madeleine Stein	Use of an Online Questionnaire to Monitor the Success of a Hospital Antimicrobial Stewardship Program (ACVIM Resident Research Award Eligible)
**SMALL ANIMAL INTERNAL MEDICINE ‐ NEPHROLOGY/UROLOGY**			
1:45 PM	NU01	Eleanor Brown	Feline Urine Ammonia‐to‐creatinine Ratio Reference Interval (ACVIM Resident Research Award Eligible)
2:00 PM	NU02	Eleanor Brown	Correlation of Ammonia Excretion with Renal Function and Serum Bicarbonate in Cats with Kidney Disease (ACVIM Resident Research Award Eligible)
2:15 PM	NU03	Colleen Bourque	Complications Associated with Percutaneous Ultrasound‐guided Renal Biopsy in 51 Dogs
2:30 PM	NU04	Hilla Chen	Comprehensive Assessment of Hemostasis in Dogs with Acute Kidney Injury and Chronic Kidney Disease
**EQUINE**			
1:45 PM	E13	Paula Viviani	Seasonal Fluctuations in Baseline Adrenocorticotrophic Hormone and Insulin Levels in Healthy Horses in Saskatchewan (ACVIM Resident Research Award Eligible)
2:00 PM	E35	Carolyn Arnold	Functional Profiling of the Fecal Microbiome of Healthy Horses and those with Colitis
2:15 PM	E45	Cynthia Xue	Two Manual Noncommercial Methods of Platelet‐Rich Plasma Preparation in Donkeys (Equus asinus)
2:30 PM	E47	Cassandra Baumgarten	The Acute Phase Response of Serum Amyloid A After Routine Vaccination in Healthy Adult Horses
10:00 AM	E49	Rosalie Fortin‐Trahan	Investigation of Vitamin D Concentrations in Healthy Horses and Horses with Colitis (ACVIM Resident Research Award Eligible)
10:15 AM	E50	Camilo Jaramillo‐Morales	Biosurveillance of Streptococcus Equi Subsp. Equi in Nasal Secretions of 9,409 Equids Across the USA (ACVIM Resident Research Award Eligible)
3:00 PM	E61	Daniel Jean	Nephrolithiasis in Horses: a Retrospective Study of 25 Cases (2007–2021)
11:15 AM	E62	Carla Enriquez	Evaluation of Real‐time PCR for the Identification of Sarcocystis neurona in Equine Cerebrospinal Fluid (ACVIM Resident Research Award Eligible)
11:30 AM	E63	Megan Palmisano	8‐hydroxy‐2’‐deoxyguanosine as a Potential Marker of Oxidative Damage in Horses with Neuroaxonal Degeneration (ACVIM Resident Research Award Eligible)
11:45 AM	E64	Clarisa Romero Hernandez	Utility of Phosphorylated Neurofilament‐heavy (pNF‐H) Assay in the Diagnosis of Equine Neurologic Disease (ACVIM Resident Research Award Eligible)
3:15 PM	E67	Elaine Norton	The Effect of Leucine and Pyridoxine on Metabolic Parameters in Ponies with Equine Metabolic Syndrome
3:30 PM	E69	camilla Quattrini	Evaluation of Serum sCD14 Concentration as Biomarker of Illness and Sepsis in Neonatal Thoroughbred Foals
10:30 AM	E73	Camilo Jaramillo‐Morales	Effects of Fluid Resuscitation on Venous Blood Gases and Electrolytes in Critically Ill Neonatal Foals (ACVIM Resident Research Award Eligible)
10:45 AM	E74	Catherine Jula	Single Dose Pharmacokinetics of Pimobendan and O‐deoxymethylpimobendan (ODMP) in Healthy Adult Horses (ACVIM Resident Research Award Eligible)
12:00 PM	E78	Carla Olave	Specialized Pro‐resolving Lipid Mediators Modulate the Effect of Dust Exposure on Airway Neutrophilia in Racehorses (ACVIM Resident Research Award Eligible)
3:45 PM	E79	Laurence Leduc	Severe Equine Asthma Is Associated with Increased Airway Innervation
**FRIDAY, JUNE 16**			
Time		Presenting Author	Abstract Title
**ONCOLOGY**			
2:45 PM	O05	Joelle Fenger	Tolerability Evaluation of Combination Verdinexor (LAVERDIA‐CA1) and Doxorubicin in Tumor‐bearing Dogs
3:00 PM	O06	Jeong‐Hwa Lee	Tumor Necrosis Factor Stimulated Gene 6 Intrinsically Regulates PD‐L1 Expression, Modulating the Breast Cancer Microenvironment.
3:15 PM	O07	Chen‐Si Lin	Kynurenine 3‐monooxygenase and ki67 Are Effective Biomarkers for Canine Cancer Diagnosis
3:30 PM	O08	Nick Parkinson	Genome‐wide CRISPR Knockout Screening Identifies Candidate Molecular Targets for Canine Oral Melanoma
4:30 PM	O09	Allison O'Kell	Patient Outcomes When Liquid Biopsy Was Used as an Aid in Diagnosis for Cancer
4:45 PM	O10	Andi Flory	Evaluation of a Next‐generation Sequencing‐based Liquid Biopsy Test for Cancer Monitoring in Dogs
5:00 PM	O11	Emily Rawlings	Neutropenia‐induced Adverse Events Following CCNU with or Without Prophylactic Antibiotics in Dogs
5:15 PM	O12	Aryana Razmara	Single‐Cell Analysis of Natural Killer Cells in Response to Immunotherapy in First‐in‐Dog Clinical Trials
5:30 PM	O13	Lucas Rodrigues	Clinico‐genomics Analysis of Canine Anal Sac Adenocarcinoma: Genomic Profile and Treatment Opportunity
5:45 PM	O14	William Hendricks	Mutation Landscape and Clinical Actionability in Canine Osteosarcoma
**SMALL ANIMAL INTERNAL MEDICINE ‐ GASTROENTEROLOGY**			
8:00 AM	GI13	Holly Ganz	Five Years of Insights from a Fecal Stool Bank for Domestic Cats and Dogs
8:15 AM	GI14	Romy Heilmann	Serum Concentrations of Calprotectin, S100A12, and alpha1‐proteinase Inhibitor in Dogs with Pancreatitis
8:30 AM	GI15	Romy Heilmann	Dysregulation of the Renin‐angiotensin‐aldosterone System (RAAS) and Dependent Electrolyte Transporters in Canine Chronic Inflammatory Enteropathy
8:45 AM	GI16	Molly Doyle	Efficacy of Two Colonoscopy Preparation Protocols in Dogs
9:15 AM	GI17	Aarti Kathrani	A Transfer Learning Approach to Intestinal Image Analysis Differentiates Treatment Response in Canine Protein‐losing Enteropathy
9:30 AM	GI18	Peter Kook	Standardized Intestinal Ultrasonography in Cats with Inflammatory Bowel Disease (IBD) and Small Cell Lymphoma (SCL)
9:45 AM	GI19	Militsa Pacheva	Effects of Prednisolone on DGGR‐Lipase Activity (LIPC Roche) and Pancreatic Lipase Immunoreactivity (SpecfPL) in Cats
10:00 AM	GI20	Mary Ann Lee	Adverse Events Following Repeat Fecal Microbiota Transplantation in Cats: A Case Series
11:00 AM	GI21	Mary Ann Lee	Assessment of Safety, Adverse Events, and Dysbiosis in Healthy Dogs Receiving Fecal Microbiota Transplantation
11:15 AM	GI22	Brandon Mason	Western Diet Promotes Bile Acid Dysmetabolism, Pro‐carcinogenic Inflammation, and Colonic Dysbiosis in Healthy, Non‐obese Dogs
11:30 AM	GI23	Sydney Oberholtzer	Prognostic Value of C‐reactive Protein in Dogs with Elevated Pancreas‐specific Lipase Reactivity
11:45 AM	GI24	Yeon Joon Park	A Retrospective Evaluation of Serum Symmetric Dimethylarginine Concentration in Dogs with Protein Losing Enteropathy
1:30 PM	GI25	Giacomo Rossi	Possible Role Of Dietary N‐glycolyneuraminic Acid And Dysbiosis In Canine Enteropathy Pathogenesis
1:45 PM	GI26	Elisa Scarsella	Blood Microbiome in Dogs with Chronic Enteropathies: The Future of Prevention and Diagnosis?
2:45 PM	GI27	Sarah Schmid	Occurrence of Owner‐reported Gastrointestinal Disease in 33,172 Dogs in the Dog Aging Project Pack
2:15 PM	GI28	Megan Slaughter	Use of a Feline Dysbiosis Index to Monitor a Probiotic's Effects on Antibiotic‐Induced Dysbiosis
2:00 PM	GI29	Stacie Summers	Effect of Wheat Dextrin on Fecal Short‐Chain Fatty Acids in Dogs with Chronic Diarrhea
3:00 PM	GI30	Chi‐Hsuan Sung	Correlation between Targeted qPCR‐Assays and Untargeted DNA Shotgun Sequencing for Assessing Fecal Microbiota in Dogs
3:15 PM	GI31	Nashua Waly	Improved Treatment Outcome & Reduced Recovery Time in Faecal Microbiota Transplanted Dogs with Parvovirus Disease
3:30 PM	GI32	Yu‐An Wu	Immunohistochemical Characterization of Lymphocytic Infiltrates in Pancreata from Shelter Cats
**SMALL ANIMAL INTERNAL MEDICINE ‐ IMMUNOLOGY**			
1:30 PM	IM01	Michael Lappin	Effect of Feeding Enterococcus Faecium Strain SF68 on Leukocyte Transcriptomic Responses in Healthy Cats
1:45 PM	IM02	Sarah Schmid	Pilot Evaluation of Inflammatory Markers in Aging Dogs
**SMALL ANIMAL INTERNAL MEDICINE ‐ NEPHROLOGY/UROLOGY**			
4:30 PM	NU05	Emily Coffey	The Impact of Nutrition on the Urobiome of 15 Clinically Healthy Adult Dogs
4:45 PM	NU06	Karina Creighton	Use of a Bipolar Resectoscope for Debulking of Obstructive Urethral Masses in Dogs
5:00 PM	NU07	Cailin Harro	The Effect of Circumferential Tape Applied to the Cuff During Blood Pressure Measurement in Cats
5:15 PM	NU08	David Haas	Evaluation of Urine Dipstick protein:urine Specific Gravity Ratio as a Test of Proteinuria in Dogs
5:30 PM	NU09	Jillian Yant	Proteinuria in Dogs and Cats with Presumed and Confirmed Urogenital Neoplasia
5:45 PM	NU10	Stephanie Harris	Urinary syndecan‐1 in Dogs Anesthetized with Isoflurane or Sevoflurane: A Randomized, Prospective Study
**SMALL ANIMAL INTERNAL MEDICINE ‐ OTHER**			
4:30 PM	OT01	Ellen Ratcliff	Cross‐sectional Investigation of Potential Biomarkers of Aging in Dogs
4:45 PM	OT02	Lindsey Summers	Clinical Findings, Treatment Recommendations, and Outcome in Dogs with Known Trilostane Toxicity: 28 Cases (2005–2020)
5:00 PM	OT03	Jennifer Adler	Development, Implementation and Impact of an I‐PASS Based Patient Handoff Initiative
5:15 PM	OT04	Jennifer Adler	Development and Implementation of a WHO‐derived Checklist Initiative
**SMALL ANIMAL INTERNAL MEDICINE ‐ PHARMACOLOGY**			
2:00 PM	P01	Kristen Messenger	Pharmacokinetics of Subcutaneous Maropitant Alone and Combined with a Subcutaneous Fluid Bolus in Healthy Dogs
**SMALL ANIMAL INTERNAL MEDICINE ‐ RESPIRATORY**			
2:45 PM	R01	Charlotte Gerhard	Objective Assessment of Computed Tomographic Attenuation in Dogs with and Without Bronchomalacia (ACVIM Resident Research Award Eligible)
3:00 PM	R02	Mireia Pascual Moreno	Clinical, Imaging and Rhinoscopy Findings in 63 Dogs and 8 Cats with Nasal Foreign Body
3:15 PM	R03	Julia Remaks	Using Probiotics to Modulate the Respiratory Microbiome in Feline Allergic Asthma (ACVIM Resident Research Award Eligible)
3:30 PM	R04	Ashlynne Wampfler	Retrospective Evaluation of High Velocity Nasal Insufflation in Cats with Respiratory Disease

**Research Abstract ePoster Program**

**Index of Abstracts**

**Table:** 

**THURSDAY, JUNE 15**			
Time	#	Presenting Author	Abstract Title
**CARDIOLOGY**			
4:05 PM	C10	Emily Herrold	Effects of Pimobendan on Left Atrial Function in Cats with Left Atrial Enlargement and Dysfunction (ACVIM Resident Research Award Eligible & Cardiology Research Abstract Award Eligible)
4:05 PM	C27	Lindsey Humphries	Short‐Term Hemodynamic Effects of Pimobendan in Dogs with Preclinical Patent Ductus Arteriosus
4:05 PM	C28	Nozomi Shiohara	Feasibility of Shear Wave Elastography/Dispersion Imaging in Dogs with Cardiac Disease
6:00 PM	C29	Katherine Murphy	Agreement of Echocardiographic‐ and Catheterization‐based Methods of Transpulmonary Pressure Gradient Measurement in Dogs
4:05 PM	C30	Victor Rivas	Multiomic, Histopathologic, and Clinicopathologic Assessment of Chronic, Weekly, Oral Rapamycin Administration in HCM‐Affected Cats
4:05 PM	C32	William Muir	The Cardiovascular Effects of Zenalpha in Ventricular Tachy‐paced Dogs in Mild to Moderate Heart Failure
6:00 PM	C34	Kentaro Kurogochi	Association between Mitral Insufficiency Echocardiographic (MINE) Score and Mitral Valve Repair Outcome
4:05 PM	C37	Jae Chun Cho	Loop‐mediated Isothermal Amplification PCR in Place of Modified Knott's Test Screening Dogs for Heartworm Infection
4:05 PM	C38	Minseok Choi	Serum Angiopoietin‐1 and Angiopoietin‐2 of Dogs with Myxomatous Mtension
4:05 PM	C39	Lisa Freeman	Red Blood Cell Morphologic Parameters and Indices in Dogs with Dilated Cardiomyopathy Differs Between Diets
6:00 PM	C40	Lily Coppinger	Comparison of Electrocardiographic and Echocardiographic Findings in Irish Wolfhounds Eating Non‐Traditional or Traditional Diets
**NEUROLOGY**			
9:25 AM	N17	Kayla Fowler	Evaluation of Neurofilament Light Chain as a Biomarker in Dogs with Structural and Idiopathic Epilepsy (ACVIM Resident Research Award Eligible)
9:45 AM	N18	Jeffrey Laifer	Serum 25‐hydroxyvitamin D3 Concentrations in Dogs with Non‐infectious Meningoencephalitis (ACVIM Resident Research Award Eligible)
9:25 AM	N19	Brittany MacQuiddy	A Minimally Invasive Surgical Technique to Hemilaminectomies in Chondrodystrophic Dogs with Thoracolumbar Intervertebral Disc Extrusions (ACVIM Resident Research Award Eligible)
9:45 AM	N20	Ruby Ng	Analgesic and Anti‐inflammatory Effects of Lidocaine in Dogs Undergoing Thoracolumbar Hemilaminectomy for Intervertebral Disc Extrusion (ACVIM Resident Research Award Eligible)
9:25 AM	N21	Aaron Rozental	Characterization of Endocannabinoids in the CSF and Serum of Dogs with Idiopathic Epilepsy (ACVIM Resident Research Award Eligible)
9:45 AM	N22	Josefa Garcia	The T2‐FLAIR Mismatch Sign as an Imaging Biomarker for Canine Oligodendrogliomas
9:25 AM	N23	Kari Foss	Disposition and Bioequivalence of Single‐dose Compounded and Commercial Extended Release Levetiracetam in Healthy Dogs
9:45 AM	N25	Minju Baek	Neurofilament Light Chain as a Biomarker for Distinguishing IE from Structural Brain Diseases in Dogs
6:00 PM	N26	Gilad Fefer	Neurological Examination and Magnetic Resonance Imaging Findings in Dogs Diagnosed with Central Vestibular Disease
9:25 AM	N27	Grace Kadler	Viability of Using Diffusion Tensor Imaging to Detect Structural Abnormalities in Dogs with Idiopathic Epilepsy
9:45 AM	N28	Marios Charalambous	The Impact of Rescue Medication in Dogs with Emergency Seizure Disorders: An Owner Perspective
9:25 AM	N29	Dohee Lee	Evaluation of Inflammatory Mediators and Hematologic Parameters in Canine Pentylenetetrazole Induced Seizure Model
9:45 AM	N30	Yelim Lee	Serum Levels of Complement C3 and C4 in Dogs with Idiopathic Epilepsy
6:00 PM	N31	Léa Monneret	Long‐term Effect of Imepitoin Treatment in Dogs with Epilepsy
9:25 AM	N32	Su‐Min Park	Deferoxamine‐preconditioned cAT‐msc‐derived Extracellular Vesicles Alleviate Inflammation in an EAE Mouse Model Through STAT3 Regulation
9:45 AM	N33	Casey Rogers	Gluten Associated Antibody Assessment in Diverse Dog Breeds with Suspected Paroxysmal Dyskinesia
9:25 AM	N34	Woo‐Jin Song	Clinical Trial of Crisdesalazine (GedaCure) on Meningioencephalitis in Dogs
9:45 AM	N35	Taesik Yun	Neurofilament Light Chain as a Biomarker of Spinal Cord Diseases in Dogs
**NUTRITION**			
6:05 PM	NM05	Hailey Rose	Serum Vitamin D Metabolites and Acute‐Phase Proteins in Critically Ill Dogs and Cats (ACVIM Resident Research Award Eligible)
6:25 PM	NM06	Julie Spears	Differential Digestive Health Benefits of Fiber Blends Fed to Healthy Adult Cats
6:45 PM	NM07	Julie Spears	Differential Digestive Health Benefits of Fiber Blends Fed to Healthy Adult Dogs
6:05 PM	NM08	Julie Spears	Evaluation of Pumpkin and Inulin Using Canine and Feline‐Specific Intestinal In Vitro Fermentation Assays
6:25 PM	NM09	Julie Spears	Supplementation of Probiotics with Psyllium Influences the Gut Microbiome Through Microbial Diversity Alteration in Dogs
9:45 AM	NM10	Isabella Corsato Alvarenga	The Tolerability of Supplementing CBD at Two Doses for 9 Months to Healthy Dogs
6:45 PM	NM13	Selena Tavener	Circulating miRNAs Show an Anti‐inflammatory Profile in Healthy Canines Fed Low Carbohydrate Ketogenic Foods
**ONCOLOGY**			
6:05 PM	O15	Jennifer Lenz	Osteopontin Overexpression in Localized Canine Histiocytic Sarcoma
6:00 PM	O16	Corey Saba	Alternating Rabacfosadine and Doxorubicin for Treatment of Naive Canine Lymphoma
6:25 PM	O17	Hiroki Sakuma	Gene Expression Profiles Associated with Sensitivity to Molecular‐Targeted Drugs in Canine Histiocytic Sarcoma Cells
6:45 PM	O19	Yong Bin Teoh	Genome‐wide DNA Methylation Analysis Identified Prognostic CPG Site Markers in Canine Multicentric High‐grade Lymphoma
6:00 PM	O20	Hiroto Toyoda	Gene Expression Profiles Associated with Early Relapse in Canine Multicentric High‐grade B‐cell Lymphoma
6:00 PM	O21	Guannan Wang	Genomic Analysis across 50 Canine Cancer Types Reveals Novel Mutations and High Clinical Actionability
6:00 PM	O23	Hwayoung Youn	The Diagnostic Value of Thymidine Kinase 1 Auto‐antibody in Canine Cancer Patients
6:00 PM	O24	William Hendricks	Novel Genomic Prognostic Biomarkers for Canine Cancer Patients
6:05 PM	O25	Chen‐Si Lin	Assessing the Efficacy of Novel Caninized VEGF Monoclonal Antibody Therapy for Mammary Tumors in Dogs
6:25 PM	O26	Ju‐Hyun An	Anticancer Effect of Superoxide Dismutase on Canine Mammary Gland Tumor in Vitro
6:45 PM	O27	Ju‐Hyun An	Urinary Exosomal Nuclear Matrix protein‐22 as a Novel Liquid Biopsy of Urothelial Carcinoma Dogs
6:05 PM	O28	Ashleigh Cournoyer	Phenotypic Description of T‐cell Density in the Tumor Microenvironment of Canine Renal Cell Carcinoma
6:00 PM	O29	Kelly Hume	BTK and BET Inhibition in Canine Lymphoma
6:25 PM	O30	Heejung Hong	A Retrospective Study on Conventional Transarterial Chemoembolization using Idarubicin for Treatment of Canine Hepatic Carcinomas
6:45 PM	O31	Jin‐Young Kim	Usefulness of Computed Tomography in Combination with Iodized Oil (Lipiodol) in Detecting Sentinel Lymph Nodes
6:00 PM	O35	Ga‐Hyun Lim	Macrophage Induced Chemoresistance in Canine Mammary Gland Tumor Cell Spheroid Model
			
**SMALL ANIMAL INTERNAL MEDICINE ‐ ENDOCRINOLOGY**			
12:25 PM	EN17	Julieann Vose	The Role of Gastrin in Dogs with Spontaneous Primary Hyperparathyroidism (ACVIM Resident Research Award Eligible)
12:45 PM	EN18	Michael Merkhassine	Effects of Glucagon Infusion on the Canine Plasma Metabolome (ACVIM Resident Research Award Eligible)
1:05 PM	EN19	JeongYeol Bae	Sialocele and Their Association with Hyperadrenocorticism and Long‐term Glucocorticoid Treatment in dogs: retrospective Case‐control Study
1:25 PM	EN20	Sijin Cha	A Retrospective Study of Neutrophil‐to‐lymphocyte and Platelet‐to‐lymphocyte Ratios as Potential Markers in Dogs with Hypercortisolism
12:25 PM	EN21	Yeon Chae	Relationship between Serum Concentration of Canine Pancreatic Lipase Immunoreactivity and Hyperglycemia in Diabetic Dogs
12:45 PM	EN22	Drew Narwold	Clinical Evaluation of a Point‐of‐Care Free Thyroxine Assay to Diagnose Canine Hypothyroidism
1:05 PM	EN23	Álan Pöppl	What Can Be Learned from Studying Plasma Lactate in Dogs with Naturally Occurring Hypercortisolism?
1:25 PM	EN24	Álan Pöppl	Evaluation of a Recombinant Porcine Corticotropin for the ACTH Stimulation Test in Healthy Cats
12:25 PM	EN25	Thomas Schermerhorn	Validation of a Copeptin Assay in Normal Dogs
12:45 PM	EN26	Woo‐Jin Song	Relationship Between Post ACTH Cortisol Concentration at Diagnosis and Dose of Trilostane in Small‐breed Dogs
1:05 PM	EN27	Samuel Stewart	New Generation Device for Blood Glucose Monitoring in Dogs
1:25 PM	EN28	Fabio Teixeira	Comparing Non‐Esterified Fatty Acids Between Diabetic Dogs and Healthy Dogs and Possible Omega‐3 Effects
**SMALL ANIMAL INTERNAL MEDICINE ‐ GASTROENTEROLOGY**			
12:25 PM	GI33	Michelle Riehm	Plasma Glucagon‐like Peptide‐2 in Dogs with Chronic Enteropathies (ACVIM Resident Research Award Eligible)
12:45 PM	GI34	Dohee Lee	Serum Vitamin D, Its Receptor, and Binding Protein Concentrations in Dogs with Acute Pancreatitis
1:05 PM	GI35	Jeongmin Lee	The Risk of Hypomagnesemia with Long‐term Use of Proton‐pump Inhibitor in Dogs
6:00 PM	GI36	Rae McAtee	Esomeprazole with and without Probiotics in Healthy Dogs (ACVIM Resident Research Award Eligible)
1:25 PM	GI37	nashwa waly	Effect of Short‐Term Dietary Change on Anemia and Diarrhea in Dogs Previously Fed Inadequate Diet
6:00 PM	GI38	Jan Suchodolski	Evaluation of Variation in Fecal Microbiota and Bile Acids over Time in Healthy Pet Cats
**SMALL ANIMAL INTERNAL MEDICINE ‐ HEMATOLOGY**			
1:05 PM	HM07	Jennifer Weng	Defining the Platelet Transcriptome in Dogs (ACVIM Resident Research Award Eligible)
1:25 PM	HM08	Amanda Garrick	Comparison of C‐reactive Protein Measurement on Three Assays and Following Storage
12:25 PM	HM09	Erin Phillips	Validation of the Calibrated Automated Thrombogram Using Low Plasma Volumes in Dogs
12:45 PM	HM10	Joonghyun Song	Effectiveness of Clopidogrel in Preventing Post‐operative Hypercoagulability in Dogs Undergoing Splenectomy for Splenic Masses
1:05 PM	HM11	Chia‐Chen Wu	Assessing the Reliability of Blood Samples Collected from Peripheral Intravenous Catheters
6:00 PM	HM12	Arom Cho	Nucleated Rbcs as a Diagnostic and Prognostic Biomarker in Canine Systemic Inflammatory Response Syndrome
6:00 PM	HM13	Cory Penn	The Evaluation of the VETSCAN IMAGYST Artificial Intelligence (AI) Blood Smear Polychromatophil Determination
**SMALL ANIMAL INTERNAL MEDICINE ‐ HEPATOLOGY**			
12:25 PM	HP05	Rommaneeya Leela‐arporn	Defining the Liver Transcriptome in Cats Using Total RNA sequencing: A Pilot Study (ACVIM Resident Research Award Eligible)
6:00 PM	HP06	Sara Jablonski	Concurrent Hepatopathy in Dogs with Gallbladder Mucocele: Prevalence, Predictors, and Impact on Long‐term Outcome
12:45 PM	HP07	Fabio Teixeira	Correlation of Ultrasound Measures with Liver Volume Assessed by Water Displacement in Dogs
6:00 PM	HP08	Erin Jackson	Comparison of 2‐dimensional Imaging to 3‐dimensional Modeling of Intrahepatic Portosystemic Shunts Using Computed Tomography Angiography
**SMALL ANIMAL INTERNAL MEDICINE ‐ INFECTIOUS DISEASE**			
12:25 PM	ID11	Petra Cerna	Transcriptome Analysis of Peritoneal Effusions in Healthy Cats and Cats with Feline Infectious Peritonitis (ACVIM Resident Research Award Eligible)
12:45 PM	ID12	Andrzej Ogrodny	Multi‐institutional Retrospective Study Investigating Blood Culture Protocols and Test Positivity in 701 Dogs (ACVIM Resident Research Award Eligible)
1:05 PM	ID13	Fernanda Amorim da Costa	Investigation of Anti ‐ Leptospira spp. Antibodies and Leptospiruria in Cats
1:25 PM	ID14	Fernanda Amorim da Costa	Mycoplasma Species and Immunosuppressive Viruses in Cats Affected by Oral Cavity Lesions
6:00 PM	ID15	Yoko Nagamori	Field Evaluation of VETSCAN IMAGYST Artificial Intelligence (AI) Fecal Canine and Feline Parasite Detection
12:25 PM	ID17	Aaren Glick	Performance of C‐reactive Protein and Haptoglobin Concentrations to Detect Remission in Dogs with Pulmonary Coccidioidomycosis
6:00 PM	ID18	Haley Uustal	Associations Between Physical and Mental Health and Exposure to Bartonella henselae in Small Animal Veterinarians
**SMALL ANIMAL INTERNAL MEDICINE ‐ NEPHROLOGY/UROLOGY**			
12:25 PM	NU11	Maxime Derré	Effect of Iodinated Non‐ionic Contrast on Cultures of Canine Urine Inoculated with E. coli. (ACVIM Resident Research Award Eligible)
12:45 PM	NU12	Allison Kendall	Low‐dose Radiation Therapy: A Novel Treatment for Feline Idiopathic/interstitial Cystitis
6:00 PM	NU13	Geoffrey Neumann	Placement of an Artificial Urethral Sphincter in 6 Male Dogs with Urethral Diverticulum
6:00 PM	NU14	Maria Chiara Sabetti	Neutrophil Gelatinase‐Associated Lipocalin as Biomarker of Acute Kidney Injury in Dogs with Congestive Heart Failure
1:05 PM	NU15	Tanner Slead	Urinary Incontinence in Male Dogs
6:00 PM	NU16	Pak Kan Tang	A Pilot Study to Identify Plasma Calciprotein Particles in Cats with Chronic Kidney Disease
6:00 PM	NU17	Tim Williams	Urinary Extracellular Vesicle Excretion Rate and Uromodulin Abundance in Cats with Calcium Oxalate Urolithiasis
1:25 PM	NU18	Karina Creighton	Use of a Pediatric Renal Replacement System (CARPEDIEM) for Extracorporeal Therapy in Dogs
**SMALL ANIMAL INTERNAL MEDICINE ‐ OTHER**			
1:05 PM	OT05	Fernanda Amorim da Costa	A Restrospective Study of Venom Animals Accidents in Cats in South Brazil
6:00 PM	OT07	Kristin Owens	Deep Learning Artificial‐intelligence (AI) Based Approach for Evaluation of Canine and Feline Dermatologic Cytology Samples
1:25 PM	OT08	Elizabeth Pearson	Evaluation of Factors Associated with Euthanasia versus Unassisted Death in the Dog Aging Project Pack
**SMALL ANIMAL INTERNAL MEDICINE ‐ PHARMACOLOGY**			
12:45 PM	R05	Fernanda Amorim da Costa	Causes of Pleural Effusion in 67 Cat ‐ A Retrospective Study
1:05 PM	R06	Fernanda Amorim da Costa	Chronic Rhinosinusitis in Cats – An Etiological Study
1:25 PM	R07	Zhe (Alice) Wang	Comparing the Nasal Transcriptome and Microbiome in Healthy Dogs and Dogs with Chronic Rhinitis
**EQUINE**			
6:00 PM	E02	Persephone McCrae	A Smart Textile Device Can Accurately Capture Heart Rate Variability in Resting Horses: Preliminary Results
6:00 PM	E03	Laura Nath	Histopathological Evaluation of Myocardial Hypertrophy and Fibrosis in Thoroughbred Racehorses
6:00 PM	E04	Cris Navas de Solis	Exercising Electrocardiograms in Eventing Horses in the Fieldg
6:00 PM	E05	Kaitlin Murphy	Association of DFA Alpha 1 with Blood Lactate Measurements in Exercise Tests in Sport Horses
6:00 PM	E06	Isabelle Piotrowski	Evaluation of the Audicor Device as Diagnostic Tool in Horses with Mitral or Aortic Regurgitation
6:05 PM	E07	Freya Stein	Echocardiographic Measurements and Findings in Healthy Standardbred and Thoroughbred Racehorses
6:25 PM	E08	Glenn Van Steenkiste	In Silico simulations for Efficacy and Risk of Transvenous Electrical Cardioversion Catheter Positions in Horses
6:45 PM	E09	Ingrid Vernemmen	Transseptal Puncture in the Horse Using Intracardiac Ultrasound Guidance: First Results with Follow‐Up
6:05 PM	E14	Amina Ayubi	Enteroinsular Axis in Healthy Horses and Horses with Colitis
6:00 PM	E15	Dante Vorster	Effect of Early and Late Sampling on the 10‐ and 30‐Minute Thyrotropin‐Releasing Hormone Stimulation Test
6:25 PM	E16	Melody de Laat	Glucagon‐like peptide‐1 Is Produced by the Equine Large Intestine in vitro
6:00 PM	E17	Isabelle Desjardins	Monthly Variations of a Clinical Pituitary Pars‐intermedia Dysfunction Score and TRH Stimulation Test in Ponies
6:00 PM	E18	Isabelle Desjardins	Comparison of Hair Characteristics in Equids Affected with Pituitary Pars Intermedia Dysfunction and Aged Controls
6:00 PM	E20	Nicolas Galinelli	Effect of Pergolide in Horses with Insulin Dysregulation with and without Pituitary Pars Intermedia Dysfunction
6:05 PM	E22	Ahmed Kamr	Serum Biomarkers of Bone Remodeling in Horses with Colitis
6:25 PM	E23	Ahmed Kamr	Vitamin D Metabolites and Antimicrobial Peptides in Hospitalized Foals
6:45 PM	E24	Ahmed Kamr	The Bone‐Pancreas Loop and Incretins in Hospitalized Foals
6:05 PM	E25	Kate Kemp	Phenylbutazone Administration Does Not Affect Insulin Sensitivity in Horses with Insulin Dysregulation
6:25 PM	E26	Kate Kemp	Effects of Phenylbutazone Administration on Insulin Secretion in Horses with Insulin Dysregulation
6:00 PM	E27	Rachel Lemcke	Prevalence of Pituitary Pars Intermedia Dysfunction and Insulin Dysregulation in Horse Breeds Classified by Clade
6:00 PM	E28	Rachel Lemcke	Exploring Endocrine Disorders in Sporthorses from Different Geographic Origins
6:45 PM	E30	Kristen Thane	Comparison of High‐dose and Low‐dose Oral Sugar Test Performance in Horses
6:05 PM	E31	Kristen Thane	Comparison of Wellness Readytm Lateral‐flow Insulin Assay to Radioimmunoassay Insulin Results in Equine Blood Samples
6:45 PM	E32	Katarzyna Dembek	Abnormal Incretin Concentration in Equine Colic
6:00 PM	E33	Steve Grubbs	Diagnosis of Equine Pituitary Pars Intermedia Dysfunction using TRH‐Stimulation is Repeatable at One‐Week Intervals
6:00 PM	E34	Steve Grubbs	Resting ACTH versus Dynamic ACTH Evaluation for PPID During Fall and Spring in Horses
6:00 PM	E36	Daniel Jean	Endoscopic Digestive Biopsies in Horses: A Retrospective Study of 36 Cases (2017–2021)
6:00 PM	E37	Leonardo Parra	Blood and Peritoneal Lactate as a Marker of Intestinal Ischemia in Horses with Colic
6:00 PM	E38	Breanna Sheahan	Manipulating Epithelial Fluid Secretion Using Equine Rectal Organoids
6:00 PM	E39	Sophie Talbot	Clinical Presentation and Outcome of Gastric Impactions with or Without Concurrent Intestinal Lesions in Horses
6:00 PM	E40	Jessica Vokes	The Effect of Medium‐term Omeprazole on Serum Gastrin and Chromogranin a Levels in the Horse
6:00 PM	E43	Diego Gomez	Endothelial Glycocalyx Integrity in Horses with Colitis
6:00 PM	E44	Pablo Fueyo	Prognostic Value of Red Blood Cell Distribution Width, and Other Hematological in Horses with Colitis
6:45 PM	E46	Ina Mersich	Impact of Decreased Circulating Red Cell Mass on Viscoelastic Coagulation Results in Healthy Horses
6:00 PM	E51	Diego Gomez	Endothelial Glycocalyx Integrity in Healthy and Septic Foals
6:00 PM	E52	Nicole Kreutzfeldt	Antibody Response to Equine Influenza Virus and herpesvirus‐1 Vaccine in Adult Horses Receiving Intravenous Dexamethasone
6:00 PM	E54	Edward Olajide	Detection of Potential Latency Sites of Equid Alphaherpesvirus 1 (EHV‐1) in Kentucky Horses
6:00 PM	E55	Erin Pearson	Association of Borrelia burgdorferi with Cranial Nuchal Bursitis and Elevated Ospa‐specific Serum Antibodies in Horses
6:00 PM	E57	Alexandre Borges	Fecal QPCR Testing for the Detection of Enteric Pathogens in Foals with or Without Diarrhea
6:00 PM	E60	Leonardo Parra	Calcium, Magnesium, and Phosphorus Concentration in Septic Foals
6:00 PM	E65	Sarah Colmer	Cerebrospinal Fluid Cell‐free DNA Concentrations in Horses with Common Neurologic Conditions
6:00 PM	E66	Sarah Colmer	Association of Vitamin E‐responsive Myopathy (VEM) and Equine Degenerative Myeloencephalopathy / Neuroaxonal Dystrophy (EDM/eNAD)
6:00 PM	E68	Kate Hepworth‐Warren	Complications Associated with Intravenous Catheter Placement in Adult Horses at a Teaching Hospital
6:00 PM	E81	Ananya Mahalingam‐Dhingra	Bronchoalveolar Lavage Hemosiderosis in Lightly Active or Sedentary Horses
6:00 PM	E82	Jane Woodrow	Initial Investigation of Bronchoalveolar Lavage Fluid Metabolomics in Healthy and Suspect Asthmatic Horses
**FOOD ANIMAL INTERNAL MEDICINE**			
9:25 AM	F03	Lynna Li	Effect of Sample Collection Time on Fecal Microbiota Composition in Diarrheic Calves
9:45 AM	F05	Hakhyun Kim	Fucoidan Increases Porcine Neutrophil Extracellular Trap Formation Through Tumor Necrosis Factor‐alpha
6:00 PM	F07	Shari Kennedy	Bovine Viral Diarrhea Virus Seroprevalence in Wild Pigs across 17 States
9:25 AM	F08	Mathilde Pas	Comparison of bacteremia Detection and Contamination Between Two Blood Culture Media in Critically Ill Calves
6:00 PM	F10	Blanca Camacho	Validation and Comparison of SDMA in Healthy and Obstructed Goats
6:00 PM	F11	Diana Perez‐Solano	Prognostic Indicators of Outcome for Non‐ambulatory Beef Cattle Presented to Two Referral Hospitals
**FRIDAY, JUNE 16**			
Time		Presenting Author	Abstract Title
**CARDIOLOGY**			
10:25 AM	C35	Edward Daly	Relationship of Serum Osmolality to the Degree of Mathematical Chloride Correction and Heart Disease Stage
10:25 AM	C41	Chaerin Kim	Neutrophil‐to‐Lymphocyte, Monocyte‐to‐Lymphocyte, and Platelet‐to‐Lymphocyte Ratios as Inflammatory Biomarkers in Dogs with Myxomatous Mitral Valve Disease
10:45 AM	C42	Jiyoung Kim	Diagnostic Value of Pulmonary Vein to Pulmonary Artery Ratio Measurement in Dogs with MMVD
10:25 AM	C43	Kentaro Kurogochi	Comparison of the Clinical Utility of Del‐Nido Cardioplegia and St. Thomas Solution with Cardiac Troponin‐I
10:45 AM	C44	Elizabeth Martin	A Novel Nonsynonymous Gene Variant in IDUA in Basset Hounds with Mucopolysaccharidosis Type 1
10:25 AM	C45	Dmitrii Oleynikov	Retrospective Analyze of Bosentan Treatment in Dogs with Severe Pulmonary Hypertension
10:45 AM	C46	Paulo Rivera	Vascular Responsiveness to Pimobendan as Assessed by Doppler Ultrasonography in Dogs with Congestive Heart Failure
10:25 AM	C47	Yunji Kim	Machine Learning‐based Risk Prediction for Canine Myxomatous Mitral Valve Disease Using Electronic Health Record Data
10:45 AM	C48	Tess Sykes	Echocardiographic Findings and Prevalence of Heart Murmurs in Clinically Healthy Adult Borzoi Dogs
**NUTRITION**			
12:30 PM	NM11	Ana Rita Pereira	Comparison of Microorganisms Concentration of Brazilian Probiotics and Doses Recommendation for Canine Enteropathies
12:50 PM	NM12	Darcy Adin	Enzyme‐linked Immunosorbent Assay for the Detection of Phytohemagglutinin in Legumes
**ONCOLOGY**			
10:25 AM	O32	Yeji Kim	Transcriptome Profiling Between Normal and Lymphoma in Comparison of T‐cell and B‐cell Types in Dogs
10:25 AM	O33	Amy LeBlanc	The National Cancer Institute's Comparative Osteosarcoma Pathology Board: Toward Harmonized Grading for Humans and Dogs
10:45 AM	O34	Amy LeBlanc	The National Cancer Institute's Decoding the Osteosarcoma Genome of the Dog (DOG2) Project
10:45 AM	O36	Woo‐Jin Song	Clinical Outcome of Multicentric Lymphoma Treated with CHOP Protocol in 38 Small‐breed Dogs
10:25 AM	O38	Kelley Zimmerman	Evaluation of Mechlorethamine, Vinblastine, Procarbazine, and Prednisone for Treatment of Resistant Multicentric Canine Lymphoma
**SMALL ANIMAL INTERNAL MEDICINE ‐ IMMUNOLOGY**			
12:10 PM	IM03	Regina Hollar	Role of CD8+ T‐cells and Immune Activation in Pathogenesis of Inflammatory Bowel Disorder in Canines
12:30 PM	IM04	Jungil Kim	Comparison of Prednisolone Alone and Prednisolone Combined with Adjunctive Immunosuppressants for Dogs with Immune‐mediated Polyarthritis
12:50 PM	IM05	Laurie Larson	Prevention of Mortality by Canine Parvovirus Monoclonal Antibody Treatment after Experimental Challenge
1:10 PM	IM06	Selena Tavener	Upregulation of SOCS1 and CXCL14 Can Contribute to the Pathogenesis of Hyperthyroidism in Cats
**SMALL ANIMAL INTERNAL MEDICINE ‐ NEPHROLOGY/UROLOGY**			
12:10 PM	NU19	Fernanda Chacar	Urinary Fractional Excretion of Calcium in Cats with Hypercalcemia or Normocalcemia
12:30 PM	NU20	Fernanda Chacar	Urinary Fractional Excretion of Phosphorus in Cats with Chronic Kidney Disease
12:50 PM	NU21	Fernanda Chacar	Traditional Approach versus Stewart Approach for Acid–Base Disorders in Cats with Chronic Kidney Disease
1:10 PM	NU22	Chien‐Hui Chen	Evaluation of Chronic Stress Status in Diseased Cats with Hair Cortisol Concentration and Psychometrics
12:10 PM	NU23	Wei‐Che Chen	Urinary Fibroblast Growth Factor‐23 in Cats with Stable or Acute Decompensated Chronic Kidney Disease
**EQUINE**			
12:10 PM	E29	Amanda Norman	Histological Changes of the Equine Placenta in the Presence of Insulin Dysregulation
12:30 PM	E48	Emily Sundman	Selection of Observation Timepoints with Intradermal Skin Testing in Horses
12:10 PM	E56	Eva‐Christina Schliewert	Investigation of the Inflammatory Response in African Horse Sickness
12:30 PM	E58	Diana Vargas	Prognostic Value of Red Blood Cell Distribution Width and Other Hematological Parameters in Hospitalized Foals
12:50 PM	E59	Kaitlyn McEnaney	Detection of Anaplasma phagocytophilum in Healthy and Febrile Horses in Central Ohio
12:10 PM	E71	Joanne Haughan	Gamma‐glutamyl Transferase Plasma Levels are Associated with Weekly Workload and Racing Status in Thoroughbred Racehorses
12:30 PM	E75	Cosette Faivre	Pharmacokinetics of Repeat Dosing of 40 mg/kg and 60 mg/kg of Acetaminophen in Neonatal Foals
12:50 PM	E76	Joe Smith	A Preliminary Investigation of the Pharmacokinetics of Esomeprazole After Intravenous and Oral Dosing in Horses
1:10 PM	E80	Laurent Couetil	Fish Oil Supplementation Reduces Airway Neutrophilia in Thoroughbred Racehorses

## Abstract C01: Two‐Dimensional Quantitative Methods for Evaluation of Mitral Regurgitation Severity in Canine Degenerative Mitral Valve Disease

### **Bruna Del Nero**; Brianna Potter; Lance Visser; Brian Scansen; E. Christopher Orton

#### Colorado State University, Fort Collins, CO, USA

**Background:** Quantitative assessment of mitral regurgitation (MR) severity has become increasingly important as surgical and catheter‐based therapies for MR reduction become available.

**Hypothesis/Objectives:** Obtain quantitative measures of MR severity across stages of degenerative mitral valve disease (DMVD) and evaluate agreement between two methods.

**Animals:** 51 client‐owned dogs stratified across three stages of DMVD (ACVIM B1, B2, and C).

**Methods:** Regurgitant volume (RVol) and fraction (RF) were measured on echocardiograms prospectively acquired by a single cardiologist by using Simpson's method of discs (volumetric) and proximal isovelocity surface area (PISA) methods. RVol and RF were compared by both methods across disease stage (Kruskall–Wallis, Conover). Agreement between methods over the range of MR severity was determined by Bland‐Altman analyses. Intra‐ and inter‐observer measurement variability assessments were made via intraclass correlation coefficients (ICC).

**Results:** RVol was different (*P* < 0.0001) across stage by both volumetric (B1—0.09 mL/kg; B2—1.49 mL/kg; C—2.72 mL/kg) and PISA (B1—0.39 mL/Kg; B2—1.67 mL/kg; C—3.1 mL/kg) methods. Similarly, RF was different (*P* < 0.0001) across stage by both volumetric (B1—13.6%, B2—55.6%, C—67.2%) and PISA (B1—34.3%, B2—58.8%, C—75.2%) methods. Bias between volumetric and PISA for RVol (0.24 mL/kg) and RF (13.7%) was biologically acceptable; however, the limits of agreement were wide. Proportional bias between methods was not present for RVol (*P* = 0.79) or RF (*P* = 0.53). Aside from PISA RF, intra‐ and inter‐observer variability assessments showed ICC greater than 0.81.

**Conclusions and Clinical Importance:** Measurement of RVol and RF provide relevant information on MR severity across ACVIM stages of DMVD. Volumetric and PISA methods are not interchangeable.

## Abstract C02: Repeatability of Early versus Late Diastolic Left Atrium/Aorta in Cats With Hypertrophic Cardiomyopathy

### **Luke C. Dutton** ^1^; Jessie Payne^2^, BVetMed, MVetMed, PhD, MRCVS, DACVIM (Cardiology); Ilaria Spalla^3^, DVM, PhD, MRCVS, MVetMed, DACVIM (Cardiology), DECVIM (Cardiology); Virginia Luis Fuentes^4^, MA, VetMB, PhD, CertVR, DVC, MRCVS, DACVIM, DECVIM

#### ^1^Royal Veterinary College, London, England, UK; ^2^Senior Clinician in Cardiology, Langford Vets, UK; ^3^Veterinary Cardiologist, Ospedale Veterinario San Francesco, Italy; ^4^Professor of Veterinary Cardiology, Royal Veterinary College, London, England, UK

**Background:** Short‐axis left atrial diameter indexed to the aorta (LA/Ao), usually measured at end‐systole (LA/Aomax), might be easier to measure at end‐diastole (LA/Aomin) without sacrificing prognostic value.

**Hypothesis:** LA/Aomin would be more repeatable than LA/Aomax and be an independent predictor of survival in cats with hypertrophic cardiomyopathy (HCM).

**Animals:** 247 client‐owned cats (81 healthy cats, 118 HCM stage B cats and 48 HCM stage C cats).

**Methods:** Patient records were retrospectively reviewed, and LA/Aomax and LA/Aomin measured by two investigators. Data presented as mean (95% CI).

**Results:** LA/Aomin reference interval for healthy cats was 0.84 (90% CI 0.80–0.87) to 1.28 (90%CI 1.25–1.32). Compared to LA/Aomax, LA/Aomin showed a higher inter‐operator ICC (0.99 [0.98–1.00] versus 0.91 [0.80–0.97]), lower inter‐operator CV (6.6% versus 10.6%) and less bias (−0.01 [−0.18–0.16] versus −0.13 [−0.43–0.18]). The aortic valve was visible 64% less frequently for LA/Aomax than LA/Aomin (*χ* ^2^ = 14.9, *P* < 0.001). In receiver operating characteristics curve analysis LA/Aomin differentiated stage C from stage B cats with an area under the curve (AUC) of 0.95 (0.92–0.98), similar to LA/Aomax (AUC 0.94 [0.90–0.98]). Kaplan‐Meier analysis showed an LA/Aomin >1.9 (upper quartile) was associated with increased all‐cause mortality (*P* < 0.001), with no difference between other quartiles (*P* > 0.4). The univariable Cox's proportional hazard ratio for LA/Aomin >1.9 was 7.5 (3.76–16.3). Multivariable analysis identified LA/Aomin >1.9, symptomatic status and left ventricular systolic function as independent predictors of survival.

**Conclusions and Clinical Importance:** LA/Aomin demonstrated higher repeatability compared to LA/Aomax and was an independent predictor of survival in cats with HCM.

## Abstract C03: Two‐ and Three‐Dimensional Echocardiographic Assessment of Canine Left Ventricular Outflow Tract Velocity and Area

### **Sarifa Lakhdhir** ^1^; Lynne O'Sullivan^2^, DVM, DVSc, DACVIM (Cardiology); Etienne Côté^2^, DVM, DACVIM (Cardiology, SAIM), FACC, FCAHS; Justin Allen^4^, DVM, DACVIM (Cardiology)

#### ^1^Atlantic Veterinary College, University of Prince Edward Island, Prince Edward Island, Canada; ^2^Professor, Companion Animals, Atlantic Veterinary College, University of Prince Edward Island, Prince Edward Island, Canada; ^4^Cardiologist, VCA West Los Angeles Animal Hospital, Los Angeles, CA, USA

**Background:** Clinically, dogs are screened for subaortic stenosis (SAS) using 2‐D and Doppler echocardiography. Distinguishing between mild SAS and unaffected can be difficult, and a more precise evaluation of the left ventricular outflow tract (LVOT) is desirable.

**Hypothesis/Objectives:** To determine the step‐up in Doppler‐derived peak velocity at different sites of the LVOT and ascending aorta, investigate associated factors, and determine the aortic orifice area using 2‐D and 3‐D echocardiography in dogs.

**Animals:** Sixty‐nine healthy, privately owned dogs.

**Methods:** Prospective study evaluating 2‐D, spectral Doppler, and 3‐D echocardiographic indices of the LVOT and aorta.

**Results:** Obtaining 3‐D images and performing LVOT planimetry was feasible in all dogs (body weight: 4.8–56.6 kg). The mean (range) total step‐up in velocity from LVOT to ascending aorta was 105 cm/s (65–168 cm/s) and 83 cm/s (34–128 cm/s) from subcostal and left apical (Lap) views, respectively. Subcostal‐derived peak LVOT velocity was higher than Lap (*P* < 0.001), and peak velocities derived from both imaging planes were greater after behavioral stimulation (*P* < 0.001). Heart rate, left ventricular volume, and 3‐D echocardiography‐derived aortic valve (AoV) area were significantly associated with LVOT velocity. The 3‐D planimetric AoV area was greater than the 2‐D AoV annulus diameter‐derived area (*P* < 0.001). The continuity equation underestimated 2‐D and 3‐D measures of LVOT area.

**Conclusions and Clinical Importance:** These results quantify the expected step‐up in LVOT velocity and identify associated factors in apparently normal dogs. This information can be used for improving the accuracy of echocardiography in assessing dogs for SAS.

## Abstract C04: Significance of Degenerative Tricuspid Valve Disease in Dogs That Have Undergone Mitral Valve Repair

### **Luke C. Dutton** ^1^; Samantha Lombardo^2^, BSc; Luke Dutton^3^, BVSc, MRes, PhD, MRCVS; Daniel Brockman^4^, BVSc, CVR, CSAO, Dipl ACVS/ECVS, FHEA, MRCVS

#### ^1^Royal Veterinary College, London, England, UK; ^2^Undergraduate Student, Royal Veterinary College, London, England, UK; ^3^Senior Clinical Training Scholar in Veterinary Cardiology, Royal Veterinary College, London, England, UK; ^4^Professor of Small Animal Surgery, Royal Veterinary College, London, England, UK

**Background:** Dogs that undergo mitral valve repair (MVR) for degenerative mitral valve disease (DMVD) frequently have degenerative tricuspid valve disease (DTVD). The significance of DTVD in these dogs has not been studied, therefore it is currently challenging to know if concurrent or subsequent tricuspid valve repair should be considered.

**Hypothesis:** DTVD is common in dogs that have undergone MVR but right‐sided congestive heart failure (R‐CHF) because of DTVD is rare.

**Animals:** 83 dogs that had MVR surgery.

**Methods:** Retrospective study. Case history and echocardiographic reports of dogs that had undergone MVR were reviewed. Dogs with severe pulmonary hypertension (PHT) were excluded from outcome analysis.

**Results:** Study dogs represented 26 different breeds; median age was 9 years (range 5–14 years) with a mean follow‐up time of 1.6 years. Prior to MVR 27% of dogs had moderate‐severe tricuspid regurgitation (TR) and there was no difference in the proportion of dogs with moderate‐severe TR at each follow‐up (1, 6 months, 1, 2 and 3 years, *P* = 0.25). At date of last follow‐up 59/83 (71%) dogs had tricuspid regurgitation documented. One dog developed severe PHT and R‐CHF 6 mo post‐MVR. Severe DTVD (without PHT) occurred in 2/83 (2.4%) dogs, with onset of R‐CHF at 1 and 4 years post‐MVR. The former dog was alive at last follow‐up (3 years) and the latter dog was euthanized 4.5 years post‐MVR due to R‐CHF.

**Conclusions and Clinical Importance:** Progression of DTVD to R‐CHF is rare and therefore simultaneous tricuspid valve repair in dogs undergoing MVR is not justified and would likely present an additional unnecessary risk.

## Abstract C05: Prospective Characterization of Electrocardiographic, Echocardiographic, and Natriuretic Peptide Changes Following Radioiodine Therapy in Hyperthyroid Cats

### **Michelle Oranges** ^1^; Katie Hogan^2^, DVM, DACVIM (Cardiology); Evan Mariotti^3^, DVM, DACVIM (Internal Medicine); Jordan Vitt^4^, DVM, DACVIM (Cardiology)

#### ^1^Angell Animal Medical Center; ^2^Cardiologist, Cardiology, Angell Animal Medical Center; ^3^Internist, Internal Medicine, Angell Animal Medical Center, Boston, MA, USA; ^4^Heart of Veterinary Cardiology

**Background:** The pro‐arrhythmic effects of thyroid hormones are well‐documented in people. Multiple ECG abnormalities have been reported in hyperthyroid cats. Few studies have evaluated echocardiographic and cardiac biomarker parameters pre‐ versus post‐radioiodine treatment for feline hyperthyroidism. It is unknown whether ECG abnormalities improve post‐radioiodine therapy in cats.

**Objectives:** Evaluate 5‐minute ECGs in hyperthyroid cats undergoing I‐131. The hypothesis was that radioiodine treatment would result in improvement of supraventricular and/or ventricular arrhythmias. Additional objectives were to characterize ECG, echocardiographic, and N‐terminal pro‐B‐type natriuretic peptide (NT‐proBNP) changes over time.

**Animals:** Client‐owned cats undergoing I‐131; 32 cats were initially eligible for inclusion. Six cats were excluded, 26 cats were enrolled. One cat did not complete the final evaluation due to euthanasia for non‐cardiac reasons.

**Methods:** Single‐center, prospective study completed June 2021–January 2023. Initial evaluation performed immediately prior to I‐131: physical exam, blood pressure, echocardiogram, NT‐proBNP, thyroid level (T4), and 5‐min 6‐lead ECG. Recheck blood pressure, ECG, T4, and NT‐proBNP were performed 4–6 weeks later. Final evaluation 4–6 months post radioiodine therapy repeated all initial diagnostics.

**Results:** Preliminary analyses revealed a reduction in T4 following I‐131 was associated with a statistically significant reduction in left atrial size (2‐D left atrial to aortic root ratio), left ventricular free wall thickness, left ventricular fractional shortening, NT‐proBNP, weight, and average heart rate. There was a significant reduction in the number of patients with arrhythmias.

**Conclusions:** Prospective studies utilizing 24‐hour Holter or telemetry monitoring are needed to further characterize arrhythmias in hyperthyroid cats undergoing radioiodine therapy.

## Abstract C06: Utility of Radiographic Heart Size Measurements for Predicting Heart Failure in Dogs With Respiratory Distress

### **Evan Ross** ^1^; Lance Visser^1^, DACVIM (Cardiology); Nicholas Sbardellati^2^, DVM;; Brianna Potter^1^, DACVIM (Cardiology); Alex Ohlendorf^1^, DACVR; Brian Scansen^1^; DACVIM (Cardiology)

#### ^1^Colorado State University, Fort Collins, CO, USA; ^2^University of Georgia, Athens, GA, USA

**Background:** Differentiating cardiogenic versus noncardiogenic causes of respiratory signs can be challenging when echocardiography is unavailable. Radiographic vertebral left atrial size (VLAS) and vertebral heart size (VHS) have been shown to predict echocardiographic left heart size, with VLAS specifically estimating left atrial size.

**Hypothesis/Objectives:** Compare the diagnostic accuracy of VLAS and VHS to predict 12 left‐sided congestive heart failure (CHF) in dogs presenting with respiratory signs.

**Animals:** One hundred and fourteen dogs with respiratory signs and radiographic pulmonary abnormalities.

**Methods:** Retrospective cross‐sectional study. Dogs had to have an echocardiogram and thoracic radiographs obtained within 24‐h. Diagnosis of CHF was confirmed based on the presence of respiratory signs, cardiac disease, LA enlargement, and cardiogenic pulmonary edema.

**Results:** Fifty‐seven dogs had CHF—yes, and 57 had CHF—no. Compared to VHS (area under the curve [AUC] 0.85, 95% CI 0.77–0.91), VLAS was a significantly (*P* = 0.03) more accurate predictor of CHF (AUC, 0.92, 95% CI 0.85–0.96). Optimal cutoff for VLAS was >2.3 vertebrae (sensitivity 93.0%, specificity 82.5%). Compared to murmur grade (*P* = 0.40) and VHS (*P* = 0.11), only VLAS (*P* < 0.0001) was independently associated with CHF. Increased VHS (54%) was significantly (*P* = 0.007) more common than increased VLAS (24%) in dogs with CHF—no. Results were similar in a subpopulation of older and smaller dogs.

## Abstract C07: Laboratory and Clinical Findings in Cats Receiving Amiodarone for Treatment of Supraventricular and Ventricular Arrhythmias

### **Graham C. Rossi** ^1^; Sonja Tjostheim^2^, DVM, DACVIM (Cardiology); Kathy Wright^3^, DVM, DACVIM (Cardiology, SAIM); Rebecca Stepien^2^, DVM, DACVIM (Cardiology); Cecilia Marshall^4^, DVM, DACVIM (Cardiology); Heidi Kellihan^2^, DVM, DACVIM (Cardiology)

#### ^1^University of Wisconsin, Madison, WI, USA; ^2^Cardiology Faculty Member, Medical Sciences, School of Veterinary Medicine, University of Wisconsin, Madison, WI, USA; ^3^Cardiologist, MedVet Medical and Cancer Centers for Pets; ^4^Cardiologist, Veterinary Specialty Services

**Background:** Time‐ and dose‐dependent side effects of amiodarone have not been described in cats.

**Hypothesis/Objectives:** Report laboratory findings, adverse events, and clinical outcome in cats receiving amiodarone therapy.

**Animals:** Twenty‐seven client‐owned cats treated with amiodarone.

**Methods:** Medical records were reviewed for signalment, arrhythmia diagnosis, presence of structural heart disease and congestive heart failure at presentation, amiodarone dose, and adverse events. The median ALT, ALP, and T4 values were compared before and after short‐term treatment (0–3 months) and long‐term treatment (>6 months).

**Results:** Amiodarone was most commonly prescribed for ventricular arrhythmias with a median dose of 50 mg/cat/day (range 25–50 mg/cat/day). Most cats had cardiomyopathic disease (26/27; 96%) and many were in congestive heart failure (17/27; 63%) at presentation. Cats received amiodarone for a median of 141 days (range 3–1543 days). The ALT significantly decreased following short‐term treatment (*n =* 19; *P* = 0.01), but there was no significant difference in ALP (*n =* 19; *P* = 0.94) and T4 (*n =* 4; *P* = 0.25). There was no significant difference in ALT (*n =* 9; *P* = 0.65) and ALP (*n =* 10; *P* = 0.87) in cats treated with amiodarone for more than 6 months. Ten cats (37%) had at least one episode of hyporexia or vomiting while receiving amiodarone.

**Conclusions and Clinical Importance:** In cats, amiodarone was used primarily for ventricular arrhythmias and appeared to be generally well tolerated. Transient gastrointestinal signs were reported in cats receiving amiodarone.

## Abstract C08: Hemodynamic Responses to Alternative Right Ventricular Pacing Site Measured in 8 Dogs With AV Block

### **Rachel Van Zile** ^1^; John Bonagura^2^, DVM, MSc, DACVIM (Cardiology, SAIM); Kursten Pierce^3^, DVM, DACVIM (Cardiology); Bruce Keene^4^, DVM, MSc, DACVIM (Cardiology)

#### ^1^North Carolina State University, Raleigh, NC, USA; ^2^Adjunct Professor of Cardiology, Department of Clinical Sciences, North Carolina State University, Raleigh, NC, USA; ^3^Assistant Professor of Cardiology, Department of Clinical Sciences, North Carolina State University, Raleigh, NC, USA; ^4^Professor of Cardiology, Department of Clinical Sciences, North Carolina State University, Raleigh, NC, USA

**Background:** Conventional, effective treatment of high‐grade atrioventricular block (AVB) involves a single endocardial pacing lead positioned near the right ventricular apex (RVA). Other pacing sites might produce less cardiac dyssynchrony and better left ventricular (LV) function.

**Hypothesis/Objectives:** Endocardial pacing at the cranioventral RV outflow tract (RVOT) might differ hemodynamically from RVA pacing.

**Animals:** Client‐owned dogs (*n =* 8) with high‐grade AVB.

**Methods:** Prospective, within‐subjects comparisons (paired t‐tests) of select ECG, LV synchrony and systolic function indices were made between pacing sites. Single plane (apical 2‐chamber) transesophageal echocardiography (TEE) and multiple‐lead ECG recordings were obtained. After transitioning from transcutaneous to temporary endocardial pacing using the permanent lead, data were collected at 100 pulses/minute following a standardized acclimation period during both RVOT and RVA pacing (Table). LV global and endocardial longitudinal strain (GLS, ELS), SD of time to peak strain, single‐plane ejection fraction (EF) and stroke volume, and QRS duration were measured/calculated.

**Results:** Mean EF was higher in the RVOT (*P* = 0.02). ELS for RVOT pacing was −23.1% vs. −20.4% for the RVA (*P* = 0.072) but with no difference in mean GLS. QRS durations were longer in the RVOT site. No intra‐ or postoperative complications occurred.

**Conclusions and Clinical Importance:** This feasibility study was not sufficiently powered to evaluate superiority of lead positioning, but the data to test this hypothesis can be obtained safely, ideally with multiple LV planes and endocardial ECG recordings.

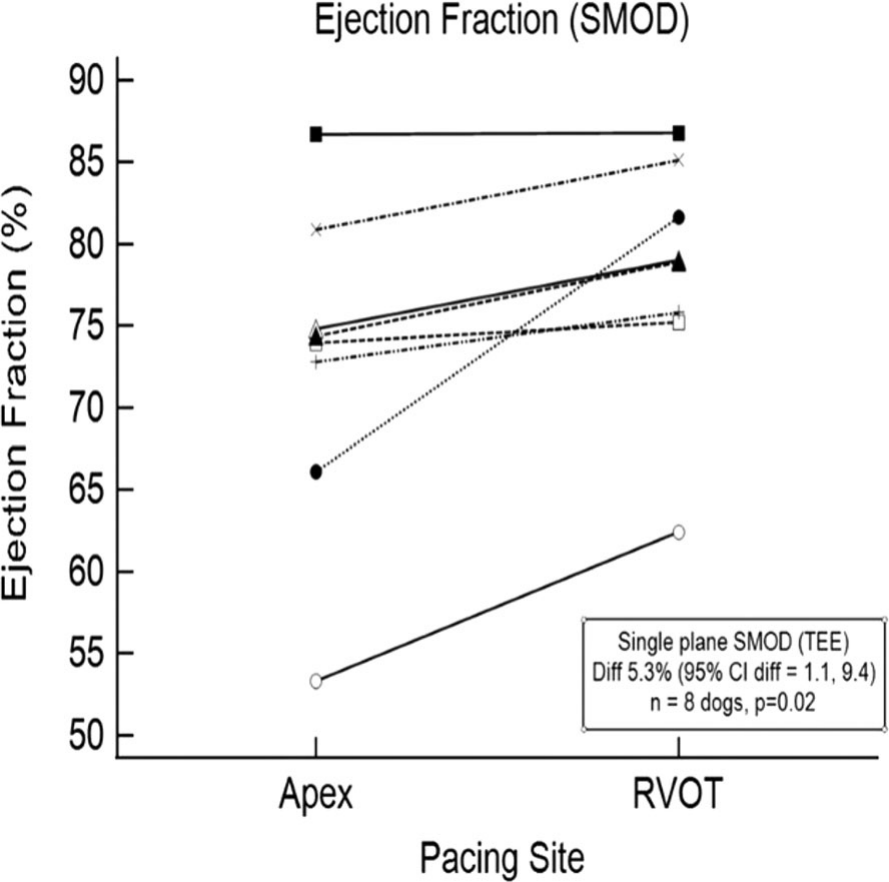

**Table:** 

Variable	RV apex mean	RV apex STD	RVOT mean	RVOT STD	RVA‐RVOT difference	95% Cl difference	*P* value
QRS‐J‐point (ms)	81.0	18.5	94.3	26.2	−13.3	−23.8, −2.8	0.021
QRS‐basline (ms)	77.1	23.1	86.9	17.3	−9.8	−18.7, −0.9	0.036
Longitudinal strain—global (%)	−18.2	3.4	−18.1	6.3	−0.1	−5.3, 5.1	0.966
Longitudinal strain—endocardial (%)	−20.4	3.6	−23.1	4.5	2.7	−0.3, 5.6	0.072
Std dev global longitudinal	2.2	1.2	2.1	1.0	0.1	−1.2, 1.5	0.832
Std dev endocardial longitudinal	2.5	1.4	2.1	0.6	0.4	−0.6, 1.5	0.375
LV ejection fraction SMOD (%)	72.9	9.9	78.1	7.5	−5.2	−9.4, −1.1	0.020
LV stroke volume SMOD (ml)	16.7	14.1	17.6	13.1	−0.9	−2, 0.2	0.095

## Abstract C09: Evaluation of Occult Gastrointestinal Bleeding in Canine Left‐Sided Congestive Heart Failure: A Pilot Study

### **Kristen Verdoorn** ^1^; Mary Tolbert^1,2^, DVM, PhD, DACVIM (SAIM); Sandra Tou^1^, DVM, DACVIM (Cardiology, SAIM); Alex Lynch^1^, BVSc (Hons), DACVECC MRCVS; Elise Andreasen^1^

#### ^1^North Carolina State University, Raleigh, NC, USA; ^2^Texas A&M University, College Station, TX, USA

**Background:** Anemia is an independent negative prognostic indicator in dogs with congestive heart failure, representing a target for improving quality of life and survival. Gastrointestinal bleeding may potentiate anemia in dogs with heart failure from myxomatous mitral valve disease (MMVD). Potential risk factors for gastric ulcerations and erosions (GUE) with MMVD include acquired platelet dysfunction from loss of functional von Willebrand factor (vWF) due to shear stress from mitral regurgitation and chronic intestinal hypoperfusion from decreased cardiac output.

**Hypothesis/Objectives:** Dogs with MMVD stage C have more GUE compared to age‐matched controls.

**Animals:** Six client‐owned MMVD stage C and five healthy dogs enrolled. Exclusion criteria included liver, kidney, or GI disease and antibiotic, steroid, NSAID, or gastric acid suppressant use within 21 days.

**Methods:** Prospective case‐control study evaluating GI mucosa by video capsule endoscopy (VCE), platelet closure time (PC), vWF antigen assay, and complete blood count. Blinded investigator performed VCE scoring.

**Results:** All MMVD dogs had GUE, three of which had diffuse GUE. Five had irregular/thickened mucosa. Four healthy dogs had GUE defined as single, few, or multiple with no diffuse GUE, and only two had irregular mucosa. PCV and vWF antigen assay did not differ between groups. Dogs with MMVD had significantly increased PC with ADP/collagen compared to controls (*P* = 0.0143, Wilcoxon exact test).

**Conclusions and Clinical Importance:** Dogs with MMVD stage C had more severe GUE and irregular gastric mucosa, and platelet dysfunction might be a contributing factor. Larger studies should assess overall prevalence as well as potential therapeutic options.

**Table:** Table 1. Video capsule endoscopy (VCE) scoring results

Patient	GUE presence	GUE severity	Irregular mucosa presence	Irregular mucosa severity
MMVD				
MMVD 1	1	4	1	1
MMVD 2	1	2	1	2
MMVD 3	1	4	1	2
MMVD 4	1	2	0	0
MMVD 5	1	4	1	1
MMVD 6	1	2	1	1
Control				
Control 1	1	1	0	0
Control 2	1	2	1	1
Control 3	0	0	0	0
Control 4	1	3	1	2
Control 5	1	3	0	0

Presence of gastric ulcerations and erosions (GUE) defined as yes (1) or no (0), and GUE severity defined as not present (0), single (1), few (2), multiple (3), or diffuse (4). Irregular mucosa presence defined as yes (1) or no (0), and irregular mucosa severity defined as not present (0), focal (1), or patchy (2).

## Abstract C10: Effects of Pimobendan on Left Atrial Function in Cats With Left Atrial Enlargement and Dysfunction

### **Emily J. Herrold** ^1^; Karsten Schober^2^, DVM, PhD, DECVIM‐CA (Cardiology); Dawn Boothe^3^, DVM, MS, PhD; Jaylyn Rhinehart^4^, DVM, MS, DACVIM (Cardiology); Jiwoong Her^5^, DVM, MS, DACVECC

#### ^1^College of Veterinary Medicine, The Ohio State University, Columbus, OH, USA; ^2^Professor, Cardiology and Interventional Medicine, College of Veterinary Medicine, The Ohio State University, Columbus, OH, USA; ^3^Alumni Professor, College of Veterinary Medicine, Auburn University, Auburn, AL, USA; ^4^Associate Professor, College of Veterinary Medicine, The Ohio State University, Columbus, OH, USA; ^5^Associate Professor, Small Animal Emergency and Critical Care, College of Veterinary Medicine, The Ohio State University, Columbus, OH, USA

**Background:** Arterial thromboembolism is a devastating consequence of hypertrophic cardiomyopathy (HCM) in cats due to left atrial (LA) enlargement and dysfunction. Prevention of ATE relies on anticoagulant medication, rather than improving atrial transport function.

**Hypothesis:** Pimobendan improves LA systolic function in cats with HCM and LA enlargement.

**Animals:** 14 client‐owned cats with stage B2 HCM.

**Methods:** Prospective, double‐blind, placebo‐controlled clinical study. Echocardiographic assessment of left atrial and left auricular systolic function was performed before and one hour after administration of a single oral dose of pimobendan (0.3 mg/kg). Plasma concentrations of pimobendan and o‐desmethyl‐pimobendan (ODMP) were also analyzed.

**Results:** Nine cats received pimobendan and 5 placebo. Preliminary data analysis demonstrated that LA size based on LA area (cm^2^) (3.70 ± 1.27 vs. 3.10 ± 0.81; *P* = 0.08, powe*r =* 0.24) and LA volume (cm3) decreases (5.11 ± 2.54 vs. 4.55 ± 2.36; *P* = 0.19, powe*r =* 0.17), left auricular flow velocity (m/s) increases (0.34 ± 0.15 vs. 0.50 ± 0.29, *P* = 0.14, powe*r =* 0.20), and pulmonary venous atrial reversal flow velocity (m/s) increases (0.31 ± 0.11 vs. 0.36 ± 0.14, *P* = 0.08, powe*r =* 0.32) after pimobendan. Compared to the 6/8 cats with expected plasma concentrations (ng/mL) of pimobendan one hour after administration (7.57–33.98) and ODMP (2.75–11.23), plasma concentrations in 2 cats were notably lower (pimobendan 1.71 and 0.43 ng/mL and ODMP not detectable).

**Conclusions:** Pimobendan may reduce LA size and improve LA transport function in cats with HCM and LA enlargement, but larger a number of cats need to be studied.

## Abstract C11: Echocardiographic Assessment of Aortic Root Size in 220 Healthy Cats

### **Karsten E. Schober**; Katherine Cherry

#### The Ohio State University, Columbus, OH, USA

**Background:** The size of the ascending aorta (aortic root) is of clinical relevance, in particular in cats with systemic hypertension and as a variable of the left atrium‐to‐aortic ratio, used in the echocardiographic diagnosis of left atrial enlargement.

**Hypothesis/Objectives:** Determine reference values and 95% prediction intervals for two‐dimensional echocardiographic variables of aortic root size in cats. We hypothesized that dimensions of the aortic root in healthy cats are mainly dependent on body weight and age.

**Animals:** 220 healthy cats.

**Methods:** Retrospective observational study. Systolic and diastolic dimensions of the aortic valve, the sinus of Valsalva, the sinotubular junction, and the ascending aorta and sinus height were evaluated. Reference values and 95% prediction intervals were determined using raw data and values after allometric transformation. The effect of body weight and age was determined using multivariable regression analysis.

**Results:** Proportionality constants (κ), 95% prediction intervals, and allometric scaling exponents (b) were computed for all variables, and reference values and prediction intervals determined. Body weight and age, but not sex and breed, were independent predictors of aortic root size. Cats >12 years old had significantly (adjusted *P* < 0.05) increased echocardiographic variables of aortic root size compared to cats 1–2 years old, >2–6 years old, and >6–12 years old.

**Conclusions and Clinical Importance:** Reference and prediction intervals of echocardiographic variables characterizing aortic root size in cats are provided. The effect of body weight and age has to be considered for clinical decision‐making when using these variables in clinical practice.

## Abstract C12: Accuracy of Bionote Point‐of‐Care Quantitative N‐Terminal Pro‐brain Natriuretic Peptide Assay in Dyspneic Dogs and Cats

### **Ryan Fries** ^1^; Emily Javery^2^, DVM; Briana Wilson^3^, DVM; Melissa Espinoza^4^; Todd Sumerfield^5^, DVM; Saki Kadotani^6^; Sonya Gordon^7^, DVM, DVSc, DACVIM (Cardiology)

#### ^1^University of Illinois at Urbana Champaign, Champaign, IL, USA; ^2^Cardiology Resident, Veterinary Clinical Medicine, University of Illinois, IL, USA; ^3^Cardiology Specialty Intern, MSPCA Angell Animal Medical Center; ^4^Veterinary Student, Texas A&M University, College Station, TX, USA; ^5^Cardiology Research Intern, Veterinary Clinical Medicine, University of Illinois, IL, USA; ^6^Clinical Assistant Professor, Veterinary Clinical Medicine, University of Illinois, IL, USA; ^7^Professor Cardiology, Eugene Ch’en Chair in Cardiology, Small Animal Clinical Sciences, Texas A&M University, College Station, TX, USA

**Background:** Circulating N‐terminal pro‐B‐type natriuretic peptide (NT‐proBNP) concentrations accurately discriminate congestive heart failure (CHF) from non‐cardiac respiratory dyspnea (NC‐RD), yet quantitative results may take >48 h. Our objective was to determine if the quantitative Bionote Vcheck point‐of‐care (POC) NT‐proBNP serum assay accurately discriminates CHF versus NC‐RD in dogs and cats.

**Hypothesis:** Quantitative POC NT‐proBNP will accurately discriminate CHF versus NC‐RD in dogs and cats.

**Animals:** Sixty‐nine dogs and 40 cats from 2 university hospitals.

**Methods:** In this prospective cross‐sectional study, serum NT‐proBNP concentrations were measured in client‐owned dogs and cats presenting for dyspnea. Categorization of CHF was based on echocardiographic cardiac disease and edema or effusion that resolved with diuretic treatment. Normality was assessed using a Shapiro‐Wilk test, and groups were compared using a Mann‐Whitney test and receiver operating curves. A *P* < 0.05 was considered significant.

**Results:** CHF animals had higher (*P* < 0.001) median NT‐proBNP concentrations (cat, 1293 pmol/L; dog, 3107 pmol/L) compared with NC‐RD (cat, 49 pmol/L; dog, 726 pmol/L). ROC analysis indicated excellent discriminatory ability in dogs (AUC = 0.97, *P* < 0.0001) and cats (AUC = 0.99, *P* < 0.0001). An optimal cutoff in dogs of >1900 pmol/L achieved a sensitivity of 85% and specificity of 95%. An optimal cutoff in cats of >272 pmol/L achieved a sensitivity of 100% and specificity of 94%.

**conclusion and clinical importance:** The Bionote Vcheck NT‐proBNP assay is an accurate quantitative POC test that may be helpful in the immediate assessment of dyspneic dogs and cats.

## Abstract C13: Mitral Valve Repair Outcomes in Different Dog Breeds

### **Kentaro Kurogochi**; Masami Uechi, DVM, PhD, DAiCVIM (Cardiology)

#### JASMINE Veterinary Cardiovascular Medical Center, Japan

**Background:** Mitral valve repair (MVR) could be a curative treatment for myxomatous mitral valve disease (MMVD) in dogs. Previous reports in medical treatment have suggested that Cavalier King Charles Spaniels (CKCS) might be more susceptible to developing MMVD than other breeds, but the breed‐specific characteristics and the long‐term outcomes of MVR are unclear.

**Hypothesis/Objectives:** We investigated the differences in cardiac reverse‐remodeling and survival for MVR in different breeds.

**Animals:** We retrospectively analyzed the data of dogs with MMVD that underwent MVR from February 2017 to February 2019 at our center.

**Methods:** Based on breeds, dogs were divided into three groups: Chihuahuas, CKCS, and others. Thoracic radiographs and echocardiography were evaluated in the preoperative period and 6, 12, and 36 months post‐surgery.

**Results:** Our study included 444 dogs (Chihuahuas = 206, CKCS = 40, and others = 198). CKCS group had significantly higher vertebral heart score, left atrial aortic root ratio, and diastolic left ventricular normalized dimension both pre‐ and post‐surgery. The three‐year survival rate for all‐cause mortality derived by the Kaplan‐Meier curve was not different among the three groups: 75%, 66%, and 67%, respectively (*P* = 0.12; log‐rank test).

**Conclusions and Clinical Importance:** Postoperative reverse remodeling might differ, especially in CKCS. Thus, early surgical intervention might be better for improving reverse remodeling in CKCS. However, MVR improves survival in all breeds despite differences in heart size.

## Abstract C14: Clinical, Electrocardiographic, and Diagnostic Imaging Features and Outcomes in 23 Cats With Ventricular Preexcitation

### **Emily A. Gavic** ^1^; Christopher Stauthammer^2^; Allison Masters^2^; Aaron Rendahl^3^; Keaton Morgan^2^; Marisa Ciccozzi^2^; Kathleen Beekmann^4^; Robbie George^5^; Emily Herrold^6^; Lauren Markovic^7^; Karsten Schober^6^; Sonja Tjostheim^8^; Kathy Wright^4^

#### ^1^University of Minnesota, MN, USA; ^2^Veterinary Medical Center, University of Minnesota, MN, USA; ^3^College of Veterinary Medicine, University of Minnesota, MN, USA; ^4^MedVet Cincinnati, Cincinnati, OH, USA; ^5^Animal Emergency and Referral Center of Minnesota, MN, USA; ^6^The Ohio State University, OH, USA; ^7^University of Georgia, GA, USA; ^8^University of Wisconsin, WI, USA

**Background:** Ventricular preexcitation (VPE) occurs when atrial electrical impulses prematurely excite the ventricles through aberrant muscle bundles known as accessory pathways (AP). Orthodromic atrioventricular reciprocating tachycardia (OAVRT) is a re‐entrant narrow complex tachyarrhythmia maintained by an AP.

**Hypothesis/Objectives:** To describe signalments, clinical signs, electrocardiographic and diagnostic imaging features, treatments, prognostic variables, and outcomes in cats with VPE. We hypothesized that cats with VPE are at risk for developing OAVRT and associated clinical signs. Negative prognostic indicators were hypothesized to include presence of clinical signs and structural heart disease.

**Animals:** Twenty‐three cats diagnosed with VPE between January 2010 and August 2022.

**Methods:** Retrospective, multicenter study. VPE diagnosis was based on ECG evidence of reduced PR interval and delta wave QRS morphology. Median survival time (MST) was estimated by Kaplan‐Meier curve.

**Results:** Fourteen (60.8%) cats with VPE had OAVRT documented on ECG and 4/23 (17.4%) had suspected OAVRT based on associated clinical signs. Common presenting signs included collapse (15/23; 65.2%) and respiratory distress (14/23; 60.8%). Five cats (21.7%) were asymptomatic. ECG during VPE revealed a reduced PR interval (median 40 ms; range 20–45 ms) and prolonged QRS duration (median 60 ms; range 45–90 ms). Heart rate during OAVRT ranged from 310–420 bpm (median 375 bpm). Treatment included atenolol (10/18), sotalol (5/18), diltiazem (2/18), and amiodarone (1/18). MST from date of diagnosis was 1872 days.

**Conclusions and Clinical Importance:** The majority of cats in this study with VPE had clinical OAVRT. The prognosis for cats with VPE is considered fair with MST >5 years.

## Abstract C15: Hemodynamic, Echocardiographic, Electrocardiographic, and Behavioral Effects of Oral Trazodone in Healthy Dogs

### **Lauren E. Quigley** ^1^; Melissa Bain^2^, DVM, DACVB, MS, DACAW; Elise Lenske^3^, BVSc; Jeffrey Fine^4^, MPH; Yunyi Ren^4^, MS; Allison Gagnon^5^, DVM, MS, DACVIM (Cardiology)

#### ^1^School of Veterinary Medicine, University of California, Davis, CA, USA; ^2^Professor of Clinical Animal Behavior, Department of Medicine and Epidemiology, School of Veterinary Medicine, University of California, Davis, CA, USA; ^3^Resident of Cardiology, Department of Medicine and Epidemiology, School of Veterinary Medicine, University of California, Davis, CA, USA; ^4^Department of Public Health Sciences, School of Medicine, University of California, Davis, CA, USA; ^5^Assistant Professor of Cardiology, Department of Medicine and Epidemiology, School of Veterinary Medicine, University of California, Davis, CA, USA

Oral trazodone is used as an anti‐anxiety medication in dogs prior to veterinary visits; however, the effects on cardiac diagnostics are not well studied. The purpose of this study was to investigate the effects of oral trazodone on blood pressure (BP), echocardiography, electrocardiography, and behavior in healthy dogs.

15 healthy, client‐owned, adult dogs aged 1–8 years were enrolled in this prospective, double‐blinded, placebo‐controlled, crossover study. Dogs were randomized to receive placebo or trazodone (9.2–11.2 mg/kg) orally 90–120 min before arrival. Physical examination, Doppler BP, electrocardiogram, echocardiogram, and 24‐h Holter placement were performed. After a minimum 7‐day wash‐out period, diagnostics were repeated with the alternate treatment. Stress scores were obtained using owner questionnaires and a blinded review of video obtained during visits. For normally distributed data, a two‐sample t‐test for comparison of means between drugs was performed.

Blood pressure was significantly decreased following trazodone (mean ± SD, 128.7 ± 22.3 vs. 143.4 ± 26.61, *P* = 0.02). Measurements of left atrial size, left ventricular size and function, and corrected QT interval were not significantly different. Average 24‐h heart rate was not significantly different. Median (interquartile range) of ventricular ectopy was 0 (0–2) for both groups. Owner‐reported stress and average video‐scored stress were significantly decreased following trazodone (*P* = 0.005 and *P* = 0.003). Adverse effects reported with trazodone were sleepiness (12/15), decreased appetite (4/15), and ataxia (1/15).

In conclusion, a single, oral, pre‐visit dose of trazodone is well‐tolerated and reduces signs of stress in dogs. Trazodone causes mild reduction in BP and clinically insignificant echocardiographic and electrocardiographic changes.

## Abstract C16: Comparison of Two‐Dimensional Transesophageal Echocardiographic Quantification of Mitral Valve Annular Area with Three‐Dimensional Planimetric Imaging

### **Todd Sumerfield** ^1^; Lindsey Humphries^2^, DVM, MS; Emily Javery^3^, DVM; Sumana Prabhakar^3^, DVM; Saki Kadotani^4^, DVM, DACVIM (Cardiology); Ryan Fries^5^, DVM, DACVIM (Cardiology)

#### ^1^University of Illinois, Urbana, IL, USA; ^2^Cardiology Resident, University of Illinois, Urbana, IL, USA; ^3^Cardiology Resident, Veterinary Clinical Medicine, University of Illinois, Urbana, IL, USA; ^4^Clinical Assistant Professor, Veterinary Clinical Medicine, University of Illinois, Urbana, IL, USA; ^5^Assistant Professor, Veterinary Clinical Medicine, University of Illinois, Urbana, IL, USA

**Background:** With new interventional procedures aimed at treating degenerative valve disease, the need to stratify patients into surgical categories is necessary. Some methods for determining regurgitant fraction require accurate quantification of mitral annular area (MAA). This retrospective study evaluated MAA with standard two‐dimensional (2D) transesophageal (TEE) measurements compared with three‐dimensional (3D) planimetry.

**Hypothesis:** 2D orthogonal views will most closely approximate MAA compared with the reference standard 3D planimetry.

**Animals:** Thirty‐two dogs of variable breed and size

**Methods:** All TEE studies were performed as part of catheter‐based interventions including congenital disease and transcatheter edge‐to‐edge mitral valve repair (TEER). Studies were reviewed using 3D planimetry and traditional apical 2D views: 2‐chamber (2CH), 4‐chamber (4CH), commissural (CC), and anterior‐posterior (AP). Data were assessed for normality using a Shapiro‐Wilk test. Continuous variables comparing 2D with 3D planimetry were assessed using a Friedmean test with Dunn's multiple comparisons, Spearman rank correlation, and Bland‐Altman plots. A *P* < 0.05 was considered significant.

**Results:** Two‐dimensional apical 2CH and 4CH MAA and orthogonal 4Ch/2CH and CC/AP ellipse MAA were not significantly different than planimetry. There was significant correlation between planimetry and 4CH (*r =* 0.95, *P* < 0.001), 2CH (*r =* 0.92, *P* < 0.01), 4CH/2CH (*r =* 0.97, *P* < 0.0001), and CC/AP (*r =* 0.99, *P* < 0.0001). Among these variables CC/AP had the smallest bias (0.09–0.37) and closest limits of agreement (−0.63 to 0.81) with planimetry.

**conclusion and clinical importance:** Two‐dimensional CC/AP derived MAA is an acceptable alternative to 3D planimetry in dogs.

## Abstract C17: Effect of Amiodarone, Diltiazem, or Both on Heart Rate Control in Dogs with Atrial Fibrillation

### **Sonja Tjostheim** ^1^; Heidi Kellihan^2^, DVM, DACVIM (Cardiology); Rebecca Stepien^2^, DVM, MS, DACVIM (Cardiology)

#### ^1^School of Veterinary Medicine, University of Wisconsin‐Madison, Madison, WI, USA; ^2^Clinical Professor, Cardiology, Medical Sciences, School of Veterinary Medicine, University of Wisconsin‐Madison, Madison, WI, USA

**Background:** Dogs with atrial fibrillation (AF) live longer with strict heart rate control (SHRC, 24‐hour mean heart rate ≤125 beats/minute). Although amiodarone (AM) and diltiazem (DTZ) are used to lower ventricular response rate in dogs with AF, information about their relative efficacy for achieving SHRC is lacking.

**Hypothesis/Objectives:** Determine if therapy with AM, DTZ or a combination will achieve SHRC in dogs with AF.

**Animals:** Twelve client‐owned dogs with newly‐diagnosed AF were enrolled and returned for follow‐up.

**Methods:** Prospective, randomized, non‐blinded clinical trial. Eligible dogs had AF with mean daily heart rate (MDHR) >125 beats/minute (baseline, 24‐h Holter) and were randomly assigned to receive AM or DTZ at standard doses. Repeat 24‐h Holter assessments were performed at 3, 6 and 9–21 weeks after starting treatment. If MDHR >125 beats/minute at any assessment, the second drug was added.

**Results:** Six dogs each received AM or DTZ first. MDHR decreased significantly after AM (*P* = 0.03) or DTZ (*P* = 0.05) initiation; 1 dog in each group achieved SHRC at 3 weeks. At last follow‐up, 11/12 dogs were receiving combined AM/DTZ for median [range] 50 [20–53] days with no difference based on which drug was initiated first (*P* = 0.87). Most dogs (7/11, 64%) had not achieved SHRC (139 [94–160]) beats/minute at standard doses (AM, 7.5 [6.2–9.3] mg/kg/day; DTZ, 7.3 [6.3–11.9] mg/kg/day).

**Conclusions and Clinical Importance:** Monotherapy with AM or DTZ or combination therapy of AM/DTZ does not achieve SHRC in most dogs with AF.

## Abstract C18: Transpulmonary Stent Implantation in 60 Dogs With Congenital Pulmonary Valve Stenosis

### **Brianna M. Potter** ^1^; Brian Scansen^2^, DVM, MS, DACVIM (Cardiology)

#### ^1^Veterinary Teaching Hospital, Colorado State University, Ft. Collins, CO, USA; ^2^Cardiology and Cardiac Surgery, Veterinary Teaching Hospital, Colorado State University, Ft. Collins, CO, USA

**Background:** Stent implantation is an emerging therapy for congenital pulmonary valve stenosis (PS) in dogs.

**Hypothesis/Objectives:** Describe two operators experience with balloon‐expandable (BE) transpulmonary stents for PS at a single institution.

**Animals:** 60 dogs with PS, including French bulldogs (*n =* 28), English bulldogs (7), pit bulls (6), other breeds (19).

**Methods:** Retrospective analysis of medical records.

**Results:** All dogs survived to discharge. The mean (±SD) age at time of intervention was 22.4 ± 19.9 months (max, min *=* 3, 104) at a weight of 12.8 ± 6.6 kg (4.2, 30.9). Anomalous coronary anatomy was present in 7 dogs. Nineteen dogs were in congestive heart failure at time of intervention. For 41 dogs, transpulmonary stent implantation was their first PS intervention, while 19 dogs had previously undergone balloon pulmonary valvuloplasty that was deemed ineffective. Seven different BE stent designs were implanted, with a 10 mm diameter bare metal stent being the most common in 40 dogs; 3 cases received covered stents. Vascular access varied from 7 Fr to 14 Fr. Pre‐mounted stents were selected in 48 cases; 12 cases used hand‐crimped stents. Post‐dilation was performed in 17 dogs. A balloon‐in‐balloon delivery catheter was used in 10. Echocardiographically‐derived peak systolic pressure gradients were 133 ± 44 mm Hg (60, 248) prior to stenting and 53 ± 28 mm Hg (10, 180) the next day for an average reduction of 58% ± 22% (0%, 90%).

**Conclusions:** Stent implantation is feasible for dogs with PS, effecting a clinically relevant reduction in pressure gradient at time of discharge.

## Abstract C19: Subclinical Cardiovascular Abnormalities in Apparently Healthy, Aged, Dogs Screened for a Large Randomized Clinical Trial

### **Rachel L. Melvin** ^1^; Anya Gambino^2^, DVM, DACVECC; Mary Iannaccone^3^, DVM; Mandy Coleman^4^, DVM, DACVIM (Cardiology)

#### ^1^School of Veterinary Medicine & Biomedical Sciences, Texas A&M University, College Station, TX, USA; ^2^Dog Aging Project, Laboratory Medicine & Pathology, School of Medicine, University of Washington, Seattle, WA, USA; ^3^DAP Consortium, Dog Aging Project, Laboratory Medicine & Pathology, School of Medicine, University of Washington, Seattle, WA, USA; ^4^Department of Small Animal Medicine and Surgery, College of Veterinary Medicine, University of Georgia, Athens, GA, USA

**Background:** There is little published research on prevalence of subclinical disease and cardiovascular abnormalities in apparently healthy mature adult dogs.

**Hypothesis/Objectives:** To describe prevalence of subclinical disease and cardiovascular examination abnormalities, and reasons for participant exclusion, in dogs screened for a clinical trial.

**Animals:** Reportedly healthy, client‐owned, mature adult (>7‐year‐old) dogs screened for a large, prospective, randomized multicenter clinical trial.

**Methods:** Prior to in‐clinic screening, electronic medical records of owner‐nominated dogs were reviewed for evidence of systemic illness or treatment with an exclusionary drug. For each, signalment, body weight, and reason(s) for exclusion were recorded. For dogs subsequently screened in‐person, indirect systolic arterial blood pressure (SBP), heart murmur presence, findings of echocardiography and electrocardiography, and any reason(s) for exclusion were recorded.

**Results:** 90 (29%) of 310 dogs were excluded prior to in‐clinic screening, most commonly for exclusionary drugs or diagnoses (40.0% and 34.4% of excluded dogs, respectively). 50 (41%) of 121 dogs screened in‐clinic were excluded, most commonly for a clinically significant cardiovascular or biospecimen abnormality (50% and 20% of excluded dogs, respectively). Degenerative mitral valve disease (DMVD) was diagnosed in 38 (33%) of 116 dogs undergoing echocardiography; of these, 18 (47%) had a heart murmur. Premature ventricular ectopy was documented in 20 (17%) of 117 dogs undergoing electrocardiography.

**Conclusions and Clinical Importance:** Cardiovascular abnormalities were common in this apparently healthy sample of mature adult dogs. Absence of a heart murmur in many dogs with DMVD suggests poor sensitivity of auscultation for this condition.

## Abstract C20: Clinical Findings, Outcomes and Risk Factors in 100 Dogs Hospitalized for Acute Cardiogenic Pulmonary Edema

### **Chung‐Yao Yin** ^1^, Ta‐Li Lu^1^, DAiCVIM (Cardiology) John Rush^2^, MS, DVM, DACVIM (Cardiology), DACVECC

#### ^1^Chuan Animal Hospital; ^2^Department of Clinical Sciences, Cummings School of Veterinary Medicine, Tufts University, Medford, MA, USA

**Background:** Acute cardiogenic pulmonary edema (CPE) is a serious development for dogs with myxomatous mitral valve disease (MMVD), and CPE commonly requires hospitalization.

**Hypothesis/Objectives:** To characterize clinical findings and evaluate risk factors in dogs with MMVD hospitalized with acute CPE.

**Animals:** 100 dogs with MMVD hospitalized with acute CPE.

**Methods:** Prospective observational study measuring clinical parameters and Tufts Dyspnea Score (TDS) within the first 24 h of hospitalization to assess outcomes. Descriptive statistics and regression analysis were used.

**Results:** Upon hospital presentation, median respiratory rate was 64 bpm and median TDS was 5 (TDS range 0 to 10). Median length of stay in the ICU was 20 h. Nighty‐five of 100 dogs survived to hospital discharge. For the 91 dogs with follow‐up information, 39 experienced death or re‐hospitalization for CPE within 2 months after hospital discharge. No clinical parameter at hospital presentation predicted survival to hospital discharge. The lowest recorded TDS within the first 12 h (TDS ≦4) had a sensitivity of 97.9% and specificity of 80% for predicting survival to hospital discharge. Dogs with a louder murmur at presentation or a persistently high respiratory rate within the first 12 h were more likely to have an adverse two‐month outcomes.

**Conclusions and Clinical Importance:** We observed a high survival rate in dogs with MMVD hospitalized with acute CPE. No clinical parameter at hospital presentation predicted survival to hospital discharge, however, parameters recorded during the initial 24 h of hospitalization did predict hospital discharge and adverse two‐month outcomes.

## Abstract C21: Development and Validation of a Machine Learning Algorithm to Detect Canine Heart Murmurs

### **Lara Barron** ^1^; Andrew McDonald^2^; Jose Novo Matos^3^; Joel Silva^3^; Catheryn Partington^3^; Eve Lo^4^; Virginia Luis Fuentes^4^; Penny Watson^3^; Anurag Agarwal^2^

#### ^1^Davies Veterinary Specialists, Shillington, UK; ^2^Department of Engineering, University of Cambridge, Cambridge, UK; ^3^Department of Veterinary Medicine, University of Cambridge, Cambridge, UK; ^4^Royal Veterinary College, London, UK

**Background:** A heart murmur is a key clinical feature of canine degenerative valve disease (DVD). Recent machine learning algorithms, combined with electronic stethoscopes, have shown promise at detecting heart murmurs in people, but further evidence is needed of their application to canine heart sounds.

**Hypothesis/Objectives:** To train and evaluate a machine learning algorithm to detect and grade heart murmurs in dogs, using recordings from an electronic stethoscope.

**Animals:** 470 client‐owned dogs (266 DVD, 132 other cardiac diseases, 72 normal) undergoing routine echocardiography at four referral centers.

**Methods:** Diagnostic method development and assessment. For each dog, a board‐certified cardiologist or cardiology resident used an electronic stethoscope to grade murmurs and make 15‐second recordings at three auscultation sites (left apex, left base, right side). A recurrent neural network, originally developed for human murmur detection, was then retrained to predict the cardiologist's murmur grade from the sound recording.

**Results:** On an unseen half of the data set, the neural network detected a murmur of any grade with an area under the receiver operating characteristic of 0.907 (95% CI 0.887–0.927), with an operating sensitivity of 89.3% (95% CI 85.1%–93.5%) and specificity of 74.9% (95% CI 66.7%–83.0%). The individual Levine murmur grade was predicted with a mean absolute error of 0.648 (95% CI 0.602–0.694).

**Conclusions and Clinical Importance:** The algorithm demonstrates good agreement with cardiologists, potentially enabling a non‐expert user to perform accurate murmur screening and grading.

## Abstract C22: Use of a Newly Developed Vascular Occluder System for Closing Patent Ductus Arteriosus in Dog

### **Min Su Kim** ^1^; Adin Darcy^2^, DVM, DACVIM (Cardiology); Meg Sleeper^3^, VMD, DACVIM (cardiology); Michael Aherne^4^, MVB, GradDipVetStud, MS, MANZCVS (Small Animal Surgery), DACVIM (Cardiology); Amara H. Estrada^5^, DVM, DACVIM (Cardiology); Benjamin Diaz Edurado J^6^, DVM, MS, DACVIM (Cardiology); Kyoung‐a Youp^7^, DVM, MS; Woon‐Bum Baek^8^, DVM, MS; Taeil Kim^9^, DVM, PhD; Jaemin Seol^10^, DVM, MS; Junwoo Bae^11^, DVM, MS; In Sung Jang^12^, DVM, MS; Daeyun Seo^13^, DVM; Seong Soo Lim^13^, DVM; Beomkwan Namgoong^13^, DVM; Ahreum Choe^13^, DVM; heesung Umh^13^, DVM; Hyea Jeong Hong^13^, DVM

#### ^1^College of Veterinary Medicine, Seoul National University, Seoul, South Korea; ^2^Clinical Professor, Large Animal Clinical Sciences, University of Florida, Gainesville, FL, USA; ^3^Clinical Professor Cardiology, Department of Small Animal Clinical Sciences, College of Veterinary Medicine, University of Florida, Gainesville, FL, USA; ^4^Clinical Assistant Professor, Small Animal Clinical Sciences, College of Veterinary Medicine, University of Florida, Gainesville, FL, USA; ^5^Professor of Cardiology, Small Animal Clinical Sciences, College of Veterinary Medicine, University of Florida, Gainesville, FL, USA; ^6^Clinical Assistant Professor, Cardiology, Small Animal Clinical Sciences, College of Veterinary Medicine, University of Florida, Gainesville, FL, USA; ^7^Clinical Veterinarian/Interventional Center, Korea Animal Medical Center, Chungcheongbuk‐do, South Korea; ^8^Clinical Veterinarian, 24h Daejeon Animal Medical Center Soop; ^9^Clinical Veterinarian, Bone Animal Medical Center; ^10^Clinical Veterinarian, Lucid Animal Medical Center, Gangbuk‐gu, Seoul, South Korea; ^11^Clinical Veterinarian, Poom Animal Medical Center; Clinical Veterinarian, Ansim Animal Medical Center; ^13^Clinical Veterinarian/Emergency Medicine, Department of Clinical Science, College of Veterinary Medicine, Seoul National University, Seoul, South Korea

**Background:** The Amplatz canine duct occluder (ACDO) is commercially available for PDA occlusion, however, additional small animal‐specific devices are still needed to address current device limitations regarding patient size or ductal morphology. We have developed a new device designed to occlude the PDA of dogs. The system is self‐contained with a side‐port for angiography, optimal sheath curvature, and is designed to reduce catheter and wire exchanges. The sheath is 5 Fr and can accommodate 6–10 mm occluders.

**Hypothesis/Objectives:** The new system and occluder can be introduced into the right femoral artery and successfully deployed into the ductus of dogs >1.5 kg with Type IIa or IIb left‐to‐right shunting PDA under fluoroscopic guidance.

**Animals:** Client owned ten dogs with PDA from May 2021 to January 2023.

**Methods:** We retrospectively evaluated the system in 10 dogs with PDA. Occluder size and procedural time were recorded. Echocardiography was used to evaluate the completeness of the PDA occlusion at 1 day and 1‐, 3‐ and 6‐months post‐procedure.

**Results:** Mean body weight was 3.14 kg (1.5–4.6 kg). Occluder sizes used were 6mm (*n =* 4), 8 mm (*n =* 2), and 10 mm (*n =* 4). Excluding anesthesia, mean procedure time was 25 min (20–30 min). Occlusion of the PDA was complete at 1 day in 3 dogs and by 1 month in 5 dogs. Residual flow was found in 2 dogs at 3 months but resolved by 6 months.

**Conclusions and Clinical Importance:** This study shows that the new PDA occluder system is effective in small dogs requiring 6–10 mm occluders.

**Title:** Use of a newly developed vascular occluder system for closing patent ductus arteriosus in dog.

**Figure:**
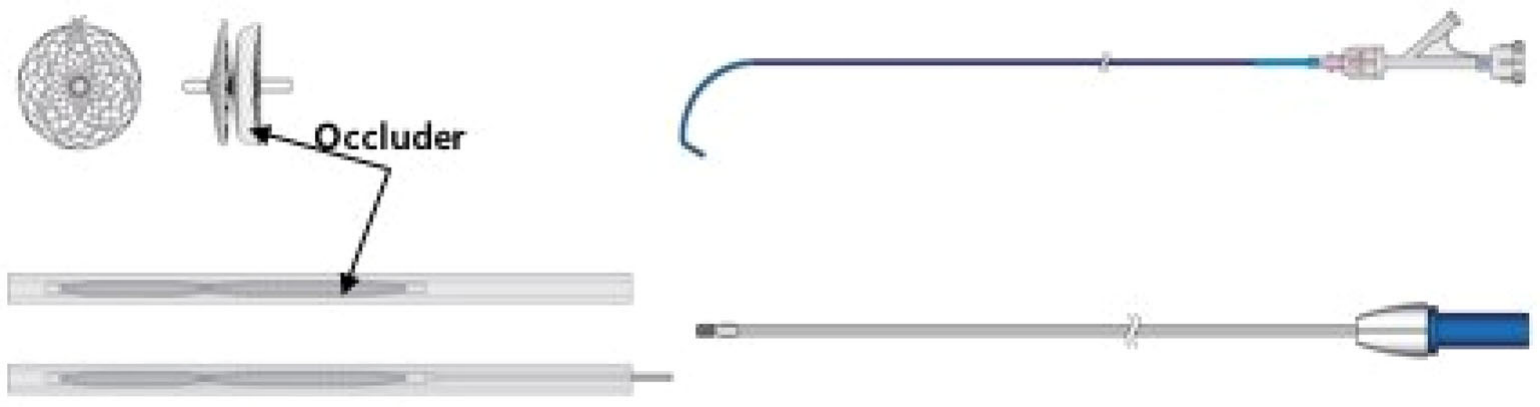
**Figure 1.** A newly developed occluder system.

## Abstract C23: Reperfusion Ventricular Fibrillation in Mitral Valve Repair: A Comparison of Blood Cardioplegia and Crystalloid Cardioplegia

### **Kentaro Kurogochi**; Masami Uechi

#### JASMINE Veterinary Cardiovascular Medical Center, Yokohama, Japan

**Background:** Mitral valve repair (MVR) could be a curative treatment for myxomatous mitral valve disease in dogs, and cardioplegic solutions play an essential role in its procedure. However, procedure methodologies are not well established, and complications have not been clarified yet.

**Hypothesis/Objectives:** We hypothesized that the incidence of ventricular fibrillation (VF), the main complication of reperfusion injury after cross‐clamping, might be lower with blood cardioplegia (BCP) than with crystalloid cardioplegia (CCP).

**Animals:** A total of 251 client‐owned dogs who underwent MVR by a single surgeon from November 2015 to November 2017 were included.

**Methods:** We retrospectively analyzed the dogs’ surgical records, including VF incidence, use of transfusion, and cross‐clamping time, as well as echocardiographic measurements obtained before and 1‐month after surgery. Dogs were divided into two groups according to the type of cardioplegia, CCP and BCP.

**Results:** Logistic regression analysis showed that group CCP (odds ratio [OR] = 2.20, confidence interval [CI] = 1.06–4.57; *P* = 0.03) and cross‐clamping time (O*R =* 1.04, CI = 1.01–1.06, *P* < 0.01) were associated with AF. In addition, group CCP (O*R =* 2.52, CI = 1.19–5.32, *P* = 0.02) and low body weight (O*R =* 0.58, CI = 0.49–0.69, *P* < 0.01) were associated with transfusion use. There was no difference between the groups in the pre‐ and postoperative echocardiographic measurements.

**Conclusions and Clinical Importance:** The BCP group had a lower incidence of VF and less use of blood transfusion than the CCP group. This finding indicates that BCP is a superior cardioplegic technique for MVR in dogs.

## Abstract C24: Transcatheter Occlusion of Patent Ductus Arteriosus: Multicenter Collaborative Study Across Pediatric and Veterinary Cardiology Centers

### **Lauren E. Markovic** ^1^; Gurumurthy Hiremath^2^, MD, FACC, FSCAI, FPICS; Brian Scansen^3^, DVM, MS, DACVIM (Cardiology); Heidi Kellihan^4^, DVM, DACVIM (Cardiology); Caitlin Calkins^5^, NP; Amanda Coleman^6^, DVM, DACVIM (Cardiology); Sonja Tjostheim^7^, DVM, DACVIM (Cardiology); Brianne Tainter^8^ PA‐C; Katie Hodges^9^, RPhT; Erin Cahill^10^; Mary Carter^11^, DVM; Dennis Kim^12^, MD, PhD, FSCAI

#### ^1^College of Veterinary Medicine, University of Georgia, Athens, GA, USA; ^2^Associate Professor, Masonic Children's Hospital, University of Minnesota, Minneapolis, MN, USA; ^3^Professor, Colorado State University, Ft. Collins, CO, USA; ^4^Clinical Associate Professor, University of Wisconsin, Madison, WI, USA; ^5^Pediatric Nurse Practitioner, Cardiac Cath Lab, Children's Healthcare of Atlanta, Atlanta, GA, USA; ^6^Associate Professor, University of Georgia, Athens, GA, USA; ^7^Clinical Assistant Professor, University of Wisconsin, Madison, WI, USA; ^8^Physician Assistant, Masonic Children's Hospital, University of Minnesota, Minneapolis, MN, USA; ^9^Research Technician, University of Georgia, Athens, GA, USA; ^10^DVM Student, Colorado State University, Ft. Collins, CO, USA; ^11^University of Wisconsin, Madison, WI, USA; ^12^Professor, Children's Healthcare of Atlanta, Emory University School of Medicine, Atlanta, GA, USA

**Background:** Transcatheter therapeutics have revolutionized management of patent ductus arteriosus (PDA) in children and animals. Further understanding of interventions for PDA across species may help improve outcomes for both.

**Hypothesis/Objectives:** To describe characteristics and outcomes of PDA in patients undergoing transcatheter occlusion in pediatric and veterinary populations.

**Animals:** 202 human patients from two pediatric cardiology centers and 106 dogs from three veterinary cardiology centers.

**Methods:** Multicenter, retrospective review analyzing data of PDA patients between July 1, 2019–June 30, 2021. Demographics, procedural characteristics, and outcomes were assessed.

**Results:** Active or historical congestive heart failure was present in 45/202 (23%) humans and 19/106 (18%) dogs prior to catheterization (*P* = 0.40). A transvenous approach from the right femoral vein was most common in 173/202 (86%) humans, compared to a transarterial approach using the right femoral artery in 101/106 (95%) dogs. Anticoagulant use during human intervention 165/165 (100%) significantly differed from canine intervention 2/103 (2%) (*P* < 0.0001). Vascular closure techniques differed with manual compression in all humans versus vessel ligation in 76 (73%) and vessel repair in 25 (24%) dogs following vascular cut‐down; an additional 2 (2%) dogs underwent suture‐based percutaneous closure and 1 dog (1%) manual compression (*P* < 0.0001). Successful intervention (appropriate device deployment) was seen in 98% of humans compared to 93% of dogs (*P* = 0.051).

**Conclusions:** Humans and dogs have similar success rates during transcatheter PDA occlusion. Roughly 1 in 5 patients of both species have congestive heart failure at the time of catheterization. Anti‐coagulation is more commonly used in humans than dogs.

## Abstract C25: Amplatz Canine Duct Occluder for Patent Ductus Arteriosus Occlusion in Dogs: Outcomes and Complications

### **Marisa Ciccozzi**; Christopher Stauthammer, DVM, DACVIM; Allison Masters, DVM, DACVIM; Cassidy Coats; Sydney Oberholtzer; Emily Gavic; Anthony Tobias

#### University of Minnesota, Minneapolis, MN, USA

**Background:** The Amplatz Canine Duct Occluder (ACDO) is routinely used for Patent Ductus Arteriosus (PDA) occlusion. Limited data is available on clinical outcomes and success rates with ACDO device placement.

**Objectives:** To report clinical outcomes and complications of standard ACDO procedures in a large cohort of dogs.

**Animals:** Dogs presenting to the University of Minnesota from 2008–2022 that underwent ACDO device placement for PDA occlusion.

**Methods:** Retrospective analysis of outcomes and complications in dogs receiving the ACDO device. Echocardiography was performed prior to occlusion, 24‐h post‐occlusion and during follow‐up recheck visits. Procedural success was determined by occlusion of flow on angiography, color, and spectral Doppler.

**Results:** 181 dogs underwent ACDO device placement. Dogs had a median weight of 6.5 kg (range 2.55–60.5 kg) and required a median device size of 6 mm (range 3–14 mm). At 24‐h post‐occlusion, 4.4% (8/181) had trivial flow through the device. Trivial flow was noted on recheck in 6% (7/116) of patients with a median follow‐up time of 116 days (range 24–4595 days). Complications occurred in 6.1% (11/181), with 2% (5/181) considered minor. Major complications occurred in 3.3% (6/181) including device embolization (*n =* 1), hemorrhage from femoral access site requiring transfusion (*n =* 1), cardiac arrest postoperatively (*n =* 1), femoral artery tear (*n =* 1), external iliac artery tear (*n =* 1), and wire perforation of the aorta (*n =* 1). Overall procedural mortality rate was 1.1% (2/181).

**Conclusions:** The high procedural success and low complication rates support use of the ACDO device as a safe and effective treatment for PDA occlusion in dogs.

## Abstract C26: Hemodynamic, Echocardiographic, and Sedative Effects of Oral Trazodone in Healthy Dogs

### **Erin McCarragher** ^1^; Katherine Scollan^2^, DVM, DACVIM (Cardiology)

#### ^1^Oregon State University, Corvallis, OR, USA; ^2^Associate Professor, Cardiology, Oregon State University, Corvallis, OR, USA

**Background:** Trazodone, a serotonin antagonist and reuptake inhibitor (SARI) with alpha‐adrenergic blocking effects, is an anxiolytic increasingly administered to dogs prior to veterinary visits. While trazodone appears to be well tolerated in dogs, its effects on echocardiographic and hemodynamic parameters are largely unknown.

**Hypothesis/Objectives:** To assess the effects of trazodone on blood pressure (BP), heart rate (HR), echocardiographic measurements, and sedation level in healthy dogs.

**Animals:** 31 adult dogs considered healthy based on physical exam (PE), M‐mode and two‐dimensional (2D) echocardiography, electrocardiogram (ECG), BP, blood chemistry, and urinalysis.

**Methods:** Prospective, double‐blinded clinical trial. Dogs were randomized to receive standard (3–5 mg/kg) or high dose (10–12 mg/kg) trazodone at home 120–180 min prior to presentation. A washout period of at least 48 h was mandated between doses. Sedation level, PE, BP, and echocardiography were performed at each visit.

**Results:** No clinically significant differences were found between baseline, standard, or high dose trazodone for HR, BP, or echocardiographic parameters including linear and volumetric left atrial and ventricular size and indices of systolic and diastolic function. There was a significant increase in sedation score between baseline (median 0, range 0–1) and standard dose (median 1, range 1–3) and between baseline and high dose (median 1, range 1–3) with no significant difference between standard and high dose.

**Conclusion:** A single dose of trazodone up to 12 mg/kg produces mild sedative effects in dogs within 120–180 min of administration and clinically insignificant changes to echocardiographic measurements, BP, and HR.

## Abstract C27: Short‐Term Hemodynamic Effects of Pimobendan in Dogs With Preclinical Patent Ductus Arteriosus

### **Lindsey M. Humphries**; Ryan Fries; Wallace Gabrielle; Emily Javery; Saki Kadotani; Leah Kruckman; Sumana Prabhakar; Todd Sumerfield

#### University of Illinois

**Background:** Patent ductus arteriosus (PDA) is the most common cardiac congenital defect in dogs and with timely correction carries a good prognosis. If closure is delayed, the risk for heart failure and death increase. This study evaluated the short‐term hemodynamic effects of pimobendan in dogs with preclinical left‐to‐right shunting PDA.

**Hypothesis:** Pimobendan will provide hemodynamic benefits in dogs with left‐to‐right shunting PDA.

**Animals:** 17 dogs with preclinical PDA.

**Methods:** For this prospective observational cohort study, patients were evaluated via physical examination, thoracic radiographs, quantitative N‐terminal pro‐brain natriuretic peptide, and transthoracic echocardiography. Dogs were eligible based on the following criteria: echocardiographic evidence of a PDA (left‐to‐right) regardless of anatomic classification, the presence of cardiomegaly defined as left atrial (LA) and left ventricular (LV) size exceeding the 95% predictive interval, and radiographic cardiomegaly (VHS >10.5) without concurrent pulmonary edema. All patients received pimobendan (0.4–0.6 mg/kg/day divided into q 12 h dosing). Re‐evaluation of all tests was performed on day 7 prior to PDA closure. Data were assessed for normality using a Shapiro‐Wilk test and pre‐post variables were compared using a paired t‐test or a Wilcoxon signed‐rank test when appropriate. A *P* < 0.05 was considered significant.

**Results:** No patients decompensated during the study and significant differences in VHS (−0.2), LA volume (−0.31 mL/kg), normalized LV diastolic (−0.45 mL/kg) and systolic (−0.54 mL/kg) volume, LV ejection fraction (+6.1%), and NT‐proBNP (−332 pmol/L) were observed.

**Conclusion and Clinical Importance:** Pimobendan may provide short‐term hemodynamic benefit in preclinical PDA patients.

## Abstract C28: Feasibility of Shear Wave Elastography/Dispersion Imaging in Dogs With Cardiac Disease

### **Nozomi Shiohara** ^1^; Kensuke Nakamura^2^; Masahiro Tamura^3^; Nozomu Yokoyama^2^; Yong Bin Teoh^4^; Mitsuyoshi Takiguchi^5^

#### ^1^Hokkaido University, Sapporo, Japan; ^2^Associate Professor, Hokkaido University, Sapporo, Japan; ^3^Associate Professor, Rakuno Gakuen University, Ebetsu, Japan; ^4^Academic Fellow, Hokkaido University, Sapporo, Japan; ^5^Professor, Hokkaido University, Sapporo, Japan

**Background:** Cardiac disease cause hepatic congestion and fibrosis, which increase liver stiffness (LS). LS can be estimated by 2D‐Shear Wave Elastography (SWE), which measures shear wave speed (SWS), an index of viscoelasticity and dispersion slope (DS), an index of viscosity. In the liver, fibrosis elevates elasticity, while congestion or inflammation elevates viscosity.

**Hypothesis/Objectives:** To analyze SWE‐measured LS in dogs with cardiac disease and to identify its determinants.

**Animals:** Sixty‐five client‐owned dogs with cardiac disease.

**Methods:** In this cross‐sectional prospective study, SWS and DS were measured using 2D‐SWE in all dogs along with the short to long diameter ratio of the caudal vena cava (CVC SD/LD), an index of congestion, and the left ventricular outflow tract velocity time integral (LVOT‐VTI), an index of cardiac output by echocardiography. The discriminatory ability of these parameters for right heart failure (RHF) was compared using ROC analysis. The relation of SWS and DS to other echocardiographic indices was evaluated by multiple regression analysis.

**Results:** SWS, DS, and CVC SD/LD were all significantly higher, while LVOT‐VTI was significantly lower in the dogs with RHF. DS had the highest accuracy in discriminating RHF (AUC = 0.998), followed by SWS (AUC = 0.992). In multiple regression analysis, CVC SD/LD was the most related factor to the SWS and DS followed by LVOT‐VTI.

**Conclusions and Clinical Importance:** Elevated SWS and DS in dogs with RHF reflects congestion and reduced cardiac output. 2D‐SWE may be useful to predict the presence of hepatic congestion and RHF in dogs with cardiac disease.

## Abstract C29: Agreement of Echocardiographic‐ and Catheterization‐Based Methods of Transpulmonary Pressure Gradient Measurement in Dogs

### **Katherine E. Murphy** ^1^; Lauren Markovic^2^, DVM, DACVIM (Cardiology); Darcy Adin^3^, DVM, DACVIM (Cardiology); Karen Moy‐Trigilio^4^, DVM; Amanda Coleman^5^, DVM, DACVIM (Cardiology)

#### ^1^College of Veterinary Medicine, University of Georgia, Athens, GA, USA; ^2^Assistant Professor of Cardiology, Department of Small Animal Medicine and Surgery, College of Veterinary Medicine, University of Georgia, Athens, GA, USA; ^3^Clinical Professor—Cardiology, Small Animal Clinical Sciences, College of Veterinary Medicine, University of Florida, Gainesville, FL, USA; ^4^Intern, College of Veterinary Medicine, University of Florida, Gainesville, FL, USA; ^5^Associate Professor of Cardiology, Department of Small Animal Medicine and Surgery, College of Veterinary Medicine, University of Georgia, Athens, GA, USA

**Background:** Pulmonary valve stenosis (PS) is often treated with balloon pulmonary valvuloplasty (BPV) if moderate or severe. While Doppler echocardiographic assessment of transpulmonary pressure gradient (PG) serves as a surrogate for catheter‐based measurements and helps determine transcatheter indication, the agreement of these techniques have not been systemically evaluated in dogs.

**Objective/Hypothesis**: The objective of this study was to describe agreement between Doppler echocardiographic methods of transpulmonary pressure gradient estimation and peak‐to‐peak pressure gradient at catheterization (PGcath) in dogs. We hypothesized that with reference to PGcath, mean echocardiographic PG (PGecho‐mean) would have less bias than peak modal instantaneous echocardiographic PG (PGecho‐peak).

**Animals:** Dogs with congenital PS that underwent BPV at one of two teaching hospitals.

**Methods:** Cases that underwent echocardiography and subsequent BVP separated by <30 days were retrospectively identified. For each echocardiogram, measurements from Doppler recordings were used to determine average PGecho‐mean and PGecho‐peak from 3–5 consecutive beats. Peak‐right‐ventricular‐to‐peak‐pulmonary‐artery pressure gradients (PGcath) were obtained from catheterization reports. Bland‐Altman analysis was used to assess agreement between echocardiographic and catheterization data.

**Results:** Data from 205 cases were evaluated. Relative to PGcath, bias (95% limits of agreement) for PGecho‐mean and PGecho‐peak were −19.9 mm Hg (−93.9 to 54.0 mm Hg) and 37.92 mm Hg (−39.8 to 115.7 mm Hg), respectively. Proportional bias, greater at higher gradients, was observed for both methods.

**Conclusions and Clinical Importance:** On average, PGecho‐mean and PGecho‐peak under‐ and over‐estimated PGcath, respectively, with wide limits of agreement for both. Over‐reliance on echocardiographic PG for assessment of PS severity might be problematic.

## Abstract C30: Multiomic, Histopathologic, and Clinicopathologic Assessment of Chronic, Weekly, Oral Rapamycin Administration in HCM‐Affected Cats

### **Victor N. Rivas** ^1^; Carina Jauregui^1^, RVT, RLAT; Joanna Kaplan^1^, DVM, DACVIM (Cardiology); Louise Grubb^2^, BSc, MBS; Susan Kennedy^2^, BSc, PhD; Stuart Fitzgerald^2^, MVB, MANZCVS; Samantha Harris^3^, PhD; Joshua Stern^4^, DVM, PhD, DACVIM (Cardiology)

#### ^1^School of Veterinary Medicine, University of California‐Davis, Davis, CA, USA; ^2^TriviumVet, Waterford, Ireland; ^3^University of Arizona, Tucson, AZ, USA; ^4^Associate Dean and Professor of Cardiology, Department of Medicine and Epidemiology, School of Veterinary Medicine, University of California‐Davis, Davis, CA, USA

**Background:** Therapies to treat and prevent feline hypertrophic cardiomyopathy (HCM) are lacking. The mammalian target of rapamycin (mTOR) is a master regulator of cell growth, metabolism, and protein synthesis, and represents a therapeutic target for HCM. A recent clinical trial demonstrated that mTOR inhibition via chronic delayed‐release (DR) rapamycin administration halts progression of left ventricular wall thickness with no adverse events or clinical pathology derangements, however, molecular effects of rapamycin in cats with HCM remain unknown.

**Hypothesis/Objectives:** This study aimed to characterize the multiomic effects of weekly low‐ and high‐dose, oral, DR rapamycin in HCM‐affected colony cats via RNA‐sequencing and proteomic profiling. We further aimed to assess clinicopathologic, cardiac biomarker, and histopathologic responses to therapy.

**Animals:** Twelve purpose‐bred cats were studied (3 low‐dose HCM+, 3 high‐dose HCM+, 3 untreated HCM+, and 3 untreated control).

**Methods:** Plasma and urine samples were obtained at day 0 and 56 of DR rapamycin dosing. Tissue samples (left ventricular posterior wall and interventricular septum) were obtained at necropsy on day 60. RNA‐sequencing was performed on all tissues and LC‐MS profiling was performed on day 0 and 56 plasma and urine and day 60 tissues.

**Results:** Differential transcript and relative protein expression analyses revealed intrinsic molecular effects between and within treatment groups with no safety or tolerability concerns identified via clinical pathology.

**Conclusions and Clinical Importance:** Results highlight pathways impacted in HCM‐affected cats receiving oral DR rapamycin and provide key insights into multiomic differences observed in disease.

## Abstract C32: The Cardiovascular Effects of Zenalpha in Ventricular Tachy‐Paced Dogs in Mild to Moderate Heart Failure

### **William Muir** ^1^; Heta Turunen^2^; Sydney St. Clair^1^; Nancy Zimmerman^3^; Steve Roof^1^; Caryn Thompson^3^; Robert Hamlin^1^

#### ^1^QTest Labs, Columbus, OH, USA; ^2^Vetcare; ^3^Dechra

**Background:** Zenalpha (medetomidine‐vatinoxan) is a sedative‐analgesic for dogs containing the novel molecule, vatinoxan, which counteracts medetomidine's cardiovascular depressive effects. To date, the use of Zenalpha has been investigated in healthy dogs and its safe use in cardiac patients is to be determined.

**Hypothesis/Objectives:** The objective was to compare the cardiovascular effects of Zenalpha to dexmedetomidine in dogs with mild to moderate heart failure.

**Animals:** Seven purpose‐bred, tachy‐paced Beagles with ejection fraction of 45 ± 5% including mild concomitant left‐ventricular enlargement.

**Methods:** In this blinded crossover study each dog received Zenalpha (0.25 mg/m^2^ medetomidine and 5 mg/m^2^ vatinoxan) or dexmedetomidine (0.25 mg/m^2^) intramuscularly. Heart rate (HR), ECG and mean arterial pressure (MAP) were recorded and cardiac output measured at intervals until 120 min post‐treatment. Differences between treatments and baseline were evaluated with repeated measures analysis (*P* < 0.05).

**Results:** Compared to dexmedetomidine stroke volume (SVI) and cardiac indices (CI) were significantly higher with Zenalpha until 60‐ and 120‐min post‐treatment, respectively. SVI did not decrease significantly from baseline with Zenalpha. CI and HR decreased significantly from baseline with both treatments. With Zenalpha CI and HR returned to baseline at 120 and 90 min, respectively, and HR was significantly higher than with dexmedetomidine at 90‐ and 120‐min post‐treatment. Compared to dexmedetomidine MAP was significantly lower from 15 to 90 min post‐treatment with Zenalpha remaining below baseline until 120 min.

**Conclusions and Clinical Importance:** In comparison to dexmedetomidine Zenalpha caused less detrimental cardiovascular changes in dogs with mild to moderate heart failure.

**Table:** **Table 1.** Cardiac and stroke volume indices, heart rate and mean arterial pressure (mean [SD]) after dexmedetomidine (0.25 mg/m^2^) or Zenalpha (medetomidine 0.25 mg/m^2^ and vatinoxan 5 mg/m^2^) IM treatments given at timepoint 0 min in tachy‐paced Beagles (*n =* 7) with ejection fraction of 45 ± 5% including mild concomitant left‐ventricular enlargement.

		Timepoint (min)							
Variable	Treatment	Baseline	10	20	30	40	60	90	120
CI	Dexmedetomidine	5.1 (0.3)	2.2 (0.2)*^¤^	2.2 (0.2)*^¤^¤ (*n =* 6)	2.2 (0.2)*^¤^	2.2 (0.2)*^¤^	2.2 (0.2)*^¤^	2.3 (0.1)*^¤^	2.1 (0.2)*^¤^
	Zenalpha	4.6 (0.3)	3.1 (0.2)*	3.5 (0.2)*	3.7 (0.2)*	3.6 (0.2)*	3.5 (0.2)*	3.8 (0.2)*	4.9 (0.2)

		Timepoint (min)							
Variable	Treatment	Baseline	10	20	30	40	60	90	120
SVI	Dexmedetomidine	43.1 (1.8)	27.0 (0.8)*^¤^	26.4 (1.1)*^¤^ (*n =* 6)	26.3 (0.8)*^¤^	27.7 (1.4)*^¤^	32.3 (1.1)*^¤^	36.7 (1.0)*	37.9 (3.0)
	Zenalpha	40.5 (1.9)	39.5 (1.6)	41.2 (1.9)	39.5 (3.4)	42.1 (2.0)	41.4 (2.1)	35.8 (2.7)	42.8 (3.2)
HR	Dexmedetomidine	117.7 (5.0)*	81.1 (6.3)*	85.1 (5.4)*	82.4 (5.7)*	79.4 (6.4)*	69.3 (6.5)*	63.0 (4.7)*^¤^	56.7 (3.5)*^¤^
	Zenalpha	115.9 (7.6)	79.3 (5.7)*	87.2 (6.2)*	99.8 (15.5)	87.9 (7.1)*	86.3 (6.5)*	108.3 (6.9)	117.7 (8.3)

Variable	Treatment	Baseline	15	25	35	45	60	90	120
MAP	Dexmedetomidine	113.8 (6.7)	137.8 (7.4)*^¤^	137.7 (7.3)*^¤^	135.6 (7.6)*^¤^	132.5 (7.3)*^¤^	122.6 (6.7)^¤^	113.5 (5.9)^¤^	105.9 (4.3)
	Zenalpha	112.9 (3.8)	98.1 (6.8)*	80.8 (4.1)*	75.9 (3.7)*	72.4 (2.8)*	73.9 (5.3)*	96.4 (3.3)*	100.8 (3.1)*

^¤^ Difference between treatment groups significantly different (*P* < 0.05)

* Mean values for the treatment group significantly different from baseline (*P* < 0.05)

*n =* 7 unless otherwise indicated

## Abstract C33: Allosteric Inhibitors Targeting the Calmodulin‐pip2 Interface of SK4 K^+^ Channels for Atrial Fibrillation Treatment

### **Shira Burg** ^1^; Shir Shapiro^2^; Asher Peretz^1^; Elvira Haimov^1^; Boris Redko^1^; Adva Yeheskel^1^; Luba Simhaev^1^; Hamutal Engel^1^; Avi Raveh^1^; Ariel Ben‐Bassat^1^; Michael Murninkas^2^; Rotem Polak^2^

#### ^1^Tel Aviv University, Tel Aviv‐Yafo, Israel; ^2^Ben‐Gurion University of the Negev, Be’er Sheva, Israel

The Ca 2+ ‐activated SK4 K+ channel is gated by Ca 2+ ‐calmodulin (CaM) and is expressed in immune cells, brain, and heart. A cryo‐EM structure of the human SK4 K+ channel recently revealed four CaM molecules per channel tetramer, where the apo CaM C‐lobe and the holo CaM N‐lobe interact with the proximal C‐terminus and the linker S4‐S5, respectively, to gate the channel. Here, we show that phosphatidylinositol 4–5 bisphosphate (PIP2) potently activates SK4 channels by docking to the boundary of the CaM binding domain. A novel allosteric blocker, BA6b9, was designed to act to the calmodulin‐PIP2 binding domain, a previously untargeted region of SK4 channels, at the interface of the proximal C‐terminus and the linker S4‐S5. Molecular docking and patch‐clamp electrophysiology indicate that BA6b9 inhibits SK4 channels by interacting with two specific residues, Arg191 and His192 in the linker S4‐S5, not conserved in SK1‐SK3 subunits, thereby conferring selectivity and preventing the Ca 2+ ‐CaM N‐lobe to properly interact with the channel linker region. Immunohistochemistry of the SK4 channel protein in rat hearts showed a widespread expression in the sarcolemma of atrial myocytes, with a sarcomeric striated Z‐band pattern, and a weaker occurrence in the ventricle but a marked incidence at the intercalated discs. BA6b9 significantly prolonged atrial and atrioventricular effective refractory periods in rat isolated hearts and reduced atrial fibrillation induction *ex vivo*. Our work suggests that inhibition of SK4 K+ channels by targeting drugs to the calmodulin‐PIP2 binding domain provides a promising anti‐arrhythmic therapy.

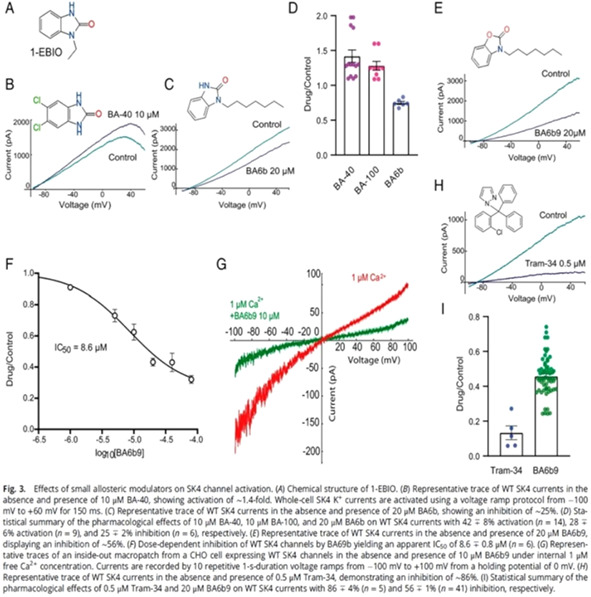

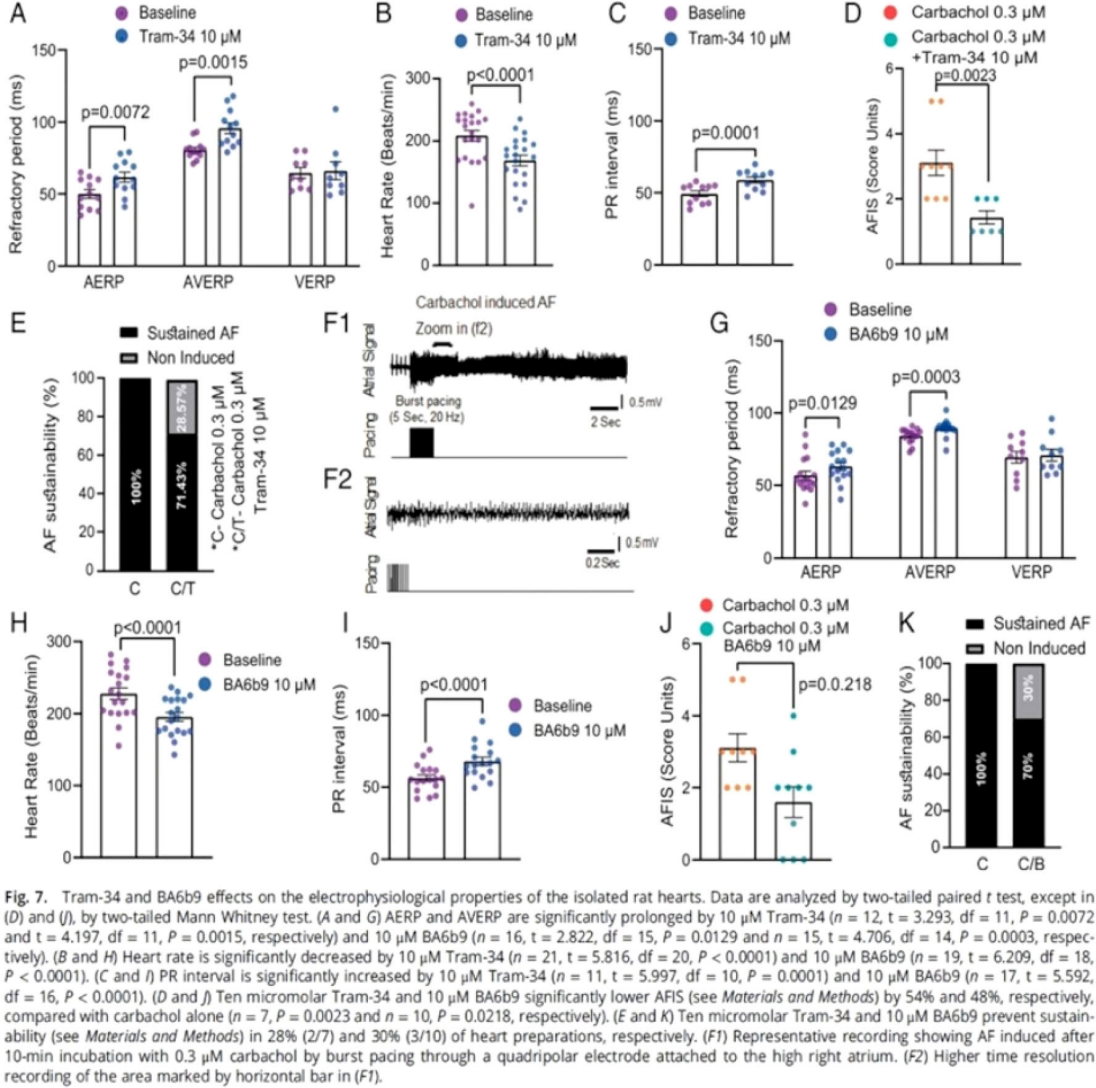

## Abstract C34: Association Between Mitral Insufficiency Echocardiographic (MINE) Score and Mitral Valve Repair Outcome

### **Kentaro Kurogochi**; Masami Uechi

#### JASMINE Veterinary Cardiovascular Medical Center

**Background:** Mitral valve repair (MVR) could be a curative treatment for myxomatous mitral valve disease (MMVD) in dogs. However, the optimal timing for surgery is not known. Mitral insufficiency echocardiographic (MINE) score is an easy‐to‐use echocardiographic based classification used to assess the severity of MMVD in dogs for medical treatment.

**Hypothesis/Objectives:** We hypothesized that the MINE score can be used for estimating the outcome of MVR.

**Animals:** We retrospectively analyzed the data of dogs with MMVD that underwent MVR at our institute from February 2017 to December 2020. We excluded cases that did not have enough data required for calculating the MINE score.

**Methods:** Echocardiographic variables were used to classify the cases into four stages preoperatively using the MINE scoring system: mild (score: 4–5), moderate (score: 6–7), severe (score: 8–12), and late (score: 13–14). Thoracic radiographs and echocardiography were evaluated in the preoperative period, 6‐ and 12‐months post‐surgery.

**Results:** The study included 897 dogs (mild = 16, moderate = 121, severe = 679, late = 81). The higher the disease stage, the larger the heart size both pre‐ and post‐surgery. The four groups did not have difference in two‐year survival rate for all‐cause mortality derived by Kaplan‐Meier curve: 92%, 77%, 85%, and 87%, respectively (*P* = 0.52; Log‐rank test).

**Conclusions and Clinical Importance:** The MINE score can be used to predict the extent of cardiac reverse remodeling. However, it is unrelated to postoperative mortality. Therefore, estimating surgical outcomes require comprehensive factors, including clinical signs, arrhythmia, and medications.

## Abstract C35: Relationship of Serum Osmolality to the Degree of Mathematical Chloride Correction and Heart Disease Stage

### **Edward Daly** ^1^; Autumn Harris^2^, DVM, DACVIM (SAIM); Darcy Adin^3^, DVM, DACVIM (Cardiology)

#### ^1^University of Florida, Gainesville, FL, USA; ^2^Assistant Professor, Small Animal Clinical Sciences, University of Florida, Gainesville, FL, USA; ^3^Clinical Professor, Small Animal Clinical Sciences, University of Florida, Gainesville, FL, USA

**Background:** Heart failure‐associated hypochoremia can be depletional or dilutional from free water retention. Serum osmolality reflects free water, but has not been evaluated in dogs with heart disease.

**Hypothesis/Objectives:** To determine if serum osmolality is related to heart disease stage and the amount of mathematical correction of serum chloride (Cl‐) concentrations in healthy dogs and dogs with myxomatous mitral valve degeneration (MMVD).

**Animals:** Seventy‐seven dogs (20 healthy, 25 Stage B MMVD, 32 Stage C/D MMVD).

**Methods:** Serum Cl‐ was mathematically corrected. Osmolality was calculated (cOsm) and directly measured by freezing point depression (dmOsm) and compared by Bland‐Altman analysis. Biochemical variables and osmolarity were compared among healthy, Stage B and Stage C/D dogs. Correlations were explored between osmolality and biochemical variables. Median and IQR are presented and *P* < 0.05 was considered significant.

**Results:** The cOsm was different among groups (*P* = 0.003), with Stage B (310 mOsm/kg; 306, 316) and C/D dogs (312 mOsm/kg; 308, 319) having higher cOsm than healthy dogs (305 mOsm/kg; 302, 308). Osmolality methods were moderately correlated (*P* < 0.0001, *r =* 0.46) but with proportional bias and poor agreement. The amount of Cl‐ correction was negatively correlated with cOsm (*P* < 0.0001, *r =* −0.78) and dmOsm (*P* = 0.004, *r =* −0.33). Serum bicarbonate was negatively correlated with Cl‐ (*P* < 0.0001, *r =* −0.67).

**Conclusions and Clinical Importance:** Dogs with Stage B and Stage C/D heart disease had higher cOsm than healthy dogs. Osmolality was inversely related to the amount of Cl‐ correction which supports its use in the assessment of relative free water. Poor agreement between cOsm and dmOsm prevents methodological interchange.

## Abstract C37: Loop‐Mediated Isothermal Amplification PCR in Place of Modified Knott's Test Screening Dogs for Heartworm Infection

### **Jae Chun Cho** ^1^; Chul Park^2^

#### ^1^BON Animal Medical Center, Suwon, Republic of Korea; ^2^Jeonbuk National University, Jeonju, Republic of Korea

**Background:** The current diagnostic recommendations for *Dirofilaria immitis* (*D. immitis*) infection in dogs rely on detecting antigens primarily produced by adult females, combined with microfilariae (mf) detection tests.

**Hypothesis/Objectives:** This study aims to compare LAMP PCR and the Modified Knott's (MK) test for mf detection, used in conjunction with antigen detection tests, to screen for canine heartworm infection in shelter dogs.

**Animals:** Matched whole blood samples were collected from 35 shelter dogs in Gunsan, Jeollabukdo (Republic of Korea), between June and October 2022. The study was approved by the Institutional Animal Care and Use Committee (IACUC).

**Methods:** Blood samples were analyzed for the presence of mf using the MK test and for *D. immitis* DNA using a species‐specific LAMP PCR.

**Results:** Among the 10 antigen kit‐positive animals, 4 (11.4%; 4/35) were found to have microfilariae morphologically identified as *D. immitis*. In comparison, LAMP PCR detected *D. immitis* DNA in two additional heartworm‐positive samples, accounting for 17.1% (6/35).

**Conclusion and Clinical Importance:** Although LAMP PCR was not statistically significant compared to the MK test, it detected a greater number of *D. immitis* cases and showed more consistency with the results of antigen detection tests. In clinical use, LAMP PCR offers advantages over the MK test, as it does not require a microscope and can be easily employed with any blood sample, similar to advanced antigen test kits, regardless of observer subjectivity. Consequently, in conjunction with antigen testing, LAMP PCR could serve as an essential screening test for *D. immitis* infection in canine populations.

## Abstract C38: Serum Angiopoietin‐1 and Angiopoietin‐2 of Dogs With Myxomatous Mitral Valve Disease and Pulmonary Hypertension

### **Minseok Choi**; Hogeun Yu; Dohee Lee; Taesik Yun; Yoonhoi Koo; Yeon Chae; Byeong‐Teck Kang; Mhan‐Pyo Yang; Hakhyun Kim

#### Laboratory of Veterinary Internal Medicine, Veterinary Teaching Hospital, Chungbuk National University, Cheongju, Republic of Korea

**Background:** Angiopoietin (Ang) plays a key role in regulating vascular integrity. Ang‐2 is considered a potential biomarker for cardiovascular diseases in humans.

**Objectives:** To evaluate the relationships between the severity of MMVD and serum Ang‐1 and Ang‐2 concentrations and between PH probability and serum Ang‐1 and Ang‐2 concentrations in dogs with MMVD.

**Animals:** Seventy‐four dogs (control, *n =* 12; MMVD, *n =* 62) were included in this study.

**Methods:** We retrospectively reviewed the medical data of dogs diagnosed with MMVD at our institution between July 2018 and June 2022. The serum Ang‐1 concentration was analyzed using a commercial canine‐specific ELISA kit. Serum Ang‐2 concentration was analyzed using a commercial Human ELISA test kit and validated for use in dogs.

**Results:** The median serum Ang‐1 concentration did not differ among the study groups. The median serum Ang‐2 concentration was significantly higher in dogs with stage B2 MMVD (*P* = 0.022) and acute CHF (*P* = 0.001) than in control dogs. In addition, the mean serum Ang‐2 concentration was significantly higher in dogs with high PH probability than in those with a low probability (*P* < 0.001) and intermediate PH probability (*P* = 0.001).

**Conclusions and Clinical Importance:** Circulating Ang‐2 levels significantly increase in dogs with advanced MMVD and high PH probability, suggesting that Ang‐2 may play a role in endothelial dysfunction in dogs with advanced heart disease.

**Figure:**
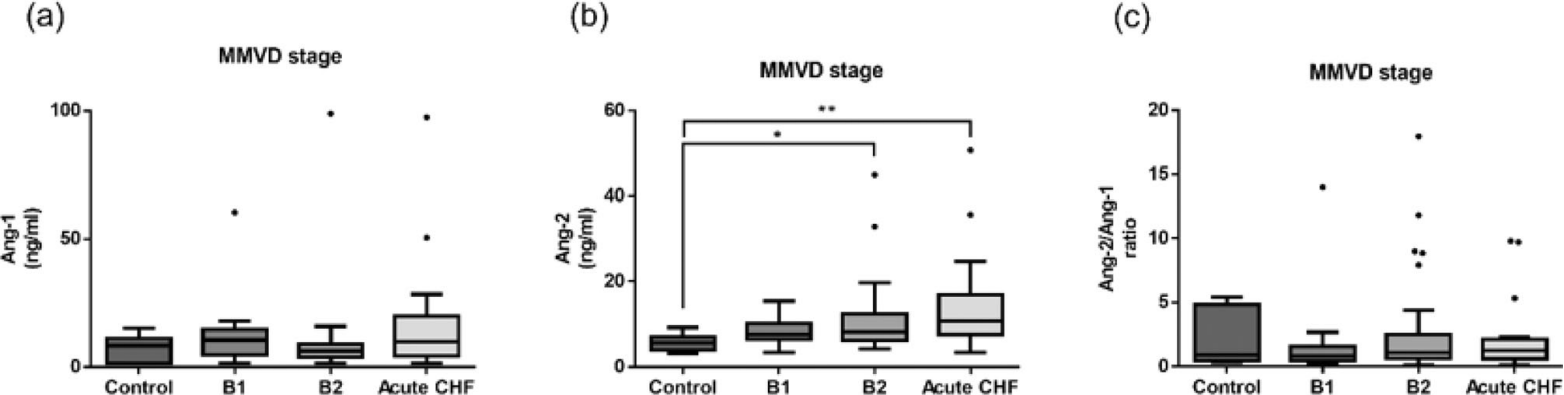
Figure 1

**Figure:**
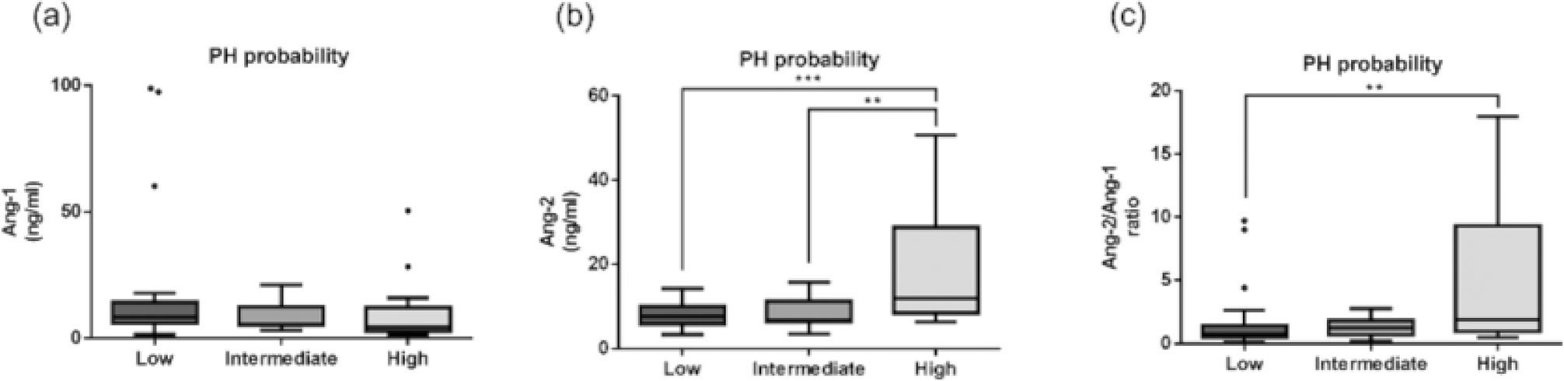
Figure 2

**Figure:**
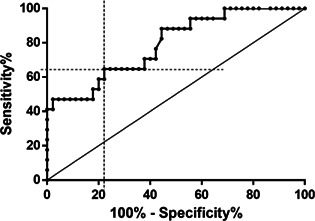
Figure 3

**Figure:**
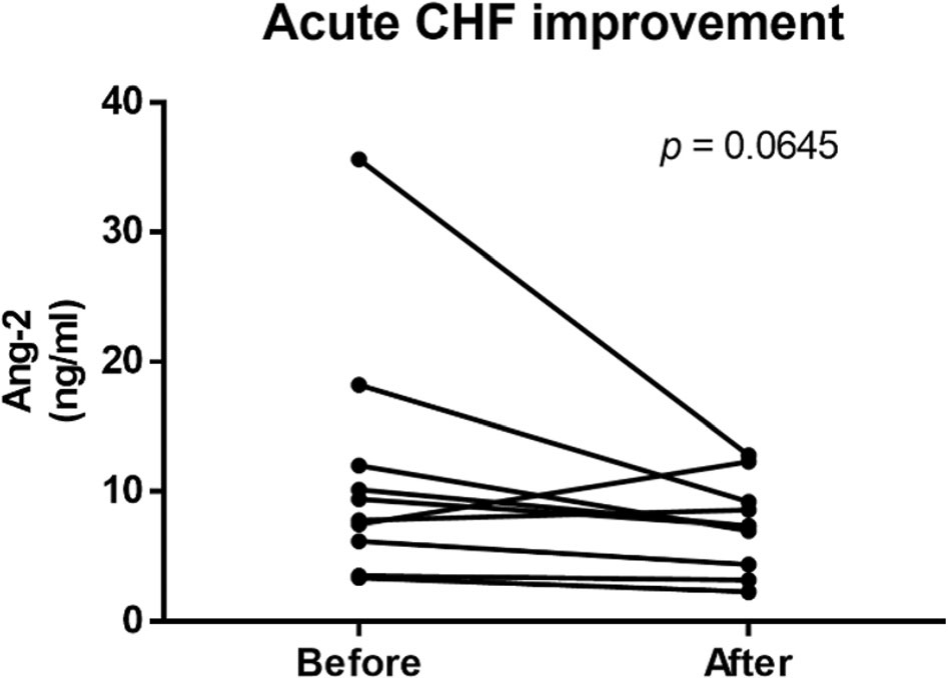
Figure 4

## Abstract C39: Red Blood Cell Morphologic Parameters and Indices in Dogs With Dilated Cardiomyopathy Differs Between Diets

### **Lisa Freeman** ^1^, DVM, PhD, DACVIM (Nutrition); Francisco Conrado^1^, DVM, MSc, DACVP; Shir Gilor^2^, DVM, MSc, DACVP; Darcy Adin^2^, DVM, DACVIM (Cardiology); Jillian Hojsak^1^; John Rush^1^, DVM, MS, DACVIM (Cardiology), DACVECC

#### ^1^Cummings School of Veterinary Medicine, Tufts University, North Grafton, MA, USA; ^2^College of Veterinary Medicine, University of Florida, Gainesville, FL, USA

**Background:** Diet‐associated DCM can occur in dogs eating non‐traditional diets (NTDs) high in pulses, but these diets also have been associated with changes in the erythron. Healthy laboratory dogs eating a high pea/lentil diet for one month had significant decreases in red blood cell count (RBC), hematocrit, and hemoglobin (Bakke et al 2022). Decreases in RBC also were seen in two other studies in healthy dogs eating high pulse diets (Quilliam et al 2021; Reis et al 2021), but the cause is unknown.

**Hypothesis/Objectives:** To compare red blood cell morphologic parameters and indices between dogs with DCM eating NTDs or TDs.

**Animals:** Forty‐six dogs with DCM: 38 eating NTDs (pulses in the top ten ingredients) and 8 eating TDs (no pulses in the top ten ingredients).

**Methods:** RBC parameters were recorded from a complete blood count from the time of DCM diagnosis. A board‐certified veterinary clinical pathologist (FC) blinded to dogs’ diets evaluated RBC indices. Variables were compared between diet groups using chi‐square and Mann‐Whitney U tests.

**Results:** RBC number (*P* = 0.72), hematocrit (*P* = 0.63), and hemoglobin (*P* = 0.59) were not significantly different between diet groups. However, red blood cell distribution width (RDW) was significantly lower in dogs eating NTDs compared to TDs (*P* = 0.01). Some RBC indices were more prevalent in the NTD group (e.g., poikilocytes, echinocytes, keratocytes, elliptocytes, and eccentrocytes), although none reached statistical significance.

**Conclusions and Clinical Importance:** RBC indices and lower RDW in dogs eating NTDs may provide clues to pathogenesis of diet‐associated DCM.

## Abstract C40: Comparison of Electrocardiographic and Echocardiographic Findings in Irish Wolfhounds Eating Non‐Traditional or Traditional Diets

### **Lily Coppinger** ^1^; Lisa Freeman^2^, DVM, PhD, DACVIM (Nutrition); William Tyrrell^3^, DVM, DACVIM (Cardiology); Steven Rosenthal^3^, DVM, DACVIM (Cardiology); Mariellen Dentino^4^, MD; Frances Abrams^4^; John Rush^2^, DVM, MS, DACVIM (Cardiology), DACVECC

#### ^1^Cummings School of Veterinary Medicine, Tufts University, North Grafton, MA, USA; ^2^Professor, Cummings School of Veterinary Medicine, Tufts University, North Grafton, MA, USA; ^3^CVCA Cardiac Care for Pets; ^4^Irish Wolfhound Foundation

**Background:** Apparently healthy dogs eating non‐traditional diets (NTDs) have larger left ventricular diameter, lower systolic function, and more ventricular premature complexes (VPCs) compared to dogs eating traditional diets (TDs). It is unknown whether this finding also occurs in Irish Wolfhounds.

**Hypothesis/Objectives:** To compare electrocardiographic and echocardiographic findings between Irish Wolfhounds eating NTDs and TDs.

**Animals:** Irish Wolfhounds that had echocardiography performed at dog shows between 2018–2020.

**Methods:** Demographic information, echocardiographic measurements, cardiac rhythm (from a rhythm strip), and main diet (i.e., the diet providing each dog's main source of calories) were recorded. Diets were classified as NTDs if the diet contained any pulses in the top ten ingredients and as TDs if there were no pulses in the top ten ingredients.

**Results:** One hundred forty‐eight Irish Wolfhounds had echocardiograms performed during this time period. Dogs were excluded because of pre‐existing heart disease (*n =* 12), raw or homemade diet (*n =* 28), and unknown diet (*n =* 9). Of the 96 eligible dogs, 35 were eating NTDs (median pulse score = 42 [range, 16–104]) and 61 were eating TDs (median pulse score = 0 [range, 0–11]; *P* < 0.001). There was no significant difference in age, sex, or body condition score. There were no significant differences in echocardiographic measurements between the two diet groups. However, 6/35 dogs eating NTDs (17%) had VPCs compared to 1/61 dogs eating TDs (2%; *P* = 0.009).

**Conclusions and Clinical Importance:** Similar to a previous study of four dog breeds, Irish Wolfhounds eating NTDs had significantly more VPCs compared to dogs eating TDs.

## Abstract C41: Neutrophil‐to‐Lymphocyte, Monocyte‐to‐Lymphocyte, and Platelet‐to‐Lymphocyte Ratios as Inflammatory Biomarkers in Dogs with Myxomatous Mitral Valve Disease

### **Chaerin Kim**; Yeon Chae; Byeong‐Teck Kang; Hakhyun Kim; Yoonhoi Koo; Dayoung Ku; Dohee Lee; Mhan‐Pyo Yang; Taesik Yun

#### Laboratory of Veterinary Internal Medicine, College of Veterinary Medicine, Chungbuk National University, Cheongju, Republic of Korea

**Background:** In human, neutrophilia is linked to the development of acute heart failure, and lymphopenia is associated with poor prognosis. The neutrophil‐to‐lymphocyte ratio (NLR), monocyte‐to‐lymphocyte ratio (MLR), and platelet‐to‐lymphocyte ratio (PLR) are newly proposed inflammatory biomarkers. As MMVD is the most common acquired heart disease in dogs, evaluation of inflammatory biomarkers is needed.

**Objectives:** To investigate NLR, MLR, and PLR in dogs with MMVD.

**Animals:** In total, 106 dogs with MMVD and 22 healthy dogs were included in the study.

**Methods:** Complete blood count data were obtained retrospectively, and NLR, MLR, and PLR were compared between dogs with MMVD and healthy dogs.

**Results:** NLR and MLR were significantly higher in dogs with MMVD C and D (NLR of 4.99 [3.69–7.27]; MLR of 0.56 [0.36–0.74]) than in healthy dogs (NLR: 3.05 [1.82–3.37], *P* < 0.001; MLR: 0.21 [0.14–0.32], *P* < 0.001), MMVD stage B1 (NLR: 3.15 [2.15–3.86], *P* < 0.001; MLR: 0.26 [0.20–0.36], *P* < 0.001), and MMVD stage B2 dogs (NLR: 3.22 [2.45–3.85], *P* < 0.001; MLR: 0.30 [0.19–0.37], *P* < 0.001). The area under the receiver operating characteristic curves of the NLR and MLR to distinguish dogs with MMVD C and D from those with MMVD B were 0.84 and 0.89, respectively. The optimal cutoff value for NLR was 4.296 (sensitivity, 68%; specificity, 83.95%), and the MLR value was 0.322 (sensitivity, 96%; specificity, 66.67%).

**Conclusions and Clinical Importance:** NLR and MLR can be used as adjunctive indicators of CHF in dogs.

**Table:** **Table 1.** Demographic, physical examination, radiographic, echocardiographic, and hematologic data for 128 dogs.

	Healthy	Stage B1 MMVD	Stage B2 MMVD	Stage C, D MMVD	*P* value
Number of dogs	22	42	39	25	—
Sex (m/f)	15/7	23/19	23/16	13/12	0.683
Age (years)	10 (8.50–12.00)	10 (8.52–12.81)	11 (9.00–13.17)	11.17 (9.92–14.04)	0.116
Weight (kg)	4.08 (2.82–5.31)	4.26 (3.25–5.42)	4.8 (3.42–6.12)	3.98 (2.88–5.72)	0.576
Heart rate (per minute)	135 (120–151.5)	144 (126–164.5)	156 (143–180)	168 (147–185)^a,b^	<0.001
Respiratory rate (per minute)	36 (30–42)	36 (30–60)	30 (24–60)	60 (45–80)^a,b,c^	<0.001
Systolic blood pressure (mm Hg)	142.5 (128.8–152.8)	140 (128.5–152.5)	132 (125.5–150)	120 (110–147.5)	0.041
Murmur grade	0 (0–0)	3 (3–4)^a^	4 (4–4)^a,b^	4 (4–5)^a,b^	<0.001
VHS	10.27 ± 0.72	10.41 ± 0.88	11.24 ± 0.92^a,b^	11.93 ± 1.22^a,b,c^	<0.001
LA:Ao ratio	—	1.56 (1.44–1.73)	1.96 (1.78–2.5)^b^	2.3 (2.00–2.56)^b^	<0.001
LVIDdN	—	1.43 ± 0.18	1.82 ± 0.25^b^	1.74 ± 0.31^b^	<0.001
NLR	3.05 (1.82–3.37)	3.15 (2.15–3.86)	3.22 (2.45–3.85)	4.99 (3.69–7.29)^a,b,c^	<0.001
MLR	0.21 (0.14–0.32)	0.26 (0.20–0.36)	0.30 (0.19–0.37)	0.56 (0.36–0.74)^a,b,c^	<0.001
PLR	200.1 (135.6–242.4)	208.0 (154.4–284.6)	200.8 (133.7–270.3)	246.5 (165.5–333.4)	0.341

Ao, aorta; LA, left atrium; LVIDdN, left ventricular end‐diastolic diameter normalized for body weight; MLR, monocyte‐to‐lymphocyte ratio; MMVD, myxomatous mitral valve disease; NLR, neutrophil‐to‐lymphocyte ratio; PLR, platelet‐to‐lymphocyte ratio; VHS, vertebral heart score. The mean ± standard deviation was used to describe normally distributed data, and the median (interquartile range) was used to represent non‐normally distributed data.

^a^ *P* < 0.05 vs. healthy

^b^ *P* < 0.05 vs. stage B1 MMVD

^c^ *P* < 0.05 vs. stage B2 MMVD

## Abstract C42: Diagnostic Value of Pulmonary Vein to Pulmonary Artery Ratio Measurement in Dogs with MMVD

### **Jiyoung Kim**; Chul Park

#### Animal Medical Center, Jeonbuk National University, Jeonju, Republic of Korea

**Background:** According to a study conducted in the United States, myxomatous mitral valve disease (MMVD) is the most common acquired cardiovascular disease in dogs. It can be suspected by cardiac murmur or chest radiography, and the golden standard for diagnosis is evaluation of cardiac morphologic changes by echocardiography.

**Hypothesis/Objectives:** To identify the diagnostic value of pulmonary vein to pulmonary artery ratio (PA/PV) measurement with echocardiography for dogs diagnosed as MMVD.

**Animals:** A total of 75 client‐owned dogs who visited Jeonbuk National University Animal Medical Center from October 2020 and October 2022 were retrospectively studied.

It was approved by IACUC.

**Methods:** The PA/PV was measured using bi‐dimensional mode in the right parasternal long axis four chamber view.

**Results:** The PV/PA in each group: control (1.08 ± 0.22), stage B1 (1.32 ± 0.30), stage B2 (2.06 ± 0.72), stage C (2.94 ± 1.01). Using One‐way ANOVA analysis, it was confirmed that the PV/PA increased significantly as the MMVD stage progressed (*P* < 0.001). Additionally, PV/PA was higher in the congested heart failure (CHF) group when compared with the no‐CHF group (*P* < 0.001).

**Conclusion and Clinical Importance:** In conclusion, as the severity of MMVD increased, PV/PA significantly increased, and it was confirmed that there was a positive correlation with conventional indicators. The PV/PA measurement has advantages of being easy and simple to evaluate compared to existing echocardiography indices. However, additional studies with various variables controlled are required, and more data should be accumulated to support the reliability of the study.

## Abstract C43: Comparison of the Clinical Utility of Del‐Nido Cardioplegia and St. Thomas Solution With Cardiac Troponin‐I

### **Kentaro Kurogochi**; Ayaka Chen; Yasuyuki Nii; Masako Nishiyama; Hiroshi Sugiya; Arane Takahashi; Masami Uechi

#### JASMINE Veterinary Cardiovascular Medical Center

**Background:** Myocardial protection techniques in open‐heart surgery have yet to be well‐researched and standardized in veterinary medicine. Recently, del‐Nido cardioplegia has been reported as a new technique in humans.

**Hypothesis/Objectives:** This prospective study aimed to compare the utility of del‐Nido cardioplegia with the conventional technique for mitral valve repair (MVR) in dogs and assessed myocardial damage using cardiac troponin‐I (cTnI).

**Animals:** Forty client‐owned dogs eligible for MVR were randomly divided into Group C (conventional technique) and Group D (del‐Nido). Consent for the study was obtained from owners beforehand.

**Methods:** Group C used St. Thomas solution with 50% blood and potassium (40–50 mEq/L) every 10 min. Group D used the del‐Nido cardioplegic technique, administered once or only when required, containing 20% blood, lidocaine (125 mg/L), and potassium (30 mEq/L). The levels of cTnI were assessed four times (before surgery and 2, 12, and 24 h after surgery).

**Results:** There were no differences between the two groups regarding operative time, cardiopulmonary bypass time, or aortic cross‐clamping time during the procedure. However, the time from declamping to the return of sinus rhythm was shorter in Group D. There were no significant differences in cTnI levels between the groups at any time.

**Conclusions and Clinical Importance:** In conclusion, both techniques in this study facilitated the safe performance of MVR: the lack of a difference in cTnI levels after surgery indicates that postoperative myocardial damage was comparable. However, the patients with del‐Nido cardioplegia had a smoother resumption of sinus rhythm following surgery.

## Abstract C44: A Novel Nonsynonymous Gene Variant in *IDUA* in Basset Hounds With Mucopolysaccharidosis Type 1

### **Elizabeth A. Martin** ^1^; Gary Johnson^2^; Garrett Bullock^3^; Gayle Johnson^4^; Stacey Leach^5^

#### ^1^College of Veterinary Medicine, University of Missouri, Columbia, MO, USA; ^2^Associate Professor, Veterinary Pathobiology, College of Veterinary Medicine, University of Missouri, Columbia, MO, USA; ^3^Graduate, Veterinary Pathobiology, College of Veterinary Medicine, University of Missouri, Columbia, MO, USA; ^4^Adjunct Professor, Veterinary Diagnostics Laboratory, College of Veterinary Medicine, University of Missouri, Columbia, MO, USA; ^5^Associate Professor, Cardiology, College of Veterinary Medicine, University of Missouri, Columbia, MO, USA

**Background:** Mucopolysaccharidoses (MPS) are a collection of heritable lysosomal storage disorders that result in multi‐systemic dysfunction including cardiac disease. The most severe form (MPS1 or Hurler's syndrome) is due to deficiencies in the alpha‐L‐iduronidase enzyme.

**Hypothesis/Objectives:** To describe the clinical cardiac features in a family of Basset hounds and to identify the causal mutation.

**Animals:** Three related Basset hounds with suspected MPS and DNA samples from 740 individual Basset hounds.

**Methods:** Echocardiography, electrocardiography, and post‐mortem evaluations were performed in the clinically affected dogs. Whole genome sequencing was performed from a related dog with compatible clinical signs and a positive toluidine urine spot test for MPS. DNA samples from both related and unrelated Basset hounds were genotyped for the likely causal sequence variant with an allelic discrimination assay.

**Results:** Affected dogs had varying degrees of mitral, tricuspid, and aortic valvular thickening and regurgitation as well as aortic root dilation. No arrhythmias or conduction disturbances were noted. Histopathology confirmed excessive glycosaminoglycan accumulation within the cardiac valves. Whole genome sequencing revealed a rare, but likely causal homozygous glycine‐to‐serine missense mutation in *IDUA*. To date, genotyping has revealed 13 DNA samples homozygous for the variant allele (including the clinically affected dogs), 50 were heterozygous, and 677 tested homozygous for the reference allele. The variant allele frequency was 5.6% for the genotyped cohort.

**Conclusions and Clinical Importance:** A novel *IDUA* variant is associated with the development of classical cardiac signs of MPS1 in Basset hounds and genetic testing may help guide breeding decisions.

## Abstract C45: Retrospective Analyze of Bosentan Treatment in Dogs With Severe Pulmonary Hypertension

### Dmitrii Oleynikov

#### Belij Klyk

Pulmonary hypertension is a complex syndrome that affects pulmonary circulation and the right heart. This syndrome progression is a prognosis‐worsening factor and is associated with decreased survival and low quality of living. Conventional treatment includes phosphodiesterase 5 inhibitors (sildenafil or tadalafil) and combined drugs such as pimobendan (phosphodiesterase 3 inhibitor). In the terminal stages, this treatment could be insufficient and cannot control all the symptoms.

In this study, we retrospectively analyzed patients with severe pulmonary hypertension and additional treatment with bosentan (endothelin‐1 inhibitor) which is believed to improve prognosis and outcome in diseased dogs.

We studied 23 client‐owned dogs with refractory pulmonary hypertension of different etiology, already treated with sildenafil and with symptoms of dyspnea, syncope, ascites, and weakness. The diagnosis was based on the EchoCG studies and disease history analysis, according to ACVIM consensus statements in pulmonary hypertension. In order to determine the efficacy of bosentan, based on its extended impact and laboratory data in animals, we divided patients into two groups: 1—dogs treated for less than two weeks (*n =* 9), 2—dogs treated for a longer period (*n =* 14).

**Results:** The 2nd group had significant improvements in ascites (9/14), dyspnea (14/14), syncope (13/14), activity (10/14). Median survival time in the 1st group was 5 days, in the 2nd—5 months. EchoCG showed a positive trend in TAPSE, pulmonary vein to right pulmonary artery ratio, and right pulmonary artery distensibility index, with a decrease in RV diameter and tricuspid regurgitation velocity after two weeks of treatment.

**Conclusion:** These findings show significant improvement in clinical and EchoCG parameters after bosentan treatment compared to previous sildenafil medication.

## Abstract C46: Vascular Responsiveness to Pimobendan as Assessed by Doppler Ultrasonography in Dogs With Congestive Heart Failure

### **Paulo A. Rivera** ^1^; Michael Aherne^2^, MVB (Hons 1), GradDipVetStud, MS, MANZCVS (Small Animal Surgery), DACVIM (Cardiology); John Bonagura^3^, DVM, MS, DACVIM (Cardiology, Internal Medicine)

#### ^1^Small Animal Hospital, University of Florida, Gainesville, FL, USA; ^2^Clinical Assistant Professor, Cardiology, Department of Small Animal Clinical Sciences, College of Veterinary Medicine, University of Florida, Gainesville, FL, USA; ^3^Adjunct Professor, Department of Clinical Sciences, College of Veterinary Medicine, North Carolina State University, Raleigh, NC, USA

**Background:** The relative importance of vascular responses to pimobendan in dogs with congestive heart failure (CHF) are incompletely studied.

**Hypothesis/Objectives:** We hypothesized that if pimobendan reduces systemic vascular resistance, femoral arterial flow (estimated by Doppler velocity‐time integral) will increase and arterial resistive index will decrease after a single dose of pimobendan in dogs with CHF.

**Animals:** Ten client‐owned dogs with myxomatous mitral valve degeneration undergoing treatment for acute onset of CHF were studied.

**Methods:** Prospective, within‐subjects study design. Duplex Doppler ultrasonography (DDU) was used to quantify femoral arterial volumetric flow and calculate resistive index at baseline and 2 h after a single oral dose of pimobendan (0.3 mg/kg). DDU recordings were measured offline by an operator blinded to treatment. Measurements pre‐and post‐pimobendan administration were compared using paired *t*‐tests or Wilcoxon's signed‐rank test.

**Results:** Mean dose (±SD) of furosemide at enrollment was 2.616 mg/kg (±1.56). Total VTI estimating flow over the cardiac cycle was unchanged from baseline to treatment (3.24 cm ± 1.82 vs. 3.24 cm ± 1.50). Resistive index at baseline (1.21 ± 0.16) was not different from pimobendan (1.30 ± 0.07, *P* = 0.105), although peak systolic velocity was 0.22 cm/s higher (*P* = 0.026) and minimal velocity 0.12 cm/s lower (*P* = 0.011) after pimobendan. Mean systolic blood pressure measurements were 126.5 mm Hg ± 20.13 and 131.0 mm Hg ± 19.97) pre‐ vs. post‐pimobendan (*P* = 0.568).

**Conclusion:** A single dose of pimobendan following administration of furosemide did not produce measurable vasodilation or increased peripheral blood flow based on DDU in dogs with acute congestive heart failure.

## Abstract C47: Machine Learning‐Based Risk Prediction for Canine Myxomatous Mitral Valve Disease Using Electronic Health Record Data

### **Yunji Kim**; Jihye Choi; Jaejin Kim; Hwayoung Youn; Sehoon Kim

#### Seoul National University, Seoul, Republic of Korea

**Background:** Myxomatous mitral valve disease (MMVD) is the most common cause of heart failure (HF) in dogs, and its risk assessment of HF is often challenging. Machine learning applied to electronic health records (EHRs) is an effective tool for predicting prognosis in the medical field.

**Objectives:** This study aimed to develop machine learning‐based HF risk prediction models for dogs with MMVD using a dataset of EHRs.

**Animals:** A total of 143 dogs with MMVD between May 2018 and May 2022.

**Methods:** Complete medical records were reviewed for all patients. Demographic data, radiographic measurements, echocardiographic values, and laboratory results were obtained from the clinical database. Four machine learning algorithms (Random Forest, K‐Nearest Neighbors, Naïve Bayes, Support Vector Machine) were used to develop the risk prediction models. Model performance was represented by plotting the receiver operating characteristic (ROC) curve and calculating the area under the curve (AUC). The best‐performing model was chosen for the feature ranking process.

**Results:** Random forest showed superior performance to the other models (AUC = 0.88), while the performance of the K‐nearest neighbors showed the lowest performance (AUC = 0.69). The top three models showed excellent performance (AUC ≥ 0.8). According to the random forest algorithm feature ranking, echocardiographic and radiographic variables had the highest predictive values for HF, followed by PCV and respiratory rates. Among the electrolyte variables, chloride had the highest predictive value for HF.

**Conclusions and Clinical Importance:** These machine‐learning models will support clinical decision‐making in estimating the prognosis of patients with MMVD.

## Abstract C48: Echocardiographic Findings and Prevalence of Heart Murmurs in Clinically Healthy Adult Borzoi Dogs

### **Tess Sykes** ^1^; Sonya Wesselowski^2^, DVM, MS, DACVIM (Cardiology); Ashley Saunders^3^, DVM, DACVIM (Cardiology)

#### ^1^Texas A&M University, College Station, TX, USA; ^2^Assistant Professor, Texas A&M University, College Station, TX, USA; ^3^Professor, Texas A&M University, College Station, TX, USA

**Background:** Prospective data reporting the prevalence of structural cardiac disease and heart murmurs in Borzoi dogs is lacking.

**objective:** To report echocardiographic findings and prevalence of heart murmurs in a cohort of healthy Borzoi.

**Animals:** 146 client‐owned, clinically healthy, Borzoi.

**Methods:** Single center prospective cohort study. Auscultation and standard echocardiograms were performed by board‐certified cardiologists. Longitudinal echocardiograms were performed in a subset.

**Results:** The population consisted of 86 females (58.9%) and 60 males (41.1%). Median age was 4 years (range 1.1–12.0). Heart murmurs were identified in 86/146 dogs (58.9%). Most Borzoi were structurally normal (119/146, 81.5%), with 64 normal dogs (43.8% of the total population) having an innocent heart murmur. Thirty‐six structurally normal dogs had trace or mild mitral regurgitation (30.2% of normal dogs) and 43 had trace or mild tricuspid regurgitation (36.1% of normal dogs). Structural cardiac disease was identified in 21 dogs (14.4%), with another six dogs having equivocal diagnoses (4.1%). Dilated cardiomyopathy (DCM) was diagnosed in nine dogs (6.2%) and stage B1 myxomatous mitral valve disease (MMVD) was diagnosed in nine dogs (6.2%). Twenty‐five dogs had longitudinal examinations. Two dogs developed occult DCM, two transitioned to an equivocal DCM diagnosis, and two developed stage B1 MMVD within two years. Two dogs with DCM (one occult, one equivocal) improved with diet change.

**Conclusions and Clinical Importance**: Structurally normal Borzoi commonly have trace or mild atrioventricular valve insufficiencies and innocent heart murmurs. All Borzoi with MMVD had normal heart size. Screening for DCM may be considered in the breed.

## Abstract E01: Effect of Single‐Dose Intravenous Furosemide on the Renin‐Angiotensin‐Aldosterone‐System of Healthy Horses

### **Mallory Lehman**; Marisa Ames, DVM, DACVIM‐Cardiology; Jessica Morgan, DVM, PhD, DACVSMR

#### University of California Davis, Davis, CA, USA

**Background:** Furosemide, a commonly used diuretic, activates the circulating renin‐angiotensin‐aldosterone system (RAAS) in other species. Little is known about RAAS activation in horses.

**Hypothesis/Objectives:** We hypothesize that furosemide will cause transient elevation in both classical and alternative RAAS hormones.

**Animals:** Adult thoroughbreds from a university teaching herd were used for the cross over study design (*n =* 6) and baseline hormone levels (*n =* 8).

**Methods:** Horses received either intravenous furosemide (1 mg/kg) or saline. Circulating RAAS hormones were quantified at 0, 0.25, 0.5, 4, and 24 h post administration, via liquid chromatography‐tandem mass spectrometry and/or equilibrium dialysis. Values were compared with a repeated measures mixed model. Paired protease inhibited samples were also analyzed and compared with Spearman's Correlation. Data are presented as median (baseline) or difference in mean (elevation), [95% CI].

**Results:** Only angiotensin‐II (7.4 [4.8–10.7] pMol/L) was consistently above the limit of quantification at baseline. Furosemide resulted in an increase in angiotensin I (8.0 [1.04–15.03] pMol/L, *P* = 0.02), angiotensin‐II (33.7 [10.0–57.4] pMol/L, *P* = 0.002), angiotensin‐IV (2.0 [0.64–3.39] pMol/L, *P* = 0.001), angiotensin 1–5 (5.6 [1.84–9.32] pMol/L, *P* = 0.0009), and aldosterone (180.8 [3.1–358.5] *P* = 0.04) compared to saline at 4 . Correlation between protease inhibition and equilibrium dialysis was high for Angiotensin‐I and Angiotensin II (*r =* 0.81 and 0.86, respectively; *P* < 0.0001).

**Conclusions and Clinical Importance:** Baseline levels of circulating RAAS hormones are low in healthy horses. Furosemide produced an increase in hormones associated with both the classical and alternative RAAS pathways. Comprehensive RAAS data offers a model to study pharmacologic and pathologic activation in horses.

## Abstract E02: A Smart Textile Device Can Accurately Capture Heart Rate Variability in Resting Horses: Preliminary Results

### **Persephone McCrae** ^1^; Hannah Spong^1^; Jordyn Moorehead^1^; Amin Mahnam^2^; Yana Bashura^2^; Wendy Pearson^1^

#### ^1^University of Guelph, Guelph, ON, Canada; ^2^Myant, Inc., Etobicoke, ON, Canada

**Background:** Smart textiles have been developed to replace standard adhesive electrodes for collection of electrocardiograms, though limited work has assessed their potential in equine medicine.

**Hypothesis/Objectives:** The objective of this study was to assess the validity of a smart textile ECG device for long‐duration heart rate variability (HRV) assessment.

**Animals:** Four healthy Warmblood horses.

**Methods:** ECGs were simultaneously recorded using a smart textile device and a standard telemetric device from unrestrained horses for 6 hrs in a controlled pilot study. Data were analyzed to calculate heart rate and HRV metrics [average RR intervals, the axial and perpendicular dispersions from the line of identity from the Poincaré plot (SD1 and SD2, respectively), root mean square of successive differences (RMSSD), percentage of successive RR interval differences (pNN50), and oscillations in the very‐low frequency (VLF), low‐frequency (LF), and high‐frequency (HF) bands]. Data were compared using Bland‐Altman analysis and Pearson coefficients.

**Results:** Excellent agreement with small biases were observed between the two devices and perfect correlations (*r =* 1.0, *P* < 0.0001) were found for all metrics except for VLF and HF. Bland‐Altman tests revealed a mean bias of 0.003 bpm (95% limits of agreement: −0.06 to 0.06) for HR, −0.05 ms (−2.62 to 2.52) for RR intervals, −0.30 ms (−3.60 to 3.00) and −1.10 ms (−5.49 to 3.29) for SD1 and SD2, respectively, and −0.43 ms (−5.09 to 4.23) for RMSSD.

**Conclusions and Clinical Importance:** This study demonstrates that a smart textile device is a practical alternative for long‐duration assessment of HRV in resting horses.

## Abstract E03: Histopathological Evaluation of Myocardial Hypertrophy and Fibrosis in Thoroughbred Racehorses

### **Laura C. Nath** ^1^; Arnela Saljic^2^, PhD; Rikke Buhl^2^; Kirsten Lundgren Virklund^2^; Helena Schmidt Royal^2^; Charles Ye^2^; Adrian Elliott^1^; Andre La Gerche^3^; Thomas Agbaendeng^1^; Andrew Stent^4^; Samantha Franklin^1^

#### ^1^University of Adelaide; ^2^University of Copenhagen, Copenhagen, Denmark; ^3^Baker Heart and Diabetes Institute, Melbourne, VIC, Australia; ^4^University of Melbourne, Melbourne, VIC, Australia

**Background:** Athletic horses have a high prevalence of cardiac arrhythmia in the absence of structural heart disease. The underlying triggers and substrate for arrhythmia in this species are not well studied.

**Hypothesis:** Thoroughbred racehorses will have myocardial fibrosis, increased myocyte diameter, and a greater density of fibroblasts compared to untrained horses.

**Animals:** Thoroughbred racehorses, comprising 15 that died from sudden cardiac death (SCD) and 18 that died from other fatal injuries (OFI), were compared to 10 healthy untrained (UT) wild horses.

**Methods:** Cardiac tissues were sampled during post‐mortem. OFI and UT were age matched to the SCD group. Two atrial and three ventricular sites were stained with Sirius red for assessment of myocardial fibrosis and triple antibody stain (WGA, vimentin, and GS‐IB4) for myocyte diameter and fibroblast density. Measurements were made by a blinded operator using automated analysis with commercial software. Results were tested for normality using the Shapiro‐Wilk test and differences assessed with one‐way ANOVA with multiple comparisons.

**Results:** SCD had a greater fibrosis proportion compared to UT at 4/5 examined sites. UT had reduced myocyte diameter at 5/5 sites compared to SCD or OFI. There were no significant differences between SCD and OFI for either fibrosis proportion or myocyte diameter and there were no differences between any group for fibroblast density.

**Conclusions and Clinical Importance:** Thoroughbred racehorses have histological myocardial hypertrophy. Myocardial fibrosis is pronounced in SCD, and this could be a substrate for arrhythmia. The absence of increased fibroblast density might suggest underlying chronic fibrotic remodeling.

**Table:** Table 1. Proportion of fibrosis, myocyte diameter and fibroblast density at each site for each of the groups studied.

Analysis and site	SCD *n =* 15, (Mean ± SD)	OFI *n =* 18, (Mean ± SD)	UT *n =* 10, (Mean ± SD)	One‐way ANOVA *P* value
Fibrosis (%)				
LAA	34 ± 8.7^a^	31 ± 5.7^ab^	25 ± 4.9^b^	0.01
RAA	38 ± 6.1^a^	33 ± 7.8^ab^	29 ± 7.1^b^	0.01
LVAPM	12 ± 3.5	12 ± 3.1	8.9 ± 0.94	0.07
LVPPM	16 ± 7.0^a^	12 ± 3.2^ab^	6.4 ± 0.92^b^	0.03
IVS	20 ± 3.7^a^	16 ± 6.1^a^	8.7 ± 3.6^b^	<0.0001
Myocyte diameter (μm)				
LAA	14 ± 2.1^a^	14 ± 1.3^a^	12 ± 1.5^b^	0.004
RAA	14 ± 1.6^a^	14 ± 1.3^ab^	13 ± 1.3^b^	0.03
LVAPM	15 ± 1.7^a^	16 ± 1.6^a^	13 ± 0.99^b^	0.009
LVPPM	15 ± 1.9^ab^	16 ± 1.3^a^	14 ± 1.0^b^	0.01
IVS	13 ± 1.9^ab^	14 ± 2.2^a^	12 ± 0.94^b^	0.02
Fibroblast density (fibroblast/mm^2^)				
LAA	1266 ± 597	1558 ± 933	1016 ± 284	0.18
RAA	1111 ± 706	1027 ± 629	1146 ± 316	0.87
LVAPM	954 ± 730	954 ± ± 730	563 ± 694	0.40
LVPPM	917 ± 838	862 ± 762	469 ± 491	0.42
IVS	2081 ± 1123	2536 ± 1309	1486 ± 886	0.09

Abbreviation: IVS, interventricular septum; LAA, left atrial appendage; LVAPM, left ventricular anterior papillary muscle; LVPPM, left ventricular posterior papillary muscle; and RAA, right atrial appendage. Variables with different superscripts are significantly different between groups, p <0.05.

## Abstract E04: Exercising Electrocardiograms in Eventing Horses in the Field

### **Cris Navas de Solis** ^1^; Darko Stefanovski^1^; Mary Durando^2^

#### ^1^University of Pennsylvania, Philadelphia, PA, USA; ^2^Sports Medicine Practice

**Background:** Exercising arrhythmias can cause poor performance or sudden death.

**Hypothesis/Objectives:** To describe the association of exercising arrhythmias in field recordings with type, intensity and duration of exercise and presence of previous cardiac signs.

**Animals:** Cohort study of 36 Eventing horses in active competition.

**Methods:** 571 recordings [median (IQR), 7(4–12) ECGs/horse] were evaluated and 167 were <95% readable and discarded. Mixed‐effects logistic regression tested the association of the dichotomous outcomes presence of arrhythmia (arrhythmia) and presence of complex arrhythmia (arrhyhmiacomplex). Duration of recording (mins) (duration), average heart rate (beats/min) (HRaverage) and peak heart rate (beats/min) (HRpeak) were set as the fixed effects. Analysis was confounded by exercise type and prior cardiac signs (horses enrolled after referral for cardiac evaluation) with horse ID as the random effect. Mixed‐effects Poisson regression analyzed number of premature complexes (nPC) analogously.

**Results:** There was arrhythmia in 33.2% of the recordings, arrhyhmiacomplex in 5.7% and the n°PC was 0(0–1) [range 0–99 PC/ECG]. 32.7% of the recordings were gallops, 23.8% flat sessions, 16.3% hacks, 15.1% jumping sessions, 5.2% cross‐county schooling sessions, 4.7% trotting sessions and 2.2% cross‐country competition. HRpeak was (mean ± SD) 169 ± 35 and HRaverage 94 ± 19. Increased likelihood of arrhythmia was associated with duration (OR *=* 1.022, CI [1.004, 1.041], *P* = 0.017) and HRpeak (OR *=* 1.037, CI [1.019, 1.055], *P* < 0.001. Risk of arrhyhmiacomplex was increased with HRpeak (OR *=* 1.040, CI [1.017, 1.064], *P* = 0.001) and cardiac signs (OR *=* 6.157, CI [1.390, 27.270], *P* = 0.017). Increased n°PC was associated with duration (IRR *=* 1.013, CI [1.009, 1.017], *P* = <0.001), HRaverage (IRR *=* 1.033, CI [1.027, 1.041], *P* < 0.001) and hacks (IRR *=* 2.44, CI [1.079, 5.539], *P* = 0.0320). None of the horses developed cardiac signs during the study.

**Conclusions and Clinical Importance:** Intensity, duration, type of exercise and previous cardiac signs are relevant when evaluating exercising ECGs in Eventers.

## Abstract E05: Association of DFA Alpha 1 With Blood Lactate Measurements in Exercise Tests in Sport Horses

### **Kaitlin Murphy**; Claire Solomon; Olivia Lorello; Darko Stefanovski; Joanne Haughan; Mary Robinson; Cristobal Navas De Solis

#### University of Pennsylvania

**Background:** Methods to determine thresholds that separate different exercise intensities often require equipment and the involvement of a veterinarian. Detrended fluctuation analysis (DFA) is a heart rate variability metric that quantifies the degree of correlation within a heart rate series and has been proposed as a non‐invasive way of assessing cardiac parasympathetic activity during exercise. The main advantage of DFA is the potential to provide real time or retrospective information under field conditions using rider friendly wearable monitors and its potential association with exercise intensity thresholds and fatigue.

**Objectives:** The objective of this study was to 1—describe the behavior of the first component of DFA (DFA‐α1) during standardized field incremental exercise tests. 2—determine if DFA‐α1 is associated with lactate concentrations in this setting. We hypothesized that DFA‐α1 would have a high correlation with lactate measurements and that this association would be stronger than the association with heart rate and speed.

**Animals:** 33 competitive Eventing horses and sport horses without competitive goals.

**Methods:** *In vivo* observational study that analyzed exercising electrocardiograms obtained in 60 standardized exercise tests in the field.

**Results:** DFA‐α1 was significantly associated with lactate levels (*ρ* = −0.49, *P* < 0.001) but the association was weak. A stronger association was observed between lactate and speed (*ρ* = −0.80, *P* < 0.001), as well as lactate and heart rate (*ρ* = 0.74, *P* < 0.001).

**Conclusions:** DFA‐α1 association with lactate does not support its use as a surrogate of lactate in field incremental standardized tests used for sport horses.

## Abstract E06: Evaluation of the Audicor Device As Diagnostic Tool in Horses With Mitral or Aortic Regurgitation

### **Isabelle Piotrowski** ^1^; Colin Schwarzwald^2^, Professor, DrMedVet, PhD, DACVIM, DECEIM

#### ^1^Vetsuisse Faculty, University of Zurich, Zurich, Switzerland; ^2^Director, Clinic for Equine Internal Medicine, Clinic for Equine Internal Medicine, Vetsuisse Faculty, University of Zurich, Zurich, Switzerland

**Background:** Cardiac murmurs caused by mitral (MR) or aortic regurgitation (AR) in horses often prompt further diagnostics to assess disease severity and prognosis.

**Hypothesis/Objectives:** This study aimed to examine the use of Audicor Acoustic Cardiography to detect murmurs and quantify cardiac mechanical and hemodynamic function in horses with mild, moderate, or severe MR or AR.

**Animals:** 30 horses with MR, 18 with AR, 18 healthy horses.

**Methods:** Prospective study. The following variables were extracted from five consecutive, subsequently averaged 10‐s snapshot analyses: heart rate (HR, min^−1^), electromechanical activation time (EMAT, ms), HR‐corrected EMAT (EMATc, %), left ventricular systolic time (LVST, ms), HR‐corrected LVST (LVSTc, %), strength of third (S3) and fourth (S4) heart sound, and systolic dysfunction index (SDI, a function of QRS duration, QT interval, EMATc, and S3). Measurements were compared between groups using one‐way ANOVA.

**Results:** Horses with AR were 6 (2–10) years [mean difference (95% CI of the difference of means)] older than horses with MR and 7 (2–12) years older than healthy horses. The LVST was 33 (5–60) ms shorter and the EMAT/LVST ratio 0.038 (0.001–0.075) higher in horses with MR compared to AR. Body weight, HR, EMAT, EMATc, LVSTc, strength of S3 and S4, and SDI were not significantly different between groups (*P* > 0.05).

**Conclusions and Clinical Importance:** Acoustic cardiography can detect differences in some systolic time intervals in horses with MR vs. AR. The clinical relevance as diagnostic point‐of‐care device in horses with MR or AR of various severity needs to be determined.

## Abstract E07: Echocardiographic Measurements and Findings in Healthy Standardbred and Thoroughbred Racehorses

### **Freya Stein** ^1^; Christopher Stauthammer^1^, DVM, DACVIM (Cardiology); Anna Rice^2^; Molly McCue^3^, DVM, MS, PhD, DACVIM (LAIM); Sian Durward‐Akhurst^4^, BVMS, MS, PhD, MRCVS, DACVIM (LAIM)

#### ^1^Equine Genetics and Genomics Lab, University of Minnesota, Minneapolis, MN, USA; ^2^University of Minnesota, Minneapolis, MN, USA; ^3^Associate Dean for Research and Professor of Equine Internal Medicine, University of Minnesota, Minneapolis, MN, USA; ^4^Assistant Professor of Genetics, Genomics, and Large Animal Internal Medicine, Veterinary Clinical Sciences, University of Minnesota, Minneapolis, MN, USA

**Background:** Echocardiography is a mainstay of cardiac evaluations. Numerous reference ranges have been published for different horse breeds, however, many were published >10 years ago. We are currently performing cardiac evaluations, including echocardiography, on 1,000 healthy Standardbred and Thoroughbred racehorses. In evaluating echocardiographic measurements, interobserver variability can be a significant source of data inaccuracy.

**Objectives:** Our primary objective is to identify structural cardiac abnormalities in and update reference ranges for healthy racehorses. Our secondary objective is to determine interobserver variability.

**Animals:** Echocardiography was performed on Thoroughbreds (*n =* 31) and Standardbreds (*n =* 53), comprised of 51 geldings, 24 mares, 4 stallions, and 5 undesignated, aged 2–10 years old.

**Methods:** This was a non‐randomized observational case‐control study. Echocardiography was performed on non‐sedated horses. Standard measurements were obtained.

**Results:** Mean (standard deviation) values for Thoroughbreds and Standardbreds respectively were obtained: ejection fraction (64.37%, 4.92; 64.0%, 4.18), fractional shortening (37.35%, 3.82; 37.07%, 3.24), left atrial diameter (12.22 cm, 0.72; 12.42 cm, 0.77), interventricular septum (2.61 cm, 0.25; 2.65 cm, 0.33), diastolic left ventricular diameter (11.08 cm, 1.18; 11.25 cm, 1.08), left ventricular free wall (2.58 cm, 0.23; 2.32 cm, 0.25) and systolic left ventricular diameter (6.96 cm, 0.88; 7.08 cm, 0.76). No horses had clinically relevant structural disease.

**Conclusions and Clinical Importance:** These measurements will provide updated reference ranges for healthy racehorses and information about the proportion of horses with structural cardiac disease. Interobserver agreement will be evaluated for three observers.

## Abstract E08: *In Silico* Simulations for Efficacy and Risk of Transvenous Electrical Cardioversion Catheter Positions in Horses

### **Glenn Van Steenkiste** ^1^; Annelies Decloedt^2^, DVM, PhD; Gunther van Loon^2^, DVM, DACVIM, DECEIM, ECVDI; Ingrid Vernemmen^3^, DVM

#### ^1^Ghent University, Ghent, Belgium; ^2^Professor, Department of Internal Medicine, Reproduction and Population Medicine, Ghent University, Ghent, Belgium; ^3^Department of Internal Medicine, Reproduction and Population Medicine, Ghent University, Ghent, Belgium

**Background:** The most efficient treatment option for atrial fibrillation (AF) in horses is transvenous electrical cardioversion (TVEC) but the position of the electrodes during TVEC has been empirically determined.

**Objective:** To rationalize electrode positioning in horses during TVEC procedures by using finite element models (FEMs) to compare electric fields using different electrode configurations.

**Animals:** Scaled 3D heart model based on a CT scan of a healthy pony.

**Methods:** The FEMs were created by segmenting a CT scan into tissue compartments, followed by meshing and assigning tissue conductivities. The FEMs were modified during segmentation by adding 9 Fr 10 cm long TVEC catheter electrodes in different configurations during the segmentation stage. The electrical fields of different configurations with the positive and negative electrodes were calculated at different energy levels typically used by truncated biphasic exponential waveform defibrillators, as described in table 1. The relative efficacy and safety were calculated by determining the amount of atrial myocardium above the defibrillation threshold (5 V/cm) and at risk for potential tissue damage (>30 V/cm).

**Table:** Table 1. Efficacy and safety parameters of different electrode configurations to achieve atrial cardioversion in horses. If multiple locations are specified per polarity, then the electrodes were interconnected.

Configuration		Applied energy	Atrial myocardium above cardioversion threshold (>5 V/cm)	Atrial myocardium above safety threshold (>30 V/cm)
Negative electrode	Positive electrode			
RA	Left PA	100 J	95.7%	20.4%
		150 J	98.5%	32.2%
		200 J	99.5%	41.6%
		250 J	99.8%	49.0%
		300 J	99.9%	54.7%
		360 J	99.9%	59.7%
Cranial vena cava	Left PA	150 J	95.2%	33.8%
Caudal vena cava	Pulmonary trunk	150 J	99.7%	36.8%
Caudal and cranial vena cava	Left PA	150 J	99.2%	36.8%
RA	Coronary sinus	150 J	99.9%	40.6%
RA	Right pulmonary artery	150 J	98.0%	43.5%
RA	Left pulmonary artery proximal of branches	150 J	98.5%	35.5%
Caudal and cranial vena cava	Left PA and pulmonary trunk	150 J	97.4%	45.6%

Abbreviations: RA: between tricuspid valve and terminal crest in the middle of the right atrium; left PA: In the first descending branch of the left pulmonary artery

**Results:** Increasing the energy above 200 J only yields marginal improvements in defibrillation efficacy. Defibrillation between the middle of the right atrium and the coronary sinus resulted in the highest efficacy. Detailed results are given in table 1.

**Conclusions and Clinical Importance:** Using an *in silico* model improves our understanding of atrial fibrillation cardioversion in horses and may contribute to the development of alternative cardioversion techniques. It may be more beneficial to change the position of the catheters rather than increase the energy.

## Abstract E09: Transseptal Puncture in the Horse Using Intracardiac Ultrasound Guidance: First Results With Follow‐Up

### **Ingrid A.L. Vernemmen** ^1^; Glenn Van Steenkiste^2^, DVM, PhD; Eva Buschmann^2^, DVM; Stijn Schauvliege^3^, DVM, PhD; Kristoff Cornelis^4^, MD; Annelies Decloedt^1^, DVM, PhD; Gunther van Loon^2^, DVM, PhD

#### ^1^Ghent University; ^2^Department of Internal Medicine, Reproduction and Population Medicine, Ghent University; ^3^Department of Surgery and Anaesthesiology of Domestic Animals, Ghent University; ^4^Cardiology Department, AZ Maria Middelares

**Background:** Radiofrequency catheter ablation has been successfully applied to treat right atrial tachycardia in horses. Ablation of left‐sided arrhythmias is complicated by the perilous retrograde arterial approach needed for left‐sided catheterization. In human medicine, the left atrium is accessed femorally through transseptal puncture (TSP) of the fossa ovalis (FO). Recently a TSP technique via the jugular vein was developed for horses.

**Hypothesis/Objective:** To determine efficacy, complications and closure process of TSP in horses.

**Animals:** Three horses without cardiovascular disease, and two horses admitted for ablation of atrial fibrillation (AF).

**Methods:** Descriptive experimental study. TSP was performed under general anaesthesia by radiofrequency energy application on a guidewire to perforate the FO and allow advancement of a deflectable sheath under transthoracic and intracardiac echocardiographic (TTE and ICE) guidance. In three horses without cardiovascular disease, puncture closure was evaluated during four weeks by TTE and ICE.

**Results:** All TSPs were successful. Time from sheath positioning in front of the FO until successful transseptal access was 19–97 min. In two horses, balloon dilation of the puncture opening was needed for sheath advancement. Minor complications included transient atrial premature depolarisations (*n =* 1), self‐terminating atrial tachycardia (*n =* 1) and self‐terminating AF (*n =* 1) during radiofrequency energy application for puncture in the horses without pre‐existing AF. Follow‐up of the closure process (*n =* 3) using ICE showed closure 1–3 weeks after puncture.

**Conclusions and Clinical Importance:** TSP could be performed safely in horses using ICE guidance, which opens perspectives for management of left‐sided atrial arrhythmias.

## Abstract E10: Investigation of Plasma Leptin Concentrations in Association with Obesity and Laminitis Status in Ponies

### **Brianna L. Clark** ^1^; Sarah Vaughn^2^; Allison Stewart^1^; Kate Kemp^1^; Nicholas Bamford^3^; Kelsey Hart^2^; Francois‐Rene Bertin^1^

#### ^1^The University of Queensland, St Lucia, QLD, Australia; ^2^University of Georgia, Athens, GA, USA; ^3^The University of Melbourne, Parkville, VIC, Australia

**Background:** Leptin is an adipokine released from adipose tissue. There are inconsistent reports of associations between increased leptin concentrations with obesity and laminitis status in ponies.

**Hypothesis/Objectives:** Increased plasma leptin concentrations will be associated with obesity and laminitis in ponies.

**Animals:** Cross‐sectional study of 211 Shetland and Welsh ponies sampled in Australia and the USA in autumn and/or spring.

**Methods:** Obesity was defined as a modified‐Henneke body condition score >6/9. Laminitis was defined as a modified‐Obel score >1/12. Plasma leptin concentrations were measured in all ponies by radioimmunoassay at Cornell University. Descriptive and interferential statistics were performed, with significance accepted at *P* < 0.05.

**Results:** Australian Welsh ponies had lower spring plasma leptin concentrations than American Welsh ponies (*P* = 0.0006). Compared to spring values, American Welsh ponies had lower autumnal plasma leptin concentrations (*P* = 0.01) while Australian Shetlands had higher autumnal plasma leptin concentrations (*P* = 0.004). Australian Welsh ponies had lower plasma leptin concentrations than Australian Shetlands in autumn (*P* = 0.009). Obese ponies (*n =* 122) had higher plasma leptin concentrations than non‐obese ponies (*n =* 136, *P* < 0.0001). No difference in plasma leptin concentrations between laminitic (*n =* 50) and non‐laminitic ponies (*n =* 120) were detected (*P* = 0.73).

**Conclusions and Clinical Importance:** Plasma leptin concentration is associated with obesity in ponies but varied with country, season, and breed, warranting careful interpretation of results. The clinical utility of measuring plasma leptin concentration in the diagnosis of laminitis remains to be determined.

## Abstract E11: Altered Insulin and Glucose Dynamics in Horses With Glucocorticoid‐Induced Laminitis

### **Erin F. Pinnell** ^1^; Mauria Watts^2^; Laura Hostnik^3^, DVM, MS, DACVIM; Kathryn Timko^2^, DVM, MSc, ACVIM‐LA; Allison Thriffiley^2^, DVM; Mercedes Stover^2^; Lauren Koenig^2^; Olivia Gorman^2^; Ramiro Toribio^2^, DVM, MS, PhD, DACVIM; Teresa Burns^2^, DVM, MS, PhD, DACVIM

#### ^1^College of Veterinary Medicine, The Ohio State University, Columbus, OH, USA; ^2^The Ohio State University, Columbus, OH, USA; ^3^Associate Professor, Clinical Equine Internal Medicine, The Ohio State University, Columbus, OH, USA

**Background:** Glucocorticoids are frequently administered to horses, and this treatment is associated with risk of hyperinsulinemia‐associated laminitis (HAL). Methods to predict development of treatment‐associated HAL are needed.

**Hypothesis/Objectives:** Evaluate insulin and glucose dynamics before and after administration of dexamethasone (0.08 mg/kg PO q 24 h; DEX) and compare results between horses that subsequently developed laminitis (LAM) and those that did not (NONLAM) after a 14‐day course of DEX administration. We hypothesized that insulin and glucose dynamics would differ between LAM and NONLAM horses.

**Animals:** 33 healthy adult horses were included.

**Methods:** Frequently‐sampled insulin‐modified IV glucose tolerance tests (FSIGTT) and oral sugar tests (OST) were performed at baseline and after 7 days of treatment with DEX. Minimal Model analysis was performed using FSIGTT data, and areas under the curve (AUC) of [insulin] (AUCins) and [glucose] (AUCg) were calculated from OST data. Paired Student's t‐tests were used to compare LAM and NONLAM groups at baseline and after DEX.

**Results:** 5/33 horses (15.15%) developed clinical signs of laminitis during or shortly after the 14‐day study period. AIRg (acute insulin response to glucose; *P* = 0.01) and AUCins (*P* = 0.002) were significantly higher in LAM versus NONLAM horses at baseline. Additionally, AUCins (*P* = 0.02) was significantly higher in LAM after DEX.

**Conclusions and Clinical Importance:** AIRg and AUCins calculated from the FSIGTT and OST, respectively, could predict development of HAL associated with glucocorticoid treatment and should be evaluated as biomarkers of this risk.

## Abstract E12: High‐Protein Meal Feeding is Associated With Post‐Prandial Hyperinsulinemia in Horses With Experimentally‐Induced Insulin Dysregulation

### **Allison A. Thriffiley**; Mauria Watts; Kathryn Timko; Erin Pinnell; Katelyn Keefer; Olivia Gorman; Laura Hostnik; Teresa Burns

#### College of Veterinary Medicine, The Ohio State University, Columbus, OH, USA

**Background:** Dietary management is the most important treatment for equine insulin dysregulation (ID) and often involves feeding low non‐structural carbohydrate (NSC), high‐protein ration balancers and grass hay. Data suggest that post‐prandial hyperinsulinemia can be exacerbated by a high‐protein meal in patients with ID, creating concern about the safety of this nutritional intervention.

**Hypothesis:** Consumption of a low‐NSC, high‐protein meal will induce post‐prandial hyperinsulinemia, which will be amplified after induction of ID in horses.

**Animals:** Seven adult, light‐breed horses with normal endogenous [ACTH].

**Methods:** Each horse underwent a frequently sampled insulin‐modified IV glucose tolerance test to characterize systemic insulin and glucose dynamics, followed by a feed challenge test (1 kg ration balancer [min 32% crude protein, max 13% NSC] consumed within 15 min, with [insulin] and [glucose] measured at baseline and every 30 min for 240 min after the meal). Both tests were repeated after induction of ID (dexamethasone 0.08 mg/kg PO once daily for 7 days). Outcomes were compared between baseline and ID conditions.

**Results:** ID was associated with exacerbation of post‐prandial hyperinsulinemia and hyperglycemia after high‐protein meal feeding. ID AUCins (548.9 ± 150 μIU/mL×min) was significantly higher than baseline AUCins (216.2 ± 104.4 μIU/mL×min; *P* = 0.003). ID AUCgluc (89.5 [60−115] mg/dL×min) was significantly higher than baseline AUCgluc (75 [34.9−94] mg/dL×min; *P* = 0.03).

**Conclusions and Clinical Importance:** Horses with experimentally‐induced ID displayed significantly higher glycemic and insulinemic responses to a high‐protein meal than at baseline, suggesting that high‐protein ration balancers may complicate nutritional management of equine ID.

## Abstract E13: Seasonal Fluctuations in Baseline Adrenocorticotrophic Hormone and Insulin Levels in Healthy Horses in Saskatchewan

### **Paula Viviani** ^1^; N. H. Chavarria^1^; V. Ragano^1^; M. Meachem^2^; Julia B. Montgomery^3^, Med Vet, PhD, DACVIM (LAIM)

#### ^1^Western College of Veterinary Medicine, University of Saskatchewan, SK, Canada; ^2^Prairie Diagnostic Services (PDS) Inc. and Western College of Veterinary Medicine, University of Saskatchewan, SK, Canada; ^3^Associate Professor, Large Animal Clinical Sciences, Western College of Veterinary Medicine, University of Saskatchewan, SK, Canada

**Background:** Early diagnosis of equine metabolic syndrome (EMS) and pituitary pars intermedia dysfunction (PPID) is associated with increased survival and improved quality of life. Diagnosis includes measurement of adrenocorticotropic hormone (ACTH) and insulin concentrations for PPID and EMS, respectively.

**Hypothesis/Objectives:** To characterize the fluctuations in ACTH and insulin levels in healthy horses in Saskatchewan.

**Animals:** Study 1: Fifty‐two (*n =* 52) client‐owned horses. Inclusion criteria: no recent travel outside of Saskatchewan, normal physical exam findings, no history or evidence of ongoing illness.

Study 2: Eighty (*n =* 80) client‐owned horses. Inclusion criteria: same as Study 1.

**Methods:** Study 1: Prospective, observational study. Blood samples were collected in spring and fall 2021, and ACTH and insulin concentrations were determined by chemiluminescence. Univariable and multivariable linear regression models were used.

Study 2: Prospective, observational study. Monthly sample collection started in July 2022 and will continue for a total of 12 months. Sample processing is the same as Study 1. Analysis will be performed with a generalized linear model.

**Results:** Study 1: ACTH and insulin levels increased in the fall compared to spring (*P* < 0.01). Age and fall ACTH levels were significantly associated (*P* = 0.005).

Study 2: Data collection is ongoing. Preliminary results will be available by June 2023.

**Conclusions and Clinical Importance:** Results so far highlight the seasonal trends in both ACTH and insulin levels in healthy horses in the Canadian prairies. These results will help characterize fluctuations in these biomarkers, in order to better understand their regional behavior and improve diagnostic accuracy.

## Abstract E14: Enteroinsular Axis in Healthy Horses and Horses With Colitis

### **Amina M. Ayubi** ^1^; Ramiro Toribio^2^; Adronie Verbrugghe^1^; Rosalie Fortin‐Trahan^1^; Ahmed Kamr^2^; David Renaud^1^; Diego Gomez^1^

#### ^1^University of Guelph, Guelph, ON, USA; ^2^The Ohio State University, Columbus, OH, USA

**Background:** Hyperglycemia is associated with mortality in horses. A key system in controlling glycemia and pancreatic function is the enteroinsular axis which consists of the incretin hormones glucagon‐like peptide‐1 (GLP‐1), glucagon‐like peptide‐1 (GLP‐2), and glucose‐dependent insulinotropic polypeptide (GIP). Information on incretins in healthy and sick horses is lacking.

**objective:** Investigate the enteroinsular axis in healthy horses and horses with colitis and to determine their association with mortality.

**Animals:** 29 healthy horses and 91 horses with colitis (66 survivors, 24 non survivors).

**Methods:** Prospective case control study. Plasma insulin, glucose, GLP‐1, and GIP concentrations were determined on admission. Data were analyzed by non‐parametric methods and univariate analysis.

**Results:** Glucose was numerically, but not statistically, higher in non‐surviving (n =23; 7.5 mg/dl, 4.8–9.5) than surviving (*n =* 63; 5.9 mg/dl, 5.3–7) horses with colitis (*P* = 0.05). GIP was significantly lower in horses with colitis (*n =* 93, median: 42 ng/ml, range: 31–90) than healthy (*n =* 29; 105 ng/ml, 50–165) horses (*P* = 0.01). GIP was lower in non‐surviving (*n =* 24; 36 ng/ml, 17–53) than surviving (*n =* 66; 49 ng/ml, 33–97) horses with colitis (*P* = 0.01). Horses with colitis and low GIP were more likely to die or be euthanized (O*R =* 1.02, 95% CI = 1.002–1.036). GLP‐1 was significantly higher in horses with colitis (*n =* 18, median: 48 ng/ml, range: 34–69) than healthy (*n =* 4; 22 ng/ml, 18–29) horses (*P* = 0.01).

**Conclusions:** Dysregulation of the enteroinsular axis occurred in horses with colitis. Hyperglycemia in non‐surviving horses with colitis can be explained, at least in part, by impaired intestinal production of GIP.

## Abstract E15: Effect of Early and Late Sampling on the 10‐ and 30‐Minute Thyrotropin‐Releasing Hormone Stimulation Test

### **Dante M. Vorster** ^1^; Wenqing Wang^2^; Kate Kemp^2^; Nicholas Bamford^3^; François‐René Bertin^2^

#### ^1^Utrecht University, Utrecht, Netherlands; ^2^The University of Queensland, St. Lucia, QLD, Australia; ^3^Melbourne Veterinary School, The University of Melbourne, Melbourne, VIC, Australia

**Background:** The thyrotropin releasing hormone (TRH) stimulation test is used to diagnose pituitary pars intermedia dysfunction (PPID) by measuring ACTH concentration either 10‐ or 30‐min post‐TRH administration. Imprecise sampling time leads to significant ACTH variability in the 10‐min protocol.

**Hypothesis/Objectives:** Determine if imprecise sampling time yields significant differences in ACTH concentrations using the 30‐min protocol of the TRH stimulation test.

**Animals:** Twenty‐seven horses >12 years, including 12 horses with PPID.

**Methods:** Blood was collected from each horse 0, 9, 10, 11, 29, 30 and 31 min after intravenous injection of 1 mg of TRH. ACTH concentrations were determined by chemiluminescent immunoassay. Differences in ACTH concentrations and variability in ACTH concentrations between protocols were analyzed with a one‐way ANOVA and visualized with Bland‐Altman plots. Significance was set at *P* < 0.05.

**Results:** Early or late sampling yielded a significant difference in ACTH concentrations using the 10‐min protocol among all horses, the control horses but not those with PPID (*P* = 0.04, 0.01 and 0.37 respectively). There was no significant difference with the 30‐min protocol in any group (*P* = 0.45, 0.21 and 0.63). No significant differences in the variability in ACTH concentrations between early or late sampling in either protocol detected in any group (*P* = 0.35, 0.24, 0.34, respectively).

**Conclusions and Clinical Importance:** The 30‐min protocol of the TRH stimulation test is less affected by imprecise sampling and might be more useful in clinical settings. Precise timing during TRH stimulation tests remains fundamental for the diagnosis of PPID.

## Abstract E16: Glucagon‐Like Peptide‐1 Is Produced by the Equine Large Intestine *In Vitro*

### **Melody A. de Laat** ^1^; Danielle Fitzgerald^2^, BSC PhD; Christina Cash^2^; Martin Sillence^3^

#### ^1^Queensland University of Technology, Brisbane City, QLD, Australia; ^2^Queensland University of Technology, Brisbane City, QLD, Australia; ^3^Professor, Queensland University of Technology, Brisbane City, QLD, Australia

**Hypothesis/Objectives:** Glucagon‐like peptide‐1 (GLP‐1; encoded by the gene GCG) released from intestinal L‐cells increases post‐prandial insulin release. GLP‐1 secretion has been examined in equine small intestine, but in other species L‐cells are mainly located in the large intestine. The aim of this study was to determine large intestinal GLP‐1 release *in vitro*. A second aim was to compare the sequence of GCG between healthy and insulin‐dysregulated horses.

**Animals:** Tissue samples were sourced post‐mortem from 10 horses at an abattoir. Genomic DNA was collected from 33 horses of varying metabolic status. Sample use was approved by an ethics committee.

**Methods:** Duplicate sections of cecum, ascending and descending colon were rinsed and placed in ice‐cold phosphate‐buffered saline prior to sectioning. Tissues were incubated for 60 min at 37°C in 1 mL Tyrode's buffer with Diprotin and 12 mM glucose. GLP‐1 secretion was normalized to protein content and compared with one‐way, repeated‐measures ANOVA. Hybridised capture was used to generate a sequence for the exons of GCG in healthy and insulin‐dysregulated horses and aligned against the reference genome.

**Results:** More GLP‐1 was secreted from the ascending (66.7 ± 23.7 pM/mg; *P* = 0.01) and descending (62.9 ± 12.6 pM/mg; *P* = 0.006) colon, compared to cecum (9.59 ± 3.75 pM/mg). Sequences for GCG had 100% pairwise identity.

**Conclusions and Clinical Importance:** Functional L‐cells are present in equine large intestine, although their importance is unknown. No variations associated with metabolic status were identified in the coding region of equine GCG.

## Abstract E17: Monthly Variations of a Clinical Pituitary Pars‐Intermedia Dysfunction Score and TRH Stimulation Test in Ponies

### **Isabelle Desjardins** ^1^; Daniel Jean^2^, DACVIM (LA)

#### ^1^VetAgro Sup; ^2^Professor Equine Internal Medicine, Clinical Sciences, University of Montreal, Montreal, QC, Canada

**Background:** Pituitary pars‐intermedia dysfunction (PPID) clinical score (CS) and TRH‐Stimulation test (TRH‐ST) results may vary according to several factors.

**Objectives:** To describe the seasonal variations of a CS combined with TRH‐ST in aged ponies, and their ability to discriminate between PPIDs and non‐PPIDs.

**Animals:** Nine crossbreed mares aged 15–28 years, kept in the same area.

**Methods:** CS (gradation/33 points scale), and TRH‐ST results including basal ACTH concentration ([ACTH]b,), 10‐ and 30‐min post‐stimulation ([ACTH]10; [ACTH]30) were assessed monthly over one year. Results were analyzed using Shapiro‐Wilk, Kruskal–Wallis and Spearman tests.

**Results:** Three mares were PPID, two were Controls and four were Equivocals. Median CS was statistically higher in PPIDs (18.0 ± 3.6) compared to Equivocals (6.5 ± 1.5) and Controls (4 ± 0.5) (*P* < 0.01). CS results showed intra‐individual monthly variations attributed to haircoat changes (*n =* 8), and/or occurrence of several simultaneous infections (*n =* 5), and/or laminitis (*n =* 3), and/or loss of body condition in winter (*n =* 2). May and June allowed optimal detection of hair changes and maximal CS differences between groups.

Annual [ACTH]b, and [ACTH]10,30 were statistically different between the three cohorts (*P* < 0.01). October and November were the most relevant months to differentiate the three groups by measuring [ACTH]b, and May for [ACTH]10—30.

**Conclusions/Clinical Importance:** CS and TRH‐ST monthly variations were observed. Groups were best differentiated in May using the combination of CS and TRH‐ST. The correlation between TRH‐ST and CS results may be variable. Further studies are needed to assess the sensitivity and specificity of the CS.

**Table:** Clinical score used to assess PPID clinical probability

Clinical sign		Points
Hypertrichosis	Generalized Constant: yes/no Regional Sites: Periodic: yes/no	5
Delayed shedding	Since:	3
Persistent guard hair	Sites:	2
Hyperhidrosis	Generalized Regional	2
One infectious site	Etiology: Location:	2
Several infectious sites	Etiology: Location:	4
Divergent hoof rings	Foot/feet:	2
Positive hoof testing	Foot/feet:	1
Stiffness at walk		1
Muscle loss	Top line Generalized	1
Supra orbital bulging		3
Pendulous abdomen		1
Delayed healing	Wound Cornea	3
Suspensory ligament breakdown		2
Depression, lethargy		1
	**Total score**	/33

## Abstract E18: Comparison of Hair Characteristics in Equids Affected With Pituitary Pars Intermedia Dysfunction and Aged Controls

### **Isabelle Desjardins** ^1^; Marion Mosca^2^, DVM, DECVD; Didier Pin^3^, DVM, DECVD

#### ^1^VetAgro Sup; ^2^Assistant Professor, Dermatology, VetAgro Sup; ^3^Professor, Dermatology, VetAgro Sup

**Background:** Progressive hair changes associated with Pituitary pars‐intermedia dysfunction (PPID) are poorly characterized.

**Objectives:** To compare the variations of hair characteristics in PPIDs and aged controls.

**Animals:** Fifty‐seven client‐owned horses and ponies, at least 15‐years‐old, included over the course of four seasons.

**Methods:** At admission, a PPID‐clinical score (CS/33), a TRH stimulation test, a shedding score, a jugular hair root density using dermoscopy (cm2), a trichogram assessing anagen (A)/telogen (T) ratio (%) and mean sternal hair length (cm; on 10 hairs) were performed. Results were analyzed using Shapiro‐Wilk and Kruskal‐Wallis tests.

**Results:** Twenty‐six equines harboring hypertrichosis were PPID, 20 presented equivocal results, and 11 were controls. Median CS was statistically higher in PPIDs (17.0 ± 5.7) compared to Equivocals (6.0 ± 1.7) and Controls (4 ± 0.7) (*P* < 0.01). In healthy controls, shedding scores increased physiologically in spring and fall. Jugular hair density and sternal hair length were maximal in winter, A/T ratio increased in fall and was minimal in summer. Median jugular hair density, A/T ratio, and sternal hair length were significantly different between the 3 groups (*P* < 0.01). Hair changes in the PPID group were characterized by sustained anagen activity, higher hair root density and hair length throughout the year, and a time‐lagged shedding score. Equids in the Equivocal group showed variable intermediate hair changes.

**Conclusions/clinical Importance:** Abnormal shedding and progressive hypertrichosis in PPIDs are characterized by higher hair density, longer hair and sustained anagen hair follicle activity. Further studies are warranted to evaluate the influence of season and breed on haircoat characteristics.

**Table:** Shedding score

Haircoat shedding score	Quantity of the loose hairs while wearing a latex glove and stroking three times in the direction of hair growth on the croup
1	No hair adhering to the glove
2	Few hairs adhering to the glove
3	Plenty of hair adhering to the glove
4	Plenty of hair adhering to the glove Additionally, hair falling onto the floor

Adapted from Schmidt K, et al *Theriogenology* (2017) 97: 170e178.

**Table:** Clinical score used to assess PPID clinical probability

Clinical sign		Points
Hypertrichosis	Generalized Constant: yes/no Regional Sites: Periodic: yes/no	5
Delayed shedding	Since:	3
Persistent guard hair	Sites:	2
Hyperhidrosis	Generalized Regional	2
One infectious site	Etiology: Location:	2
Several infectious sites	Etiology: Location:	4
Divergent hoof rings	Foot/feet:	2
Positive hoof testing	Foot/feet:	1
Stiffness at walk		1
Muscle loss	Top line Generalized	1
Supra orbital bulging		3
Pendulous abdomen		1
Delayed healing	Wound Cornea	3
Suspensory ligament breakdown		2
Depression, lethargy		1
	**Total score**	**/33**

## Abstract E20: Effect of Pergolide in Horses With Insulin Dysregulation With and Without Pituitary Pars Intermedia Dysfunction

### **Nicolas C. Galinelli** ^1^; Nicholas Bamford^2^; Madison Erdodi^1^; Skye Mckenzie^1^; Tobias Warnken^3^; Johanna Sonntag^3^; Patricia Harris^4^; Martin Sillence^5^; Simon Bailey^6^

#### ^1^Faculty of Veterinary and Agricultural Sciences, The University of Melbourne, Melbourne, VIC, Australia; ^2^Senior Lecturer, Veterinary Biosciences, School of Veterinary Medicine, The University of Melbourne, Melbourne, VIC, Australia; ^3^Boehringer Ingelheim Vetmedica GmbH; ^4^Head of the Equine Studies Group, Mars Horse Care; ^5^Academic Lead (Research), School of Biology and Environmental Science, Queensland University of Technology, Brisbane City, QLD, Australia; ^6^Professor, Basic Vet (Preclinical) Sciences, Veterinary Biosciences, School of Veterinary Medicine, The University of Melbourne, Melbourne, VIC, Australia

**Background:** Equine pituitary pars intermedia dysfunction (PPID) is associated with reduced dopamine secretion and many cases are insulin dysregulated (ID), which increases laminitis risk. Dopamine agonists improve insulin sensitivity in other species, but the role of dopamine in insulin regulation in equids is unclear.

**Objectives:** To determine whether dopamine agonist treatment affects tissue insulin sensitivity and/or the post‐prandial insulin response in ID equids, with and without PPID.

**Animals:** Sixteen horses and ponies with ID; eight with concurrent PPID and eight without PPID, paired by age and breed.

**Methods:** Animals were treated (4 weeks) with pergolide mesylate (2 μg/kg PO SID) or no drug, in a randomized crossover study. A combined glucose and insulin test (CGIT) and standard meal test (SMT; 1.2 g/kg BW starch plus 0.11 g/kg BW free sugar) were performed before and after treatment. Glucose and insulin responses were compared using a paired *t*‐tests.

**Results:** Pergolide had no significant effect on insulin sensitivity as measured by the CGIT (45 min glucose and 75 min insulin), nor on the glycemic responses to the SMT, in either the PPID or non‐PPID groups. However, the area under the curve for insulin following the SMT was significantly reduced in the PPID group following pergolide treatment (47251 ± 5609 μIU/ml/min vs. 27655 ± 9089, mean and SEM; *P* = 0.036).

**Conclusions and Clinical Importance:** No evidence was found for pergolide modifying systemic insulin sensitivity in insulin resistant equids with or without PPID. However, pergolide may have beneficial post‐prandial insulin‐lowering effects in PPID cases with ID.

## Abstract E22: Serum Biomarkers of Bone Remodeling in Horses With Colitis

### **Ahmed Kamr** ^1^; Diego Gomez^2^; Luis Arroyo^2^; Rosalie Fortin‐Trahan^2^; Ramiro Toribio^1^

#### ^1^College of Veterinary Medicine, The Ohio State University, Columbus, OH, USA; ^2^Ontario Veterinary College, University of Guelph, Guelph, ON, Canada

**Background:** Hypocalcemia has been documented in horses with gastrointestinal disease and colitis. PTH maintains calcium homeostasis during hypocalcemia by enhancing bone resorption and renal calcium reabsorption. Markers of bone remodeling include C‐terminal telopeptide of type I collagen (CTX‐I; resorption) and osteocalcin (OCN; formation). The skeletal response to hypocalcemia and high PTH concentrations has not been evaluated in critically ill horses.

**Objectives:** To measure markers of bone resorption (CTX‐I) and formation (OCN) and to determine their association with serum PTH, total calcium (tCa), ionized calcium (iCa) and phosphorus concentrations, disease severity, and mortality in horses with colitis.

**Methods:** Prospective, multicenter. 70 horses (55 with colitis and 15 healthy) were included. Blood samples were collected on admission. Serum CTX‐I, OCN, and PTH concentrations were measured by immunoassays. Data were analyzed by non‐parametric methods and univariate analysis.

**Results:** Serum CTX‐1 and PTH concentrations were higher, while serum OCN, tCa, iCa and phosphorus concentrations were lower in horses with colitis compared to healthy ones (*P* < 0.05). Serum tCa and iCa concentrations were lower and PTH higher in non‐surviving horses with colitis (*P* < 0.05). Horses with PTH concentrations >139.9 pg/mL were more likely to die (OR *=* 2.9; *P* < 0.05).

**Conclusions:** Increased PTH and CTX‐I together with reduced OCN, tCa, iCa and phosphorus concentrations in horses with colitis are consistent with increased bone turnover to restore normocalcemia. Excessive PTH response was associated with mortality, suggesting PTH resistance. Elevated PTH and reduced iCa concentrations predicted mortality with fair sensitivity and specificity in horses with colitis.

## Abstract E23: Vitamin D Metabolites and Antimicrobial Peptides in Hospitalized Foals

### **Ahmed Kamr** ^1^; Jamie Summers^1^; Julia Horton^1^; Hailey Snyder^1^; William Gilsenan^2^; Laura Hostnik^1^; Ramiro Toribio^1^

#### ^1^College of Veterinary Medicine, The Ohio State University, Columbus, OH, USA; ^2^Rood and Riddle Equine Hospital, Lexington, KY, USA

**Background:** Hypovitaminosis D is common in critically ill patients, including foals. In addition to calcium and phosphorus homeostasis, vitamin D has antibacterial properties through antimicrobial peptides (β‐defensin‐1 and cathelicidin). These peptides have not been evaluated in equine patients but could play a role in disease progression.

**Objectives:** To measure serum vitamin D metabolites (25‐hydroxyvitamin D [25(OH)D] and 1,25‐dihydroxyvitamin D [1,25(OH)2D] and determine their association with β‐defensin‐1 and cathelicidin concentrations, disease severity and outcome in hospitalized foals.

**Methods:** Prospective, multicenter study. 60 foals <72 h (hospitalized = 41; healthy = 19) were included. Blood samples were collected over 72 h to measure serum 25(OH)D, 1,25(OH)2D, β‐defensin‐1 and cathelicidin concentrations. Data were analyzed by non‐parametric methods and univariate logistic regression.

**Results:** On admission, serum 25(OH)D and 1,25(OH)2D concentrations were lower in hospitalized compared to healthy foals (*P* < 0.05). Serum β‐defensin‐1 and cathelicidin concentrations were lower in all time points in hospitalized than healthy foals (*P* < 0.05). There was a positive correlation between serum β‐defensin‐1 and 25(OH)D in hospitalized foals (*r =* 0.72; *P* < 0.05). Non‐surviving foals had the lowest serum 25(OH)D concentrations (*P* < 0.05). Hospitalized foals with serum 25(OH)D<6.1 ng/mL were more likely to die (OR *=* 5.1; *P* < 0.05).

**Conclusions:** Decreased antimicrobial peptides in the presence of hypovitaminosis D and their association with disease severity indicates that vitamin D directly and through its regulated genes is protective against illness in newborn foals.

## Abstract E24: The Bone‐Pancreas Loop and Incretins in Hospitalized Foals

### **Ahmed Kamr**; Hannah Kinsella; Julia Horton; Laura Hostnik; Teresa Burns; Ramiro Toribio

#### College of Veterinary Medicine, The Ohio State University, Columbus, OH, USA

**Background:** It was recently shown in humans and mice that there is an active regulatory system between bone (osteocalcin; OCN) and the endocrine pancreas (insulin) where OCN promotes insulin secretion and insulin stimulates osteoblast function and OCN secretion. This system (bone‐pancreas loop) appears to be important in energy homeostasis (role of the skeleton on glucose metabolism). There is no information on the bone‐pancreas loop in veterinary or equine medicine.

**Objectives:** To investigate the bone‐pancreas loop in hospitalized foals by measuring serum OCN, insulin and incretins (glucagon‐like peptide‐1 [GLP‐1] and glucose‐dependent insulinotropic polypeptide [GIP]) and determine their association with disease severity and non‐survival.

**Methods:** Prospective, multicenter. 84 newborn foals ≤3 days old (70 hospitalized; 14 healthy). Serum OCN and insulin, and plasma GLP‐1 and GIP concentrations were measured over 72 h. Data analysis was carried out using non‐parametric methods and univariate logistic regression.

**Results:** Hospitalized foals had decreased OCN, insulin and GIP, but higher GLP‐1 concentrations compared to healthy ones (*P* < 0.05). Serum OCN concentrations were positively correlated with insulin in hospitalized foals (*r =* 0.5; *P* < 0.05). Non‐surviving foals had lower OCN and insulin, but elevated GlP‐1 concentrations than survivors (*P* < 0.05). Hospitalized foals with OCN concentrations <8.5 ng/mL were more likely to die (OR *=* 5.4; *P* < 0.05).

**Conclusions:** Decreased blood OCN, insulin and GIP concentrations together with elevated GLP‐1 concentrations could reflect a disruption of systemic inflammation on the bone/pancreas loop to maintain energy homeostasis and osteoblast function in hospitalized foals.

## Abstract E25: Phenylbutazone Administration Does Not Affect Insulin Sensitivity in Horses With Insulin Dysregulation

### **Kate L. Kemp** ^1^; Ka Yuen^2^, Dr; Jazmine Skinner^3^, PhD; Francois‐Rene Bertin^4^, DVM, MS, PhD, FHEA, DACVIM (LAIM)

#### ^1^The University of Queensland, Gatton, QLD, Australia; ^2^PhD Candidate, School of Veterinary Science, The University of Queensland, Gatton, QLD, Australia; ^3^Lecturer (Animal Science), School of Agriculture and Environmental Sciences, University of Southern Queensland, Darling Heights, QLD, Australia; ^4^Associate Professor, School of Veterinary Science, The University of Queensland, Gatton, QLD, Australia

**Background:** Phenylbutazone is used for the management of hyperinsulinemia‐associated laminitis; however, we have shown that it alters insulin and glucose dynamics of horses with insulin dysregulation (ID).

**Hypothesis/Objectives:** Investigate the effect of phenylbutazone on insulin sensitivity in horses.

**Animals:** Seventeen University‐owned adult horses including nine diagnosed with ID and eight controls.

**Methods:** In a randomized crossover design study with a 10‐day washout period between treatments, horses received intravenous phenylbutazone (4.4 mg/kg SID) or placebo. On day eight of treatment, a modified frequently sampled intravenous glucose tolerance test (mFSIGTT) was performed. Insulin and glucose concentrations were measured with an IMMULITE1000 or a handheld glucometer respectively. Minimal model analysis was performed, and changes between treatments assessed with paired *t*‐tests, with *P* < 0.05 considered significant.

**Results:** Phenylbutazone administration did not significantly alter insulin sensitivity in ID or control horses (*P* = 0.3280 and *P* = 0.3617, respectively) nor glucose effectiveness (*P* = 0.1187 and *P* = 0.2585, respectively). There was no significant effect on the acute insulin response to glucose in either group (*P* = 0.6723 and *P* = 0.5867). There was no significant effect on the areas under the curves for insulin (*P* = 0.4202 and *P* = 0.3171) or glucose (*P* = 0.6044 and *P* = 0.1321).

**Conclusions and Clinical Importance:** The changes in insulin and glucose dynamics observed after phenylbutazone administration in ID horses are not mediated by an alteration in insulin sensitivity. Further investigation into the unique mechanisms associated with phenylbutazone administration in ID horses is required.

## Abstract E26: Effects of Phenylbutazone Administration on Insulin Secretion in Horses With Insulin Dysregulation

### **Kate L. Kemp** ^1^; Francois‐Rene Bertin^2^, DVM, MS, PhD, FHEA, DACVIM (LAIM)

#### ^1^The University of Queensland, Gatton, QLD, Australia; ^2^Associate Professor, School of Veterinary Science, The University of Queensland, Gatton, QLD, Australia

**Background:** Phenylbutazone is prescribed for the management of hyperinsulinemia‐associated laminitis; however, non‐steroidal anti‐inflammatory drugs are used in diabetic people to stimulate pancreatic activity and insulin secretion. We have demonstrated that phenylbutazone decreased glucose concentration in horses with insulin dysregulation (ID); however, it did not appear directly regulated by insulin secretion.

**Hypothesis/Objectives:** Phenylbutazone alters pancreatic activity through the entero‐insular axis in horses with ID.

**Animals:** Nineteen University‐owned horses including 10 with ID.

**Methods:** In a randomized crossover trial, horses received intravenous phenylbutazone (4.4 mg/kg daily) or placebo, with a 10‐day washout period between treatments. On day 9 of treatment, an oral glucose test (OGT) was performed. Insulin concentrations were measured with the IMMULITE1000, glucose with a handheld glucometer and gastric inhibitory polypeptide (GIP) with a previously validated ELISA. Changes in insulin, glucose and GIP concentrations, as well as glucose‐induced secretions of insulin and GIP were analyzed with linear mixed effect models and paired tests with *P* < 0.05 considered significant.

**Results:** In horses with ID, phenylbutazone administration decreased post‐OGT glucose concentrations with both lower areas under the curve (*P* = 0.0031) and lower maximum glucose concentrations (*P* = 0.0062). Although, post‐OGT insulin concentrations were not altered by phenylbutazone administration (*P* = 0.7903), a higher glucose‐induced insulin secretion was detected (*P* = 0.0466) which was associated with an increased glucose‐induced GIP secretion (*P* = 0.0200). No phenylbutazone‐induced changes were detected in control horses.

**Conclusion and Clinical Importance:** In horses with ID, phenylbutazone induces a higher pancreatic activity mediated by the entero‐insulin axis.

## Abstract E27: Prevalence of Pituitary Pars Intermedia Dysfunction and Insulin Dysregulation in Horse Breeds Classified by Clade

### **Rachel Lemcke** ^1^; Kelly Graber^2^; Steve Grubbs^2^, DVM, PhD, DACVIM

#### ^1^Amwell Data Services, LLC; ^2^Boehringer Ingelheim

**Background:** The influence of genetics on endocrine disorders is not well understood. Evaluating endocrine disorders among different horse breeds may improve identification and management of these pathologies.

**Hypothesis/Objectives:** The objective was to compare PPID and ID status and frequency of endocrine‐associated signs within horse breeds grouped by clade.

**Animals:** Horses and ponies (*n =* 5,605), ages 10 to 45, with endocrine‐associated clinical signs were tested for endocrine disorders by their veterinarian.

**Methods:** A retrospective analysis was performed on veterinarian‐provided data from a 2016–2020 study of ponies and horses with suspected endocrine disorders. Equids were considered PPID positive if spring basal, spring post‐TRH‐stimulated, or fall basal ACTH levels were >35, >110, or >50 pg/mL, respectively, and considered ID positive if basal insulin levels were >20 μIU/mL. Frequencies of PPID, ID, laminitis, and recurrent infections were analyzed using chi‐square or two‐way ANOVA.

**Results:** PPID and ID rates were highest in Clades 3 and 1 at 61.49 and 72.89 percent, respectively, and lowest in Clades 2 and 4 at 24.05 and 27.93 percent, respectively. All clades had higher rates of ID than PPID except Clades 4 and 6. Recurrent infections were least correlated with endocrine disorders (*P* = 0.0001) and were lowest in PPID+/ID+ horses (*P* = 0.0234) and clades 1–3 and 5 (*P* < 0.0001). Laminitis was observed more frequently in ID+ horses (*P* = 0.0002) and less frequently in clades 4 and 6 (*P* = 0.0051).

**Conclusions and Clinical Importance:** The observed differences in endocrine disorder frequencies among clades highlight the need to better understand the involvement of genetics within endocrine disorders.

**Table:** 

PPID+/ID+		PPID+/ID−			PPID−/ID+			PPID−/ID−		
Clade	Incl. breeds	Qty horses	Perc.	Qty horses	Perc.	Qty horses	Perc.	Qty horses	Perc.	Total qty horses
1	Paso Fino, Peruvian Paso	104	38.10	21	7.69	95	34.80	53	19.41	273
2	Lusitano, Andalusian	10	12.66	9	11.39	31	39.24	29	36.71	79
3	Miniature Horse, Shetland, Dwarf, Icelandic Horse, Fjord, Exmoor	452	47.43	134	14.06	234	24.55	133	13.96	953
4	Clydesdale, Shire, Percheron, Belgian	24	13.41	33	18.44	26	14.53	96	53.63	179
5	Morgan	171	32.02	56	10.49	197	36.89	110	20.60	534
6	Thoroughbred, Hanoverian	275	22.21	297	23.99	233	18.82	433	34.98	1,238
7	American Saddlebred, Tennessee Walking Horse, Standardbred	368	34.39	155	14.49	349	32.62	198	18.50	1,070
8	Arabian, Akhal Teke, Florida Cracker	492	38.47	243	19.00	316	24.71	228	17.83	1,279

**Percentage of horses within clade**

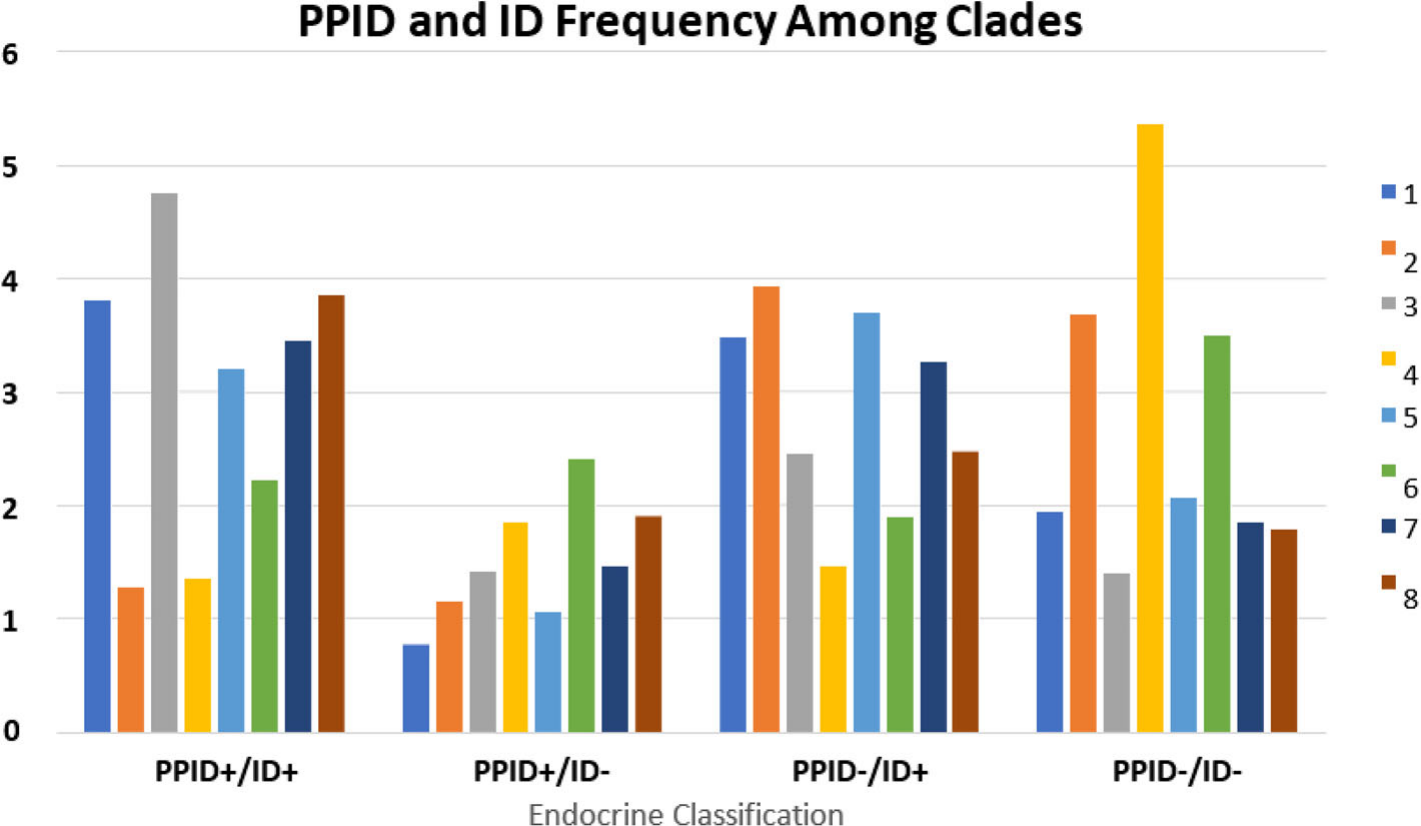

Classification of endocrine disorders (e.g., PPID and/or ID) in eight genetic clades of horses (*P* < 0.0001) showing the variability of these pathologies among horse and pony breeds.

## Abstract E28: Exploring Endocrine Disorders in Sporthorses from Different Geographic Origins

### **Rachel Lemcke** ^1^; Kelly Graber^2^; Steve Grubbs^2^, DVM, PhD, DACVIM

#### ^1^Amwell Data Services, LLC; ^2^Boehringer Ingelheim

**Background:** Sporthorse breeds are frequently trained through upper‐level equestrian disciplines. Since sporthorses often serve as schoolmasters, understanding the prevalence of endocrine disorders (“ED”) in these breeds is important.

**Hypothesis/Objectives:** The objective was to assess PPID and ID status and frequency of endocrine‐associated clinical signs (“EACS”) within different sporthorse breeds grouped by geographic origin.

**Animals:** Sporthorses (*n =* 784), ages 10 to 35, with EACS were tested for ED by their veterinarian.

**Methods:** A retrospective analysis was performed on veterinarian‐provided data from a 2016–2020 study of sporthorses with suspected ED. Equids were considered PPID positive if spring basal, spring post‐TRH‐stimulated, or fall basal ACTH levels were >35, >110, or >50 pg/mL, respectively, and considered ID positive if basal insulin levels were >20 μIU/mL. Frequencies of PPID, ID, and EACS were analyzed using chi‐square or two‐way ANOVA.

**Results:** All four geographic regions evaluated had higher rates of ID than PPID. ED were highest in German sporthorse breeds at 69.72 percent (*P* = 0.0331). PPID and ID rates were highest in German and Spanish sporthorse breeds at 41.73 and 57.89 percent, respectively. Irish sporthorses had the lowest rates of PPID and ID at 26.92 and 42.31 percent, respectively. Rates of Laminitis, decreased athletic performance/lethargy, and delayed/decreased shedding were similar regardless of region or endocrine classification. Regional adiposity was highest in Spanish sporthorses (*P* < 0.0001).

**Conclusions and Clinical Importance:** While clinical conclusions are limited due to sample size, particularly in Irish sporthorses, these high rates of ED and EACS highlight the need for careful management of sporthorses.

## Abstract E29: Histological Changes of the Equine Placenta in the Presence of Insulin Dysregulation

### **Amanda J. Norman** ^1^; Dalen Agnew^2^, DVM, PhD, DACVP; Elaine Norton^3^, DVM, MS, PhD, DACVIM; Chelsey Yob^4^, BS, MS; Jane Manfredi^5^, DVM, MS, PhD, DACVS‐LA, DACVSMR (Equine), PG Cert Vet Med Ed

#### ^1^College of Veterinary Medicine, Michigan State University, East Lansing, MI, USA; ^2^Professor and Chair, Pathobiology and Diagnostic Investigation, College of Veterinary Medicine, Michigan State University, East Lansing, MI, USA; ^3^Assistant Professor, Animal and Comparative Biomedical Sciences, The University of Arizona, Tucson, AZ, USA; ^4^Laboratory Manager, Pathobiology and Diagnostic Investigation, College of Veterinary Medicine, Michigan State University, East Lansing, MI, USA; ^5^Assistant Professor, Pathobiology and Diagnostic Investigation, College of Veterinary Medicine, Michigan State University, East Lansing, MI, USA

**Background:** Gestational diabetes mellitus (GDM) in humans can lead to significant histological and secondary functional placental changes which contribute to fetal hypoxia and growth abnormalities. It is unknown if this occurs in horses.

**Hypothesis:** We hypothesize that the placenta from horses with insulin dysregulation (ID; similar to GDM) will show increased weight, villous abnormalities, and vascular lesions as compared to insulin sensitive (IS) horses.

**Animals:** Ten adult pregnant Arabian mares.

**Methods:** Case control study. Placentas were collected immediately after expulsion. The weight and gross characteristics of the placenta were recorded, along with mare reproductive history. Histological samples were collected from predetermined areas of the placenta using a Swiss roll method and were routinely sectioned and stained. Samples were randomized, read blindly and lesions were scored as not present, mild, moderate, or severe (0–3). Endocrine testing was performed on all mares. Comparisons were made between ID and IS mares using Mann‐Whitney U tests, Student's t‐tests, and Spearman's Correlation Coefficients (significant at *P* < 0.05).

**Results:** There were six IS mares and four ID mares. Mares with ID had more histologic lesions (villous atrophy or immaturity, thickened basement membrane, and vascular changes including proliferation, fibrinoid degeneration, edema, congestion, and hemorrhage; all p values <0.05) and heavier placental weights (*P* = 0.04) than IS mares. There were no significant correlations between histologic scores and age, parity, or BCS.

**Conclusions and Clinical Importance:** Findings from this study could support an equine translational model of placental dysfunction as well as guide peri‐natal mare and foal care.

Figure 1. Mean (**±**standard deviation) histologic placental lesion score from insulin (IS; orange) and insulin dysregulated (ID: brown) mares. All lesions were significantly higher in ID vs. IS mare placentas. *=significant at *P* < 0.05.

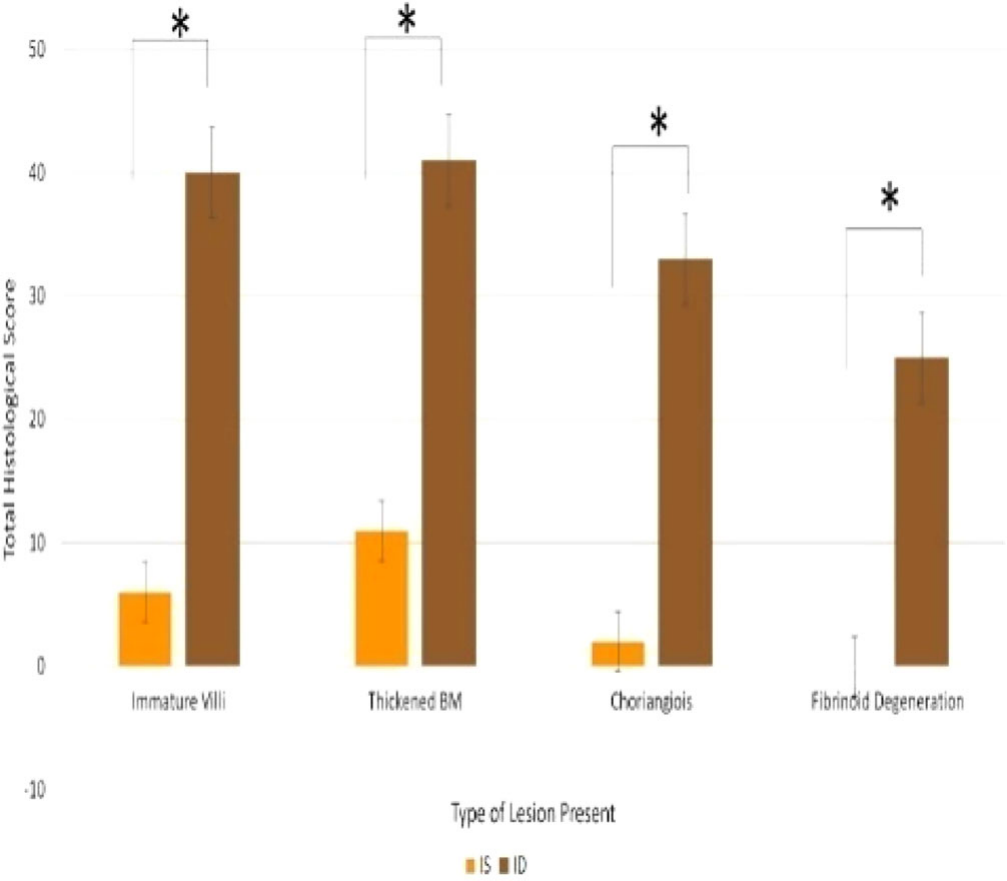

## Abstract E30: Comparison of High‐Dose and Low‐Dose Oral Sugar Test Performance in Horses

### **Kristen Thane** ^1^; Jacquelyn Bowser^2^, DVM, DACVIM (LAIM); Nicholas Frank^1^

#### ^1^Cummings School of Veterinary Medicine, Tufts University, North Grafton, MA, USA; ^2^Johnson & Wales University, Providence, RI, USA

**Background:** Low‐dose (LD) and high‐dose (HD) oral sugar tests have been compared in ponies, but further investigation is required in horses.

**Hypothesis:** That the HD‐oral sugar test (OST) would induce significantly higher blood glucose (BG) and insulin concentrations than the LD‐OST.

**Animals:** Twenty‐four adult horses (median age 16 years) of unknown insulin status.

**Methods:** Horses were administered a LD‐OST and a HD‐OST (0.15 and 0.45 mL Karo syrup per kg body weight, respectively) 7 days apart. Test order was randomly assigned. Blood was collected at T0, T60, and T90 minutes, with BG measured using a handheld glucometer and insulin measured using radioimmunoassay at a commercial veterinary laboratory. Results were compared using Wilcoxon matched‐pairs signed‐rank testing and bias between methods was assessed using Bland‐Altman plots.

**Results:** Median BG and insulin at T0 were not significantly different between methods (P>0.05). Median BG and insulin were significantly higher for the HD‐OST than the LD‐OST at both T60 (*P* < 0.02) and T90 (*P* < 0.01). Mean bias for HD‐OST glucose concentrations was 6.8% at T60 and 10.5% at T90, compared to the LD‐OST. Mean bias for HD‐OST insulin concentrations was 35.4% at T60 and 44.1% at T90 compared to the LD‐OST. Using established cutoffs, ID was diagnosed in one horse using the LD‐OST and two horses using the HD‐OST.

**Conclusion:** HD‐OST yielded significantly higher BG and insulin concentrations at both T60 and T90 than LD‐OST. Test performance should now be investigated in horses with results that fall closer to cutoff values.

## Abstract E31: Comparison of Wellness Ready Lateral‐Flow Insulin Assay to Radioimmunoassay Insulin Results in Equine Blood Samples

### **Kristen Thane** ^1^; Jacquelyn Bowser^2^; Nicholas Frank^1^

#### ^1^Cummings School of Veterinary Medicine, Tufts University, North Grafton, MA, USA; ^2^Johnson & Wales University, Providence, RI, USA

**Background:** The Wellness Ready insulin assay (WR‐INS) is now available to assess insulin status in horses. An independent comparison of this assay with the radioimmunoassay for insulin (RIA‐INS) is warranted.

**Hypothesis:** That WR‐INS concentration would correlate closely with RIA‐INS concentration in horses.

**Animals:** Twenty‐four adult horses (median age 16 years) undergoing routine oral sugar testing to assess insulin status.

**Methods:** 144 EDTA whole blood samples were collected from horses undergoing low‐dose and high‐dose oral sugar tests in a companion study. WR‐INS was performed immediately after sample collection; the remaining sample was centrifuged to separate plasma, which was stored at −80°C until radioimmunoassay analysis at a commercial veterinary laboratory. Results were compared using Spearman correlation, Wilcoxon matched‐pairs signed‐rank testing; bias between methods was assessed using Bland‐Altman plots.

**Results:** Insulin concentration for 78 samples (54%) fell within the working range of the WR‐INS assay (20.0–99.9 μIU/mL). WR‐INS and RIA‐INS results had good correlation for these 78 samples (rs = 0.84, *P* < 0.001). For these time points, median insulin concentrations were significantly higher (*P* < 0.001) for WR‐INS concentrations compared to RIA‐INS concentrations. A mean bias of 17% was detected for WR‐INS compared to RIA‐INS concentrations. Using previously established relevant insulin thresholds for the low‐ and high‐dose oral sugar tests, two horses (8%) had positive results using the WR‐INS and negative results with the RIA‐INS.

**Conclusion:** WR‐INS concentrations are well correlated with RIA‐INS results; however, the WR‐INS results are significantly higher than RIA‐INS, which warrants consideration when interpreting WR‐INS assay results.

## Abstract E32: Abnormal Incretin Concentration in Equine Colic

### **Katarzyna Dembek**; Kate Nierle; Maddie Edwards; Jenna Schirmer

#### North Carolina State University, Raleigh, NC, USA

**Background:** Colic remains the most prevalent and challenging disease in horses. Increased mortality in horses with gastrointestinal disease has been associated with insulin resistance and glucose dysregulation. Dysfunction of the enteroinsular axis (EA), characterized by abnormal concentrations of insulin‐regulating incretins, may contribute to insulin dysregulation, disease progression, and poor outcome in equine colic.

**Hypothesis/Objectives:** We hypothesized that the concentration of incretins glucagon‐like peptide‐1 (GLP‐1), glucagon‐like peptide‐2 (GLP‐2), and glucose‐dependent insulinotropic polypeptide (GIP) would be decreased in horses presented for colic when compared to healthy and associated with non‐survival.

**Animals:** 10 healthy horses and 19 horses with colic (>1‐year‐old).

**Methods:** Incretin concentrations were determined in all horses on admission and at 24, 48, 72 and 96 h of hospitalization in this prospective, longitudinal study. Plasma concentration of incretins was measured with ELISA kits previously validated for horses.

**Results:** In healthy horses, GIP concentration increased at 48, 72 and 96 h compared to baseline (*P* < 0.01). At 24 h, GIP concentration was lower in horses with colic compared to healthy (*P* < 0.05). There were no differences in GLP‐1 and GLP‐2 concentration between time points and group of horses (*P* > 0.05). GLP‐1 concentration was lower in non‐survivors compared to survivors on admission (*P* < 0.05). There were no differences in GIP and GLP‐2 between survivors and non‐survivors (*P* > 0.05).

**Conclusions and Clinical Importance:** The EA dysfunction was characterized by decreased concentration of GIP in horses with colic during the first 24 h of hospitalization. GLP‐1 may be used as a prognosticating factor in horses presented with gastrointestinal disease.

## Abstract E33: Diagnosis of Equine Pituitary Pars Intermedia Dysfunction using TRH‐Stimulation is Repeatable at One‐Week Intervals

### **Steve Grubbs** ^1^; Rhonda Hoffman^2^, PhD, PAS; John Haffner^3^, DVM

#### ^1^Boehringer Ingelheim Animal Health USA, Inc.; ^2^Middle Tennessee State University, Murfreesboro, TN, USA; ^3^Associate Professor, Agriculture, Middle Tennessee State University, Murfreesboro, TN, USA

**Hypothesis:** This study evaluated a hypothesis that the thyrotropin releasing hormone (TRH) stimulation procedure repeated at weekly intervals would yield reproducible diagnosis of Pituitary Pars Intermedia Dysfunction (PPID). Understanding TRH‐stimulation repeatability is useful if TRH‐stimulation testing is initiated but unable to be completed or when PPID research protocols require multiple days of testing. The protocol was approved by the Institutional Animal Care and Use Committee.

**Animals:** Five PPID‐positive and 5 PPID‐negative horses were used.

**Methods:** Basal blood samples were collected, then 1 mg TRH given IV, and blood collected 10 and 30 min after TRH‐stimulation, then repeated on days 7, 14, and 21. Immulite assay analyses of T0‐ACTH, T10‐ACTH, T30‐ACTH on Day 0 vs. subsequent days were compared using a mixed model with repeated measures, Pearson's correlation coefficients, and Bland‐Altman plots.

**Results:** There was no Day effect on T0‐ACTH or T30‐ACTH in PPID‐Negative or PPID‐Positive horses, or T10‐ACTH in PPID‐Negative horses. In PPID‐Positive horses, T10‐ACTH was lower on Day 14 (*P* < 0.036) than Days 7 and 21. While statistically significant, these differences were not clinically relevant, as ACTH remained above the PPID‐Positive diagnostic cutoff. Bland‐Altman plots indicated a Day bias in PPID‐Negative horses of ‐1.97, ‐0.2, and 2.5 pg/mL at T0‐ACTH, T10‐ACTH, and T30‐ACTH respectively; Day bias in PPID‐Positive horses was 44.3, 8.7, and 62 pg/mL at T0‐ACTH, T10‐ACTH, and T30‐ACTH respectively. The Immulite inter‐assay CV was 9.3%, accounting for observed Day biases.

**Conclusions and Clinical Importance:** TRH‐stimulation test repeated at weekly intervals for 21 days provided consistent results.

## Abstract E34: Resting ACTH versus Dynamic ACTH Evaluation for PPID During Fall and Spring in Horses

### **Steve Grubbs** ^1^; Rhonda Hoffman^2^, PhD, PAS; John Haffner^3^, DVM

#### ^1^Boehringer Ingelheim Animal Health USA, Inc.; ^2^Professor, Middle Tennessee State University, Murfreesboro, TN, USA; ^3^Associate Professor, Middle Tennessee State University, Murfreesboro, TN, USA

**Hypothesis:** Compared to resting (T0‐ACTH), dynamic ACTH evaluation 30 min after TRH‐stimulation (T30‐ACTH) will provide more reliable diagnostic for PPID. T30‐ACTH diagnostic cut‐off values will be established.

**Animals:** The protocol was approved by the Institutional Animal Care and Use Committee, with 15 PPID‐positive, 15 PPID‐negative horses used.

**Methods:** In Fall and Spring, blood samples collected before and 30 min after 1 mg TRH IV were analyzed using the Immulite assay. Using T30‐ACTH, the upper 95% level of PPID‐Negative horses and the lower 95% level of PPID‐Positive horses quantified highest Negative and lowest Positive diagnostic limits.

**Results**:

**During Fall**

Mean ± SE of T0‐ACTH and T30‐ACTH, respectively, was 56.8 ± 13.4 and 98.4 ± 19.9 pg/mL in PPID‐Negative; 229 ± 63 and 547 ± 106 pg/mL in PPID‐Positive. The T30‐ACTH 95% Confidence Interval was 55.8–141 pg/mL in PPID‐Negative, 320–774 pg/mL in PPID‐Positive horses. Recommended Fall cutoffs are PPID‐Negative <141 pg/mL and PPID‐Positive >320 pg/mL.

**During Spring**

Mean ± SE of T0‐ACTH and T30‐ACTH, respectively, was 13.8 ± 2.5 and 21.5 ± 3.9 pg/mL in PPID‐Negative; 129 ± 81 and 300 ± 107 in PPID‐Positive. The T30‐ACTH 95% Confidence Interval was 13.1–30.0 pg/mL in PPID‐Negative and 70–530 pg/mL in PPID‐Positive horses. Recommended Spring cutoffs are PPID‐Negative <30 pg/mL and PPID‐Positive >70 pg/mL.

**Conclusions:** PPID‐Negative horses showed similar variation for T0‐ACTH and T30‐ACTH during Fall and Spring, but PPID‐Positive horses had greater variation at T0‐ACTH versus T30‐ACTH during Fall and Spring, suggesting T30‐ACTH is a more reliable test.

## Abstract E35: Functional Profiling of the Fecal Microbiome of Healthy Horses and Those With Colitis

### **Carolyn E. Arnold** ^1^; Bruna Lopes^2^, DVM; Rachel Pilla^3^, DVM, PhD; Jan Suchodolski^4^, MedVet, DrVetMed, PhD, AGAF, DACVM

#### ^1^Texas Tech University, Lubbock, TX, USA; ^2^Graduate Student, Small Animal Clinical Sciences, Texas A&M University, College Station, TX, USA; ^3^Research Assistant Professor, Small Animal Clinical Sciences, Texas A&M University, College Station, TX, USA; ^4^Professor, Small Animal Clinical Sciences, Texas A&M University, College Station, TX, USA

**Background:** Horses with colitis have changes in their fecal microbiome composition based upon 16S rRNA gene sequencing. As multiple bacteria can occupy the same ecological niche, changes in taxonomic composition do not always correspond to changes in microbiome function. Shotgun sequencing is a more accurate way to predict functional microbiome changes by directly measuring functional genes. These changes caused by colitis‐associated dysbiosis in horses have not been described.

**Hypothesis/Objectives:** We hypothesize that horses with colitis will have altered KEGG ortholog pathways compared to healthy horses and propose to quantify those changes using shotgun sequencing.

**Animals:** Healthy horses (*n =* 40) and horses with colitis caused by antimicrobial use (*n =* 14), *Salmonella* (*n =* 14) and *Clostridia* (*n =* 5).

**Methods:** DNA was extracted from feces (PowerSoil Kit, MoBio Labs, Carlsbad, CA, USA) and submitted for shallow shotgun sequencing (Boostershot, Diversigen, Minneapolis, MN, USA). A Kruskal‐Wallis test followed by Tukey's HSD (Metaboanalyst 5.0) was used to identify metabolic pathways that were significantly different between groups. Significance was set at *P* < 0.05 for adjusted p‐values.

**Results:** Significant differences were observed in 224/379 KEGG pathways at L2, and 404/682 KEGG pathways at L3 between groups. These pathways included bacterial transporters, secretion systems, replication factors, and toxins, among others.

**Conclusions and Clinical Importance:** Colitis‐associated dysbiosis is correlated with a significant number of changes in KEGG pathways, indicating that there is major disruption to the functions of the microbiome. These functional changes may represent novel targets for prognostic or therapeutic purposes.

## Abstract E36: Endoscopic Digestive Biopsies in Horses: A Retrospective Study of 36 Cases (2017–2021)

### **Daniel Jean** ^1^; Emmie Vuillier^2^, DVM, PhD, DACVP; Guillaume St‐Jean^3^, DVM, PhD, DACVP; Nanny Wenzlow^4^, DVM, MRCVS, PhD, DACVP

#### ^1^University of Montreal, Montreal, QC, USA; ^2^Private Practitioners, University of Montreal, Montreal, QC, USA; ^3^Assistant Professor, Pathology and Microbiology, University of Montreal, Montreal, QC, USA; ^4^Assistant Professor, Pathobiological Sciences Department, Louisiana State University, Baton Rouge, LA, USA

**Background:** Endoscopic digestive biopsies are commonly taken in horses with a typical clinical context of weight loss, malabsorption, hypoproteinemia and/or recurrent abdominal pain.

**hypothesis**/**Objective:** The objectives are to describe the clinical and histological findings in horses that had endoscopic duodenal and rectal biopsies taken.

**Animals:** This retrospective study evaluated 36 horses with digestive biopsies.

**Methods:** Retrospective clinical study includes horses with complete medical records that also had digestives biopsies taken at the VMTH (2017–2021).

**Results:** Over the past 5 years, we observed an increasing trend of endoscopic duodenal/rectal sampling at our Equine Hospital and identified 36 horses with such biopsy samples. Ten of those horses had no significant histological changes and 26 had increased immune cell infiltrations. Duodenitis/proctitis were most often described as lymphoplasmacytic (54% and 37% from duodenal and rectal tissues respectively). The infiltration intensity ranged in most cases from minimal to mild (82%) with low correlation between duodenal and rectal changes (28%) in the same patient. Horses with increased duodenal infiltrates were also more likely to have an additional diagnosis of gastric glandular ulcers (46%), compared to horses without increased duodenal infiltrates (20%).

**Conclusion and Clinical Relevance:** The clinical usefulness for endoscopic digestive biopsies in horses is likely more important than previously described in the literature and lymphoplasmacytic enteritis is the most frequent histological finding. The histological interpretation of both duodenal and rectal biopsies in equine patients provides a more complete evaluation of the infiltrating immune cells and their intensity.

## Abstract E37: Blood and Peritoneal Lactate as a Marker of Intestinal Ischemia in Horses With Colic

### **Leonardo A. Parra** ^1^; Alejandro Cedeño^2^, DVM, MSc; Nathalie Cotê^3^, DVM, DVSc, DACVS; David Renaud^4^, DVM, PhD; Diego Gomez^3^, DACVIM, MSc, PhD

#### ^1^University of Guelph, Guelph, ON, Canada; ^2^Clinica Equina Equine Hospital; ^3^Assistant Professor, Clinical Studies, University of Guelph, Guelph, ON, Canada; ^4^Population Medicine, University of Guelph, Guelph, ON, Canada

**Background:** Early and accurate identification of ischemic strangulating lesions of the small intestine (SISO) and large colon (LCSO) is crucial to reduce complications and increase survival of horses with colic. The predictive value of Peritoneal (PL) and blood (BL) lactate ratio (PL:BL ratio) and PL‐BL difference for SISO and LCSO is still unclear.

**Objective:** Determine whether PL:BL ratio and PL‐BL difference values are sensitive indicators of SISO and LCSO in horses with colic.

**Animals:** 58 horses with SISO, 71 non‐SISO, 204 non‐LCSO, and 38 LCSO.

**Methods:** Retrospective case‐control study. Final diagnosis was retrieved from medical records. Diagnosis of SISO and LCSO was made in surgery or necropsy. Non‐SISO diagnoses included: proximal enteritis (*n =* 23), ileal impaction (*n =* 41) and others (*n =* 7). Non‐LCSO included nephrosplenic entrapment (*n =* 48), gas colic (*n =* 36), impaction (*n =* 38), colitis (*n =* 27), right dorsal colon displacement (*n =* 27), others (*n =* 28).

**Results:** A PL:BL ratio of 1.5 had a sensitivity and specificity of 0.67 and 0.65 and 0.71 and 0.76 for detection of SISO and LCSO, respectively. The AUC‐ROC for this PL:BL ratio cut‐point was 0.66 and 0.73 for SISO and LCSO, respectively. A PL‐BL difference of 2.2 mmol/L and 2.6 mmol/L had a sensitivity and specificity of 0.79 and 0.82 and 0.79 and 0.88 for detection of SISO and LCSO, respectively. The AUC‐ROC for PL‐BL difference was 0.79 and 0.84 for SISO and LCSO, respectively.

**Conclusions:** PL:BL ratio and PL‐BL difference can be used for diagnosis of SISO or LCSO, although PL‐BL difference may be a better predictor of strangulating lesions.

## Abstract E38: Manipulating Epithelial Fluid Secretion Using Equine Rectal Organoids

### **Breanna Sheahan** ^1^; Lara Madding^2^; Ashley Kropf^2^

#### ^1^College of Veterinary Medicine, North Carolina State University, Raleigh, NC, USA; ^2^North Carolina State University, Raleigh, NC, USA

**Background:** A common mechanism of fluid loss in equine colitis is chloride secretion via the cystic fibrosis transmembrane receptor (CFTR) protein present on epithelial cells. Human rectal biopsies are used to test CFTR function and inhibition. Inhibition of CFTR is a promising target for anti‐diarrheal medications, but there are no current CFTR inhibitors for equine diarrhea.

**Hypothesis:** Rectal organoids will swell in response to CFTR activation and this can be modulated with CFTR inhibitors.

**Animals:** 5 adult healthy horses (four mares, one gelding; age range: 3–21 years) from NC State Research herds or donated for research.

**Methods:** Rectal biopsies were obtained under sedation or post‐euthanasia (unrelated to study). Rectal crypts were isolated and plated to culture organoids. For all assays, organoids were passaged into 96‐well plates and drug application was performed 2–3 days post‐passaging. CFTR modulation was performed with the following activators (forskolin, DCEBIO, IBMX) and inhibitors (CFTRinh‐172, GlyH‐101). Organoid swelling was measured over time with calcein green fluorescence. Drug toxicity 2–3 days post‐assay was identified with resazurin dye.

**Results:** Forskolin induces significant swelling in rectal organoids (Figure 1), consistent with CFTR activation, with no toxicity observed. DCEBIO, IBMX, and CFTRinh‐172 minimally affected swelling, with no toxicity observed. GlyH‐101 impaired forskolin‐induced swelling at the highest dose tested but also exhibited toxicity.

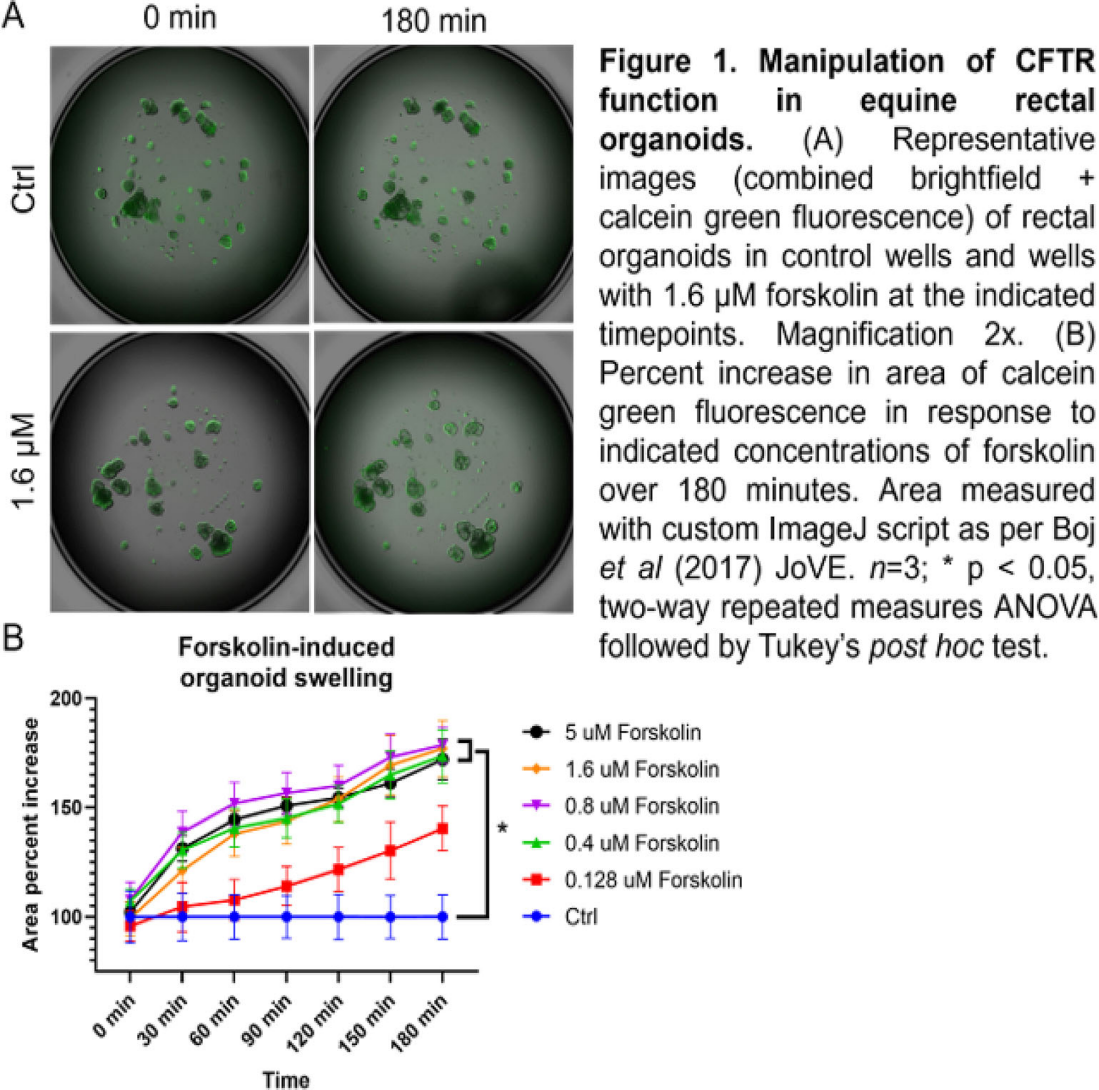

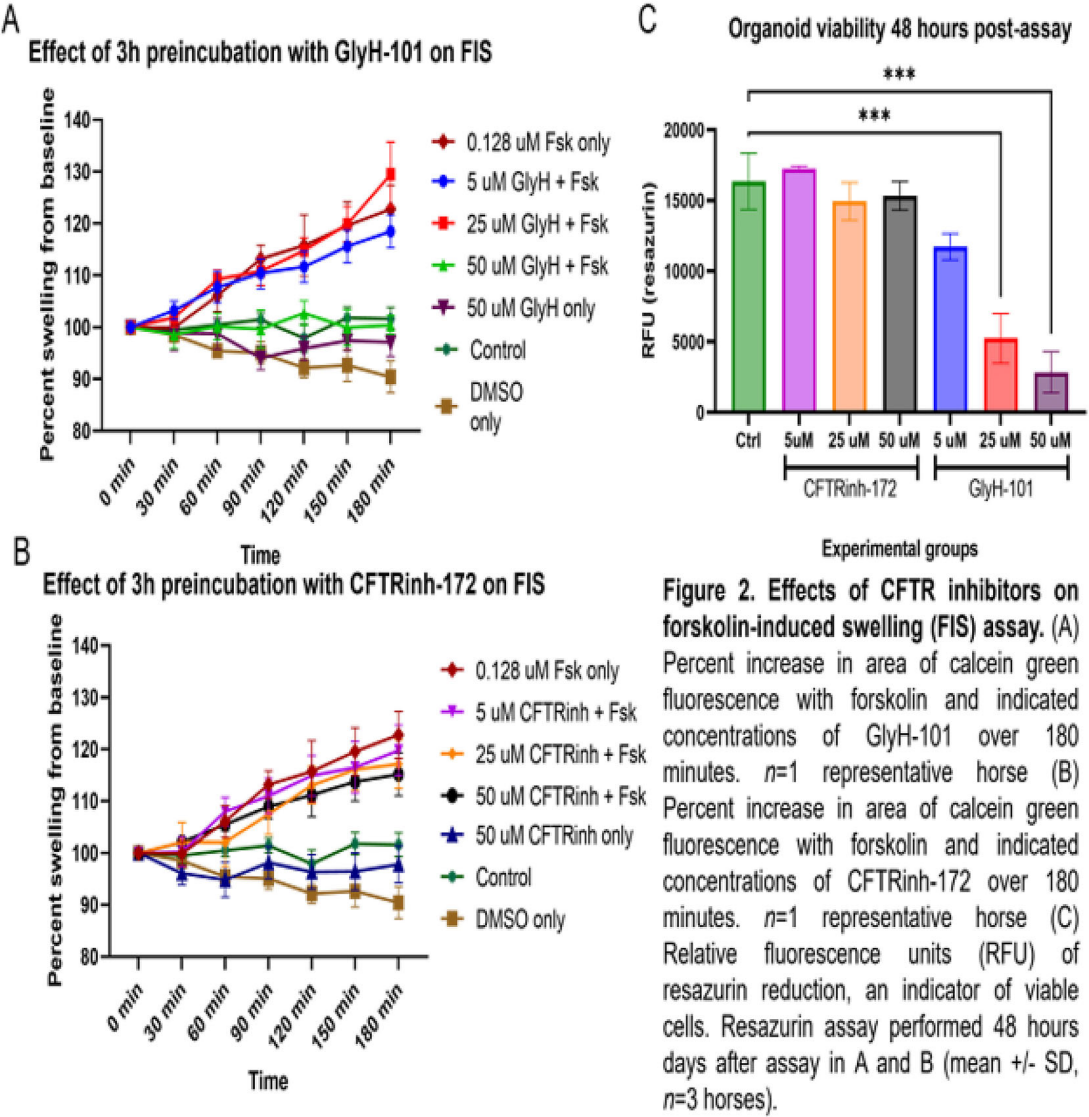

**Conclusions and Clinical Importance:** Equine rectal organoids can serve as an *in vitro* model for chloride and fluid secretion in response to CFTR stimulation. Further experiments are necessary to identify safe and effective CFTR inhibitors for clinical use.

## Abstract E39: Clinical Presentation and Outcome of Gastric Impactions With or Without Concurrent Intestinal Lesions in Horses

### **Sophie E. Talbot** ^1^; Rose Tallon^2^, MA, VetMB, MVetMed, DACVIM, DECEIM, MRCVS; Bettina Dunkel^3^, DVM, PhD, DACVIM, DECEIM, DACVECC, FHEA, MRCVS

#### ^1^Royal Veterinary College Equine Hospital, Hatfield, England, UK; ^2^Equine Internal Medicine Specialist, Equine Internal Medicine, Donnington Grove Veterinary Group, Newbury, England, UK; ^3^Head of RVC Equine and Professor in Equine Internal Medicine, Emergency and Critical Care, Equine Internal Medicine, Royal Veterinary College Equine Referral Hospital, Hatfield, England, UK

**Background:** Gastric impactions (GI) have been identified as primary lesions (lone GI; LGI) or during investigation of other intestinal lesions (concurrent GI; CGI). Anecdotally, CGI resolve faster and carry a better prognosis than LGI.

**Objectives:** To determine clinical, laboratory and ultrasonographic findings, short‐ and long‐term survival in horses with GI, hypothesizing that LGI carry a worse prognosis than CGI.

**Animals:** 71 horses from two referral hospitals (2007–2022).

**Methods:** Retrospective cohort study. GI were defined as feed extending to the margo plicatus after ≥24 h fasting. Clinical parameters, diagnostic findings, and outcome were compared between LGI and CGI. Long‐term survival was determined by questionnaire.

**Results:** Twenty‐seven horses had LGI and 44 had CGI. Large intestinal lesions were more common than small intestinal lesions (32/44 vs. 12/44). CGI resolved more slowly than LGI (2 days (0–8) vs 4 days (1–10); *P* = 0.003). Short‐ (LGI 17/27 vs. CGI 26/44; *P* = 0.74) and long‐term survival (LGI 3.5 ± 1.9 years vs. CGI 2.3 ± 2.3 years; *P* = 0.42) were not significantly different. However, LGI were more likely to experience gastric rupture (LGI 8/27 vs. CGI 5/44; *P* = 0.05). LGI were 8.7 times more likely to require dietary changes (LGI 8/11; CGI 4/16; 95% CI 1.526–49.220; *P* = 0.01). GI recurrence rate was 21.7% (LGI 6/20; CGI 4/26; *P* = 0.23).

**Conclusions and Clinical Importance:** LGI and CGI present similarly with a comparable prognosis, though LGI are more likely to rupture. Long‐term dietary changes are often necessary for LGI cases.

## Abstract E40: The Effect of Medium‐Term Omeprazole on Serum Gastrin and Chromogranin a Levels in the Horse

### **Jessica R. Vokes** ^1^; Bethanie Clark^2^; Ran Shan^3^; Catherine Steel^3^; Benjamin Sykes^4^; Kristene Gedye^4^; Amy Lovett^4^

#### ^1^Massey University, Palmerston North, New Zealand; ^2^School of Veterinary Science, University of Queensland, St. Lucia, QLD, Australia; ^3^Hong Kong Jockey Club, Hong Kong; ^4^School of Veterinary Science, Massey University, Palmerston North, New Zealand

**Background:** Rebound gastric hyperacidity (RGH) secondary to hypergastrinemia has been suggested to contribute to the rapid recurrence of equine squamous gastric disease (ESGD) following omeprazole discontinuation.

**Objectives:** To evaluate changes in serum gastrin and chromogranin A (CgA) concentrations in response to medium‐term omeprazole treatment and following omeprazole discontinuation.

**Animals:** Fourteen mature Thoroughbred racehorses in simulated race training.

**Methods:** Prospective cohort study. All horses received ~4 mg/kg of oral omeprazole daily for 61 days, excluding withholding periods where treatment was stopped as part of a concurrent study (57 treatment days). Serum samples were collected on day 0 prior to omeprazole treatment, on day 1 of each week of the treatment period, and for a further five weeks post‐discontinuation of treatment. Serum gastrin and CgA concentrations were analyzed using radioimmunoassay and ELISA, respectively.

**Results:** Median serum gastrin concentrations had a 2.5‐fold increase from baseline by day 7 (*P* < 0.0005) but did not increase further during the treatment period. Median serum gastrin concentrations returned to baseline within 2–4 days after administration of the last dose of omeprazole. No effect of treatment or discontinuation was seen in serum CgA concentrations.

**Conclusions and Clinical Importance:** Serum gastrin concentrations increased in response to omeprazole treatment but returned to baseline within 2–4 days of the last dose. No effect of treatment or discontinuation was seen on serum CgA concentrations, however, accuracy of the CgA results is limited. The findings do not support the use of extended tapering protocols in horses but suggest that management practices targeted at reducing ESGD risk should be optimized in the 2–4 days following discontinuation of omeprazole.

## Abstract E43: Endothelial Glycocalyx Integrity in Horses With Colitis

### **Diego E. Gomez** ^1^; Luiza Zakia^2^, DVM, DVSc, DACVIM; Emma Jessop^3^, BSc; Ahmed Kamr^4^, PhD; Ramiro Toribio^5^, DVM, MSc, PhD, DACVIM

#### ^1^University of Guelph, Guelph, ON, Canada; ^2^PhD Student, Clinical Studies, University of Guelph, Guelph, ON, Canada; ^3^MSc Student, Clinical Studies, University of Guelph, Guelph, ON, Canada; ^4^The Ohio State University, Columbus, OH, USA; ^5^Professor, The Ohio State University, Columbus, OH, USA

**Background:** In critically ill humans with sepsis, trauma, ischemia/reperfusion, and prolonged hyperglycemia, often lead to diffuse glycocalyx alterations that are linked to endothelial dysfunction. Endothelial glycocalyx (EG) information in healthy horses or horses with colitis is lacking.

**Objective:** Determine the blood concentrations of EG degradation markers in healthy horses and horses with colitis.

**Animals:** 19 healthy horses and 70 horses with acute colitis (52 survivors and 18 non‐survivors).

**Methods:** Prospective case control study. Serum heparan sulfate (HS), syndecan‐1 (Syn‐1), and Angiopoeitin‐2 (Ang‐2) concentrations were determined on admission and compared among groups. Horses were classified as SIRS and non‐SIRS based on alterations in body temperature, heart and respiratory rate and total white blood cells.

**Results:** Serum Ang‐2 concentrations were significantly higher in colitis (*n =* 23; 820 pg/ml, 476–1562) than healthy (*n =* 9; 502 pg/ml, 370–534) horses (*P* = 0.04). Serum HS concentrations were numerically, but not statistically, higher in colitis (*n =* 70, median: 32 ng/ml, range: 21–45) than healthy (*n =* 19; 23 ng/ml, 10–42) horses (*P* = 0.06). Serum concentrations of EG degradation markers were not significantly different in surviving and non‐surviving horses with colitis or SIRS and non‐SIRS horses (*P* > 0.05). A significant positive correlation between HS and Syn‐1 was identified in horses with colitis (*r =* 0.37, *P* < 0.0001).

**Conclusions:** Horses with colitis had glycocalyx injury and endothelial activation biomarkers, but an association between EG degradation markers and mortality was not identified. Investigation of the impact of therapies aiming to preserve EG integrity in horses with colitis are warranted.

## Abstract E44: Prognostic Value of Red Blood Cell Distribution Width, and Other Hematological in Horses With Colitis

### **Pablo Fueyo** ^1^; Luiza Zakia^2^, DVM, DVSc, DACVIM; Janet Beeler‐Marfisi^3^, DVM, DVSc, DACVP; Amy Lack^4^, DVM, MSC, DACVIM; David Renaud^5^; Diego Gomez^4^, DVM, MSc, MVSc, PhD, DACVIM

#### ^1^University of Guelph, Guelph, ON, Canada; ^2^PhD Student, Population Medicine, University of Guelph, Guelph, ON, Canada; ^3^Pathobiology, University of Guelph, Guelph, ON, Canada; ^4^Assistant Professor, Clinical Studies, University of Guelph, Guelph, ON, Canada; ^5^Associate Professor, Population Medicine, University of Guelph, Guelph, ON, Canada

**Background:** In humans, the red blood cell distribution width (RDW), monocyte‐to‐lymphocyte count ratio (MC:LC ratio), platelet‐to‐lymphocyte count ratio (PC:LC ratio), mean platelet volume (MPV), and MPV‐to‐PC ratio (MVP:PC ratio) are associated with mortality in septic patients. The prognostic value of these variables in critically ill horses is unknown.

**Objective:** Investigate whether RDW, MC:LC ratio, PC, PC:LC ratio, MPV, and MPV:PC ratio can predict the mortality of horses with acute colitis.

**Animals:** 131 horses >1‐year‐old with colitis. Composed of 102 surviving and 29 non‐surviving horses.

**Methods:** Retrospective cohort study. Colitis was defined as a horse with diarrhea and leukopenia (total white blood cell count <5×109 cells/L) on admission. Data from admission CBC were used. Survival was defined as discharge from hospital. Data were analyzed by non‐parametric methods and multivariate analysis.

**Results:** The WBC, RDW, MC, PC, MC:LC ratio, and MPV:PC ratio were lower in non‐surviving than surviving horses with colitis (*P* < 0.05, for all comparisons). Colitis horses with lower RDW (OR: 2.28, 95% CI: 1.29 to 4) and PC (OR:1.02, 95% CI: 1.01 to 1.03) values were more likely to die or being euthanized.

**Conclusion:** RDW and PC are independent prognostic markers of mortality in horses with colitis. These variables can be used as markers of systemic dysfunction and a dysregulated inflammatory response in critically ill horses.

## Abstract E45: Two Manual Noncommercial Methods of Platelet‐Rich Plasma Preparation in Donkeys (*Equus asinus*)

### **Cynthia Xue** ^1^; Alexis Hall^1^; Silvia Marchi^1^; Patrice Bernier^1^; Hilari French^2^, DVM, PHD, DACT, DABVP; Robert Gilbert^3^, BVSc, MMedVet, DACT, FRCVS; Lorenzo Segabinazzi^4^, DVM, MS, PhD

#### ^1^Ross University School of Veterinary Medicine, Basseterre, St. Kitts; ^2^Head of Department, Clinical Sciences, Ross University School of Veterinary Medicine, Basseterre, St. Kitts; ^3^Associate Dean for Academic Affairs/Chief Academic Officer, Ross University School of Veterinary Medicine, Basseterre, St. Kitts; ^4^Assistant Professor, Clinical Sciences, Ross University School of Veterinary Medicine, Basseterre, St. Kitts

**Background:** Developing manual, noncommercial platelet‐rich plasma (PRP) production methods in donkeys would facilitate cost‐efficient field management of various diseases in a species often treated under resource‐limitations.

**Objectives:** To evaluate two methods of PRP production validated in horses (double [DC] versus single centrifugation [SC]) in donkeys.

**Animals:** Six healthy adult donkeys from a research herd.

**Methods:** Whole blood (WB) collected into either a citrate‐phosphate‐dextrose‐adenine blood bag or sodium citrate vacutainer tubes subsequently underwent DC or SC, respectively, to produce PRP. Three collections were performed two weeks apart per animal. Concentrations of platelets ([PLT]), leukocytes ([WBC]), and erythrocytes ([RBC]), and platelet‐derived growth factor‐BB (PDGF‐BB) activity were assessed in WB and PRP derived from both methods.

**Results:** Both protocols concentrated platelets 1.7–5.2‐fold, reduced WBC 1.1–50.4‐fold, and decreased RBC at least 829‐fold compared to WB (*P* < 0.0001). Activity of PDGF‐BB in DC‐PRP and SC‐PRP increased 1.2–7.0‐fold in comparison to WB (*P* < 0.003). In DC‐PRP, [PLT] was 3‐fold (*P* < 0.0001) and PDGF‐BB activity 5.8‐fold (*P* = 0.0007) higher compared to SC‐PRP. Reduction in [WBC] and [RBC] was 42‐fold and 22‐fold greater, respectively, in SC‐PRP than DC‐PRP (*P* < 0.0001), but [WBC] did not differ between WB and DC‐PRP (*P* = 0.61). Double centrifugation yielded higher [PLT] and PDGF‐BB activity in PRP despite greater WBC and RBC contamination than SC.

**Conclusions and Clinical Importance:** Manual, noncommercial methods of equine PRP preparation can be used to produce PRP in donkeys. Future studies are necessary to determine the *in vivo* efficacy of asinine PRP.

## Abstract E46: Impact of Decreased Circulating Red Cell Mass on Viscoelastic Coagulation Results in Healthy Horses

### **Ina Mersich** ^1^; Sandra Yucupicio^2^; Rebecca Bishop^3^; Ana Nobrega^4^; Scott Austin^5^; Anne Barger^6^; Meghan Fick^7^; Pamela Wilkins^8^

#### ^1^University of Illinois; ^2^Large Animal Internal Medicine Resident, Department for Veterinary Clinical Medicine, University of Illinois; ^3^Equine Surgery Resident, Department for Veterinary Clinical Medicine, University of Illinois; ^4^Equine Surgery and Medicine Intern, Department for Veterinary Clinical Medicine, University of Illinois; ^5^Clinical Associate Professor, Department for Veterinary Clinical Medicine, University of Illinois; ^6^Department Head and Professor, Department for Veterinary Clinical Medicine, University of Illinois; ^7^Assistant Clinical Professor, Department for Veterinary Clinical Medicine, University of Illinois; ^8^Professor of Equine Internal Medicine, Department of Clinical Veterinary Medicine, University of Illinois

**Background:** Coagulopathy is common in equine critical disease with early recognition crucial for patient management and prognosis. Increased red blood cell mass (RCM) creates viscoelastic (VE) hypocoagulable profiles in dogs and horses. While small animal studies show *in vitro* VE hypercoagulability with decreased RCM, there are no data in horses to date.

**Hypothesis/Objectives:** Acepromazine induced splenic volume increase is associated with decreased RCM and altered VE profiles.

**Animals:** Eight healthy adult horses from the University of Illinois research herd.

**Methods:** Non‐randomized, controlled experimental study. Acepromazine (0.1 mg/kg) IV. CBC, fibrinogen, PT, PTT, PCV, TP and VE testing with VCM Vet performed stallside: pre‐acepromazine administration(T0), 1 h post(T1) and 12 h post(T2). Splenic volumes determined ultrasonographically. One‐way repeated measures ANOVA was performed to determine the effect of time (sample) on the VCM Vet and CBC parameters, as well as spleen volume. Where a significant effect was identified (*P* < 0.05), pair‐wise comparisons between groups were performed by Tukeys HSD.

**Results:** PCV decreased ~13% following acepromazine administration, *P* < 0.001 (T1–T0 *P* < 0.001; T2–T0 *P* < 0.001; T2–T1 *P* < 0.001). MCF (maximal clot firmness) increased *P* = 0.04 (T0–T1 *P* = 0.03), WBC decreased *P* < 0.001(T1–T0 *P* < 0.001; T2–T1 *P* = 0.006), and splenic volume increased *P* = 0.045 (T1–T0 *P* = 0.04). No other examined variables were significantly altered.

**Conclusions and Clinical Importance:** This normovolemic non‐inflammatory acute model of equine anemia demonstrated both VE hypercoagulability‐increased MCF‐ and splenic volume increase. A normovolemic subacute RCM removal anemia model in dogs, using native uncitrated blood, demonstrated *in vitro* VE hypercoagulability as decreased CFT (clot formation time), with increased alpha and MCF, potentially artefactual. Horse blood may be similar.

## Abstract E47: The Acute Phase Response of Serum Amyloid A After Routine Vaccination in Healthy Adult Horses

### **Cassandra Baumgarten** ^1^; Katherine Delph Miller^2^, DVM, MS, DACVIM; Elizabeth Davis^3^, DVM, PhD, DACVIM; Laurie Beard^4^, DVM, MS, DACVIM; Christopher Blevins^5^, MS, DVM; Robert Larson^6^, DVM, PhD, DACT, DACVPM (Epidemiology), ACAN

#### ^1^Department of Clinical Sciences, Kansas State University, Manhattan, KS, USA; ^2^Clinical Assistant Professor, Equine Internal Medicine, Clinical Sciences, Kansas State University, Manhattan, KS, USA; ^3^Associate Dean of Clinical Programs, Department Head and Professor, Equine Internal Medicine, Kansas State University, Manhattan, KS, USA; ^4^Clinical Professor and Section Head, Equine Medicine and Surgery, Clinical Sciences, Kansas State University, Manhattan, KS, USA; ^5^Clinical Professor, Equine Field Service, Clinical Sciences, Kansas State University, Manhattan, KS, USA; ^6^Executive Director, Veterinary Medical Continuing Education, Edgar E. and M. Elizabeth Coleman Chair Food Animal Production Medicine; and Professor, Production Medicine, Clinical Sciences, Kansas State University, Manhattan, KS, USA

**Background:** Serum amyloid A (SAA) is an acute phase protein. Routine vaccination is intended to induce an antigen‐specific inflammatory response. In some instances, this response can be debilitating. Characterizing the acute phase inflammatory response after vaccination in healthy horses will provide a baseline for expected SAA expression.

**Hypothesis/Objectives:** The objective was to measure the effect of routine vaccination on SAA concentrations in apparently healthy horses. It was hypothesized that SAA would increase following routine vaccination compared to placebo administration.

**Animals:** Apparently healthy client‐owned horses (*n =* 21) and Kansas State University College of Veterinary Medicine teaching horses (*n =* 15) were included.

**Methods:** In Experiment 1 (*n =* 8), a blinded, randomized, prospective, cross‐over study was performed. Horses were either vaccinated (rabies, tetanus, West Nile, Eastern and Western equine encephalomyelitis, equine herpesvirus‐1/‐4, influenza) or administered saline, and SAA was measured at 6, 12, and 24 h and daily until day 10. In Experiment 2 (*n =* 28), a prospective, observational study measured SAA post‐vaccination at 12 and 24 h and daily until day 10.

**Results:** Over time, vaccination resulted in increased SAA concentrations compared to placebo without clinical evidence of an adverse reaction. The median SAA post‐vaccination peaked on day 2 (median 1872 μg/ml, interquartile range: 0–2, 402.5) and returned to normal (<20 μg/ml) by day 9 (median 6 μg/ml, interquartile range: 0–23.5) post‐vaccination.

**Conclusions and Clinical Importance:** Routine vaccination results in an increased SAA. This provides evidence for a period of convalescence following vaccination. Measuring SAA following vaccination cannot be used as an indicator of illness.

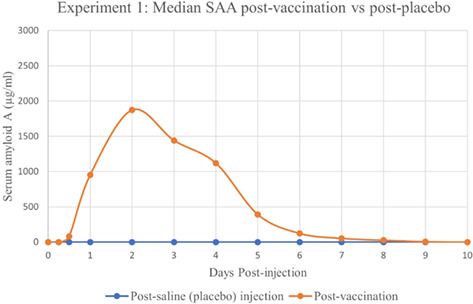

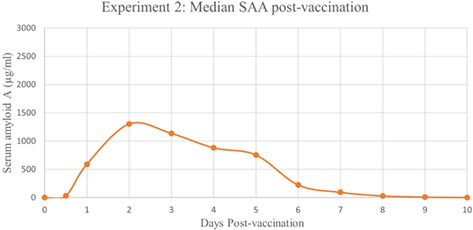

## Abstract E48: Selection of Observation Timepoints With Intradermal Skin Testing in Horses

### **Emily Sundman** ^1^; Laszlo Hunyadi^2^, DACVIM (LAIM)

#### ^1^School of Veterinary Medicine, Texas Tech University, Lubbock, TX, USA; ^2^Research Professor, School of Veterinary Medicine, Texas Tech University, Lubbock, TX, USA

**Background:** Intradermal skin testing in horses is a complicated and time‐consuming procedure in ambulatory settings. Few recommendations currently are available for the selection of time points to evaluate.

**objective:** Compare the results of intradermal skin testing between 15 and 30 min to identify the optimum time for observations in ambulatory settings.

**Animals:** 17 client‐owned horses in Texas.

**Methods:** All horses were clipped and prepped for intradermal skin testing of 23 allergens, 1 positive control (histamine), and 1 negative control (saline). Lesions sides were measured by the maximum diameter 15 and 30 min post‐injection. Saline ratio, histamine ratio, and severity were calculated for each lesion.

**Results:** Statistically significant differences in diameter were identified in 13/23 allergens; however, no statistical differences were found in for the saline ratio, histamine ratio, or in severity (normal, mild, severe). 5/17 horses had a minimum of 4/23 positive allergens (either severe or mild) at 15 min that were normal by 30 min. 8/17 horses had a minimum of 4/23 normal allergens at 15 min that were positive (either severe or mild) by 30 min.

**Conclusions and Clinical Importance:** Further work is necessary to identify the relevance of temporal changes in lesions size when evaluating intradermal skin testing results. Evaluation of multiple time points may be useful for the identifying positive allergens.

## Abstract E49: Investigation of Vitamin D Concentrations in Healthy Horses and Horses With Colitis

### **Rosalie Fortin‐Trahan** ^1^; Luis G. Arroyo^2^; Diego Gomez‐Nieto^2^; David Renaud^2^; Ramiro Toribio^3^; Adronie Verbrugghe^2^

#### ^1^Ontario Veterinary College, University of Guelph, Guelph, ON, Canada; ^2^University of Guelph, Guelph, ON, Canada; ^3^Ohio State University, Columbus, OH, USA

**Background:** Hypovitaminosis D is associated with mortality in critically ill foals. Vitamin D concentrations [VitD] in horses with colitis has not yet been studied.

**Objectives:** Determine the [VitD] in healthy horses and horses with colitis and their association with mortality and the severity of the disease.

**Animals:** Twenty‐five healthy and eighty‐nine horses with colitis.

**Methods:** Prospective case‐control study. Serum [VitD] were measured on admission in horses with colitis. Survival was determined by hospital discharge. Horses were classified as SIRS and non‐SIRS based on alterations in body temperature, heart and respiratory rate, and total white blood cells. Blood concentration of creatinine was also recorded on admission. Data was analyzed by non‐parametric methods.

**Results:** [VitD] were not statistically different between healthy (9.7 ng/ml [4.9–29.6]) and horses with colitis (10.3 ng/ml [3.5–31.3]; *P* = 0.96). In horses with colitis, [VitD] were not different between survivors and non‐survivors (10.4 ng/ml [4.5–31.3] vs. 10.0 ng/ml [3.5–20.9]; *P* = 0.98). Horses with colitis that had SIRS and azotemia (creatinine >130 μmol/L) had significantly lower [VitD] (9.7 ng/ml [3.5–17.0]) than those without SIRS and azotemia (10.8 ng/ml [4.5–31.3]; *P* < 0.05). The survival proportion of horses with colitis, SIRS and azotemia was lower than horses with colitis without these alterations (*P* = 0.04).

**Conclusions and Clinical Importance:** There were no statistical differences in [VitD] between healthy and horses with colitis. However, horses with colitis with systemic inflammation and volume depletion tend to have lower [VitD] and are less likely to survive.

## Abstract E50: Biosurveillance of *Streptococcus Equi* Subsp. *Equi* in Nasal Secretions of 9409 Equids Across the USA

### **Camilo Jaramillo‐Morales** ^1^; Nicola Pusterla^1^; Kaitlyn James^2^; Samantha Barnum^1^; Wendy Vaala^3^; Duane Chappell^3^; Chrissie Schneider^3^; Bryant Craig^3^; Fairfield Bain^3^; Craig Barnett^3^; Earl Gaughan^3^

#### ^1^University of California Davis, Davis, CA, USA; ^2^Massachusetts General Hospital, Boston, MA, USA; ^3^Merck Animal Health, Madison, NJ, USA

**Background:** Strangles has remained a very important respiratory disease in horses worldwide, with little epidemiological studies available.

**Hypothesis/Objectives:** The aim of the study was to describe selected epidemiological aspects of horses with acute onset of fever and respiratory signs testing qPCR‐positive for *Streptococcus equi* subsp. *equi* (*S. equi*).

**Animals:** 9409 horses with acute onset of fever and respiratory signs from all regions of USA were included in a voluntary biosurveillance program from 2008 to 2020.

**Methods:** Information pertaining to demographics, location, season, clinical signs, and vaccination status was collected. Nasal swabs were collected and tested via qPCR for *S. equi* and common respiratory viruses including equine influenza virus, equine herpesvirus‐1/‐4, and equine rhinitis A/B virus.

**Results:** A total of 715 (7.6%) of study horses tested qPCR‐positive for *S. equi*, of which, 226 (32%) had coinfections. The median age for the *S. equi* qPCR‐positive horses was 8 ± 4 years and nasal discharge (639%–89%) and fever (590%–83%) were the most common clinical signs reported in these horses. There was a significantly greater number of horses testing qPCR‐positive for *S. equi* during winter and spring compared to summer and fall (*P* < 0.001). Also, there was a significant difference (*P* = 0.001) in the frequency of reported *S. equi* vaccination between *S. equi* qPCR‐positive (89 horses) and *S. equi* qPCR‐negative cases (824 horses).

**Conclusions and Clinical Importance:** The qPCR prevalence of *S. equi* in the USA was 7.6%, supporting the endemic occurrence of strangles. Further, horses vaccinated against *S. equi* were less likely to test qPCR‐positive against *S. equi*.

## Abstract E51: Endothelial Glycocalyx Integrity in Healthy and Septic Foals

### **Diego E. Gomez** ^1^; Alyssia Perica^2^; Leonardo Parra^3^, MV, MSc; Ahmed Kamr^4^; Ramiro Toribio^5^

#### ^1^University of Guelph, Guelph, ON, Canada; ^2^BSc Student, University of Guelph, Guelph, ON, Canada; ^3^Intern, University of Guelph, Guelph, ON, Canada; ^4^The Ohio State University, Columbus, OH, USA; ^5^Professor, The Ohio State University, Columbus, OH, USA

**Background:** The endothelial glycocalyx (EG) regulates vascular permeability, provides anti‐coagulant and anti‐adhesive effects on the surface of endothelial cells, and can shield endothelial cells from oxidative stress. Pathological injury to the glycocalyx occurs in ischemia‐reperfusion, inflammation, sepsis, and shock.

**objective:** Determine the blood concentrations of EG degradation markers in healthy, sick‐non septic (SNS) and septic foals.

**Animals:** 15 healthy, 13 SNS and 33 septic foals. Sick foals were further divided into 34 survivors and 11 nonsurvivors.

**Methods:** Prospective case control study. Plasma heparan sulfate (HS), syndecan‐1 (Syn‐1), and Angiopoeitin‐2 (Ang‐2) concentrations were determined on admission and compared among groups.

**Results:** Plasma HS concentration was significantly higher in septic (*n =* 33, median: 48 ng/ml, range: 43–55) than healthy (*n =* 15; 37 ng/ml, 19–49) foals (*P* = 0.02). Ang‐2 concentration was also higher in septic (*n =* 19; 1110 pg/ml, 792–1388) than healthy (*n =* 8; 810 pg/ml, 392–520) foals (*P* = 0.04). No differences in plasma Syn‐1 concentrations were identified among healthy, SNS and septic foals, but Syn‐1 was higher in non‐surviving (*n =* 11; 22 ng/ml, 9.5–28) than surviving (*n =* 34; 48 ng/ml, 43–55) foals (*P* = 0.026). No other differences were present between surviving and non‐surviving foals. A significant correlation between HS and Syn‐1 was identified (*r =* 0.54, *P* < 0.0001).

**Conclusions:** Foals with sepsis had glycocalyx injury and endothelial activation biomarkers. EG degradation markers are associated with the outcome of survival in sick foals. Investigation on the pathophysiological importance of EG degradation in septic foals and the impact of therapies aiming to preserve EG integrity are warranted.

## Abstract E52: Antibody Response to Equine Influenza Virus and Herpesvirus‐1 Vaccine in Adult Horses Receiving Intravenous Dexamethasone

### **Nicole Kreutzfeldt** ^1^; Nicola Pusterla^2^, DVM, PhD, DACVIM

#### ^1^Veterinary Medicine Teaching Hospital, University of California‐Davis, Davis, CA, USA; ^2^Professor, Medicine and Epidemiology, University of California‐Davis, Davis, CA, USA

**Background:** The effect of intravenous dexamethasone on the antibody response to a commercial EIV/EHV‐1/EHV‐4 vaccine in healthy adult horses has not been investigated.

**Hypothesis:** Dexamethasone will result in decreased systemic inflammatory response measured by SAA and decreased antibody titers against EIV and EHV‐1 post‐vaccination.

**Animals:** 55 healthy adult horses from the research herd of School of Veterinary Medicine, University of California, Davis.

**Methods:** Randomized cohort study, four study groups. Healthy control (group 1), vaccination only (EIV/EHV‐1, Prestige 2, Merck Animal Health, group 2), vaccination and single dose intravenous dexamethasone (20 mg, group 3), vaccination and intravenous dexamethasone every 24 h for three doses (20 mg each dose, group 4). Serum SAA levels were measured on day 1 and at 72 h. Antibody titers against EIV (hemagglutination inhibition assay, Kentucky 2014 antigen) and EHV‐1 (multiplex ELISA targeting total IgG and IgG 4/7) were measured on day 1 and day 30.

**Results:** Only group 2 showed a significant increase in SAA values after 72 h (*P* = 0.04). There was no statistical difference between pre‐ and post‐vaccination levels of anti‐EHV‐1 antibodies (*P* > 0.05). Group 2 and 3 showed a significant increase in anti‐EIV antibody titers (*P* = 0.05 and *P* = 0.01, respectively). There was no significant difference between the anti‐EIV IgG titers in other study groups.

**Conclusion:** Dexamethasone reduced the SAA response post EIV/EHV‐1/EHV‐4 vaccine but resulted in similar antibody responses to EHV‐1 when compared to vaccinated horses only. A single dose of dexamethasone significantly increased the antibody response to EIV.

## Abstract E54: Detection of Potential Latency Sites of Equid Alphaherpesvirus 1 (EHV‐1) in Kentucky Horses

### **Edward Olajide** ^1^; Amjad Khan^2^, DVM, PhD; Jennifer Janes^3^, DVM, PhD, DACVP; Rebecca Ruby^4^, MSc, BVSc, DACVP, ACVIM‐LAIM; Lutz Goehring^5^, DVM, MS, PhD, DACVIM/ECEIM

#### ^1^University of Kentucky, Lexington, KY, USA; ^2^Visiting Scholar, Veterinary Science, University of Kentucky, Lexington, KY, USA; ^3^Associate Professor, Veterinary Science, Lexington, KY, USA; Veterinary Diagnostic Laboratory, University of Kentucky, Lexington, KY, USA; ^4^Assistant Professor, Veterinary Science; Veterinary Diagnostic Laboratory, University of Kentucky, Lexington, KY, USA; ^5^Professor, Veterinary Science, University of Kentucky, Lexington, KY, USA

**Background:** Reactivation of latent EHV‐1 can result in horizontal spread, leading to abortion and/or myeloencephalopathy (EHM). Historically, the trigeminal ganglion (TG) and upper respiratory tract associated lymphatics (RALT) have been identified as latency sites. An experimental EHV‐1 infection study suggested additional sites of EHV‐1 persistence after recent intranasal infection.

**Hypothesis/Objectives:** Alternative sites of EHV‐1 persistence exist and can be detected in a random horse population.

**Animals:** Animals included 26 horses or ponies over 1 year of age with a postmortem interval less than 24 h, submitted to the University of Kentucky Veterinary Diagnostic Laboratory (UKVDL) for necropsy.

**Methods:** The trigeminal ganglion (TG); dorsal root ganglion (DRG); sympathetic trunk (ST); pharyngeal roof (PhR); retropharyngeal (retLn) and mesenteric (mesLn) lymph nodes. Tissues were formalin fixed and paraffin embedded. Block sections underwent DNA extraction (QIAamp FFPE tissue kit, QIAGEN, Germany). Semi‐quantitative polymerase chain reaction (sqPCR) was used to estimate EHV‐1 genomic DNA.

**Results:** 17 male and 9 female (predominately Thoroughbred) horses with age range 1–25 years (8.90 ± 6.89; median = 7; mode 2) were included. Results are reported in table 1.

**Conclusion and Clinical Importance:** This study confirms the TG and RALT as tissues of EHV‐1 persistence. However, EHV‐1 genome copies were more frequent in PhR than in retLn. This is the first reported detection of EHV‐1 genome in the ST of a random horse population. High abundance of EHV‐1 genome in 2 PhR suggests lytic infection. A larger sample size is required for independent confirmation.

**Table:** Table 1. EHV‐1 genome copy numbers in trigeminal ganglion (TG); dorsal root ganglion (DRG); sympathetic trunk (ST); pharyngeal roof (PhR); retropharyngeal lymph node (retLn); mesenteric lymph node (mesLn)

	TG	DRG	ST	PhR	retLn	mesLn
Positive samples out of total (n) EHV‐1 genome copy abundance	8/25**	1/26	4/26	6/26	2/26	0/25**
Low (CT* >37)	4	0	1	2	1	0
Moderate (CT 34–37)	3	1	2	2	0	0
High (CT 25–33.9)	1	0	1	0	1	0
very high (CT <25)	0	0	0	2	0	0
EHV‐1 positive (%)	32.0	3.8	15.4	23.1	7.7	0

## Abstract E55: Association of *Borrelia burgdorferi* With Cranial Nuchal Bursitis and Elevated OspA‐Specific Serum Antibodies in Horses

### **Erin K. Pearson** ^1^; Toby Pinn‐Woodcock^2^, DACVIM (LAIM); Cassandra Guarino^3^, PhD; Jenna Lambert^4^; Julie Engiles^5^, VMD, DACVP; Kara Brown^5^, VMD, DACVSMR; Marta Cercone^2^, DVM, DACVIM (LAIM), PhD; Jose Garcia‐Lopez^5^, VMD, DACVS, DACVSMR; Amy Johnson^5^, DVM, DACVIM (LAIM & Neurology)

#### ^1^Cornell University, Ithica, NY, USA; ^2^College of Veterinary Medicine, Cornell University, Ithica, NY; ^3^Serology/Immunology, College of Veterinary Medicine, Cornell University, Ithica, NY, USA; ^4^Cummings School of Veterinary Medicine, Tufts University, North Grafton, MA, USA; ^5^School of Veterinary Medicine, University of Pennsylvania, Philadelphia, PA, USA

**Background:** *Borrelia burgdorferi* was identified in one case of equine cranial nuchal bursitis (CNB) with elevated serum OspA antibody value, warranting further investigation.

**Objectives:** To determine the significance of elevated serum OspA antibodies in cases of equine CNB and its correlation with the molecular identification of *B. burgdorferi* in bursal tissue or synovial fluid. Additionally, to compare the histologic changes in CNB with and without detection of *B. burgdorferi*.

**Animals:** Eighteen horses from the northeastern United States with PCR‐confirmed *Borrelia burgdorferi*‐associated CNB and 15 controls with normal cranial nuchal bursa, all unvaccinated for *B. burgdorferi*. Eleven additional horses with histologic diagnosis of CNB and *B. burgdorferi* PCR were included.

**Methods:** Retrospective multicenter cohort study (2013–2022). Medical records from horses with a diagnosis of CNB and *B. burgdorferi* PCR testing on CNB tissue or synovial fluid were reviewed. Serum equine Lyme multiplex assay results were compared between these and controls using an unpaired t‐test. Histologic findings in cranial nuchal bursa tissue were compared between CNB cases with and without *B. burgdorferi* PCR detection.

**Results:** Serum OspA antibody values in *B. burgdorferi* positive cases were significantly elevated (*P* < .001) in horses with CNB (*n =* 12) compared to controls (*n =* 15; Figure 1). Histopathology did not vary between CNB cases with and without *B. burgdorferi* PCR detection.

**Conclusions:** Increased serum OspA antibodies are associated with the presence of *B. burgdorferi* in the cranial nuchal bursa of horses. The role of this pathogen in equine CNB may be underestimated and targeted therapy requires investigation.

**Figure:**
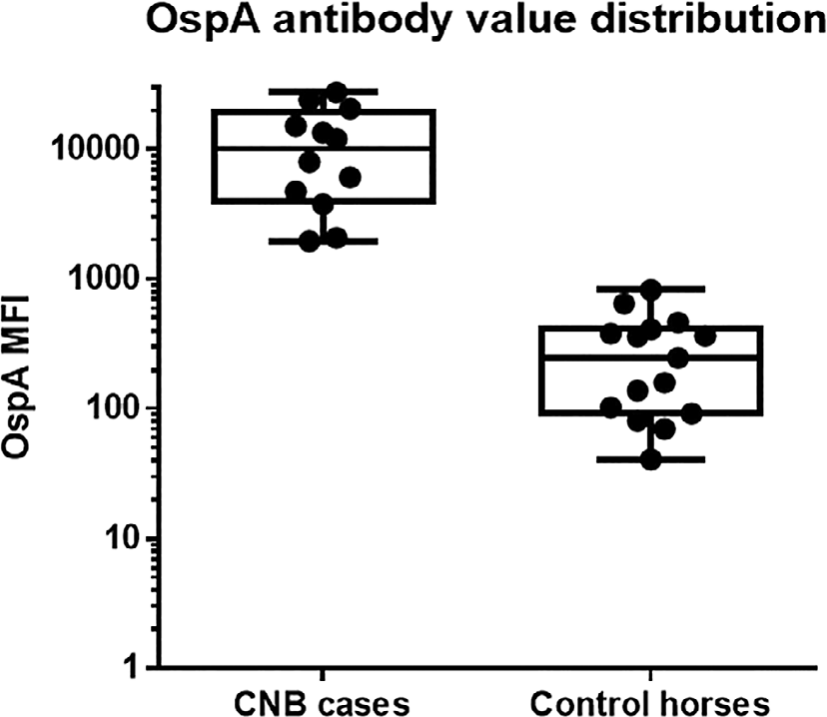
Figure 1. OspA antibody value distribution for clinical cases of cranial nuchal bursitis (*n =* 12) and control horses (*n =* 15) compared by an unpaired t‐test (*P* < 0.001, *t*(25) = 5.048)

## Abstract E56: Investigation of the Inflammatory Response in African Horse Sickness

### **Eva‐Christina Schliewert** ^1^; Emma Hooijberg^2^, DECVP, PhD; Johannes Steyl^3^; Christiaan Potgieter^3^; Geoffrey Fosgate^4^, PhD, DACVP; Amelia Goddard^5^, PhD, MMedVet (ClinPath)

#### ^1^University of Pretoria, Pretoria, South Africa; ^2^Assistant Professor in Clinical Pathology, Department of Companion Animal Clinical Studies, University of Pretoria, Pretoria, South Africa; ^3^Deltamune Ltd, Roodeplaat, South Africa; ^4^Professor in Veterinary Epidemiology, Department of Production Animal Studies, University of Pretoria, Pretoria, South Africa; ^5^Professor in Clinical Pathology, Department of Companion Animal Clinical Studies, University of Pretoria, Pretoria, South Africa

**Background:** African Horse Sickness (AHS) is caused by the African Horse Sickness Virus (AHSV). Infection results in high morbidity and mortality in naïve horses. The nature and role of the immune system and inflammatory cascade has only been investigated *in vitro*, in limited studies.

**Hypothesis:** Experimental AHSV infection results in an inflammatory response.

**Animals:** Four AHSV‐negative horses.

**Methods:** Prospective, longitudinal, experimental study. Following infection with AHSV, blood was obtained q 12 h until humane euthanasia. Complete white blood cell count (WBC), serum amyloid A (SAA), iron concentration, and cytokines IL‐1α, IL‐2, IL‐6, IL‐8, IL‐10, IL‐12, IL‐17, IFN‐γ, TNF‐α, and MCP‐1 were measured.

**Results:** All horses developed severe clinical signs typical of AHS, necessitating humane euthanasia. Statistically significant changes were observed in WBC (*P* = 0.026); however, these were not deemed clinically relevant. Changes in acute phase reactants SAA (*P* < 0.001) and iron (*P* = 0.001) were significant but unexpectedly mild and delayed. Substantial changes in cytokine concentration were observed in TNF‐α (up to 30‐fold) and IL‐10 (up to 150‐fold) towards the final disease stages. All other cytokines remained under the detection limit or only displayed minor increases inconsistent over time and between horses.

**Conclusions and Clinical Importance:** Despite severe clinical signs, no horses mounted an effective inflammatory response indicated by the lack of an inflammatory leukogram and the subtle changes in acute phase reactants. The lack of a proinflammatory cytokine response is unexpected. These findings suggest viral interference with the host innate immune system resulting in a muted inflammatory response.

## Abstract E57: Fecal QPCR Testing for the Detection of Enteric Pathogens in Foals With or Without Diarrhea

### **Alexandre S. Borges** ^1^; Roberta Basso^1^; Pollyana Braga^1^; Fabricio Cerri^1^; Joao Araujo^1^; Jose Pantoja^1^; Marcio Ribeiro^1^; Luis Arroyo^2^; Jose Oliveira‐Filho^1^

#### ^1^Sao Paulo State University (UNESP), São Paulo, Brazil; ^2^University of Guelph, Guelph, ON, Canada

**Background:** Diarrhea is an important clinical problem in foals. The etiological diagnosis should consider coinfections and is essential for treatment and prevention.

**Hypothesis/Objectives:** To detect the genetic material of the most frequently described enteric pathogens in foals with or without diarrhea.

**Animals:** Foals up to one year of age with diarrhea (*n =* 100) and controls (similar age and farms) without diarrhea (*n =* 100).

**Methods:** 3DNA and RNA extraction from fecal samples and real‐time PCR (qPCR) were used to detect *Salmonella* spp., *Clostridioides difficile* and toxins A and B, *Clostridium perfringens*, *Rhodococcus equi* vapA, *Cryptosporidium parvum*, *Giardia duodenalis*, *Lawsonia intracellularis*, *Neorickettsia risticii*, *Rotavirus* and *Coronavirus*. A chi‐square test was used to compare pathogen frequencies and coinfection between groups.

**Results:** The pathogen genetic material was detected in foals with and without diarrhea (respectively): *Salmonella* spp. (31%/9%; p.0001), *C. difficile* (39%/20%; p.003), *C. difficile* tox A and B (20%/8%), *C. perfringens* (26%/16%), *R. equi* vapA (33%/38%), *C. parvum* (17%/1%; p.0001), *Giardia duodenalis* (11%/10%), and rotavirus (1%/0%). *L. intracellularis*, *N. risticii* and coronavirus were not detected. Coinfections were observed in 50 of the foals with diarrhea and 26% of the control foals (p.0005).

**Conclusions and Clinical Importance:** There was a greater number of pathogen detections in foals with diarrhea, and a higher frequency of coinfections than controls. *Salmonella*, *C. difficile*, and *C. parvum* were the pathogens observed at significantly higher frequencies in these two groups. This information can be used to better understand the etiological diagnosis of diarrhea in foals.

## Abstract E58: Prognostic Value of Red Blood Cell Distribution Width and Other Hematological Parameters in Hospitalized Foals

### **Diana Vargas** ^1^; Luiza Zakia^2^, DVM, DVSc, DACVIM; Janet Beeler‐Marfisi^3^, DVM, DVSc, DACVP; Daniel Kenney^4^, DVM; David Renaud^5^, DVM, PhD; Diego Gomez^6^, DVM, MSc, MVSc, PhD, DACVIM

#### ^1^Ontario College of Veterinary Medicine; ^2^PhD Student, Population Medicine, University of Guelph, Guelph, ON, Canada; ^3^Assistant Professor, Pathobiology, University of Guelph, Guelph, ON, Canada; ^4^Clinical Studies, University of Guelph, Guelph, ON, Canada; ^5^Associated Professor, Population Medicine, University of Guelph, Guelph, ON, Canada; ^6^Assistant Professor, Clinical Studies, University of Guelph, Guelph, ON, Canada

**Background:** Red blood cell distribution width (RDW) is a commonly reported variable on the complete blood count (CBC). It is associated with an adverse prognosis in critically ill humans. Furthermore, the neutrophil count (NC), monocyte‐to‐lymphocyte count ratio (MC:LC ratio), platelet‐to‐lymphocyte count ratio (PC:LC ratio), mean platelet volume (MPV), and MPV‐to‐PC ratio (MVP:PC ratio) are associated with increased mortality in septic human patients. The prognostic value of these variables in critically ill foals is unknown.

**Objective:** Investigate the prognostic value of RDW and other hematological variables in hospitalized ill foals.

**Animals:** 228 foals <3‐days‐old. 188 surviving and 40 non‐surviving foals.

**Methods:** Retrospective cohort study. Variables from blood samples collected at the time of admission to the hospital were used in the analysis. Survival was defined as discharge from hospital. Data were analyzed using non‐parametric methods and multivariate analysis.

**Results:** RDW values were similar between non‐surviving and surviving foals (P>0.05). The MC and MC:LC ratio and NC were lower in non‐surviving than surviving foals (*P* < 0.05, for both comparisons). Multivariate analysis showed that foals with lower NC (OR: 1.18, 95% CI: 1.04 to 1.35) and MC:LC ratio (OR: 7.54, 95% CI: 1.25 to 47) values were more likely to die or be euthanized.

**Conclusion:** RDW was not a prognostic marker of mortality in this population of hospitalized foals. However, additional studies evaluating the association of RDW with severity of disease in foals (e.g., sepsis, SIRS) are warranted. Lower neutrophil count and MC:LC ratio were associated with mortality in this population.

## Abstract E59: Detection of *Anaplasma phagocytophilum* in Healthy and Febrile Horses in Central Ohio

### **Kaitlyn McEnaney** ^1^; Jonathan Yardley^2^, DVM; Laura Hostnik^3^, DVM, DACVIM (LAIM); Kathryn Timko^2^, DVM, DACVIM (LAIM)

#### ^1^The Ohio State University, Columbus, OH, USA; ^2^Assistant Professor, Clinical, Equine Field Services, The Ohio State University, Columbus, OH, USA; ^3^Associate Professor, Clinical, Equine Internal Medicine, The Ohio State University, Columbus, OH, USA

**Background:** Equine anaplasmosis is a zoonotic, tick‐borne disease caused by *Anaplasma phagocytophilum* (AP). In the United States, AP is common in the northeast and upper Midwest, although the prevalence in central Ohio is unknown.

**Hypothesis/Objectives:** To determine the seroprevalence of AP in healthy and febrile horses in central Ohio.

**Animals:** Thirty‐three febrile and 259 healthy horses were included in this study. Febrile horses with concurrent respiratory disease were excluded.

**Methods:** A prospective surveillance study evaluated the seroprevalence of AP in apparently healthy and febrile horses in Ohio. Seropositivity was determined using the IDEXX SNAP 4Dx (SNAP) and confirmed with immunofluorescent antibody (IFA). A polymerase chain reaction (PCR) was used to detect AP in all seropositive and febrile horses.

**Results:** Thirty‐one of 259 healthy horses were antibody‐positive for AP on SNAP (31/259, 11.9%), and five of these were PCR‐positive for AP (5/31, 16.1%). Nine of 33 febrile horses were PCR‐positive for AP (9/33, 27%). Of the 33 febrile horses, 6 were SNAP‐positive (6/33, 18%), and 1 of these was PCR‐positive (1/6, 16%). IFA detected antibodies in 76% and 78% of SNAP‐positive healthy and febrile horses, respectively.

**Conclusions and Clinical Importance:** The seroprevalence of AP in healthy horses was 11.9%, suggesting that anaplasmosis has a low seroprevalence in central Ohio. Among febrile horses, 27% were PCR‐positive for AP. However, there was poor agreement between the SNAP and PCR for AP, limiting its utility in diagnosing clinical disease. Additionally, clinicians should cautiously interpret the SNAP in healthy horses, as false positives are common.

## Abstract E60: Calcium, Magnesium, and Phosphorus Concentration in Septic Foals

### **Leonardo A. Parra** ^1^; Luisa Zakia^2^, DVM, DACVIM; Vylan Tran^3^; Amy Lack^1^, DACVIM; Ramiro Toribio^4^, DACVIM, PhD; David Renaud^5^, PhD; Diego Gomez^1^, DVM, DACVIM, PhD

#### ^1^University of Guelph, Guelph, ON, Canada; ^2^Large Animal Internal Medicine, University of Guelph, Guelph, ON, Canada; ^3^Biomedical Science Student, University of Guelph, Guelph, ON, Canada; ^4^The Ohio State University, Columbus, OH, USA; ^5^Population Medicine, University of Guelph, Guelph, ON, Canada

**Background:** Alterations in total (tCa) and ionized (iCa) calcium, total magnesium (tMg), and phosphorus (Pho) homeostasis are well documented in critically ill humans and horses, and have been associated with disease severity. One study reported low iCa and high Pho concentrations in septic foals. There is limited information on calcium, magnesium, and their regulation in septic foals.

**Objective:** Describe blood concentrations of tCa, iCa, tMg, and Pho in septic and sick nonseptic foals, and determine their association with survival.

**Animals:** 144 foals <3 days of age. 91 sick non‐septic (SNS) and 53 septic foals.

**Methods:** A retrospective case‐control study. Sepsis was defined as a foal with a sepsis score >11, blood culture positive, or evidence of sepsis in the postmortem examination. Survival was defined as discharge from the hospital. Data were retrieved from medical records and analyzed by non‐parametric methods and multivariate analysis.

**Results:** Blood concentrations of iCa and tMg were lower in septic foals compared with SNS, whereas tCa and Pho concentrations were higher in septic compared to SNS foals (*P* < 0.05). Positive correlations between tCa and creatinine (*r =* 0.26; *P* < 0.001), tMg and creatinine (*r =* 0.22; *P* < 0.001) were identified. Negative correlations between pH and Pho (*r =* −0.34 *P* < 0.003), pH and tCa (*r =* −0.27; *P* < 0.001), and Pho and tMg (*r =* −0.19; P 0.04) were also detected.

**Conclusions:** Alterations in iCa, tMg, and Pho in septic foals were likely consequences of endocrine dysregulation. The pH and Pho negative correlation suggests that acidemia can promote transcellular Pho redistribution in critically ill foals.

## Abstract E61: Nephrolithiasis in Horses: A Retrospective Study of 25 Cases (2007–2021)

### **Daniel Jean** ^1^; Mathilde Leclere^2^, DVM, PhD, DACVIM (LA); Susana Macieira^3^, DVM, ACVIM (LA); Estelle Manguin^3^, DVM, MSc, DACVIM (LA); Pavlina Ruzickova^3^, DVM, DACVS

#### ^1^University of Montreal, Montreal, QC, Canada; ^2^Professor, Clinical Sciences Department, University of Montreal, Montreal, QC, Canada; ^3^Clinician, Centre Hospitalier Universitaire Vétérinaire, University of Montreal, Montreal, QC, Canada

**Background:** Nephrolithiasis in horses is poorly documented.

**Objectives:** The objectives are to describe the clinical findings, alterations in renal function and short‐term survival rate of horses with nephrolithiasis.

**Animals:** 25 horses with nephrolithiasis presented to the Centre Hospitalier Universitaire Vétérinaire (CHUV).

**Methods:** Medical files from 2007 to 2021 with “nephrolithiasis” as a primary or secondary diagnosis based on ultrasonography were reviewed. Creatinine concentration was determined for 24 out of 25 cases.

**Results:** 25 horses (14 females, 11 males) with a wide age range (4–30 years) were included. The most common breeds were QH (5 cases) and Standardbred (4 cases). The short‐term survival rate was 88% (22/25 cases) and one horse was euthanized 8 months after hospitalization for poor quality of life. Three of these four euthanized horses had grade 3 Acute Kidney Injury (AKI). Hypercreatinemia (highest value during hospitalization) were observed in 16/24 cases (67%) and grades 2 or 3 AKI were detected in 7/24 cases (29%). Nephrolithiasis was diagnosed bilaterally (56% cases) or unilaterally (44% cases), affecting the left and right kidney almost equally. Grade 2 to 3 AKI was observed in horses with unilateral (10%) and bilateral (43%) nephrolithiasis.

**Conclusion and Clinical Importance:** Nephrolithiasis can affect a variety of breeds, a wide range of ages, and both sexes of horses. It can be uni‐ or bilateral, with or without altered renal parameters, or ultrasonographic changes in the renal parenchyma. In some horses, nephrolithiasis may even be an incidental clinical findings.

## Abstract E62: Evaluation of Real‐Time PCR for the Identification of *Sarcocystis neurona* in Equine Cerebrospinal Fluid

### **Carla K. Enriquez** ^1^; Amy Graves^2^; Amy Johnson^3^, DACVIM; Jennifer Morrow^2^

#### ^1^University of Pennsylvania New Bolton Center, Kennett Square, PA, USA; ^2^Equine Diagnostic Solutions, Lexington, KY, USA; ^3^Department of Clinical Studies, University of Pennsylvania, Philadelphia, PA, USA

**Background:** Equine protozoal myeloencephalitis (EPM) caused by *Sarcocystis neurona* remains an ante‐mortem diagnostic challenge for some horses. Recent work suggested the use of real‐time PCR (rtPCR) on cerebrospinal fluid (CSF) as a promising diagnostic tool.

**Objective:** To evaluate the sensitivity and specificity of *S. neurona* rtPCR on CSF for EPM diagnosis from horses with EPM and *S. neurona*‐seropositive horses with other neurologic conditions.

**Animals:** 99 horses with neurologic disease that underwent complete neurologic examination, cerebrospinal fluid collection, and, if euthanized, neurologic necropsy.

**Methods:** Retrospective case‐control study using biobanked CSF samples. CSF samples from neurologic horses with post‐mortem confirmed EPM diagnosis, presumptive EPM diagnosis using strict criteria (SnSAG 2, 4/3 ELISA serum:CSF titer ratios <50) and horses diagnosed with other neurologic diseases were used.

**Results:** There were 52 horses with EPM; 23 were confirmed on postmortem exam, and 29 were presumptive clinical diagnoses. The other 47 horses all had postmortem confirmed diagnoses of other neurologic diseases including equine degenerative myeloencephalopathy (EDM), cervical vertebral stenotic myelopathy, trauma, and others. CSF samples from all horses were tested for *S. neurona* via rtPCR. One CSF sample was weakly positive for *S. neurona* by rtPCR, this sample was obtained from a horse with confirmed EDM. CSF samples from the other 98 horses were negative for *S. neurona* by rtPCR.

**Conclusions and Clinical Importance:** The results from this study contradict previous conclusions that *S. neurona* rtPCR is potentially useful for EPM diagnosis, as they indicate that it has a low sensitivity (0%) for EPM.

## Abstract E63: 8‐hydroxy‐2’‐deoxyguanosine as a Potential Marker of Oxidative Damage in Horses With Neuroaxonal Degeneration

### **Megan Palmisano**; Jeaneen Kulp; Susan Bender, VMD, DACVP, PhD; Darko Stefanovski, PhD; Mary Robinson, VMD, PhD; Amy Johnson, DVM, DACVIM (LAIM and Neurology)

#### University of Pennsylvania New Bolton Center, Kennett Square, PA, USA

**Background:** Equine neuroaxonal dystrophy/equine degenerative myeloencephalopathy (eNAD/EDM) is increasingly recognized as a cause of neurologic disease in adult horses. Diagnosis requires postmortem examination, with no accurate antemortem diagnostic test available. 8‐hydroxy‐2’‐deoxyguanosine(8‐OHdG), a biomarker of oxidative damage utilized in human neurodegenerative disease, has potential to correlate with postmortem diagnosis of eNAD/EDM.

**Hypothesis/Objectives:** We hypothesized that 8‐OHdG will be increased in CSF and serum from eNAD/EDM horses compared to horses with other neurologic diseases (CVSM, EPM) and a control group of neurologically normal horses. 8‐OHdG will be increased in CSF compared to serum of eNAD/EDM horses.

**Animals:** Case‐control study using biobanked samples from 50 horses with postmortem diagnoses (20 with eNAD/EDM, 10 in each group‐ EPM, CVSM, and control).

**Methods:** A commercial competitive ELISA kit (Highly Sensitive 8‐OHdG Check ELISA) previously used in a study of horses with asthma was utilized. Concentration of 8‐OHdG was quantitated in both CSF and serum and compared between groups.

**Results:** The mean concentration of 8‐OHdG amongst all groups was 179.3 pg/mL (41.5–635.4) in CSF and 150.1 pg/mL (36.8–857.5) in serum. Poisson regression showed no significant difference once confounding variables (breed, age, sample age, vitamin E concentration) were considered.

**Conclusions:** 8‐OHdG is detectable in equine CSF and serum, although does not aid in ante‐mortem diagnosis of eNAD/EDM. The findings support the concept that at the time of diagnosis horses with eNAD/EDM do not have ongoing oxidative stress, and that further studies are needed to improve our ability to obtain an antemortem diagnosis.

**Figure:**
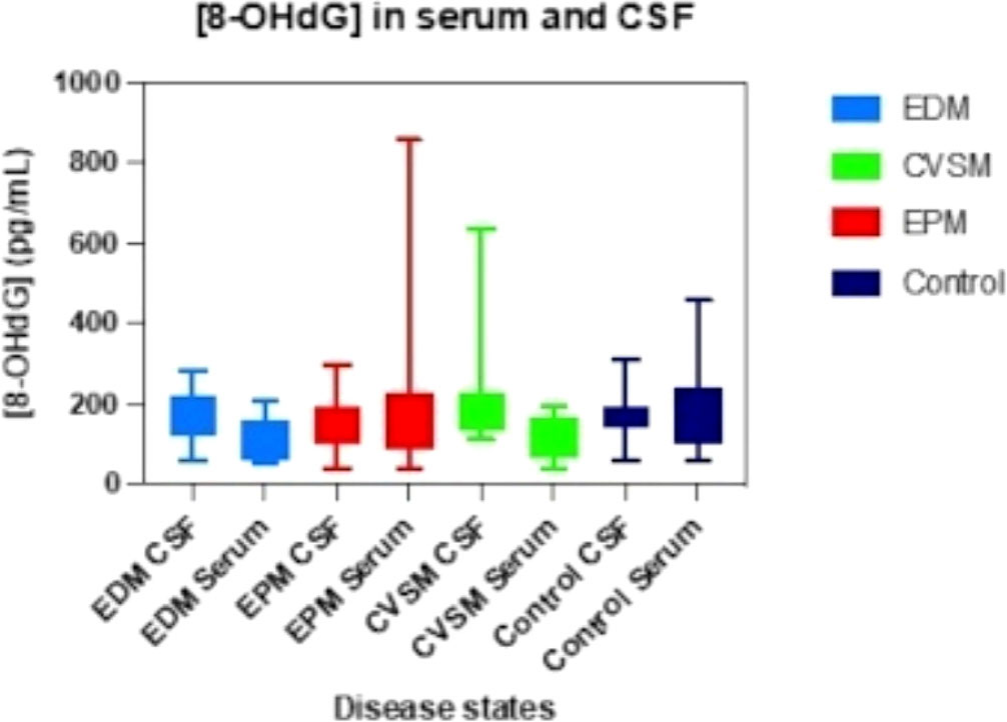
Figure 1. Concentration of 8‐OHdG ([8‐OHdG]) of both CSF and Serum compared across disease state groups. Top and bottom of box indicate first and third quartiles. Vertical lines on both the top and bottom of the box indicating minimum and maximum concentrations measured.

## Abstract E64: Utility of Phosphorylated Neurofilament‐Heavy (pNF‐H) Assay in the Diagnosis of Equine Neurologic Disease

### **Clarisa Romero Hernandez** ^1^; Emilie Setlakwe^2^, DVM, DACVIM‐LAIM; Lisa Edwards^3^, DVM, DACVIM‐LAIM, DACVECC; Carrie Finno^4^, DVM, DACVIM‐LAIM; Amy Johnson^5^, DVM, DACVIM, LAIM, DACVIM; Marilyn M. Simpson^6^

#### ^1^School of Veterinary Medicine, University of Pennsylvania, Philadelphia, PA, USA; ^2^Tryon Equine Hospital, Columbus, NC, USA; ^3^Peterson Smith Equine Hospital, Ocala, FL, USA; ^4^Associate Professor, School of Veterinary Medicine, University of California Davis, Davis, CA, USA; ^5^Associate Professor of Large Animal Medicine and Neurology, School of Veterinary Medicine, University of Pennsylvania, Philadelphia, PA, USA; ^6^Associate Professor of Equine Medicine, Section Chief, Internal Medicine, School of Veterinary Medicine, University of Pennsylvania, Philadelphia, PA, USA

**Background:** Phosphorylated neurofilament‐heavy (pNF‐H), a biomarker of axonal injury and degeneration, has been proposed to assist in the diagnosis of equine neurologic disease. Previous publications suggest limited utility for neonatal hypoxic‐ischemic encephalopathy and equine acute grass sickness, but increased concentrations of neurofilaments seem to occur in equine protozoal myeloencephalitis (EPM), equine motor neuron disease (EMND) and cervical vertebral stenotic myelopathy (CVSM). Recently, a pNF‐H ELISA became commercially available and widely utilized, particularly for horses with suspected neurodegenerative diseases including equine neuroaxonal dystrophy/degenerative myeloencephalopathy (eNAD/EDM). However, the aid of this biomarker in the diagnosis of equine neurological diseases is uncertain.

**Hypothesis/Objectives:** To evaluate the clinical utility of pNF‐H results in horses with neurologic disease.

**Animals:** 178 neurologic cases recruited from three hospitals.

**Methods:** This retrospective study includes horses that underwent a complete neurological evaluation. Serum and cerebrospinal fluid samples were submitted for pNF‐H ELISA analysis. Postmortem diagnosis was included when available.

**Results:** Most neurologic horses did not have increased [pNF‐H]; 15 (8%) had increased serum concentrations (>1 ng/mL) and 38 (21%) had increased cerebrospinal fluid (CSF) concentrations (>3 ng/mL; Table 1). Postmortem confirmation of diagnosis was available for 65 horses and the most common diagnosis was eNAD/EDM.

**Conclusions and Clinical Importance:** The pNF‐H testing is perceived to have a low sensitivity for any specific neurologic disease but cases with increased serum pNF‐H concentration have a likely diagnosis of eNAD/EDM. Further investigations need to be done on its utility for prognosis, monitoring of progression and response to treatment in equine neurologic diseases.

**Table:** Table 1. pNF‐H ELISA results for horses with neurologic disease

Diagnosis (*n*)	Increased serum [pNF‐H] *n* (%)	Increased CSF [pNF‐H] *n* (%)	Increased serum + CSF [pNF‐H] *n* (%)
EPM (*n =* 9)	0	6/9 (67%)	0
Postmortem diagnoses (*n =* 65)			
EDM/eNAD (*n =* 46)	5/46 (11%)	13/46 (28%)	4/46 (9%)
CVSM + EDM (*n =* 5)	0	0	0
Open/other (*n =* 14)	0	1/14 (7%)	0
Negative EPM, no postmortem exam (*n =* 104)	10/104 (10%)	18/104 (17%)	6/104 (6%)
Total (*n =* 178)	15/178 (8%)	38/178 (21%)	10/178 (6%)

## Abstracts E65: Cerebrospinal Fluid Cell‐Free DNA Concentrations in Horses with Common Neurologic Conditions

### **Sarah F. Colmer** ^1^; Mary Robinson^2^; Jeaneen Kulp^3^; Darko Stefanovski^4^; Amy Johnson^5^, DVM, DACVIM‐LAIM, DAVIM (Neurology)

#### ^1^New Bolton Center, School of Veterinary Medicine, University of Pennsylvania, Philadelphia, PA, USA; ^2^Assistant Professor of Veterinary Pharmacology, New Bolton Center, School of Veterinary Medicine, University of Pennsylvania, Philadelphia, PA, USA; ^3^Manager, Laminitis Laboratory, New Bolton Center, School of Veterinary Medicine, University of Pennsylvania, Philadelphia, PA, USA; ^4^Associate Professor of Biostatistics, New Bolton Center, School of Veterinary Medicine, University of Pennsylvania, Philadelphia, PA, USA; ^5^Marilyn M. Simpson Associate Professor of Equine Medicine, Large Animal Internal Medicine, New Bolton Center, School of Veterinary Medicine, University of Pennsylvania, Philadelphia, PA, USA

**Background:** Three commonly diagnosed equine neurologic conditions in North American horses are cervical vertebral stenotic myelopathy (CVSM), equine protozoal myeloencephalitis (EPM) and equine degenerative myeloencephalopathy (EDM)/equine neuroaxonal dystrophy (eNAD), which are often clinically indistinguishable. EDM/eNAD is a diagnosis of exclusion with no confirmatory antemortem biomarkers. Cell‐free DNA (cfDNA) has been associated with neurodegeneration in other species, though no studies have examined its utility in equine cerebrospinal fluid (CSF).

**Hypothesis/Objectives:** The objectives were to determine if equine CSF cfDNA concentrations are detectable and differ significantly between healthy control horses and those with common neurologic diagnoses, and if cfDNA is a candidate biomarker for EDM/eNAD. We hypothesized that cfDNA concentrations would be quantifiable and significantly higher in horses with EDM/eNAD compared with healthy controls and other disease states.

**Animals:** 45 client‐owned horses with confirmed diagnoses.

**Methods:** Case‐controlled study design. Fluorometry was used to determine biobanked CSF cfDNA concentrations from four groups (neurologically normal, CVSM, EPM and EDM/eNAD) which were compared using ANOVA and Kruskal‐Wallis statistics.

**Results:** There were no significant differences in CSF [cfDNA] (median, IQR) between horses with EDM/eNAD (25.65 ng/mL, 18.28–30.38), CVSM (23.95 ng/mL, 17.84–37.81) or EPM (34.90 ng/mL, 29.84–37.61) and neurologically normal controls (34.95 ng/mL, 15.85–45.9), nor between disease groups (*P* > 0.05)(Figure 1).

**Conclusions and Clinical Importance:** This study is the first to evaluate cfDNA in equine CSF. Although cfDNA is a burgeoning research area, these data do not support its candidacy as a biomarker for equine neurodegenerative disease where further efforts are needed.

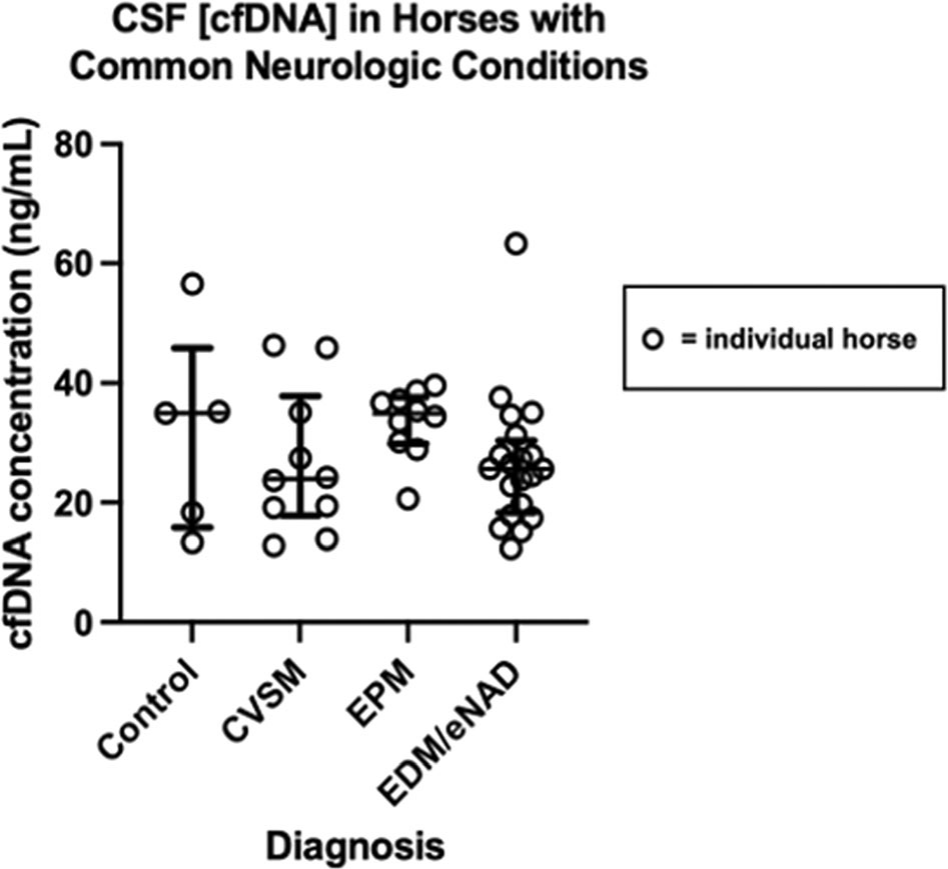

## Abstract E66: Association of Vitamin E‐Responsive Myopathy (VEM) and Equine Degenerative Myeloencephalopathy/Neuroaxonal Dystrophy (EDM/eNAD)

### **Sarah F. Colmer** ^1^; Stephanie Valberg^2^, DVM, PhD, DACVIM, ACVSMR; Susan Bender^3^, BS, PhD, VMD, DACVP; Clarisa Romero Hernandez^4^, MVZ; Amy Johnson^5^, DVM, DACVIM‐LAIM, DACVIM (Neurology)

#### ^1^New Bolton Center, School of Veterinary Medicine, University of Pennsylvania, Philadelphia, PA, USA; ^2^Valberg Neuromuscular Diagnostic Laboratory; ^3^Assistant Professor of Clinical Pathobiology, New Bolton Center, School of Veterinary Medicine, University of Pennsylvania, Philadelphia, PA, USA; ^4^Resident, Large Animal Internal Medicine, Clinical Studies, New Bolton Center, School of Veterinary Medicine, University of Pennsylvania, Philadelphia, PA, USA; ^5^Marilyn M. Simpson Associate Professor of Equine Medicine, Section Chief, Internal Medicine & Ophthalmology, New Bolton Center, School of Veterinary Medicine, University of Pennsylvania, Philadelphia, PA, USA

**Background:** Equine degenerative myeloencephalopathy/equine neuroaxonal dystrophy (EDM/eNAD) is a common postmortem neurologic diagnosis in North American horses. Though temporal deficiency of antioxidant vitamin E is implicated in the etiology, EDM/eNAD remains a diagnosis of exclusion. A vitamin E‐responsive myopathy (VEM) is diagnosed by mitochondrial alterations in highly oxidative type 1 muscle fibers in the sacrocaudalis dorsalis medialis (SCDM) muscle.

**Hypothesis/Objectives:** We hypothesize that mitochondrial alterations in the SCDM may be a proxy for oxidative damage to the central nervous system (CNS). The objective was to determine what percentage of horses with EDM/eNAD exhibit typical VEM histopathology. We also compared percentages of horses with EDM/eNAD exhibiting low serum vitamin E concentrations and increased serum and CSF phosphorylated neurofilament‐heavy (pNF‐H) concentrations.

**Animals:** 38 client‐owned horses evaluated between 2021–2022.

**Methods:** Cohort study. Incisional formalin‐fixed SCDM biopsies were obtained from horses with postmortem‐confirmed EDM/eNAD and histopathology evaluated for mitochondrial alterations using succinate dehydrogenase A stains. Serum [vitamin E] was determined with high‐performance liquid chromatography and [pNF‐H] determined using sandwich ELISA.

**Results:** 15/38 horses (39%) with EDM/eNAD at postmortem exhibited comorbid VEM histopathology in SCDM biopsies. 3/21 (14%) exhibited increased serum [pNF‐H], and 13/21 (62%) exhibited increased CSF [pNF‐H]. 6/31 (19%) had low serum [vitamin E].

**Conclusions and Clinical Importance:** SCDM mitochondrial alterations were identified in >1/3 of horses with EDM/eNAD and can be used to increase suspicion of EDM/eNAD antemortem. Future investigation into the frequency of VEM in horses with other neurologic conditions could enable estimation of specificity.

## Abstract E67: The Effect of Leucine and Pyridoxine on Metabolic Parameters in Ponies with Equine Metabolic Syndrome

### **Elaine M. Norton** ^1^; Kent McKendry^2^; Sydney Plumb^1^; Bob Scharf^2^; Lowell Smalley^3^; Michael Zemel^4^

#### ^1^University of Arizona, Tucson, AZ, USA; ^2^John Ewing Company, La Salle, CO, USA; ^3^Smalley Equine Veterinary Consulting, LLC., Omaha, NE, USA; ^4^NuSirt Bioparma Nashville, TN, USA

**Background:** Insulin dysregulation is the key component of equine metabolic syndrome (EMS) with management regimens focus on improving insulin sensitivity. In dogs and humans, the combination of leucine and pyridoxine has been shown to aid in weight reduction and improve insulin sensitivity. However, the effect of these supplements have not been evaluated in horses.

**Objective:** Determine if the combination of leucine and pyridoxine would improve EMS biochemical parameters.

**Animals:** A total of 38 ponies across five farms were enrolled in a 60 day trial.

**Methods:** Ponies were blocked by farm, age, and sex matched, and randomly assigned to the supplement (*n =* 20) or placebo (*n =* 18) group. Ponies were excluded if they were on medications or supplements labeled for the management of EMS. Ponies were evaluated using a two day testing protocol starting on day −1, 7, 30, and 60. On day one, ponies were weighed on a scale, baseline measurements (insulin, triglycerides, non‐esterified fatty acids, adiponectin, and leptin) were drawn, and a two‐step insulin tolerance test was performed. On day 2, an oral sugar test was performed using 0.45 ml/kg Karo light corn syrup. Chi square analyses and pairwise comparison of the estimated marginal means were performed.

**Results:** Ponies in the supplement group lost more weight (≥2% body weight; *P* < 0.04) and had a larger reduction in their max insulin concentration post oral sugar test (≥40% decrease; *P* < 0.03) compared to the placebo group.

**Conclusion:** These data suggest that this combination supplement may be used to aid in the management of EMS.

## Abstract E68: Complications Associated with Intravenous Catheter Placement in Adult Horses at a Teaching Hospital

### **Kate L. Hepworth‐Warren**; Sara Erwin‐Craig

#### College of Veterinary Medicine, North Carolina State University, Raleigh, NC, USA

**Background:** Complications of intravenous catheters (IVC) are common in hospitalized horses, but specific risk factors associated with placement have not previously been investigated. Specifically, the impact of veterinary student involvement on the likelihood of complications has not been evaluated in horses.

**Objectives:** To investigate factors of IVC placement, including the role of individuals involved that may lead to complications such as thrombophlebitis and subcutaneous inflammation and infection.

**Animals:** 255 IVC placed in hospitalized adult horses over a 9‐month period at a teaching hospital.

**Methods:** Data were collected from the electronic medical record for all horses in which an IVC was placed including the role of the individual that prepared the site and placed the IVC, site of placement, use of sedation, disease category of patient, type of catheter, reason for removal, and presence of complications at time of removal. Data were evaluated retrospectively. Univariate analyses were performed using Fisher's exact test. Significance was set at *P* ≤ 0.05.

**Results/Findings:** 41/255 (16.1%) of IVC were associated with a complication at the time of IVC removal. The relative risk of complications associated with IVCs placed by veterinary students was not significantly different to the relative risk when compared to IVCs placed by non‐student personnel (*P* = 0.35).

**Conclusions and Clinical Importance:** Client perception of student involvement in procedures may affect referral decisions to veterinary teaching hospitals. The data here shows that student involvement in IVC placement does not increase the risk of complications and can be utilized to encourage client support of student involvement.

## Abstract E69: Evaluation of Serum sCD14 Concentration as Biomarker of Illness and Sepsis in Neonatal Thoroughbred Foals

### **Camilla Quattrini** ^1^; Rana Bozorgmanesh^2^; Nicole Kreutzfeldt^1^; Bettina Wagner^3^; Gary Magdesian^1^

#### ^1^University of California, Davis, Davis, CA, USA; ^2^Hagyard Equine Medical Institute, Lexington, KY, USA; ^3^College of Veterinary Medicine, Cornell University, Ithaca, NY, USA

**Background:** Sepsis is a leading cause of mortality in neonatal foals. Early diagnosis is key to improved outcomes, but the diagnosis remains difficult. Soluble CD14 (sCD14) is a promising biomarker for inflammatory diseases in humans, including sepsis.

**Hypothesis/Objectives:** Sick foals have higher serum sCD14 concentrations than healthy foals, and septic foals have higher sCD14 concentrations as compared to sick, nonseptic and healthy foals.

**Animals:** 63 sick (29 septic, 34 nonseptic) and 26 healthy neonatal Thoroughbred foals.

**Methods:** Prospective observational study. Blood samples were collected from hospitalized foals at admission, d3 and prior to discharge or death, and healthy foals on days 1, 3, and 5 of life. Sick foals were categorized as septic or sick, non‐septic based on sepsis score and blood culture results. Serum sCD14 was measured using a previously validated fluorescent bead‐based assay.

**Results:** On day 3, the sick foals combined (962.0, 432.0–2002.0 ng/mL) had higher sCD14 as compared to healthy foals (769.5, 482–1154 ng/mL, *P* = 0.0009). At the last collection time, sCD14 was significantly greater in non‐survivors (1504 ng/mL, 1443–1589) compared to surviving foals (760.0 ng/mL, 357.0–1950; *P* = 0.02) and healthy foals (756.0 ng/mL, 481.0–1381.0) (*P* = 0.03). There were no differences between septic and sick, nonseptic foals at any time point.

**Conclusions and Clinical Importance:** Serum sCD14 concentrations were increased in critically ill foals and non‐survivors. sCD14 could be an adjunctive biomarker of neonatal health status, along with other clinical and clinicopathologic data. However, sCD14 is not necessarily specific to sepsis and there is considerable overlap between sick and healthy foals.

## Abstract E71: Gamma‐Glutamyl Transferase Plasma Levels are Associated With Weekly Workload and Racing Status in Thoroughbred Racehorses

### **Joanne E. Haughan** ^1^; Lauren Pittman^2^, BS; Darko Stefanovski^2^, MS, PhD; Mary Robinson^2^, VMD, PhD, DACVCP

#### ^1^Equine Pharmacology Lab, New Bolton Center, School of Veterinary Medicine, University of Pennsylvania, Kennett Square, PA, USA; ^2^Clinical Studies, New Bolton Center, School of Veterinary Medicine, University of Pennsylvania, Kennett Square, PA, USA

**Background:** Elevated plasma levels of gamma‐glutamyl transferase (GGT) are frequently seen in Thoroughbred racehorses and are thought to be associated with increased training workload and poor performance.

**Hypothesis:** Recent training workload and current racing status influence plasma GGT levels in Thoroughbred racehorses.

**Animals:** 181 venous blood samples were obtained from 60 Thoroughbred racehorses in training (28 females, 16 intact males and 16 geldings, aged 1–7 years, mean: 3.1 ± 1.2 years).

**Methods:** Samples were obtained 1–4 h after morning workouts on a non‐race day. Age, sex, whether the horse was racing (R) and number of days since last raced (DLR) was recorded. Maximum training workload on the morning of sampling (WLAM) and during the previous week (WLWK) was obtained for 131 and 90 samples respectively. Workload was categorized: none/walk/jog/gallop/breeze/race. Plasma was analyzed for GGT concentration using a Vitros XT 3400 chemistry system (Ortho Clinical Diagnostics, NJ, USA). Stepwise regression was used to identify which parameters significantly influenced GGT, then a multivariable mixed‐effects regression model was used to analyze fixed effects at the level of horse nested within trainer.

**Results:** Median plasma GGT was 47 U/L (range: 17–142). GGT was above laboratory reference range (45 U/L) in 100/180 samples and in at least one sample from 35/60 horses. Plasma GGT levels were significantly affected by DLR and WLWK (Table 1) but not age, sex, R or WLAM (Figure 1).

**Conclusion:** Preliminary findings indicate further investigation of the effect of workload and performance metrics on plasma GGT levels in Thoroughbred racehorses is warranted.

**Table:** Table 1. Plasma GGT model adjusted marginal means and 95% confidence intervals for days since last race (DLR) and maximum workload during previous week (WLWK)

Parameter	Nr. of horses	Marginal mean plasma GGT (U/L)	95% confidence interval
**DLR**			
1–50	64	51.2(r)	48.06–54.32
51–100	14	54.0	50.92–57.14
101–150	10	66.8*†	60.87–72.70
151–200	12	66.6*	62.98–70.31
201–250	9	60.0*	55.48–64.48
>250	9	54.7*	51.87–57.55
**WLWK**			
None	2	34.4(r)	32.90–35.99
Walk only	2	40.4*†	38.98–41.88
Jog only	2	56.4*†	53.57–59.32
Gallop only	16	46.9*	42.73–51.03
Breeze/race	64	58.8*†	53.54–64.13

* = significantly different (*P* < 0.05) from reference group (r). † = significantly different from prior category (*P* < 0.05).

**Figure:**
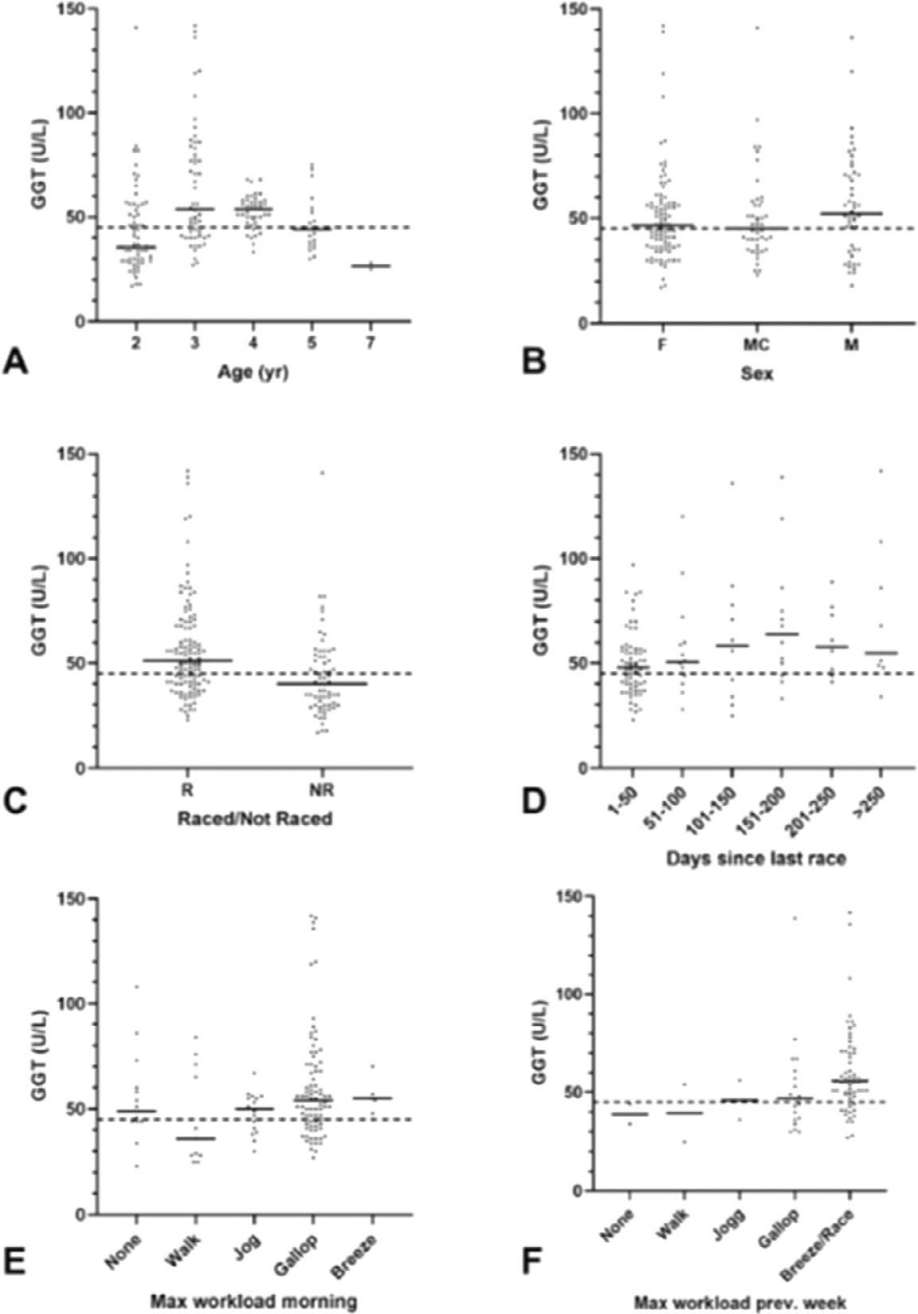
Figure 1. Plasma GGT levels (U/L) by age (A), sex (B), raced vesus not raced (C), days since last race (D), maximum morning workload (E) and maximum workload in the previous week (F). Solid lines represent median values. Dashed lines represent the upper laboratory reference range (45 U/L).

## Abstract E73: Effects of Fluid Resuscitation on Venous Blood Gases and Electrolytes in Critically Ill Neonatal Foals

### **Camilo Jaramillo‐Morales**; Gary Magdesian

#### University of California, Davis, Davis, CA, USA

**Background:** There is a need to evaluate the physiological effects of resuscitative fluids administered to sick neonatal foals admitted to intensive care units.

**Hypothesis/Objectives:** The hypothesis was that administration of isotonic replacement solutions would result in improvements in blood gas parameters in sick neonatal foals. We aimed to compare electrolytes, acid base variables and blood gas results pre‐ and post‐fluid therapy.

**Animals:** 115 neonatal foals.

**Methods:** Retrospective study of sick neonatal foals that received isotonic replacement fluids upon admission to a neonatal ICU. Foals had measurement of venous blood gases and electrolytes before and after initial fluid therapy. Variables were compared using a Wilcoxon signed‐rank test.

**Results:** The study included 115 foals. PlasmaLyte was administered to 92% of the foals. Significant decreases were found in the following analytes after fluid therapy: PCV, pre‐ and post‐fluids (39[22–54] vs. 35[21–51%]; *P* < 0.0001); Total protein pre‐ and post‐fluids (5.2[3.1–11.0] vs. 4.9[2.9–10.6 g/dL]; *P* < 0.0001); Lactate, pre‐ and post‐fluids (3.4[0.8–25] vs. 2.6[0.6–16 mmol/L] *P* < 0.0001); AG, pre‐ and post‐fluids (10.9[−1.4–37.1] vs. 10.5[−3.3–28.4] *P* = 0.002).

The following had significant increases after fluids: Sodium, pre‐ and post‐fluids (137[109–161] vs. 137[112–158 mEq/L] *P* = 0.007); Bicarbonate, pre‐ and post‐fluids (28.0[10.6–34.3] vs. 28.3[11.2–37.7 mEq/L] *P* < 0.0001); Base excess [BE] pre‐ and post‐fluids (2.9[−18.6–9.5] vs. 3.5[−20.7–11.7] *P* < 0.0001); abbreviated strong ion difference (SID), pre‐ and post‐fluids (35[14–53] vs. 36[16–52] *P* = 0.0003); Glucose, pre‐ and post‐fluids (122[4–336] vs. 137[16–438 mg/dL] *P* = 0.0004).

**Conclusions and Clinical Importance:** Markers of hydration (PCV, TP) and metabolic acidosis (BE, bicarbonate, anion gap, SID, and lactate) improved with fluid therapy in ill neonatal foals.

## Abstract E74: Single Dose Pharmacokinetics of Pimobendan and O‐deoxymethylpimobendan (ODMP) in Healthy Adult Horses

### **Catherine A. Jula** ^1^; Jennifer Davis^2^, DVM, MS, PhD, DACVIM, DACVCP; Harold McKenzie^3^, DVM, MS, MSc (VetEd), FHEA, DACVIM

#### ^1^Virginia‐Maryland College of Veterinary Medicine, Blacksburg, VA, USA; ^2^Biomedical Sciences and Pathobiology, Virginia‐Maryland College of Veterinary Medicine, Blacksburg, VA, USA; ^3^Large Animal Clinical Sciences, Virginia‐Maryland College of Veterinary Medicine, Blacksburg, VA, USA

**Background:** Pimobendan is an inodilator approved for treatment of canine cardiac disease. The pharmacokinetics have not been investigated in horses.

**Hypothesis/Objectives:** This study evaluated the pharmacokinetics of oral Vetmedin (V) in horses. Additional objectives were to determine the bioequivalence of compounded pimobendan capsules (C) and suspension (S) and the effects of sample site on plasma drug concentrations.

**Animals:** Six privately‐owned healthy adult horses (5 mares, 1 gelding).

**Methods:** All horses received a single 0.5 mg/kg dose of pimobendan via oral syringe. The initial two horses received C, S, or V using a crossover design with a minimum 1‐week washout period. Samples were collected simultaneously from lateral thoracic and jugular catheters before and after drug administration at predetermined times. Differences between formulation and sample site were analyzed by one‐way ANOVA. The remaining 4 horses received (V) only with jugular samples collected. Analysis was by LC‐MS/MS and noncompartmental pharmacokinetics for (V).

**Results:** No significant differences were noted between formulations or sample site (p>0.05). Concentrations in compounded formulations were 90%(C) and 88%(S) of label. For V, mean (±SD) maximum plasma concentration (*C*_max_) was 4.96 ± 2.13 ng/mL at 2.17 ± 0.98 hr, and area under the curve (AUC0–∞) was 22.1 ± 8.8 hr ng/mL. ODMP was not detected in any sample (<0.07 ng/mL).

**Conclusions and Clinical Importance:** At 0.5 mg/kg PO, pimobendan plasma concentrations were considerably lower than reported in dogs. There was no evidence of oral transmucosal absorption. Pimobendan is poorly absorbed in horses, regardless of formulation, and appears unlikely to have clinical effects.

## Abstract E75: Pharmacokinetics of Repeat Dosing of 40 mg/kg and 60 mg/kg of Acetaminophen in Neonatal Foals

### **Cosette M. Faivre** ^1^; David Wong^2^, DVM, MS, DACVIM, DACVECC; Nicolas Villarino^3^, DVM, PhD, DACVCP; Michael Court^4^, BVSc, PhD, DACVAA; Jenifer Gold^5^, DVM, DACVIM‐LA, DACVECC‐LA

#### ^1^Iowa State University, Ames, IA, USA; ^2^Department Chair, Veterinary Clinical Sciences, Iowa State University, Ames, IA, USA; ^3^Associate Professor, Pharmacology, Washington State University, Pullman, WA, USA; ^4^Professor, Pharmacology & Genomics, Washington State University, Pullman, WA, USA; ^5^Associate, Wisconsin Equine Clinic and Hospital, Summit, WI, USA

**Background:** Acetaminophen is used in infants for antipyresis and analgesia. Neonatal foals might benefit from acetaminophen therapy, but effective and safe dosages for foals are unknown.

**Objectives:** To determine the pharmacokinetics and clinical safety of acetaminophen following oral administration of 40 mg/kg q 12 h or 60 mg/kg q 24 h for 14 days.

**Animals:** Twelve healthy neonatal light‐breed foals.

**Methods:** Foals received acetaminophen orally either at 40 mg/kg q 12 h or 60 mg/kg q 24 h for 14 days. Physical exams were performed daily. Plasma disposition of acetaminophen was determined after first and last drug administration and at the study midpoint. Hematology and serum chemistry analysis was performed prior to the study, at midpoint, and on the last day of administration. Plasma acetaminophen concentrations were determined by high performance liquid chromatography, and plasma pharmacokinetic parameters estimated using non‐compartmental analysis.

**Results:** No statistically significant changes occurred on hematology and biochemistry profiles; elevations in GGT or SDH were seen in 4 foals at various timepoints. *C*_max_ occurred within 2 h for both doses. The 60 mg/kg dose resulted in a larger median *C*_max_ (range) at 28 mg/ml (22–32) than the 40 mg/kg dose at 23 mg/ml (19–27). The median AUC_0–8 h_ (range) was 100 h μg/mL (82–100) at 40 mg/kg and 128 h μg/mL (120–168) for 60 mg/kg. Trough concentrations decreased over time for both regimens.

**Conclusions and Clinical Importance**: Foals tolerate oral acetaminophen at 40 mg/kg q 12 h or 60 mg/kg q 24 h. Further analgesic and antipyretic studies will help to delineate optimal dosage regimens of acetaminophen to treat foals.

**Table:** Table 1. Plasma pharmacokinetic parameters (median [range]) of acetaminophen estimated by noncompartmental analysis in foals after oral administration at 40 mg/kg of body weight every 12 h or 60 mg/kg of body weight every 24 h

PK parameter	Unit	40 mg/kg (*n =* 6)	60 mg/kg (*n =* 6)
*T*_max_	H	0.5 (0.5–1)	1 (0.5–2)
*C*_max_	μg/mL	23 (19–27)	28 (22–32)
AUC_0–4h_	H* μg/mL	64 (56–79)	83 (72–103)
AUC_0–8h_	H* μg/mL	100 (82–120)	128 (120–168)
*C*_max_/dose	N/a	0.58 (0.48–0.55)	0.46 (0.36–0.53)
*C*_Trough—day 1_	μg/mL	3.1 (1.5–6.6)	0.48 (0.2–1)
*C*_Trough—day 7_	μg/mL	1.6 (0.5–3.1)	0.26 (0.05–0.5)
*C*_Trough—day 15_	μg/mL	1.3 (0.4–2.6)	0.4 (0.17–0.6)

AUC_0–4h_ and AUC_0–8h_ = area under the plasma concentration‐time curve from 0, 4 and 8 h after the first drug administration; *C*_max_ = maximum concentration time to maximum concentration after the first administration; *T*_max_ = time to maximum concentration after the first administration. *C*_Trough‐day 1,7_ corresponds to the plasma concentration before the next administration on the days indicated in the table. *C*_Trough‐day 15_ corresponds to 12 and 24 h after the administration for the 40 mg/kg and 60 mg/kg, respectively. N/a = not applicable.

**Figure:**
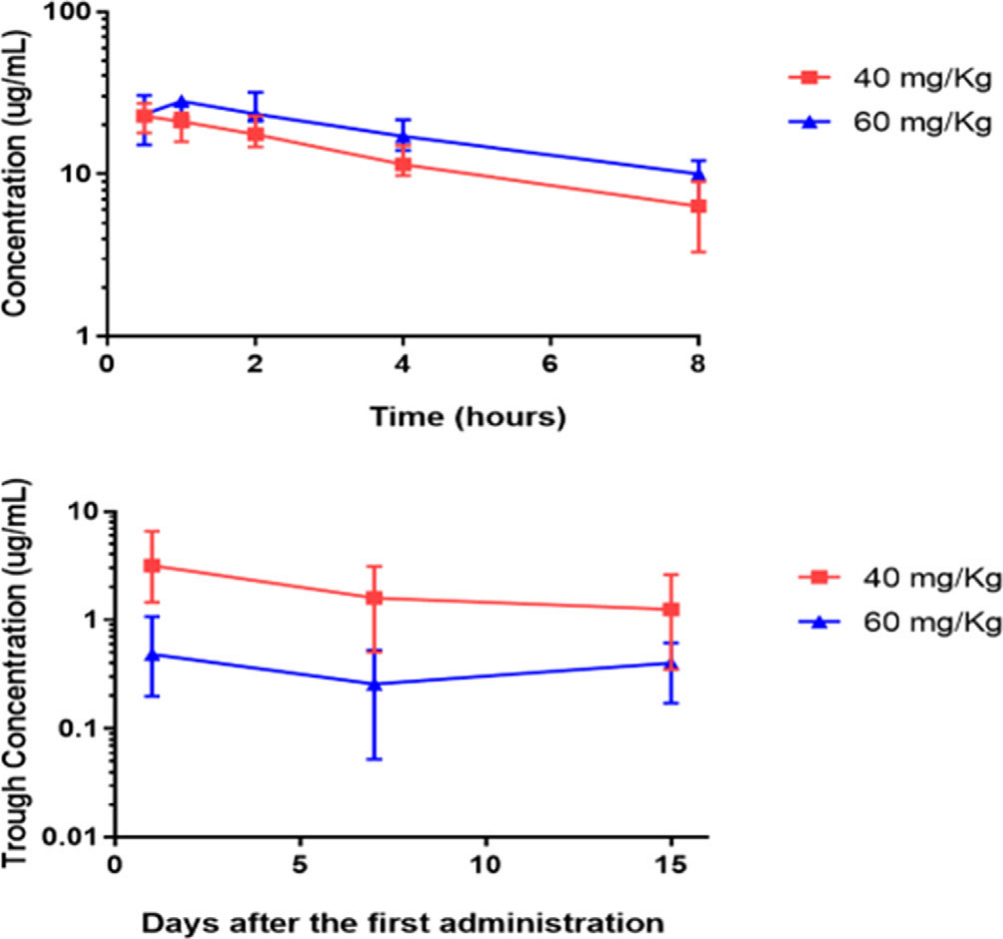
Figure 1. Plasma disposition of acetaminophen in 5–6 days old neonates. Panel A: plasma disposition after the first oral administration at 40 mg/kg (*n =* 6) or 60 mg/kg (*n =* 6). Panel B: Trough plasma concentration of acetaminophen at day 1, 7 and 15 following the administration of 40 mg/kg every 12 h or 60 mg/kg every 24 h.

## Abstract E76: A Preliminary Investigation of the Pharmacokinetics of Esomeprazole After Intravenous and Oral Dosing in Horses

### **Joe Smith** ^1^; Elizabeth Collar^2^, DVM, PhD, DACVS; Rebekah Johnson^2^; Xan Carlson, DVM^2^; Layla Gray^2^; Jessica Gebert^3^, MS; Melissa Hines^2^, DVM, PhD, DACVIM; Joan Bergman^4^, MS; Sherry Cox^5^, MS, PhD

#### ^1^University of Tennessee, Knoxville, TN, USA; ^2^Large Animal Clinical Sciences, University of Tennessee, Knoxville, TN, USA; ^3^College of Veterinary Medicine, Lincoln Memorial University, Harrogate, TN, USA; ^4^Biomedical and Diagnostic Sciences, University of Tennessee, Knoxville, TN, USA; ^5^Professor, Biomedical and Diagnostic Sciences, University of Tennessee, Knoxville, TN, USA

While oral omeprazole is a standard treatment for gastric ulcers in horses, an intravenous formulation is no longer commercially available in some areas. For horses unable to absorb orally administered omeprazole, intravenous esomeprazole, the s‐enantiomer of omeprazole may be an option, but the pharmacokinetics of this drug in horses are currently unknown. Six healthy adult mares were administered a 0.5 mg/kg dose of esomeprazole intravenously and orally in a randomized crossover fashion. After administration, nineteen blood samples were taken over 24 h and repeated with the opposite treatment after a washout period. Samples were analyzed via HPLC for esomeprazole concentrations and time versus concentration data was modeled with standard industry software. The C0, *T*½, and AUC of IV administered esomeprazole was 2030 ng/mL, 24 min, and 660 ng/ml × h respectively. The *C*_max_, *T*½, AUC, and *F* of oral esomeprazole was 104 ng/mL, 40 min, 114 ng/ml × hr and 25%. Clinicians should consider higher oral doses due to low bioavailability, and further research should focus on pharmacodynamic studies evaluating ulcer healing with esomeprazole therapy in horses.

## Abstract E78: Specialized Pro‐Resolving Lipid Mediators Modulate the Effect of Dust Exposure on Airway Neutrophilia in Racehorses

### **Carla J. Olave**; Kathleen Ivester, DVM, PhD, DACVS; Abhijit Mukhopadhyay, PhD; Magdalene Evers; Laurent Couetil, DVM, PhD, DACVIM‐LAIM

#### Purdue University, West Lafayette, IN, USA

**Background:** In horses, the proportion of neutrophils in bronchoalveolar lavage (BAL) is related to dust exposure. Dietary intake of omega‐3 polyunsaturated fatty acids also affects BAL neutrophil proportions, presumably due to changes in the concentrations of specialized pro‐resolving lipid mediators (SPM).

**Hypothesis/Objectives:** Plasma SPM concentrations are related to BAL neutrophil proportions and modulate the relationship between dust exposure and neutrophils in the airway.

**Animals:** Twenty‐five Thoroughbred racehorses housed at the local racetrack.

**Methods:** As part of a feed trial, BAL cytology, dust exposure (PM2.5), and plasma SPM (ELISA) were measured at week‐0 and week‐6. We used univariate generalized linear mixed models (GLMM) to screen for the effect of each SPM on BAL neutrophil proportions. Any SPM identified as significantly related to neutrophil proportions was included in a multivariate GLMM of BAL neutrophil proportions that also included dust exposure and the interaction between SPM and dust exposure as explanatory variables. Significance was set at *P* < 0.05.

**Results:** Plasma resolvin D2 (RvD2) concentration was significantly related to BAL neutrophil proportions (*P* = 0.0496). Multivariate analyses revealed a significant interaction between RvD2 concentration and PM2.5 exposure (*P* = 0.0052). At the mean PM2.5 exposure (0.01 mg/m^3^), a 50 pg/ml increase in the RvD2 concentration resulted in a 15% reduction in the BAL neutrophil proportion.

**Conclusions and Clinical Importance:** In the horse, plasma RvD2 dampens the neutrophilic response of the airway to dust exposure. Diets or supplements that increase plasma RvD2 concentration may be beneficial in the management of neutrophilic airway inflammation.

## Abstract E79: Severe Equine Asthma is Associated With Increased Airway Innervation

### **Laurence Leduc** ^1^; Jean‐Pierre Lavoie^2^, DVM, DACVIM; Mathilde Leclère^3^, DVM, DACVIM, PhD

#### ^1^Université de Montréal, Montreal, QC, Canada; ^2^Full professor, Vice‐Dean, Clinical Studies, Université de Montréal, Montreal, QC, Canada; ^3^Associate Professor, Clinical Studies, Université de Montréal, Montreal, QC, Canada

**Background:** Altered innervation structure and function contributes to airway hyperresponsiveness in humans with asthma. However, the morphology of equine airway innervation and its contribution to airflow limitation in equine asthma remains largely unknown.

**Hypothesis/Objectives:** To characterize peribronchial innervation in horses with asthma. We hypothesized that peribronchial nerve density increases in horses with asthma compared to controls.

**Animals:** Formalin‐fixed lung samples from eight horses with severe asthma (five in exacerbation, three in remission) and eight controls. Lung function had been recorded prior to sample collection.

**methos:** Blinded case‐control study. Immunohistochemistry was performed using rabbit anti‐s100 antibody as a neuronal marker for myelinating and non‐myelinating Schwann cells. Using histomorphometry, the number and the area of nerves in the peribronchial space and in proximity to airway smooth muscle were recorded and corrected for airway size.

**Results:** Both the number and the area of peripheral nerves were increased in the peribronchial region of horses with asthma (median (range): 1.87 × 10^−5^ nerves/μm^2^ (6.25 × 10^−6^ to 6.29 × 10^−5^) and 1.03 × 10^−3^ nerve area/μm2 (5.40 × 10^−4^ to 2.38 × 10^−3^)) compared to controls (5.17 × 10^−6^ nerves/μm^2^ (2.37 × 10^−6^ to 1.94 × 10^−5^) and 4.14 × 10^−4^ nerve area/μm^2^ (1.08 × 10^−4^ to 1.61 × 10^−3^)) (Mann‐Whitney, *P* < 0.01). The number of nerves within or surrounding airway smooth muscle was significantly increased in horses with asthma (4.47 × 10^−6^ nerves/uum2 (2.10 × 10^−6^ to 2.73 × 10^−5^)) compared to controls (2.26 × 10^−6^ nerves/μm2 (4.60 × 10^−7^ to 1.07 × 10^−5^)) (Mann‐Whitney, *P* < 0.05). Airway innervation in horses in exacerbation was not correlated with resistance or elastance (Spearman's correlation: *r* < 0.35, *P* > 0.50).

**Conclusions and Clinical Importance:** Equine asthma is associated with increased airway innervation, which may contribute to airway smooth muscle remodeling and asthma severity.

## Abstract E80: Fish Oil Supplementation Reduces Airway Neutrophilia in Thoroughbred Racehorses

### **Laurent Couetil** ^1^; Kathleen Ivester^2^, DVM, PhD, DACVS; Laura Murray^3^, RVT, LATG; Ryan Carpenter^4^, DVM, MS, DACVS; Wade Byrd^4^, DVM; Scott Hay^5^, DVM; Clayton McCook^6^, DVM, DACVIM(LA); John Burgess^7^, PhD; Thomas Peters^8^, PhD; Jae Hong Park^9^, PhD, CIH

#### ^1^College of Veterinary Medicine, Purdue University, West Lafayette, IN, USA; ^2^Research Scientist, Veterinary Clinical Scientist, Purdue University, West Lafayette, IN, USA; ^3^Research Technologist, Veterinary Clinical Sciences, Purdue University; ^4^Equine Medical Center, Cypress, CA, USA; ^5^Teigland, Franklin and Brokken, Fort Lauderdale, FL, USA; ^6^Equine Sports Medicine and Surgery, Weatherford, TX, USA; ^7^Associate Professor, Nutrition Science, Purdue University, West Lafayette, IN, USA; ^8^Professor, Occupational and Environmental Health, University of Iowa, Iowa City, IA, USA; ^9^Assistant Professor, Occupational Health and Environmental Health Sciences, Purdue University, West Lafayette, IN, USA

**Background:** When combined with low dust diets, omega‐3 polyunsaturated fatty acids (ω‐3) supplementation can reduce neutrophil proportions in the bronchoalveolar lavage fluid (BAL) of horses with asthma and healthy horses. It is unknown if ω‐3 supplementation is effective without concurrent dust mitigation.

**Hypothesis/Objectives:** Dietary supplementation with fish oil rich in ω‐3 will lower the proportion of neutrophils in BAL of horses fed a conventional diet including hay.

**Animals:** Clinically healthy racing Thoroughbreds (55) housed at 4 US racetracks.

**Methods:** In this double‐masked controlled trial, horses were randomly assigned to receive daily fish oil (50 mL) or placebo (corn oil) for 4 weeks. Exposures of particulate matter smaller than 10 μm (PM10) were measured and BAL was collected at baseline and after 4 weeks of supplementation. The effects of supplementation on BAL neutrophil proportions and the relationship with PM10 exposure were evaluated using a generalized linear model. Significance was set at *P* < 0.05.

**Results:** BAL neutrophils decreased from 7.8 ± 0.01% to 5.3 ± 0.01% (mean ± s.e.m.) in horses given fish oil compared to placebo (5.9 ± 0.01% to 9.3 ± 0.02%; *P* = 0.029). After 4 weeks of fish oil supplementation, PM10 exposure was not related to BAL neutrophils (*P* = 0.2) but after placebo supplementation, PM10 exposure was related to BAL neutrophils (*P* < 0.0001), with a 10 μg/m^3^ increase in PM10 from the mean exposure resulting in a 1.3‐fold increase in BAL neutrophils.

**Conclusions and Clinical Importance:** Supplementation with fish oil reduces BAL neutrophil proportions in clinically healthy racehorses independent of dust exposure. Increasing ω‐3 intake might help mitigate neutrophilic asthma in horses.

## Abstract E81: Bronchoalveolar Lavage Hemosiderosis in Lightly Active or Sedentary Horses

### **Ananya Mahalingam‐Dhingra**; Daniela Bedenice, DrMedVet, DACVIM (LA), DACVECC (Eq); Melissa Mazan, DVM, DACVIM (LAIM)

#### Cummings School of Veterinary Medicine, Tufts University, North Grafton, MA, USA

**Background:** Hemosiderophages in bronchoalveolar (BAL) cytology are commonly ascribed to exercise‐induced pulmonary hemorrhage (EIPH); little information exists regarding the presence of these cells in horses that perform light or no work and referred for respiratory evaluation.

**Objectives:** To evaluate the presence of and predictors for hemosiderophages in BAL cytology of horses suspected of respiratory disease without history of or risk factors for EIPH.

**Animals:** BAL cytologies of 353 horses evaluated for respiratory disease between 2012 and 2022 at a referral center were retrospectively reviewed.

**Methods:** This was an observational retrospective cross‐sectional study. Horses with a history or likelihood of past strenuous exercise were removed; the remaining 91 were classified as hemosiderin‐positive (HSD‐POS) or hemosiderin‐negative (HSD‐NEG) based on Prussian Blue staining. Potential predictors of the presence of hemosiderophages (history, clinical evaluation, baseline lung function, airway reactivity, BAL cytology and hemosiderin score) were compared between the two groups, using univariate and multivariate analyses.

**Results:** Horses with a diagnosis of severe equine asthma were significantly more likely to be HSD‐POS than horses with mild‐moderate equine asthma (odds ratio 11.1 (95% confidence interval [3.2–38.5]), *P* < 0.001).

**Conclusions and Clinical Importance:** Hemosiderophages were found in the BAL cytology in a subset of horses that perform light or no work and presenting for respiratory evaluation. The link between hemosiderophages and severe equine asthma may help elucidate previously unstudied pathology associated with equine asthma.

## Abstract E82: Initial Investigation of Bronchoalveolar Lavage Fluid Metabolomics in Healthy and Suspect Asthmatic Horses

### **Jane S. Woodrow** ^1^; Christopher Petucci^2^; Melissa Hines^3^; Carla Sommardahl^3^; Bente Flatland^4^; Elizabeth Lennon^5^

#### ^1^New Bolton Center, University of Pennsylvania, Kennett Square, PA, USA; ^2^Metabolomics Core, Cardiovascular Institute, Perelman School of Medicine, University of Pennsylvania, Philadelphia, PA, USA; ^3^Department of Large Animal Clinical Sciences, College of Veterinary Medicine, University of Tennessee, Knoxville, TN, USA; ^4^Department of Biomedical & Diagnostic Sciences, College of Veterinary Medicine, University of Tennessee; ^5^Department of Clinical Sciences and Advanced Medicine, College of Veterinary Medicine, University of Pennsylvania, Kennett Square, PA, USA

**Background:** Metabolomics studies have been performed in limited studies of horses with asthma with varying results. In this study, mass spectrometry‐based metabolomics was used to profile BALF from horses to identify metabolites related to the pathogenesis of this disease.

**Hypothesis/Objectives:** We hypothesized that horses with asthma would have significant differences in metabolic profiles of BALF supernatant versus healthy. Our objective was to utilize untargeted LC/MS metabolomics to identify biomarkers in suspect asthmatic horses.

**Animals:** Institution‐owned, and client‐owned horses were enrolled. Five healthy and 5 suspect asthma horses were included.

**Methods:** BALF was collected from horses with suspect asthma during active disease and supernatant stored at −80°C. Metabolites were extracted from BALF in ice cold methanol and separated using reversed‐phase C18 and HILIC chromatography coupled to a Thermo Vanquish UHPLC/Orbitrap ID‐X mass spectrometer. Compound Discoverer (Thermo Scientific) was used to process raw mass spectrometry data and perform a metabolite database search. Metabolites having a Welch's *t*‐test *P* value <0.05 with Benjamini‐Hochberg correction were considered. Post‐processing involved generation of principal component analysis (PCA) plot, volcano plot, box‐and‐whisker plots, and heat maps.

**Results:** Several metabolites were identified in BALF including some that were important for group clustering and separation. Two metabolites identified that are potentially interesting in relation to airway disease are N‐acetylserotonin and N‐tetradecanoylsphinganine.

**Conclusions and Clinical Importance:** Identification of potential significant metabolites in horse BALF was possible utilizing LS/MS metabolomics, which may improve our understanding of equine asthma pathophysiology in follow‐up studies.

## Abstract F01: Congestive Heart Failure in Goats: A Retrospective Study

### **Megan Palmisano** ^1^; JoAnn Slack^2^, DVM, DACVIM (LAIM), MS; Claire Underwood^2^, VetMB, PhD, MA

#### ^1^University of Pennsylvania New Bolton Center, Kennett Square, PA, USA; ^2^Diagnostic Imaging, University of Pennsylvania New Bolton Center, Kennett Square, PA, USA

**Background:** Congestive heart failure (CHF) in goats is poorly described but increasingly diagnosed as pet goats become more popular. More information on caprine cardiac disease is warranted.

**Hypothesis/Objectives:** This retrospective study aimed to describe presentation, clinical findings, treatment, and outcome of goats diagnosed with CHF.

**Animals:** Medical records of goats admitted to a tertiary referral center from 2008–2022 were reviewed.

**Methods:** Goats were included if diagnosed with CHF. Clinical findings, diagnosis, treatment, and survival were recorded. Fisher's exact tests were performed to assess for association between clinical findings and survival.

**Results:** Twelve cases met inclusion criteria, 58% presented for anorexia/weight loss. Findings included tachycardia (92%), effusion (92%), elevated GGT (92%), low or inappropriately normal fibrinogen given disease severity (58%), tachypnea (58%), a murmur (25%), arrhythmia (17%), edema (25%), cough (25%), and jugular pulses (8%). There was no association between these findings and survival (*P* ≥ 0.05). All 12 were diagnosed with right‐sided CHF, 9 had primary cardiac disease. 33% were euthanized shortly after presentation, 33% died or were euthanized after some treatment and 33% survived. Cardiac medications included beta blockers (*n =* 2), digoxin (*n =* 1), benazepril (*n =* 1), and furosemide (*n =* 4). One goat received transcutaneous electrical cardioversion (TCEC) of atrial fibrillation. Sixty percent of the goats that received cardiac targeted treatment survived to discharge.

**Conclusions:** CHF should be considered in goats presenting with weight loss, tachycardia, effusions, and biochemical values consistent with hepatic dysfunction. Although not previously reported TCEC and medical management are possible. Further research is needed to better understand CHF in goats.

## Abstract F02: Abdominal Emergencies in Dairy Cattle Admitted to a Referral Hospital: A Retrospective Study

### **Maria Sara Santos**; Marie Babkine; André Desrochers; Vincent Doré; Gilles Fecteau; David Francoz; Sylvain Nichols

#### University of Montreal, Montréal, QC, Canada

**Background:** Conditions that present as abdominal emergencies in dairy cattle are frequent. Decisions regarding diagnostic and treatment are challenging. Prognosis remains difficult to estimate.

**Hypothesis/Objectives:** Describe the clinical presentation and outcome of abdominal emergencies in dairy cows admitted to a referral hospital.

**Methods:** Retrospective study. Review of medical records of dairy cows admitted between 2016 and 2021 for an abdominal emergency. Definition included: decreased or absent fecal production and/or visceral or parietal pain and duration of less than 7 days. Data regarding history, physical examination, blood work, treatment, diagnosis, and outcome were studied. Descriptive statistics were performed.

**Results:** Two hundred and thirty‐seven cows were enrolled. Fifty‐eight percent (129/222) were in the first 3 months of lactation. The most common clinical signs were dehydration (65%, 155/237), decrease or absence of feces (62%, 146/237), tachycardia (54%, 128/237), and parietal pain (41%, 96/237). A median lactate of 4.3 mmol/L, chloride of 95 mmol/L, and ionized calcium of 1.06 mmol/L were present at admission. Of the cows that were treated, 53% (125/226) underwent surgery. Final diagnosis consisted of small intestinal disorders (32%, 76/237), peritonitis (27%, 63/237), abomasal disorders (25%, 60/237), large intestinal disorders (5%, 13/237), and others (10%, 25/237). Sixty‐nine percent of the admitted cattle were discharged from the hospital, 45% and 40% were alive after 2 and 6 months, respectively.

**Conclusions and Clinical Importance:** The majority of cows were in early lactation. Abdominal pain was predominantly parietal. About half of the cases were treated only medically. The overall prognosis was fair.

## Abstract F03: Effect of Sample Collection Time on Fecal Microbiota Composition in Diarrheic Calves

### **Lynna Li** ^1^; David Renaud^2^, DVM, PhD; Hanna Goetz^3^, DVM, PhD; Emma Jessop^4^, BSc; Marcio Costa^5^, DVM, MSc, DVSc, PhD; Lisa Gamsjaeger^6^, DVM, PhD, DACVIM; Diego Gomez^7^, DVM, MSc, MVSc, PhD, DACVIM

#### ^1^Cornell University, Ithaca, NY, USA; ^2^Associated Professor, Population Medicine, University of Guelph, Guelph, ON, Canada; ^3^PhD student, Population Medicine, University of Guelph, Guelph, ON, Canada; ^4^MSc Student, Clinical Studies, University of Guelph, Guelph, ON, Canada; ^5^Assistant Professor, Universite de Montreal, Montreal, QC, Canada; ^6^Assistant Professor, Population Health and Pathobiology, North Carolina State University, Raleigh, NC, USA; ^7^Assistant Professor, Clinical Studies, University of Guelph, Guelph, ON, Canada

**Background:** Fecal samples for microbiota studies in calves are usually collected on a weekly basis. This approach can result in collection of samples from diarrheic calves without considering the time of diarrhea onset.

**Objective:** Compare the fecal microbiota of healthy calves, calves with diarrhea onset on the day of sampling (*D* < 24 h), and calves having diarrhea for >24 to 48 h (*D* 24–48 h).

**Animals:** 20 healthy, 20 *D* < 24 h, and 11 D 24–48 h veal calves of 3–7 days of age.

**Methods:** Case control study. Calves with loose or watery feces were considered as diarrheic. Fecal samples were collected on day 7 after arrival on farm. Fecal microbiota was characterized by sequencing of 16S ribosomal RNA gene amplicons.

**Results:** The fecal community membership (Jaccard index) and structure (Yue & Clayton index) of healthy calves were significantly different than those of diarrheic calves (AMOVA, *P* < 0.001 for both comparisons). Both the bacterial membership and structure differed significantly between *D* < 24 h and *D* 24–48 h calves (AMOVA, *P* < 0.001 for both comparisons). Phocaeicola, Bacteroides, Prevotella, Faecalibacterium, Butyricicoccus, Ruminococcaceae, and Lachnospiraceae were enriched in healthy calves. Enterococcus, Ligilactobacillus, Lactobacillus, and Gallibacterium were enriched in the feces of D<24 h calves, while Enterococcus, Streptococcus, Clostridium sensu stricto and Escherichia/Shigella were enriched in the *D* 24–48 h calves.

**Conclusions:** Rapid changes in fecal microbiota occur during the first 48 h of diarrhea. The time from diarrhea onset to sampling affect the bacterial composition of feces of diarrheic calves. Researchers should standardize times for fecal collection based on the time of diarrhea onset rather than convenient times.

## Abstract F04: Effects of Plasma and Hetastarch on Colloid Oncotic Pressure and Coagulation Parameters in Dairy Calves

### **Sarah W. Cuneo** ^1^, DVM; Munashe Chigerwe^2^, BVSc, MPH, PhD, DACVIM (LAIM)

#### ^1^Veterinary Medical Teaching Hospital, University of California Davis, Davis, CA, USA; ^2^Professor, Department of Medicine and Epidemiology, School of Veterinary Medicine, University of California Davis, Davis, CA, USA

**Background:** Transfusion with fresh frozen plasma (FFP) or hetastarch 6% (HES) are indicated for management of decreased colloid oncotic pressure (COP) in hypoproteinemic calves. However, HES has been associated with coagulopathies in other species. The effectiveness of HES compared to FFP has not been extensively studied in calves.

**hypotheses:** Administration of HES increases COP to levels comparable to FFP. The administration of HES does not alter coagulation parameters.

**Animals:** 7 dairy bull calves from a university teaching herd.

**Methods:** Cohort, clinical trial in 2‐way crossover design, with a 2‐week washout period. HES and FFP were administered intravenously at 10 and 20 mL/kg, respectively, once. Serum COP was measured pre‐transfusion, and at 0, 1, 2, 4, 6, 12, 24, 36, and 72 h and 7 days after each transfusion. Coagulation parameters including prothrombin time, partial thromboplastin time, fibrinogen, fibrinogen degradation products and thrombocytes analysis were performed pre‐transfusion, and at 0, 24, and 72 h after transfusion. Effect of treatment and time on COP and coagulation parameters was determined by MANOVA.

**Results:** Transfusion with HES or FFP was positively associated with serum COP. Time did not have an effect on COP. Administration of FFP or HES did not significantly change (*P* > 0.05) the coagulation parameters.

**Conclusions and Clinical Importance:** HES is an effective alternative to FFP transfusion in calves, and it does not cause significant changes in coagulation parameters.

## Abstract F05: Fucoidan Increases Porcine Neutrophil Extracellular Trap Formation Through Tumor Necrosis Factor‐Alpha

### **Hakhyun Kim** ^1^; Yoonhoi Koo^2^; Changwoo Nahm^2^; Taesik Yun^2^; Hakhyun Kim^2^; Byeong‐Teck Kang^2^; Mhan‐Pyo Yang^2^

#### ^1^College of Veterinary Medicine, Chungbuk National University, Cheongju, South Korea; ^2^Laboratory of Veterinary Internal Medicine, College of Veterinary Medicine, Chungbuk National University, Cheongju, South Korea

**Background:** Fucoidan which is sulfated polysaccharide extracted from brown seaweed has a wide variety of biological activities. Neutrophil extracellular traps (NETs) formation is an immune response for the invasion of microbes. Neutrophils release granule protein and chromatin that together form extracellular fibers that bind pathogens.

**Hypothesis/Objectives:** To examine the effect of fucoidan on NET formation of porcine peripheral blood polymorphonuclear cells (PMNs).

**Animals:** Clinically healthy 6‐month‐old pigs were included as donors for peripheral blood mononuclear cells (PBMCs).

**Methods:** The NET formation was measured by fluorescence microplate reader. The production of tumor necrosis factor (TNF)‐α in the culture supernatants from peripheral blood mononuclear cells (PBMCs) was measured by ELISA method.

**Results:** Fucoidan itself did not cause any direct effect on NET formation. However, NET formation of PMNs were increased by the culture supernatant from PBMCs treated with fucoidan. The NET formation of PMNs were also enhanced by treatment with recombinant porcine (rp) TNF‐α. The ability of the culture supernatant from fucoidan treated PBMCs to stimulate the NET formation on porcine PMNs was inhibited by addition of goat anti‐rp TNF‐α polyclonal antibody (pAb; IgG) prior to the culture. NET formation of rp TNF‐α was also suppressed by goat anti‐rp TNF‐α pAb. The level of TNF‐α in culture supernatant from PBMCs was increased by treatment with fucoidan.

**Conclusion and Clinical Importance:** These results suggested that fucoidan has an immunoenhancing effect on the NET formation of porcine PMNs, which is mediated by TNF‐α released from fucoidan‐treated PBMCs.

## Abstract F07: Bovine Viral Diarrhea Virus Seroprevalence in Wild Pigs Across 17 States

### **Shari M. Kennedy** ^1^; Thomas Passler^2^, DVM, PhD, DACVIM; Stephen Ditchkoff^3^, BS, PhD; Vienna Brown^4^, MPH, PhD; Gage Raithel^5^; Manuel Chamorro^6^, DVM, MS, PhD, DACVIM; Paul Walz^7^, DVM, MS, PhD, DACVIM; Shollie Falkenberg^8^, BS, MS, PhD; Constantinos Kyriakis^9^, DVM, PhD

#### ^1^Large Animal Teaching Hospital, Auburn University, Auburn, AL, USA; ^2^Professor, Clinical Sciences, College of Veterinary Medicine, Auburn University, Auburn, AL, USA; ^3^Ireland Professor, Forestry and Wildlife Sciences, Auburn University, Auburn, AL, USA; ^4^Biologist, National Feral Swine Damage Management Program, USDA/APHIS/Wildlife Services; ^5^Lab Tech III, Animal Health and Disease Research, College of Veterinary Medicine, Auburn University, Auburn, AL, USA; ^6^Associate Professor, Clinical Sciences, College of Veterinary Medicine, Auburn University, Auburn, AL, USA; ^7^Professor and Head, Pathobiology, College of Veterinary Medicine, Auburn University, Auburn, AL, USA; ^8^Associate Professor, Coordinator, Pathobiology, College of Veterinary Medicine, Auburn University, Auburn, AL, USA; ^9^Assistant Professor, Pathobiology, College of Veterinary Medicine, Auburn University, Auburn, AL, USA

**Background:** Wild pigs are a reservoir for numerous infectious diseases, many of which are zoonotic or of concern to other species. Bovine viral diarrhea virus (BVDV) represents a major disease of economic concern for the livestock industry. Infections in swine typically do not result overt disease but may spill‐back into cattle and other ruminants.

Hypothesis/**Objective:** The first objective was to determine the seroprevalence of BVDV in wild pigs by comparative serology. The second objective was to determine if state, age category, or sex significantly affected serum neutralization titers.

**Animals:** A total of 1129 serum samples from wild pigs were collected across 17 states.

**Methods:** The samples underwent an initial virus neutralization (VN) assay at a dilution of 1:2 to screen for the presence of neutralizing antibodies or cytotoxicity. For positive samples, serial 2‐fold dilutions starting at 1:2 with a final dilution of 1:65 536 was performed to determine end point titers. All samples underwent comparative serology to both BVDV‐1b strain TGAC and BVDV‐2a strain 125‐c.

**Results:** Sixty‐seven samples were excluded from analysis due to cytotoxicity. Thirty‐nine samples were positive to BVDV‐1b. Thirty‐two samples were positive to BVDV‐2a. Overall prevalence for BVDV‐1b was 4.4% and 3.6% for BVDV‐2a, respectively. The prevalence among the different states varied from 0% to 16.7%.

**Conclusions and Clinical Importance:** The seroprevalence was determined to be similar to previously reported results for wild pigs in other countries. Age category and sex did not significantly affect serum neutralization titers, nor were risk factors for having a positive antibody titer.

**Table:** Table 1. BVDV positive samples and titers.

	No. samples positive	Highest titers
BVDV‐1b total	39	8192
BVDV‐1b only	19	1024
BVDV‐2a total	32	64
BVDV‐2a only	19	16
Both <4× difference (1b:2a)	11	128:64
Both >4× difference (1b:2a)	9	8192:64

Total and individual number of BVDV‐1b and BVDV‐2a positive samples and the highest titers detected. Samples positive to both BVDV‐1b and BVDV‐2a were divided between less than or greater than four‐fold differences between the titers to demonstrate potential cross‐reactivity between the species, dual infections, or lack of specificity indicating a potential

**Table:** Table 2. Individual and total seroprevalence across 17 states

State	BVDV‐1b (no. positive samples)	Prevalence BVDV‐1b (%)	BVDV‐2a (No. positive samples)	Prevalence BVDV‐2a (%)
Alabama	1	1.05	2	2.11
Arkansas	2	5.0	0	0
California	5	11.63	7	16.28
Florida	4	5.88	5	7.35
Georgia	2	3.13	4	6.25
Iowa	1	16.67	0	0
Kansas	0	0.0	0	0
Kentucky	0	0.0	0	0
Louisiana	1	1.52	0	0
Missouri	2	4.0	1	2.0
Mississippi	1	1.37	1	1.37
North Carolina	1	4.55	0	0
Oklahoma	5	9.8	4	7.84
Oregon	0	0	0	0
South Carolina	0	0	0	0
Tennessee	1	1.92	0	0
Texas	13	7.93	8	4.88
Total	39	4.44	32	3.64

Apparent prevalence of positive neutralizing antibody titers across states for BVDV‐1b and BVDV‐2a.

## Abstract F08: Comparison of Bacteremia Detection and Contamination Between Two Blood Culture Media in Critically Ill Calves

### **Mathilde L. Pas** ^1^; Filip Boyen^2^, PhD; Donatienne Castelain^3^, DVM; Bart Pardon^4^, DECBHM, PhD; Jade Bokma^5^, PhD

#### ^1^Ghent University, Ghent, Belgium; ^2^Doctor, Department of Pathobiology, Pharmacology and Zoological Medicine, Ghent University, Ghent, Belgium; ^3^Department of Internal Medicine, Reproduction and Population Medicine, Ghent University, Ghent, Belgium; ^4^Professor, Department of Internal Medicine, Reproduction and Population Medicine, Ghent University, Ghent, Belgium; ^5^Post‐Doc, Department of Internal Medicine, Reproduction and Population Medicine, Ghent University, Ghent, Belgium

**Background:** Sepsis is a life‐threatening condition for which critically important antimicrobials are often indicated. The value of blood culture for sepsis is indisputable, but appropriate sampling guidelines are lacking in calves.

**Objectives:** To compare diagnostic accuracy and time to positivity (TTP) of two blood culture media (pediatric plus [PP] and plus aerobic [PA]), with a different volume (3 vs. 10 mL) and composition. To estimate blood culture contamination in calves.

**Animals:** 126 critically ill calves.

**Methods:** Diagnostic test study in which performance of PP, PA, and hypoglycemia (<60 mg/dL) was assessed to detect sepsis using a Bayesian latent class model. Survival analysis was used to compare TTP. Contamination was descriptively analyzed. Pathogens were considered relevant when: (1) member of the Enterobacteriaceae family, (2) highly relevant in other bovine pathologies, or (3) isolated from both blood cultures.

**Results:** The sensitivities for PP, PA, and hypoglycemia were 68.7% (95% credibility interval = 30.5%–93.7%), 87.5% (47.0%–99.5%) and 61.3% (49.7%–72.4%), respectively. Specificity was estimated at 95.1% (82.2%–99.7%), 94.2% (80.7%–99.7%) and 72.4% (64.6%–79.6%). No significant difference in TTP was identified between PP and PA. An overall contamination rate of 8.3% (21/252) was assumed, and blood culture positivity reduced (40.5% to 22.2%) when presumed contaminants were excluded.

**Conclusions and Clinical Importance:** The PA culture seems to have the highest diagnostic test accuracy compared to PP and hypoglycemia, and the type of culture did not influence TTP or the contamination rate. Therefore, sampling PA culture twice is theoretically the best option to determine sepsis in critically ill calves.

## Abstract F09: D,L Methionine and Ammonium Chloride Effect on Urine pH and Excretion of Calcium in Goats

### **Casey E. Neal** ^1^; Gretchen Grissett^2^, MS, DACVIM (LAIM); Amelia Woolums^2^, MS, PhD, DACVIM (LAIM); Sherrill Fleming^1^, DACVIM (LAIM); Robert Wills^3^, MS, PhD, DACVPM

#### ^1^Mississippi State University, Mississippi State, MS, USA; ^2^Assistant Clinical Professor, Pathobiology and Population Medicine, Mississippi State University, Mississippi State, MS, USA; ^3^Professor, Comparative Biomedical Sciences, Mississippi State University, Mississippi State, MS, USA

**Background:** Urinary acidification with ammonium chloride (NH_4_Cl) for urolith dissolution is a common treatment for goats with urolithiasis. Studies have reported increased fractional excretion of calcium (FECa) following NH_4_Cl administration, which could increase calcium‐based urolithiasis. D,L methionine may result in similar acidification with less calcium excretion.

**Objectives:** To compare effects of orally administered D,L methionine and NH4Cl on urine and blood pH, FECa, and blood HCO3− concentrations in male goats.

**Animals:** Twelve healthy 4–6‐month‐old Boer‐cross wethers.

**Methods:** Prospective, randomized, cross over study. Wethers were administered 200 mg/kg of NH_4_Cl or D,L methionine orally for 14 days with a 7 day washout period between trials. Venous blood and urine samples were collected every 2 days. The effects of treatment and treatment day on urine and blood pH, FECa, and HCO3‐ was assessed using linear mixed models.

**Results:** In models with treatment and day, mean urine pH on day 6 (*μ* = 7.49, *P* < 0.001), 8 (*μ* = 7.78, *P* = 0.008, and 10 (*μ* = 7.53, *P* < 0.001) was significantly lower than on day 0 (*μ* = 8.23). A significant treatment effect was not detected on urine pH (*P* = 0.1158), blood pH (*P* = 0.7683), FECa (*P* = 0.4038), and blood HCO3‐ (*P* = 0.1422).

**Conclusion and Clinical Importance:** Urine pH was decreased on days 6, 8, and 10, however, values were not low enough to dissolve uroliths. Under the conditions of this study, there was no difference in FECa between treatments. Further studies should investigate effects of higher doses of these drugs.

## Abstract F10: Validation and Comparison of SDMA in Healthy and Obstructed Goats

### **Blanca Camacho** ^1^, DVM; Jennifer Halleran^2^, DVM, PhD, DACVIM (LAIM); Derek Foster^3^, DVM, PhD, DACVIM (LAIM); Siena Mitman^4^, DVM

#### ^1^Food Animal Medicine Resident, North Carolina State University, Raleigh, NC, USA; ^2^Assistant Professor, North Carolina State University, Raleigh, NC, USA; ^3^Assistant Professor, Ruminant Medicine, North Carolina State University, Raleigh, NC, USA; ^4^Food Animal Intern, North Carolina State University, Raleigh, NC, USA

**Background:** Obstructive urolithiasis (OU) is a common small ruminant emergency and can lead to hydronephrosis and intrinsic renal disease. As post‐renal azotemia is common in goats with OU, diagnosis of intrinsic renal disease prior to surgical correction of OU is challenging. Symmetric dimethylarginine (SDMA) is a renal biomarker correlated with early detection of renal disease. SDMA has not been validated in goats.

**Hypothesis/Objectives:** The objectives of this study are to establish reference ranges for SDMA in healthy goats and describe changes in SDMA in goats with OU.

**Animals:** Healthy goats, both male and female, from a research herd (*n =* 59). Goats with clinical OU (*n =* 12).

**Methods:** Healthy goats had blood collected at a single time point. Clinically affected goats had blood collected at presentation, 24, 48 and 72 h post‐surgery for SDMA and biochemistry analysis. Urine was collected for urinalysis at presentation. Descriptive statistics will be used to assess values in both the healthy and obstructed goat populations.

**Results:** For the healthy group, the mean SDMA value was 17.6 μg/dL, median SDMA value of 17 μg/dL and a range of 7 to 43 μg/dL. Currently, eight goats with obstructive urolithiasis have samples collected. Analysis will be conducted once the sample size has been achieved.

**Conclusions and Clinical Importance:** SDMA has the potential to be used in clinical cases of caprine OU to provide prognostic information prior to surgery.

## Abstract F11: Prognostic Indicators of Outcome for Non‐ambulatory Beef Cattle Presented to Two Referral Hospitals

### **Diana M. Perez‐Solano** ^1^; Emily Reppert^2^, DVM, MS, DACVIM (LAIM); Jenna Stockler^3^, DVM, DACVIM (LAIM); Thomas Passler^3^, DVM, PhD, DACVIM (LAIM); Laura Huber^4^, DMV, MSc, PhD, DACVPM; Jenna Bayne^3^, DVM, PhD, DACVIM (LAIM); Manuel Chamorro^3^, DVM, MS, PhD, DACVIM (LAIM)

#### ^1^College of Veterinary Medicine, Auburn University, Auburn, AL, USA; ^2^College of Veterinary Medicine, Kansas State University, Manhattan, KS, USA; ^3^Department of Clinical Sciences, College of Veterinary Medicine, Auburn University, Auburn, AL, USA; ^4^Department of Pathobiology, College of Veterinary Medicine, Auburn University, Auburn, AL, USA

**Background:** Diagnosis and treatment of non‐ambulatory beef cattle are challenging, and scientific reports of prognostic indicators for survival in downer beef cattle are unavailable.

**Hypothesis/Objectives:** To evaluate records of downer adult (≥2 years of age) beef cattle presented to two referral hospitals to identify prognostic indicators for survival to discharge.

**Animals:** Sixty‐three recumbent beef cattle.

**Methods:** Retrospective case study. Medical records of beef cattle presented to two referral hospitals for recumbency (inability to stand) between 2010 and 2022 were analyzed. Univariable and multivariable logistic regression analyses were performed to evaluate the associations of predictors with survival to discharge.

**Results:** Of 63 cattle included, 51 (81%) died or were euthanized and 12 (19%) survived to discharge. Cattle not receiving float tank therapy (OR = 4.11), those with neutrophilia (OR *=* 10.67) or with hyperglobulinemia (OR *=* 2.77) at presentation had lower odds of survival to discharge. Cattle diagnosed with musculoskeletal disease including spinal/vertebral disease had lower odds (OR = 5.33) of survival to discharge compared to cattle diagnosed with peripheral nerve paralysis.

**Conclusions:** Recumbency is associated with a poor prognosis for survival to discharge in beef cattle. Float tank therapy may improve survival to discharge in some cases.

## Abstract N01: Degradation of Midazolam over Time in a Simulated Home Environment

### **Christopher Adams**; Michael Reese, DVM, DACVIM (Neurology)

#### Southeast Veterinary Neurology of Boynton Beach, Boynton Beach, FL, USA

**Background:** Midazolam is frequently used for management of status epilepticus or cluster seizures at home. There are currently no studies looking at the stability of midazolam in the home environment.

**Objective:** The aim of this prospective study was to evaluate the degradation of midazolam over the course of 360 days in a simulated home environment using three different storage methods (light‐protected glass vial [group A], light‐protected syringe [group B] and non‐light‐protected syringe [group C]).

**Methods:** The concentration of midazolam was determined using high‐performance liquid chromatography (HPLC) in triplicate, at each of the time points (0, 14, 30, 60, 120, 180, 270, and 360 days). A two‐way ANOVA using Tukey's procedure was used to compare each storage environment at each time point.

**Results:** By 360 days, the midazolam concentration revealed an increase from the starting concentration. The mean concentration for group A, B, and C was 79.9%, 102.0%, and 107.0%, respectively, of the labeled concentration at day 360. The increase in concentration was not statistically significant between groups B and C, but was statistically significant between groups A and B and groups A and C.

**Conclusions:** This study demonstrated that dispensing ready‐made syringes would be appropriate in order to facilitate ease of use and to expedite rescue therapy for epileptic dogs at home.

## Abstract N02: Effect of Postoperative N‐Acetylcysteine on Canine Myelopathy Secondary to Intervertebral Disc Extrusion

### **Sarvenaz Bagheri** ^1^; Annie Chen^2^, DVM, MS, DAVCIM (Neurology); Sabrina Hoehne^3^, Dr med. vet., DACVECC, DECVECC; Christine Toedebusch^4^, DVM, PhD, DAVCIM (Neurology); Vishal Murthy^5^

#### ^1^Washington State University, Pullman, WA, USA; ^2^Associate Professor (Neurology & Neurosurgery), Department of Veterinary Clinical Sciences, College of Veterinary Medicine, Washington State University, Pullman, WA, USA; ^3^Assistant Professor, Department of Veterinary Clinical Sciences, College of Veterinary Medicine, Washington State University, Pullman, WA, USA; ^4^Assistant Professor, Department of Surgical and Radiological Sciences, School of Veterinary Medicine, University of California ‐ Davis, Davis, CA, USA; ^5^Assistant Professor, Department of Veterinary Clinical Sciences, College of Veterinary Medicine, Washington State University, Pullman, WA, USA

**Background:** Primary spinal cord injury (SCI) due to intervertebral disc extrusion (IVDE) is treated by surgical decompression, but no proven treatments exist for secondary SCI. N‐acetylcysteine (NAC), a free radical scavenger, may reduce secondary SCI.

**Hypothesis:** Postoperative administration of NAC results in faster ambulation and reduced secondary SCI, as measured by serum phosphorylated neurofilament (pNF‐H) and urinary 15F2t‐isoprostane:creatinine (ISO).

**Animals:** 21 client‐owned dogs with thoracolumbar IVDE.

**Methods:** Randomized, blinded, placebo‐controlled trial. Dogs received intravenous NAC or placebo (0.9% saline) over 48 h postoperatively. Daily neurological examinations were performed. Serum and urine samples were collected before (t0) and after treatment (t48). pNF‐H and ISO concentrations were quantified by ELISA and time to ambulation estimated. Outcome measures were compared within and between groups using Wilcoxon matched‐pairs signed rank and Mann‐Whitney U tests and paired/unpaired t‐tests.

**Results:** pNF‐H significantly increased over time in both the NAC [t0: 0.31 (0.00–1.12) ng/mL; t48: 1.67 (0.00–38.23) ng/mL, *P* = 0.01] and placebo group [t0: 0.12 (0.00–13.09) ng/mL; t48: 1.34 (0.00–41.33) ng/mL, *P* = 0.008]. ISO significantly increased in the NAC [t0: 728.1 ± 186.5 pg/mg; t48: 978.6 ± 215.9 pg/mg, *P* = 0.004] but not placebo group [t0: 1103.0 ± 334.1 pg/mg; t48: 1104.0 ± 435.9 pg/mg, *P* = 0.69]. No significant differences in pNF‐H (*P* = 0.96) or ISO concentrations (*P* = 0.41) were found between groups at t48. Mean time to ambulation was not significantly different between groups [NAC: 15.6 ± 12.2 days; placebo: 9.5 ± 7.6 days, *P* = 0.31]. No adverse reactions were observed.

**Conclusions and Clinical Importance:** While well tolerated, NAC did not significantly reduce time to ambulation or secondary SCI.

## Abstract N03: Clinical Characteristics Associated With Cervical Hydrated Nucleus Pulposus Extrusion in Canines

### **Megan Lin** ^1^; Jonathan Wood^2^, DACVIM (Neurology); Emma Davies^2^, DECVN; Mark Rishniw^3^, DACVIM (Internal Medicine and Cardiology)

#### ^1^Cornell University, Ithaca, NY, USA; ^2^Neurology/Neurosurgery, Cornell University, Ithaca, NY, USA; ^3^Adjunct Professor, Department of Clinical Sciences, Cornell University, Ithaca, NY, USA

**Background:** Clinical characteristics of cervical hydrated nucleus pulposus extrusion (HNPE) in canines compared to other causes of cervical myelopathy are not well described.

**Hypothesis/Objectives:** HNPE dogs will have specific clinical characteristics allowing differentiation compared to intervertebral disc extrusion (IVDE) and fibrocartilaginous embolism (FCE).

**Animals:** 268 client‐owned dogs (46 HNPE, 192 IVDE, 30 FCE) presented to a neurology service from 2010 to 2022.

**Methods:** Retrospective study. Patients diagnosed with HNPE, IVDE, or FCE on MRI were included. Age, weight, pain score, Modified Frankel Score, and lateralizing signs were included for logistic regression. Central cord signs, disc space involvement, and ventilation requirement were evaluated separately.

**Results:** In a multinomial model (HNPE vs. IVDE, and HNPE vs. FCE), decreasing age modestly decreased the odds of HNPE (OR: 0.98 and 0.96), decreasing weight modestly increased the odds of HNPE (OR: 1.03 and 1.01). Increasing pain score increased the odds of IVDE (vs. HNPE; OR:2.05); while decreasing pain score increased the odds of FCE (vs. HNPE; OR: 0.42). Lateralization dramatically increased the odds of either IVDE or FCE (vs. HNPE; OR:2.3 and 7.3, respectively). 35% (16/46) of HNPE dogs had central cord signs, compared to 19% (36/192) IVDE and 13% (4/30) FCE. 70% of HNPE dogs had a lesion at C3‐4 or C4‐5. Ventilation was required for 9% HNPE, 1% IVDE, and 0 FCE cases.

**Conclusions and Clinical Importance:** Compared to IVDE and FCE, HNPE dogs have differences in clinical characteristics, and might be more likely to require ventilation. These differences should be considered for case management.

## Abstract N04: The CCL2/CCR4 Axis in Regulatory T‐cell Trafficking to Canine Glioma – A Novel Therapeutic Target

### **Wojciech K. Panek** ^1^; Ryan Toedebusch^2^, PhD; Bridget Mclaughlin^3^, PhD; Peter Dickinson^4^, BVSc, PhD, DACVIM (Neurology); Karen Vernau^4^, DVM, MAS, DACVIM (Neurology); Beverly Sturges^4^, DVM, DACVIM (Neurology); Chai‐Fei Li^4^, DVM, DACVIM (Neurology); Michael Berens^5^, PhD; Kevin Woolard^6^, DVM, PhD, DACVP; Maciej Lesniak, MD, MHCM, FAANS; Michael J. Marchese^7^; Jason Miska^8^, PhD; Christine Toedebusch^9^, DVM, PhD, DACVIM (Neurology)

#### ^1^University of California Davis, Davis, CA, USA; ^2^Associate Project Scientist, Surgical & Radiological Sciences, VMTH, University of California, Davis, Davis, CA, USA; ^3^Academic Program Mgmt. Ofc., Comprehensive Cancer Center, University of California, Davis, Davis, CA, USA; ^4^Professor Neurology/Neurosurgery, Department of Surgical & Radiological Sciences, Veterinary Medical Teaching Hospital, University of California, Davis, CA, USA; ^5^Professor and Director, Cancer and Cell Biology Division, The Translational Genomics Research Institute, Phoenix, AZ, USA; ^6^Associate Professor, Department of Pathology, Microbiology and Immunology, University of California, Davis, Davis, CA, USA; ^7^Professor of Neurological Surgery, Neurosurgeon‐in‐Chief, Northwestern Medicine Program Leader, Neuro‐Oncology, Department of Neurological Surgery, Lou and Jean Malnati Brain Tumor Institute, Robert H. Lurie Comprehensive Cancer Center, Feinberg School of Medicine, Northwestern University, Chicago, IL, USA; ^8^Assistant Professor, Department of Neurological Surgery, Lou and Jean Malnati Brain Tumor Institute, Robert H. Lurie Comprehensive Cancer Center, Feinberg School of Medicine, Northwestern University, Chicago, IL, USA; ^9^Assistant Professor, Department of Surgical and Radiological Sciences, VMTH, University of California, Davis, Davis, CA, USA

**Background:** Canine gliomas share many similarities with their human counterparts, including recruitment of immunosuppressive regulatory T‐cells (Tregs) that inhibit host immune responses. We have shown previously the importance of the chemokine CCL2 and its high‐affinity receptor CCR4 in recruitment of Tregs in a murine orthotopic glioma model and elevated CCL2 in canine high grade gliomas. However, mechanisms responsible for Treg recruitment in canine glioma are poorly defined.

1. **Hypotheses:** Canine Tregs possess canonical Treg markers and can be isolated for downstream assays
2. Glioma‐derived CCL2 is a potent chemoattractant for canine Tregs signaling via CCR4

**Animals:** Four healthy dogs.

**Methods:** *In vitro*, mechanistic study. Canine Tregs were characterized by flow cytometry using canonical markers CD3, CD4, CD25, FOXP3, and CCR4 and sorted using fluorescent‐activated cell sorting. CCL2 mRNA expression was assessed via RTqPCR in four canine glioma cell lines (1110, 0514, J3T‐Bg, G06A). Treg migratory capacities were assessed using a Boyden chamber assay. Anti‐CCL2 antibody and a CCR4 inhibitor (C021) were used in abrogation studies.

**Results:** The canine CD4+CD25high T‐cell population expressed canonical Treg markers FOXP3 and CCR4. Canine Treg migration was enhanced by CCL2 and glioma cell line‐derived supernatant. Blockade of the CCL2/CCR4 axis abrogated migration of Tregs. CCL2 mRNA was expressed in all cell lines and expression increased when exposed to Tregs but not to conventional CD4+ T‐cells.

**Conclusion:** CD25high expression is critical for isolation of canine Tregs. Our study validated CCL2/CCR4 as a bi‐directional Treg‐glioma signaling axis and represents a rational target for immunotherapy in clinical patients.

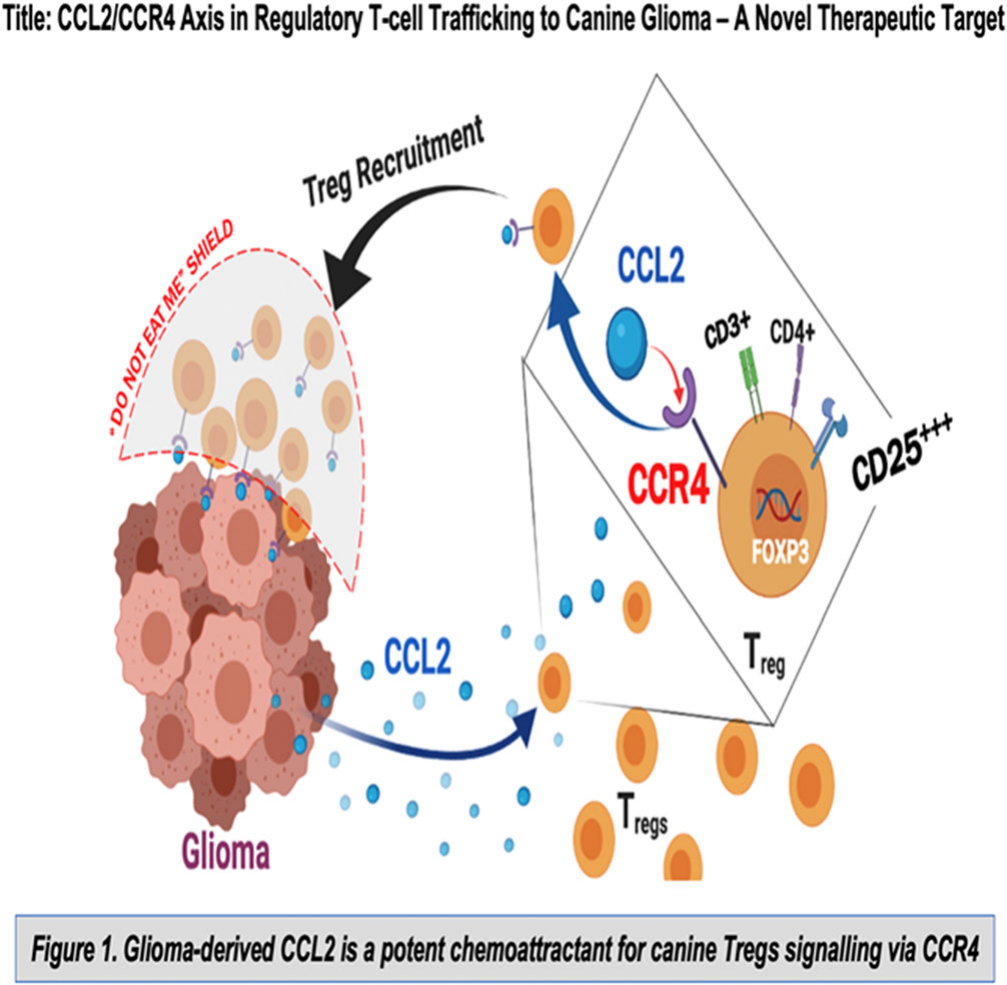

## Abstract N05: Kinematic Evaluation of Lumbosacral Vertebral Region in Large‐Breed Dogs With and Without Degenerative Lumbosacral Stenosis

### **Ashley Potts** ^1^; Ronaldo da Costa^2^, DMV, MSU, PhD, DACVIM (Neurology); Eric Hostnik^2^, DVM, MS, DACVR; Sarah Moore^2^, DVM, DACVIM (Neurology); Carolyn Nye^2^, DVM, DACVIM (Neurology and Neurosurgery); Laura Selmic^3^, BVetMed (Hons), MPH, DACVS‐SA, DECVS

#### ^1^College of Veterinary Medicine, The Ohio State University, Columbus, OH, USA; ^2^The Ohio State University, Columbus, OH, USA; ^3^Associate Professor, The Ohio State University, Columbus, OH, USA

**Background:** Degenerative lumbosacral stenosis (DLSS) shows poor correlation between imaging and clinical findings in dogs. Kinematic or dynamic imaging may improve elucidation of this relationship. Kinematic MR imaging has not been evaluated in dogs with DLSS.

**Objectives:** Describe the morphologic features of the lumbosacral vertebral column of large‐breed dogs with or without DLSS in neutral, flexed, and extended MRI positions.

**Animals:** 27 large‐breed dogs: 15 DLSS‐affected, and 12 clinically normal.

**Methods:** Prospective blinded morphologic study. Patients underwent a 3.0 T MRI in lateral recumbency (mimicking natural standing position), followed by imaging in flexed and extended positions using a kinematic positioning device. Eight morphologic parameters were assessed in each position, including Pfirrmann grade of discs, direction and cause of compression, presence/severity of foraminal stenosis, and percentage reduction in vertebral canal height.

**Results:** The lumbosacral angle was significantly different between the three positions (neutral, extension, and flexion) in both groups (*P* < 0.05). In neutral position, 15 compressions (counting both ventral and dorsal) were identified at the L7‐S1 region in DLSS dogs, with nine in the normal group. In extension, there were 20 compressions in DLSS dogs, with 14 in the normal group. There were fewer compressions in flexion in both groups. There was a significant difference in vertebral canal height between neutral and extension (*P* < 0.05) and neutral and flexion (*P* < 0.05) in the DLSS group.

**Conclusions:** Kinematic MRI is a feasible technique for evaluation of the lumbosacral region. It can exacerbate existing compressive lesions and reveal new compressions not identified with neutral positioning alone.

## Abstract N06: Double‐Blinded Crossover Trial of Adjunct Treatment With Cannabidiol in 51 Dogs With Refractory Idiopathic Epilepsy

### **Aaron Rozental** ^1^; Brooke Gallagher^2^; Daniel Gustafson^3^, PhD; Breonna Kusick^4^, MS; Isabella Corsato Alvarenga^1^, MS, PhD; Sangeeta Rao^1^, PhD; Lisa Bartner^5^, DVM, MS, DACVIM (Neurology); Stephanie McGrath^5^, DVM, MS, DACVIM (Neurology)

#### ^1^Colorado State University, Ft Collins, CO, USA; ^2^Veterinary Student, Colorado State University, Ft Collins, CO, USA; ^3^Professor, Colorado State University, Ft Collins, CO, USA; ^4^Neurology Clinical Trials Coordinator, Colorado State University, Ft Collins, CO, USA; ^5^Associate Professor, Neurology, Colorado State University, Ft Collins, CO, USA

**Background:** Approximately 30% of dogs with idiopathic epilepsy are refractory to treatment. Recent studies have suggested cannabidiol (CBD) is an effective anticonvulsant in dogs with idiopathic epilepsy but more evidence is required.

**Objective:** Evaluate the effect of adding CBD to conventional antiepileptic drugs on seizure control and to report adverse events in dogs with idiopathic epilepsy.

**Animals:** 51 client‐owned dogs diagnosed with idiopathic epilepsy were included in this study. Inclusion criteria required at least two seizures per month documented for 3 months preceding the trial.

**Methods:** This was a double‐blinded, placebo‐controlled crossover clinical trial. Twelve dogs were administered 5 mg/kg/day oral CBD, and 39 dogs were administered 9 mg/kg/day oral CBD. Dogs were randomly assigned to receive either CBD or placebo for 3 months then switched to the opposite oil for 3 months following a one‐month washout period. Total number of seizures and seizure days (clusters over a 24‐h period) were recorded. Blood work was performed periodically.

**Results:** At the 9 mg/kg/day dose, there was a decrease in number of total seizures during CBD treatment compared to placebo (*P* = 0.036). There was a significant reduction in seizure days (−24.1%) for dogs receiving 9 mg/kg/day CBD compared to placebo (+5.8%) (*P* = 0.0017). Liver enzymes increased with both doses of CBD administration.

**Conclusions:** CBD oil treatment resulted in a significant reduction in total seizures and seizure days compared to placebo when administered orally at dose of 9 mg/kg/day. This supports further studies investigating the role of CBD in the treatment of idiopathic epilepsy in dogs.

## Abstract N07: Evaluation of microRNA Dysregulation as a Prognostic Indicator in Dogs With Spinal Cord Injury

### **Alexa Stephen** ^1^; Ji‐Hey Lim^2^, DVM, PhD, DACVIM (Neurology); Sehwon Koh^3^, PhD

#### ^1^University of Missouri, Columbia, MO, USA; ^2^Assistant Professor of Neurology and Neurosurgery, University of Missouri, Columbia, MO, USA; ^3^Assistant Professor of Veterinary Medicine and Surgery, Neurosciences, University of Missouri, Columbia, MO, USA

**Background:** Spinal cord injury (SCI) induces global changes in the microRNA (miRNA) expression patterns in the experimental and clinical setting. Identification of dysregulated miRNA could potentially correlate with neurologic outcome and become a prognostic biomarker.

**Hypothesis/Objectives:** Based upon our miRNA expression profile data, our objective was to validate miRNA biomarker candidates specifically dysregulated in naturally occurring severe SCI in dogs. We hypothesize that relative expression levels of candidate miRNA would correlate with neurologic grade and outcome.

**Animals:** Serum samples were obtained from a total of 13 dogs (severe: seven paraplegics without pain perception, moderate: three paraplegics with intact pain perception, and three healthy control).

**Methods:** This is a prospective study. Total miRNA was isolated and transcribed to cDNA. qRT‐PCR was carried out against cfa‐miR‐23a, cfa‐miR‐574, chr21_19093, cfa‐miR‐195, cfa‐let‐7e, chr10_3880_hsa‐miR‐920 and chrX_46645_mmu‐miR‐5101. Relative expression levels were calculated by the Delta‐Delta Ct method using cfa‐miR‐16 as a normalization control. T‐test were performed and compared in dogs with favorable and unfavorable neurologic outcomes.

**Results:** Results showed specific up‐regulation of cfa‐miR‐574, chr21_19093 and cfa‐miR‐195, and down‐regulation of cfa‐miR‐23a, chr10_3880_hsa‐miR‐920 and chrX_46645_mmu‐miR‐5101 in dogs without pain perception. cfa‐miR‐574 demonstrated the most significant (15.9‐fold increase, *P* < 0.04) and consistent up‐regulation in the severe group. There was a positive correlation between miRNA expression and neurologic outcome at 6 weeks post‐surgery, particularly for cfa‐miR‐574 in dogs without pain perception (*P* < 0.02).

**Conclusions and Clinical Importance:** The results suggest that dysregulated miRNA expression can be used to predict SCI severity and neurologic outcome. A large cohort study is warranted.

## Abstract N08: Clinical Outcome in Paraplegic Dogs With Fibrocartilaginous Embolic Myelopathy or Acute Non‐compressive Nucleus Pulposus Extrusion

### **Go Togawa** ^1^; Melissa Lewis^2^, VDM, PhD, DACVIM (Neurology); Dillon Devathasan^3^, DVM, MS, DACVIM (Neurology); R. Timothy Bentley^4^, BVSc (Dist), MRCVS, ACVIM (Neurology); Stephanie Thomovsky^5^, DVM, MS, DACVIM (Neurology), CCRP

#### ^1^Purdue University, West Lafayette, IN, USA; ^2^Assistant Professor, Neurology, Purdue University, West Lafayette, IN, USA; ^3^Assistant Professor, Neurology, Auburn University, Auburn, AL, USA; ^4^Professor, Neurology, Purdue University; West Lafayette, IN, USA; ^5^Associate Professor, Neurology, Purdue University, West Lafayette, IN, USA

**Background:** Fibrocartilaginous embolic myelopathy (FCEM) and acute non‐compressive nucleus pulposus extrusion (ANNPE) are common causes of canine spinal cord injury with clinical similarities. Limited information is available regarding the prognosis of paraplegic dogs with FCEM and ANNPE.

**Hypothesis/Objectives:** To describe the clinical features and outcome of paraplegic deep pain positive (DPP) and deep pain negative (DPN) dogs with FCEM and ANNPE.

**Animals:** Thirty‐one client‐owned paraplegic dogs with thoracolumbar FCEM or ANNPE presenting to university hospitals between 2012 and 2022.

**Methods:** Multi‐center retrospective study. Paraplegic dogs with a clinical and magnetic resonance imaging diagnosis of FCEM or ANNPE were included. Logistic regression analysis was performed to investigate associations between diagnosis, clinical and imaging variables, and outcome (recovery of independent ambulation or not).

**Results:** On initial presentation, 14 dogs were paraplegic DPP (8 FCEM, 6 ANNPE), 17 dogs were paraplegic DPN (11 FCEM, 6 ANNPE). Outcome was available for 26 dogs (14 DPP, 12 DPN) with a median follow‐up time of 182 days (range, 0–2311). Excluding 2 DPN dogs euthanized at diagnosis, 1/10 DPN dogs (10%) regained independent ambulation, whereas 9/14 DPP dogs (64%) regained independent ambulation. DPN dogs had a significantly higher risk of not regaining independent ambulation compared to DPP dogs (OR: 29.3, CI: 2.1–419.2, *P* = 0.013). No other variables were associated with outcome (*P* > 0.05).

**Conclusions and Clinical Importance:** While the recovery of ambulation is possible, the absence of pain perception appears to be a useful negative prognostic indicator in dogs with severe thoracolumbar FCEM or ANNPE.

## Abstract N09: Concurrent Conditions and Long‐term Outcome Associated With Canine Ischemic Stroke

### **Cecilia‐Gabriella Danciu** ^1^; Rita Goncalves^2^; Jordina Caldero Carret^3^; Christophoros Posporis^4^; Espinosa Javier^4^; Steven De Decker^5^; Hanne Gredal^6^; Sophie Elizabeth Wyatt^5^

#### ^1^Royal Veterinary College, London, UK; ^2^Small Animal Teaching Hospital, Neurology Service, University of Liverpool, Liverpool, UK; ^3^Small Animal Teaching Hospital, Neurology Service, University of Liverpool, Liverpool, UK; ^4^Neurology and Neurosurgery Service, Pride Veterinary Referrals, Derby, UK; ^5^Clinical Science and Services, Royal Veterinary College, London, UK; ^6^Department of Veterinary Clinical and Animal Science, University of Copenhagen, Copenhagen, Denmark

**Background:** Little is known about prognostic factors associated with the long‐term outcome of dogs with ischemic strokes. Although post‐stroke epilepsy is a well‐recognized syndrome in humans, it is unclear if this phenomenon also occurs in dogs.

**Hypothesis/Objective:** Evaluate the occurrence of concurrent conditions, long‐term outcome, stroke recurrence and development of epileptic seizures in dogs with ischemic strokes. We hypothesized that dogs with concurrent conditions or those which develop epileptic seizures long‐term are more likely to have a poor outcome.

**Methods:** Retrospective multicenter study including dogs with ischemic stroke diagnosed with MRI. Clinical records between 2000 and 2021 were reviewed. Referring veterinarians and owners were contacted to obtain follow‐up information. Associations between outcome, concurrent disease, and occurrence of post‐stroke epileptic seizures were evaluated.

**Results:** A total of 124 dogs were included; 73 dogs (58.9%) had a concurrent condition. Median survival time for dogs with a concurrent condition was 851 days (range 1–3013) versus 696 days (range 3–3027) in dogs without concurrent conditions. Stroke recurrence was 18.5% (23/116) of which 73.9% (16/23) had a concurrent condition. A total of 8 dogs (6.5%) experienced post‐stroke epileptic seizures, without having epileptic seizures as a primary complaint. No significant association was found between long‐term outcome, presence of concurrent conditions, and occurrence of post‐stroke epileptic seizures.

**Conclusion and Clinical Importance:** In dogs diagnosed with ischemic stroke, occurrence of post‐stroke epileptic seizures or presence of a concurrent condition had no impact on long‐term outcome. Post‐stroke epileptic seizures are an uncommon occurrence in canine patients.

## Abstract N10: Causal Agents and Imaging in 85 Dogs Diagnosed With Discospondylitis (2018–2022)

### **Eileen Donoghue** ^1^; Nick Jeffery^2^, DECVN, FRCVS, BVSc, DECVS, PhD, MSc –; Sharon Kerwin^2^, DVM, DACVS, DACVIM (Neurology)

#### ^1^Veterinary Medical Teaching Hospital, Texas A&M University, College Station, TX, USA; ^2^Clinical Professor, Neurology and Neurosurgery, Veterinary Medical Teaching Hospital, Texas A&M University, College Station, TX, USA

**Background:** Discospondylitis is a well‐known cause of spinal pain and, sometimes, neurologic deficits, but the infectious causes can be difficult to identify.

**Objectives:** To describe clinical features, imaging characteristics, infectious agents, and outcomes in dogs diagnosed with discospondylitis.

**Animals:** Eighty‐five client‐owned dogs with an imaging diagnosis of discospondylitis.

**Methods:** Prospective case series systemically evaluated by serology and culture for etiology. Cases were classified as definitive (infectious etiology confirmed) or presumptive (imaging consistent with discospondylitis, no agent identified). Signalment, imaging findings, etiologic agents, and outcomes were recorded.

**Results:** A **definitive** diagnosis was made in 45 of 85 dogs. Labradors (*n =* 14) and German shepherds (*n =* 15) were the most commonly affected breeds. Of 70 blood cultures, 28 were positive (40%), while 12 of 76 urine cultures were positive (16%). Ten dogs were confirmed to have *Brucella canis*, ten had *Aspergillus*, and the remaining had various bacterial infections. Forty‐eight (56%) of dogs had multifocal lesions, with the L7/S1 disc space being the most affected site (46%). Subluxation was present in 9.4% of dogs. Fifty‐six percent of dogs were treated with antibiotics for over 3 months. Eight of ten dogs diagnosed with fungal disease were still alive at follow‐up (0 to 15 months). Five of seven dogs that underwent stabilization surgery had favorable outcomes.

**Conclusions:** Systemic investigation of causative agents of discospondylitis detected a definitive diagnosis in over 50% of the cases and suggested a high prevalence of *Brucella canis* and fungal infections. Fungal discospondylitis can be managed satisfactorily with antifungal medications in certain cases.

**Table:** Table 1. Etiologic agents in definitively diagnosed cases of discospondylitis

Definitive discospondylitis: Etiology					
	*Brucella* serology	*Aspergillus* antigen EIA	Blood culture	Urine culture	Intra‐operative culture
*Brucella*	9	0	7	4	1
Fungal	0	9	2	2	0
Other bacteria	0	0	19	6	5

## Abstract N11: Utility of Thoracic Radiography Before Advanced Imaging in Dogs With Presumed Intervertebral Disc Herniation

### **Nicole Block** ^1^; Steven Frederick^2^, LVT, VTS (Surgery); Karina Pinal^3^, DVM; Jennifer Parkes Berryessa^4^, DVM, DACVIM (Neurology); Ronald Johnson^5^, DVM, DACVIM (Neurology)

#### ^1^BluePearl Emergency + Specialty Pet Hospital, Sandy Springs, GA, USA; ^2^Clinical Studies Coordinator, BluePearl Science, BluePearl Specialty + Emergency Pet, Hospital, Tampa, FL, USA; ^3^Intern, BluePearl Specialty + Emergency Pet Hospital; ^4^Neurologist, Neurology, BluePearl Specialty + Emergency Pet Hospital, Sandy Springs, GA, USA; ^5^Neurologist, Neurology, BluePearl Specialty + Emergency Pet Hospital, Sandy Springs, GA, USA

**Background:** Acute intervertebral disc herniation (IVDH) may cause lasting neurological deficits without immediate diagnosis and treatment. However, advanced spinal imaging (anesthesia) and surgery present additional health risks. Thoracic radiography is often utilized in anesthetic risk assessment and guide clinician and client decision making in presumed IVDH cases (pIVDH), but even negative findings may not supersede the importance of emergent treatment.

**Hypothesis/Objectives:** This study's objective was to evaluate the utility of thoracic radiography in decision making in pIVDH cases. Our null hypothesis was that treatment plans for pIVDH dogs would not change due to thoracic radiograph findings.

**Animals:** 105 client‐owned dogs with pIVDH.

**Methods:** Medical records from 105 consecutive pIVDH dogs presented to the neurology service at a referral hospital were evaluated. Signalment, physical and radiographic examination notes, client communications, and treatment plans were recorded. Fisher's exact tests were used to test for associations of having a thoracic pathology with treatment plan changing. Logistic regressions were used to estimate odds‐ratios and test for the effects of breed, age, and weight on having radiographic thoracic pathology. Significance was set at 0.05.

**Results:** Radiographic diagnosis of thoracic pathology was associated with alteration of treatment plans (*P* = 0.014). The odds of a radiographic thoracic pathology were 4.6 × (OR(95%CI)=4.6 (1.4–17), *P* = 0.026) higher if the dog was aged 12 years or older.

**Conclusions and Clinical Importance:** We reject our null hypothesis. Performing routine thoracic radiography prior to advanced spinal imaging in pIVDH cases, especially in dogs 12 years of age or older, may influence the recommended treatment plan.

## Abstract N12: Veterinary Students’ Perception of Their Knowledge and Comfort of Clinical Neurology Prior to Their Rotation

### **Devon W. Hague**; Kari Foss, DVM, MS, DACVIM (Neurology)

#### Department of Veterinary Clinical Medicine, University of Illinois, Urbana, IL, USA

**Background:** Neurology curriculum varies among veterinary schools and is not a required core clinical rotation for many.

**Hypothesis/Objectives:** To evaluate the psychometric properties of a survey measuring the knowledge and comfort in clinical neurology of clinical students prior to their rotation.

**subjects:** Forty‐one fourth year veterinary students.

**Methods:** All veterinary students were asked to complete a survey one week prior to starting their elective neurology rotation. The survey included thirty‐five 7‐point Likert scale questions assessing study motivation and confidence in clinical neurology. Data was analyzed using a descriptive method.

**Results:** One hundred twenty‐three students participating in a neurology clinical elective rotation at one college of veterinary medicine. Forty‐one students (33%) completed the survey prior to the rotation. Student perceptions on their studying and learning motivation were slightly favorable, such as looking up information to gain further understanding (mean *=* 5.1) and applying practical contexts to new ideas (mean 5.7). Prior to the rotation, student confidence in neurology skills were neutral in a few areas, including performing the neurologic exam, neurolocalization, and identifying seizures (means of 4.3, 4.0, 4.6). Their confidence was low in all other areas, such as treating status epilepticus (mean 3.19), produce appropriate differentials for encephalopathy, myelopathy, lower motor neuron, and vestibular cases (means of 2.8, 3.2, 3.4, and 3.5).

**Conclusions and Clinical Importance:** Veterinary students appear to have low confidence in neurology prior to a clinical rotation. There may be a benefit of a neurology rotation to improve student confidence in this area.

## Abstract N13: Novel COL6A3 Frameshift Mutation Causes Ullrich‐Like Congenital Muscular Dystrophy in American Staffordshire Terrier Dogs

### **Leanne Jankelunas** ^1^; Vishal Murthy^2^, DVM, DACVIM (Neurology); Annie Chen^3^, DVM, MS, DACVIM (Neurology); Katie Minor^4^, RN; Steven Friedenberg^4^, DVM, PhD, DACVECC; Jonah Cullen^4^, MS; Ling Guo^5^, MD, PhD; G. Diane Shelton^5^, DVM, PhD, DACVIM (Internal Medicine)

#### ^1^Washington State University, Pullman, WA, USA; ^2^Assistant Professor, Department of Veterinary Clinical Sciences, College of Veterinary Medicine, Washington State University, Pullman, WA, USA; ^3^Associate Professor, Department of Veterinary Clinical Sciences, College of Veterinary Medicine, Washington State University, Pullman, WA, USA; ^4^Department of Veterinary Clinical Sciences, College of Veterinary Medicine, University of Minnesota, Minneapolis, MN, USA; ^5^Department of Pathology, School of Medicine, University of California San Diego, San Diego, CA, USA

**Background:** Muscular dystrophies occur in several dog breeds, arising from mutations in laminin α2, dystrophin, sarcoglycans, dystroglycan, and collagen‐6 genes. To date, there are no confirmed mutations causing any form of muscular dystrophy in American Staffordshire terriers.

**Objective:** To describe the clinical features, diagnostic testing and genetic mutation resulting in congenital muscular dystrophy in American Staffordshire terriers.

**Animals:** Two young client‐owned American Staffordshire terrier littermates with suspected congenital neuromuscular disease.

**Methods:** Neurological examination, creatine kinase activity, infectious disease titers, cerebrospinal fluid analysis, and electrodiagnostic testing were performed. Muscle biopsies were collected for histopathology and immunohistochemical staining for localization of dystrophy‐associated proteins. Genomic DNA was extracted from muscle and whole genome sequencing was performed on one of the affected dogs, and variants were compared to a database of over 700 unrelated dogs.

**Results:** Clinical signs included progressive weakness, joint contracture, and distal limb joint hyperlaxity. Creatine kinase activity was elevated in both dogs. Histopathology confirmed a dystrophic phenotype and immunofluorescence staining of muscle cryosections revealed an absence of staining for collagen‐6. Whole genome sequencing identified a novel frameshift mutation in the COL6A3 gene that was homozygous and unique to the affected dog: Sanger sequencing confirmed homozygosity for the mutation in the affected dog and littermate.

**Conclusions and Clinical Importance:** A novel COL6A3 mutation resulting in Ullrich‐like congenital muscular dystrophy was confirmed in American Staffordshire terriers. The clinical phenotype of distal joint contractures and laxity should alert clinicians to this disorder. A genetic test for this variant is now available.

## Abstract N14: Land Treadmill Gait Scoring Adapted for Underwater Treadmill in Dogs With Thoracolumbar Intervertebral Disc Extrusion

### **Melissa J. Lewis** ^1^; Stephanie Thomovsky^2^, DVM, MS, DACVIM (Neurology), CCRP, cVMA; George Moore^3^, DVM, PhD

#### ^1^Purdue University, West Lafayette, IN, USA; ^2^Clinical Associate Professor of Neurology, Purdue University, West Lafayette, IN, USA; ^3^Professor of Epidemiology and Director of Clinical Trials, Purdue University, West Lafayette, IN, USA

**Background:** The underwater treadmill (UWTM) is utilized in dogs recovering from thoracolumbar intervertebral disc extrusion (TL‐IVDE). Gait scoring is validated for dogs with TL‐IVDE walking on the land treadmill (LT) but has not been reported for the UWTM.

**Hypothesis/Objectives:** Investigate if LT gait analysis could be applied to the UWTM and if non‐ambulatory dogs walking on the UWTM, at a standardized water level, would be more likely to generate gait scores compared to the LT without support.

**Animals:** 20 dogs with TL‐IVDE managed surgically.

**Methods:** Prospective, observational study. At 0, 2, 4, 8, and 12 weeks postoperatively, paired video footage of dogs walking on the LT and UWTM (water level at the greater trochanter) was used to generate 0–100 stepping (SS) and coordination (regularity index, RI) scores. Scores were compared between treadmill type and over time.

**Results:** Seventy‐eight paired recordings were available for review. Median gait scores increased over time but did not differ significantly by treadmill type. Combining SS and RI, more recordings received scores of 0 for the LT (58/156; 37.2% [95%CI: 29.6%–45.3%]) compared to the UWTM (44/156; 28.2% [95%CI: 21.3–36.0). Scores of 0, at a visit when there was at least motor present at all joints, was more common on the LT (11/108; 10.2% [95%CI: 5.2–17.5%]) compared to the UWTM (2/108; 1.9% [95%CI: 0.2%–6.5%]).

**Conclusions and Clinical Importance:** In dogs recovering from TL‐IVDE, LT‐based gait scoring was feasible to perform in dogs walking on the UWTM and might complement other gait analysis techniques, especially for non‐ambulatory dogs.

## Abstract N15: Idiopathic and Structural Episodic Non‐intentional Head Tremor Syndrome in Dogs: 100 Cases (2004–2022)

### **Theofanis Liatis** ^1^; Sofie Bhatti^2^; Magdalena Dyrka^3^; Rodrigo Gutierrez Quintana^3^; Rita Gonçalves^4^; Megan Madden^5^; Steven De Decker^6^

#### ^1^Royal Veterinary College, London, UK; ^2^Faculty of Veterinary Medicine, Ghent University, Ghent, Belgium; ^3^School of Veterinary Medicine, University of Glasgow, Glasgow, Scotland; ^4^School of Veterinary Medicine, University of Liverpool, Neston, UK; ^5^Royal (Dick) School of Veterinary Studies, University of Edinburgh, Edinburgh, Scotland; ^6^Royal Veterinary College, University of London, London, UK

**Background:** Although episodic head tremor (EHT) is a clinical sign of idiopathic head tremor syndrome (IHTS), there is little known about structural brain abnormalities that can cause structural EHT (SHTS).

**Hypothesis/Objectives:** To describe signalment, semiology, MRI findings and outcome of dogs with EHT. We hypothesized that dogs with lesions affecting the middle cranial fossa, including third ventricle, or mesencephalic aqueduct could manifest SHTS semiology.

**Animals:** 100 client‐owned dogs with IHTS (*n =* 72) or SHTS (*n =* 28) that underwent MRI of the head.

**Methods:** Observational retrospective, multi‐center, study between 2010–2021.

**Results:** All 100 dogs had a non‐intentional isolated EHT. Lesions on MRI in SHTS dogs were localized to the middle cranial fossa (15/28), cerebrocortex (4/28), brainstem (2/28) or were multifocal (7/28) with involvement of the thalamus (5/7). Compression of the mesencephalic aqueduct (18/28) or third ventricle and/or the interthalamic adhesion (14/28) was present. Dogs with SHTS had more likely additional neurological signs and were older at onset of clinical signs and presentation than dogs with IHTS (*P* < 0.001 for both). Dogs with SHTS were more likely to be euthanized at the time of diagnosis (*P* < 0.001). Many dogs with IHTS received no treatment (41/72). In 80% of dogs with IHTS, EHT episodes decreased more than 50% or completely abated. In SHTS dogs treated for an underlying meningoencephalitis, EHT remission occurred.

**Conclusions and Clinical Importance:** Presence of other neurological signs and older age might indicate an underlying structural cause for EHT. EHT might be associated with lesions affecting the mesencephalic aqueduct, third ventricle or interthalamic adhesion.

## Abstract N16: Efficacy of High Dose Cytarabine in 62 Dogs With Severe Meningoencephalitis of Unknown Etiology (MUE)

### **Kara Majors**; Jessica Schmidt, DVM, DACVIM (Neurology); Rebecca Windsor, DVM, DACVIM (Neurology)

#### Wheat Ridge Animal Hospital, Wheatridge, CO, USA

**Background:** Severe meningoencephalitis of unknown etiology (MUE) typically carries a guarded prognosis. Standard cytarabine arabinoside (CA) dose for MUE is 100–300 mg/m^2^.

**Objective:** Describe clinical outcome of 62 dogs with severe MUE treated with monthly 600 mg/m^2^ CA

**Animals:** 62 client‐owned dogs from a private specialty hospital

**Methods:** All dogs presenting within a 30‐month period with rapidly progressive severe neurological abnormalities with marked MRI and/or CSF changes suggestive of MUE were included. 600 mg/m^2^ CA was initially administered intravenously over 6–12 h, followed by monthly 600 mg/m^2^ subcutaneous CA for a minimum of 6 months or until death.

**Results:** No severe negative effects were noted with 600 mg/m^2^ CA. Immunosuppressive corticosteroids were used for ≤7 days in all dogs before tapering. 38/62 (61%) dogs returned to normal with survival times >6 months. 36/62 (58%) dogs showed dramatic neurological improvement within 48 h of the initial dose of CA. 25/62 (40%) dogs died due to poor disease control (median time to death = 24 days, mean *=* 84 days, range 1–340 days), including 9/14 (64%) dogs with thoracolumbar myelitis and 6/7 (86%) dogs with suspected necrotizing disease. Negative medication effects were mild and attributed to corticosteroids as they resolved with taper.

**Conclusions:** 600 mg/m^2^ cytarabine was well tolerated and effectively controlled disease in the majority of dogs with severe MUE. Corticosteroids were tapered rapidly in most dogs, minimizing negative treatment side effects and suggesting disease control was likely attributable to CA. Necrotizing diseases and thoracolumbar myelitis carried a more guarded prognosis.

## Abstract N17: Evaluation of Neurofilament Light Chain as a Biomarker in Dogs with Structural and Idiopathic Epilepsy

### **Kayla M. Fowler**; Richard Shinn; John Rossmeisl; Rell Parker

#### Virginia‐Maryland College of Veterinary Medicine, Blacksburg, VA, USA

**Background:** Neurofilament light chain (NfL) is a frequently used biomarker in human medicine for both diagnostic and therapeutic monitoring purposes in various neurologic diseases. It has yet to be evaluated in either humans or dogs with epilepsy.

**Hypothesis/Objectives:** It was hypothesized that dogs with diagnosed structural epilepsy would have a significantly increased NfL concentration compared to dogs with idiopathic epilepsy. It was also hypothesized that idiopathic epileptic dogs with a recent onset of seizure activity would have a significantly increased NfL concentration compared to dogs with a chronically well‐controlled seizure frequency.

**Animals:** A total of 50 client‐owned dogs that presented to the Neurology service for an evaluation of seizures were enrolled in this study. There were 14 dogs with diagnosed structural epilepsy and 36 dogs with suspected idiopathic epilepsy.

**Methods:** A total of 52 serum and 6 CSF samples were obtained for NfL concentration measurement using single molecule array technology (Simoa) for a prospective cohort study.

**Results:** Serum NfL concentration was significantly increased in dogs with structural epilepsy when compared with dogs with idiopathic epilepsy (*P* = 0.0009). There was no significant difference in NfL concentration in dogs with an acute onset of seizures when compared to chronically well‐controlled epilepsy regardless of the underlying etiology.

**Conclusions and Clinical Importance:** Neurofilament light chain may serve as a reliable biomarker for the differentiation of structural and idiopathic epilepsy.

## Abstract N18: Serum 25‐Hydroxyvitamin D3 Concentrations in Dogs with Noninfectious Meningoencephalitis

### Jeffrey D. Laifer

#### Dogwood Veterinary Referral Hospital, Farmington, MI, USA

**Background:** Vitamin D3 (25‐OH‐D3) is a vitamin/hormone involved in calcium homeostasis. Recent research has focused on its role in immunomodulation and expression in various disease states, with low levels being associated with increased risk for immune‐mediated disease. This study evaluated serum 25‐OH‐D3 levels in dogs with non‐infectious meningoencephalitis, principally steroid‐responsive meningitis‐arteritis (SRMA) and meningoencephalitis of unknown etiology (MUE), which share similarities with multiple sclerosis in humans.

**Hypothesis/Objectives:** Mean serum 25‐OH‐D3 levels for non‐infectious meningoencephalitis (NIME) would be 25% lower than control dogs.

**Animals:** 17 client‐owned, mature dogs with NIME (SRMA and MUE) were compared to a control population of 71 adult dogs of normal health status. Patients with comorbidities, prior treatment with steroids, and on non‐commercial diets were excluded.

**Methods:** A prospective, case‐control study was performed evaluating serum 25‐OH‐D3 levels with liquid‐chromatography tandem mass spectroscopy (LC‐MS/MS).

**Results:** The SRMA group had six dogs (mean age 1.5 years), mean 25‐OH‐D3 of 19.4 ng/mL, median 16.5 ± 6 ng/mL. The MUE group had 11 dogs (mean age 5.8 years) with a mean of 26.1 ± 6.9 ng/mL. Control group had 71 adult dogs, mean of 29 ± 7.1 ng/mL. The SRMA group had a significantly lower mean 25‐OH‐D3 level compared to the control population (*P* = 0.002), while the MUE group did not (*P* = 0.102).

**Conclusions and Clinical Importance:** Lower 25‐OH‐D3 levels may allow for its use as a biomarker for SRMA. It may offer insight into its pathogenesis and alternative ways to manage this disease.

## Abstract N19: A Minimally Invasive Surgical Technique to Hemilaminectomies in Chondrodystrophic Dogs with Thoracolumbar Intervertebral Disc Extrusions

### **Brittany MacQuiddy**; Lisa Bartner, MS, DVM, DACVIM (Neurology); Angela Marolf; Sangeeta Rao; Emily Dupont; Taylor Adams; Eric Monnet

#### Veterinary Teaching Hospital, Colorado State University, Fort Collins, CO, USA

**Background:** The minimally invasive surgical (MIS) technique may be a viable alternative to the standard open approach hemilaminectomy; however, there are no prospective clinical studies that evaluate this technique in small‐breed dogs with naturally occurring intervertebral disc extrusions (IVDE) in the thoracolumbar region.

**Objectives:** The objective was to evaluate the use of a MIS approach to perform hemilaminectomies in chondrodystrophic dogs with IVDE. Additionally, we aimed to evaluate the degree of soft tissue trauma using the MIS procedure compared to the standard open approach.

**Animals:** Eight client‐owned dogs presented to the Colorado State University Veterinary Teaching Hospital with acute onset thoracolumbar IVDE were included in this study.

**Methods:** This was a prospective, randomized case series. Patients were assigned to undergo an MIS (group 1; *n =* 4) or a standard open approach (group 2; *n =* 4) for a hemilaminectomy. A post‐operative MRI was performed in all cases.

**Results:** Conversion to an open approach was not necessary for any case in group 1. All cases had adequate spinal cord decompression on post‐operative MRI. There was no significant difference in soft tissue changes noted on post‐operative MRI between the two groups.

**Conclusions and Clinical Importance:** The MIS approach to hemilaminectomies in dogs with IVDE can successfully be performed to decompress the neural tissue and appears to have similar clinical outcomes in the early postoperative period compared to the open approach. Larger studies are needed to determine potential advantages of the MIS technique compared to the standard approach in veterinary medicine.

## Abstract N20: Analgesic and Anti‐inflammatory Effects of Lidocaine in Dogs Undergoing Thoracolumbar Hemilaminectomy for Intervertebral Disc Extrusion

### **Ruby Ng**; Andrea Sanchez; Geoffrey Wood; Fiona James; Gabrielle Monteith; Luis Gaitero

#### Ontario Veterinary College, Guelph, ON, Canada

**Background:** Lidocaine reduces post‐operative pain and shows anti‐inflammatory properties through reduction of interleukin‐6 (IL‐6) levels in humans undergoing spinal surgery. The analgesic effects of lidocaine after spinal surgery have not been studied in dogs.

**Objectives:** To evaluate the effects of intra‐operative intravenous (IV) lidocaine continuous rate infusion (CRI) on post‐operative pain and IL‐6 levels in dogs undergoing thoracolumbar hemilaminectomy.

**Animals:** 40 client‐owned dogs undergoing hemilaminectomy for intervertebral disc extrusion (IVDE).

**Methods:** Prospective, randomized, blinded, controlled study. Dogs were anesthetized with a standardized protocol that included fentanyl CRI for intra‐ and post‐operative analgesia. Additionally, dogs received either lidocaine CRI at 100 μg/kg/min or NaCl 0.9% (control) intraoperatively. Pain scores were assessed on presentation, 0‐, 2‐, 6‐, 12‐, and 24‐h post‐anesthesia recovery via the short form of the Glasgow Composite Measure Pain Scale (CMPS‐SF). IL‐6 levels were assessed on presentation, 0‐, 6‐, and 48‐h post‐operatively.

**Results:** Lidocaine group had significantly lower pain scores compared to control group at 0‐hour post‐anesthesia (*P* = 0.023) with no significant differences between groups at other times. There were no significant differences in IL‐6 levels between groups and no correlation between IL‐6 values and post‐operative pain scores.

**Conclusion and Clinical Importance:** Intra‐operative IV lidocaine administration is associated with a lower CMPS‐SF score during the immediate post‐operative period with no change in IL‐6 levels 48 h post‐operatively when compared to dogs receiving only opioids. Intra‐operative IV lidocaine CRI should be considered in the anesthetic protocol for thoracolumbar hemilaminectomy in dogs although its benefits are not mediated by an IL‐6 reduction.

## Abstract N21: Characterization of Endocannabinoids in the CSF and Serum of Dogs with Idiopathic Epilepsy

### **Aaron Rozental** ^1^; Justine Stalnaker^2^, DVM; Jacqueline Chaparro^3^, MS, PhD; Jessica Prenni^4^, PhD; Stephanie McGrath^4^, DVM, MS, DACVIM (Neurology)

#### ^1^Colorado State University, Fort Collins, CO, USA; ^2^Woodside Equine Clinic, Ashland, VA, USA; ^3^Research Scientist, Colorado State University, Fort Collins, CO, USA; ^4^Associate Professor, Colorado State University, Fort Collins, CO, USA

**Background:** Endocannabinoids have been implicated in the pathogenesis of seizures. Specifically, an endocannabinoid, anandamide (AEA), has been shown to be elevated in the CSF of dogs with idiopathic epilepsy (IE), compared to healthy dogs. This study aimed to evaluate AEA, 2‐arachydonoylglycerol (2‐AG), and three metabolites of AEA (prostamides) in the CSF and serum of dogs with poorly controlled IE (PCIE), well‐controlled IE (WCIE), and healthy controls (HC).

**Objective:** We hypothesized higher AEA and prostamides in PCIE compared to HC and WCIE dogs and that 2‐AG would not be different between groups.

**Animals:** Banked samples from 47 dogs were included in this study. Epileptic dog samples were collected during routine workups. Healthy control samples were collected as part of other (unrelated) studies.

**Methods:** There were 47 banked CSF and 34 paired serum samples. Dogs were deemed PCIE with >2 seizures per month and WCIE with <2 seizures per month. All samples were analyzed using liquid chromatography‐mass spectrometry.

**Results:** Prostamides were below detectable limits in all samples. Serum AEA was higher in PCIE compared to other groups (*P* = 0.0007). There were no differences in 2‐AG levels in any group. Serum AEA was lower in dogs with seizures ≤4 days from collection compared to those collected >4 days after the most recent seizure (*P* = 0.0392).

**Conclusions:** This supports previous research and suggests AEA has a role in IE that requires further analysis. Prostamide levels were below detectable limits in this study and may not be significant in IE.

Serum anandamide levels in each group (nM)

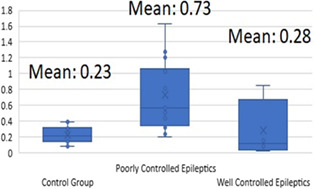

## Abstract N22: The T2‐FLAIR Mismatch Sign as an Imaging Biomarker for Canine Oligodendrogliomas

### **Josefa K. Garcia** ^1^; Rell Parker^2^; Thomas Cecere^3^; John Robertson^4,5,6^; John Rossmeisl^2,4,5,6^

#### ^1^Virginia‐Maryland College of Veterinary Medicine, Blacksburg, VA, USA; ^2^Department of Small Animal Clinical Sciences and Animal Cancer Care and Research Center, Virginia‐Maryland College of Veterinary Medicine, Blacksburg, VA, USA; ^3^Department of Biomedical Sciences & Pathobiology, Virginia‐Maryland College of Veterinary Medicine, Blacksburg, VA, USA; ^4^Veterinary and Comparative Neuro‐oncology Laboratory, Virginia‐Maryland College of Veterinary Medicine, Blacksburg, VA, USA; ^5^School of Biomedical Engineering and Sciences, Virginia Tech‐Wake Forest University, Blacksburg, VA, USA; ^6^Brain Tumor Center of Excellence, Comprehensive Cancer Center, School of Medicine, Wake Forest University, Winston‐Salem, NC, USA

**Background:** In humans, the T2‐FLAIR mismatch sign (T2FMM) is a specific imaging biomarker for IDH1‐mutated, 1p19q non‐codeleted low‐grade astrocytomas (LGA). The T2FMM is characterized by a homogeneous hyperintense T2W signal and a hypointense signal with a hyperintense peripheral rim on FLAIR sequences. In canine gliomas, the T2FMM has not been described.

**hypotheses/objectives:** In dogs with focal intra‐axial brain lesions, T2FMM will discriminate gliomas from other pathologies. The T2FMM will be associated with the LGA phenotype and presence of microcysts on histopathology. Interobserver agreement for T2FMM MRI features will be high.

**Animals:** 186 dogs with focal intra‐axial lesions on brain MRI; 146 with histologically confirmed oligodendrogliomas (*n =* 90), astrocytomas (*n =* 47), undefined gliomas (*n =* 9), 33 with cerebrovascular accidents, and 7 with inflammatory lesions.

**Methods:** Two blinded raters evaluated the 186 MRI studies retrospectively to identify cases with the T2FMM. Histopathologic and immunohistochemical slides of T2FMM cases were evaluated for morphologic features and IDH1‐mutations and compared to cases without the T2FMM. Gene expression analyses were performed on a subset of oligodendrogliomas (*n =* 10) with and without T2FMM.

**Results:** T2FMM was identified in 14/186 (8%) of MRI, and all dogs with T2FMM had oligodendrogliomas (*n =* 12 low‐grade [LGO], *n =* 2 high‐grade [HGO]; *P* < 0.001). Microcystic change was significantly associated with the T2FMM (*P* < 0.00001). In oligodendrogliomas with T2FMM, IDH1‐mutations or specific differentially expressed genes were not identified.

**Conclusion and Clinical Importance:** T2FMM can be readily identified on routinely obtained MRI sequences. T2FMM is a specific biomarker for canine oligodendroglioma and was significantly associated with non‐enhancing LGO.

## Abstract N23: Disposition and Bioequivalence of Single‐Dose Compounded and Commercial Extended‐Release Levetiracetam in Healthy Dogs

### Aaron Paushter, JD, DVM; **Kari Foss**, DVM, MS, DACVIM (Neurology); Devon Hague, DVM, DACVIM (Neurology); Jennifer Reinhart, DVM, PhD, DACVIM (SAIM), DACVP; Lauren Forsythe, PharmD, DICVP

#### University of Illinois Urbana‐Champaign, Champaign, IL, USA

**Background:** Levetiracetam is an antiepileptic medication used in veterinary medicine and is available in an extended release (ERLEV) oral formulation administered twice daily. Per manufacturer specification, the large tablets cannot be crushed or split, limiting its use in small dogs.

**Hypothesis/Objectives:** To determine the bioequivalence of compounded ERLEV (COMP) and commercial ERLEV (COMM) in healthy dogs following administration of a single oral dose.

**Animals:** Twelve healthy dogs, no less than 12 months old and weighing at least 15 kilograms.

**Methods:** Dogs were prospectively enrolled in a randomized crossover study and assigned to receive a single oral dose of COMM (intact tablet) or COMP (partitioned tablet). Samples were collected at prescribed timepoints during the 24 h after administration, with the other formulation given following a washout period. Serum levels were quantified using a validated immunoassay. Pharmacokinetic analysis for COMM and COMP was performed using non‐compartmental methods. COMM and COMP were considered bioequivalent if the 90% CI fell within ±20% for maximum serum concentration (*C*_max_) and area under the curve (AUC).

**Results:** The geometric mean of the difference between treatments in *C*_max_ was −0.56 μg/mL (90% CI: −7.4%, +1.1%) and AUC was −22 μg*h/mL (90% CI: −17.8%, −10.8%). Based on a target CI of ±20% for both parameters, the treatments were considered bioequivalent.

**Conclusions and Clinical Importance:** Partitionable COMP may be a viable treatment option in small breed epileptic dogs who cannot receive COMM and may improve medication adherence relative to immediate‐release levetiracetam, which requires three times daily dosing.

## Abstract N25: Neurofilament Light Chain as a Biomarker for Distinguishing IE from Structural Brain Diseases in Dogs

### **Minju Baek**; Jookyung Sung, DVM; Yoonhoi Koo, DVM; Taesik Yun; Yeon Chae, DVM; Dohee Lee; Hakhyun Kim; Mhan‐Pyo Yang; Byeong‐Teck Kang

#### Veterinary Teaching Hospital, Chungbuk National University, Cheongju, Korea

**Background:** The neurofilament light chain (NfL) is a cytoskeletal protein found in axons. When there is neuroaxonal damage, NfL is released into the peripheral circulation. Therefore, NfL has been used as a potential biomarker for diagnosing various human neurologic diseases. However, there have been few studies of NfL in veterinary medicine.

**Objectives:** To evaluate the diagnostic value of NfL in dogs with intracranial disease.

**Animals:** This retrospective study included 46 healthy dogs, 31 dogs with idiopathic epilepsy (IE), 45 dogs with meningoencephalitis of unknown aetiology (MUE), 20 dogs with hydrocephalus, and 19 dogs with brain tumors (BT).

**Methods:** In this case‐control study, serum NfL levels were measured using single‐molecule array technology.

**Results:** The serum NfL level in dogs with each structural disease was significantly higher than in healthy dogs and dogs with IE (*P* = 0.01). The area under the receiver operating characteristic curve of NfL for differentiating between dogs with structural diseases and IE was 0.868. An optimal cut‐off value of the NfL 27.10 pg/mL had a sensitivity of 86.67% and a specificity of 74.19% to differentiate the dogs with IE from those with other structural brain diseases. There were significant correlations between NfL concentrations and lesion size: (1) MUE, *P* = 0.009, *r =* 0.429; (2) hydrocephalus, *P* = 0.012, *r =* 0.563.

**Conclusions and Clinical Importance:** Serum NfL could be a useful biomarker for distinguishing IE from structural diseases in dogs and predicting the lesion sizes of MUE and hydrocephalus.

## Abstract N26: Neurological Examination and Magnetic Resonance Imaging Findings in Dogs Diagnosed with Central Vestibular Disease

### **Gilad Fefer**; Karen Munana, DVM, DACVIM (Neurology); Peter Early, DVM, DACVIM (Neurology); Chris Mariani, DVM, PhD, DACVIM (Neurology); Natasha Olby, Vet MB, PhD, DACVIM (Neurology); Arangan Nagendran, DECVN

#### College of Veterinary Medicine, North Carolina State University, Raleigh, NC, USA

**Background:** Neurological signs consistent with central vestibular disease include a head tilt with vertical nystagmus, proprioceptive deficits, altered mentation, cranial nerve dysfunction or cerebellar signs. There is little data regarding which of these signs are most predictive of central vestibular disease.

**Hypothesis/Objectives:** Evaluate the relationship between clinical signs associated with central vestibular disease and magnetic resonance imaging (MRI)‐identified lesions.

**Animals:** One hundred and five hospital dogs diagnosed with central vestibular disease based on neurological examination with an MRI performed.

**Methods:** Retrospective evaluation. Dogs with a head tilt, and facial nerve dysfunction only, were excluded. Clinical signs, presence and location of lesions on MRI, and final diagnosis were recorded and evaluated. Prediction of MRI lesions were compared between dogs with one and multiple clinical examination abnormalities.

**Results:** Lesions on MRI were confirmed in 87 of the dogs (83%). Vertical nystagmus as the sole additional neurological finding was predictive of a lesion on MRI in 20% of dogs (*n =* 15). All dogs with concurrent proprioceptive deficits (*n =* 32) or cerebellar ataxia (*n =* 2) only, had lesions on MRI. Dogs with only one clinical examination abnormality (*n =* 55) were less likely to have a structural lesion on MRI compared to dogs with two or more abnormalities (*n =* 50) (*P* = 0.003).

**Conclusions and Clinical Importance:** Vertical nystagmus as the sole addition clinical sign of central vestibular disease was the least predictive for identifying a lesion on MRI. More than one concurrent abnormality on examination is associated with higher likelihood of identifying lesions on MRI.

## Abstract N27: Viability of Using Diffusion Tensor Imaging to Detect Structural Abnormalities in Dogs with Idiopathic Epilepsy

### **Grace Kadler** ^1^; Alex zur Linden^2^, DVM, DACVR; Luis Gaitero^3^, DVM, DECVN; Fiona James^4^, DVM, MSc, DVSc, DACVIM (Neurology)

#### ^1^Ontario Veterinary College, Guelph, ON, USA; ^2^Associate Professor in Diagnostic Imaging, Clinical Studies, Ontario Veterinary College, Guelph, ON, USA; ^3^HSC Chief Medical Officer, Associate Professor in Neurology and Neurosurgery, Clinical Studies, Ontario Veterinary College, Guelph, ON, USA; ^4^Associate Professor & Chief Neurology and Neurosurgery Service, Clinical Studies, Ontario Veterinary College, Guelph, ON, USA

**Background:** Idiopathic epilepsy (IE), a common breed‐specific disease in dogs, affects cortical white matter connectivity. This can be studied using diffusion tensor magnetic resonance imaging (DTI) by measuring fractional anisotropy (FA).

**Hypothesis/Objectives:** To determine whether DTI identifies abnormalities in the genu and splenium of the corpus callosum in dogs with IE. The hypothesis predicted a significant difference in FA of healthy dogs (HD) and dogs with IE (IE) who were either responsive to anti‐seizure drugs (IE+) or resistant (IE−).

**Animals:** Thirteen Nova Scotia duck tolling retrievers: 3 HD, 5 IE+, and 5 IE−.

**Methods:** Retrospective observational study design: FA of the genu and splenium was acquired by hand‐drawing regions of interest determined by consensus between researchers’ blind to animal groupings. ANOVA compared FA means between groups. Levene's test examined variance.

**Results:** Age was a confounding variable, *r =* 0.74, *P* = 0.02. No differences in mean FA were identified; for the splenium, *P* = 0.0993. Variance in the genu between HD and IE was *P* = 0.0535.

**Conclusions and Clinical Importance:** This exploratory study had fewer HD than IE and was confounded by age. Two questions were highlighted for future investigations: mean FA in the splenium or other white matter regions and variance distribution patterns between HD and IE. A larger sample size with age‐matched groups would be needed to determine true differences.

## Abstract N28: The Impact of Rescue Medication in Dogs with Emergency Seizure Disorders: An Owner Perspective

### Charlotte S. Kähn^1^; Holger Volk^2^, DECVN, PhD, PGCAP, FHEA, MRCVS; **Marios Charalambous** ^3^, DVM, PhD, DACVIM, DECVN, FHEA, MRCVS

#### ^1^Department of Small Animal Medicine and Surgery, University of Veterinary Medicine Hannover, Hannover, Germany; ^2^Department Chair for Small Animal Diseases, Neurology, Department of Small Animal Medicine and Surgery, University of Veterinary Medicine Hannover, Hannover, Germany; ^3^Research Associate, Neurology, Department of Small Animal Medicine and Surgery, University of Veterinary Medicine Hannover, Hannover, Germany

**Background:** Emergency seizure disorders such as status epilepticus are unlikely to stop spontaneously, and prolonged seizures become progressively more resistant to treatment. Early administration of effective rescue medications, in particular benzodiazepines, at the seizure onset by the owners can be life‐saving and brain protecting. Clinical studies evaluating the use of rescue medications in hospital environment exist; however, the owner perspective has not been assessed to date.

**Hypothesis/Objectives:** To analyze the use of seizure rescue medication in dogs by the owner at home.

**method:** Observational study based on surveys of owners of dogs suffering from seizure disorders.

**Results:** The questionnaire was answered by 1563 dog owners, of which 761 provided complete and accurate answers suitable for analysis. Of these, 71% administered diazepam, 19% midazolam, 6% levetiracetam, 0.3% lorazepam, and 4% more than one rescue or other medication(s). Overall, the owner reported success rate for intranasal midazolam (97%) was perceived better than rectal diazepam (63%). Intranasal midazolam and rectal diazepam could cease the seizures within 5 min in 90% and 45% of the dogs, respectively. Owners reported a compliance and comfort level of 95% for intranasal midazolam administration and 66% for rectal diazepam.

**Conclusions and Clinical Importance:** Even though rectal diazepam was the most commonly used rescue medication in this survey population, intranasal midazolam was perceived by the owners as a better option regarding success rates, time to seizure cessation and owner compliance.

## Abstract N29: Evaluation of Inflammatory Mediators and Hematologic Parameters in Canine Pentylenetetrazole‐Induced Seizure Model

### **Dohee Lee** ^1^; Yoonhoi Koo^2^; Taesik Yun^2^; Yeon Chae^2^; Dohee Lee^2^; Hakhyun Kim^2^; Mhan‐Pyo Yang^2^; Byeong‐Teck Kang^2^

#### ^1^College of Veterinary Medicine, Chungbuk National University, Cheongju, Korea; ^2^Laboratory of Veterinary Internal Medicine, College of Veterinary Medicine, Chungbuk National University, Cheongju, Korea

**Background:** Neuroinflammation is known as the cause and result of seizure and epilepsy.

**Hypothesis/Objectives:** This study aimed to identify hematologic inflammatory parameter (HIP) and inflammatory mediators that change after single seizure in the canine pentylenetetrazole (PTZ)‐induced seizure model.

**Animals:** Five healthy beagle dogs were included in this study.

**Methods:** The 3% PTZ solution was infused until generalized convulsion occurred. Blood sampling time points were divided into two periods: short period (baseline, 30, 60, 90, and 120 min after seizure induction) and long period (baseline, 2, 6, 12, 24, and 48 h after seizure induction). HIP was calculated and serum prostaglandin E2 (PGE2) and leukotriene B4 (LTB4) concentrations were estimated using the enzyme‐linked immunosorbent assay.

**Results:** Significant changes (*P* < 0.05) in each HIP were observed at each time point; NM (90 min), NLR (60, 90, and 120 min), LMR (60, 90, 120 min, 2, 12, and 24 h), PAR (90 min), LA (60, 90, 120 min, and 2 h), and NP (6 h). After seizure induction, LTB4 significantly increased (*P* < 0.05) at 60, 90 min, 2, 6, and 48 h. PGE2 significantly increased only 6 h after seizure induction (*P* < 0.05). The LA was identified as an LTB4‐correlated biomarker and showed significant changes in long‐period experiment (*P* < 0.05, *r =* −0.4194).

**Conclusion and Clinical Importance:** The LA was the only HIP that the 5‐lipoxygenase pathway may be related with seizure‐associated neuroinflammation.

## Abstract N30: Serum Levels of Complement C3 and C4 in Dogs with Idiopathic Epilepsy

### **Yelim Lee**; Seonggweon Kang; Yoonhoi Koo; Taesik Yun; Yeon Chae; Dohee Lee; Hakhyun Kim; Mhan‐Pyo Yang; Byeong‐Teck Kang

#### Laboratory of Veterinary Internal Medicine, College of Veterinary Medicine, Chungbuk National University, Cheongju, Korea

**Background:** The complement system, a key component of innate immunity, is a neuroinflammatory pathway. There are increased levels of complement factors in animal epilepsy models and patients with epilepsy.

**Objectives:** To determine whether complement dysregulation occurs in dogs with idiopathic epilepsy (IE) and whether serum complement levels are potential diagnostic markers of IE.

**Animals:** Forty‐nine dogs with IE and 29 healthy dogs were included in this study.

**Methods:** In this case‐control study, the serum levels of the third (C3) and fourth (C4) components of the complement system were measured using a canine‐specific ELISA kit.

**Results:** Serum C3 and C4 levels were significantly higher (*P* < 0.001 and *P* = 0.03, respectively) in dogs with IE than in healthy control dogs. In contrast, no significant differences were observed in serum C3 and C4 concentrations between dogs in the treatment and non‐treatment groups. Regarding clinical characteristics, dogs with a seizure frequency >3 times/month presented with significantly higher serum C3 (*P* < 0.01) and C4 (*P* = 0.01) concentrations than those with a seizure frequency ≤3 times/month.

**Conclusions and Clinical Importance:** The classical complement pathway might be associated with epileptogenesis in dogs with IE, and serum C3 and C4 levels could be diagnostic biomarkers of IE.

## Abstract N31: Long‐Term Effect of Imepitoin Treatment in Dogs with Epilepsy

### **Léa Monneret** ^1^; Jasmin Neßler^2^; Enrice Hünerfauth^2^; Johannes Erath^3^; Holger Volk^2^; Andrea Tipold^2^

#### ^1^Anicura Flensburg; ^2^Kleintierklinik Tierärztliche Hochschule Hannover; ^3^Tierklinik Dr Erath+Vetter

**Background:** In randomized, blinded studies, imepitoin had similar efficacy to reduce seizure frequency as phenobarbital but with a broader safety profile. After approximately 10 years since drug approval in Europe, the efficacy has been re‐evaluated in a heterogeneous dog population.

**Hypothesis/Objectives:** Imepitoin is effective in reducing seizure frequency during long‐term treatment in dogs with epilepsy.

**Animals:** Study participants were 113 dogs with epilepsy treated with imepitoin for between 3 months and 8 years.

**Methods:** In a questionnaire‐based, retrospective study, dog owners provided information on effect of treatment on seizure frequency, seizure severity (single seizures, cluster seizures, status epilepticus), side effects, and quality of life of the dogs and their owners.

**Results:** Seizure reduction of at least 50% was observed in 43.36% of study participants, with 25 of these dogs becoming seizure‐free. 56.7% (64/113) of the dogs had no cluster seizures before or during treatment. Sixteen dogs became free of cluster seizures under therapy; twelve dogs developed cluster seizures. A similar effect was seen evaluating the occurrence of status epilepticus. Polyphagia was the most frequently observed side effect. During treatment, 46.04% of dog owners became less concerned about seizure frequency and 51.32% became less concerned about seizure severity.

**Conclusions:** Efficacy in seizure frequency reduction remains satisfactory during long‐term treatment with imepitoin, leading to a better quality of life for dogs with epilepsy and for their owners.

## Abstract N32: Deferoxamine‐Preconditioned cAT‐MSC‐Derived Extracellular Vesicles Alleviate Inflammation in an EAE Mouse Model Through STAT3 Regulation

### **Su‐Min Park** ^1^; Yong‐Hun Oh^1^; Ga‐Hyun Lim^1^; Ga‐Hee Yun^1^; Kim Kyung‐Bo^1^; Ju‐Hyun An^2^; Kyung‐Won Seo^1^; Hwa‐Young Youn^1^

#### ^1^Seoul National University, Seoul, Republic of Korea; ^2^Kangwon National University, Chuncheon, Gangwon, Republic of Korea

**Background:** Extracellular vesicles (EVs) derived from mesenchymal stem cells (MSCs) have been investigated as a treatment option for autoimmune diseases. Hypoxic stimulation is a strategy to improve the function of MSC‐derived EVs.

**Methods:** DFO, an iron chelator, was used to induce hypoxic conditions. The cAT‐MSCs were cultured with DFO for 48 h, and EVs were isolated from culture media. EAE mice were divided into different groups based on intranasal administration of EVs or EVDFO. On day 25 post‐EAE induction, the mice were euthanized, and the spleen, brain, and spinal cord were analyzed into histopathologic and expression of RNA and protein level.

**Results:** EVDFO administration resulted in a significant treatment effect in the EAE model. Histologically, in the EV and EVDFO groups, the infiltration of inflammatory cells decreased significantly, and demyelination was alleviated. Immunofluorescence staining showed that the expression of CD206 and Foxp3, markers of M2 macrophages and regulatory T (Treg) cells, respectively, increased significantly in the EVDFO group compared to the EAE and EAE+EV groups. Also, the number of CD4+CD25+Foxp3+Treg cells in the spleen showed a greater increase in the EAE+EVDFO group than in the EAE+EV group. Following EV treatment, STAT3 expression of brain and spleen decreased compared to the EAE group.

**Conclusions:** EVDFO treatment has a relatively higher efficacy in reducing inflammation than non‐preconditioned EV treatment. Moreover, our findings suggest that STAT3 regulation may play a major role in regulating T cells and that EVDFO can control the STAT3 pathway.

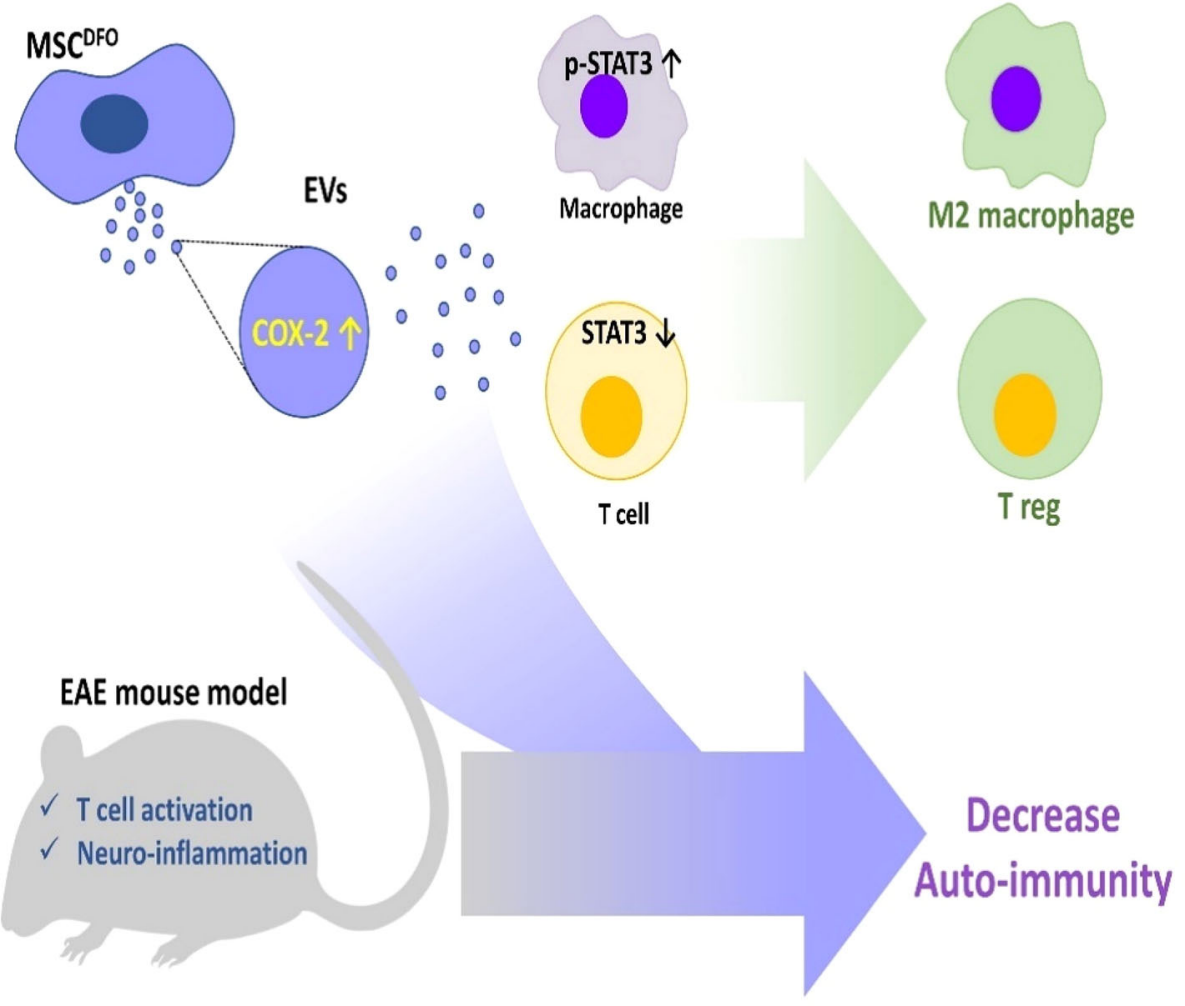

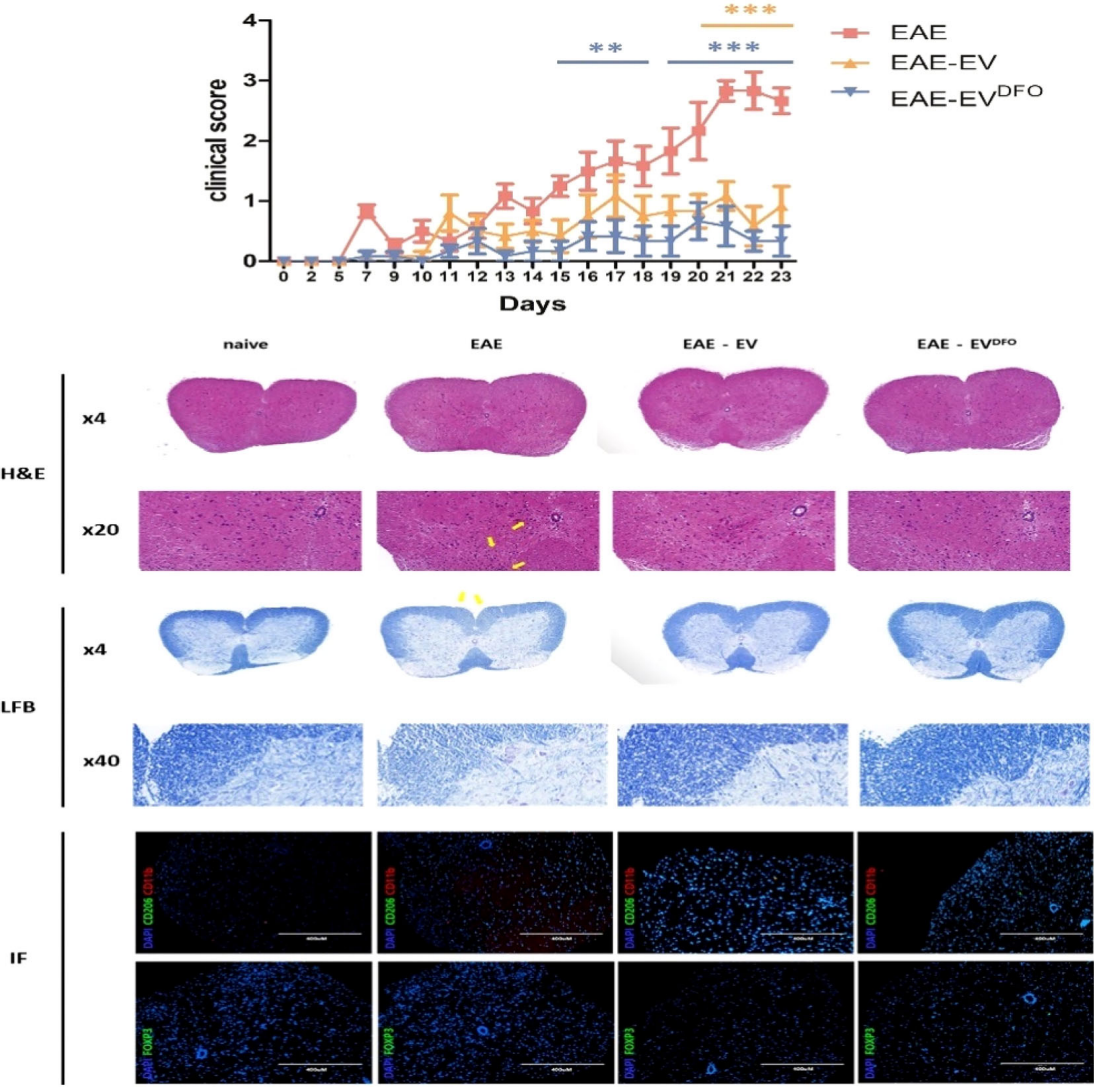

## Abstract N33: Gluten‐Associated Antibody Assessment in Diverse Dog Breeds With Suspected Paroxysmal Dyskinesia

### **Casey B. Rogers** ^1^; Dr Nina Meyerhoff^2^, DECVN; Prof. Dr Holger Volk^2^, PhD, PGCAP, DECVN, FHEA, MRCVS

#### ^1^University of Veterinary Medicine Hannover, Hannover, Lower Saxony, Germany; ^2^Neurology, Small Animal Clinic, University of Veterinary Medicine Hannover, Hannover, Lower Saxony, Germany

**Background:** Paroxysmal gluten‐sensitive dyskinesia is a subtype of movement disorders classified as canine paroxysmal dyskinesia (cPD) which until now has only been diagnosed in Border Terriers (BT). Dogs show elevated levels of anti‐gliadin and transglutaminase immunoglobulins.

**Objectives:** The objective of this study was to report cPD with positive gluten serology in dog breeds other than BT.

**Animals:** Thirty‐one client‐owned dogs with suspected cPD.

**Methods:** Dogs’ hospital records in which serum was tested for anti‐gliadin and transglutaminase immunoglobulins were retrospectively assessed. Thirty‐one dogs showing episode characteristics for cPD and having undergone a work‐up consistent with tier 1 or tier 2 confidence level for canine epilepsy were analyzed. A follow‐up was held to inquire on the dogs’ wellbeing and response to diet change.

**Results:** Fourteen dogs were tested positive for gluten‐sensitivity with antibodies against gliadin or transglutaminase being elevated. In seven dogs, serology was considered questionable, being mildly elevated, and ten dogs tested negative for the aforementioned antibodies. According to the owners’ reports, five of the positive dogs had no further episodes after feeding a strictly gluten‐free diet, with one of the dogs relapsing twice after being fed gluten. Three dogs had a reduction in episode frequency of >50%, and two dogs showed shorter and less intense episodes.

**Conclusion and Clinical Importance:** A considerable subset of various dog breeds with presumed cPD showed laboratory signs of gluten sensitivity, as well as response to gluten‐free diets. Clinicians presented with cPD cases should consider gluten‐sensitive cPD, as a possible underlying cause.

**Table:** Table 1. Sex, breed, weight and age at presentation and episode onset of the 31 dogs, categorized by gluten‐sensitivity test results into positive, questionable, negative and total

Sex	Positive dogs	Questionable dogs	Negative dogs	Total
Female	4	3	1	8
Female, neutered	1	0	4	5
Male	5	2	1	8
Male, neutered	4	2	4	10
Breed	Positive dogs	Questionable dogs	Negative dogs	Total
Mixed breed	5	2	4	11
Großpudel	1	0	0	1
Cairn Terrier	1	0	0	1
Rhodesian Ridgeback	1	1	0	2
Russian Terrier	1	0	0	1
Perro de Agua espanol	1	0	0	1
Sheltie	1	0	0	1
Pomeranian	1	0	1	2
Border Terrier	1	0	0	1
Norwich Terrier	0	1	0	1
Akita Inu	0	1	0	1
Chihuahua	0	1	0	1
Poodle	0	1	1	2
Beagle	0	0	1	1
Podenco	0	0	1	1
Pug	0	0	1	1
Bichon frise	0	0	1	1
Australian Shepherd	1	0	0	1
Total	14	7	10	31
Weight	Positive dogs	Questionable dogs	Negative dogs	Total
<10 kg	6	2	6	14
10–20 kg	4	3	3	10
20–30 kg	2	0	1	3
>30 kg	2	2	0	4
Range (kg)	2,0–35,5	3,0–45,6	2,8–23,6	2,0–45,6
Median (kg)	16,06	17,5	10,05	14,45
Age at presentation (years)	Positive dogs	Questionable dogs	Negative dogs	Total
Range	3–11	2–12	1,5–8	2–12
Median	5	6 1/3	5 1/7	5 1/2
Age at onset (years)	Positive dogs	Questionable dogs	Negative dogs	Total
Range	1,5–9	1–8	1–8	1–8
Median	4 1/6	3 6/7	3 5/7	4

## Abstract N34: Clinical Trial of Crisdesalazine (GedaCure) on Meningoencephalitis in Dogs

### **Woo‐Jin Song** ^1^; Saeyoung Lee^1^, DVM; Jongjin Park^1^, DVM; Minkun Kim^1^, DVM; Youngmin Yun^2^, DVM, DKCVIM, PhD

#### ^1^Jeju National University, Jeju, Republic of Korea; ^2^Professor, Jeju National University, Jeju, Republic of Korea

**Background:** Crisdesalazine, 2‐hydroxy‐5‐[2‐(4‐trifluoromethylphenyl)‐ethylaminobenzoic acid] (AAD‐2004), is a dual‐functional drug derived from aspirin and sulfasalazine, which are widely used to treat inflammatory diseases. The drug has an anti‐oxidative property as a potent spin‐trapping molecule and also an anti‐inflammatory effect as a microsomal prostaglandin E synthase‐1 inhibitor.

**Hypothesis:** Crisdesalazine was used as an add‐on therapy on ongoing immunosuppressive treatment, expecting the anti‐inflammatory and anti‐oxidative benefits or immunosuppressant‐sparing effect on inflammatory brain disease.

**animal:** Three dogs diagnosed with meningoencephalitis of unknown etiology (MUE) by neurologic examination, magnetic resonance imaging, and cerebral spinal fluid analysis were included.

**Methods:** Case series.

**Results:** A 4‐year, female spayed, Yorkshire terrier dog (case 1) showing head tilt, an 8‐year, male castrated, Shih‐tzu dog (case 2) showing ataxia and gait disorder, and a 4‐year, male castrated, Pomeranian dog (case 3) showing ataxia were referred to our veterinary hospital. These dogs were diagnosed with meningoencephalitis of unknown etiology (MUE) through neurologic examination, blood analysis, MRI, and CSF analysis. And the dogs had started treatment with immunosuppressants (prednisolone and cytarabine) and anti‐epileptic drugs (phenobarbital with or without levetiracetam). At least 3 months later, crisdesalazine was added to these dogs to improve clinical signs or reduce dose of immunosuppressants. After adding crisdesalazine on ongoing treatment, neurologic signs of dogs (case 1 and 3) were markedly improved. Also, in case 2, the dog could reduce the dosage of the prednisolone successfully.

**Conclusion and Clinical Relevance:** This is the first successful clinical trial case series of adding crisdesalazine on ongoing treatment for MUE dogs.

## Abstract N35: Neurofilament Light Chain as a Biomarker of Spinal Cord Diseases in Dogs

### **Taesik Yun** ^1^; Yoonhoi Koo^1^, DVM, MS, PhD; Yeon Chae^1^, DVM, MS; Dohee Lee^1^, DVM, MS; Hakhyun Kim^2^, DVM, MS, PhD; Mhan‐Pyo Yang^2^, DVM, MS, PhD; Byeong‐Teck Kang^2^, DVM, MS, PhD

#### ^1^Laboratory of Veterinary Internal Medicine, College of Veterinary Medicine, Chungbuk National University, Cheongju, Republic of Korea; ^2^Professor, Laboratory of Veterinary Internal Medicine, College of Veterinary Medicine, Chungbuk National University, Cheongju, Republic of Korea

**Background:** Neurofilament light chain (NfL) is a neuron‐specific cytoskeletal protein. Because damaged axons release NfLs into blood, NfL has been used as a biomarker for diagnosis of central nervous system diseases. General practitioners may have difficulties in distinguishing ataxia from lameness at times. Additionally, diagnosis of spinal cord diseases could be limited due to costs and concurrent disorders. Therefore, serum biomarker that can be easily measured is required.

**Objectives:** To evaluate the potential role of NfL as a biomarker for spinal cord disorders in dogs.

**Animals:** Thirty‐seven healthy dogs, 9 normal Beagle dogs, 43 dogs with intervertebral disc herniation (IVDH), 13 dogs with syringomyelia (SM), 5 dogs with fibrocartilaginous embolism, and 3 dogs with acute non‐compressive nucleus pulposus extrusion.

**Methods:** Cohort study. The levels of serum NfL were measured using single‐molecule array technology.

**Results:** There was a significant difference in the serum NfL level between the healthy dogs (12.6 pg/mL) and dogs with spinal cord disorders (91.1 pg/mL; *P* < 0.0001). The NfL level in dogs with SM (50.7 pg/mL) was significantly lower than those in dogs with IVDH (99.9 pg/mL; *P* = 0.005) and other diseases (241 pg/mL; *P* = 0.0018). The cut‐off values to differentiate from healthy dogs were 42.0 pg/mL (spinal cord disorders, sensitivity 79.7%, specificity 97.8%), 42.6 pg/mL (IVDH, sensitivity 83.7%, specificity 97.8%), and 27.5 pg/mL (SM, sensitivity 69.2%, specificity 87%).

**Conclusions and Clinical Importance:** Neurofilament light chain could be a potential biomarker for identifying spinal cord disorders and differentiating SM from other spinal cord disorders.

## Abstract NM01: A High Starch Kibble Diet Favor Body Weight Control in Neutered Cats Fed *Ad Libitum*

### **Aulus C. Carciofi** ^1^; Camila Goloni^2^; Letícia Pacheco^1^; Leticia Luis^1^; Stephanie Theodoro^1^; Lucas Scarpim^1^; Celina Torres^3^; Gener Pereira^1^

#### ^1^São Paulo State University, Sao Paulo, Brazil; ^2^Post Docotoral, São Paulo State University, Sao Paulo, Brazil; ^3^Affinity Petcare, Barcelona, Catalonia, Spain

**Background:** The “carbohydrate ceiling” theory proposes that cats may limit food intake on high starch (HS) diets, may helping to maintain a stable body weight (BW) when fed *ad libitum*.

**Hypothesis/Objectives:** To compare the *ad libitum* intake of two kibble diets with different starch to protein ratios (but similar in fat and fiber) on BW and composition (BC), and energy expenditure (EE) of privately owned cats.

**Animals:** Female (F) Non‐Obese (NO), 2.9 ± 2.1 years (*n =* 9); F Obese (OB; body condition score ≥7), 4.2 ± 1.6 years (*n =* 7); Male (M) NO, 2.0 ± 1.1 years (*n =* 9); M‐OB, 4.5 ± 2.6 years (*n =* 5).

**Methods:** In a crossover design, cats were fed *ad libitum* for 4 months a HS (starch 40%, protein 38%) or high protein (HP: starch 20%, protein 55%) kibble diet. The BC and EE were evaluated by the doubly labeled water method. Results were submitted to ANOVA in a 2 diets x 2 sex × 2 BC arrangement (*P* ≤ 0.05 significant; *P* ≤ 0.10 trend).

**Results:** Cats fed HS diet maintained constant BW (*P* > 0.05), but lean mass (LM) tended to reduce in F‐OB (*P* = 0.07; numerically lower EE and protein intake). The BW and LM of cats increased when fed the HP diet (*P* < 0.05), but also fat mass in 17% on F‐NO (*P* = 0.04) and 7% in F‐OB (*P* = 0.06). The EE tended to be higher in M (351 ± 8 kJ/kg0.67/day) than F (330 ± 8; *P* = 0.06) and decreased then cats become older (*P* < 0.01; Figure 1).

**Conclusions:** In *ad libitum* feeding system, the intake of HS diet favored a better control of the cat's BW.

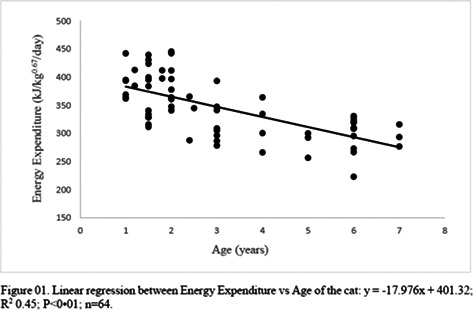

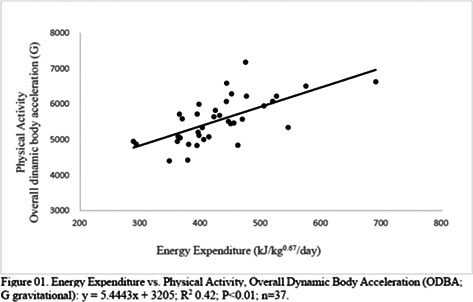

## Abstract NM02: High‐Fat Diet Enhances Stemness and Compromises Intestinal Permeability in a Translational Canine Colonoid Models

### **Itsuma Nagao** ^1,2^; Yoko Ambrosini^1^

#### ^1^Washington State University, Pullman, WA, USA; ^2^The University of Tokyo, Tokyo, Japan

**Background:** The molecular mechanisms underlying obesity‐related intestinal dysfunctions remain poorly understood due to the limited availability of relevant animal models and *in vitro* systems. However, as a spontaneous and naturally occurring large animal model of various human intestinal diseases, dogs have been increasingly recognized as valuable for advancing our understanding of such conditions.

**Hypothesis/Objectives:** To demonstrate how the canine colonoid (CC) model can be used to assess the direct impact of a high‐fat diet (HFD) on intestinal function.

**Animals:** Canine colonoids (CCs) were obtained by biopsying colonic tissues from healthy donor dogs at the Veterinary Teaching Hospital at Washington State University.

**Methods:** The HFD‐induced stem‐cell phenotype was determined in CCs after 24 h of palmitic acid (PA) exposure, a well‐established main component of the HFD, using RT‐qPCR and the EdU assay. The HFD‐induced gut epithelial integrity was determined by assessing the transepithelial electrical resistance (TEER) value after 24 h of PA exposure using colonoid‐derived intestinal monolayers. Results were evaluated by ANOVA and t‐tests.

**Results:** Significant increases in LGR5 expression (1.7 times, *P* < 0.01) as well as the size of organoids (3.0 times, *P* < 0.01) and the percentage of EdU‐positive cells (2.7 times, *P* < 0.01) were confirmed following PA exposure. In colonoid‐derived intestinal monolayers, significant decrease in TEER values (*P* < 0.01) was demonstrated following PA exposure.

**Conclusions and Clinical Importance:** We confirmed exposure to PA increased the stemness of CCs and reduced the barrier function, recapitulating the findings in dogs and humans following HFD consumption. These studies establish proof‐of‐concept that CCs can be utilized to assess the effect of early exposure of a nutrient on intestinal function.

## Abstract NM03: Investigating Differences in Energy Density and Theoretical Calorie Intake of Canadian Raw Diets for Dogs

### **Caitlin Grant** ^1^; Amrit Rooprai^2^

#### ^1^Ontario Veterinary College, Guelph, Ontario, Canada; ^2^University of Guelph, Guelph, Ontario, Canada

**Background:** Pet food labels are used to describe calorie content and feeding directions. There is no standard method for determining energy density and feeding directions (FD) of commercial raw food diets (CRFDs) vary.

**Objectives:** This study aimed to investigate the variation in pet food label information of 20 CRFDs for dogs, specifically in relation to metabolizable energy (ME) and (FD).

**Methods:** Two diets from 5 Canadian CRFD companies were randomly selected, samples of each were purchased at 2 locations (*n =* 20). Samples were submitted for analysis of ME. Analyzed ME was compared to ME reported on the label and compared between diets within companies. Theoretical calorie intake (CI) was estimated using the FD reported on each label for 4 adult weight categories: 10, 25, 50, and 100 lbs. Mean CI was compared between diets and locations of the same company.

**Results:** Factorial ANOVA and paired t‐test were performed using IBM SPSS statistics and significance was set as *P* < 0.05. Mean label ME for the 20 diets (1592.11 kcal/kg) was less than mean analyzed ME (2500.45 kcal/kg) (*P* < 0.001). Mean theoretical CI differed between diets of the same company for all companies (*P* < 0.001) (Table 1) and differed between locations for 3 of the 5 companies (*P* < 0.03).

**Conclusions and Clinical Importance:** Variation between label and analyzed ME and inconsistent FD can lead to inaccurate determination of CI and could cause fluctuation in CI if there is rotation of diets fed. Companies should re‐evaluate FD to ensure consistency between diets.

**Table:** Table 1. Mean differences in theoretical intake between diets and locations of the same company for 4 CRFDs selected from 5 Canadian per food companies (*n =* 20).

Company	Mean difference in theoretical intake between diets (diet 1‐diet 2) (kcal/kg^0.75^)	Mean difference in theoretical intake between locations (location 1‐location 2) (kcal/kg^0.75^)
A	38.558*	26.902*
B	25.484*	−12.066*
C	77.028*	23.817*
D	25.771*	9.954
E	40.370*	0.583

Supercript* = the mean difference is significant with a *P* < 0.05

## Abstract NM04: Effect of Spirulina (*Arthrospira platensis*) Supplementation in Overweight Dogs Undergoing a Weight Loss Program

### **Davide Stefanutti** ^1^; Lorenzo Serva^1^; Michele Berlanda^1^; Federico Bonsembiante^2^; Gianfranco Gabai^2^; Rebecca Ricci^1^

#### ^1^Department of Animal Medicine, Production and Health, University of Padua, Padua, Italy; ^2^Department of Comparative Biomedicine and Food Science, University of Padua, Padua, Italy

**Background:** Spirulina has shown to have hypolipidemic and antioxidant effects in rats and humans.

**Hypothesis/Objectives:** Spirulina supplementation in overweight dogs undergoing a weight loss program will result in improved serum lipid profile and antioxidant capacity.

**Animals:** Forty client‐owned neutered overweight (BCS ≥7/9) dogs were enrolled in the study.

**Methods:** Double‐blind randomized placebo‐controlled trial. Dogs received either Spirulina (S) (Livegreen Srl, Italy) or placebo (P) tablets in a body weight‐dependent amount for 12 weeks, during which both groups were fed the same calorie‐restricted diet. Dogs were weighed weekly and the calorie restriction was adjusted accordingly to ensure a 1% body weight loss per week. A blood sample was collected at baseline (T0), midpoint (T1), and endpoint (T2).

**Results:** Thirty‐two dogs completed the study in full compliance with the protocol. No difference in body weight loss was detected between groups at T2 (P: −10.6%, S: −11.9%; *P* = 0,229). After 12 weeks, in the S group average serum triglycerides significantly decreased from 104.2 ± 8.9 to 65.9 ± 8.9 mg/dl (*P* < 0.0001) and antioxidant capacity (BAP test) significantly increased from 2038 ± 155 to 2605 ± 155 μM/L (*P* = 0.027). Antioxidant capacity improved from 1988 ± 153 to 2730 ± 153 μM/L also in the P group (*P* = 0.014), whereas the decrease of triglycerides from 83.6 ± 8.7 to 65.3 ± 8.7 mg/dl was not significant (*P* = 0.28). Significant reductions of serum total cholesterol, LDL and glucose were identified at T1 already, regardless of the supplementation received.

**Conclusion and Clinical Importance:** Spirulina enhanced the hypotriglyceridemic effect observed in overweight dogs attaining 10% weight loss.

## Abstract NM05: Serum Vitamin D Metabolites and Acute‐Phase Proteins in Critically Ill Dogs and Cats

### **Hailey E. Rose** ^1^; Jared Jaffey^2^, DACVIM (SAIM); John Loftus^3^, DACVIM (SAIM, Nutrition), PhD

#### ^1^Cornell University, Ithaca, NY, USA; ^2^Assistant Professor, Internal Medicine, Midwestern University, Glendale, AZ, USA; ^3^Assistant Professor, Internal Medicine, Cornell University, Ithaca, NY, USA

**Background:** Serum vitamin 25‐hydroxyvitamin D (25(OH)D) decreases occur throughout hospital stays in people. Several veterinary studies found that decreased blood 25(OH)D levels were associated with mortality.

**Hypothesis/Objectives:** Low serum 25(OH)D and 1,25(OH)2D concentrations are associated with mortality and negatively correlate with acute‐phase protein (APP) in ill dogs and cats admitted to nursing care units.

**Animals:** Client‐owned dogs (*n =* 77) and cats (*n =* 17) admitted to two academic veterinary hospital nursing care units.

**Methods:** A diagnostic lab measured 25(OH)D, 1,25(OH)2D, and haptoglobin in dogs and cats, C‐reactive protein (CRP) in dogs, and serum amyloid A (SAA) in cats. Serum was collected within 12 h of admission. Illness severity (APPLEfast) scores and survival data were recorded. Cox proportional hazard and correlation matrix (Spearman r) analyses were applied, and *P* < 0.05 established significance.

**Results:** Mortality hazard ratios were approximately 1 for 25(OH)D, 1,25(OH)2D, and APP. In dogs, haptoglobin negatively correlated with 25(OH)D (*P* = 0.004, *r =* −0.38) and 1,25(OH)2D (*P* = 0.001, *r =* −0.28). APPLEfast scores positively correlated with CRP (*P* = 0.001, *r =* 0.32). In cats, APPLEfast scores negatively correlated with 1,25(OH)2D (*P* = 0.040, *r =* −0.51). SAA was negatively correlated with 25(OH)D (*P* = 0.027, *r =* −0.2).

**Conclusions and Clinical Importance:** Serum 25(OH)D or 1,25(OH)2D were not associated with mortality in our mixed nursing care population. Relationships between APP and serum vitamin D metabolites with APPLEfast scores in cats suggest promise as illness severity biomarkers that warrant further investigation.

## Abstract NM06: Differential Digestive Health Benefits of Fiber Blends Fed to Healthy Adult Cats

### **Julie K. Spears** ^1^; Alison Beloshapka^2^, PhD

#### ^1^Nestle Purina PetCare Company; ^2^Principal Nutritionist, Global Nutrition and Claims Development, Nestle Purina PetCare Company

**Background:** Dietary fibers and prebiotics confer health benefits to pets. Because they have variable fermentation rates and soluble:insoluble composition, fiber blends may better improve digestive health parameters. Health benefits may be differential for dogs and cats depending on their anatomy and gut microbiome to ferment dietary fibers and prebiotics.

**Hypothesis/Objectives:** To determine if fiber blends provide a digestive health benefit to cats.

**Animals:** Thirty‐two colony‐housed adult cats were recruited for randomized controlled feeding study. All cats were apparently healthy and had not received antibiotics for at least 60 days prior to study start.

**Methods:** To stabilize the intestinal microbiome, a low‐fiber control diet was fed for 28 days. Following stabilization, cats were randomized to 1 of 4 test diets for an additional 28 days: control, blend 1 (inulin, psyllium, pumpkin), blend 2 (inulin, oat fiber, beet pulp, pumpkin, citrus fiber), or blend 3 (inulin, wheat aleurone, pumpkin). Biological samples were collected to evaluate the impact of fibers on digestive and immune health. Parameters were compared to performance on the low‐fiber control using paired t‐tests.

**Results:** Test diets were highly digestible. No differences were noted following blend 1 consumption. Blend 2 decreased *Streptococcus* spp. (*P* < 0.05). Blend 3 consumption increased *Bifidobacterium* spp. and *Actinobacteria* spp. relative abundance while decreasing total branched‐chain fatty acids, isovalerate, dysbiosis index, and *Streptococcus* spp. (*P* < 0.05).

**Conclusions and Clinical Importance:** Fiber blends performed differently when fed *in vivo*. Cats consuming the blend of inulin, wheat aleurone, and pumpkin had an improved gastrointestinal environment.

## Abstract NM07: Differential Digestive Health Benefits of Fiber Blends Fed to Healthy Adult Dogs

### **Julie K. Spears** ^1^; Alison Beloshapka^2^, PhD

#### ^1^Nestle Purina PetCare Company; ^2^Principal Nutritionist, Global Nutrition and Claims Development, Nestle Purina PetCare Company

**Background:** Dietary fibers and prebiotics confer health benefits to pets. Because fibers have different fermentation rates and soluble:insoluble ratios, blends may better improve digestive health parameters. Health benefits may be differential for dogs and cats depending on their ability to ferment dietary fibers and prebiotics.

**Hypothesis/Objectives:** To determine if fiber blends provide a digestive health benefit *in vivo*.

**Animals:** Thirty‐two colony‐housed dogs were recruited for a randomized controlled feeding study. All dogs were apparently healthy and had not received antibiotics for at least 60 days prior to test start.

**Methods:** To stabilize the intestinal microbiome, a low‐fiber control diet was fed for 28 days minimum. Following stabilization, dogs were randomized to 1 of 4 test diets for an additional 28 days: control, blend 1 (inulin, psyllium, pumpkin), blend 2 (inulin, oat fiber, beet pulp, pumpkin, citrus fiber), or blend 3 (inulin, aleurone, pumpkin). Biological samples were collected to evaluate the impact of fiber blends on digestive and immune health. Parameters were compared to performance on the low‐fiber control using paired t‐tests.

**Results:** Test diets were highly digestible. Blend 1 increased fecal IgA while decreasing total branched‐chain fatty acids, *Clostridium perfringens*, *E. coli*, and *Streptococcus* spp. (*P* < 0.05). Blend 2 increased *Bifidobacterium* spp. and *Turicibacter* spp. (*P* < 0.05). Blend 3 increased *Bifidobacterium* spp. and *Lactobacillus* spp. (*P* < 0.05).

**Conclusions and Clinical Importance:** While all fiber blends performed well, dogs consuming the blend of inulin, psyllium, and pumpkin had improved the gastrointestinal environment.

## Abstract NM08: Evaluation of Pumpkin and Inulin Using Canine‐ and Feline‐Specific Intestinal *In Vitro* Fermentation Assays

### **Julie K. Spears** ^1^; Alison Beloshapka^2^, PhD

#### ^1^Nestle Purina PetCare Company; ^2^Principal Nutritionist, Global Nutrition and Claims Development, Nestle Purina PetCare Company

**Background:** Digestive health benefits of fibers and prebiotics are dependent on host microbiome fermentation ability. *In vitro* fermentation can be used to predict ingredients that provide a health benefit and reduce the number of animal tests conducted.

**Hypothesis/Objectives:** To use species‐specific intestinal *in vitro* fermentation studies to determine potential digestive health benefits of the combination of pumpkin and inulin.

**Animals:** Fresh fecal inoculum was collected from colony‐housed healthy dogs and cats.

**Methods:** Fibers evaluated were inulin, pumpkin, and the combination of pumpkin and inulin (PI). *In vitro* fermentation studies were conducted under anaerobic conditions. Incremental measures of microbial fermentation and metabolic activity were evaluated following 12 h of fermentation.

**Results:** An indication of fermentation (pH decrease) was lowest for inulin and PI for both species. Bifidobacteria and *Lactobacillus* were increased with all substrates, with the largest increase with PI in both species. Total short‐chain fatty acids were increased with all substrates for canine, the largest increase was observed for PI. Acetate, butyrate, and total short‐chain fatty acids were increased with pumpkin and PI for feline. Total branched‐chain fatty acids were decreased with pumpkin and PI for canine and all substrates for feline, with the largest decrease with PI.

**Conclusions and Clinical Importance:** In dogs, inulin and pumpkin improved digestive health measures when evaluated individually; however, the combination of the two performed best. In cats, pumpkin and PI improved digestive health measures. Bacteria shifts were greatest with PI for both species.

## Abstract NM09: Supplementation of Probiotics With Psyllium Influences the Gut Microbiome Through Microbial Diversity Alteration in Dogs

### **Julie K. Spears** ^1^; Alison Beloshapka^2^, PhD; Jacob Witkop^1^; Arleigh Reynolds^1^

#### ^1^Nestle Purina PetCare Company; ^2^Principal Nutritionist, Global Nutrition and Claims Development, Nestle Purina PetCare Company

**Background:** Synbiotics are a mixture of live microorganisms and a substrate that confers a health benefit on the host. The combination of psyllium and *Enterococcus faecium* SF68 as a synbiotic has not been evaluated previously in companion animals.

**Hypothesis/Objectives:** To determine if the addition of psyllium to *E. faecium* SF68 has a beneficial effect on microbiome health through the evaluation of microbial diversity in dogs.

**Animals:** Forty‐eight healthy adult sled dogs were supplemented daily with a probiotic (*E. faecium* SF68 5 × 10^8^ CFU/g) or synbiotic (*E. faecium* SF68 5 × 10^8^ CFU/g plus 1 g psyllium) for 28 days (*n =* 24/group).

**Methods:** Fresh fecal samples were collected prior to supplementation (baseline) and at the end of supplementation for microbiome analysis. Principal components analysis was performed on CLR‐transformed counts, and PERMANOVA was performed on Aitchison distances (Euclidean distances based on CLR‐transformed counts). Relationships between principal components and treatment groups were explored using linear mixed‐effects models controlling for random effects of each dog.

**Results:** Permutational multivariate analyses of variance (PERMANOVA) revealed that dogs assigned to the probiotic and synbiotic groups did not significantly differ in fecal microbial community composition at baseline (*P* = 0.598) but did by the end of 28 days of feeding (*P* = 0.012).

**Conclusions and Clinical Importance:** Psyllium husk fiber has been observed to be moderately fermented by dogs, resulting in changes in gut microbial composition. The beneficial role of intestinal microbiota and a balanced microbiome in maintaining the health of dogs can be strengthened by the supplementation of the synbiotic *E. faecium* SF68 and psyllium.

## Abstract NM10: The Tolerability of Supplementing CBD at Two Doses for 9 Months to Healthy Dogs

### **Isabella Corsato Alvarenga** ^1^; Hannah Hess^2^; Kim Wilson^3^; Stephanie McGrath^4^, DVM, MS, DACVIM

#### ^1^Colorado State University, Fort Collins, CO, USA; ^2^DVM Student, Clinical Sciences, Colorado State University, Fort Collins, CO, USA; ^3^Hill's Pet Nutrition; ^4^Associate Professor, Clinical Sciences, Colorado State University, Fort Collins, CO, USA

**Background:** Cannabidiol (CBD) has gained recognition for its therapeutic potential in companion animals. Dog owners have been using CBD mostly for neurological and inflammatory conditions, although long‐term tolerability has not been established. Short‐term studies have determined CBD is well tolerated with mild side effects and an elevation in alkaline phosphatase.

**Hypothesis/Objectives:** Determine the long‐term tolerability of oral CBD administered to healthy dogs.

**Animals:** Eighteen healthy adult research dogs were randomly assigned to three groups (*n =* 6) that received either 0, 5, or 10 mg/kg/day CBD.

**Methods:** After adapting for 3 weeks, dogs received 9 months oral treatment once daily postprandially. Adverse events were recorded daily. Monthly blood profiles, postprandial bile acids, and plasma CBD at fasting state and 2 h post dosing were measured. Data were analyzed as repeated measures over time using a mixed model, with significance at *α* = 0.05.

**Results:** The 0 and 5 mg/kg had similar fecal scores. Although 10 mg/kg had greater incidence of soft stools, no other significant AEs were noted. An elevation (*P* < 0.0001) in ALP occurred in groups that received CBD (61.4, 284.5, and 373.5 IU/L in 0, 5, and 10 mg/kg, respectively). Remaining blood parameters were within reference range. Average plasma CBD at 5 and 10 mg/kg were 97.3 ng/mL and 236.8 ng/mL preprandial, 340.8 ng/mL and 1067.7 ng/mL postprandial, respectively. A cumulative effect at fasting state over time occurred (*P* = 0.0122).

**Conclusions and Clinical Importance:** Chronic administration of CBD at 5 mg/kg was better tolerated than at 10 mg/kg, and both doses caused elevated ALP.

638

## Abstract NM11: Comparison of Microorganisms Concentration of Brazilian Probiotics and Dose Recommendation for Canine Enteropathies

### **Ana Rita C. Pereira** ^1^, DVM; Renan da Silva^2^, DVM; Patricia Eri Ishii^3^, PhD; Ricardo Duarte^4^, DVM, MS, PhD; Fabio Teixeira^5^

#### ^1^School of Veterinary Medicine and Animal Science, University of São Paulo, São Paulo, Brazil; GastroVet; ^2^Faculty of Veterinary Medicine and Animal Science, University of São Paulo, São Paulo, Brazil; ^3^Stonewell; ^4^GastroVet; All Care Vet; University Center of the United Metropolitan Colleges, São Paulo, Brazil; ^5^Anclivepa Veterinary College, São Paulo, Brazil

**Background:** Intestinal microbiota plays an important role in canine enteropathies. Probiotics are often used as an adjuvant treatment in dogs with acute and chronic enteropathy. Several studies suggest an optimal concentration for probiotics; however, there is no literature comparing the dosages of probiotics available in the market.

**Objectives:** To evaluate the concentration and dose recommendation of microorganisms listed on the label of probiotic products sold for dogs in Brazil, with the doses recommended in the literature.

**Animals:** None.

**Methods:** Evaluation of microorganisms, concentration per strain (cfu/g) and the global amount of microorganisms (cfu/g) of probiotics available in Brazil and compared with the average dose (cfu/day) used in the studies that suggested beneficial effect of probiotics in dogs with enteropathies.

**Results:** 18 probiotics were included in the study. *E. faecium* (range: 1.66 × 10^5^ to 3.5 × 10^9^ cfu/g) and *L. acidophilus* (range: 3.33 × 10^5^ to 8.6 × 10^9^ cfu/g) were the most frequent, found in 16 of 18 products. The global average of microorganisms was 2.55 × 10^9^ cfu/g (1.33 × 10^6^ to 25 × 10^10^). From the 22 studies that suggested improved gastrointestinal clinical signs from probiotics the average global dose recommended was 7.83 × 10^10^ cfu/dog/day (1.5 × 10^7^ to 4.5 × 10^11^). In Brazilian probiotics the average daily amount to reach the minimum study doses were 0.94 g/dog (range: 0.001‐11; mean: 0.015), average study dose 4897 g/dog (3.5‐58818; 77 g/dog), and maximum 28149 g/dog (20‐338092; 445 g/dog).

**Conclusions:** There is wide variation in the concentration of microorganisms declared on the labels of Brazilian probiotic products. Reaching the mean dose used in studies that showed improvement in dogs with enteropathies is not feasible.

## Abstract NM12: Enzyme‐Linked Immunosorbent Assay for the Detection of Phytohemagglutinin in Legumes

### **Darcy B. Adin** ^1^; Thomas Sewell^2^, BS; Titan Fan^3^, PhD; Kate Hourihan^4^

#### ^1^University of Florida, Gainesville, FL, USA; ^2^Senior Scientist, Beacon Analytical Systems, Inc, Saco, ME, USA; ^3^President, Beacon Analytical Systems, Inc, Saco, ME, USA; ^4^Manufacturing Manager, Manufacturing, Beacon Analytical Systems, Inc, Saco, ME, USA

**Background:** PHA is a lectin found in kidney beans and other legumes that is toxic if improperly processed during food manufacturing. PHA consists of two closely related subunits, PHA‐E and PHA‐L. The E and L subunits are responsible for erythroagglutination (red blood cell aggregation) and leucoagglutination (white blood cell aggregation) respectively. Increased levels of PHA‐E originating from legumes in grain‐free (GF) dog foods could theoretically cause DCM from red cell agglutination causing cardiac muscle strain. Legume‐rich, GF dog foods are in the process of being evaluated for lectins.

**Objective:** We sought to develop an ELISA to measure levels of phytohemagglutinin (PHA) in legumes that are used in GF dog foods.

**Method:** For the development of the ELISA, PHA‐E analyte was coated onto 96 well microtiter plates. Antibody against the analyte was produced through the immunization of rabbits. Thirty‐two legume samples were ground up and extracted in a 0.9% NaCl buffer to test for PHA‐E by ELISA. The standard curve of the assay ranges from 1 to 100 ppm.

**Results:** The 2 samples yielding the highest PHA‐E levels were red kidney bean at 35,204 ppm and dark kidney bean at 14,480 ppm. Samples with medium PHA‐E levels; yellow eyed peas at 4,676 ppm and navy beans at 1290 ppm. Various lentils tested had lower results ranging between 1 and 197 ppm.

**Conclusion:** The ELISA developed in this study could be used to measure PHA‐E in food which might contribute to our understanding of DCM associated with legume‐rich GF dog foods.

## Abstract NM13: Circulating miRNAs Show an Anti‐inflammatory Profile in Healthy Canines Fed Low Carbohydrate Ketogenic Foods

### **Selena K. Tavener** ^1^; Matthew Jackson^2^, PhD; Kiran Panickar^3^, PhD

#### ^1^Science and Technology Center, Hill's Pet Nutrition; ^2^Senior Principal Scientist, Science and Technology Center, Hill's Pet Nutrition; ^3^Manager, Science and Technology Center, Hill's Pet Nutrition

**Background:** Ketogenic foods contain high fat but limit the amount of digestible carbohydrate and generally have anti‐inflammatory effects. Circulating microRNAs (miRNAs) are important biomarkers of biological processes including inflammation.

**Hypothesis:** Circulating miRNAs in canines fed LoCHO_FAT and LoCHO_PROT diets have an anti‐inflammatory effect.

**Animals:** Healthy canines (*n =* 10) housed in the Hill's colony fed two low‐carbohydrate (LoCHO) foods varying in protein (LoCHO_PROT) and fat (LoCHO_FAT) and compared to a control food (HiCHO).

**Methods:** Canines randomized to two groups in a crossover design and fed control food high in carbohydrate (HiCHO) followed by LoCHO_PROT then LoCHO_FAT. The other group was fed the same control food followed by LoCHO foods in reverse order (LoCHO_FAT then LoCHO_PROT). Gene expression for miRNAs from blood collected in PAXgene tubes was assessed and analyzed using Qiagen's miRNA PCR array platform.

**Results:** When compared to HiCHO there was a significant increase in cfa‐miR‐106a, cfa‐miR‐15a, cfa‐miR‐15b, cfa‐miR‐16, cfa‐miR‐20a, cfa‐miR‐21, cfa‐miR‐148a, and cfa‐miR‐195 in LoCHO_FAT (*P* < 0.05) but not LoCHO_PROT. miR‐106a and other miRNAs have been reported to have anti‐inflammatory effects by attenuating the production of pro‐inflammatory cytokines possibly through inhibiting the NFκB and the MAPK pathway. miRNAs upregulated in LoCHO_PROT and LoCHO_FAT include cfa‐miR‐200c, cfa‐let‐7a, cfa‐let‐7c, cfa‐let‐7f, and cfa‐let‐7g (all *P* < 0.05 versus CON). Let‐7 miRNAs have anti‐inflammatory effects. For instance, let‐7f can attenuate inflammation by targeting NLRP3, an inflammasome.

**Conclusions and Clinical Importance:** LoCHO_PROT and LoCHO_FAT foods might be important as part of immune‐modulating therapeutic nutritional strategies to reduce inflammation or inflammatory pathways in canines.

## Abstract O01: Baseline Tumor Gene Expression Signatures Correlate with Chemoimmunotherapy Treatment Responsiveness in Canine B Cell Lymphoma

### **Katie Dittrich** ^1^; Ummugulsum Yildiz‐Altay^2^, MD; Fatima Qutab^2^; Danny Kwong^2^; Rao Zechuan^2^; Sebastian Nievez‐Lozano^2^; Heather Gardner^1^, DVM, PhD, DACVIM; Jillian Richmond^2^, PhD; Cheryl London^1^, DVM, PhD, DACVIM

#### ^1^Cummings School of Veterinary Medicine, Tufts University, Medford, MA, USA; ^2^Chan Medical School, University of Massachusetts, Worcester, MA, USA

**Background:** Chemoimmunotherapy including an anti‐CD20 monoclonal antibody is the standard of care in human diffuse large B cell lymphoma (DLBCL). An increased knowledge of canine DLBCL biology has led to a resurgence in efforts to incorporate novel agents into veterinary chemoimmunotherapy treatment protocols. However, it is unknown whether stratification of patients into treatment regimens based on a molecular profile will predict treatment outcome.

**Hypothesis/Objectives:** We hypothesized that differentially expressed gene (DEG) signatures in the pre‐treatment lymph node aspirates of dogs with DLBCL would directly correlate with clinical response to chemoimmunotherapy treatment.

**Animals:** Eighteen dogs were enrolled as part of a prospective, non‐randomized clinical trial evaluating the use of combination doxorubicin, anti‐CD20 monoclonal antibody, and one of three small molecule inhibitors: KPT‐9274, TAK‐981, or RV1001.

**Methods:** We evaluated gene expression in RNA extracted from pre‐treatment lymph node aspirates using the NanoString nCounter Canine Immuno‐Oncology Panel. Samples were compared within each treatment group based on early (<90 days) or late (>90 days) progression‐free survival (PFS). Trends for selected candidate biomarker genes were confirmed using qPCR.

**Results:** Prognostic DEGs clustered within each treatment arm, including CREBBP, CDKN1A, TLR3, PI3Kδ, AKT3, PTEN, and NRAS. The presence of pathway alterations downstream of the inhibitor received were associated with PFS.

**Conclusions and Clinical Importance:** Our findings emphasize the transcriptomic heterogeneity of canine DLBCL and provide a blueprint for validation of clinically relevant biomarkers in prospective chemoimmunotherapy studies.

## Abstract O02: The Immune Landscape of Canine Soft Tissue Sarcomas

### **Regina Hayburn** ^1^; Juliana Chi Kei Ng^2^; Xinlei Mi^2^, PhD; Tasha Miller^3^; Weiqing Jing^2^, PhD; Christine Chang^3^, DVM; Emma Warry^3^, BVSc (Hons), MS, DACVIM (Oncology); Seth Pollack^2^, MD; Shay Bracha^4^, DVM, MS, DACVIM (Oncology)

#### ^1^Colorado State University, Fort Collins, CO, USA; ^2^Northwestern University, Evanston, IL, USA; ^3^Texas A&M University, Collage Station, TX, USA; ^4^The Ohio State University, Columbus, OH, USA

**Background:** Soft tissue sarcomas (STSs) are a heterogeneous group of tumors of mesenchymal origin with similar biological behavior. These tumors comprise 10–15% of solid malignant tumors in canines, and have up to 30% mortality rate. The aim of this study was to evaluate the immune make up of soft tissue sarcomas in canines and identify its correlation with tumor stage and patient outcome. We hypothesized that tumors exhibiting an immune competent landscape are more likely to be associated with less aggressive biological behavior and a better prognosis.

**Methods:** A retrospective study of 74 client owned dogs from a single institution with confirmed diagnosis of STSs was conducted. Patient information, clinical data and outcomes were collected and analyzed. In addition, correlated tumor samples were subjected to immunohistochemistry and nanostring analysis, to identify immune components that are associated with overall survival (OS), progression free survival (PFS), and metastatic risk (MTR).

**Results:** We have identified a comprehensive and significant correlation between immunosuppressive gene signatures and patient outcome. Genes such as AXL, LFNG, MCAM and TAL1 had all exhibited a significant statistical correlation with decreased OS, PFS, and MTR. Furthermore, we have identified distinct immune contextures that included tumors of high, low, or marginal immune cell infiltrations.

**Conclusion:** This study highlights the significance of the tumor immunity for disease progression and patient's outcome. In addition, the detection of potential disease markers and novel targets will promote the design of future immunotherapies for dogs with soft tissue sarcomas.

## Abstract O03: Vincristine‐Induced Adverse Events as Related to Body Weight in Dogs Treated for Lymphoma

### **Keira Sztukowski** ^1^; Zachary Yaufman^2^, DVM; Matthew Cook^3^, VMD, MS, DACVIM (Oncology); Turi Aarnes^1^, DVM, MS, DACVAA; Brian Husbands^1^, DVM, DACVIM (Oncology)

#### ^1^College of Veterinary Medicine, The Ohio State University, Columbus, OH, USA; ^2^The Ohio State University, Columbus, OH, USA; ^3^Metropolitan Veterinary Hospital, Akron, OH, USA

**Background:** Traditional dosing of chemotherapy based on body surface area (BSA) may overdose small dogs leading to increased incidence of adverse events (AEs).

**Hypothesis/Objectives:** Objectives were to evaluate the incidence of hematologic and gastrointestinal AEs in dogs with naïve high‐grade lymphoma treated with vincristine weighing ≤15 kg in comparison to dogs weighing >15 kg. We hypothesized that dogs weighing ≤15 kg would experience a higher incidence of AEs.

**Animals:** One‐hundred thirty‐eight chemotherapy‐naive dogs with high‐grade lymphoma treated with vincristine at standard doses.

**Methods:** Multi‐center retrospective study reviewing hematologic data and medical record information of dogs diagnosed with naïve lymphoma treated with vincristine. Complete blood counts were measured 24‐h before vincristine administration, then between 4 and 8 days post administration. Data were evaluated using logistic regression or ordinal logistic regression.

**Results:** Thirty‐eight dogs weighing ≤15 kg and 100 dogs weighing >15 kg were included. Seventeen (12.3%) instances of neutropenia occurred with no significant difference in overall incidence or grade between groups (*P* = 0.85). Thirty initially asymptomatic, substage A dogs (29.4%) experienced gastrointestinal AEs with no significant difference in overall incidence or grade between groups (*P* = 0.46). Seven instances of hospitalization occurred (5.0%) and the risk of hospitalization did not significantly differ between groups (*P* = 0.37).

**Conclusions and Clinical Importance:** Vincristine dosed using BSA does not increase risk of either hematologic or gastrointestinal AEs in dogs weighing ≤15 kg. Clinicians can feel safe using standard doses of vincristine in dogs ≤15 kg as the induction agent for lymphoma.

## Abstract O04: Tolerability and Efficacy of Adjuvant Temozolomide and Doxorubicin Combination Chemotherapy for Canine Splenic Hemangiosarcoma

### **Skylar R. Sylvester** ^1^; Alexandra Yiambilis^2^; Kelly Hume^3^, DVM, DACVIM (Oncology); Cheryl Balkman^4^, DVM, DACVIM (SAIM, Oncology)

#### ^1^College of Veterinary Medicine, Cornell University, Ithica, NY, USA; ^2^Veterinary Student, College of Veterinary Medicine, Cornell University, Ithica, NY, USA; ^3^Associate Professor, Clinical Sciences, College of Veterinary Medicine, Cornell University, Ithica, NY, USA; ^4^Clinical Professor, Clinical Sciences, College of Veterinary Medicine, Cornell University, Ithica, NY, USA

**Background:** Canine splenic hemangiosarcoma has an aggressive biologic behavior and short survival is common with standard‐of‐care splenectomy and adjuvant anthracycline chemotherapy.

**Objectives:** To evaluate tolerability and efficacy of adjuvant oral temozolomide in combination with standard‐of‐care treatment for canine splenic hemangiosarcoma.

**Animals:** 18 client‐owned dogs with splenic hemangiosarcoma.

**Methods:** A prospective single‐arm clinical trial was performed with 9 dogs enrolled to receive adjuvant doxorubicin‐temozolomide chemotherapy (stage 1, *n =* 1; 2, *n =* 5; 3, *n =* 3) and compared to 9 contemporaneous control dogs that received single‐agent doxorubicin (stage 1, *n =* 2; 2, *n =* 4; 3, *n =* 3). Doxorubicin (30 mg/m^2^ or 1 mg/kg) was administered intravenously every 21 days, followed by oral temozolomide (60 mg/m^2^) for 5 consecutive days.

**Results:** Median time to chemotherapy after splenectomy was 15 days (range 14–32). A median of 3 doxorubicin‐temozolomide cycles were completed (range 1–5). Six dogs experienced grade 1 or 2 gastrointestinal toxicity, with grade 1 hyporexia most common (*n =* 3). One episode of grade 3 anorexia was observed. Grade 1 hematologic toxicity occurred in 7 dogs, including anemia (*n =* 4), neutropenia (*n =* 5), and thrombocytopenia (*n =* 1). Three dogs required a doxorubicin dose reduction, but none in temozolomide. Median progression‐free survival was 98 days (95% CI 50–218) and median overall survival was 103 days (95% CI 50–218) in the doxorubicin‐temozolomide group compared to 127 days (95% CI 22–222) and 156 days (95% CI 51–236) in the control group (*P* = 0.723, *P* = 0.530).

**Conclusions:** Results suggest that doxorubicin‐temozolomide combination chemotherapy is tolerated, but not more efficacious than adjuvant doxorubicin in prolonging survival of dogs with splenic hemangiosarcoma.

## Abstract O05: Tolerability Evaluation of Combination Verdinexor (LAVERDIA‐CA1) and Doxorubicin in Tumor‐bearing Dogs

### **Joelle M. Fenger** ^1^, DVM, PhD, DACVIM; Jacob Cawley^2^, DVM, MS, DACVIM (Oncology); Samuel Stewart^3^, DVM, DACVECC; David Bruyette^4^, DVM, DACVIM (SAIM); Chand Khanna^5^, DVM, PhD, DACVIM (Oncology), DACVP (Hon)

#### ^1^Ethos Discovery, Ethos Veterinary Health, Columbus, OH, USA; ^2^Postdoctoral Fellow, Department of Genetics and Genomic Sciences, Case Comprehensive Cancer Center, School of Medicine, Case Western Reserve University, Cleveland, OH, USA; ^3^Director of Ethos Science Consultancy, Ethos Veterinary Health Science Consultancy, Ethos Discovery, Woburn, MA, USA; ^4^Chief Medical Officer, Anivive Lifesciences, Long Beach, CA, USA; ^5^President, Chief Science Officer, Ethos Discovery, Ethos Veterinary Health, Chevy Chase, MD, USA

**Background:** Verdinexor is a selective inhibitor of nuclear export (SINE) conditionally approved by the FDA for treatment of lymphoma. SINE compounds have demonstrated encouraging results in *in vitro* preclinical studies when combined with cytotoxic chemotherapeutics; however, understanding the tolerability of combination strategies in tumor‐bearing dogs is necessary before therapeutic activity can be assessed.

**Hypothesis/Objectives:** The primary objective is to define tolerability of verdinexor given concurrently with doxorubicin over a 42‐day exposure period. Secondary objectives include evaluation of chronic tolerability of this combination over a 120‐day period.

**Animals:** 20 client‐owned dogs with spontaneous malignancy.

**Methods:** This was a single‐arm, rolling six interpatient dose escalation trial. Dogs were administered doxorubicin (30 mg/m^2^ or 1 mg/kg for BW <15 kg) IV on day 0 and continued every 3 weeks. Verdinexor was started on day 7 at 1.0 mg/kg PO twice weekly, pending tolerability to doxorubicin and escalated or de‐escalated at fixed dose increments according to rolling six design. Adverse events (AEs) were recorded/graded according to VCOG‐CTCAE v2.0.

**Results:** Verdinexor dose regimens began at 1.0 mg/kg PO twice weekly, with increments of 0.25 mg/kg, to a maximum dose of 1.5 mg/kg. Dose‐limiting toxicities were hematologic and gastrointestinal, with the maximal tolerated dose of verdinexor determined to be 1.25 mg/kg twice weekly when given concurrently with doxorubicin. No unexpected toxicities occurred. Incidence of AEs was not increased beyond that expected with single‐agent doxorubicin.

**Conclusions:** Combination verdinexor and doxorubicin was well‐tolerated and warrants further investigation to characterize whether efficacy is enhanced in this setting.

## Abstract O06: Tumor Necrosis Factor Stimulated Gene 6 Intrinsically Regulates PD‐L1 Expression, Modulating the Breast Cancer Microenvironment.

### **Jeong‐Hwa Lee** ^1^; Ju‐Hyun An^2^; Hwa‐Young Youn^1^

#### ^1^Seoul National University, Seoul, Seoul‐t'ukpyolsi, Republic of Korea; ^2^Kangwon National University, Chuncheon, Gangwon‐do, Republic of Korea

**Background:** Recent studies have shown that tumor cells express tumor necrosis factor‐inducible gene 6 (TSG‐6) and its protein, which is known to play a key role in regulating excessive immune responses and proliferation and growth of mesenchymal stem cells (MSCs).

**Hypothesis/Objectives:** This study aimed to determine whether inhibition of TSG‐6 on tumor cells could inhibit the growth of tumor cells and modulate the activation of immune cells in the tumor microenvironment (TME).

**Methods:** TSG‐6‐specific small interfering RNA was transfected into canine and human breast cancer cells (CIPp, CIPm and BT‐20).

**Results:** TSG‐6‐down‐regulated (siTSG‐6) cells showed decreased cell proliferation, migration, and invasion abilities. Decreased mRNA expressions of NF‐κB, STAT3, and Sox2, confirming that TSG‐6 is an upper factor governing tumor growth and metastasis. Notably, siTSG‐6 cells showed significantly decreased expression levels of CD44 and PD‐L1. Direct and indirect co‐culture of canine peripheral blood mononuclear cells (cPBMCs) and the siTSG‐6 cells showed significant activation in M1 type macrophages and cytotoxic T cells. They also showed a tendency to decrease in the expression of CTLA‐4 and increase in the expression of PD‐1.

**Conclusion and Clinical Importance:** This study suggests that the down regulation of TSG‐6 in breast cancer cells could not only suppress tumor growth and metastasis but also regulate TME. Since modulation of immune checkpoint proteins occurs in both tumor cells and immune cells, inhibiting TSG‐6 and its protein within the TME could be novel therapeutic target for anticancer treatment.

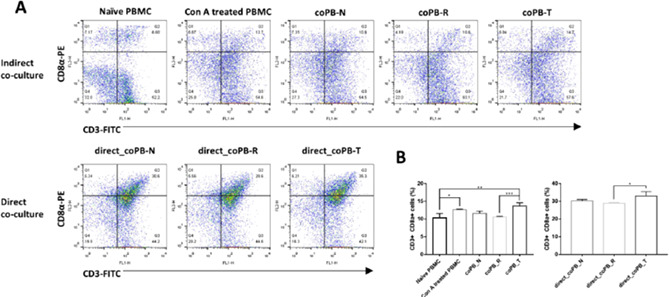

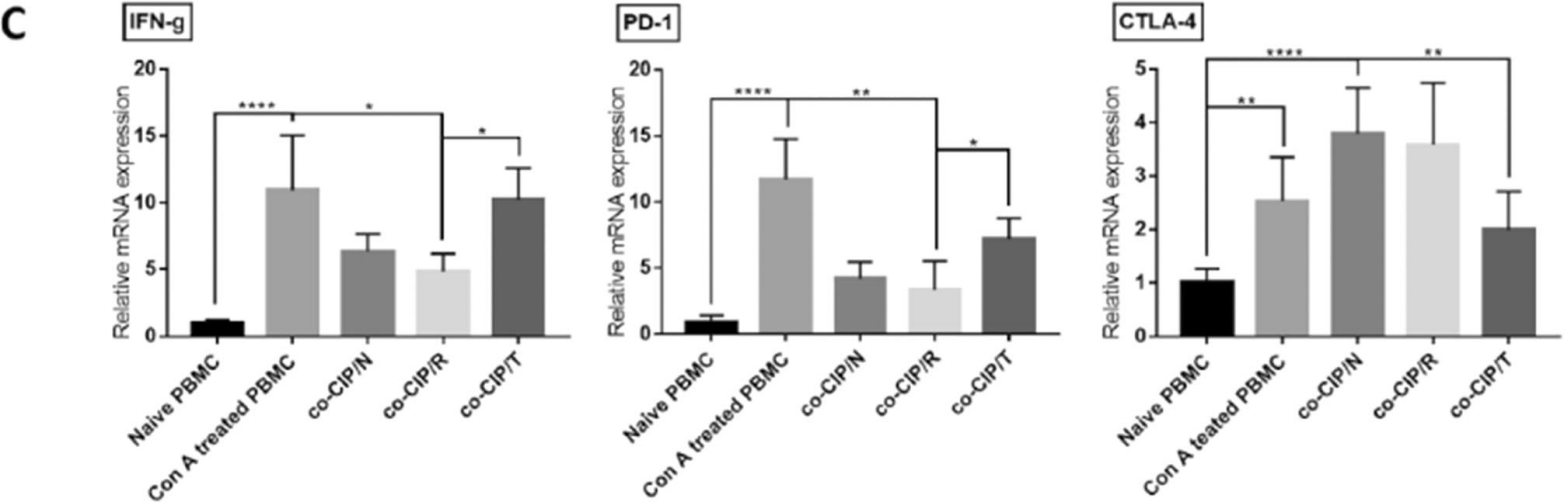

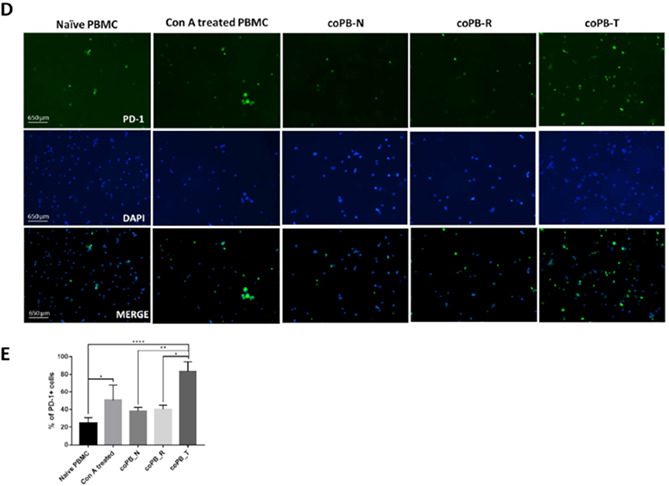

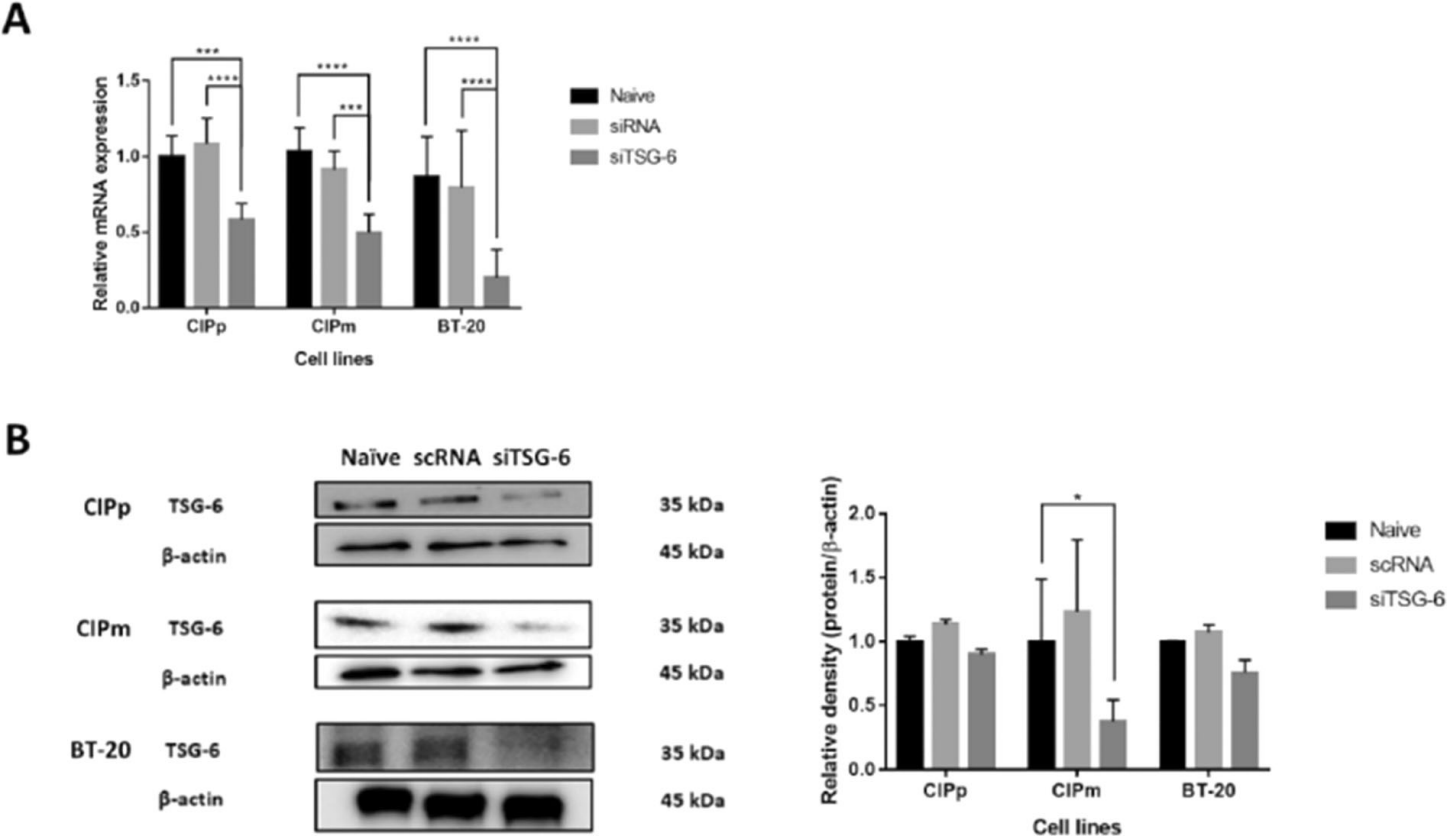

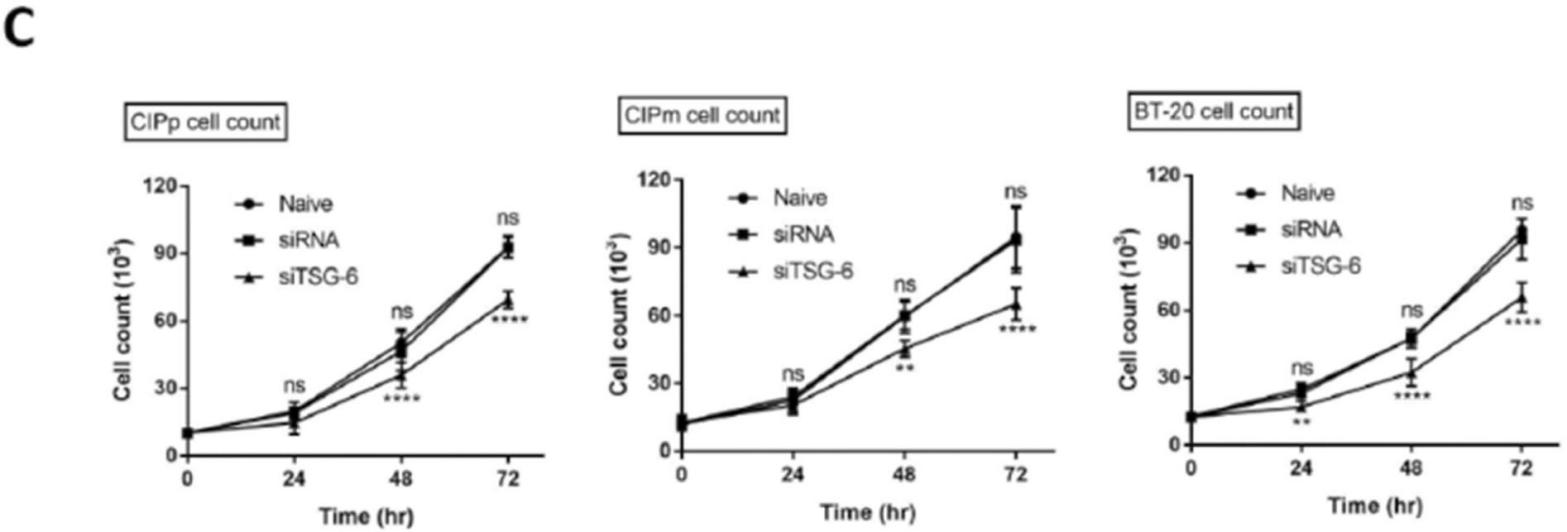

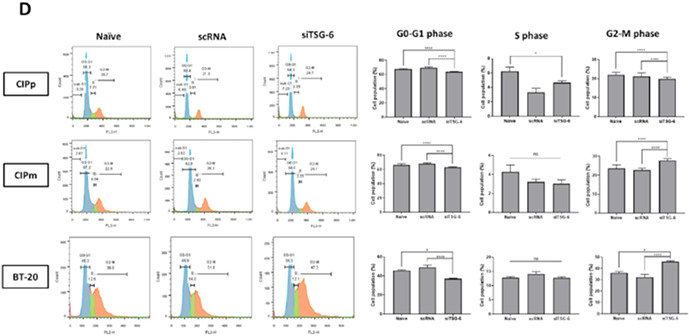

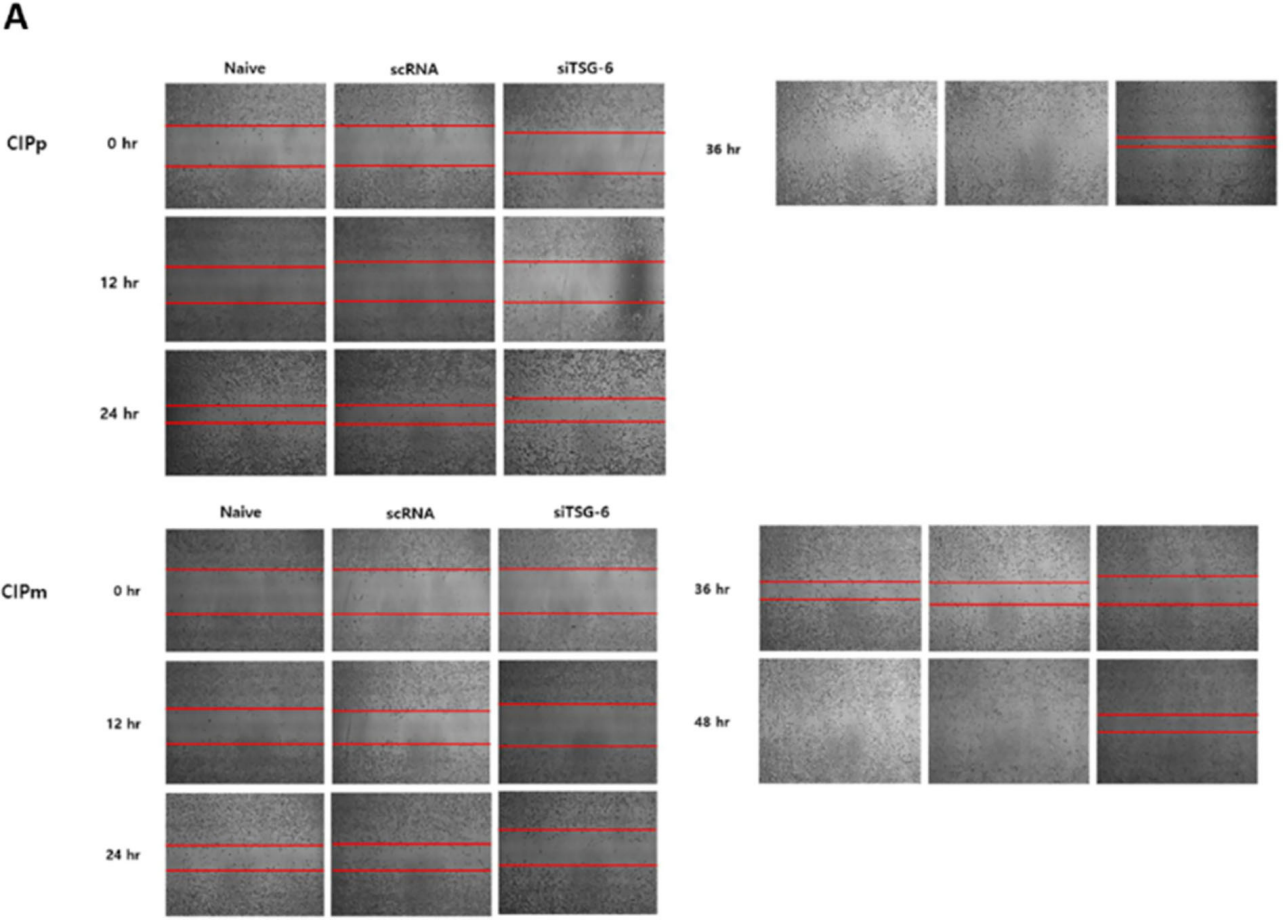

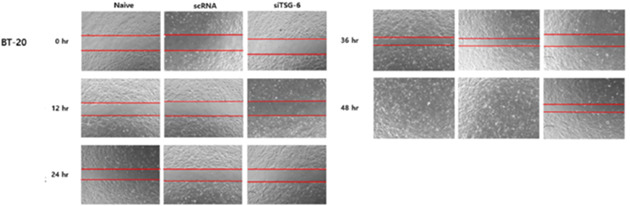

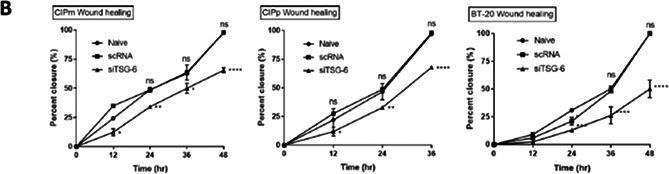

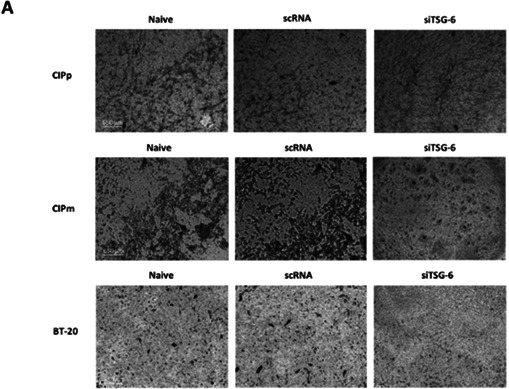

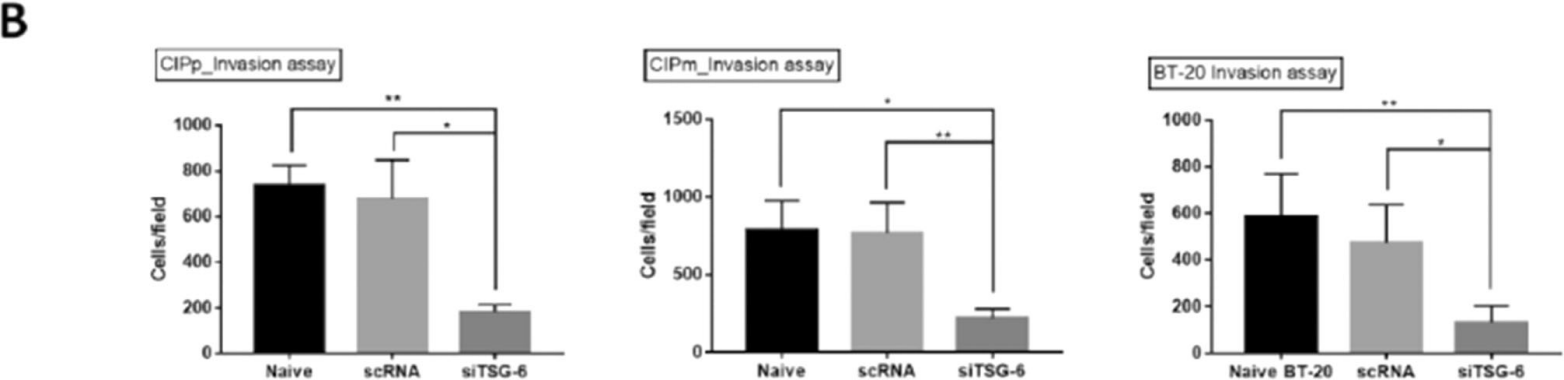

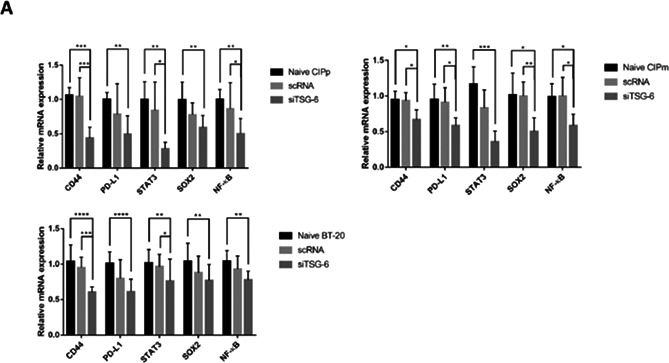

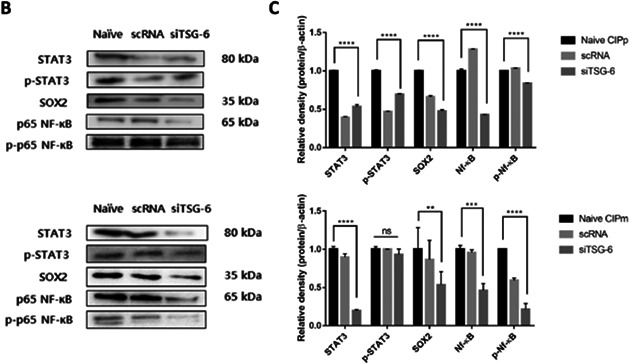

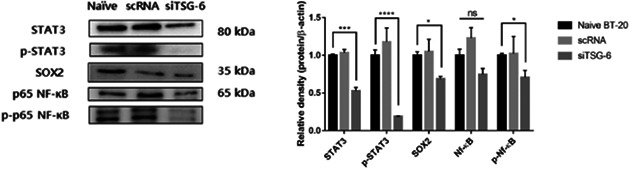

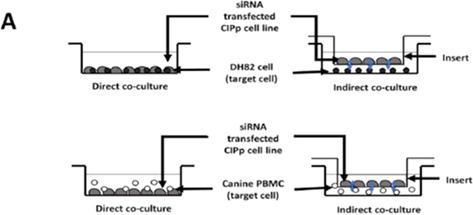

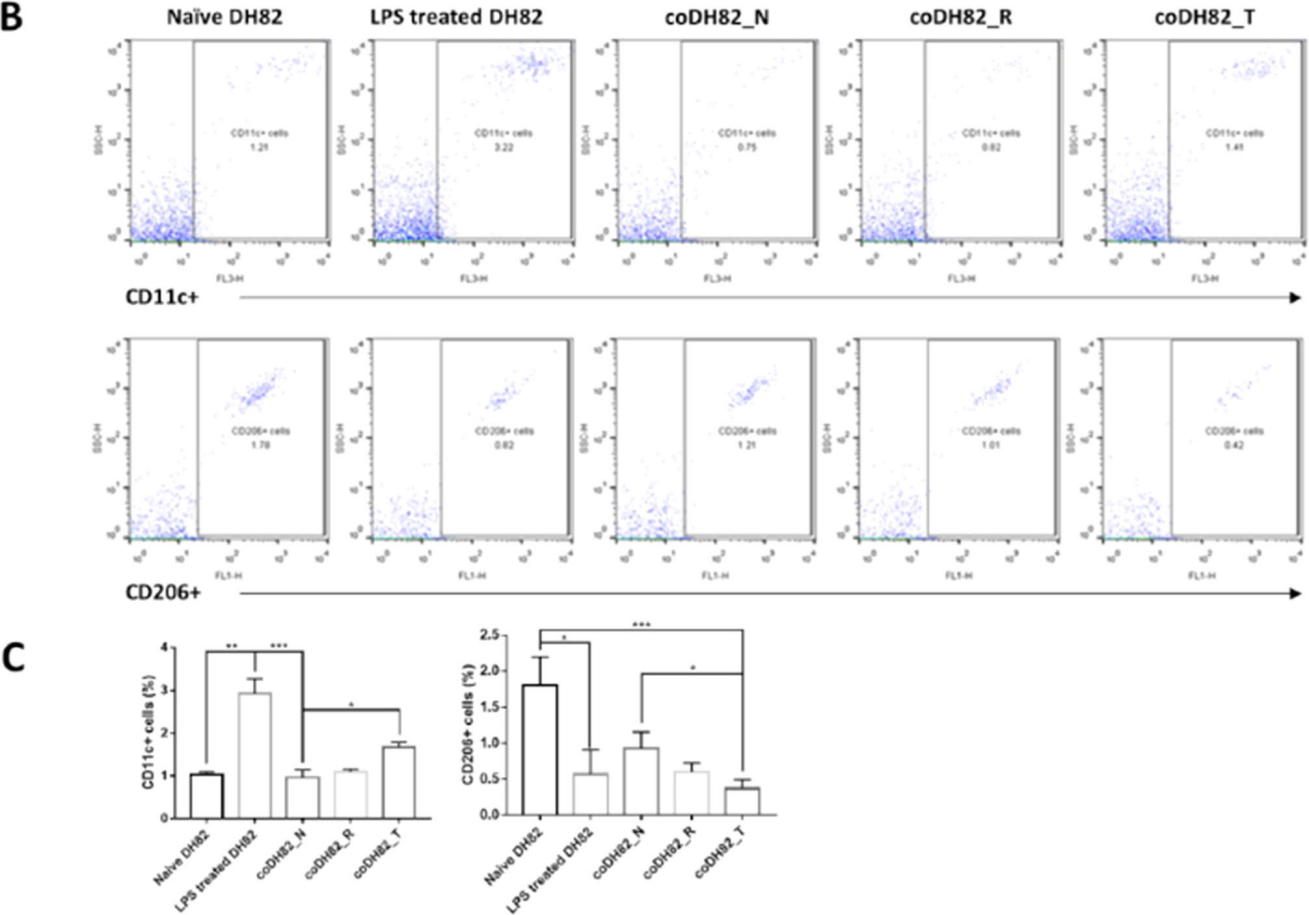

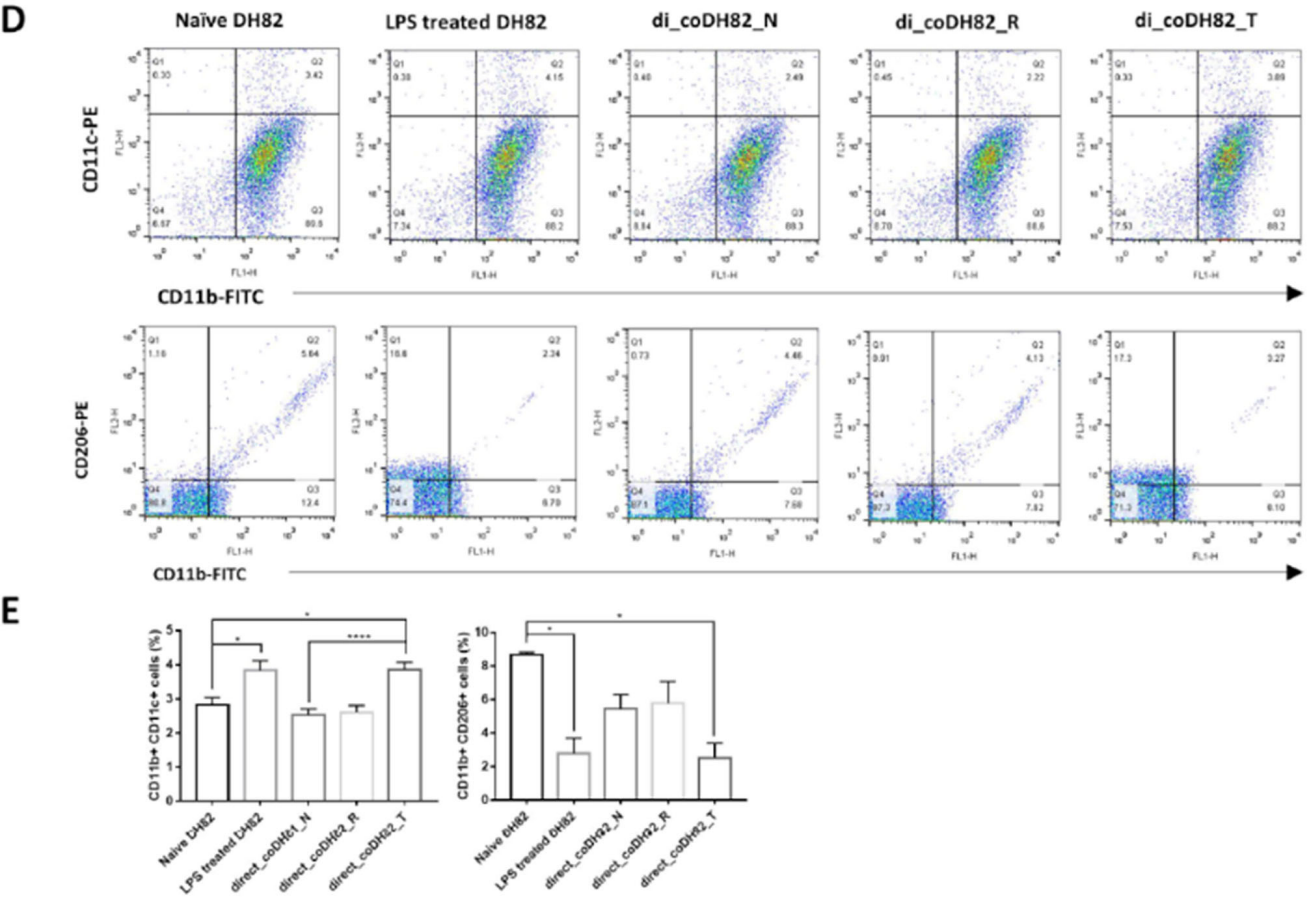

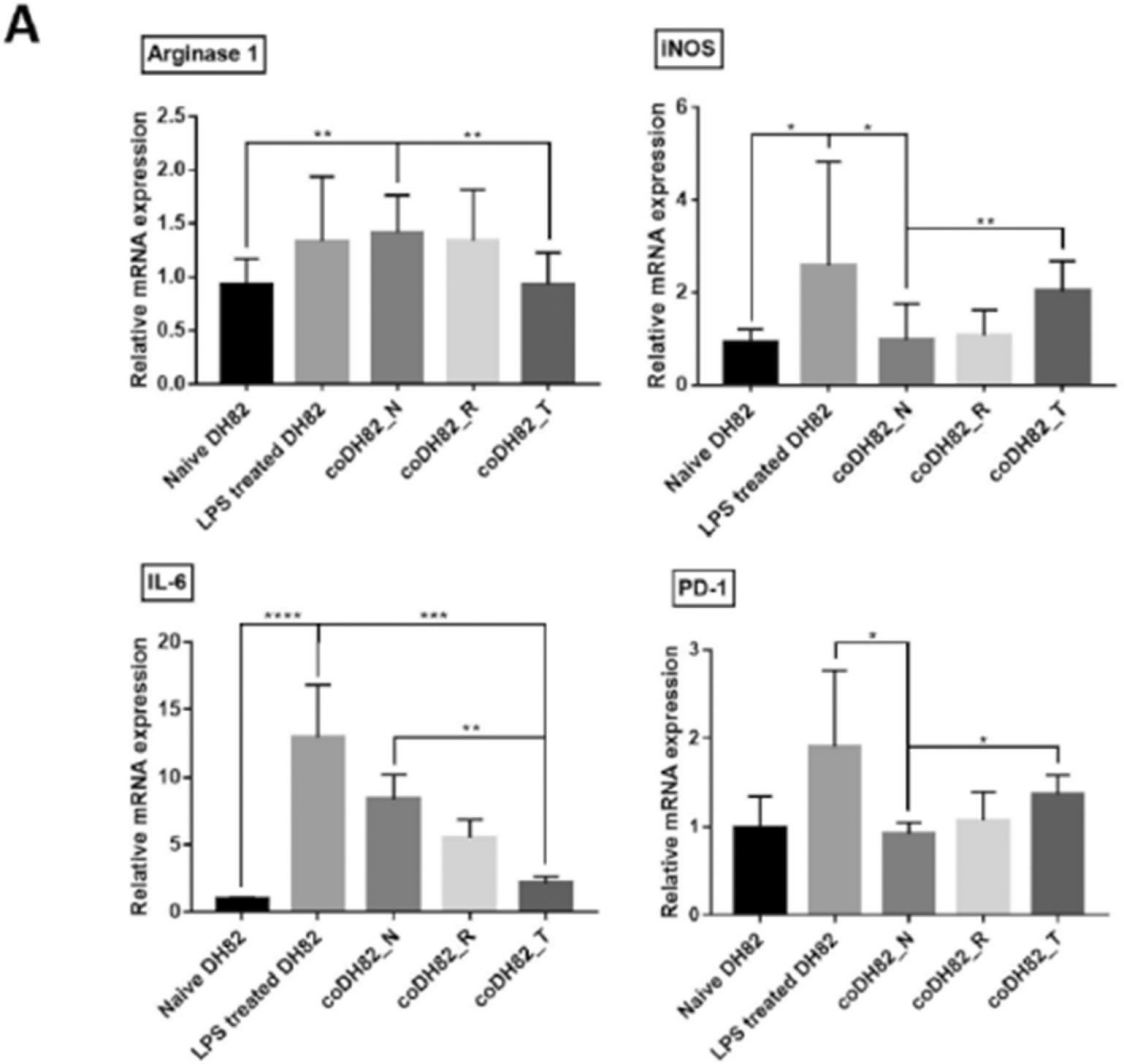

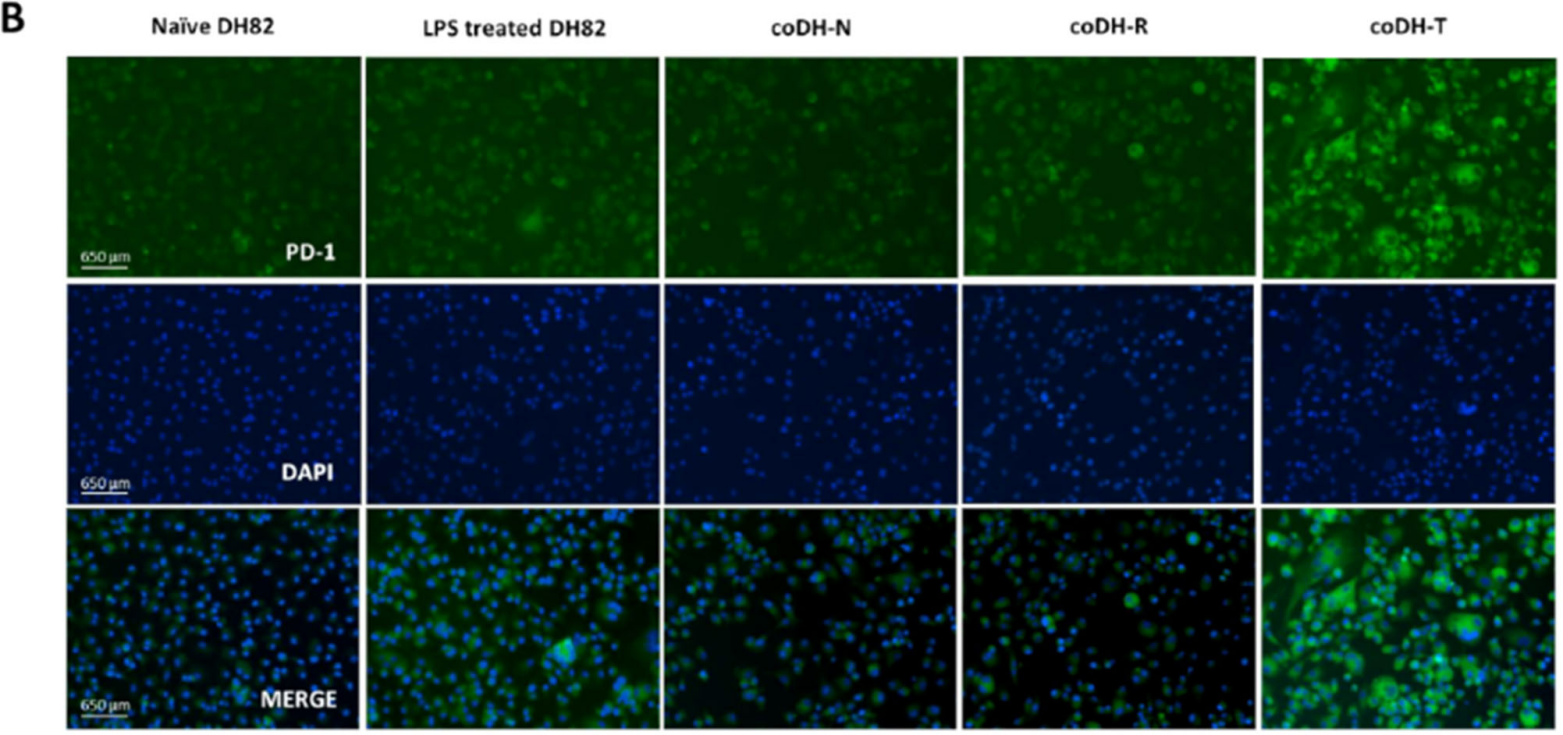

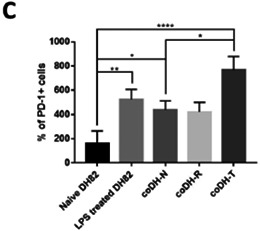

## Abstract O07: Kynurenine 3‐Monooxygenase and Ki67 Are Effective Biomarkers for Canine Cancer Diagnosis

### **Chen‐Si Lin**; Chiao‐hsu Ke

#### National Taiwan University, Taipei, Taipei, Taiwan (Republic of China)

**Background:** Early detection of tumors prolongs the lifespan of affected dogs. Though tumor biomarkers have been used, single detection provided limited information in clinical oncology. Kynurenine 3‐monooxygenase (KMO) is reported as a tumor‐related antigen, and Ki67 is strongly associated with tumor cell proliferation. However, few studies explore the combined evaluation of KMO and Ki67 in canine cancer diagnosis.

**Objective:** This study aimed to investigate the diagnostic values of KMO and Ki67 in canine plasma.

**Methods:** KMO and Ki67 concentrations in plasma were determined through a commercial enzyme‐linked immunosorbent assay (ELISA) from 180 cancer dogs and 56 tumor‐free individuals.

**Results:** KMO levels were significantly higher in cancer dogs (2.31 ± 0.88 μg/ml) than those in tumor‐free individuals (0.74 ± 0.70 μg/ml, *P* < 0.0001). The values of Ki67 increased in cancer dogs (4.65 ± 1.13 ng/ml) compared with those in controls (4.07 ± 0.78 ng/ml, <0.05). We further defined the tumor dogs into tumor‐positive or negative groups with the cut‐off values of 0.99 μg/ml (KMO) and 4.10 ng/ml (Ki67). Notably, 31.8% of tumor cases exhibited inconsistent KMO and Ki67 increase, which might imply that simultaneously determining KMO and Ki67 can potentiate the diagnostic power.

**Conclusions and Clinical Importance:** The results show that KMO and Ki67 were effective biomarkers in discriminating tumor‐bearing dogs from normal ones. Furthermore, we also established a dual‐screening platform with practical diagnostic ability in cancer dogs.

## Abstract O08: Genome‐wide CRISPR Knockout Screening Identifies Candidate Molecular Targets for Canine Oral Melanoma

### **Nick J. Parkinson**, MA, MS, VetMB, CertEM (IntMed), DACVIM, MRCVS; Keyi Tang, BSc MSc; Maciej Parys, DVM

#### University of Edinburgh, Roslin, Scotland, UK

**Background:** Canine oral melanoma carries a poor prognosis, and there is a need for additional therapeutic approaches. Genome‐wide knockout screening using the CRISPR‐Cas9 system is now a well‐established technique for identifying molecular vulnerabilities and genes underpinning treatment resistance *in vitro* in human molecular oncology, but this has not previously been applied to dogs.

**Hypothesis/Objectives:** Genome‐wide CRISPR knockout screening can identify potential novel molecular targets in cultured canine oral melanoma cells.

**Animals:** Commercially available oral melanoma cell lines derived from 3 dogs.

**Methods:** A first‐in‐species canine genome‐wide CRISPR knockout library was created allowing inactivation of 20,230 genes in parallel. This was introduced into canine melanoma cell lines CMGD2, CMGD5 and CML‐10, and control non‐neoplastic cells (fibroblasts), via a lentiviral vector. Integrated guide RNAs (sgRNAs) were sequenced as a proxy for gene knockout, and compared between baseline and surviving cells after 14–21 days of culture, to determine genes conditionally essential to melanoma cell survival, using statistical algorithm MAGeCK.

**Results:** Efficacy of the technique in canine cells was confirmed by consistent depletion of sgRNAs targeting known universally essential genes in human cells. Genes with statistically significant negative enrichment (FDR<0.05), without evidence of universal essentiality in normal cells, included potentially targetable genes implicated in other cancers, involved in biological pathways including Wnt signaling, growth hormone signaling and the hypusination pathway.

**Conclusions and Clinical Importance:** Genes identified as conditionally essential could be targets for novel therapeutics. Top candidates will be subject to *in vitro* validation using available inhibitors, to determine suitability for further translational investigation.

## Abstract O09: Patient Outcomes When Liquid Biopsy Was Used as an Aid in Diagnosis for Cancer

### **Allison L. O’Kell** ^1^, DVM, MS, DACVIM (SAIM); Katherine Lytle^1^, DVM, MPH, MS; Todd Cohen^1^, DVM, DACVIM (SAIM); Lilian Wong^1,2^, DVM, MS; Emily Sandford^1^, RVT; Jill Rafalko^1^, MS, CGC; Angela McCleary‐Wheeler^1^, DVM, PhD, DACVIM (Oncology); Patrick Fiaux^1^, PhD; Francesco Marass^1^, PhD; Maggie Marshall^1^, MS; Carlos Ruiz‐Perez^1^, PhD; Kristina Kruglyak^1^, PhD; John Tynan^1^, PhD; Susan Hicks^1^, MAS; Daniel Grosu^1^, MD, MBA; Jason Chibuk^1^, MS, CGC; Ilya Chorny^1^, PhD; Dana WY Tsui^1^, PhD; Andi Flory^1^, DVM, DACVIM (Oncology)

#### ^1^PetDx, La Jolla, CA USA; ^2^Carlson College of Veterinary Medicine, Oregon State University, Corvallis, OR, USA

**Background:** When a dog is suspected of having cancer, the workup can take many forms and a variety of testing modalities may be needed to arrive at a definitive or presumptive diagnosis.

**Hypothesis/Objectives:** To assess the performance of liquid biopsy testing and examine patient outcomes in a cohort of dogs that had samples submitted for liquid biopsy as an aid in diagnosis (AiD) for various reasons.

**Animals:** Client‐owned dogs that had blood samples submitted for liquid biopsy testing at one clinical laboratory.

**Methods:** Blood samples were subjected to DNA extraction, library preparation, and next‐generation sequencing (NGS). Sequencing data were analyzed for genomic alterations associated with the presence of cancer. Patient outcome data were obtained from veterinary clinics via phone and/or email.

**Results:** In a cohort of 1,500 consecutive clinical samples submitted for testing, 366 reportable samples were submitted as an AiD; 35% (*n =* 127) of these patients had information available regarding the reason for AiD testing and had sufficient outcome data to classify their results (Table 1). The positivity rate of liquid biopsy in the overall AiD population was 35% but highest for patients with inconclusive tissue testing. In this study population, 98% of dogs with a suspicion of cancer and positive result from liquid biopsy went on to receive a presumptive or definitive diagnosis of cancer.

**Conclusions and Clinical Importance:** Liquid biopsy offers a novel tool to aid in the diagnosis of cancer and has a high positive predictive value for patients that receive a Cancer Signal Detected result.

**Table:** Table 1. Outcome data for 127 dogs that had liquid biopsy testing submitted as an aid in diagnosis (AiD), and test performance by reason for AiD testing

Reason for AiD testing	Percentage of cases with cancer diagnoses following confirmatory workup	Positivity rate of liquid biopsy	True positive	False positive	True negative	False negative	Sensitivity	Specificity	PPV	NPV
Clinical suspicion* (*n =* 73)	45.2% (33/73)	27.4% (20/73)	19	1	39	14	57.6% (95% CI: 39.4–74.0)	97.5% (95% CI: 85.3–99.9)	95.0% (95% CI: 73.1–99.7)	73.6% (95% CI: 59.4–84.3)
Imaging suspicion (*n =* 34)	55.9% (19/34)	35.3% (12/34)	12	0	15	7	63.2% (95% CI: 38.6–82.8)	>99.9% (95% CI: 74.7–>99.9)	>99.9% (95% CI: 69.9–>99.9)	68.2% (95% CI: 45.1–85.3)
Inconclusive tissue testing (*n =* 14)	100% (14/14)	64.3% (9/14)	9	0	0	5	64.3% (95% CI: 35.6–86.0)	N/A	>99.9% (95% CI: 62.9–>99.9)	N/A
Other** (*n =* 6)	80% (4/5)	60% (3/5)	3	0	2	1	–	–	–	–
Overall (*n =* 127)	55.2% (70/127)	34.6% (44/127)	43	1	56	27	61.4% (95% CI: 49.0–72.6)	98.2% (95% CI: 89.4–99.9)	97.7% (95% CI: 86.5–99.9)	67.5% (95% CI: 56.2–77.1)

* Clinical suspicion: based on presenting history, results of physical exam, and/or lab results

** Other: Failure to achieve a diagnosis despite extensive workup (*n =* 2), to help convince the owner of the cancer diagnosis (*n =* 2), and owner suspicion of cancer (*n =* 1)

## Abstract O10: Evaluation of a Next‐Generation Sequencing‐Based Liquid Biopsy Test for Cancer Monitoring in Dogs

### **Andi Flory**, DVM, DACVIM (Oncology); Angela McCleary‐Wheeler, DVM, PhD, DACVIM (Oncology); Patrick Fiaux, PhD; Lisa McLennan, SCRA; Gilberto Hernandez, MPH, MBA; Carlos Ruiz‐Perez, PhD; Francesco Marass, PhD; Allison O’Kell, DVM, MS, DACVIM (SAIM); Todd Cohen, DVM, DACVIM (SAIM); Jill Rafalko, MS, CGC; Jason Chibuk, MS, CGC; Daniel Grosu, MD, MBA; Susan Hicks, MAS; John Tynan, PhD; Ilya Chorny, PhD; Kristina Kruglyak, PhD; Dana WY Tsui, PhD

#### PetDx, La Jolla, CA, USA

**Background:** After a dog is diagnosed with cancer and receives therapy, physical examination and imaging are typically used to assess disease status and monitor for recurrence.

**Hypothesis/Objectives:** To evaluate the performance of a next‐generation sequencing (NGS) based liquid biopsy test as an adjunct tool for cancer monitoring in dogs.

**Animals:** Client‐owned dogs enrolled in the CANcer Detection in Dogs study that had confirmed cancer diagnoses and Cancer Signal Detected results at the pre‐treatment visit.

**Methods:** Blood samples collected pre‐treatment and following therapeutic intervention (e.g., surgery, chemotherapy, radiation, etc.) were subjected to DNA extraction, library preparation, and NGS. Sequencing data were analyzed for genomic alterations associated with the presence of cancer.

**Results:** Following excisional surgery, in the absence of clinical residual disease at the first post‐operative visit, patients with Cancer Signal Detected (CSD) results at that visit were twice as likely to have clinical recurrence within 6 months, compared to patients with Cancer Signal Not Detected results. For patients that achieved complete response following therapy, a CSD result was issued prior to or concomitant with clinical recurrence in 70% of cases that experienced clinical recurrence during the study period. Overall, >95% of samples with CSD at a follow‐up visit had clinical disease at that visit or at a later visit.

**Conclusions and Clinical Importance:** NGS‐based liquid biopsy has the potential to improve monitoring of cancer in dogs. Patients with no clinical evidence of disease but with molecular evidence of disease may benefit from closer clinical evaluation and monitoring.

## Abstract O11: Neutropenia‐induced Adverse Events Following CCNU With or Without Prophylactic Antibiotics in Dogs

### **Emily J. Rawlings** ^1^; Jessica Lawrence^2^, DVM, DACVIM (Oncology), DACVR, DECVDI; Kyla Walter^3^, DVM, PhD; Amber Wolf‐Ringwall^4^, DVM, PhD, DACVIM (Oncology)

#### ^1^University of Minnesota, Minneapolis, MN, USA; ^2^Associate Professor of Radiation Oncology, Department of Veterinary Clinical Sciences, College of Veterinary Medicine, University of Minnesota, Minneapolis, MN, USA; ^3^Small Animal Surgery Resident, Surgical & Radiological Sciences, School of Veterinary Medicine; University of California–Davis, Davis, CA, USA; ^4^Assistant Professor of Oncology, Department of Veterinary Clinical Sciences, College of Veterinary Medicine, University of Minnesota, Minneapolis, MN, USA

**Background:** Lomustine (CCNU) can cause severe neutropenia in dogs, increasing the risk of life‐threatening secondary bacterial infections. Prophylactic antibiotics (pAB) may decrease the incidence of bacterial infection but contribute to growing antimicrobial resistance. To improve judicious use of pAB, it is important to define their role in improving patient care.

**Hypothesis/Objectives:** Similar rates of neutropenia‐related adverse events occur in dogs when pAB are given with CCNU at only the first treatment compared to pAB given with each treatment.

**Animals:** Pet dogs treated with CCNU by an oncology service.

**Methods:** Medical records were retrospectively queried between 2019–2023 for dogs with lymphoma or histiocytic sarcoma treated with CCNU. Use of pAB, CCNU dosage, grade 4 neutropenia, and febrile and hospitalization events were compared between dogs receiving pAB with the first cycle or with 2 cycles using Fisher's exact test.

**Results:** Seventy‐eight CCNU treatments in 39 dogs were evaluated. Twenty‐one dogs received pAB with the first CCNU treatment only (Group 1); 18 dogs received pAB with both treatments (Group 2). Five treatments in group 1 (12%) and 11 in group 2 (30%) resulted in grade 4 neutropenia (*P* = 0.052). Six treatments in group 1 (14%) and 15 in group 2 (42%) resulted in dose reduction (*P* = 0.010). Febrile events occurred in 19% of treatments without pAB compared to 3.5% with pAB (*P* = 0.042).

**Conclusions and Clinical Importance:** The timing and role of pAB in dogs undergoing CCNU treatment needs to be better defined. The advantages must be weighed against potential disadvantages of antibiotic resistance.

## Abstract O12: Single‐Cell Analysis of Natural Killer Cells in Response to Immunotherapy in First‐in‐Dog Clinical Trials

### **Aryana Razmara** ^1^; Rayna Harris^1^; Lauren Farley^1^; Sean Judge^1^; Marshall Lammers^1^; Cordelia Dunai^1^; William Murphy^1^; Robert Rebhun^1^; Zachary Morris^2^; David Vail^2^; Titus Brown^1^; Michael Kent^1^; Robert Canter^1^

#### ^1^University of California–Davis, Davis, CA, USA; ^2^University of Wisconsin, Madison, WI, USA

**Background:** We have completed canine immunotherapy trials using overlapping but distinct methods to target natural killer (NK) cells in dogs with naturally occurring cancer. High‐throughput single‐cell (sc)RNASeq allows the profiling of individual cell subtypes to thoroughly examine transcriptomic changes in clinical samples.

**Hypothesis/Objectives:** Our objective was to investigate changes in NK cell populations in response to immunotherapy treatment and compare gene expression profiles between dogs receiving two separate NK‐targeting treatments.

**Animals:** Samples were available for five dogs treated for cancer at UC Davis (UCD, *n =* 1) and University of Wisconsin (UW, *n =* 4) in IACUC‐approved pilot trials.

**Methods:** The UCD cohort underwent palliative radiotherapy (RT) in addition to allogeneic NK cell transfer. Dogs in the UW cohort received low‐dose molecular targeted radionuclide therapy (MTRT) with intratumoral injection of IL‐2 fusion cytokine. Single‐cell (sc)RNASeq was performed on isolated PBMCs before, during, and following treatment.

**Results:** During and following treatment with RT and allogeneic transfer, NK cells retained robust expression of NK activating and functional genes, including KLRD1, GZMB, KLRA1, and NCR3. Dogs receiving MTRT therapy had increased expression of activation markers, such as CD69 and KLRK1, in post‐treatment NK cells compared to pretreatment. Notably, the MTRT therapy‐specific NK gene activation signature was similar across patients receiving the same treatment.

**Conclusions and Clinical Importance:** This scRNASeq analysis provides insight into the diverse mechanisms of NK activation and serves as a basis for investigating innovative therapeutic combinations to improve cancer treatment and survival in canine patients.

## Abstract O13: Clinico‐Genomics Analysis of Canine Anal Sac Adenocarcinoma: Genomic Profile and Treatment Opportunity

### **Lucas Rodrigues**; Aubrey Miller; Abigail Hull; Lindsay Lambert; Dorothy Girimonte; Garrett Harvey; Gerald Post; Christina Lopes; Michelle White

#### FidoCure, Madison, WI, USA

Anal sac adenocarcinoma (ASA) is an aggressive tumor from the epithelium of anal sac gland. Clinico‐genomics data of 121 dogs with ASA from FidoCure Precision medicine platform were evaluated. Eighty‐six dogs had genetic alterations such as somatic mutations, BRCA1/2 somatic/germline deleterious mutations or CNVs. KMT2D (16/121), BRCA2 (13/121), PARP1 (11/121) and BRCA1 (10/121) were the most common alterations identified. Six dogs had an ERBB2 mutation: 5 located at position K676E and one at position V659E. The ERBB2 K676E mutation has been identified solely in ASA across 2811 canine tumors. Targeted therapies were recommended based on detected mutations. Overall survival of dogs with local lymph node involvement receiving targeted therapy (TT) and chemotherapy (CHEMO) was 794 days (n: 24), for dogs treated with TT only 462 days (n: 11), and 934 days (n: 6) for dogs receiving TT+CHEMO and radiation therapy (*P* = 0.0124). Survival of dogs with no lymph node involvement treated with TT only were 1211 days (n: 11) and 1186 days (n: 5) for TT+CHEMO (*P* = 0.8399). Survival of dogs with lymph node involvement receiving TT only recommended based on genomic targets was 721 days (n: 5) compared to 462 days (n: 6) when TT was used alone regardless of the genomic profile (*P* = 0.9927). This is the first time ERBB2 K676E was exclusively identified in dogs with ASA. More studies are needed to identify opportunities to use TT guided by genomics to improve survival of dogs with ASA.

## Abstract O14: Mutation Landscape and Clinical Actionability in Canine Osteosarcoma

### **William Hendricks**; Esther Chon Jonathan Adkins; Natalie Duran; Sara Aman; Darwin Tsinajinnie; Manisha Warrier; Derick Whitley; David Haworth; Sharadha Sakthikumar; Guannan Wang

#### Vidium Animal Health, Scottsdale, AZ, USA

**Background:** Osteosarcomas account for most primary bone malignancies in dogs. Despite aggressive conventional therapies, most dogs eventually succumb to this disease. A rapidly growing genomic understanding of canine osteosarcoma alongside an increasing arsenal of genomically guided therapeutic options for human cancers is now pointing to new potential therapeutic targets for canine osteosarcoma.

**Hypothesis/Objectives:** To uncover actionability horizons, our objective was to evaluate the genomic landscape of canine osteosarcoma and identify biomarkers with diagnostic, prognostic, and therapeutic associations.

**Animals:** Sixty‐five canine osteosarcoma cases submitted for tumor genomic analysis with SearchLight DNA were selected for evaluation.

**Methods:** Osteosarcoma samples profiled with SearchLight DNA, a canine cancer gene sequencing panel, were included in this case series. Mutations with biomarker associations, including those associated with responses to targeted therapies based on canine or human cancer evidence, were identified and quantified.

**Results:** In 65 dogs with osteosarcoma, 754 (167 unique) mutations were identified in 79 genes, with TP53 being the most frequently mutated gene. Ten genes bore mutations with evidence supporting sensitivity to 4 FDA‐approved targeted therapies, accounting for 46 patients (71%) for whom therapeutic biomarkers were associated with at least 1 FDA‐approved targeted therapy. Three of these drugs are readily available to veterinarians from at least 1 major compounding pharmacy.

**Conclusions and Clinical Importance:** Understanding the genomic landscape of canine osteosarcoma in the context of mutation‐based biomarker evidence supports the use of targeted therapies already available to veterinarians. These data also present opportunities to leverage human FDA‐approved therapies in veterinary patients.

## Abstract O15: Osteopontin Overexpression in Localized Canine Histiocytic Sarcoma

### **Jennifer A. Lenz** ^1^; Charles‐Antoine Assenmacher^2^; Enrico Radaelli^2^; Matthew Atherton^2^

#### ^1^School of Veterinary Medicine, University of Pennsylvania, Philadelphia, PA, USA; ^2^University of Pennsylvania, Philadelphia, PA, USA

**Background:** Histiocytic sarcoma (HS) is a highly lethal malignancy. Increased tumor‐infiltrating lymphocyte density has been positively associated with survival in canine HS patients, however, even patients with high numbers of intratumoral T cells ultimately succumb to their disease, suggesting the eventual failure of anti‐tumor immunity.

**Hypothesis/Objectives:** The objective of this study was to identify potential drivers of tumor progression and immunoevasion in canine HS.

**Animals:** Eighteen client‐owned dogs diagnosed with localized HS of variable anatomic locations.

**Methods:** Archived HS tumors were retrospectively identified. RNA extraction was performed on a subset of tumors from patients with short and long survival times and isolated RNA was subjected to NanoString immune transcriptional profiling and compared to RNA isolated from healthy control tissues. All 18 samples were subsequently stained for osteopontin using immunohistochemistry to assess expression at a protein level and digital images were captured.

**Results:** Immune transcriptional profiling of HS tumors revealed SPP1 (osteopontin) to be the most upregulated gene expressed in canine HS samples. Osteopontin protein was expressed variably in all canine HS tumors with the strongest expression noted in peri‐articular tumors.

**Conclusions and Clinical Importance:** We demonstrated overexpression of osteopontin at a transcriptional and protein level implicating a potential pro‐tumorigenic role for this protein in canine HS. Previous literature reports a T cell suppressive role of osteopontin, and future studies are planned to determine the functional effect of osteopontin exposure on canine T cells.

## Abstract O16: Alternating Rabacfosadine and Doxorubicin for Treatment of Naive Canine Lymphoma

### **Corey F. Saba** ^1^; Katherine Robinson^2^; Timothy Fan^3^; Brenda Phillips^4^; Douglas Thamm^5^; Zachary Wright^6^

#### ^1^University of Georgia, Athens, GA, USA; ^2^BluePearl Seattle, Seattle, WA, USA; ^3^University of Illinois, IL, USA; ^4^Veterinary Specialty Hospital by Ethos Veterinary Health; ^5^Colorado State University, Fort Collins, CO, USA; ^6^VCA Animal Diagnostic Clinic

**Background:** The current standard of care treatment for canine lymphoma is a multi‐agent, CHOP‐based chemotherapy protocol. Single agent doxorubicin (DOX) is a less time‐consuming option; however, multi‐agent chemotherapy protocols are often superior. The recently approved drug rabacfosadine (RAB, Tanovea) provides an attractive option for combination therapy with DOX, as both drugs demonstrate efficacy against lymphoma and possess different mechanisms of action. A previous study evaluating alternating RAB/DOX reported an overall response rate (ORR) of 84%, with a median progression‐free interval (PFI) of 194 days.

**Hypothesis/Objectives:** The aim of this prospective trial was to evaluate the same protocol in an additional population of dogs.

**Animals:** Fifty‐nine dogs with treatment‐naïve lymphoma.

**Methods:** RAB (1.0 mg/kg IV) was alternated with DOX (30 mg/m^2^ IV) every 21 days for up to six total treatments (3 cycles). Response assessment and adverse event (AE) evaluation were performed every 21 days using VCOG criteria.

**Results:** The ORR was 93% (79% CR, 14% PR). The median time to maximal response was 21.5 days; median PFI was 199 days. T‐cell immunophenotype and lack of treatment response were predictive of inferior outcomes. AEs were mostly gastrointestinal. Six dogs developed confirmed or presumed pulmonary fibrosis; four were grade 5. One dog experienced grade 3 extravasation injury with RAB that resolved with supportive treatment.

**Conclusions and Clinical Significance:** These data mirror those of the previously reported RAB/DOX study and support the finding that alternating RAB/DOX is a reasonable treatment option for canine lymphoma.

## Abstract O17: Gene Expression Profiles Associated With Sensitivity to Molecular‐Targeted Drugs in Canine Histiocytic Sarcoma Cells

### **Hiroki Sakuma** ^1^; Yuko Goto‐Koshino^1^; Akiyoshi Tani^1^; Keita Ito^2^; Makoto Bonkobara^2^; Hirotaka Tomiyasu^1^

#### ^1^The University of Tokyo, Tokyo, Japan; ^2^Nippon Veterinary and Life Science University, Tokyo, Japan

**Background:** The effective treatments for canine histiocytic sarcoma (CHS) remain to be established. We previously revealed that the sensitivities to three molecular‐targeted drugs, dasatinib, trametinib, and ponatinib, were significantly different among CHS cell lines.

**Hypothesis/Objectives:** To investigate the gene expression profiles associated with the sensitivity to the three molecular‐targeted drugs in CHS cell lines.

**Methods:** RNA was extracted from twelve CHS cell lines, and the gene expression profiles were analyzed by RNA sequencing. Differentially expressed genes (DEGs) were extracted by the comparisons between the drug‐sensitive and ‐resistant cell lines, and they were subjected to KEGG pathway analysis. The differences in the expression levels of these DEGs were validated by RT‐qPCR.

**Results:** The clustering analysis showed that CHS cell lines were divided into two distinct clusters, and one cluster consisted of the three most drug‐resistant cell lines (Figure 1). The comparisons of these three cell lines and the three most drug‐sensitive cell lines extracted 624 DEGs, and KEGG pathway analysis indicated these DEGs were enriched with those related to the regulation of PI3K‐Akt pathway. RT‐qPCR analysis confirmed the significant upregulations of TLR4, IL6R, PKN1, PPP2R3C, and SPP1 genes and the significant downregulations of F2R, GNG12, COL4A2, PDGFA, ITGA3, and LAMA3 genes in the three drug‐resistant cell lines compared to other nine cell lines.

**Conclusions and Clinical Importance:** The results of this study suggested that the differences in the activations status of intracellular signaling pathways including PI3K‐Akt pathways were associated with the differences in the sensitivities to the three molecular‐targeted drugs.

**Figure:**
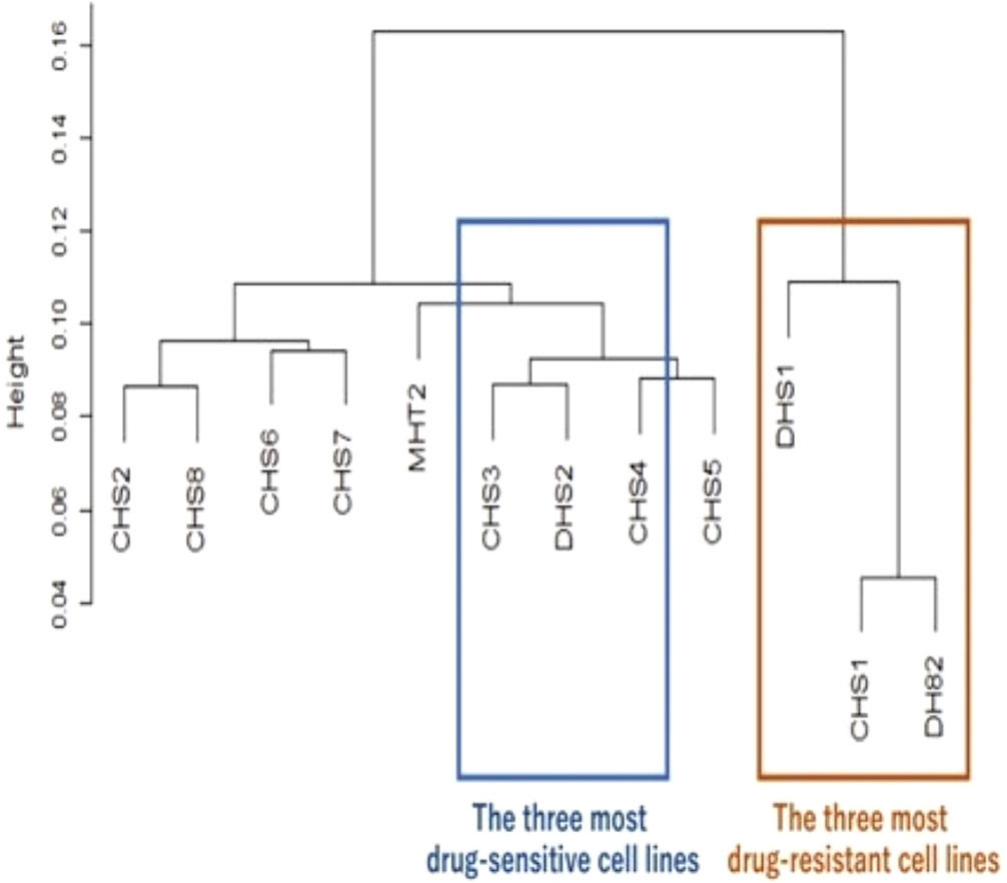
Figure 1. The results of the clustering analysis based on the gene expression profiles of twelve canine histiocytic sarcoma cell lines

## Abstract O19: Genome‐wide DNA Methylation Analysis Identified Prognostic CpG Site Markers in Canine Multicentric High‐grade Lymphoma

### **Yong Bin Teoh** ^1^; Teita Ishizaki^2^, DVM, PhD, DACVP; Yumiko Kagawa^3^, DVM, PhD, DACVP; Yuki Matsumoto^4^, PhD; Hirotaka Tomiyasu^5^, OhD; Mitsuyoshi Takiguchi^6^, DVM, PhD, DAiCVIM; Hajime Tsujimoto^7^, DVM, PhD, AiCVIM; Jumpei Yamazaki^8^, DVM, PhD

#### ^1^Veterinary Teaching Hospital, Hokkaido University, Hokkaido, Japan; ^2^Clinical Pathologist, North Lab; ^3^Anatomic Pathologist, North Lab; ^4^Chief Researcher, Department of Research Development, Anicom Specialty Medical Institute, Inc; ^5^Associate Professor, Department of Veterinary Medical Sciences, The University of Tokyo, Tokyo, Japan; ^6^Professor, Clinical Sciences, Hokkaido University, Hokkaido, Japan; ^7^Tokyo Branch Hospital Director, Japan Animal Referral Medical Center, Tokyo, Japan; ^8^Program Associated Professor, Hokkaido University, Hokkaido, Japan

**Background:** Canine multicentric high‐grade lymphoma (MHGL) patients usually benefit from chemotherapy, but some have poorer prognosis. DNA methylation analysis may identify prognostic CpG sites for canine MHGL.

**Objective:** Identify prognostic CpG sites of MHGL through genome‐wide DNA methylation analysis with pyrosequencing validation.

**Animals:** Test group: 24 dogs. Validation group: 86 dogs. All client‐owned dogs were diagnosed with MHGL and treated with CHOP chemotherapy.

**Methods:** Genome‐wide DNA methylation analysis using Digital Restriction Enzyme Analysis of Methylation (DREAM) was performed on the test group to identify differentially methylated CpG sites (DMCs). Bisulfite pyrosequencing was used to measure the methylation status of candidate CpG sites in the validation group. Survival curves were generated and analyzed using the Kaplan‐Meier (log‐rank) product limit method.

**Results:** DNA methylome analysis of 101,576 CpG sites hierarchically clustered the test group into 4 groups with different mean survival times (MST). The longest MST group (463 days, *P* = 0.0015) had 278 hyper‐ and 441 hypo‐DMCs. Among significant DMCs, DMCs near FAM213 and PHLPP1 genes were identified as candidates. Pyrosequencing results of the validation group showed that the group with DMC methylation near the FAM213 gene at <40% had a longer MST (499 days, *P* = 0.0102). Dogs grouped with combined DMC methylation near genes of FAM213 at <40% and PHLPP1 at <10% had an even longer MST (694 days, *P* = 0.00024).

**Conclusion and Clinical Importance:** Methylation status of prognostic CpG sites can delineate canine MGHL cases with longer MST, warranting active pursuit of CHOP chemotherapy.

## Abstract O20: Gene Expression Profiles Associated With Early Relapse in Canine Multicentric High‐grade B‐Cell Lymphoma

### **Hiroto Toyoda** ^1^; Akiyoshi Tani^1^; Yuko Goto‐Koshino^2^; Tomoki Motegi^3^; Mika Sakamoto^4^; Takako Mochizuki^4^; Kei Harada^5^; Tetsuya Kobayashi^5^; Asuka Setoguchi^6^; Yohei Shizuta^7^; Takuya Mizuno^8^; Mitsuhiro Irie^9^; Jun Nakamichi^10^; Hajime Tsujimoto^10^; Aki Ohmi^11^; Ray Fukuoka^2^; Yasukazu Nakamura^12^; Hirotaka Tomiyasu^2^

#### ^1^Department of Veterinary Internal Medicine, Graduate School of Agricultural and Life Sciences, The University of Tokyo, Tokyo, Japan; ^2^The University of Tokyo, Tokyo, Japan; ^3^ School of Medicine, Boston University, Boston, MA, USA; ^4^National Institute of Genetics; ^5^Japan Small Animal Cancer Center; ^6^JASMINE Animal Referral Hospital, Japan; ^7^Bayside Animal Clinic; ^8^Yamaguchi University, Yamaguchi, Japan; ^9^Shikoku Veterinary Medical Center, Kagawa, Japan; ^10^Japan Animal Referral Medical Center, Japan; ^11^Veterinary Medical Center, The University of Tokyo, Tokyo, Japan; ^12^Genome Informatics Laboratory, National Institute of Genetics, Japan

**Background:** Multicentric high‐grade B‐cell lymphoma (mhBCL) is the most common subtype of canine lymphoma. Although chemotherapy by CHOP protocol induces remission in most cases, part of the cases develops early relapse during the first remission induction chemotherapy.

**Objective:** To investigate the gene expression profiles associated with early relapse during the first remission induction chemotherapy in canine mhBCL.

**Animals:** Twenty‐five mhBCL cases diagnosed with centroblastic type and treated by UW‐25 protocol as first remission induction chemotherapy were included in this study. Sixteen cases completed UW‐25 protocol without relapse (S‐group), and nine relapsed during the protocol (R‐group) (Figure 1).

**Methods:** Tumor cell samples were collected by fine‐needle aspirations at diagnosis, and RNA was extracted from the samples. RNA‐seq was performed using the samples of eighteen dogs and the gene expression profiles were compared between the S‐ and R‐groups. The mRNA expression levels of the extracted differentially expressed genes (DEGs) were compared between the S‐ and R‐groups using the samples of twenty‐three dogs by RT‐qPCR (Figure 1).

**Results:** Extracted 179 DEGs included the genes related to T‐cell receptor signaling pathway and PD‐L1 expression and PD‐1 checkpoint pathway. CD3E, ITK, and LAT genes were confirmed to be significantly upregulated in the R‐group by RT‐qPCR (Figure 2).

**Conclusions and Clinical Importance:** The results of this study suggested that the tumor microenvironment including T cells in mhBCL tissues were associated with the early relapse during first UW‐25 protocol.

**Figure:**
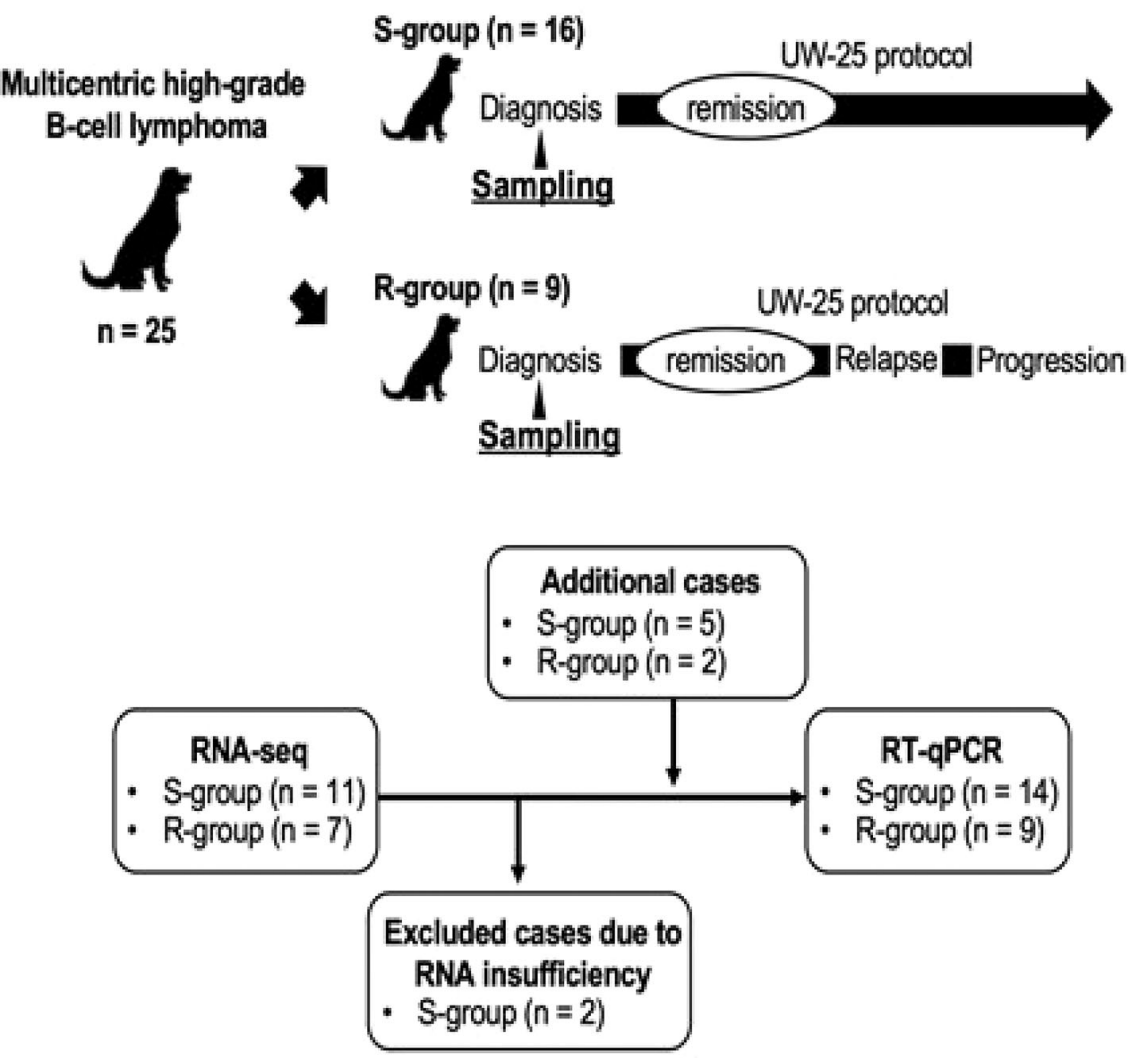
Figure 1. Dogs included in this study

**Figure:**
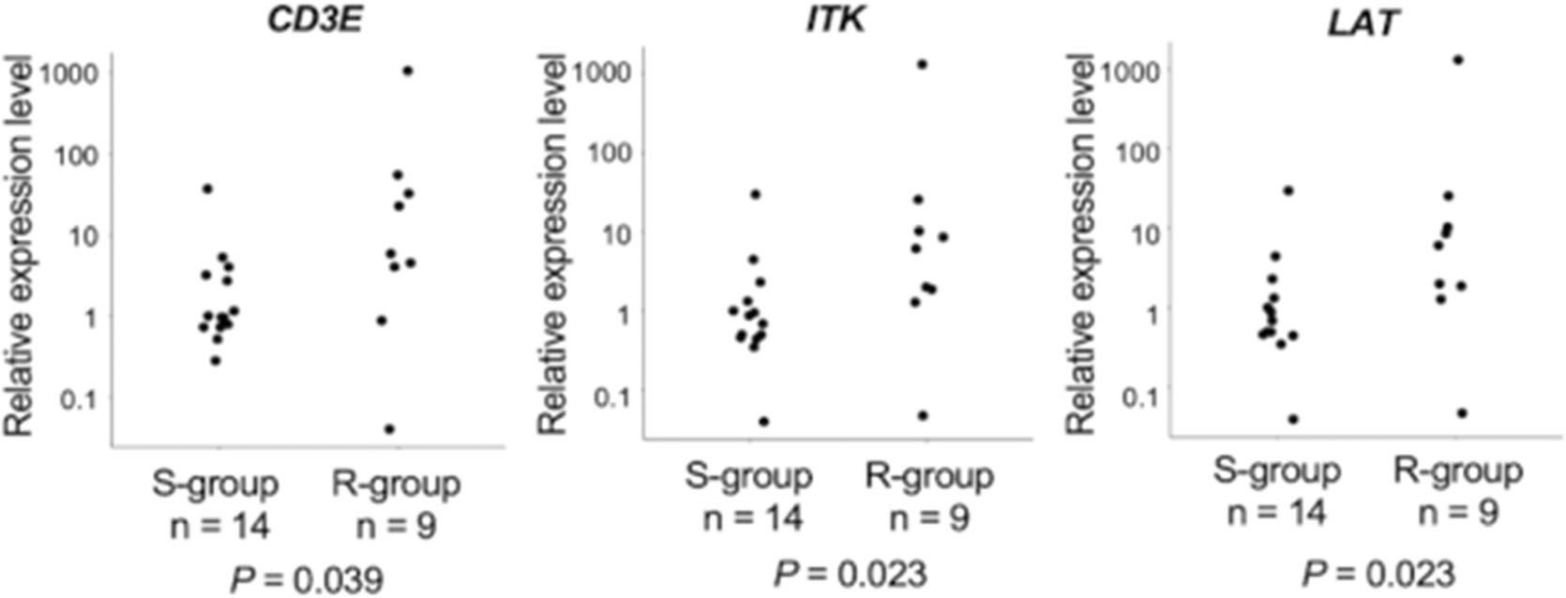
Figure 2. Comparisons of the expression levels of the extracted differentially expressed genes between S‐ and R‐groups

## Abstract O21: Genomic Analysis Across 50 Canine Cancer Types Reveals Novel Mutations and High Clinical Actionability

### **Guannan Wang** ^1^, PhD; Jonathan Adkins^1^; Zeeshan Ahmed^1^; Sara Aman^1^; Esther Chon^1^; Natalie Duran^1^; Salvatore Facista^1^; David Haworth^2^, DVM, PhD; William Hendricks^1^, PhD; Sharda Sakthikumar^3^; Darwin Tsinajinnie^1^; Derick Whitley^1^, DVM; Manisha Warrier^4^

#### ^1^Vidium Animal Health; ^2^President, Vidium Animal Health ^3^Cancer Genomic Scientist, Vidium Animal Health; ^4^Bioinformatician II, Vidium Animal Health

Genomics‐guided precision medicine has revolutionized human oncology. Genomic knowledge, diagnostics, and novel therapeutics are now also increasingly available for canine cancers. A need still exists for the continued expansion of this genomic understanding.

To define the clinical utility of genomic diagnostics for guiding cancer management in canine patients, we report on mutations and biomarkers identified by SearchLight DNA, a canine cancer gene sequencing panel, in 693 canine cancer cases.

Cancer tissue from 693 dogs was collected via retrospective study of biobanked material as well as through clinical cases submitted for SearchLight DNA analysis.

For genomic analysis, SearchLight DNA is an analytically validated next‐generation sequencing‐based, 120‐gene panel, designed to detect most cancer‐driving mutation types. For biomarker annotation, Vidium Insight is a canine precision oncology biomarker knowledgebase containing over 4000 mutational biomarkers with diagnostic, prognostic, and therapeutic associations, used to annotate candidate pathogenic and clinically relevant mutations from sequenced cases.

We identified 6582 (927 unique) mutations in 103 genes for 693 cases, of which, 644 harbored mutations previously detected in canine or human cancer, and associated with diagnostic, prognostic, and/or therapeutic indications (actionable yield: 92.9%). Over 50 well‐defined cancer histologies were analyzed, 19 with at least 5 cases (actionable yield: 72.2–100%; Median: 95.8%). Notably, this study comprises the first in‐depth genomic analysis of 11 of these 19 types.

We present mutation data with structured biomarker annotation across 693 samples and 50 cancer types, providing unprecedented insight into the genomic underpinnings and clinical actionability of canine cancers.

## Abstract O23: The Diagnostic Value of Thymidine Kinase 1 Auto‐Antibody in Canine Cancer Patients

### **Hwayoung Youn** ^1^; Dong‐Ha Bang^2^; Ul‐Soo Choi^3^; Yoon‐Hee Kim^3^; Jeong‐Joo Park^2^; Kyung‐Rok Yu^4^

#### ^1^College of Veterinary Medicine, Seoul National University, Seoul, Republic of Korea; ^2^Biattic Inc, Anyang‐si, Gyeonggi‐do, Republic of Korea; ^3^Department of Veterinary Clinical Pathology, College of Veterinary Medicine, Jeonbuk National University, Jeonju, Republic of Korea; ^4^Department of Agricultural Biotechnology and Research Institute of Agriculture and Life Sciences, Seoul National University, Seoul, Republic of Korea

**Background:** Recognizing the need for a more effective malignant tumor screening method in dogs, we tried to devise an algorithm by comparing a combination of several biomarkers.

**Objectives:** We investigated the possibility of TK1 autoantibody as a tumor biomarker and devised a reliable algorithm after comparative analysis of several biomarker candidates.

**Animals:** Random serum samples of 1,702 dogs, healthy or diagnosed with certain diseases or tumors were collected from local and referral animal medical centers in Korea.

**Methods:** Using LFIA methods, the concentrations of thymidine kinase 1 (TK1) autoantibody were measured in serum samples of dogs classified into healthy control, nontumor disease group, and patients with benign and malignant tumors. A multiple logistic regression model was designed combining all biomarker candidates.

**Results:** TK1 autoantibody was statistically higher in malignant tumor patients than in the control group (Welch t test, *P* < 0.0001), and also showed significant differences in carcinoma, hematopoietic tumor, and sarcoma (*post hoc* Games‐Howell test, *P* < 0.0001). As a result of the combined analysis of TK1 autoantibody, TK1 protein, CRP, age and breed, AUC was 0.966, which was similar to the result of replacing TK1 autoantibody with ECPKA autoantibody (AUC, 0.974).

**Conclusions and Clinical Importance:** TK1 autoantibody proved useful as one of the biomarkers for malignant tumor screening in dogs, and it was confirmed that an algorithm combining several biomarker candidates had high sensitivity and specificity.

## Abstract O24: Novel Genomic Prognostic Biomarkers for Canine Cancer Patients

### **William Hendricks** ^1^; Esther Chon^1^, DVM, DACVIM (Oncology); Jonathan Adkins^1^; Natalie Duran^1^; Sara Aman^1^; Darwin Tsinajinnie^1^; Manisha Warrier^1^; Sharadha Sakthikumar^1^; Salvatore Facista^1^; Derick Whitley^1^; David Haworth^1^; Min Tang^2^; Guannan Wang^1^

#### ^1^Vidium Animal Health, AZ, USA; ^2^Statbeyond Consulting LLC

**Background:** Growing evidence from dogs and humans supports the existence of abundant mutation‐based biomarkers in canine cancers. Increasing use of clinical genomic diagnostics now provides another powerful data source for biomarker discovery.

**Hypothesis/Objectives:** We analyzed clinical outcomes from cancer cases profiled with SearchLight DNA, a canine cancer gene panel, to identify mutations with prognostic value.

**Animals:** One hundred twenty‐nine canine cancer cases analyzed with SearchLight DNA and for which clinical outcome information was available were evaluated.

**Methods:** Clinical data points were collected through record review. Variables including mutated genes, individual mutations, signalment, and treatment were fitted with univariate and multivariate Cox proportional hazard models to identify factors associated with progression‐free survival (PFS). The log‐rank test compared PFS between patients receiving and not receiving targeted therapy prior to first progression or death.

**Results:** Combined genomic and outcomes analysis identified 243 mutations in 89 genes across 27 cancer types. Four genes (SMARCB1, FANCG, CDKN2B, MSH6) were significantly associated with shorter PFS in univariate analysis. Patients who received targeted therapy prior to first progression or death (*n =* 45) were associated with a significantly longer PFS (139 days) compared to those who did not (*n =* 84; PFS = 108 days; *P* = 0.0104).

**Conclusions and Clinical Importance:** We demonstrate the progression‐free survival benefit of targeted therapy across multiple cancer types and identify novel mutations with prognostic value. This work highlights the potential for discovery of new actionable genomic information from clinical cases to benefit cancer management in dogs.

## Abstract O25: Assessing the Efficacy of Novel Caninized VEGF Monoclonal Antibody Therapy for Mammary Tumors in Dogs

### **Chen‐Si Lin** ^1^; Ciao‐Yun Chen^2^; Chia‐Yee Chun^2^; Tian‐Yu Lin^2^

#### ^1^National Taiwan University, Taipei City, Taiwan; ^2^Yung Shin Pharm. Ind. Co., Ltd

**Background:** Mammary tumors (CMTs) are commonly found in unspayed female dogs, with about 50% malignant. Surgery, chemotherapy, and radiation therapy are shown to have a high recurrence rate and poor prognosis for dogs with malignant CMTs. The high expression of vascular endothelial growth factor (VEGF) in malignant tumors is a protein that induces endothelial cell proliferation and migration and stimulates angiogenesis.

**Objective:** Targeted therapy against VEGF has been proven effective in suppressing tumor growth in many human cancer studies. This study developed a caninized VEGF monoclonal antibody (mAb) and evaluated its safety and efficacy in CMT dogs.

**Methods:** Four CMT dogs (grade II–IV) participated in this pilot study, three (cases 1 to 3) received 10 mg/kg antibody treatment every two weeks for a total of six times, and case 4 received the same dose of antibody treatment after surgery to remove the primary tumor.

**Results:** The cases were evaluated with physical and blood biochemistry examinations every two weeks, and no apparent adverse events were found in 3 of 4 cases. Cases 1, 2, and 4 completed six treatments. Based on the RECIST (v1.1) standard, case 1's tumor (grade II) achieved a complete response (CR), case 2's tumor (grade III) shrunk by 50% (PR), and case 4's tumor (grade IV) shrank by 20%.

**Conclusions and Clinical Importance:** This pilot study suggests that the caninized VEGF monoclonal antibody could effectively treat CMT‐bearing dogs; however, the possible side effects are needed for further study.

## Abstract O26: Anticancer Effect of Superoxide Dismutase on Canine Mammary Gland Tumor *In Vitro*

### **Ju‐Hyun An** ^1^; Byeong‐Gi Go^2^, DVM; Jeong‐Hwa Lee^2^, DVM, PhD; Kyeong‐Bo Kim^2^, DVM; Hwa‐Young Youn^3^, DVM, PhD

#### ^1^College of Veterinary Medicine, Kangwon National University, Chuncheon, Gangwon, Republic of Korea; ^2^Laboratory of Veterinary Internal Medicine, Department of Veterinary Clinical Science, College of Veterinary Medicine, Seoul National University, Seoul, Republic of Korea; ^3^Professor, Laboratory of Veterinary Internal Medicine, Department of Veterinary Clinical Science, College of Veterinary Medicine, Seoul National University, Seoul, Republic of Korea.

**Background:** Nearly 35% of malignant mammary gland tumors (MGT) have the potential to cause metastasis and dogs with lymph node metastasis or distant metastasis have a significantly shorter survival expectation than dogs without metastasis. Therefore, it is important to seek the factors that can control tumor proliferation and metastasis ability.

**Hypothesis/Objectives:** We applied SOD (superoxide dismutase) to canine MGT and confirmed the anticancer effect of SOD and its role in regulating the tumor microenvironment.

**Animals:** This is an *in vitro* experiment using a cell line derived from canine malignant MGT (CIPp and CIPm).

**Methods:** Viability assay and apoptosis assay were performed to determine the effect of superoxide dismutase (SOD) on cell survival. In addition, the cytoskeleton expression of tumor cells was confirmed through mRNA and western blot analysis.

**Results:** The tumor was suppressed by SOD and we confirmed that it was due to mechanisms other than apoptosis and necrosis. In addition, it was observed that the tumor's migration ability decreased, and it was confirmed that this was due to a decrease in mRNA and protein levels of cytoskeletons N‐cadherin and vimentin.

**Conclusions and Clinical Importance:** This is the first study to incorporate SOD into a canine mammary gland tumor, and it is expected that a new approach can be proposed through the control of the microenvironment.

## Abstract O27: Urinary Exosomal Nuclear Matrix Protein‐22 as a Novel Liquid Biopsy of Urothelial Carcinoma Dogs

### **Ju‐Hyun An** ^1^; Jee‐Hyo Shin^2^, DVM; Su‐Min Park^2^, DVM, PhD; Ga‐Hyun Lim^2^, DVM; Kyeong‐Won Seo^3^, DVM, PhD; Hwa‐Young Youn^3^, DVM, PhD

#### ^1^College of Veterinary Medicine, Kangwon National University, Chuncheon, Gangwon, Republic of Korea; ^2^Laboratory of Veterinary Internal Medicine, Department of Veterinary Clinical Science, College of Veterinary Medicine, Seoul National University, Seoul, Republic of Korea; ^3^Professor, Laboratory of Veterinary Internal Medicine, Department of Veterinary Clinical Science, College of Veterinary Medicine, Seoul National University, Seoul, Republic of Korea

**Background:** Nuclear matrix protein (NMP) 22 is found in higher‐than‐normal amounts in the urine of some types of cancer patients, including bladder cancer, and various studies are underway to use it as a cancer diagnostic tool. In particular, exosomes in urine are stable due to the lipid bilayer and resistant to enzymes and uric acid. Therefore, the use of urinary exosomes as a diagnostic and prognostic biomarker for urothelial carcinoma (UC) is currently being studied, but no study has been conducted to confirm NMP‐22 in urinary exosomes.

**Hypothesis/Objectives:** We aimed to investigate the clinical usefulness of urinary exosomal NMP‐22 in diagnosing urothelial carcinoma in dogs.

**Animals:** Total 30 dogs (UC group, *n =* 6; UC suspected, *n =* 6, control group, *n =* 17) were included in the analysis.

**Methods:** Among the canine patients who visited the veterinary hospital, urine was collected from patients who obtained consent from their owners, and after exosome was isolated from the urine, NMP‐22 was measured using ELISA.

**Results:** Compared to the control group and the UC suspected group, a significant difference was confirmed in the concentration of NMP‐22 in urine exosomes in the UC group.

**Conclusions and Clinical Importance:** It was confirmed that NMP‐22 was significantly increased in exosome urine of dogs diagnosed with UC, suggesting that exosome NMP‐22 can be considered as one of the liquid biopsies tools for diagnosing UC in dogs.

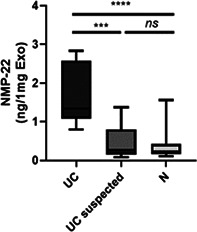

## Abstract O28: Phenotypic Description of T‐Cell Density in the Tumor Microenvironment of Canine Renal Cell Carcinoma

### **Ashleigh Cournoyer** ^1^; Hayley Amerman^1^; Charles Assenmacher^1^; Amy Durham^1^; Nicholas Keuler^2^; Matthew Atherton^1^, BVSc, PhD, DECVIM (Oncology); Jennifer Lenz^1^, DVM, DACVIM (Oncology)

#### ^1^University of Pennsylvania, Philadelphia, PA, USA; ^2^University of Wisconsin, Madison, WI, USA

**Background:** Tumor‐infiltrating lymphocytes (TILs) have been associated with prognosis in both human and canine malignancies. The number of intratumoral cytotoxic T‐lymphocytes (CTLs) have been positively associated with survival in human renal cell carcinoma (RCC) whereas the number of intratumoral T‐regulatory (Treg) cells have been negatively associated with RCC survival.

**Hypothesis/Objectives:** To evaluate the association of TIL density with survival times in canine RCC (CRCC). A secondary aim was to evaluate associations of clinicopathologic and histologic variables with CRCC survival times.

**Animals:** Fifteen dogs diagnosed with CRCC between 2008 and 2021.

**Methods:** Cases of CRCC were identified retrospectively and hematoxylin and eosin sections were reviewed to evaluate histologic prognostic variables (mitotic index, Fuhrman nuclear grade, vascular invasion, local invasion, histopathological subtype, sarcomatoid change). TIL density was quantified using immunohistochemistry (CD3, FoxP3, granzyme‐B) and subsequent manual or automatic cell counting via ImageScope software. Kaplan‐Meier curves were created to estimate progression free survival (PFS) and overall survival (OS). Potential prognostic subsets were compared using the log rank test.

**Results:** Median PFS and OS were 524 and 739 days, respectively. No significant association between CD3, FoxP3, or granzyme‐B positive TIL density with survival was identified. No histologic variable assessed was associated with patient outcome. Elevated GGT prior to nephrectomy was negatively associated with PFS on single variable analysis.

**Conclusions and Clinical Importance:** TIL density was not associated with prognosis in CRCC. Elevated GGT prior to nephrectomy was identified as a potential negative prognostic indicator.

## Abstract O29: BTK and BET Inhibition in Canine Lymphoma

### **Kelly R. Hume** ^1^; Danielle Hudak^2^; Dania Villarnovo^2^; Kristy Richards^2^ (deceased)

#### ^1^College of Veterinary Medicine, Cornell University, Ithaca, NY, USA; ^2^Cornell University, Ithaca, NY, USA

**Background:** Canine B‐cell lymphoma is a heterogenous group of neoplasms commonly treated with chemotherapy. Long‐term remissions are uncommon and novel therapeutics are needed.

**Hypothesis/Objectives:** The goal of our research was to evaluate tolerability and efficacy of combined inhibition of BTK (Bruton's tyrosine kinase) and BET (bromodomain and extraterminal domain) in dogs.

**Animals:** Three healthy, adult, research Beagles and five client‐owned dogs with B‐cell lymphoma.

**Methods:** First, a modified toxicity study was performed in research dogs. These dogs received the BET inhibitor mivebresib (0.1 mg/kg/day by mouth) and the BTK inhibitor ibrutinib (5 mg/kg/day by mouth) in combination for 4 weeks. Subsequently, 5 client‐owned dogs received mivebresib (0.05 mg/kg/day by mouth) and ibrutinib (5 mg/kg/day by mouth) in an 8‐week pilot study.

**Results:** During the modified toxicity study, 2 of 3 dogs experienced transient thrombocytopenia and one dog experienced self‐limiting decreased stool consistency. Median treatment duration in client‐owned dogs was 34 days (range, 7–57). Grade 2 diarrhea was the most common adverse event (*n =* 3). Mivebresib dosage was reduced in 2 dogs. No grade 3 or 4 toxicities were observed. Two dogs with stage V disease experienced a clinical response, specifically a decrease in size of target lesions of 21% and 38%, with the date of maximum response occurring after 6 weeks of treatment in both dogs.

**Conclusions and Clinical Importance:** Inhibition of BTK and BET has clinical activity in canine B‐cell lymphoma. Additional studies to fully define tolerability and efficacy are indicated.

## Abstract O30: A Retrospective Study on Conventional Transarterial Chemoembolization Using Idarubicin for Treatment of Canine Hepatic Carcinomas

### Sunghoon Jeon^1^, DVM, MS; Gahyun Lee^1^, DVM, MS; **Heejung Hong** ^2^, DVM, MS; Jinkyung Kim^1^, DVM, MS; Hyo‐Cheol Kim^3^, MD, PhD; Dongwoo Chang^4^, DVM, PhD

#### ^1^Haemaru Referral Animal Hospital; ^2^Laboratory of Veterinary Internal Medicine, Department of Clinical Veterinary Science, College of Veterinary Medicine, Seoul National University, Seoul, Republic of Korea; ^3^Department of Radiology, Seoul National University Hospital, Seoul, Republic of Korea; ^4^College of Veterinary Medicine, Chungbuk National University, Cheongju, Republic of Korea

**Background:** Idarubicin has been widely used in conventional transarterial chemoembolization (cTACE) in human patients due to its lipophilic properties. Though, there is lack of clinical study of cTACE using idarubicin in dogs.

**Hypothesis/Objectives:** To investigate the safety and tumor response of cTACE using idarubicin in dogs with hepatic carcinomas.

**Animals:** Nine dogs diagnosed with hepatocellular carcinoma (*n =* 8) and cholangiocarcinoma (*n =* 1) via cytologic or histologic examination.

**Methods:** A retrospective review of medical records that underwent cTACE using idarubicin. Data that was extracted from the medical records included signalment, clinical signs, body weight, serum biochemistry, pre‐ and post‐treatment (4 to 6 weeks) CT images, complications.

**Results:** Initial cTACE was technically successful in all dogs. Mild post‐embolization syndrome including anorexia (*n =* 5), lethargy (*n =* 2), abdominal pain (*n =* 5), fever (*n =* 1) was seen. There was no major complication such as hepatic abscess, cholecystitis. After treatment, clinical signs were improved, including loss of ascites (*n =* 1), increased body weight (*n =* 3) and appetite (*n =* 4). Serum biochemistry after 4 weeks revealed significantly decrease in ALT and AST levels (*P* < 0.05) compared to initial treatment. Based on elliptical tumor volume calculation, partial response (*n =* 7, decreased by average of 51.4%) was the most common outcome, followed by stable disease (*n =* 1), and progressive disease (*n =* 1).

**Conclusions:** Hepatic cTACE using idarubicin is feasible procedure with limited side effects for hepatic cancers. Alleviation of the clinical signs and tumor regression were seen in most patients.

## Abstract O31: Usefulness of Computed Tomography in Combination With Iodized Oil (Lipiodol) in Detecting Sentinel Lymph Nodes

### **Jin‐Young Kim** ^1^; Jeong‐Yeol Bae^1^, DVM; Guk‐Il Joung^1^, DVM; Jung‐Il Kim^1^, DVM; Joong‐Hyun Song, DVM, PhD, Professor, DKCVIM

#### ^1^Veterinary Teaching Hospital, Chungnam National University, Daejeon, Republic of Korea

**Background:** Visualization of sentinel lymph nodes (SLN) with computed tomography (CT) using iodized oil has not been thoroughly investigated yet.

**Hypothesis/Objectives:** To demonstrate methodological review and clinical recommendations of using CT in combination with lipiodol.

**Animals:** Five client‐owned dogs with subcutaneous tumors diagnosed with mammary gland tumors (*n =* 2) and mast cell tumors (*n =* 3) were included.

**Methods:** A pilot prospective study. Whole body radiography was taken before injection. Lipiodol of 0.5 or 1 mL, depending on the size of patient, was administered into four quadrants encircling the tumor using a 25‐G needle. After 24 h, both radiographic and CT images were taken. Surgically accessible identified lymph nodes were resected with primary tumor, followed by histopathology or cytology.

**Results:** A total of sixteen draining lymph nodes were identified on CT (16/16, 100%), while only eight of them (8/16, 50%) were clearly identified on radiography. Cytology or histopathology was performed in 5/16 (31.3%: CT, 5; radiograph, 3). Malignancy was identified in 2/5 (40.0%, CT, 2; radiograph, 1). Adverse effects were identified in one dog as granuloma adjacent to injection site. 3 dogs were capable of follow‐up radiography or CT scanning. Residence of lipiodol was identified at injection sites 42, 168, and 218 days after injection, respectively.

**Conclusions and Clinical Significance:** CT using iodized oil was able to better clarify SLN in dogs. Malignancy may be identified in draining nodes detected clearly only in CT, emphasizing clinical importance. Lipiodol should be used with caution regarding adverse effects and residual period.

Figure 1. Frog‐leg ventrodorsal radiographic view (A) of the caudal abdomen showing enhancement of mixed pattern around the right superficial inguinal lymph node (black arrow) 24 h after subcutaneous injection of 0.5 mL of lipiodol into the skin overlying the left fifth mammary gland and right fourth mammary gland. Lipiodol leakage from the nodes or overlapping with adjacent structures makes clear distinction of draining lymph notes difficult. CT image of the same dog taken 24 h after the injection (B) showed clear enhancement of the right superficial inguinal lymph node (white arrow), which was ambiguous to identify on the radiographic image. The right **superficial lymph node in this patient revealed malignancy confirmed by histopathology.**

**Figure:**
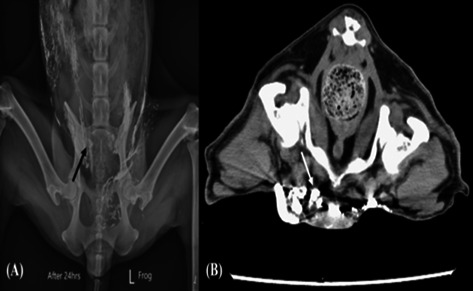
Figure 1

## Abstract O32: Transcriptome Profiling Between Normal and Lymphoma in Comparison of T‐Cell and B‐Cell Types in Dogs

### **Yeji Kim** ^1^; Jihyun Kim^1^; Yunji Song^1^; Songju Oh^1^; Geun‐Hwan Jang^1^; Ha‐Jung Kim^1^

#### ^1^College of Veterinary Medicine, Chonnam National University, Gwangju, Republic of Korea

**Background:** Several studies of B‐cell lymphoma (BCL) transcriptome analyses have been reported in dogs. However, no studies have been reported about the difference between T‐cell lymphoma (TCL) and BCL in dogs.

**Hypothesis/Objectives:** This study aims to identify differences in transcriptome profiling between normal and lymphoma as well as TCL and BCL in dogs.

**Animals:** Ten client‐owned dogs diagnosed with lymphoma in a hospital were enrolled in the patient group. Six dogs were classified as TCL, and four were BCL. Seven healthy beagles were also enrolled as the controls.

**Methods:** Transcriptome profiling was performed with tumor tissues obtained by ultrasound‐guided fine needle aspiration or biopsy. Data analysis was performed employing the fold change, independent T‐test, hierarchical clustering, gene‐enrichment and functional annotation analysis, and Kyoto Encyclopedia of Genes and Genomes pathways. Statistical significance was set at *P* value <0.05.

**Results:** Based on the threshold criteria, several differentially expressed genes (DEGs) were identified between normal and BCL (*n =* 2215). There were fewer significant DEGs between the normal and TCL (*n =* 125), and various expression patterns were identified among each patient. Also, DEGs such as JUN, CCL4, and PIK3CD, known to be related with tumor malignancy, were significantly upregulated in TCL compared to BCL (Table 1).

**Conclusions and Clinical Importance:** Significant DEGs were identified between TCL and BCL, and these DEGs are presumed to have been related to poor treatment response and prognosis of TCL. This is the first study of transcriptome profiling between normal and lymphoma in the comparison of TCL and BCL in dogs.

**Table:** Table 1. Fold change and *P* value of DEGs between TCL and BCL

DEGs	Fold change	*P* value
JUN	3.765477	0.000896232
CCL4	10.14129	0.025482128
ANAPC4	−1.63589	0.02913123
PIK3CD	2.32213	0.030929825
CXCL12	4.133969	0.03678425
ANAPC10	−1.55833	0.039219851
IKFZ2	11.25469	0.043498788
LCK	4.751128	0.047681921

## Abstract O33: The National Cancer Institute's Comparative Osteosarcoma Pathology Board: Toward Harmonized Grading for Humans and Dogs

### **Amy K. LeBlanc** ^1^; Jessica Beck^2^, DVM, PhD, DACVP; Christina Mazcko^3^; Pooja Hingorani^4^, MD; Troy McEachron^5^, PhD; Ryan Roberts^6^, MD, PhD; Hibret Adissu^7^, DVM, PhD, DACVP; Jonathan Bush^8^, MD, FRCPC; David Parham^9^, MD; Courtney Schott^10^, DVM, PhD, DACVP; Poul Sorensen^11^, MD, PhD; Geoffrey Wood^12^, DVM, PhD, DVSc

#### ^1^Comparative Oncology Program, Center for Cancer Research, National Cancer Institute, National Institutes of Health; ^2^Staff Scientist and Investigative Pathologist, Comparative Oncology Program, Center for Cancer Research, National Cancer Institute; ^3^Center for Cancer Research, National Cancer Institute; ^4^Medical Director, Oncology Early Development, AbbVie Inc; ^5^Head, Integrated Solid Tumor Biology Section, Pediatric Oncology Branch, Center for Cancer Research, National Cancer Institute; ^6^Department of Pediatrics, Division of Hematology and Oncology, Nationwide Children's Hospital, Columbus, OH, USA; The Ohio State University, Columbus, OH, USA; ^7^Director of Pathology, Respiratory and Immunology, Clinical Pharmacology and Safety Sciences, AstraZeneca R&D; ^8^Clinical Associate Professor, Department of Pathology and Laboratory Medicine, University of British Columbia, Vancouver, Canada; ^9^Emeritus Member, Children's Oncology Group; ^10^Assistant Professor, Department of Pathobiology, Ontario Veterinary College, University of Guelph, Guelph, Canada; ^11^Professor, Department of Pathology, University of British Columbia, Vancouver, Canada; ^12^Associate Professor, Department of Pathobiology, Ontario Veterinary College, University of Guelph, Guelph, Canada

**Background:** Key gaps in knowledge persist as to the true translatability of canine osteosarcoma (OS) to its human counterpart, inclusive of histologic, genomic, and transcriptomic features. Under the NCI's Decoding the Osteosarcoma Genome of the Dog (DOG2) project, we are addressing these gaps through a comparative OS pathology board comprised of both physician and veterinary pathologists.

**Hypothesis/Objectives:** Specific objectives are: (1) compare/contrast canine and human OS histopathologic features, (2) establish updated diagnostic criteria for canine OS, with insight from physician sarcoma pathologists, and (3) incorporate molecular characterization to further validate dogs as a suitable model for translational drug development and biomarker studies.

**Animals:** Treatment‐naive primary OS tumor samples were subjected to veterinary pathologist QA/QC prior to inclusion. A sample/data archive was created using LabMatrix to organize and track samples, images, and accompanying clinical and genomic/transcriptomic datasets.

**Methods:** Monthly focused discussions are underway on specific comparative aspects of canine vs. human OS. Specific histologic features and patterns are discussed and compared by board members. A revised classification and grading scheme is being developed to harmonize the approach between species.

**Results:** Initial observations of *n =* 200 canine OS primary tumors find similar incidence of hallmark cell types and histologic patterns in both species (osteoblastic and chondroblastic differentiation, vessel‐rich and necrotic regions, giant cell‐rich regions, immune cell infiltration, and presence of lymphoid aggregates).

## Abstract O34: The National Cancer Institute's Decoding the Osteosarcoma Genome of the Dog (DOG2) Project

### **Amy K. LeBlanc** ^1^; Kate Silver^2^; Jessica Beck^2^; Christina Mazcko^2^

#### ^1^Comparative Oncology Program, Center for Cancer Research, National Cancer Institute, National Institutes of Health; ^2^Comparative Oncology Program, Center for Cancer Research, National Cancer Institute

**Background:** Key gaps in knowledge persist in the translatability of canine osteosarcoma (OS) to its human counterpart, inclusive of histologic, genomic, and transcriptomic features. Under the NCI's Decoding the Osteosarcoma Genome of the Dog (DOG2) project, we are addressing these gaps through a multi‐omic approach.

**Hypothesis/Objectives:** To leverage the DOG2 outcome‐linked OS biorepository to 1. identify prognostic signatures for canine OS that could be translated to human patients and 2. to identify new druggable targets that can be biologically and experimentally validated.

**Animals:** 324 dogs with osteosarcoma were enrolled in 2 NCI‐COTC clinical trials, resulting in over 22,000 outcome‐linked samples. A sample/data archive was created using LabMatrix to organize and track samples, images, and accompanying clinical and genomic/transcriptomic datasets.

**Methods:** We have completed pathology review and nucleic isolation from primary OS tumors, normal tissues, and metastatic samples. Analytical platforms include bulk and single‐nuclei RNAseq, whole genome sequencing, whole exome sequencing, methylation profiling, multiplex IHC, and Nanostring IO profiling.

**Results:** See table for a summary of sample numbers and analysis platforms.

**Conclusions and Clinical Importance:** The DOG2 biospecimen repository is enabling the creation of a clinical, genomic, and pathologic framework for canine OS, which will further enhance the translational value of the pet dog as a naturally occurring animal model for humans.

**Table:** Table 1. Overview of canine osteosarcoma samples subjected to ‐omic analyses under the DOG2 project

	Tumor samples	Normal samples	Metastatic samples	Total samples
Bulk RNA sequencing	189	47	23	**259**
Whole exome sequencing	69	55	22	**146**
Whole genome sequencing (low pass, 2×)	68	55	22	**145**
Whole genome sequencing (60–80×)	66	55	22	**143**
Whole genome methylation (bisulfite seq)	47	0	22	**69**
Nanostring IO frozen samples	10	0	14	**24**
Nanostring IO FFPE samples	12	12	84	**108**

## Abstract O35: Macrophage‐Induced Chemoresistance in Canine Mammary Gland Tumor Cell Spheroid Model

### **Ga‐Hyun Lim** ^1^; Ju‐Hyun An^2^, DVM, PhD, Professor; Su‐Min Park^3^, DVM, PhD; Kyoung‐Won Seo^3^, DVM, PhD, Prof; Ga‐Hee Youn^3^, DVM; Hwa‐Young Youn^3^, DVM, PhD, Prof

#### ^1^Seoul National University, Seoul, Republic of Korea; ^2^Department of Veterinary Emergency and Critical Care Medicine, Institute of Veterinary Science, College of Veterinary Medicine, Kangwon National University, Chuncheon, Gangwon, Republic of Korea; ^3^Laboratory of Veterinary Internal Medicine, Department of Veterinary Clinical Science, College of Veterinary Medicine, Seoul National University, Seoul, Republic of Korea

**Background:** Tumor‐associated macrophages (TAMs) act in multi‐functional roles in tumor progression, immune regulation, metastasis, angiogenesis and chemoresistance. Hypoxia in the tumor microenvironment induces M2 polarization of TAMs, which enhances chemoresistance.

**Hypothesis/Objectives:** In this study, a hybrid spheroid model of canine mammary gland tumor (MGT) cell lines (CIPp, CIPm) and canine macrophages (DH82) was established to mimic tumor microenvironment. We aim to investigate the effects of hypoxia induced by the spheroids model on the chemoresistance of canine MGT cells. We also wanted to investigate the interactions between canine MGT cells and macrophages under doxorubicin.

**Animals:** This study didn’t use animals.

**Methods:** *In vitro* study. Hybrid spheroids were created using ultralow adhesion plate. The interactions between canine MGT cells and macrophages were investigated through gene expression, cell viability, apoptosis, and cell cycle arrest.

**Results:** We confirmed the effects of hypoxia on the growth factors and multi‐drug resistance of canine MGT cells in the spheroid model. Moreover, infiltration of M2 polarized macrophage in the spheroid model was identified. Finally, we confirmed that macrophages induce chemoresistance by examining the effects of doxorubicin on growth, apoptosis, cell cycle arrest, and multi‐drug resistance gene expression in canine MGT cells.

**Conclusions and Clinical Importance:** We suggest that the crosstalk between macrophages and canine MGT cells contributes to chemoresistance in tumor cells, thus indicating that macrophages are an important factor in tumor cell resistance to anti‐cancer drugs. The hybrid spheroid model can be applied to anti‐cancer drug research in the future.

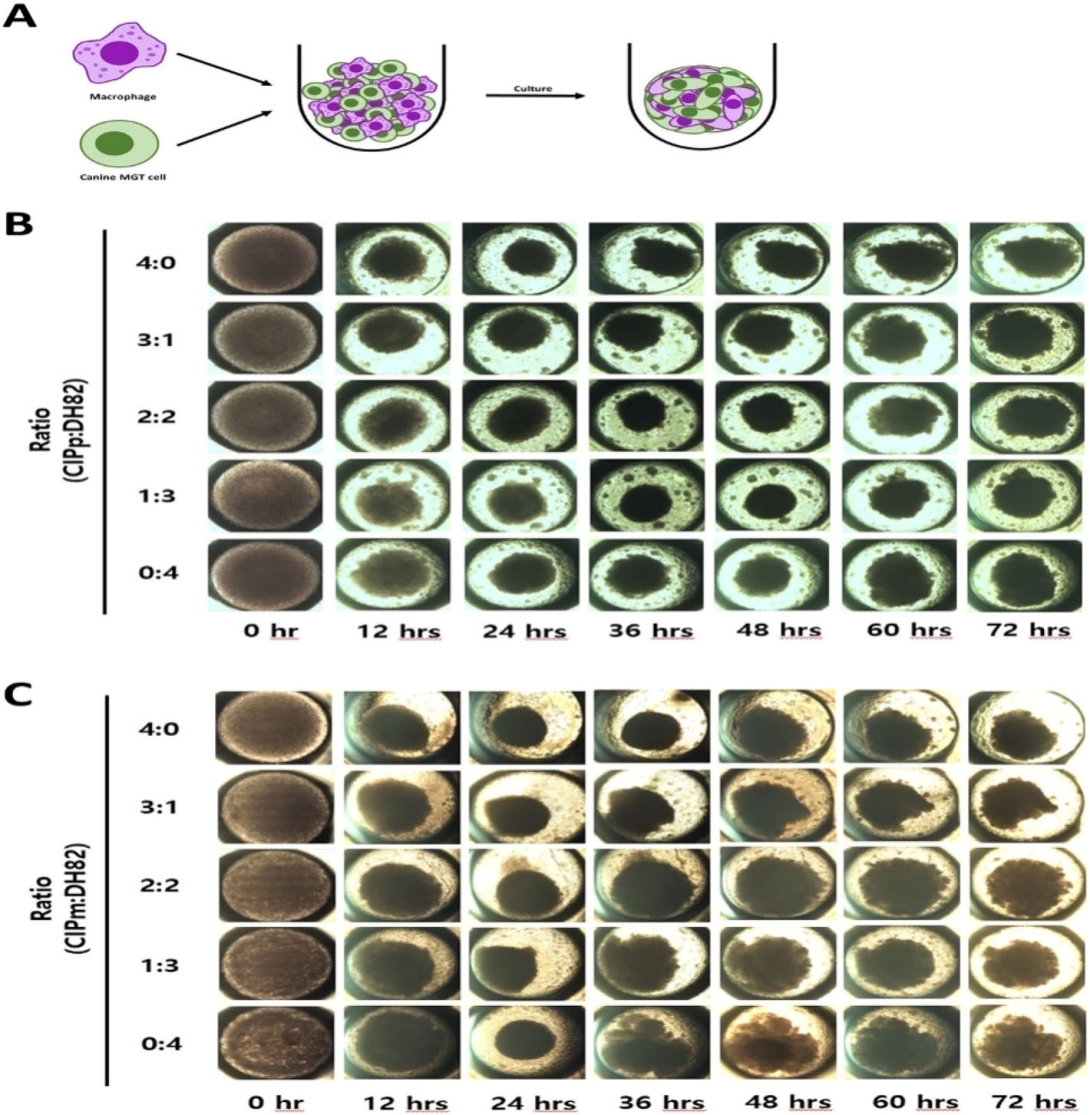

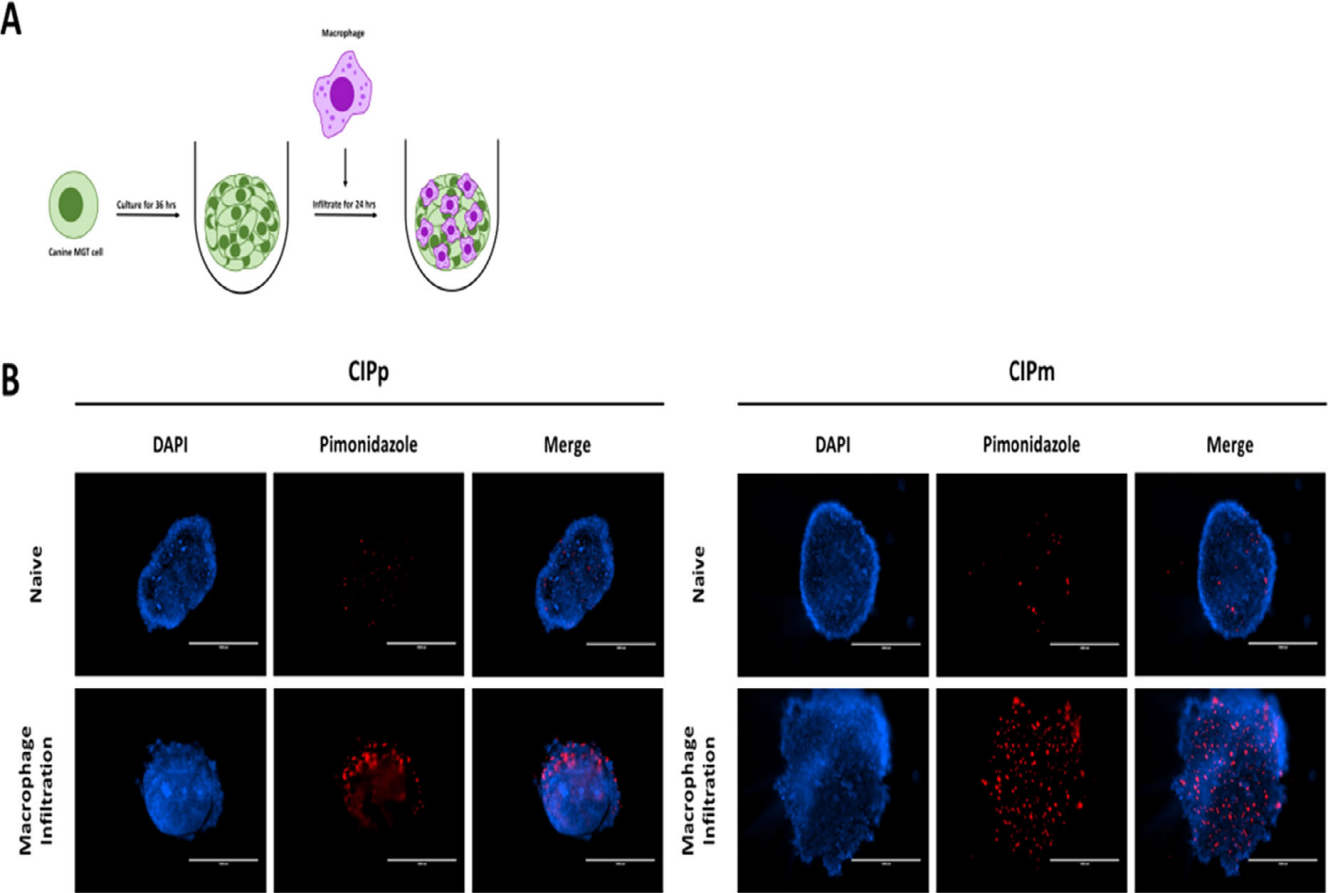

## Abstract O36: Clinical Outcome of Multicentric Lymphoma Treated With CHOP Protocol in 38 Small‐Breed Dogs

### **Woo‐Jin Song** ^1^; Minkun Kim^1^; Saeyoung Lee^1^; Jongjin Park^1^; Youngmin Yun^1^; Hwa‐Young Youn^2^

#### ^1^Jeju National University, Jeju, Republic of Korea; ^2^Seoul National University, Seoul, Republic of Korea

**Background:** Lymphoma is one of the most common malignant tumors in dogs. Combination chemotherapy with vincristine, cyclophosphamide, doxorubicin and prednisolone (CHOP) is the most effective treatment for multicentric lymphoma. Previous studies have evaluated the response of large dogs to CHOP treatment and identified prognostic factors; however, studies on small‐breed dogs are lacking.

**Objectives:** The aim of this study was to evaluate the outcomes and prognostic factors in multicentric lymphoma small‐breed dogs (weighing under 15 kg) administered the CHOP protocol.

**Animals:** Thirty‐eight small‐breed dogs were included.

**Methods:** Retrospective study

**Results:** The responses of lymphoma dogs to CHOP treatment were assessed, and 54.3% were evaluated as being in complete remission (CR), 31.4% in partial remission (PR), and 14.3% in no remission (NR). The overall response rate was 85.7%. The mean survival time (MST) of all patients was 490.6 ± 474.5 days. MSTs for CR, PR, and NR patients were 666.9 ± 436.7, 297.2 ± 263.3, and 170.8 ± 136.0 days, respectively. Among the CR patients, MST was longer under the following conditions: age under 10 years (*P* = 0.011), no cardiovascular heart disease (*P* = 0.002), and no history of hospitalization due to side effects from chemotherapy (*P* = 0.031).

**Conclusion and Clinical Importance:** These results might help clinicians build treatment plans for multicentric lymphoma in small‐breed dogs.

**Figure:**
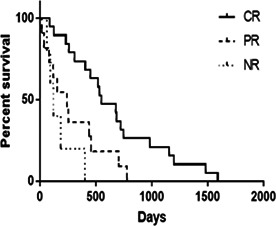
Figure 1. Variation of survival time in each response group**.** Mean survival time (MST) and the range is 666.9 (±436.7) days and 85 to 1588 days in complete remission (CR), 297.2 (±263.3) days and 61 to 400 days in non‐remission (NR). MST of CR is statistically significantly longer than PR or NR (*P* = 0.029, *P* = 0.027 respectively). The results are presented as the mean ± standard deviation, as determined by one‐way ANOVA).

**Figure:**
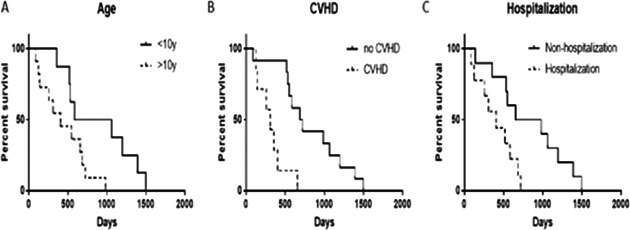
Figure 2. Distribution of different survival time according to influencing factors**.** (A) age, (B) presence of chronic valvular heart disease (CVHD), and (C) hospitalization make a difference in the survival time. The results are presented as the mean ± standard deviation, as determined by Student's *t*‐tests.

## Abstract O38: Evaluation of Mechlorethamine, Vinblastine, Procarbazine, and Prednisone for Treatment of Resistant Multicentric Canine Lymphoma

### **Kelley Zimmerman** ^1^; Koranda Walsh^1^; Nicholas Keuler^2^; Matthew Atherton^1^; Jennifer Lenz^1^

#### ^1^University of Pennsylvania, Philadelphia, PA, USA; ^2^University of Wisconsin–Madison, Madison, WI, USA

**Background:** Most naïve high‐grade canine lymphoma patients treated with standard of care multiagent chemotherapy will achieve complete remission; however, disease recurrence is common, and rescue protocols are needed to reinduce remission. MOPP (mechlorethamine, vincristine, procarbazine, and prednisone) is an effective rescue protocol, but associated with gastrointestinal toxicity and can be a less desirable option for patients that previously failed vincristine‐containing protocols. Previous studies have suggested cross‐resistance between members of the vinca alkaloid family may be incomplete; thus, vinblastine may be used as a substitute for vincristine to reduce gastrointestinal toxicity and chemoresistance.

**Hypothesis/Objectives:** The objective of this study was to report clinical outcomes and toxicity in dogs with relapsed or refractory multicentric lymphoma treated with a modified MOPP protocol whereby vincristine was replaced with vinblastine (MVPP).

**Animals:** Thirty‐six client‐owned dogs with relapsed or refractory lymphoma treated with MVPP.

**Methods:** Medical records of patients treated with MVPP between 2009–2021 were retrospectively reviewed. Clinical responses and toxicities were recorded in line with Veterinary Cooperative Oncology Group recommendations.

**Results:** The overall response rate to MVPP was 25.7% with a median progression‐free survival of 15 days and a median overall survival of 45 days. The median number of chemotherapy protocols prior to MVPP was 3 (range 1–8). MVPP at the prescribed doses was well tolerated with no treatment delays or hospitalizations secondary to side effects.

**Conclusions and Clinical Importance:** MVPP resulted in modest and transient anti‐tumor activity. Given the minimal toxicity, vinblastine dose intensification could be considered to improve clinical responses.

## Abstract EN01: Expression of Steroidogenic Enzymes in Canine Functional and Non‐functional Adrenocortical Tumors

### **Frederik Allan**; Alice Watson; Harriet Syme

#### Royal Veterinary College, London, UK

**Background:** Functionality of human adrenal tumors is inferred by CYP11B1 (cortisol synthase) and/or CYP11B2 (aldosterone synthase) expression. However, dogs have a single multi‐functional CYP11B enzyme so alternative strategies are required to interrogate functionality. It has been proposed CYP17 expression controls production of cortisol, rather than aldosterone, by adrenocortical cells in dogs.

**Hypothesis/Objectives:** Non‐functional canine adrenal tumors will have low expression of steroidogenic enzymes, while aldosterone‐producing tumors express CYP11B, and cortisol‐producing tumors both CYP11B and CYP17.

**Animals:** 17 dogs with adrenal tumors (8 non‐functional, 7 cortisol‐producing and 2 aldosterone‐producing) and two with normal adrenal glands.

**Methods:** Adrenal functionality was determined from clinical signs and endocrine testing. CYP11B and CYP17 expression were detected by immunohistochemistry on formalin‐fixed paraffin‐embedded adrenal tissue. Protein expression was semi‐quantified by two blinded observers using H‐scoring and compared in non‐functional and cortisol‐producing adrenal tumors using Mann‐Whitney U tests. Results are reported as median [range].

**Results:** Normal adrenal zona glomerulosa was CYP11B positive, zona fasciculata and reticularis were CYP11B and CYP17 positive. Expression of CYP11B (190 [130–265] vs. 147.5 [95–202.5]; *P* = 0.07) and CYP17 (177.5 [87.5–240] vs. 247.5 [55–292.5]; *P* = 0.397) was not significantly different between cortisol‐producing and non‐functional canine adrenal tumors respectively. Expression of CYP11B (142.5, 230) and CYP17 (95, 220) in aldosterone‐producing adrenal tumors was similar to that of the other tumors.

**Conclusions and Clinical Importance:** Retrospective determination of functionality of canine adrenal tumors using immunohistochemistry is difficult. The mechanisms for differential control of cortisol and aldosterone synthesis in species with a single CYP11B enzyme remain poorly understood.

## Abstract EN02: The Impact of Single‐Dose Trazodone on Endogenous Adrenocorticotropic Hormone and Cortisol Concentrations in Healthy Dogs

### **Morgan Brown** ^1^; Tekla Lee‐Fowler^2^, DVM, MS, DACVIM (SAIM); Ellen Behrend^3^, DVM, MS, PhD, DACVIM (SAIM); Megan Grobman^2^, DVM, MS, PhD, DACVIM (SAIM)

#### ^1^College of Veterinary Medicine, Auburn University, Auburn, AL, USA; ^2^Auburn University, Auburn, AL, USA; ^3^VIN, Davis, CA, USA

**Introduction:** Conditions affecting the hypothalamic‐pituitary‐adrenal (HPA) axis are common in dogs. Testing the function of the HPA axis includes measurement of endogenous adrenocorticotropic hormone (eACTH) and/or adrenocorticotropic hormone (ACTH) stimulation test. Trazodone is an anxiolytic commonly administered to dogs to reduce stress. In humans, trazodone significantly decreases plasma cortisol concentration via alpha‐1 adrenergic activity. The aims of this study were to determine the impact of trazodone on eACTH and serum cortisol concentrations in healthy dogs.

**Animals:** Fourteen healthy, adult, privately‐owned dogs were prospectively enrolled.

**Methods:** Randomized placebo‐controlled study. Trazodone (8–10 mg/kg) or placebo was administered orally 1 hour prior to eACTH measurement and ACTH stimulation testing. After a ≥7‐day wash‐out, dogs received the opposite treatment. Differences in eACTH, pre‐ and post‐ACTH stimulation cortisol levels, and delta (difference between pre‐ and post‐ACTH) cortisol concentrations were analyzed using a Paired T or Signed Rank Test with a *P* < 0.05 significance level.

**Results:** The eACTH concentrations were not significantly different (*P* = 0.233) between treatments. Similarly, no significant differences were found in the pre‐ACTH cortisol concentrations between treatments (*P* = 0.401). There was a significant difference between treatments for post‐ACTH cortisol concentrations (*P* = 0.049) and delta cortisol concentrations (*P* = 0.042). Two dogs receiving trazodone had post‐ACTH cortisol concentrations below the reference range compared to no dogs receiving placebo.

**Conclusions and Clinical Importance:** Preliminary data suggest trazodone administration may dampen the adrenocortical response to stimulation in healthy dogs. If similar effects are found in dogs with hyper‐ or hypoadrenocorticism, use of trazodone may impact clinical decision making in these populations.

## Abstract EN03: Evaluating a Second‐Generation Flash Glucose Monitoring System in Nondiabetic Dogs With Rapidly Induced Hypoglycemia

### **Jessica Herman** ^1^; Jonathan Lidbury^2^, BVMS, MRCVS, PhD, DACVIM, DECVIM‐CA; Nicholas Jeffery^3^, DVM, PhD; Shannon Washburn^4^, DVM, PhD; Carly Patterson^5^, DVM, DACVIM

#### ^1^College of Veterinary Medicine, Texas A&M University, College Station, TX, USA; ^2^Associate Professor, Veterinary Teaching Hospital, Texas A&M University, College Station, TX, USA; ^3^Veterinary Teaching Hospital, Texas A&M University, College Station, TX, USA; ^4^Veterinary Medicine & Biomedical Sciences, Texas A&M University, College Station, TX, USA; ^5^Clinical Assistant Professor, Veterinary Teaching Hospital, Texas A&M University, College Station, TX, USA

**Background:** A flash glucose monitoring system (FGMS; FreeStyle Libre 14‐day system, Abbott Laboratories) was commonly utilized for the management of canine diabetes but has been discontinued and replaced by a second‐generation system (FreeStyle Libre2, Abbott Laboratories).

**objective(s):** To assess the utility of a second‐generation FGMS in non‐diabetic dogs during rapidly induced hypoglycemia.

**Animals:** Twenty‐three apparently healthy teaching dogs.

**Methods:** Prospective, observational study performed in tandem with a teaching laboratory. Regular insulin was administered to dogs and resulting hypoglycemia corrected. Before insulin administration and every 10 min over a 90‐min period, serial measurements of interstitial glucose (IG) with FGMS and blood glucose (BG) with a portable blood glucose meter (PBGM) were made. At each time point, readings of both PBGM and FGMS were compared to a clinical chemistry analyzer BG concentration as a gold standard. Analytical and clinical accuracy were assessed using ISO 15197:2013 criteria, including Parkes error grid analysis.

**Results:** The proportions of readings in the low BG range (BG <100 mg/dL) for which the test method measurement was within ±15 mg/dL of the reference BG for the PBGM and FGMS were 66.7% (132/198) and 45.1% (69/153), respectively. The proportions of readings for the PBGM and FGMS, which were not likely to affect clinical outcome according to Parkes error grid analysis, were 94.92% (217/230) and 88.2% (157/178), respectively.

**Conclusions and Clinical Importance:** In this model, there was limited agreement between the FGMS and reference standard BG measurements. The FGMS failed to reliably detect hypoglycemia.

## Abstract EN04: Selective Osmotic Shock for the Isolation of Feline Pancreatic Islets of Langerhans

### **Lauren T. Porter** ^1^; Christopher Adin^2^, DVM, DACVS; Chiquitha Crews^3^, CVT, MS; Jocelyn Mott^4^, DVM, DACVIM (SAID); Chen Gilor^5^, DVM, PhD, DACVIM (SAIM)

#### ^1^University of Florida, Gainesville, FL, USA; ^2^Executive Associate Dean, Dean's Office, College of Veterinary Medicine, University of Florida, Gainesville, FL, USA; ^3^Laboratory Technical Staff, Small Animal Clinical Sciences, University of Florida, Gainesville, FL, USA; ^4^Post‐Doctoral Associate in Feline and Canine Diabetes, Small Animal Clinical Sciences, University of Florida, Gainesville, FL, USA; ^5^Associate Professor, Small Animal Clinical Sciences, University of Florida, Gainesville, FL, USA

**Background:** Pancreatic islet isolation is critical for studying islet physiology and transplantation. Traditional protocols use collagenases, causing injury to islets. In cats, collagenase protocols fail to produce functional islets, as demonstrated by glucose‐stimulated insulin secretion (GSIS). Since islet cells express GLUT‐2 they may adapt to hyperosmolar glucose solutions, enabling islet isolation by selective destruction of exocrine tissue.

**Objective:** To compare islet yield, purity, morphology, and GSIS between four protocols for islet isolation using selective osmotic shock (SOS).

**Animals:** Pancreata were obtained from cats in which necropsy or pancreatectomy was performed for reasons unrelated to this study.

**Methods:** Pancreatic tissue was mechanically disrupted and incubated in hyperosmolar RPMI solution with either 300 mmol/L (protocol A and B) or 600 mmol/L glucose (protocol C and D) for 20 (A and C) and 40 min (A and D). Islets were cultured for 24–48 h. Using light microscopy, islets were quantified and assessed for purity and morphology. GSIS was measured by incubating approximately 120 islets from each protocol for 60 min in low (2.8 mM) and high (28 mM) glucose concentrations. GSIS was calculated as [insulin] at 28 mM glucose/[insulin] at 2.8 mM glucose. Data are presented as median and range.

**Results:** Islet yield was moderate (713 islet/g, [407–2261]) and morphology excellent across protocols. Protocol C resulted in the highest GSIS stimulation index (2.2 [1.0–3.6], *P* = 0.05).

**Conclusions:** SOS resulted in isolation of functional feline islets and will allow *in vitro* studies. Optimization to improve purity and yield are required before use in clinical islet transplantation.

## Abstract EN05: Effect of Sodium‐Glucose Cotransporter 2 Inhibitor, Canagliflozin, on Interstitial Glucose in Diabetic Insulin Treated Dogs

### **Jessica R. Box** ^1^; Mark Oyama^1^, DVM, MSCE, DACVIM; Ariel Mosenco^1^, DVM, DACVIM; Rebecka Hess^2^, DVM, MSCE, DACVIM

#### ^1^Department of Clinical Sciences & Advanced Medicine, School of Veterinary Medicine, University of Pennsylvania, Philadelphia, PA, USA; Professor of Internal Medicine, Internal Medicine, Department of Clinical Sciences & Advanced Medicine, School of Veterinary Medicine, University of Pennsylvania, Philadelphia, PA, USA

**Background:** Sodium‐glucose cotransporter 2 inhibitors (SGLT2i) are approved for use in some diabetic cats. The utility of SGLT2i has not been reported in insulin treated diabetic dogs.

**Hypothesis:** Canagliflozin, an oral SGLT2i, decreases interstitial glucose (IG) concentrations in insulin treated diabetic dogs.

**Animals:** Four insulin treated diabetic dogs from a university teaching hospital population.

**Methods:** Ongoing uncontrolled open label longitudinal study. Diabetic dogs are enrolled if they have an unchanged insulin dose for at least 2 weeks prior to enrollment, IG ≤300 mg/dl for at least 2 h/day in one of the 3 days preceding canagliflozin treatment initiation, no vomiting, diarrhea, lower urinary tract clinical signs, or clinical signs consistent with diabetic ketoacidosis, a body weight ≥7 kg, and remaining on the same diet for the study duration. A flash glucose monitoring sensor is placed on day 0, and IG is recorded until day 3, when canagliflozin treatment at 2–4 mg/kg, orally, once a day, begins and is continued until day 10. Insulin treatment continues at the same dose it has been given for the preceding 2 weeks.

**Results:** Median IG concentration in 2372 measurements obtained while dogs were treated with insulin and canagliflozin was 85 mg/dl (range, 40–455 mg/dl) and was significantly lower than median IG concentration in 1,111 measurements obtained while dogs were treated with insulin and no canagliflozin (208 mg/dl, range, 41–500 mg/dl).

**Conclusions and Clinical Importance:** Canagliflozin may have a role in improving glycemic control in insulin treated diabetic dogs.

## Abstract EN06: A Comparison of Biochemical Findings in Cats With Diabetes Mellitus Between Different Body Condition Categories

### **Sophie E. Broughton** ^1^; Amrita Mohanty^2^; Elizabeth Brenhouse^1^; Freja Kragh Joergensen^3^; Dong Xia^1^; Tracy Van Der Merwe^1^; Mette Rasmussen^3^; Charlotte Bjoernvad, DVM, PhD, DECVCN^3^; John Flanagan^4^; Lucy Davidson^5^; Ruth Gostelow^1^

#### ^1^Royal Veterinary College, London, UK; ^2^Veterinarian, Royal Veterinary College, University of Copenhagen, København, Denmark; ^3^University of Copenhagen, København, Denmark; ^4^Royal Canin, Aimargues, France; ^5^University of Oxford, Oxford, UK

**Background:** Obesity is a risk factor for type 2‐like diabetes mellitus (DM) in cats, but DM also occurs in non‐obese cats. It is unknown if DM in non‐obese cats is biochemically distinct from obesity‐associated DM.

**Hypothesis/Objectives:** Evaluation of data from cats with DM will reveal biochemical differences associated with body condition.

**Animals:** Cats diagnosed with type 2‐like DM excluding those with insulin‐like growth factor (IGF‐1) >1000 ng/ml, at first presentation to a single feline referral DM clinic between December 2013 and December 2022 (*n =* 111).

**Methods:** Retrospective cohort study. Signalment, anamnesis and biochemical values were statistically compared (parametric: one‐way ANOVA; non‐parametric: Kruskal‐Wallis) among cats classified into the following body condition score (BCS) categories: underweight (1–3), normal (4–5), overweight (6–7), obese (8–9).

**Results:** BCS of 111 cats were classified as underweight (*n =* 17), normal (*n =* 24), overweight (*n =* 52), obese (*n =* 18). Sex (*P* = 0.54), age at diagnosis (*P* = 0.358), and insulin dose (total dose *P* = 0.37, units/kg *P* = 0.34) did not differ among groups. Underweight cats had a shorter median (range) duration of insulin treatment (days) [45(12–116)] than obese cats [129(29–768)] (*P* = 0.018). Mean (±SD) fructosamine was greater in underweight (533.82 ± 103.28 mmol/l) than overweight (436.25 ± 124.81 mmol/l; *P* = 0.022), and nearing significance in obese cats (428.18 ± 97 mmol/l; *P* = 0.051). Median (range) total T4 was significantly lower in non‐overweight (16.6(5.8–31.5 nmol/l)) than in overweight and obese cats (20.3(5.7–47.4 nmol/l); *P* = 0.004). Median (range) IGF‐1 was lower in underweight (296(171–489) ng/ml) than overweight (481(95–985) ng/ml; *P* = 0.004) and obese (572(196–902) ng/ml; *P* = 0.002) cats.

**Conclusions and Clinical Importance:** Underweight cats had biochemical changes consistent with poorer diabetic control. Variation in IGF‐1 by body condition has not been reported in non‐diabetic cats.

## Abstract EN07: The Effect of Storage Conditions on Canine Free Thyroxine Measurement by Chemiluminescence and Bulk‐Acoustic‐Wave Assays

### **Susan V. Carr** ^1^; Jennifer Hawley^2^, ACRP‐CP; Michael Lappin^3^

#### ^1^Colorado State University, Ft. Collins, CO, USA; ^2^Senior Research Associate, Colorado State University, Ft. Collins, CO, USA; ^3^Professor & Director, Companion Animal Studies, Department of Clinical Sciences, Colorado State University, Ft. Collins, CO, USA

**Background:** Behrend et al (1998) showed serum samples stored at 37C had increased canine free thyroxine (fT4) results when measured by radioimmunoassay. Since then, chemiluminescence (CLIA) and Bulk Acoustic Wave (BAW) assay methods have been developed, however, it is unknown if similar increases will be seen when samples are exposed to heat.

Due to concerns over fT4 stability, previously frozen samples used for research purposes should also be evaluated for post‐thaw quality.

**Hypotheses:**

3 fT4 concentration is not different between samples analyzed fresh on‐site and the same sample after a freeze‐thaw cycle.
4 fT4 will increase with warm‐temperature exposed samples compared to controls when analyzed by CLIA and BAW technology.

**Animals:** Eleven dogs receiving thyroid evaluation at a teaching hospital.

**Methods:** Phase 1: An experimental study measuring fT4 by CLIA (IMMULITE 2000, Siemens Medical Solutions, USA) on a fresh serum sample and thawed serum samples.

Phase 2: A prospective placebo‐controlled evaluation of the effects of heat exposure. Samples were divided into two; a control sample kept at 4 °C overnight, and a test sample exposed to 37C. Both were tested for fT4 using both CLIA and BAW (TRUFORMA, Zomedica Inc, USA) analyzers.

**Results:** The fT4 on thawed samples was significantly different compared to pre‐freezing (Figure 1) (*P* = 0.0006), with a mean sample difference of 0.69 ng/dL. Heat‐exposed samples were variably and unpredictably affected compared to controls (Figure 2).

**Conclusions:** Frozen storage and heat exposure affect fT4 results. Minimizing these pre‐analytic variables is critical for accurate results and correct interpretation of thyroid status.

**Figure:**
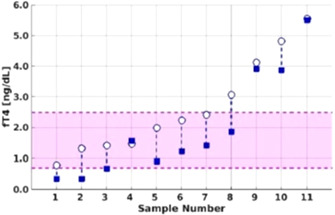
Figure 1. fT4 pre and post freeze‐thaw as measured by CLIA. Open circles represent same day on‐site serum fT4 analysis, solid squares represent fT4 after undergoing a freeze‐thaw cycle. The shaded pink section represents the normal reference range for the CLIA analyzer.

**Figure:**
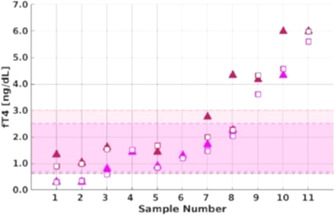
Figure 2. fT4 in refrigerated vs. heat‐exposed samples as measured by CLIA and BAW. Open squares represent samples maintained at 4C overnight, and solid triangles represent samples at 37C overnight. Pink squares represent analysis by CLIA and the pink‐shaded area represents the normal reference range for this analyzer. Red squares and triangles represent analysis by a BAW assay and the red‐shaded area represents the normal reference range for the BAW analyzer.

## Abstract EN09: Untargeted Serum Metabolomic Profiles in Hyperthyroid Cats Before and After I‐131 Therapy

### **Molly Bechtold** ^1^; Carol Frederick^2^; Lucinda Bennett^1^; Yimei Lin^1^; Mark Peterson^3^; Kenneth Simpson^4^; John Loftus^5^, PhD, DVM, DACVIM (SAIM & Nutrition)

#### ^1^Cornell University, Ithaca, NY, USA; ^2^Clinical Trials Coordinator, Clinical Sciences, Cornell University, Ithaca, NY, USA; ^3^Animal Endocrine Clinic, New York, NY, USA; ^4^Professor, Clinical Sciences, Cornell University, Ithaca, NY, USA; ^5^Assistant Professor, Clinical Sciences, Cornell University, Ithaca, NY, USA

**Background:** Hyperthyroidism is the most common feline endocrinopathy. In hyperthyroid humans, untargeted metabolomic analysis identified persistent metabolic derangements despite achieving a euthyroid state. Metabolomic data for hyperthyroid cats is lacking.

**Hypothesis/Objectives:** To identify metabolomic signatures in hyperthyroid cats pre‐ and post‐I131 therapy and identify persistent metabolic changes.

**Animals:** Privately‐owned hyperthyroid cats (*n =* 7) admitted for I‐131 treatment and euthyroid privately‐owned control (CON) cats (*n =* 12).

**Methods:** Prospective case‐control study. Serum samples were collected before (T0), 1‐month (T1), and three months after (T3) I‐131 therapy. Untargeted metabolomic analysis was performed, and data were analyzed with MetaboAnalyst software (Figure 1).

**Results:** Hyperthyroid cats (T0) had a distinct metabolic signature with 277 significantly different than controls (70 increased, 207 decreased). After treatment, 66 (T1 vs. CON) and 64 (T3 vs. CON) metabolite differences persisted. Clustering and data reduction analysis (Figure 2A and B) revealed separate clustering of hyperthyroid (T0) and CON cats with intermediate phenotypes after treatment (T1 and T3). Random forest analysis disclosed thyroxine as the best model discriminator (Figure 2C), with the lipids 1‐pentadecanoyl‐GPC and mevalonate following. Median serotonin levels were higher in hyperthyroid cats (Figure 2D). Carnitine and alpha‐tocopherol levels were lower (T0 vs. CON), persisting after I‐131 treatment (Figure 2D).

**Conclusions and Clinical Importance:** Metabolic derangements do not completely resolve after achieving a euthyroid state after treating hyperthyroid cats with I‐131. Further investigation is warranted to determine if the persistent metabolic alterations demonstrated here could be a therapeutic target in treated hyperthyroid cats.

**Figure:**
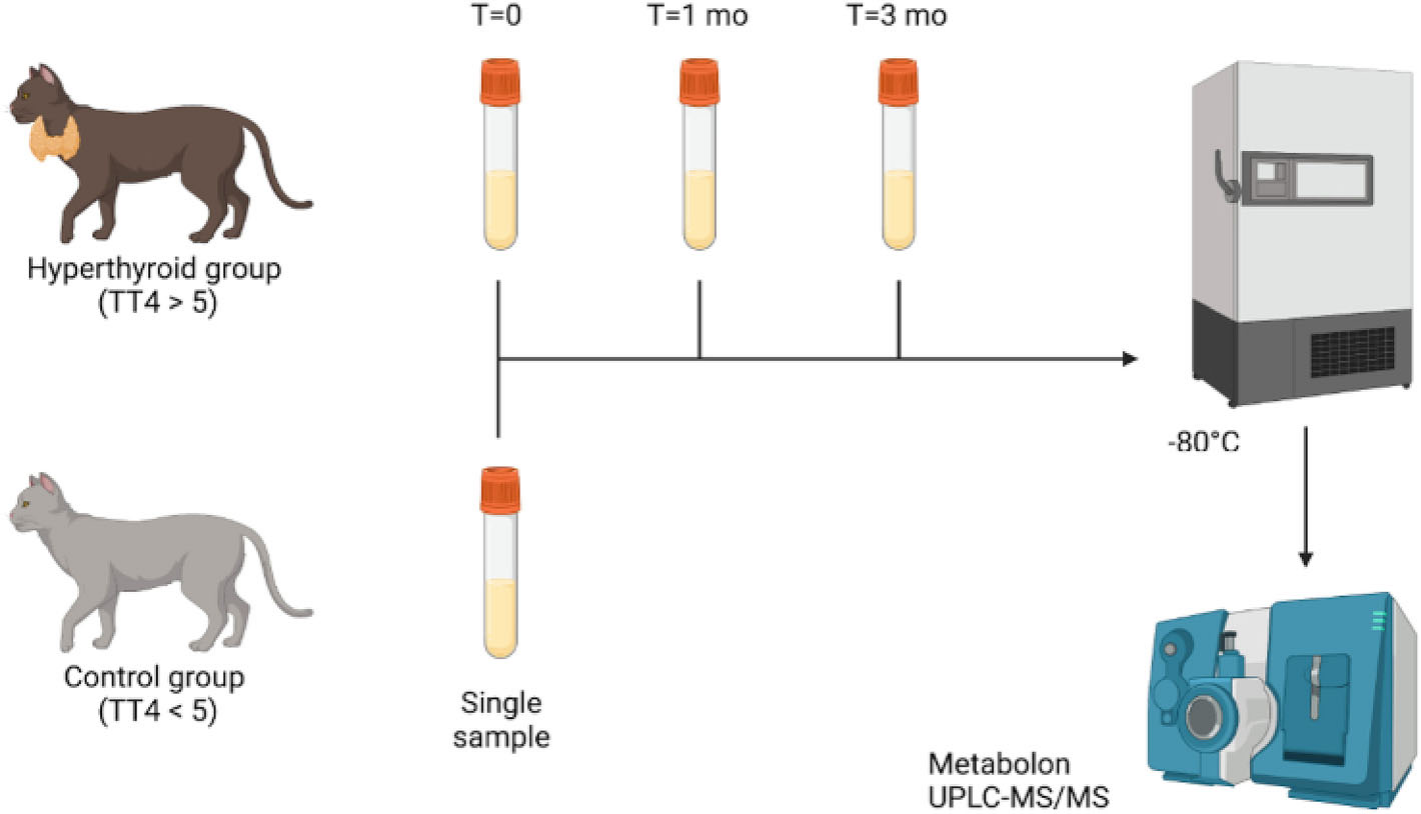
Figure 1. Methodology overview. Serum was collected from hyperthyroid cats before (*T* = 0) and after (at *T* = 1 mo and *T* = 3 mo) I131 treatment. A single sample was collected from control cats. Samples were stored for subsequent untargeted metabolic profiling and analysis.

**Figure:**
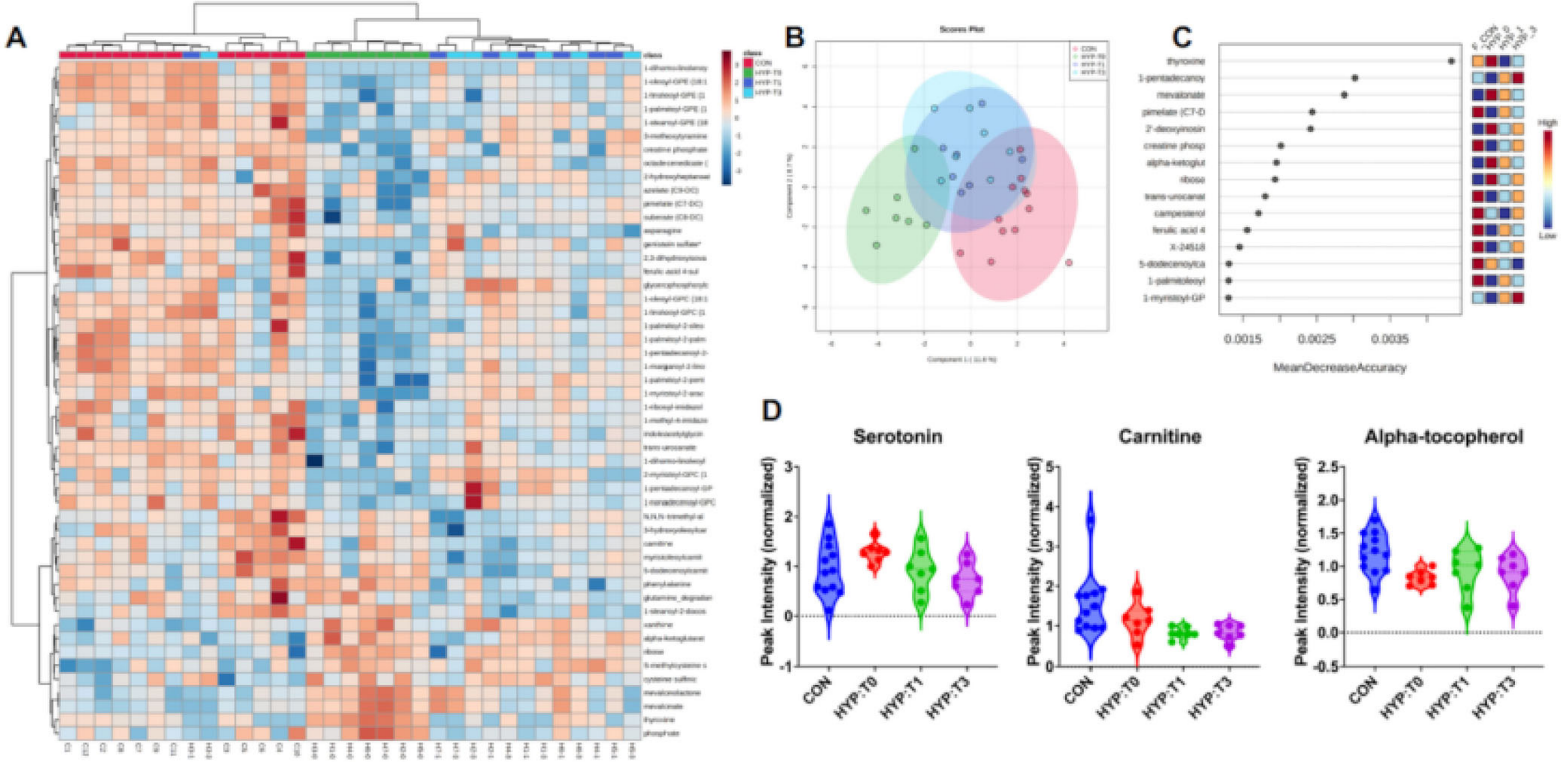
Figure 2. Metabolic analysis results. Cluster (A), data‐reduction by sPLS‐DA (B), random forest (C) analysis, and selected clinically notable metabolites (D) results of hyperthyroid cats before (HYP‐T0), one‐month (HYP‐T0), and three‐months (HYP‐T3) after I131 treatment compared to healthy control (CON) cats.

## Abstract EN11: Analytic Performance of a Veterinary‐Specific Biosensor‐Based Assay for Canine Serum Cortisol Measurement

### **Cody Brady** ^1^; Shelly Olin^2^, DVM, DACVIM (SAIM); Luca Giori^3^, DVM, PhD; Jacqueline Whittemore^4^, DACVIM (SAIM)

#### ^1^College of Veterinary Medicine, University of Tennessee, Knoxville, TN, USA; ^2^Associate Professor, Small Animal Clinical Sciences, College of Veterinary Medicine, University of Tennessee, Knoxville, TN, USA; ^3^Associate Professor, Biomedical and Diagnostic Sciences, College of Veterinary Medicine, University of Tennessee, Knoxville, TN, USA; ^4^Animal Emergency and Specialty Center of Knoxville, Knoxville, TN, USA

**Background:** Point‐of‐care, cortisol quantitation can facilitate case management, but independent analytic performance verification is warranted prior to widespread use.

**Hypothesis/Objectives:** To evaluate the precision of a point‐of‐care cortisol assay (TCA; Truforma cortisol assay, Zomedica) for canine serum cortisol quantitation, as well as agreement and bias compared to a validated chemiluminescence assay (CLA; Immulite 2000XPi, Siemens Healthcare Diagnostics Products).

**Animals:** Residual frozen (−20°C) serum from client‐owned dogs (*n =* 92) submitted for cortisol quantitation.

**Methods:** Precision of the TCA was assessed using pooled serum samples with low (0.5–2 μg/dL), medium (5–10 μg/dL), and high (18–30 μg/dL) cortisol concentrations. For method comparison, 20 samples each with low (0.5–6 μg/dL), medium (7–17 μg/dL), high (18–30 μg/dL) recorded cortisol concentrations were used. Samples were analyzed in duplicate by CLA and TCA. Spearman rank correlation, Bland‐Altman analysis, Passing‐Bablok regression, and a clinical decision error grid were used to compare results.

**Results:** Within‐laboratory coefficients of variation for pooled low, medium, and high cortisol concentrations were 15.3%, 12.2%, and 11.3%, respectively. Correlation between TCA and CLA was strong (rho = 0.92) for the method comparison sample set. Constant bias (y‐intercept = −0.043, 95% CI −0.070 to 0.069) was not identified but Passing‐Bablok analysis revealed proportional bias (slope = 1.26, 95% CI 1.11–1.39). Disagreement sufficient to cause misdiagnosis occurred for 6 and 5 samples with low and high cortisol concentrations, respectively.

**Conclusions and Clinical Importance:** Overall, the TCA and CLA assays had strong agreement. Coefficients of variation were above recommended standards for low concentrations, which could have contributed to clinical disagreements in that sample subset.

**Figure:**
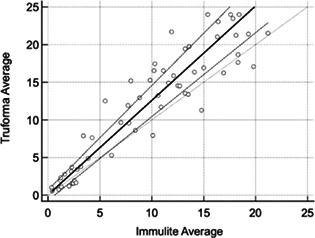
Figure 1. Passing‐Bablok regression comparing results of Immulite (CLA) and Truforma (TCA) for measuring cortisol concentrations (μg/dL) in 60 serum samples from dogs. The solid line represents the linear regression line, the dashed lines indicate the 95% CI for the regression line, and the dotted line (light grey) represents perfect agreement between methods (i.e., a regression line slope of 1).

## Abstract EN12: New Generation Device for Blood Glucose Monitoring in Cats

### **Samuel D. Stewart** ^1^; Alex Walker^2^, DVM

#### ^1^Ethos Veterinary Health; ^2^Small Animal Surgery Resident, Surgery, Ethos Veterinary Health, Woburn, MA, USA

**Background:** Accurate methods for blood glucose measurement are imperative to directing therapy in patients with dysglycemic conditions. AlphaTRAK 2 (AT2) Blood Glucose Monitoring System is currently available for glucose measurement in dogs and cats. A newer generation device, AlphaTrak 3 (AT3), has been developed to continue to provide accurate blood glucose monitoring.

**Objectives:** Demonstrate clinical accuracy of AT3 in a clinical cohort of cats compared to a reference method.

**Animals:** 62 client‐owned cats with normoglycemia and dysglycemia.

**Methods:** 3 mL blood was collected from each cat. Whole blood aliquots were immediately tested concurrently on three AT3 devices using three lots of test strips. Plasma glucose was also measured in triplicate by Beckman Coulter Chemistry Analyzer. Success was defined as 95% of measured values falling within the International Organization for Standardization (ISO) accuracy threshold and 99% of measurements falling within zones A and B of the Consensus Error Grid (CEG). A linear regression model was also used to demonstrate correlation.

**Results:** 179 of 186 glucose measurements (96.2%) fell within the ISO accuracy threshold. 100% of measurements fell within CEG zones A and B. The linear regression analysis correlation coefficient (*R* ^2^) of 0.99 indicated good correlation across the full dynamic range of glucose concentrations (20–750 mg/dL).

**Conclusions and Clinical Importance:** The analytical performance of AlphaTrak 3 was comparable to the reference analyzer over a range of feline blood glucose measurements, confirming that AlphaTrak 3 met the ISO success criteria and can be utilized for clinical decision making in diabetic cats.

## Abstract EN13: Likelihood Assessment of Hypothyroidism in Dogs Treated for Hypothyroidism at Primary Care Practices

### **Victoria BS Travail** ^1^; Florence Juvet^2^, DVM DECVIM‐CA MRCVS; Darren Kelly^3^, MVB DECVIM‐CA MRCVS; Valerie Lamb^3^, BVM&S DECVIM‐CA MRCVS

#### ^1^Southern Counties Veterinary Specialists, Hangersley, Ringwood, UK; ^2^Head of Internal Medicine, Southern Counties Veterinary Specialists, Hangersley, Ringwood, UK; ^3^Internal Medicine Clinician, Internal Medicine, Southern Counties Veterinary Specialists, Hangersley, Ringwood, UK

**Background:** There is a significant possibility that an incorrect diagnosis of hypothyroidism could be made in euthyroid patients, and the prevalence of hypothyroidism in the dog population remains controversial.

**Objectives:** To retrospectively assess the percentage of dogs diagnosed with, and treated for, hypothyroidism at first opinion practice which are likely to be truly hypothyroid and require levothyroxine supplementation.

**Animals:** 102 dogs were included in this study.

**material and methods:** The computerized databases of seven first opinion practices were searched, to identify dogs treated with levothyroxine supplementation. Three ECVIM‐CA diplomates independently assigned one of four clinical assessments to each case as follows: confirmed or very likely hypothyroid, hypothyroidism suspected but not confirmed, hypothyroidism considered unlikely, and no reason to suspect hypothyroidism. They commented as to whether or not they thought levothyroxine supplementation was appropriate.

**Results:** The measure of serum total T4 concentration was not specifically requested by the veterinarian in 30 cases (29.4%). The clinical assessments of “confirmed or very likely hypothyroid;” “Hypothyroidism suspected but not confirmed;” “Hypothyroidism considered unlikely;” and “No reason to suspect hypothyroidism” was assigned respectively by clinician 1 to 38.2%, 5.9%, 3.9%, and 52% of cases, by clinician 2 to 48%, 22.55%, 22.55%, 6.9% of cases, and by clinician 3 to 55.9%, 11.8%, 13.7%, and 18.6%. Clinician 1, clinician 2, and clinician 3 considered levothyroxine supplementation not indicated in 56.9%, 53.9%, and 45.1% of cases, respectively.

**Conclusion:** These results support the concern that hypothyroidism might be overly and incorrectly diagnosed in first opinion practice.

## Abstract EN14: Concurrent Disorders of Cats with Diabetes Mellitus and Arterial Systolic Hypertension

### **Jonathon G. Williams** ^1^; Rebecca Hess^2^, B.Sc., DVM, MSCE, DACVIM (SAIM)

#### ^1^School of Veterinary Medicine, University of Pennsylvania, Philadelphia, PA, USA; ^2^Chief, Section of Medicine, Professor, Internal Medicine, School of Veterinary Medicine, University of Pennsylvania, Philadelphia, PA, USA

**Background:** The prevalence of arterial systolic hypertension (SH) in cats with diabetes mellitus (DM) has been reported infrequently, in a small number of small studies, and ranges between zero to 37%.

**Objectives:** To determine the prevalence of SH in a large group of cats with DM and to report the clinical characteristics of cats with DM and SH.

**Animals:** 750 cats with DM including 733 cats without SH and 17 cats with SH, all examined at a university teaching hospital between January 1st, 2011, and December 31st, 2021.

**Methods:** Retrospective longitudinal study. Medical records of diabetic cats were searched for keywords “hypertension,” “blood pressure,” “amlodipine,” “benazepril,” and “telmisartan” to identify cats with (17) and without (733) SH, which was defined as systemic arterial blood pressure ≥160 mm Hg. Comorbidities including chronic kidney disease, hypertrophic cardiomyopathy, and hyperthyroidism were recorded. Hospital period prevalence was calculated by dividing the number of cats with DM and SH by the total number of cats with DM.

**Results:** The hospital period prevalence of SH among cats with DM was 17/750 or 2.3%. Most cats (15/17, 88%) with DM and SH had at least one other concurrent illness including chronic kidney disease (12/17, 71% of cats), hypertrophic cardiomyopathy (7/17, 41%), and hyperthyroidism (4/17, 23%).

**Conclusions and Clinical Importance:** The prevalence of SH among cats with DM is low. Cats with DM and SH should be evaluated for the presence of concurrent illness.

## Abstract EN15: Adrenal Incidentaloma: Epidemiology, Clinicopathological Evaluation, Vascular Invasion Pattern, and Histopathological Results

### **Shino Yoshida** ^1^, DVM; Noriyuki Nagata^2^, DVM; Kenji Hosoya^3^, DVM, PhD, DACVR (Radiation Oncology), DACVIM (Oncology), JCVS; Kensuke Nakamura, DVM, PhD;^6^ Noboru Sasaki, DVM;^7^ Sangho Kim^4^, DVM, PhD; Nosumu Yokoyama, DVM;^7^ Ryohei Kinoshita^2^, DVM; Kyosuke Takeuchi^2^, DVM; Genya Shimbo^2^, DVM; Yoshihisa Yamane^1^; Mitsuyoshi Takiguchi^5^, DVM, PhD

#### ^1^Hokkaido University Veterinary Teaching Hospital, Sapporo, Hokkaido, Japan; ^2^Specially Appointed Assistant Professor, Hokkaido University Veterinary Teaching Hospital, Sapporo, Hokkaido, Japan; ^3^Associate Professor, The Laboratory of Advanced Veterinary Medicine, Hokkaido University, Sapporo, Hokkaido, Japan; ^4^Assistant Professor, The Laboratory of Veterinary Surgery, Hokkaido University, Sapporo, Hokkaido, Japan; ^5^Professor, The Laboratory of Veterinary Internal Medicine, Hokkaido University, Sapporo, Hokkaido, Japan; ^6^Associate Professor, Hokkaido University Veterinary Teaching Hospital, Sapporo, Hokkaido, Japan; ^7^Assistant Professor, Hokkaido University Veterinary Teaching Hospital, Sapporo, Hokkaido, Japan

**Background:** Adrenal incidentalomas (AIs) are defined as the unexpectedly found mass in the adrenal gland. Thanks to the development of imaging modalities such as ultrasonography and computed tomography, the chance to detect AIs is increasing. However, information about the characteristics of AIs and approaches to AIs is lacking.

**Objectives:** This study aims to describe; endocrinological characteristics and the final diagnosis of AIs.

**Animals:** Dogs that were found AIs and diagnosed via histopathology or endocrinological examinations were included.

**Methods:** Electronical records from 2019–2022 in the Hokkaido university veterinary teaching hospital were retrospectively reviewed.

**Results:** A total of 46 dogs were included in this study. 28 dogs were surgically treated and diagnosed via histopathology (Sx group); pheochromocytoma (*n =* 14), adenocarcinoma (*n =* 6), adenoma (*n =* 6), sarcoma (*n =* 1), no abnormality (*n =* 1). 18 dogs were diagnosed via clinicopathological examination such as dexamethasone suppression test, urinary cortisol creatinine ratio, and urinary normetanephrine/metanephrine‐to‐creatinine ratio (non‐Sx group); pheochromocytoma suspected (*n =* 7); non‐functional AIs suspected (*n =* 7), hyperadrenocorticism suspected (*n =* 7). In the Sx group, 14 dogs had a vascular invasion and 12 of these dogs were diagnosed as having pheochromocytoma. The remaining two dogs had bilateral AIs. In the non‐Sx group, the vascular invasion was only detected in the pheochromocytoma suspected group.

**Conclusion:** Most AIs were diagnosed as pheochromocytoma and these dogs tend to have a vascular invasion, although they were incidental findings. Therefore, even if they are incidental findings, AIs should be carefully investigated. In addition, the urinary normetanephrine/metanephrine‐to‐creatinine ratio should be measured.

## Abstract EN16: Whole Transcriptome Analysis of Canine Pheochromocytoma and Paraganglioma (ESVE Award Winner)

### **Marit F. van den Berg** ^1^; Hans Kooistra^2^, DVM, PhD, DECVIM‐CA (IM); Guy Grinwis^3^, DVM, PhD; Monique van Wolferen^4^, Ing; Elpetra Timmermans‐Sprang^4^, PhD; Maurice Zandvliet^5^, DVM, PhD, DECVIM‐CA (Oncology); Frank van Steenbeek^5^, PhD; Sara Galac^5^, DVM, PhD

#### ^1^Utrecht University, Utrecht, The Netherlands; Professor, ^2^Department of Clinical Sciences, Utrecht University, Utrecht, The Netherlands; ^3^Assistant Professor, Department of Biomolecular Health Sciences, Utrecht University, Utrecht, The Netherlands; ^4^Research and Education Assistant, Department of Clinical Sciences, Utrecht University, Utrecht, The Netherlands; ^5^Assistant Professor, Department of Clinical Sciences, Utrecht University, Utrecht, The Netherlands

**Background:** Given the current lack of effective medical treatment for pheochromocytomas and paragangliomas (PPGLs), there is a need for novel therapeutic strategies.

**Hypothesis/Objectives:** To identify druggable pathways driving PPGL development, RNA sequencing was performed on PPGLs and normal adrenal medullas (NAMs) of dogs.

**Animals:** 10 healthy dogs, 19 PPGL dogs.

**Methods:** Tissue was snap‐frozen in liquid nitrogen or fixed in RNA *later* stabilization solution and stored at −70°C until RNA extraction. RNA sequencing was performed. The Mann–Whitney *U*‐test was used to calculate *P* values, which were corrected for multiple comparisons using the false discovery rate method. Genes were considered differentially expressed when the corrected *P* value was <0.05 and the log_2_ fold change was >3 or <−3.

**Results:** Principal component analysis revealed that PPGLs clearly clustered apart from NAMs. Between PPGLs and NAMS, 232 genes were differentially expressed. Compared with NAMs, PPGLs had increased expression of genes related to the cell cycle (e.g., *PCLAF*, *CCNB2*, *CDC6*, *TOP2A*), tumor development, progression and metastasis (e.g., *POSTN*, *FOXI3*, *MYBL2*), hypoxia and angiogenesis (e.g., *CDCP1*, *EDN2*, *ESM1*), and the Wnt signaling pathway (e.g., *WNT3*, *SFRP2*), and decreased expression of genes related to adrenal steroidogenesis (e.g., *SF‐1*, *SOAT1*, *CYP11A1*). Our data revealed several overexpressed genes that could provide targets for novel therapeutics, such as *RET*, *DRD2*, and *SFRP2*.

**Conclusions and Clinical Importance:** This study has shed light on the transcriptomic profile of canine PPGL, which will be instrumental in understanding the pathogenesis of PPGL in dogs, and revealed novel targets for therapy.

## Abstract EN17: The Role of Gastrin in Dogs with Spontaneous Primary Hyperparathyroidism

### **Julieann Vose** ^1^; Jean Brudvig^2^, DVM, MPH, PhD, DACVP; Enass Bassiouny^3^; Ashley LaClair^4^; Brian Petroff^5^; Jared Jaffey^6^, DVM, MS, DACVIM; Harry Cridge^7^

#### ^1^College of Veterinary Medicine, Michigan State University, East Lansing, MI, USA; ^2^Academic Specialist, Endocrinology, Veterinary Diagnostic Laboratory, Michigan State University, Lansing, MI, USA; ^3^Laboratory Technologist Supervisor, Veterinary Diagnostic Laboratory, Michigan State University, Lansing, MI, USA; ^4^Laboratory Technologist, Veterinary Diagnostic Laboratory, Michigan State University, Lansing, MI, USA; ^5^Section Chief, Endocrinology, Veterinary Diagnostic Laboratory, Michigan State University, Lansing, MI, USA; ^6^Assistant Professor, College of Veterinary Medicine, Midwestern University, Glendale, AZ, USA; ^7^Assistant Professor, Small Animal Clinical Sciences, College of Veterinary Medicine, Michigan State University, East Lansing, MI, USA

**Background:** Hypercalcemia has been associated with hypergastrinemia in humans. Hypergastrinemia could be responsible for gastrointestinal signs in dogs with primary hyperparathyroidism (PHPT).

**Hypothesis/Objectives:** To i) determine the prevalence of hypergastrinemia in dogs with PHPT, ii) assess correlation between ionized calcium (iCa) and serum gastrin concentrations, iii) determine whether gastrin concentrations decrease following treatment of PHPT.

**Animals:** 144 dogs with spontaneous PHPT.

**Methods:** Prospective study. Diagnosis of PHPT was based on medical record review. Dogs with azotemia, concurrent disease, or those receiving acid suppressants were excluded. Gastrin concentrations were quantified via radioimmunoassay. Eleven dogs had repeat quantification of gastrin, PTH, and iCa, 4 weeks after parathyroidectomy or ethanol ablation. Spearman's rank correlation coefficients (rs) were used to evaluate association between iCa and gastrin. A paired t‐test was used to assess changes in gastrin, PTH, and iCa, concentrations from baseline to 28 days post treatment.

**Results:** Increased gastrin concentrations (>78.9 ng/L) were found in 19.7% of dogs at PHPT diagnosis, compared to 9.1% post treatment. The median gastrin concentrations pre vs. post treatment were 49.0 ng/L (IQR: 29.5 ng/L) and 64.0 ng/L (IQR: 31.0 ng/L) respectively. PHPT treatment did not significantly alter gastrin concentrations (*P* = .60) but did reduce iCa (*P* = <.001) and PTH (*P* = .01) concentrations. Serum gastrin and iCa concentrations were not correlated (*P* = .12).

**Conclusions and Clinical Importance:** Mild elevations in serum gastrin concentration may be seen in dogs with PHPT. Gastrin is not correlated with iCa concentration and does not decrease following PHPT treatment.

## Abstract EN18: Effects of Glucagon Infusion on the Canine Plasma Metabolome

### **Michael Merkhassine** ^1^; Carol Frederick^2^; Lucinda Bennett^2^, LVT; Benjamin Morse^1^; Reilly Coch^3^, MD; Bethany Cummings^4^; John Loftus^5^, PhD, DVM, DACVIM (SAIM & Nutrition)

#### ^1^Cornell University, Ithaca, NY, USA; ^2^Clinical Trials Coordinator, Clinical Sciences, Cornell University, Ithaca, NY, USA; ^3^Adjunct Professor, Clinical Sciences, Cornell University, Ithaca, NY, USA; ^4^University of California–Davis, Davis, CA, USA; ^5^Assistant Professor, Clinical Sciences, Cornell University, Ithaca, NY, USA

**Background:** Glucagon helps maintain glucose and amino acid homeostasis but may be dysregulated in disease states. Increased plasma glucagon reduces plasma amino acids, may increase urinary amino acid clearance, acts as an insulin secretagogue, and modulates cellular metabolism.

**Hypothesis/Objectives:** Exogenous glucagon significantly alters the canine plasma metabolome.

**Animals:** Five healthy male purpose‐bred research beagles.

**Methods:** Two 6‐h glucagon infusions were performed, a low‐dose CRI (3 ng/kg/min; CRI‐LO) and a high‐dose CRI (50 ng/kg/min; CRI‐HI), with a 7‐day washout in between. A continuous flash glucose system monitored interstitial glucose. Untargeted liquid chromatography‐mass spectrometry (LC‐MS) was performed on plasma samples collected before and after infusions. Data were analyzed with MetaboAnalyst.

**Results:** A transient increase (peak 90–120 min) in glucose occurred during the CRI‐HI but not CRI‐LO. Glucose levels returned to near baseline by the end of the 6‐hour infusion. The CRI‐LO significantly changed 372 plasma metabolites, with 333 metabolites reduced. The CRI‐HI significantly affected 414 metabolites, with 369 reduced. Generally, changes were in the same direction under both conditions and were reflected by distinct clustering of post‐CRI metabolites (Figure 1) by data‐reduction (sPLS‐DA). The CRI‐HI broadly reduced circulating amino acid levels, especially arginine and proline. Other affected pathways included purine, pyrimidine, arachidonic acid, and primary bile acid biosynthesis (Table 1).

**Conclusions and Clinical Importance:** Exogenous glucagon significantly alters the canine plasma metabolome independently of glucose levels. These data will aid our understanding of glucagon physiology and the pathophysiology of canine disorders involving glucagon.

**Figure:**
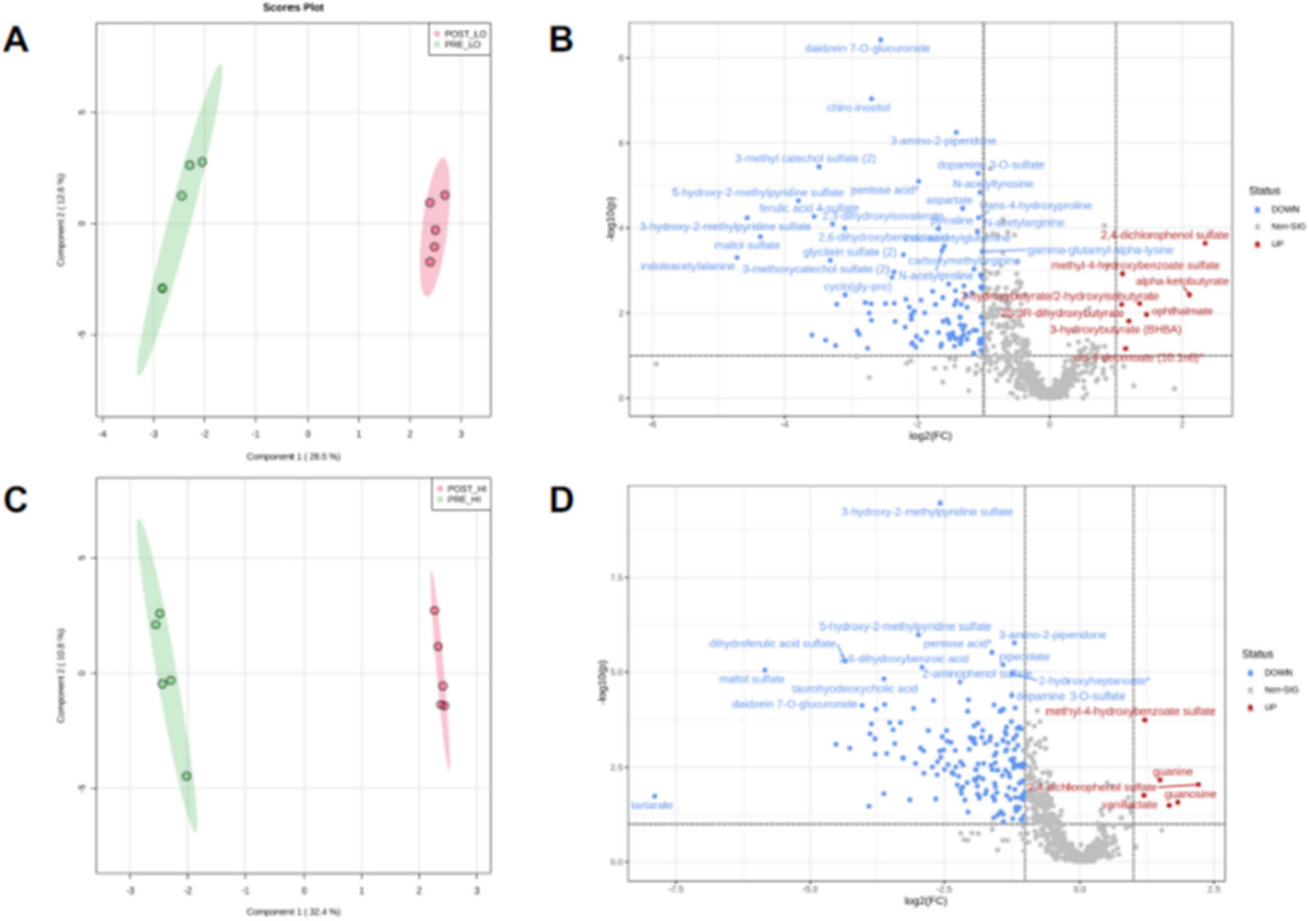
Figure 1. Metabolomic analysis results. Data‐reduction by sPLS‐DA (A&C) and volcano plots (B&D) constructed from metabolomic results of the low‐dose (A&B) and high‐dose (C&D) glucagon infusions.

**Figure:**
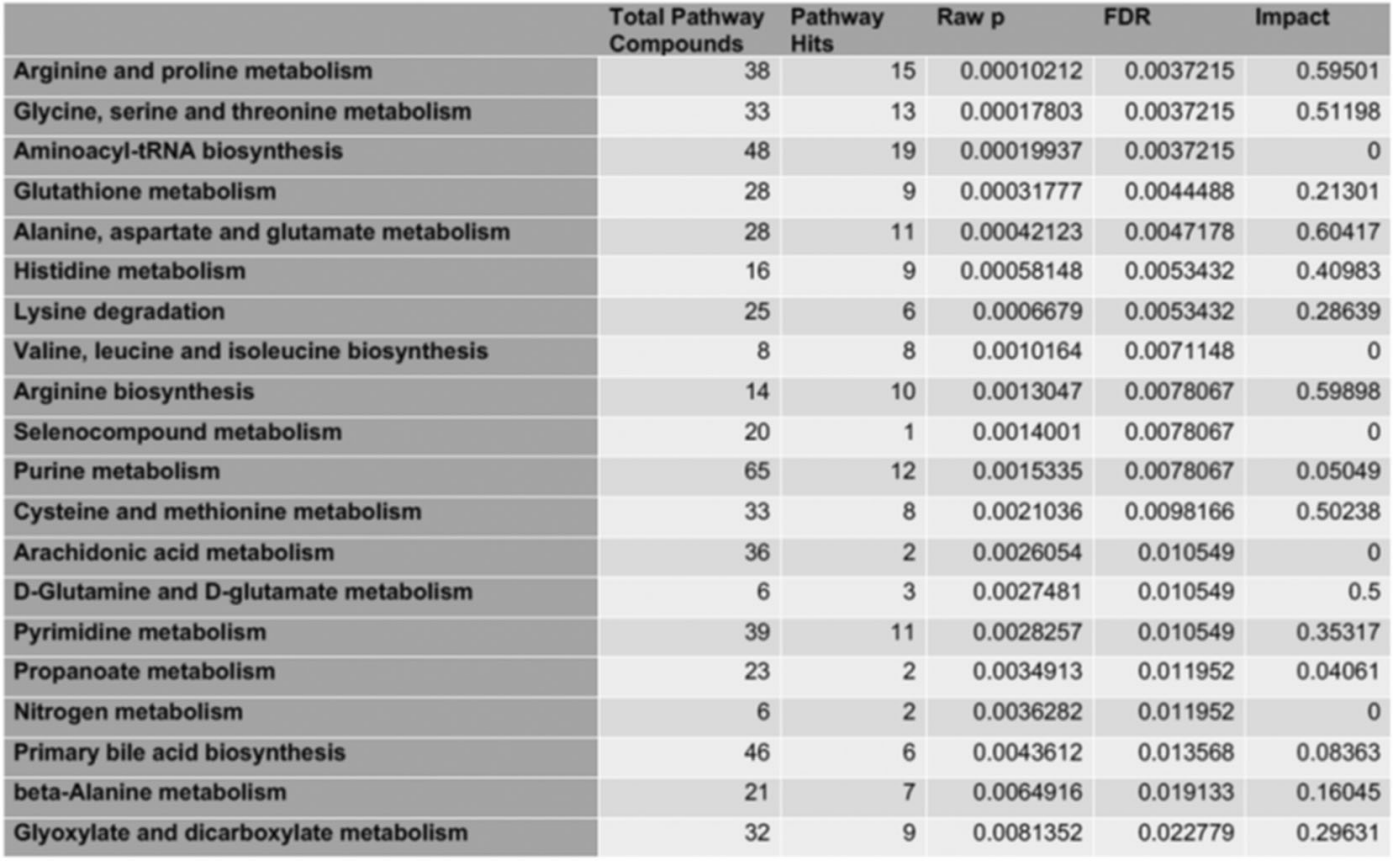
Table 1. Pathway analysis of high‐dose glucagon metabolomic results

## Abstract EN19: Sialocele and Their Association With Hyperadrenocorticism and Long‐term Glucocorticoid Treatment in Dogs: Retrospective Case‐Control Study

### **JeongYeol Bae**; Guk‐Il Joung, DVM; Jin‐Young Kim, DVM; Jung‐Il Kim, DVM; Joong‐Hyun Song, DVM, PhD, Professor

#### Internal Medicine, Veterinary Medical Teaching Hospital, Chungnam National University, Korea

**Background:** Dogs with sialocele often have concurrent hyperadrenocorticism (HAC) or are receiving long‐term glucocorticoid (GC). Their association has not been investigated, and previous human studies have suggested that GC may affect the salivary glands.

**Hypothesis/Objectives:** This retrospective matched case‐control study aimed to investigate the association between HAC, long‐term GC treatment, and diagnosis of sialocele in dogs.

**Animals:** Fifty‐seven client‐owned dogs were enrolled. 19 dogs diagnosed with sialocele, 38 dogs without sialocele.

**Methods:** This study constitutes a retrospective review of the records from 2018 to 2022. Records of 19 dogs diagnosed with sialocele were investigated for the presence or absence of HAC and long‐term GC treatment. Two age‐ and breed‐matched controls for each sialocele dog (38 dogs) were equally investigated for the same concurrent diseases. Logistic regression analysis was used.

**Results:** The odds of sialocele in dogs with HAC were 16.21 times that of dogs without HAC (*P* = .022; 95% CI: 1.5–175.2). And the odds of sialocele in dogs with long‐term GC treatment (median, 8 months; range, 5–13) were 7.71 times that of dogs without long‐term GC treatment (*P* = .034; 95% CI: 1.16–50.94). No associations were found between age, sex, body weight, and the presence of a sialocele.

**Conclusions and Clinical Importance:** Sialocele was significantly associated with HAC and long‐term GC treatment in dogs. Hyperadrenocorticoid dogs and dogs with long‐term GC administration should consider the possible presence of a sialocele. Dogs diagnosed with sialocele should be identified for concurrent HAC if clinically suspected, as well as prolonged GC exposure.

**Table:** Table 1. Signalment of dogs with sialocele and control dogs (age‐ and breed‐matched) without sialocele

	Dogs with sialocele (*n =* 19)	Control dogs (*n =* 38)
Age in years—median (range)	11 (3–17)	11 (3–17)
Bodyweight in kilograms (range)	3.8 (2–17)	5.1 (2.4–28.1)
**Sex**		
Female	11 (58%)	23 (61%)
Male	8 (42%)	15 (39%)
**Breed**		
Maltese	7 (37%)	14 (37%)
Pomeranian	4 (21%)	8 (21%)
Poodle	2 (11%)	4 (11%)
Mixed	2 (11%)	4 (11%)
Jindo	2 (11%)	4 (11%)
French bulldog	1 (5%)	2 (5%)
Boston terrier	1 (5%)	2 (5%)

**Table:** Table 2. Odds ratio and *P* value (and 95% confidence intervals, CI) from logistic regression analysis identifying associations between HAC, long‐term GC treatment, and diagnosis of sialocele in dogs

Variables	OR	*P* value	95% CI
Age	1.007	0.928	0.858–1.183
Sex	1.120	0.862	0.313–4.008
Bodyweight	1.047	0.373	0.946–1.160
HAC	16.210	0.022*	1.5–175.2
Long‐term GC administration	7.713	0.034*	1.16–50.94

## Abstract EN20: A Retrospective Study of Neutrophil‐to‐Lymphocyte and Platelet‐to‐Lymphocyte Ratios as Potential Markers in Dogs with Hypercortisolism

### **Sijin Cha** ^1^; Sumin Yun^1^; Taesik Yun^1^, PhD; Jimin Oh^1^; Dohee Lee^1^; Yoonhoi Koo^1^; Yeon Chae^1^; Hakhyun Kim^2^, PhD; Byeong‐Teck Kang^2^, PhD; Mhan‐Pyo Yang^2^, PhD

#### ^1^Laboratory of Veterinary Internal Medicine, College of Veterinary Medicine, Chungbuk National University, Cheongju, South Korea; ^2^Professor, Internal Medicine, Laboratory of Veterinary Internal Medicine, College of Veterinary Medicine, Chungbuk National University, Cheongju, South Korea

**Background:** The neutrophil‐to‐lymphocyte ratio (NLR) and platelet‐to‐lymphocyte ratio (PLR) have been considered inflammatory biomarkers in several diseases. Although changes in NLR and PLR have been identified in humans with Cushing syndrome (CS), changes in these inflammatory biomarkers have not been fully understood in dogs with hypercortisolism (HC).

**Objective:** To evaluate whether NLR and PLR could be used as biomarkers for the diagnosis and monitoring of treatment response in dogs with HC.

**Animals:** 67 dogs with HC, 58 dogs with non‐adrenal illness (NAI), and 39 healthy dogs.

**Methods:** Retrospective study. NLR and PLR were compared among the three groups. In addition, the NLR and PLR were compared before and after trilostane treatment.

**Results:** NLR and PLR were significantly higher in the HC group than in the NAI and healthy groups. The NLR cut‐off value of 4.227 had a sensitivity of 67.16% and specificity of 65.52%, and the PLR cut‐off value of 285 had a sensitivity of 56.72% and specificity of 70.69% for differentiating between dogs with HC and those with NAI, respectively. Furthermore, a significant decline in NLR was observed after treatment in the well‐controlled HC group. The cutoff value of percent change in NLR to identify well‐controlled HC was −7.570%; sensitivity and specificity were 100% and 63.64%, respectively.

**Conclusion and Clinical Importance:** NLR and PLR might be used cautiously as supportive biomarkers for HC diagnosis, and NLR could be a potential monitoring tool in assessing the treatment response of HC in dogs.

## Abstract EN21: Relationship between Serum Concentration of Canine Pancreatic Lipase Immunoreactivity and Hyperglycemia in Diabetic Dogs

### **Yeon Chae**; Junseok Kim; Taesik Yun; Yoonhoi Koo; Dohee Lee; Mhan‐Pyo Yang; Byeong‐Teck Kang; Hakhyun Kim

#### Chungbuk National University, Cheongju, South Korea

**Background:** Hypertriglyceridemia can partially mediate between diabetes mellitus (DM) and pancreatitis in dogs, implying that another mediator, such as chronic hyperglycemia, might exist.

**Objectives:** To evaluate the relationship between hyperglycemia and serum canine pancreatic lipase immunoreactivity (cPLI) concentration in diabetic dogs.

**Animals:** Twenty‐six client‐owned dogs with DM were included, and the DM dogs were divided into two subgroups according to serum fructosamine level (<500 μmol/l = good, controlled DM group; >500 μmol/l = poorly controlled DM group).

**Methods:** Retrospective cohort study. Serum fructosamine and cPLI concentration were measured in all the dogs.

**Results:** Five of 26 dogs with DM (19.2%) had high serum cPLI concentration consistent with pancreatitis, and two of them also showed ultrasonographic evidence of pancreatitis without clinical signs. The median serum cPLI concentrations were significantly higher in poorly controlled DM group (median: 520 μg/L, range: 179.76–1000 μg/L) than those of well controlled DM group (median: 77 μg/L, range: 32.22–244.6 μg/L, *P* = 0.0147). Serum fructosamine concentration was positively correlated with serum cPLI concentration (*r =* 0.4816; *P* = 0.0127). Multivariate analysis showed that serum triglyceride and fructosamine concentration were associated with the serum cPLI concentration.

**Conclusions and Clinical Importance:** Chronic hyperglycemia may induce pancreatic inflammation in diabetic dogs; however, the clinical significance of increased cPLI concentration is unknown.

## Abstract EN22: Clinical Evaluation of a Point‐of‐Care Free Thyroxine Assay to Diagnose Canine Hypothyroidism

### **Drew J. Narwold, Jr.** ^1^; Cynthia Ward^2^, VMD, PHD, DACVIM (SAIM); Ashley Wood^3^, PhD

#### ^1^Zomedica, Ann Arbor, MI, USA; ^2^Professor Emerita, University of Georgia, Athens, GA, USA; ^3^Vice President, Research and Development, Zomedica Inc., Ann Arbor, MI, USA

**Background:** Hypothyroidism is commonly diagnosed in dogs. In addition to evaluating an animal's clinical signs, measurement of endogenous thyroid molecules—including total thyroxine (tT_4_), thyroid‐stimulating hormone (TSH), and free thyroxine (fT_4_)—frequently aids in identifying hypothyroid animals. In‐clinic tT_4_ and TSH tests are available but a point‐of‐care (POC) fT_4_ assay with accurate measurements is needed, especially since approximately 25% of hypothyroid dogs do not have elevated TSH levels.

**Objective:** Assess the diagnostic performance of canine POC fT_4_ measurements for identifying hypothyroidism.

**Animals:** 66 canines believed to be euthyroid (*n =* 28) or suspected of having hypothyroidism (*n =* 38) based on clinical and clinicopathologic signs from across four American veterinary clinics.

**Methods:** All animals underwent a physical exam and screening workup, including complete blood count, chemistry, urinalysis, and thyroid diagnostic profile, performed at a reference lab. In addition, serum tT_4_, TSH, and fT_4_ levels were measured in‐clinic on the TRUFORMA diagnostic platform. Blinded to TRUFORMA results, a board‐certified endocrinologist diagnosed the animals based on the reference lab results and their clinical and clinicopathologic signs.

**Results:** The clinical sensitivity of the TRUFORMA fT_4_ assay was 100% (95% CI: 46.3–100%) while the specificity was 88.5% (77.2–94.9%) when compared to the final endocrinologist diagnosis. Factoring in additional TRUFORMA results (tT_4_ and/or TSH) led to even higher clinical specificity with a maximum of 96.7% (95% CI: 87.6–99.4%) being achieved.

**Conclusion:** The TRUFORMA point‐of‐care fT_4_ assay can aid in distinguishing hypothyroid dogs from those that are euthyroid.

## Abstract EN23: What Can Be Learned from Studying Plasma Lactate in Dogs with Naturally Occurring Hypercortisolism?

### **Álan G. Pöppl** ^1^; Tais Nogueira^2^, DVM; José Lucas Lopes^2^; Frederico Carvalho Soares^3^, DVM, MSc, DSc; Daniela Lopes^4^, DVM, MSc; Denise Da Silva^5^; Bruna Machado^5^; Laura Ritzel^5^; Rodrigo Rabelo^6^, DVM, MSc, DSc; Vanessa Eder^7^, DVM; Stella Valle^8^, DVM, MSc, DSc

#### ^1^Universidade Federal do Rio Grande do Sul (UFRGS); ^2^Master's Degree Student, Veterinary Sciences Post‐Graduation Program (PPGCV), UFRGS; ^3^Frederico Soares; ^4^Brisbane Pet Surgery; ^5^Graduation Student, Faculty of Veterinary, UFRGS; ^6^CEO, Intensivet; ^7^Resident, Veterinary Clinical Pathology, UFRGS; ^8^Professor, Veterinary Clinical Pathology, UFRGS

**Background:** Hyperlactatemia in humans with Cushing's syndrome and dogs under glucocorticoid therapy are well described. The relevance of plasma lactate in dogs with naturally occurring hypercortisolism (NOHC) is unknown.

**Hypothesis/Objectives:** Assess plasma lactate as a prognostic tool in canine NOHC.

**Animals:** Twenty‐two dogs newly diagnosed with NOHC from a veterinary teaching hospital. Dogs were classified accordingly with plasma lactate at diagnosis: CG (control group, lactatemia ≤2.5 mmol/L, *n =* 7) and HLG (hyperlactatemia group, lactatemia >2.51 mmol/L, *n =* 15).

**Methods:** Cohort study. Age, weight, BCS, serum LDH activity, perfusion index, SBP, and echocardiographic measurements (LA/Ao, LVIDd, LVDWd, IVSd) were compared between groups and correlations with lactatemia studied by Pearson's correlation. Survival, ALIVE Cushing's Clinical Score (ACCS), trilostane doses prescribed, and complications/comorbidities occurrence were set as outcomes and compared between groups. Lactatemia after reaching satisfactory NOHC control was compared with initial results.

**Results:** Differences were found in plasma lactate (HLG = 4.37 ± 1.77, CG = 1.81 ± 0.49, *P* < 0.0001), ACCS at diagnosis (HLG = 10/15, range 9–13, CG = 9/15, range 3–9, *P* = 0.0013), and complications/comorbidities (*P* = 0.0085) between groups. There were five deaths in the HLG and none in the CG (*P* = 0.0956, Figure 1). There were no differences between the other parameters compared between groups, as well as lactatemia showed no significant correlation with the studied variables. Lactatemia significantly reduced after disease satisfactory control (*P* = 0.037, Figure 2).

**Conclusions and Clinical Importance:** Hyperlactatemia suggests a poorer short‐term prognosis in canine NOHC and was associated with a worsened clinical initial presentation.

**Figure:**
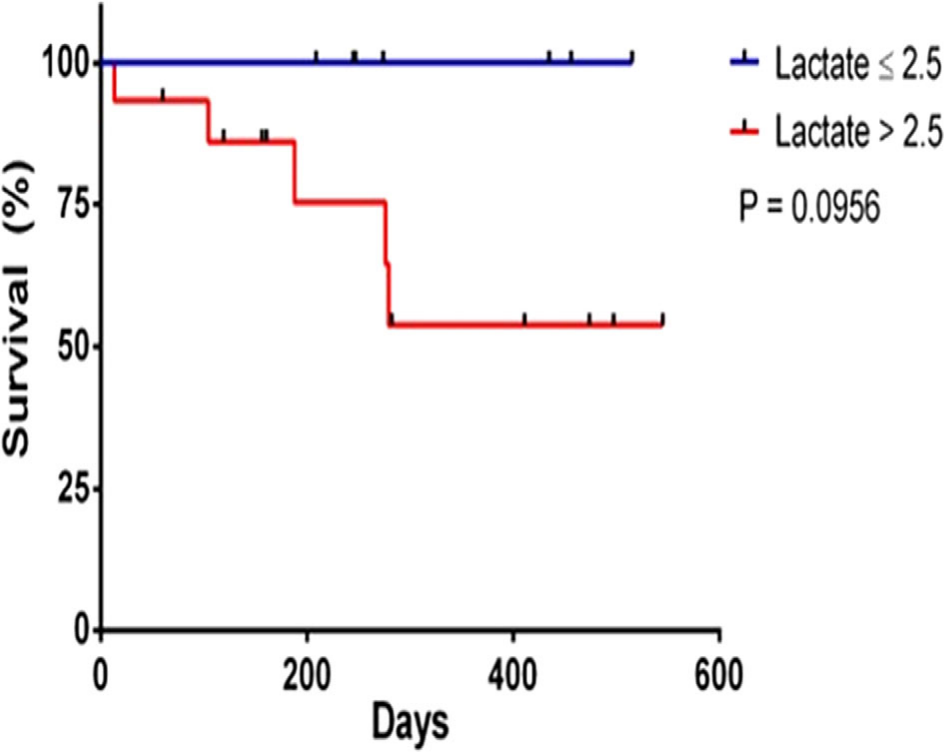
Figure 1. Kaplan‐Meier survival curve for 22 dogs with naturally occurring hypercortisolism divided accordingly with their plasma lactate at diagnosis (up to 2.5 mmol/L or greater than 2.5 mmol/L).

**Figure:**
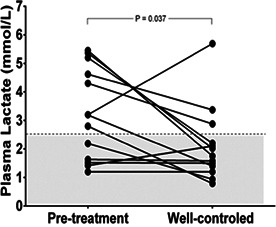
Figure 2. Individual plasma lactate from 13 dogs with naturally occurring hypercortisolism that achieved satisfactory disease control (mea*n =* 2.11 ± 1.29 mmol/L) during the study compared with their pre‐treatment results (mea*n =* 3.24 ± 1.69 mmol/L). The dotted line represents the reference range.

## Abstract EN24: Evaluation of a Recombinant Porcine Corticotropin for the ACTH Stimulation Test in Healthy Cats

### **Álan G. Pöppl** ^1^; Daniela Lopes^2^; Luciana de Jesus^3^, DVM, MSc; Lara Girotto^4^, DVM; Milena Oliveira^3^, DVM; Mirela Paim^4^, DVM, MSc; Barbara Rivas^3^, DVM, MSc; Fernanda Amorim da Costa^6^, DVM, MSc, DSc

#### ^1^Universidade Federal do Rio Grande do Sul (UFRGS); ^2^Brisbane Pet Surgery; ^3^Veterinary Sciences Post‐Graduation Program (PPGCV), UFRGS; ^3^Faculty of Veterinary, UFRGS; ^4^PhD Student, Veterinary Sciences Post‐Graduation Program (PPGCV), UFRGS; ^6^Professor, Veterinary Sciences Post‐Graduation Program (PPGCV), UFRGS

**Background:** Equivalence between 5 μg/kg and 125 μg/cat doses in the ACTH stimulation test (AST) was documented in cats regarding different synthetic corticotropins such as tetracosactide and cosyntropin.

**Hypothesis/Objectives:** Assess the dose‐equivalence for the AST between 1 or 5 μg/kg against the 125 μg/cat dose of a commercial product with recombinant porcine corticotropin (ACTHEL 25 UI).

**Animals:** Seven healthy neutered cats (four males) from a shelter.

**Methods:** Cross‐sectional study. Intravenous ACTHEL injections of 1, 5, and 125 μg/cat with a seven‐day interval for each test were applied in each cat. Blood samples were drawn before and one hour after ACTHEL for aldosterone, cortisol, and progesterone measurements by radioimmunoassay and androstenedione by chemiluminescent assay. Pre‐ and post‐ACTH results were compared by the Wilcoxon matched‐pairs test. The Δ‐hormonal for each dose tested was compared by Friedman's test followed by Dunn's test.

**Results:** Androstenedione concentration was not stimulated for any tested dose. A significant (*P* = 0.03) increase in post‐ACTH progesterone with 1 μg/kg was observed. The 5 μg/kg dose promoted increases in post‐ACTH aldosterone (*P* = 0.01) and progesterone (*P* = 0.01). The 125 μg/cat dose promoted a significant increase (*P* = 0.01) in post‐ACTH aldosterone, cortisol, and progesterone serum concentration. The 125 μg/cat dose showed higher Δ‐hormonal for aldosterone (*P* = 0.003), cortisol (0.008), and progesterone (*P* = 0.05).

**Conclusions and Clinical Importance:** The reduced doses tested were considered inappropriate to assess cortisol adrenal reserve in the AST; notwithstanding, they were capable of significantly increasing progesterone (1 and 5 μg/kg) and aldosterone (5 μg/kg) serum concentration.

## Abstract EN25: Validation of a Copeptin Assay in Normal Dogs

### **Thomas Schermerhorn** ^1^; Grace Calo^2^

#### ^1^College of Veterinary Medicine, Kansas State University, Manhattan, KS, USA; ^2^Clinical Sciences, College of Veterinary Medicine, Kansas State University, Manhattan, KS, USA

**Background:** Copeptin is a 39 aa peptide produced in the pituitary by post‐transcriptional processing of pre‐pro‐arginine vasopressin, which also produces antidiuretic hormone (ADH). Copeptin and ADH are co‐secreted in equimolar amounts into circulation, but copeptin measurement is less impacted by pre‐analytic factors than ADH assay. Copeptin is a useful surrogate for ADH secretion in numerous human disorders but its role as a biomarker for ADH release has not been investigated in dogs.

**Hypothesis/Objectives:** Validate a commercial ELISA assay for measurement of canine serum copeptin.

**Animals:** Healthy dogs participating in a voluntary blood donor program.

**Methods:** Banked serum samples from nine healthy dogs were analyzed using a commercial copeptin ELISA (Copeptin (Human) EIA Extraction Free kit, Phoenix Pharmaceutical Inc). Assays were performed according to manufacturer's instructions. Standards and canine samples were analyzed in duplicate. Standard curves were plotted using the 4‐parameter logistic (4PL) model. Validation parameters determined were intra‐ and inter‐assay coefficient of variation (CV), recovery, parallelism, and dilutional linearity.

**Results:** Serum copeptin was detected in all samples (*n =* 9). Mean serum copeptin concentration was 1.67 ng/mL (range 0.85–4.09 ng/mL). Intra‐assay CV was 8.42% and inter‐assay CV was 9.6%. The limits and range of detection were 0.1–10 ng/ml. The assay displayed dilutional linearity and parallelism. Recovery from spiked samples averaged 147%.

**Conclusion:** The assay displayed acceptable parameters for precision, linearity, and recovery. The results provide an expected range for copeptin in healthy dogs, suggesting that this copeptin assay may be a useful research tool for investigating water balance in dogs.

## Abstract EN26: Relationship Between Post ACTH Cortisol Concentration at Diagnosis and Dose of Trilostane in Small‐Breed Dogs

### **Woo‐Jin Song** ^1^; Minkun Kim^1^; Saeyoung Lee^1^; Jongjin Park^1^; Youngmin Yun^1^; Hwa‐Young Youn^2^

#### ^1^Jeju National University; ^2^Seoul National University

**Background:** Only few studies have evaluated the initiation dose and frequency of trilostane medication in small‐breed dogs. Further, little is known about the factors that may influence the dose of trilostane required for clinical improvement.

**Objectives:** This study aimed to investigate the correlation between post‐ACTH cortisol concentration at diagnosis and the optimal trilostane dose in small‐breed dogs with hyperadrenocorticism.

**Animals:** Small‐breed dogs (*n =* 68, weighing less than 20 kg) diagnosed with hyperadrenocorticism by adrenocorticotropic hormone (ACTH) stimulation test were included. The dogs were given trilostane after diagnosis (starting dose: 1 mg/kg q 12 h), and the ACTH stimulation test was performed periodically to monitor treatment response. Stable state was defined as having post‐ACTH cortisol concentration within 2.0–9.0 μg/dL and clinical improvement. The dose of trilostane in the stable state was regarded as the optimal dose.

**Methods:** Retrospective study.

**Results:** A positive correlation was observed between post‐ACTH cortisol concentration at diagnosis and the optimal dose. However, in 61 of 68 dogs, the optimal dose was less than 2.5 mg/kg q 12 h, which slightly exceeds the authors’ starting dose. Therefore, it is not clinically significant to determine the initial dose of trilostane in proportion to the post‐ACTH cortisol concentration at diagnosis.

**Conclusions and Clinical Importance:** Starting at a low dose of 1 mg/kg q 12 h regardless of post‐ACTH cortisol concentration at diagnosis is desirable to reach the optimal dose to reduce the side effects caused by trilostane in small‐breed dogs.

**Figure:**
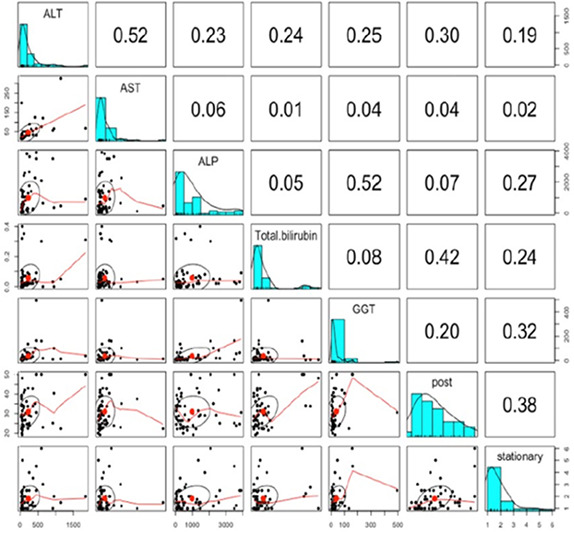
Figure 1. Correlations and histograms between the variables

**Table:** Table 1. Optimal dose of trilostane

Optimal dose of trilostane (mg/kg)	No. of dogs	Percentage (%)	Cumulative percentage (%)
1	20	29.41	29.41176
1–1.25	6	8.82	38.23529
1.25–1.5	13	19.12	57.35
1.5–1.75	3	4.41	61.76
1.75–2.0	9	13.24	75.00
2.0–2.25	2	2.94	77.94
2.25–2.5	8	11.76	89.71
>2.5	7	10.29	100
**Total number of cases**	68	100	

## Abstract EN27: New Generation Device for Blood Glucose Monitoring in Dogs

### **Samuel D. Stewart** ^1^; Kenneth Palladino^2^, DVM

#### ^1^Ethos Veterinary Health; ^2^Internal Medicine, Ethos Veterinary Health

**Background:** Accurate methods for blood glucose measurement are imperative to directing therapy in patients with dysglycemic conditions. AlphaTRAK 2 (AT2) Blood Glucose Monitoring System is currently available for glucose measurement in dogs and cats. A newer generation device, AlphaTrak 3 (AT3), has been developed to continue to provide accurate blood glucose monitoring.

**Objectives:** Demonstrate clinical accuracy of AT3 beta device in a clinical cohort of dogs compared to reference method.

**Animals:** 108 client‐owned dogs with normoglycemia and dysglycemia.

**Methods:** 3 mL blood was collected from each dog. Whole blood aliquots were immediately tested concurrently on three AT3 devices using three lots of test strips. Plasma glucose was also measured in triplicate by a Beckman Coulter Chemistry Analyzer. Success defined as 95% of measured values falling within the International Organization for Standardization (ISO) accuracy threshold and 99% of measurements falling within zones A and B of the Consensus Error Grid (CEG). A linear regression model was also used to demonstrate correlation.

**Results:** 308 of 324 glucose measurements (95.1%) fell within the ISO accuracy threshold. 100% of measurements fell within CEG zones A and B. The linear regression analysis had a correlation coefficient (R²) of 0.98, with a slight positive bias in glucose measurements of 500–630 mg/dL.

**Conclusions and Clinical Importance:** The analytical performance of AlphaTrak 3 was comparable to the reference over a range of canine blood glucose measurements, confirming that AlphaTrak 3 met the ISO success criteria and can be utilized for clinical decision making in diabetic dogs.

## Abstract EN28: Comparing Non‐Esterified Fatty Acids Between Diabetic Dogs and Healthy Dogs and Possible Omega‐3 Effects

### **Fabio A. Teixeira** ^1^; Vinicius Oliveira^1^; Gabriel Santos^1^; Tatiane Pooli^1^; Cristiana Pontieri^2^; Marcio Brunetto^1^

#### ^1^School of Veterinary Medicine and Animal Science of University of Sao Paulo/Nutricare Vet; ^2^Grandfood Ind and Com LTDA, Premier Pet

**Background:** Non‐esterified fatty acids (NEFA) play a complex role in glucidic and lipidic homeostasis and are converted to β‐hydroxybutyrate (BHB). Omega‐3 is considered useful in metabolic disorders.

**Objectives:** To compare NEFA and BHB concentrations between diabetic dogs and healthy dogs and to investigate the effects of omega‐3 supplementation on those parameters.

**Animals:** Healthy dogs (convenience sample) and diabetic dogs from Veterinary Teaching Hospital with adequate glycemic control and non‐obese.

**Methods:** Twenty‐two healthy dogs (HE) and 21 diabetic dogs (DM) ate for three months the same higher‐fiber extruded diet. From those, seventeen diabetic dogs concluded a randomized double‐blind trial with that diet (without omega‐3; DMc) and the same diet enriched with 5.0% inclusion fish oil (DMω3), for three months each period. Fasting serum NEFA and BHB were measured after each period. Data of HE vs. DM and DMc vs. DMω3 were compared using T‐test or Wilcoxon test (*P* < 0.05).

**Results:** Diabetic dogs had higher mean (±standard deviation) serum NEFA (1.52 ± 0.72 vs. 0.85 ± 0.40 mMol/L; *P* < 0.001) and BHB (0.12 ± 0.07 vs. 0.09 ± 0.02 mMol/L; *P* = 0.03) concentrations than healthy dogs. Supplementation of omega‐3 was approximately 142 mg EPA+DHA/kg body weight. There were no differences in serum NEFA (*P* = 0.32) and BHB (*P* = 0.48) concentrations among dogs receiving or not omega‐3.

**Conclusions and Clinical Importance:** Despite being under good glycemic control, diabetic dogs still have increased concentrations of NEFA and BHB, which are not affected by omega‐3 supplementation. More studies are needed to investigate whether these would be parameters for monitoring the treatment of diabetic dogs.

## Abstract GI01: ADAMTS13 Activity in Dogs with Chronic Enteropathies

### **Samantha Barth** ^1^; Ashley Wilkinson^2^, DVM, MS, DACVIM (SAIM); Stefanie DeMonaco^3^, DVM, MS, DACVIM (SAIM); Katie Boes^4^, DVM, MS, DACVP (Clinical Pathology); Bobbi Conner^5^, DVM, DACVECC

#### ^1^Virginia‐Maryland College of Veterinary Medicine, Blacksburg, VA, USA; ^2^Assistant Professor, Department of Small Animal Clinical Sciences, Virginia‐Maryland College of Veterinary Medicine, Blacksburg, VA, USA; ^3^Associate Professor, Veterinary Clinical Sciences, Long Island University, Brooklyn, NY, USA; ^4^Diagnostic Clinical Pathologist, Antech Diagnostics, Englewood, CO, USA; ^5^Clinical Associate Professor, Department of Small Animal Clinical Sciences, Virginia‐Maryland College of Veterinary Medicine, Blacksburg, VA, USA

**Background:** Chronic enteropathies (CE) predispose dogs to thromboembolic disease, but the underlying mechanisms are poorly understood. Humans with CE have decreased activity of a disintegrin and metalloproteinase with a thrombospondin type 1 motif, member 13 (ADAMTS‐13), a von Willebrand factor (vWF) cleaving enzyme, and increased circulating vWF. The primary aim of this study is to determine whether dogs with CE have reduced ADAMTS13 activity, increased plasma vWF antigen concentration (vWF:Ag), and increased vWF:collagen binding activity (vWF:CBA) compared to healthy control dogs.

**Hypothesis/Objectives:** Dogs with CE have reduced ADAMTS13 activity, increased vWF:Ag, and increased vWF:CBA compared to healthy control dogs.

**Animals:** Sixteen privately‐owned dogs with CE and 37 healthy dogs were recruited from a hospital population.

**Methods:** Prospective observational study. Dogs will be identified as having CE using histopathology. ADAMTS13 activity will be measured using a commercially available ELISA kit (Diapharma). ADAMTS13 activity will be assessed in 20 dogs with CE and 40 healthy control dogs. Plasma vWF:Ag and vWF:CBA will be assessed in 20 dogs with CE and 20 healthy control dogs.

**Results:** Preliminary analysis reveals that vWF:Ag and vWF:CBA are significantly decreased in dogs with CE compared to healthy dogs (*P* = 0.005 and *P* = 0.03, respectively). ADAMTS13 activity is not significantly different between dogs with CE and healthy dogs (*P* = 0.12).

**Conclusions and Clinical Importance:** Circulating vWF:Ag and vWF:CBA are reduced in dogs with CE. ADAMTS13 activity is not reduced in dogs with CE and is unlikely to be a mechanism for hypercoagulability associated with CE.

## Abstract GI02: Clinicopathological Findings, Treatment and Outcome of 60 Cats With Feline Gastrointestinal Eosinophilic Sclerosing Fibroplasia

### **Petra Cerna** ^1^; Cristobal Lopez‐Jimenez^2^; Kenjiro Fukushima^3^; Ko Nakashima^4^; Taisuke Nakagawa^5^; Fiona Adam^2^; Anna Groth^2^; Andrew Denning^2^; Nicolas Israeliantz^6^; Danièlle Gunn‐Moore^6^

#### ^1^Colorado State University, Fort Collins, CO, USA; ^2^North Downs Specialist Referrals, Bletchingley, UK; ^3^Veterinary Specialists & Emergency Center; ^4^Small Animal Medical Center; ^5^Graduate School of Agricultural and Life Sciences, Veterinary Medical Centre, The University of Tokyo, Tokyo, Japan; ^6^The Royal (Dick) School of Veterinary Studies, University of Edinburgh, Midlothian, Scotland, UK

**Background:** Feline gastrointestinal eosinophilic sclerosing fibroplasia (FGESF) presents as mass(es) associated with the gastrointestinal tract, mesentery, and abdominal lymph nodes.

**Hypothesis/Objectives:** To report the clinicopathological findings, treatment, and outcome of cats with FGESF.

**Animals:** Sixty client‐owned cats diagnosed with FGESF.

**Methods:** Retrospective review of medical records of cats with histopathologically confirmed FGESF.

**Results:** The median age of the cats was 5.4 years (range 1.3–14.5); 30% were Domestic Shorthairs and 12% were Domestic Longhair cats, with the most prevalent pedigree breeds being Ragdolls (25%), Exotic Shorthair (10%) and Persian (8%) cats. The mean duration of clinical signs was 182 days (range 3–1080); the most common clinical signs were weight loss (60%), hyporexia/anorexia (55%), chronic vomiting (37%), lethargy (35%) and chronic diarrhea (27%). Abdominal mass was palpated in 58% of the cases; located in the small intestine (32%), stomach (27%), ileocolic junction (15%), colon (10%), lymph node (8%) and mesentery (8%). Eosinophilia was present in 50% and hypoalbuminemia in 28% of cats. The mass was removed surgically in 37% of cases. Most cats (98%) were treated with corticosteroids and 1 cat with antibiotics alone. The mean survival was 925 days (range 44–2689), and this was not statistically different between cats treated with surgical resection and cats treated with medical therapy alone, 88% of the cats still alive at the time of writing.

**Conclusions and Clinical Importance:** FGESF is an important differential diagnosis for abdominal masses in cats, and has a much better prognosis than previously reported.

## Abstract GI03: Management of Benign Esophageal Strictures in Dogs and Cats—Long‐Term Follow‐Up of 32 Cases

### **Mary K. Bollman** ^1^; Frederic Gaschen^2^, Dr med. vet., Dr habil., DACVIM (SAIM), DECVIM‐CA (IM), AGAF

#### ^1^Louisiana State University, Baton Rouge, LA, USA; ^2^Professor, Veterinary Clinical Sciences, Louisiana State University, Baton Rouge, LA, USA

**Background:** Benign esophageal strictures (BES) may compromise quality of life (QoL) and survival in dogs and cats. Little data is available on long‐term survival after management.

**Hypothesis/Objectives:** To describe the outcome of BES in dogs and cats treated with esophagoscopy‐guided balloon dilation and identify factors influencing the outcome.

**Animals:** 28 dogs and 4 cats.

**Methods:** Retrospective analysis (2006–2022). Signalment, BES number and etiology, submucosal injection and triamcinolone acetate dose, PEG‐tube placement, and number of dilations were recorded. The animals’ owners were contacted to obtain survival data and diet information. Effects of recorded variables on survival were evaluated with Cox proportional hazards regression.

**Results:** Median [range] values were as follows: age, 6.5 years [0.3–14]; body weight, 5.45 kg [1–37]; number of BES present at initial visit 1 [1–5]. Identified causes for BES were peri‐anesthetic regurgitation (17), esophageal foreign body (8), and vomiting (7). Twenty‐two animals received submucosal injections of triamcinolone (median dose 0.5 mg/kg [0.22–0.76]). A PEG‐tube was placed in 11 animals. A median of 2 dilations [1–6] was performed. Follow‐up data were obtained for 26 animals. Ten could eat kibbles and 16 required a soft diet. Most animals had good QoL. Median survival time was 2,746 days [11–4,191]. Increased age at the time of diagnosis negatively impacted outcome (HR 1.39, 95% CI 1.11–1.85, *P* = 0.009).

**Conclusions and Clinical Importance:** Balloon dilation afforded prolonged survival with good QoL in dogs and cats with BES. More than one procedure was required in most cases. Increased age at presentation had a negative prognostic value.

## Abstract GI04: Evaluation of KI‐67, Goblet Cell and MUC2 Mucin Expression in Canine Lymphoplasmacytic and Granulomatous Colitis

### **Chelsea Lim** ^1^; Caroline Mansfield^2^, BSc, BVMS, MVM, PhD, MANZCVS, DECVIM‐CA; Julien Dandrieux^3^, BSc, Dr med vet, PhD, DACVIM (SAIM); Richard Ploeg^3^, BVSc, DACVP; Simon Firestone^3^, BSc, BVSc, MAppEpi, PhD, MANZCVS; Cameron Nowell^4^, BSc

#### ^1^University of Melbourne, Melbourne, Australia; ^2^School of Veterinary Science, University of Queensland, Brisbane, Australia; ^3^Faculty of Science, Melbourne Veterinary School, University of Melbourne, Melbourne, Australia; ^4^Monash Institute of Pharmaceutical Sciences, Monash University, Melbourne, Victoria, Australia

**Background:** It is unknown if Ki‐67 expression shows potential as a marker of intestinal inflammation, or if goblet cell (GbC) depletion correlates with decreased MUC2 mucin expression or inflammation severity in dogs with chronic colitis.

**Objectives:** To compare Ki‐67, GbC and MUC2 expression in endoscopic biopsies from dogs with granulomatous colitis (GC), lymphoplasmacytic colitis (LPC), and normal colon (NC).

**Animals:** 48 client‐owned dogs [GC (*n =* 19), LPC (*n =* 19), NC (*n =* 10)].

**Methods:** Sections of formalin‐fixed, paraffin‐embedded endoscopic colonic biopsies were stained using Ki‐67 immunohistochemistry; RNAscope *in situ* hybridization was performed using customized canine MUC2‐targeted probes. Five microscopic fields per dog were selected for measuring Ki‐67 labelling index (Ki67%), proportions of GbC staining (Gbc%) and MUC2‐positive cell staining (MUC2%) using image analysis software (FIJI). Data was analyzed using Pearson's correlation and linear regression.

**Results:** Median normalized Ki67% in NC dogs (6.69%; 95% ‐CI, 2.94%–12.72%), was higher compared to LPC (4.91%; 95% ‐CI, 3.94%–7.89%) and GC dogs (1.13%; 95% ‐CI, 0.79%–2.04%). Median GbC% did not correlate with severity of colonic inflammation overall. MUC2 expression was highest in NC dogs. Colonic inflammation was associated with markedly decreased MUC2 expression in LPC (*r =* −0.917; 95% CI, −0.991–0.411) but not GC dogs (*r =* 0.078; 95% ‐CI, −0.663–0.742). MUC2% and GbC% were positively correlated in NC (*r =* 0.911; 95% ‐CI, 0.503–0.987) but not GC and LPC dogs.

**Conclusion:** Variations in MUC2 expression between GC and LPC dogs reflect differences in pathways regulating MUC2 biosynthesis and secretion. MUC2 expression levels with GC and LPC are influenced by mechanisms modulating GbC function rather than quantity.

## Abstract GI05: Comparison of Gastroesophageal Reflux Between Nasoesophageal and Nasogastric Tubes in Dogs Undergoing Enteral Nutrition

### **Douglas Margarucci** ^1^; John Thomason^2^, DVM, MS, DACVIM (SAIM); Andrew Mackin^2^, BVMS, MVS, DVSc, DACVIM (SAIM); Todd Archer^2^, DVM, MS, DACVIM (SAIM); Robert Wills^3^, DVM, MS, PhD, DACVPM

#### ^1^College of Veterinary Medicine, Mississippi State University, Mississippi State, MS, USA; ^2^Department of Clinical Sciences, College of Veterinary Medicine, Mississippi State University, Mississippi State, MS, USA; ^3^Department of Pathobiology and Population Medicine, College of Veterinary Medicine, Mississippi State University, Mississippi State, MS, USA

**Background:** Nasoenteric feeding tubes are commonly used to provide short‐term enteric nutrition in dogs. However, it is unknown if the placement of the tube through the lower esophageal sphincter increases the risk of gastroesophageal reflux.

**Hypothesis/Objectives:** Our objective was to compare evidence of gastroesophageal reflux between dogs with nasogastric (NG) and nasoesophageal (NE) tubes.

**Animals:** Twelve healthy research dogs.

**Methods:** Prospective, randomized cross‐over study. Nasal feeding tubes were positioned with the distal tip terminating in either the esophagus or stomach. Continuous mucosal pH was recorded by radiotelemetric pH capsules in the distal esophagus. Following a recovery period, the study was repeated with the other type of feeding tube. The effect of tube type on a series of reflux parameters was assessed in separate T‐tests while accounting for the crossover design.

**Results:** The mean analyzed study time was 85.81 (SD = 3.57) and 86.43 (SD = 3.99) hours for NE and NG tubes, respectively. There were no significant differences between NE and NG tubes in the time with esophageal pH <4 (mean ± standard deviation, 2.22% ± 2.75 and 2.59% ± 3.62, *P* = 0.446), number of reflux episodes/hour (0.54 ± 0.67 and 0.44 ± 0.44, *P* = 0.369), number of reflux episodes >5 min/h (0.06 ± 0.08 and 0.08 ± 0.11, *P* = 0.353), and duration of longest reflux episode (15.77 ± 13.94 and 20.36 ± 21.27 min, *P* = 0.291), respectively.

**Conclusions and Clinical Importance:** For all parameters measured, there was no significant difference between tube type in healthy dogs.

## Abstract GI06: Expanded Fecal Bile Acid Profile in Dogs with Chronic Enteropathy

### **Amanda B. Blake**; Chih‐Chun Chen; Jonathan Lidbury; Joerg Steiner; Jan Suchodolski

#### Gastrointestinal Lab, Texas A&M University, College Station, TX, USA

**Background:** Bile acids (BA) are key signaling molecules in the intestine, regulating metabolism and inflammation, and maintaining gut health. Some BA produced through gut microbial isomerization (iso‐BA) or oxidation (oxo‐BA) of BA are relatively abundant members of the human fecal BA pool, but have not yet been fully characterized in dogs.

**Objectives:** Compare fecal BA concentrations between dogs with chronic enteropathy (CE) and healthy control dogs (HC).

**Animals:** 10 dogs with CE and 10 HC dogs.

**Methods:** Retrospective cross‐sectional study. Fecal concentrations of 30 BA (including unconjugated, taurine‐ and glycine‐conjugated, and iso‐ and oxo‐BA) were measured with an LC‐MS/MS method using a novel online solid phase extraction method. BA concentrations were compared between groups with Mann‐Whitney tests.

**Results:** Fecal concentrations of primary unconjugated BA, primary glycine‐conjugated, taurocholic, iso‐cholic, and 3‐oxo‐cholic acid were significantly higher in dogs with CE than in HC dogs. Fecal concentrations of secondary unconjugated, secondary glycine‐conjugated, iso‐chenodeoxycholic, and 3‐oxo‐deoxycholic acid were significantly lower in dogs with CE than in HC dogs (Table 1). In HC dogs, iso‐BA comprised 5–23% (median: 12%) of the total BA pool, and oxo‐BA comprised 12–31% (median: 25%) of the total BA pool.

**Conclusions:** Oxo‐ and iso‐BA are abundant members of the fecal BA pool in dogs and show altered concentrations in dogs with CE. Future studies are warranted to elucidate the role these BA play in canine CE, as well as their relation to members of the intestinal microbiota.

**Table:** Table 1. Fecal bile acid concentrations in dogs with chronic enteropathy (CE) compared to healthy control dogs (HC)

Fecal Bile Acid	HC (median [range] ng/mg fecal dry matter)	CE (median [range] ng/mg fecal dry matter)	*P* value
Cholic acid	196 [10–661]	701 [447–1004]	<0.001
Chenodeoxycholic acid	65 [4–344]	545 [109–802]	<0.001
Deoxycholic acid	575 [68–696]	23 [3–1004]	0.043
Lithocholic acid	259 [3–518]	8 [0.3–427]	0.005
Hyodeoxycholic acid	371 [11–696]	27 [1–353]	0.011
Glycocholic acid	0.3 [0.0–3.0]	7 [2–88]	<0.001
Glycochenodeoxycholic acid	0.2 [0.0–0.7]	0.9 [0.1–23]	0.016
Glycodeoxycholic acid	1.1 [0.0–5.8]	0.0 [0.0–2.7]	0.028
Glycolithocholic acid	0.4 [0.0–1.4]	0.0 [0.0–0.2]	0.001
Taurocholic acid	5 [2–63]	115 [3–6548]	0.027
Iso‐cholic acid	22 [1–95]	225 [0.0–498]	0.011
Iso‐chenodeoxycholic acid	268 [14–570]	40 [10–333]	0.011
3‐oxo‐cholic acid	4 [0.5–37]	40 [3–1961]	0.004
3‐oxo‐deoxycholic acid	187 [4–292]	0.4 [0.0–56]	<0.001

## Abstract GI07: Efficacy of Oral or Injectable Supplementation With Cobalamin in Cats With Hypocobalaminemia

### **Chee‐Hoon Chang**; Jonathan Lidbury; Jan Suchodolski; Joerg Steiner

#### Texas A&M University, College Station, TX, USA

**Background:** A previous study has shown equivalent efficacy of oral and injectable cobalamin supplementation in dogs with hypocobalaminemia. In cats, only one retrospective study evaluating oral supplementation has previously been reported.

**Objectives:** To investigate the efficacy of oral or injectable supplementation with cobalamin in hypocobalaminemic cats with chronic enteropathy (CE) or exocrine pancreatic insufficiency (EPI).

**Animals:** 48 client‐owned cats with hypocobalaminemia.

**Methods:** Prospective randomized trial. Cats with CE or EPI were randomly assigned to two groups that received either oral (250 μg of cyanocobalamin [Cobalaquin, Nutramax Laboratories Veterinary Sciences] per cat, daily for 12 weeks total) or injectable (250 μg of cyanocobalamin [vitamin B‐12, VEDCO] per cat, weekly injection for 6 weeks with an additional dose 4 weeks later) supplementation. During the study, diet or other medical treatments for CE or EPI were determined by the primary care veterinarian of each cat. Each cat was examined three times with serum cobalamin and methylmalonic acid (MMA) concentrations being measured at each visit.

**Results:** Serum cobalamin concentrations significantly increased with either oral or injectable supplementation in cats with CE or EPI. Serum MMA concentrations significantly decreased with either oral or injectable supplementation with cobalamin in cats with CE or EPI (Table 1).

**Conclusions and Clinical Importance:** Both oral or injectable supplementation with cobalamin are effective in normalizing serum cobalamin and MMA concentrations in hypocobalaminemic cats with CE or EPI.

**Table:** Table 1. Results (medians, ranges, and *P* values) of serum cobalamin and methylmalonic acid (MMA) concentrations at two time points (baseline and second recheck) in cats with chronic enteropathy (CE) or exocrine pancreatic insufficiency (EPI) that received either oral or injectable cobalamin supplementation. The median difference between the two time points and the 95% confidence interval between the two time points are also shown.

Measurement (Reference interval)	Group	Treatment	Baseline		Second recheck		*P* value	Median difference	95% CI
			Median	Range	Median	Range			
Cobalamin (RI:290–1,500 ng/L)	CE	Oral	149	149–244	3,638	509–55,139	<0.01	3,456	970 to 8,610
		Injectable	149	149–196	1,824	969–6,424	<0.01	1,675	1,122 to 2,658
	EPI	Oral	149	149–283	1,001	352–16,873	0.03	852	203 to 16,724
		Injectable	149	149–287	3,923	1,001–4,749	<0.01	2,629	852 to 3,785
MMA (RI:139–898 nmol/L)	CE	Oral	3,034	406–27,390	331	169–26,889	<0.01	−1,678	−10,257 to −333
		Injectable	2,239	381–28,175	468	191–1,480	<0.01	−1,398	−5,565 to ‐405
	EPI	Oral	26,912	1,190–30,342	399	222–1,264	0.02	−26,152	−30,120 to −356
		Injectable	20,678	1,022–27,878	242	170–1,325	<0.01	−20,436	−27,445 to −827

## Abstract GI08: Evaluation of the Abundance of *Ruminococcus gnavus* in Dogs with Chronic Enteropathies

### **Chih‐Chun Chen** ^1^; Chi‐Hsuan Sung^1^; Linda Toresson^2^; Rachel Pilla^1^; Jonathan Lidbury^1^; Jörg Steiner^1^; Jan Suchodolski^1^

#### ^1^Gastrointestinal Laboratory, Department of Small Animal Clinical Sciences, Texas A&M University, College Station, TX, USA; ^2^Evidensia Specialist Animal Hospital, Stockholm, Sweden

**Background:** Based on metagenomic DNA shotgun sequencing analysis, *Ruminococcus gnavus* is one of the most abundant bacterial species in both the human and the canine fecal microbiota. The abundance of *R. gnavus* has been reported to be increased in humans with inflammatory bowel disease and plays a role in bile acid (BA) metabolism. However, there is limited information regarding *R. gnavus* and its role in dogs with chronic enteropathy (CE).

**Objectives:** Describe the fecal abundance of *R. gnavus* from dogs with CE in comparison to healthy control dogs (HC) and its association with BA metabolism.

**Animals:** 75 dogs with CE and 37 HC dogs.

**Methods:** Retrospective cross‐sectional study. Fecal abundance of *R. gnavus* and dysbiosis index (DI) were quantified by qPCR. Fecal concentration of unconjugated primary and secondary BAs were measured by GC‐MS. Fecal *R. gnavus* abundance was compared by Mann‐Whitney U tests. The correlation analysis between fecal *R. gnavus* abundance and DI, and fecal *R. gnavus* abundance and BA composition were evaluated by Spearman's correlation test.

**Results:** No significant difference (*P* = 0.61) was found in fecal *R. gnavus* abundance between HC dogs (median[range]: 10.4[6.1–11.8] log DNA) and dogs with CE (10.2[6.0–12.4]). No significant correlations were found between fecal *R. gnavus* abundance and DI (rs = −0.08; *P* = 0.41) nor fecal *R. gnavus* abundance and percentage of primary BAs (rs = 0.01; *P* = 0.90).

**Conclusions and Clinical Importance:** Our results do not support a role of *R. gnavus* in canine CE.

## Abstract GI09: Stability of Frozen and Lyophilized *Clostridium hiranonis* in Fecal Microbiota Transplantation Preparations

### **Bruna Correa Lopes** ^1^; M. Katherine Tolbert^2^; Paula Giaretta^3^; Jan Suchodolski, MedVet, DrVetMed, PhD, AGAF, DACVM^4^; Rachel Pilla^5^

#### ^1^Texas A&M University, College Station, TX, USA; ^2^Clinical Associate Professor in Small Animal & Comparative Gastroenterology, Department of Small Animal Clinical Sciences, Texas A&M University, College Station, TX, USA; ^3^Clinical Assistant Professor, Department of Small Animal Clinical Sciences, Texas A&M University, College Station, TX, USA; ^4^Associate Professor of Small Animal Internal Medicine and Associate Director for Research of the GI Lab, Department of Small Animal Clinical Sciences, Texas A&M University, College Station, TX, USA; ^5^Research Assistant Professor, Department of Small Animal Clinical Sciences, Texas A&M University, College Station, TX, USA

**Background:** *Clostridium hiranonis* is a key player in bile acid metabolism and its decreased abundance has been observed in dogs with chronic enteropathies. Different protocols to conserve fecal microbiota transplantation (FMT) preparations are used in the treatment of chronic enteropathies, but the viability of *C. hiranonis* in those preparations is unknown.

**Objectives:** To assess abundances of *C. hiranonis* over time in the feces of healthy dogs using different methods and temperatures to conserve feces: lyophilization and storage at 4°C and −20°C, and freezing with glycerol at −20°C.

**Animals:** Six healthy dogs.

**Methods:** Abundances of *C. hiranonis* were quantified by bacterial culture using Brucella Blood Agar at 36°C in an anaerobic environment. Friedman test was used to compare the abundances of *C. hiranonis* across time points.

**Results:** All methods resulted in lower *C. hiranonis* viability, but the decrease in *C. hiranonis* abundance was significant only in lyophilized samples kept at 4°C and −20°C after one month (*P* < 0.0001 and *P* = 0.0055, respectively). *C. hiranonis* could not be cultured in 4/6 lyophilized samples kept at 4°C after one month but was cultured in 6/6 lyophilized samples kept at −20°C for one month.

**Conclusions and Clinical Importance:** While all storage methods led to a decrease in the abundance of *C. hiranonis*, a relatively high abundance of organisms could be detected in lyophilized feces kept at −20°C and in frozen feces with glycerol. Both methods were satisfactory to conserve viable *C. hiranonis* for FMT preparations.

## Abstract GI10: Assessment of Mucosal Fibrosis and its Correlation With Disease Severity in Cats With Chronic Enteropathy

### **Evan J. Cosgrove** ^1^; Valérie Freiche^2^; Paula Giaretta^3^; Sina Marsilio^1^

#### ^1^School of Veterinary Medicine, University of California Davis, Davis, CA, USA; ^2^Ecole Nationale Vétérinaire d’Alfort, Alfort, France; ^3^School of Veterinary Medicine & Biomedical Sciences, Texas A&M University, College Station, TX, USA

**Background:** Intestinal fibrosis is a major complication in people with inflammatory bowel disease and has been directly correlated to severity of mucosal injury and inflammation. The correlation of mucosal fibrosis with clinical disease activity and underlying diagnosis in cats with chronic enteropathy has not been investigated.

**Hypothesis:** Mucosal fibrosis is correlated to disease severity in cats with chronic enteropathy.

**Animals:** Small intestinal biopsy specimens from 27 cats with chronic enteropathy, 13 cats with lymphoplasmacytic enteritis (LPE) and 14 cats with low‐grade intestinal T‐cell lymphoma (LGITL).

**Methods:** The proportional area of fibrosis in the lamina propria (fractional fibrotic area) was quantified in Masson's‐Trichrome‐stained slides in 3 distinct mucosal regions (villi, apical crypts, and basal crypts). Analysis was performed using the software ImageJ. Results were correlated to a modified clinical disease activity index and the disease status of either LPE or LGITL.

**Results:** Fractional fibrotic area in the villi was significantly negatively correlated with the modified disease activity index (*P* = 0.005). Cats with LPE had higher fractional fibrotic areas (median 12.6%, range 6.3%–35.4%) in the apical crypt area compared to cats with LGITL (median 2.3%, range 0.0%–52.2%) (*P* = 0.05).

**Conclusion:** Cats with higher clinical disease activity show lower degrees of fibrosis in villi, which may be related to higher cellular infiltrates as seen in LGITL. Cats with LPE have more fibrosis in the apical crypt area compared to cats with LGITL. No fractional fibrotic area cut‐off reliably differentiating LPE from LGITL has been established.

## Abstract GI11: Effect of Low‐Fat Diet on Serum Triglyceride and cPLI Concentrations in Dogs with Hypertriglyceridemic Pancreatitis

### **Floris C. Dröes** ^1^; Tim Kretzschmar^2^, DVM; Yuri Lawrence^3^, DVM, PhD, MS, MA, DACVIM (SAIM); Jan Suchodolski^4^, MedVet, DrVetMed, PhD, AGAF, DACVM; Jonathan Lidbury^5^, BVMS, MRCVS, PhD, DACVIM, DECVIM‐CA; S. Dru Forrester^6^, DVM, MS, DACVIM; Jörg Steiner^7^, med.vet., Dr med. vet., PhD, DACVIM, DECVIM‐CA, AGAF

#### ^1^Gastrointestinal Laboratory, School of Veterinary Medicine & Biomedical Sciences, Texas A&M University, Collee Station, TX, USA; ^2^Veterinarian, Dodd Animal Hospital, San Antonio, TX, USA; ^3^Owner, Board‐Certified Small Animal Internal Medicine Specialist, Lawrence Mobile Internal Medicine Consulting Services, PLLC, Austin, TX, USA; ^4^Associate Professor of Small Animal Internal Medicine, Associate Director for Research of the GI Lab, Veterinary Small Animal Clinical Sciences, Gastrointestinal Laboratory, Texas A&M University, College Station, TX, USA; ^5^Assistant Department Head of Research and Graduate Studies, Associate Professor Small Animal Internal Medicine, Associate Director for Clinical Services, Veterinary Small Animal Clinical Sciences, Gastrointestinal Laboratory, Texas A&M University, College Station, TX, USA; ^6^Director, Global Veterinary Innovation, Hill's Pet Nutrition, Inc., Topeka, KS, USA; ^7^Regents Professor and University Distinguished Professor (with Tenure) Small Animal Internal Medicine, Dr Mark Morris Chair in Small Animal Gastroenterology & Nutrition, Director Gastrointestinal Laboratory, Veterinary Small Animal Clinical Sciences, Gastrointestinal Laboratory, Texas A&M University, College Station, TX, USA

**Background:** Low‐fat diets are commonly recommended for management of pancreatitis in dogs. The efficacy of these diets in dogs with hypertriglyceridemic pancreatitis remains unknown.

**Objective:** Assess effects of low‐fat diet on serum triglyceride (TRIG) and cPLI concentrations in dogs with hypertriglyceridemic pancreatitis.

**Animals:** Twenty‐two dogs with serum cPLI >400 μg/L and TRIGs >300 mg/dL.

**Methods:** Double‐blinded randomized incomplete cross‐over study. After pre‐screening (t0), either a low‐fat (LFD; 2.27 g fat/100 kcal) or regular intestinal diet (RID; 4.00 g fat/100 kcal) was randomly assigned. After 3 weeks (t1), each dog was rechecked by physical examination and standard gastrointestinal and biochemistry panels. If improvement was observed (i.e., decreased cPLI, TRIG, or both), the same diet was continued for another 3 weeks. Otherwise, dogs were switched to the other diet for 3 weeks. At end of study (t2), another health screen was performed. Matched pairs (Mann‐Whitney *U*‐test or *t*‐tests) were used to compare timepoints (t0 vs. t1; t0 vs. t2).

**Results:** At t0, serum TRIGs, ALP, and ALT were significantly lower in dogs starting on LFD (*P* < 0.05). Eleven dogs (5 LFD, 6 RID) received the same diet for 6 weeks. LFD did not significantly decrease serum TRIGs after 3 weeks, but significantly decreased TRIGs after 6 weeks. Diet did not affect serum cPLI at any timepoint.

**Conclusions and Clinical Importance:** In dogs with hypertriglyceridemic pancreatitis, LFD decreased serum TRIGs after 6 weeks. However, in this cohort of dogs, LFD did not affect serum cPLI concentrations.

**Table:** Table 1 Serum triglycerides (TRIGs) and canine PLI (cPLI) concentrations in dogs with hypertriglyceridemic pancreatitis fed a regular intestinal diet (RID) or low‐fat intestinal diet (LFD) for 3 weeks until cross‐over

RID (*n =* 11) LFD (*n =* 11)	T0 median [IQR]	T1 median [IQR]	*P* value
TRIGs on RID (in mg/dL)	859 [572–1270]	918 [441–1319]	0.920
TRIGs on LFD (in mg/dL)	501 [329–877]	236 [181–604]	0.053
cPLI on RID (in μg/dL)	616 [511–1001]	561 [279–774]	0.130
cPLI on LFD (in μg/dL)	704 [474–1001]	739 [554–914]	0.770

**Table:** Table 2 Serum triglycerides (TRIGs) and canine PLI (cPLI) in dogs with hypertriglyceridemic pancreatitis fed a regular intestinal diet (RID) or low‐fat intestinal diet (LFD) for 6 weeks without cross‐over

RID (*n =* 6), LFD (*n =* 5)	T0, median [IQR] or mean [± SD]	T2, median [IQR] or mean [± SD]	*P* value
TRIGs on RID (in mg/dL)	876 [727–1280]	882 [368–1317]	0.749
TRIGs on LFD (in mg/dL)	599 [337–928]	243 [185–457]	0.047*
cPLI on RID (in μg/dL)	705 [± 91]	509 [± 91]	0.317
cPLI on LFD (in μg/dL)	843 [± 131]	642 [± 131]	0.211

## Abstract GI12: Microbiome Responses to Oral Fecal Microbiota Transplantation in a Cohort of Domestic Dogs

### **Connie Rojas** ^1^; Zhandra Entrolezo^2^, BS; Jessica Jarett^3^, PhD; Guillaume Jospin^4^, MS; Alex Martin^1^, BA; Jonathan Eisen^5^, PhD; Holly Ganz^6^, PhD

#### ^1^AnimalBiome, Oakland, CA, USA; ^2^Laboratory Manager, Molecular Biology, AnimalBiome, Oakland, CA, USA; ^3^Director of Research, R&D, AnimalBiome, Oakland, CA, USA; ^4^Bioinformatician, R&D, AnimalBiome, Oakland, CA, USA; ^5^University of California, Davis, Davis, CA, USA; ^6^Chief Science Officer, AnimalBiome, Oakland, CA, USA

**Background:** Studies on microbiome responses to fecal microbiota transplants (FMTs) in large cohorts of companion animals are lacking in veterinary medicine but provide useful insights.

**Hypothesis/Objectives:** We examine fecal microbiome changes in dogs receiving an oral FMT treatment.

**Animals:** 71 dogs exhibiting chronic vomiting, diarrhea, constipation, or skin issues were enrolled.

**Methods:** Fecal samples were collected from recipients before and two weeks after FMT treatment with capsules containing lyophilized stool given orally BID for ~25 days. Owners provided information on their dog's health and response to treatment. 16S rRNA sequencing was used to analyze microbiome composition and statistical analyses were conducted using permutational anovas and linear models.

**Results:** 75% of dogs were reported to show improvement in their clinical signs. Before FMT, dogs diagnosed with IBD had distinct microbiome compositions compared to dogs without this diagnosis (*P* = 0.019). FMT recipients had less diverse fecal microbiomes than healthy dogs before FMT and remained less diverse afterwards. After FMT, microbiome composition was significantly related to response to treatment (*P* = 0.039) and diet (*P* = 0.024). Relative abundances of several bacteria changed differentially in FMT recipients. *Clostridium* decreased in those with vomiting and diarrhea (*x̄* −2.86%). *Blautia* increased in dogs with an IBD diagnosis (x̄ 2.03%) and decreased in dogs not diagnosed with IBD (*x̄* −2.51%). *Fusobacterium* increased in dogs with diarrhea (x̄ 8.70%) compared to dogs without clinical signs or with skin issues (x̄ −2.24%).

**Conclusions and Clinical Importance:** Oral capsule FMT treatment was helpful in this population and was accompanied by changes in fecal microbiome composition.

**Table:** Table 1. Summary characteristics for the seventy‐one dogs that underwent oral capsule FMT treatment.

Characteristic	Subcategory	FMT recipients (*N =* 71)
Age, in years		5.52 ± 3.82
Body condition (1−10)		4.69 ± 1.39
Sex	Female	31 (44%)
	Male	40 (56%)
Breed (broad)	Shepherd	11 (15%)
	Poodle	9 (13%)
	Golden retriever	7 (10%)
	Terrier	6 (8%)
	Labrador	4 (6%)
	Other	34 (48%)
Diet	Dry	7 (10%)
	Wet & dry	12 (17%)
	Wet & raw	11 (16%)
	Wet & dry & raw	8 (11%)
	Raw	15 (21%)
	Other diet (including wet; raw & dry)	18 (25%)
Spayed or neutered	Yes	54 (76%)
	No	17 (24%)
Antibiotics	Yes	46 (65%)
	No	25 (35%)
IBD	Yes	21 (30%)
	No	39 (55%)
	Maybe	11 (15%)
Response to FMT	Improved	53 (75%)
	No change	12 (17%)
	Worsened	6 (8%)
Initial clinical signs	Diarrhea (only)	26 (37%)
	Vomiting (only)	7 (10%)
	Vomiting & diarrhea	16 (22%)
	Other (including none, diarrhea & constipation, skin issues, inapettite)	22 (31%)

Seventy‐one dogs exhibiting symptoms of a chronic digestive condition (e.g., diarrhea, vomiting, and/or constipation) were recruited for this study. Owners completed a health and demographic survey on their dogs and collected fecal samples before and two‐weeks after oral capsule FMT treatment.

## Abstract GI13: Five Years of Insights from a Fecal Stool Bank for Domestic Cats and Dogs

### **Holly H. Ganz**; Alex Martin, BA

#### AnimalBiome, Oakland, CA, USA

**Objectives:** To explore the ideal frequency of screening and diagnostic testing for stool banks and to describe the parasites and pathogens observed in this population.

**Animals:** 406 cats and 556 dogs were screened for donation to a stool bank.

**Methods:** Fecal samples were collected from potential donors for a stool bank in the San Francisco Bay Area in California. Samples were submitted for third party testing for parasites and pathogens on a weekly basis. Microbiome testing using 16S rRNA sequencing (Illumina) was also performed on a monthly basis.

**Results:** Of the 962 cats and dogs screened, only 16% (50 cats and 82 dogs) were accepted as donors after screening for parasites, pathogens, and microbiome composition. Pathogen testing on a weekly basis was identified as the ideal time frame, allowing for better detection of *Giardia* infections than monthly testing and we found that daily testing was unnecessary. The top pathogens identified in dogs were *Giardia* and *Cryptosporidium* and the top pathogen in cats was the feline coronavirus. The most common potential pathogen found was *Clostridium perfringens*, which was found in more than half of the individuals screened. The parasites, hookworm, roundworm, and whipworm were rarely found in this population.

**Conclusions:** Identification and maintenance of healthy donors for stool banks requires extensive monitoring, particularly for parasites, and pathogens.

**Table:** Table 1. Donor samples submitted for diagnostic testing for pathogens and parasites from June 2022 to January 2023.

Species	Total samples submitted	366
Cat	Feline coronavirus (+)	6
Cat	*Salmonella* (+)	2
Dog	*Campylobacter jejuni*	18
Dog	*Cryptosporidium*	9
Dog	*Salmonella*	5
Dog	Hookworm	4
Dog	Canine circovirus	3
Dog	*Giardia*	3
Dog	*Clostridium difficile* toxin A	2
Dog	*Clostridium difficile* toxin B	2
Dog	Canine enteric coronavirus	2

## Abstract GI14: Serum Concentrations of Calprotectin, S100A12, and Alpha1‐Proteinase Inhibitor in Dogs With Pancreatitis

### **Romy M. Heilmann** ^1^; Annina Jandel^2^; Henri Sander^2^; Joerg Steiner^3^, DVM, DACVIM (SAIM), DECVIM‐CA, PhD, AGA Fellow; Panagiotis Xenoulis^4^

#### ^1^College of Veterinary Medicine, University of Leipzig, Leipzig, Germany; ^2^Department for Small Animals, College of Veterinary Medicine, University of Leipzig, Leipzig, Germany; ^3^Professor of Small Animal Internal Medicine and Director of the Gastrointestinal Laboratory, School of Veterinary Medicine and Biomedical Sciences, Texas A&M University, College Station, TX, USA; ^4^Professor of Small Animal Internal Medicine, Clinic of Medicine, Faculty of Veterinary Science, University of Thessaly, Volos, Greece

**Background:** Miniature Schnauzers are predisposed to develop pancreatitis, with familial hypertriglyceridemia (HTG) being a potential risk factor. Diagnosing pancreatitis in dogs is currently based on the integration of serum canine specific pancreatic lipase (cPLI) concentration, clinical presentation, and diagnostic imaging findings.

**Objective:** To investigate markers of systemic inflammation and antiprotease action in dogs with pancreatitis.

**Animals:** Miniature Schnauzers (*n =* 35) with suspected pancreatitis (serum cPLI >400 μg/L, clinical signs, abdominal imaging).

**Methods:** Calprotectin, S100A12, and α1‐proteinase (α1PI) concentrations were measured in serum samples. These markers were evaluated for associations with patient characteristics, clinical presentation, risk factors for pancreatitis, and outcome.

**Results:** Serum calprotectin, S100A12, and α1PI concentrations were increased in 57%, 57%, and 58% of dogs, with moderate to strong correlations among these biomarkers. Dogs with the typical presentation of pancreatitis had significantly higher α 1PI concentrations than dogs with an atypical clinical presentation (*P* = 0.0110), and serum α 1PI concentrations >1,579 mg/L distinguished both subgroups with 70% sensitivity and 100% specificity. No difference in serum calprotectin or S100A12 concentrations was seen, but presenting with fewer clinical signs was associated with higher serum calprotectin (*P* = 0.0377) and S100A12 concentrations (*P* = 0.0149) in dogs with a typical pancreatitis presentation. No other associations were detected with patient characteristics, risk factors for pancreatitis (e.g., HTG, medications), or outcome.

**Conclusions:** Biomarkers of systemic inflammation and antiprotease activity are commonly increased in Miniature Schnauzers with pancreatitis. Serum calprotectin and S100A12 have limited utility in differentiating pancreatitis presentations, whereas serum α1PI concentrations might prove to serve as a marker of disease severity.

## Abstract GI15: Dysregulation of the Renin‐Angiotensin‐Aldosterone System (RAAS) and Dependent Electrolyte Transporters in Canine Chronic Inflammatory Enteropathy

### **Romy M. Heilmann** ^1^; Oliver Domenig^2^, Dr; Stefanie Kather^3^; Iwan Burgener^4^, Dr med. vet., DACVIM (SAIM), DECVIM‐CA, PhD; Joerg Steiner^5^, Dr med. vet., DACVIM (SAIM), DECVIM‐CA, PhD, AGA Fellow; Franziska Dengler^6^

#### ^1^College of Veterinary Medicine, University of Leipzig, Leipzig, Germany; ^2^Attoquant Diagnostics, Wien, Austria; ^3^Department for Small Animals, College of Veterinary Medicine, University of Leipzig, Leipzig, Germany; ^4^Division of Small Animal Internal Medicine, University of Veterinary Medicine Vienna, Vienna, Austria; ^5^Department of Veterinary Clinical Sciences, School of Veterinary Medicine and Biomedical Sciences, Texas A&M University, College Station, TX, USA; ^6^Institute of Physiology, Pathophysiology, and Biophysics, University of Veterinary Medicine Vienna, Vienna, Austria

**Background:** Chronic diarrhea with intestinal electrolyte losses is a hallmark sign of canine chronic inflammatory enteropathy (CIE). Compared to hyponatremia predominating in human inflammatory bowel disease, hypokalemia is more prevalent in canine CIE suggesting species‐specific counter‐regulation of potential therapeutic relevance.

**Objective:** To investigate intestinal electrolyte transporters and components of the renin‐angiotensin‐aldosterone system (RAAS) in canine CIE.

**Animals:** Eight dogs with CIE and 12 healthy controls.

**Methods:** Serum RAAS fingerprint analysis was performed by mass spectrometry (5 CIE, 5 controls), and mRNA levels of electrolyte transporters and ATGR1 were quantified by RT‐qPCR in ileal (7 CIE, 10 controls) and colonic (6 CIE, 12 controls) tissue biopsies. The results were compared between groups of dogs and tested for associations among each other and with clinical variables in CIE dogs.

**Results:** Components of traditional and alternative RAAS pathways were increased in CIE compared to health, with statistical significance for Ang I, Ang II, and Ang 1‐7 (all *P* < 0.037). Abundances of ileal (but not colonic) Na+/K+‐ATPase, ENaC, and NHE3 (all *P* < 0.023) mRNA (but not ATGR1) were increased in CIE.

**Conclusions:** Differential activation of ileal and colonic electrolyte transporters could be an efficient mechanism of sodium preservation in canine CIE, with the signaling mediated by alternative RAAS components and lack of AGTR1 dysregulation suggesting direct endocrine RAAS effects. Unchanged colonic electrolyte transporter expression in CIE dogs with marked diarrhea suggests the compensatory reserve of the colon is not activated and may reflect the dual role of moderate vs. high systemic Ang II levels.

## Abstract GI16: Efficacy of Two Colonoscopy Preparation Protocols in Dogs

### **Molly Doyle**; Tracy Hill; Laura Motschenbacher

#### University of Minnesota, Minneapolis, MN, USA

**Background:** Colonoscopy is amongst the most common small animal endoscopic procedures, yet there are no clear guidelines for effective colonic preparation protocols. Continuous rate infusions (CRI) of polyethylene glycol (PEG) through a feeding tube and oral administration of an oral laxative agent are two of the more common protocols.

**Hypothesis/Objective:** CRIs of PEG through a nasogastric feeding tube provides better colonic preparation than an oral laxative bisacodyl tablet.

**Animals:** 10 client‐owned dogs receiving colonoscopy for investigation of chronic gastrointestinal signs.

**Methods:** Dogs were randomized to receive either a CRI of PEG through a nasogastric tube or administered bisacodyl tablets the afternoon prior to colonoscopy. All dogs received enemas in addition to the laxative agent. Colonoscopies were recorded and blindly scored, with each colonic segment (ascending, transverse, descending) independently scored 0–3 for a total score of 0–9.

**Results:** Mean scores for PEG CRI preparation were higher than oral laxative [6.9 (95% CI 5.25, 8.55) vs. 2.75 (95% CI 0.69, 4.81), *P* = 0.017]. All 5/5 dogs receiving PEG CRIs were adequately prepared (total score >5) as compared to 0/5 of the oral bisacodyl dogs.

**Conclusions and Clinical Relevance:** PEG solution administered as a CRI through a nasogastric feeding tube more effectively prepares colons in dogs receiving colonoscopy.

## Abstract GI17: A Transfer Learning Approach to Intestinal Image Analysis Differentiates Treatment Response in Canine Protein‐Losing Enteropathy

### **Aarti Kathrani**; Kenneth Ancheta; Isla Trewin; Jonathan Williams

#### Royal Veterinary College, London, UK

**Background:** Dogs with inflammatory protein‐losing enteropathy have a guarded prognosis, with death occurring as a result of the disease in approximately 50% of cases. Although dogs treated with dietary therapy alone are significantly associated with a positive outcome, there is limited ability to differentiate between food‐responsive protein‐losing enteropathy (FR‐PLE) and steroid‐responsive protein‐losing enteropathy (SR‐PLE) at diagnosis.

**Objective:** To determine if a transfer learning computational approach to image classification on intestinal biopsy specimens collected at diagnosis is able to differentiate FR‐PLE from SR‐PLE.

**Animals:** Nine client‐owned dogs diagnosed with inflammatory protein‐losing enteropathy that were subsequently classified based on treatment response into FR‐PLE (*n =* 3) or SR‐PLE (*n =* 6).

**Methods:** A retrospective study using formalin‐fixed, paraffin‐embedded intestinal biopsy specimens collected during diagnostic investigations for protein‐losing enteropathy at a referral veterinary teaching hospital. A machine‐based algorithm was used on 20 and 51 images of intestinal biopsy specimens from dogs with FR‐PLE and SR‐PLE, respectively.

**Results:** Using the pre‐trained Convolutional Neural Network (CNN) model with 70/30 training/test ratio for images, the model was able to differentiate intestinal biopsy images from dogs with FR‐PLE and SR‐PLE with an accuracy of 81.82%.

**Conclusions and Clinical Importance:** Our results suggest that dogs with inflammatory PLE can be predicted as FR‐PLE or SR‐PLE at histopathologic diagnosis through computational approaches. This will help to ensure dogs with inflammatory PLE are prescribed the most appropriate treatment at diagnosis to ensure optimal response and outcome.

## Abstract GI18: Standardized Intestinal Ultrasonography in Cats With Inflammatory Bowel Disease (IBD) and Small Cell Lymphoma (SCL)

### **Peter H. Kook** ^1^; Laura Beatrice^2^, DACVIM; Junwei Föhr^2^; Paula Grest^2^, DECVP; Maja Ruetten^3^, DECVP; Manfred Henrich^4^, DECVP; Karolin Campbell^5^, DECVDI; Paul Torgerson^2^, DECVPH; Peter Kook^1^, DACVIM

#### ^1^Clinic for Small Animal Internal Medicine, Vetsuisse Faculty, University of Zurich, Zurich, Switzerland; ^2^University of Zurich, Zurich, Switzerland; ^3^PathoVet AG, Lindau, Switzerland; ^4^Justus‐Liebig‐Universität Giessen, Giessen, Germany; ^5^Radiovet, Arbon, Switzerland

**Background:** Controversy exists whether histology/immunohistochemistry (IHC) or clonality testing is more appropriate to diagnose IBD and SCL in cats, and whether the diagnostic method affects the ultrasonographic differentiation between both entities.

**Objectives:** To compare utrasonographic measurements between IBD and SCL diagnosed either by H/IHC or clonality testing and to evaluate effects of standardized treatment in a subset of cats.

**Animals:** 16 client‐owned cats.

**Methods:** Duodenal, jejunal, and ileal total wall, mucosa, muscularis propria and submucosa thicknesses were quantified and ratios (muscularis/submucosa, muscularis/total wall thickness) calculated. All cats underwent standardized full‐thickness duodenal, jejunal, and ileal biopsies. Samples for clonality testing were frozen; treatment was based on H/IHC. Intestinal segments were grouped by diagnosis either based on H/IHC or clonality testing and ultrasonographic measurements were compared between IBD/SCL using a linear mixed model. Ultrasonographic measurements were repeated in 7 cats (5 IBD, 2 SCL) after 12 and 24 weeks of treatment. Differences were compared using a mixed model.

**Results:** Based on each of the two methods (H/IHC and clonality testing), 11 cats had IBD and 5 cats had SCL. Discordant results between diagnostic methods were found in 6/16 (38%) cats. Ultrasonographic differences were not found between IBD and SCL independent of the chosen diagnostic method. During treatment, muscularis/total wall thickness ratio decreased by 0.053 cm every 12 weeks of the study (*P* = 0.0254) in IBD cats.

**Conclusions and Clinical Importance:** Regardless of the diagnostic method, ultrasonographic findings did not differ between IBD and SCL. Change over time in IBD cats was minimal and thus of questionable clinical benefit.

## Abstract GI19: Effects of Prednisolone on DGGR‐Lipase Activity (LIPC Roche) and Pancreatic Lipase Immunoreactivity (SpecfPL) in Cats

### **Militsa Pacheva** ^1^; Daniel Brugger^2^; Barbara Riond^3^; Peter Kook^1^, DACVIM

#### ^1^Clinic for Small Animal Internal Medicine, Vetsuisse faculty, University of Zurich, Zurich, Switzerland; ^2^Senior Research Assistant, Institute of Animal Nutrition and Dietetics, Vetsuisse Faculty, University of Zurich, Zurich, Switzerland; ^3^Clinical Laboratory, Department for Clinical Diagnostics and Services, Vetsuisse Faculty, University of Zurich, Zurich, Switzerland

**Background:** Corticosteroids are among the most commonly used drugs in cats and have been discussed as a treatment for feline pancreatitis. However, its effects on serum lipase in healthy cats remain unknown.

**Objectives:** To evaluate effects of prednisolone on DGGR‐lipase activity and pancreatic lipase immunoreactivity (PLI) in cats.

**Animals:** 7 clinically healthy colony cats, aged 4–7 years, with unremarkable CBC/biochemistry panel.

**Methods:** Prednisolone (1.1–1.5 mg/kg, median 1.28 mg/kg PO) was given daily for 7 consecutive days. DGGR‐lipase activity (LIPC Roche; RI, 8–26 U/L), and PLI (Spec fPL; RI, 0–3.5 μg/L) were determined before first treatment (day 0) and at days 1, 2, 3, 8, 10, 14. Cats were examined daily. A priori power analysis indicated that 6 cats were needed to find a biological relevant effect at 1‐β = 0.8. A Friedman test followed by a *post hoc* Dunn's test was used to compare lipase activities/PLI concentrations at multiple time points with day 0.

**Results:** Median (range) day 0 lipase activities and PLI were 22 U/L (14–52 U/L) and 3.2 μg/L (2.3–15.7 μg/L). One cat with increased lipase activity (52 U/L) and PLI (15.7 μg/L) at day 0 continued having increased lipase activities and PLI throughout the study. Lipase activities and PLI concentrations did not differ significantly between time points regardless of whether the cat with increased values was included or not. All cats remained healthy throughout the study.

**Conclusions and Clinical Importance:** Administration of prednisolone in anti‐inflammatory doses does not significantly increase DGGR‐lipase activity and PLI concentration.

## Abstract GI20: Adverse Events Following Repeat Fecal Microbiota Transplantation in Cats: A Case Series

### **Mary Ann Lee** ^1^; Tanner Slead^2^, DVM, DACVIM (SAIM); M. Katherine Tolbert^3^, DVM, PhD, DACVIM (SAIM); Sina Marsilio^4^, Dr med. vet., PhD, DACVIM (SAIM), DECVIM‐CA

#### ^1^School of Veterinary Medicine, University of California Davis, Davis, CA, USA; ^2^Advanced Urinary Procedures and Extracorporeal Therapies Fellow, NC State Veterinary Hospital, Raleigh, NC, USA; ^3^Clinical Associate Professor, Small Animal Clinical Sciences, Texas A&M University, College Station, TX, USA; ^4^Assistant Professor, Veterinary Medicine and Epidemiology, School of Veterinary Medicine, University of California Davis, Davis, CA, USA

**Background:** Fecal microbiota transplantation (FMT) is a growing therapeutic modality in companion animals with gastrointestinal diseases. Information on adverse events (AE) following FMTs in companion animals are scarce. However, serious AE have anecdotally been reported in cats with gastrointestinal diseases following repeat FMTs.

**Objective:** Describe the AE of FMT in a series of cats with underlying gastrointestinal disease receiving multiple FMTs.

**Animals:** Nine cats receiving at least two FMTs for acute or chronic gastrointestinal disease.

**Methods:** Retrospective multicenter case series. AE were graded according to the Veterinary Cooperative Oncology Group‐Common Terminology Criteria for Adverse Events (VCOG‐CTCAE v2) grading system.

**Results:** Four adult cats (median 10 years of age, range 7–14 years) and five kittens (all aged 2 months) received repeat FMTs (median 3 FMTs, range 2–6) at 5 g/kg (*n =* 7) or 6 g/kg (*n =* 2) via a rectal enema. Reasons for receiving FMT were therapy‐resistant diarrhea (5 kittens) and chronic enteropathy (4 cats). AE included lethargy (*n =* 7), vomiting (*n =* 5), watery diarrhea (*n =* 5), inappetence (*n =* 5), fever (*n =* 4), and abdominal pain (*n =* 2). AE after FMTs were categorized as grade III (*n =* 2), grade II (*n =* 5), and grade I (*n =* 2). Cats showed recovery with metronidazole and anthelmintics (*n =* 4), gabapentin (*n =* 2), marbofloxacin and anthelmintics (*n =* 1), pradofloxacin (*n =* 1), and self resolvement (*n =* 1).

**Conclusions and Clinical Importance:** Repeat FMT in cats with gastrointestinal disease may cause clinically relevant AE, of which clinicians should be aware. Prospective systematic studies evaluating AE after FMT administration in cats are needed.

## Abstract GI21: Assessment of Safety, Adverse Events, and Dysbiosis in Healthy Dogs Receiving Fecal Microbiota Transplantation

### **Mary Ann Lee** ^1^; Maria Questa^1^, PhD; Bart Weimer^2^, PhD; Angel Avalos^1^, BS; Bridget McLaughlin^3^; Agostino Buono^4^, DVM; Jan Suchodolski^5^, MedVet, DrVetMed, PhD, AGAF, DACVM; Sina Marsilio^6^, Dr med. vet., PhD, DACVIM (SAIM), DECVIM‐CA

#### ^1^School of Veterinary Medicine, University of California Davis, Davis, CA, USA; ^2^Chair & Professor, Population Health & Reproduction, School of Veterinary Medicine, University of California Davis, Davis, CA, USA; ^3^Director, Flow Cytometry Shared Resource, University of California Davis, Davis, CA, USA; ^4^Small Animal Internal Medicine Resident & PhD Candidate, Department of Small Animal Clinical Sciences, College of Veterinary Medicine, Texas A&M University, College Station, TX, USA; ^5^Associate Director for Research, Gastrointestinal Laboratory, Texas A&M University, College Station, TX, USA; ^6^Assistant Professor, Veterinary Medicine and Epidemiology, School of Veterinary Medicine, University of California Davis, Davis, CA, USA

**Background:** Fecal microbiota transplantation (FMT) is a growing therapeutic modality in gastrointestinal and extra‐gastrointestinal diseases. No experimental canine studies on safety have been conducted and adverse events (AE) are difficult to differentiate from primary disease exacerbation in patients with pre‐existing disease.

**Hypothesis/Objectives:** To determine the safety and AE of FMT in dogs.

**Animals:** Ten clinically healthy, client‐owned dogs.

**Methods:** Prospective pilot study using a single rectal enema of 5 g/kg. Owners recorded AE using a modified canine IBD activity index and fecal scores based on the Nestlé Purina fecal scoring system. Stool and blood samples were collected at days 0 (baseline), 1, 4, 10, and 28 following FMT. Dysbiosis index was calculated via abundance of 9 bacteria taxa using qPCR. AE were graded using the Veterinary Cooperative Oncology Group‐Common Terminology Criteria for Adverse Events (VCOG‐CTCAE v2). A repeated‐measures mixed‐effects analysis was used.

**Results:** Complete blood count, serum biochemistry, C‐Reactive Protein, serum cytokines IL‐2, IL‐6, IL‐8, and TNF‐α, peripheral lymphocytes (CD79a+, CD3+, CD8+, CD4+, FOXp3+), and fecal score before and after FMT did not significantly change. Owners of 9/10 dogs reported self‐resolving minor clinical signs of vomiting (*n =* 4), diarrhea (*n =* 3), and lethargy (*n =* 2), all categorized as grade I. Dysbiosis index did not differ significantly from baseline following FMT (*P* = 0.18).

**Conclusions and Clinical Importance:** Data from this pilot study supports the safety of FMT administration in dogs, as FMT did not induce dysbiosis and had minimal AE.

## Abstract GI22: Western Diet Promotes Bile Acid Dysmetabolism, Pro‐carcinogenic Inflammation, and Colonic Dysbiosis in Healthy, Non‐obese Dogs

### **Brandon L. Mason** ^1^; Dipak Sahoo^1^; Chelsea Iennarella‐Servantez^1^; Shannon Morgan^1^; Agnes Bourgois‐Mochel^1^; Alex Bray^1^; Jan Suchodolski^2^; Mark Ackermann^1^; Jonathan Mochel^1^; Karin Allenspach^1^; Albert Jergens^1^

#### ^1^College of Veterinary Medicine, Iowa State University, Ames, IA, USA; ^2^College of Veterinary Medicine, Texas A&M University, College Station, TX, USA

**Background:** Metabolic dysfunction secondary to high‐fat diet consumption may seriously diminish quality of life and shorten the lifespan in dogs and humans, alike. Diets high in fat increase the secretion of bile acids (BA), promote microbial imbalances, and drive chronic intestinal inflammation.

**Hypothesis:** A high‐fat, low‐fiber Western diet (WD) fed to healthy, non‐obese dogs disrupts BA metabolism and promotes microbial imbalances, altered apoptosis, and pro‐carcinogenic inflammation.

**Animals:** Eight healthy, laboratory‐reared, beagle dogs.

**Methods:** A dietary trial compared the effects of a control diet (CD) formulated on recommended maintenance macronutrient and fiber levels to a high‐fat, high‐monosaccharide, and low‐fiber WD. The study had three consecutive feeding periods: (1) five weeks of CD, followed by (2) seven weeks of WD and (3) four weeks of CD (recovery). Outcome measures included molecular/microbiologic testing of colonic biopsies, intestinal histopathology, and fecal BAs at the completion of each feeding period. Statistical analysis was performed using one‐way ANOVA with significance defined as *P* < 0.05.

**Results:** Physiological parameters suggestive of metabolic dysfunction were observed in dogs fed WD. Cell markers of apoptosis (TUNEL) and inflammation (NF‐ĸB) as well as serum high‐sensitivity C‐reactive protein were increased (*P* < 0.05) in dogs fed WD vs. CD. Other perturbations seen with WD ingestion included altered (*P* < 0.05) mucosal populations of *Bacteroides*, *Clostridia*, and *Enterobacteriaceae* and increased (*P* < 0.05) levels of fecal cholic acid and serum myeloperoxidase.

**Conclusions:** High‐fat diet fed to healthy dogs promotes microbial imbalances, disturbed host metabolism, and pro‐carcinogenic inflammation.

## Abstract GI23: Prognostic Value of C‐Reactive Protein in Dogs With Elevated Pancreas‐Specific Lipase Reactivity

### **Sydney M. Oberholtzer** ^1^; Audrey Cook^1^, BVM&S, MSc Vet Ed, MRCVS, DACVIM, DECVIM, DABVP (Feline Practice); Robynne Gomez^2^, M.S.; Jörg Steiner^2^, MedVet, DrMedVet, PhD, DACVIM, DECVIM‐CA, AGAF

#### ^1^Veterinary Medical Teaching Hospital, Texas A&M University, College Station, TX, USA; ^2^Gastrointestinal Laboratory, Texas A&M University, College Station, TX, USA

**Background:** C‐reactive protein (CRP) is frequently elevated in dogs with pancreatitis, but its prognostic value is unclear.

**Objective:** Determine the prognostic value of CRP in dogs with pancreatitis.

**Animals:** 503 client owned animals with pancreatic lipase immunoreactivity (PLI) >600 ug/L.

**Methods:** Prospective study. Routine canine submissions to Texas A&M Gastrointestinal Laboratory were monitored for samples with PLI >600 μg/L. Clinics were emailed 14–15 days after PLI measurement and asked: (1) Was the dog hospitalized? and (2) Is the patient alive? If a response was received, CRP was measured using banked serum.

**Results:** Paired measurements were available for 503 dogs. Median PLI was 984 ug/L (range: 603–2001); median CRP was 9.9 mg/L (range: 9.9–395.3; ref: <10 mg/L). There was modest positive correlation between PLI and CRP (*r =* 0.2; CI: 0.12–0.29). In‐patient care was provided to 136 dogs (27%); 49 dogs (9.7%) died. Median CRP was higher for hospitalized dogs (36.1 v 9.9 mg/L; *P* < 0.0001) and those that died (37.2 v 9.9 mg/L; *P* < 0.0001) (Figure 1). Compared to dogs with CRP <10 mg/L, those with CRP >10 mg/L were 5.3 times more likely to die (CI: 2.7–10.2) and 5.7 (CI: 3.6–8.7) times more likely to be hospitalized. The odds ratio of death for dogs with CRP >30 mg/L was 6.9 (CI: 3.5–13.9) (Figure 2).

**Conclusions:** In dogs with PLI >600 ug/L, CRP >10 mg/L was associated with increased risk of hospitalization or death. This biomarker may provide useful prognostic information in dogs with clinical evidence of pancreatitis.

**Figure:**
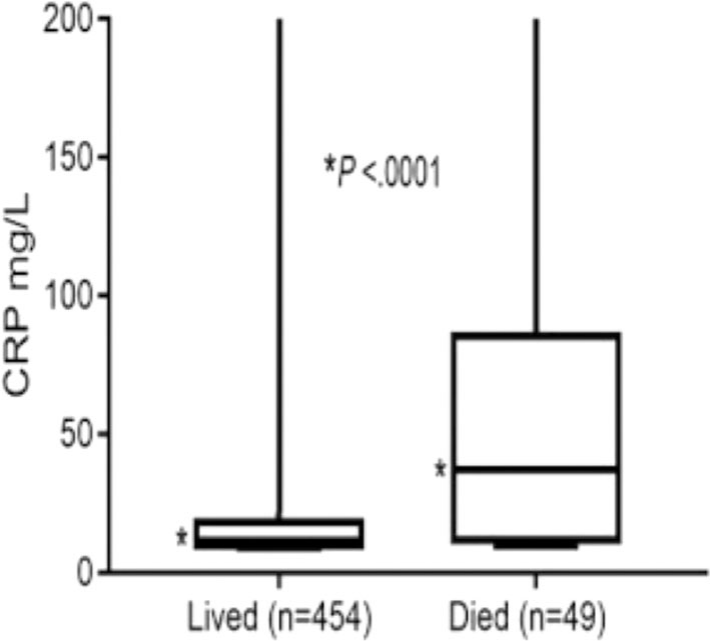
Figure 1. Box and whiskers plot of CRP results for dogs with PLI >600 ug/L; CRP values >200 mg/L not shown

**Figure:**
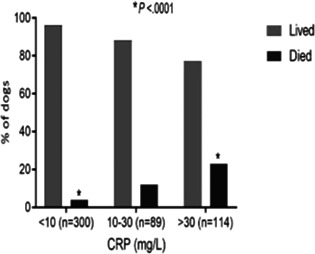
Figure 2. Percentage of dogs that lived vs. died with CRP <10 mg/L, 10–30 mg/L and >30 mg/L

## Abstract GI24: A Retrospective Evaluation of Serum Symmetric Dimethylarginine Concentration in Dogs With Protein Losing Enteropathy

### **Yeon Joon Park** ^1^; Alexander German^2^, BVSc PhD CertSAM DECVIM‐CA SFHEA FRCVS; David Brewer^3^; Erin O’Connell^4^, BVSc DECVIM‐CA FHEA

#### ^1^University of Liverpool, Liverpool, UK; ^2^Royal Canin Professor of Small Animal Medicine, Institute of Life Course & Medical Sciences and School of Veterinary Science, University of Liverpool, Liverpool, UK; ^3^Post Residency Clinician in Oncology, Dick White Referrals, Cambridgeshire, UK; ^4^Lecturer in Small Animal Internal Medicine, University of Liverpool, Liverpool, UK

**Background:** Serum symmetric dimethylarginine (SDMA) is produced by all nucleated cells and is increased in people with inflammatory bowel disease (IBD). Changes in dogs with protein‐losing enteropathy (PLE) have not been assessed.

**Objectives/Hypothesis:** To evaluate SDMA concentration in non‐azotemic dogs with PLE.

**Animals:** 63 client owned dogs: 21 with PLE and 42 age/breed/sex/neuter status‐matched controls.

**Methods:** The clinical records of a referral hospital in the United Kingdom were retrospectively reviewed over a 2‐year period. Dogs with azotemia or prior glucocorticoid/immunosuppressive therapy were excluded. Dogs diagnosed with PLE that had SDMA measured were compared with the matched controls. Signalment, clinical presentation, clinicopathological abnormalities, treatment, and SDMA concentration pre‐ (PLE‐T0) and post‐ (PLE‐T1) treatment were recorded.

**Results:** At baseline, SDMA concentration was greater in PLE (T0 14.4 ± 3.09 μg/dL) than control (11.3 ± 3.17 μg/dL) dogs (*P* < 0.001, Hedge's G 0.98), but decreased with treatment (PLE‐T1: 10.1 ± 2.73 μg/dL; T0 vs. T1: *P* = 0.003, Hedge's G 1.14). Creatinine concentration was similar in PLE (T0 68 ± 22.4 μg/dL) and control (77 ± 21.3 μg/dL) dogs at baseline (*P* = 0.122, Hedge's G 0.41). Albumin concentration was less in PLE (17.0 ± 5.56 g/L) than control (29.5 ± 5.12 g/L) dogs (*P* < 0.001, Hedge's G 2.33) before treatment, but increased with treatment (PLE‐T1: 23.3 ± 6.34 g/L; T0 vs. T1: *P* = 0.001, Hedge's G 1.09), although remained less than the concentration in controls (*P* = 0.001, Hedge's G 1.14). No other clinicopathological differences were evident.

**Conclusions and Clinical Importance:** Similar to people with IBD, SDMA may be increased in dogs with PLE, the clinical significance of which requires further investigation.

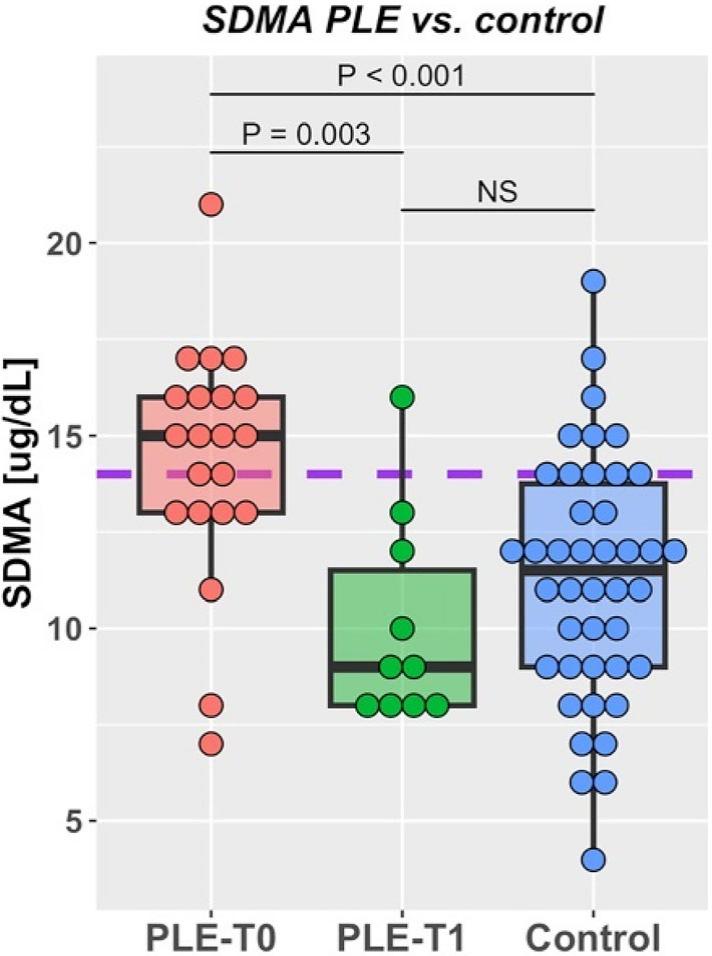

Comparison of serum symmetric dimethylarginine (SDMA) concentrations in dogs with protein losing enteropathy before (PLE‐T0) and after (PLE‐T1) treatment, and control dogs.

## Abstract GI25: Possible Role of Dietary N‐glycolyneuraminic Acid and Dysbiosis in Canine Enteropathy Pathogenesis

### **Giacomo Rossi** ^1^; Lucia Biagini, DVM^1^; Sara Ricci^2^, DVM, PhD; Daniela Oliviero^3^; Livio Galosi^1^; Sinem Gultekin^1^; Matteo Cerquetella^1^; Alessandra Gavazza^1^; Barbara Simionati^4^; Ilaria Patuzzi^4^; Fiorella Carnevali^5^

#### ^1^University of Camerino, Camerino, Italy; ^2^University of Veterinary Medicine Vienna, Vienna, Austria; ^3^Biessea, Milan, Italy; ^4^EUBIOME, Padova, Italia; ^5^ENEA, Santa Maria di Galeria, Italy

**Background:** N‐glycolylneuraminic acid (Neu5Gc) is synthesized from its N‐acetyl precursor (Neu5Ac) by cytidine‐5′‐monophospho‐N acetylneuraminic acid hydroxylase (CMAH). Absent in humans and ferrets, it is polymorphic in dogs. Loss of the CMAH gene generate a change in the structural profile of glycans of all tissues inducing the production of antibodies against Neu5Gc‐glycans.

**Hypothesis/Objectives:** Prolonged uptake of Neu5Gc by negative‐CMAH dog through red meat and dairy products from +CMAH mammals leads to a progressive Neu5Gc‐glycans incorporation in host's tissue (xenosialization), particularly if the gut microbiota is altered in de‐sialilating bacteria determining an inflammatory reaction (“Xenosialitis”).

**Animals:** For immunohistochemistry, gastro‐entero‐colic biopsies from archive material belonging to European, Asian, and American breeds were analyzed (35 dogs per group). Also, the fecal microbiota of 2 cohorts (127+167) of healthy and enteropathic dogs was evaluated.

**Methods:** We chose a polyclonal antibody (Creative Diagnostic, DMABH‐C003) for Neu5GC expression. The distribution of desializing bacteria was performed using two different sequencing techniques for different regions of the 16S rRNA gene.

**Results:** Neu5Gc resulted mainly expressed in colon of dogs with enteropathy (*P* < 0.005) with no relation to breed. Greater prevalence of Clostridiales and Bactroidales was observed in enteropathic dogs.

**Conclusions and Clinical Importance:** Dysbiosis with increase Clostridiales and Bacteroidales could predispose to xenosialization and intestinal inflammation in negative‐CMAH dogs, with a possible greater desializing activity compared to healthy dogs. Increased Neu5GC could cause greater uptake of xenosialoantigens by enteropathic dogs since Bifidobacteria, known for their cross‐feeding of sialic acids activity, did not differ between healthy and pathologic.

**Figure:**
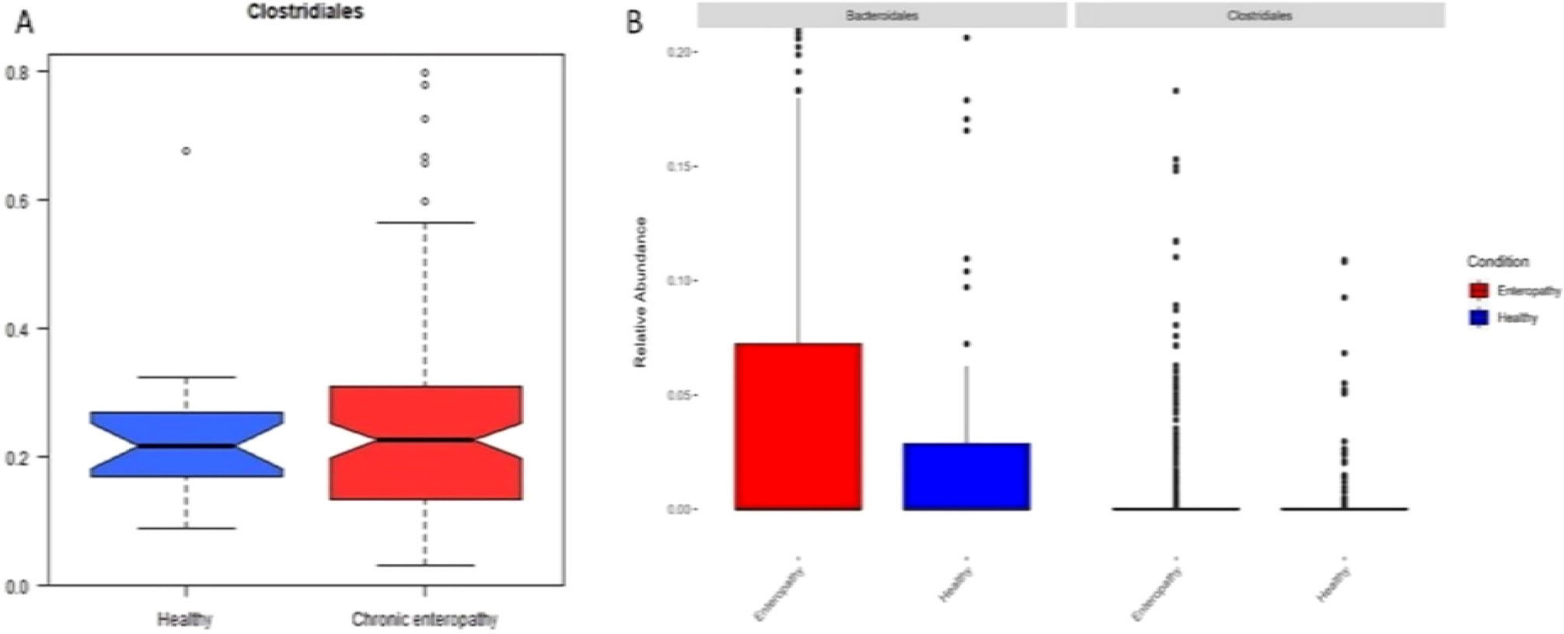
Figure 1. Fecal microbiota of dogs: (A) relative abundance of Clostridiales (sequencing of regions V3‐V4); (B) relative abundance of Clostridiales and Bacteroidales (sequencing of regions V2‐V9).

**Figure:**
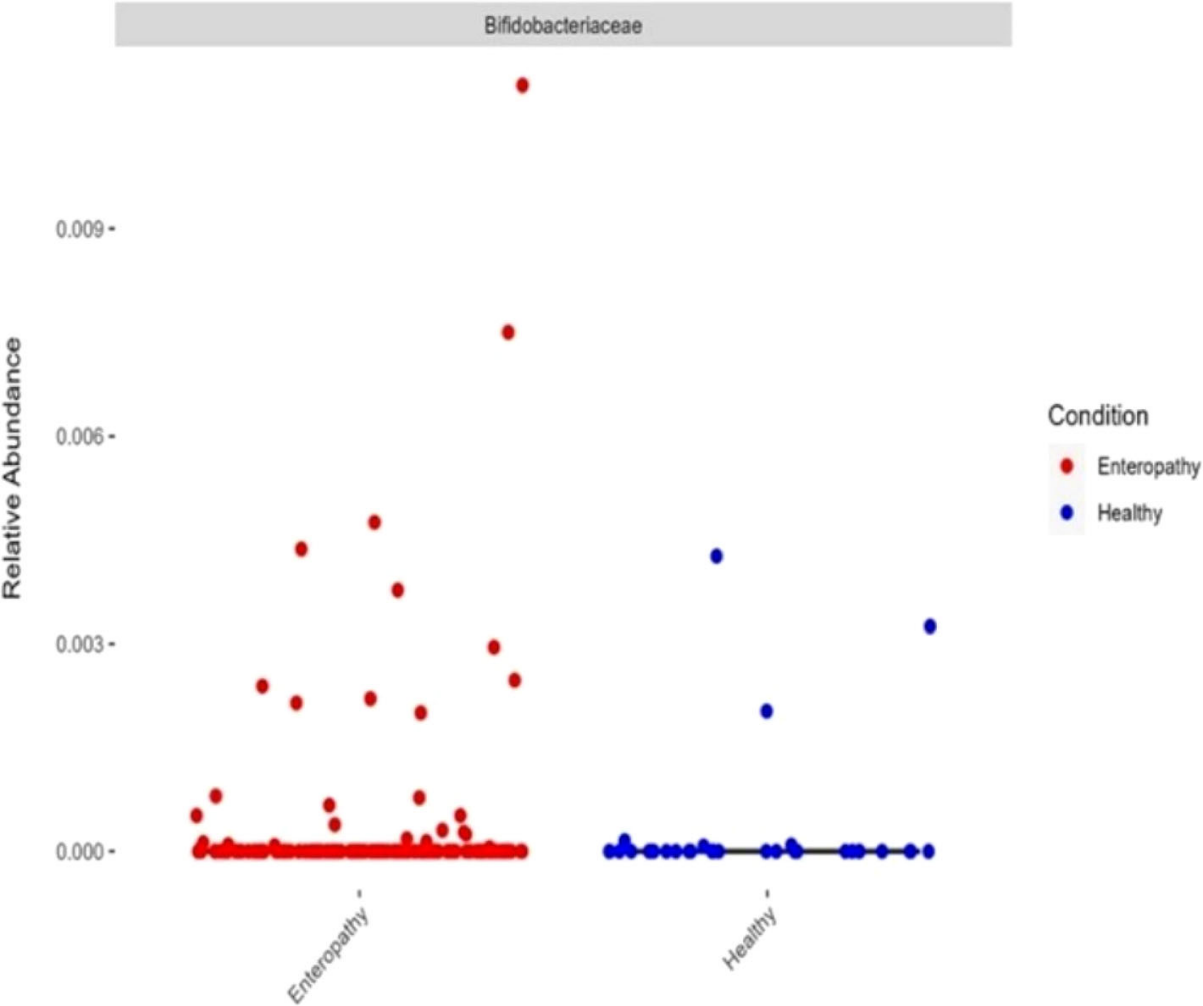
Figure 2. Relative abundance of Bifidobacteriaceae (sequencing of regions V2‐V9).

## Abstract GI26: Blood Microbiome in Dogs With Chronic Enteropathies: The Future of Prevention and Diagnosis?

### **Elisa Scarsella** ^1^; Guillaume Jospin^2^, MSc; Niokhor Dione^3^, DVM, PhD; Holly Ganz^4^, PhD

#### ^1^AnimalBiome, Oakland, CA, USA; ^2^Bioinformatician, AnimalBiome, Oakland, CA, USA; ^3^Senior Director of Microbial Discovery, AnimalBiome, Oakland, CA, USA; ^4^Chief Science Officer, AnimalBiome, Oakland, CA, USA

**Background:** The presence of bacteria in the blood has been often associated with an infection. However, recent studies have found bacterial DNA in the blood of healthy subjects. Currently, several hypotheses support the existence of a blood microbiome also in healthy hosts.

**Objectives:** The aim of this study is to characterize the blood microbiome of healthy dogs and dogs with chronic enteropathies (CE), and to correlate targeted microorganisms observed in blood and stool samples with ongoing disease. The final purpose is to assess a list of detectable blood bacteria correlated with different levels of the gut inflammation, that are possible to evaluate during a chronic pathology.

**Animals:** 17 healthy dogs and 19 CE dogs.

**Methods:** Blood and fecal samples were collected from healthy and diseased dogs and analyzed for the full 16S rRNA gene, though PacBio long‐read high‐throughput sequencing.

**Results:** Alpha and beta diversities of fecal microbiome were significantly different between the two groups of dogs. Principal components analysis revealed that healthy and sick subjects were significantly clustered, for both blood and fecal microbiome samples. Some of the taxa shared between blood and stool samples were further analyzed through quantitative Real Time PCR, in order to get the true picture of their abundances. Further studies are needed to confirm the origin of the blood microbiome.

**Conclusions:** The characterization of a core microbiome in the blood of domestic dogs has potential for use as a diagnostic tool to monitor for the development of gastro‐intestinal disease.

## Abstract GI27: Occurrence of Owner‐Reported Gastrointestinal Disease in 33,172 Dogs in the Dog Aging Project Pack

### **Sarah M. Schmid** ^1^; Jessica Hoffman^2^, PhD; Emily Gould^3^, DVM, MS, DACVIM (SAIM), PhD; Alaina Moon^4^, DVM, DACVIM (SAIM); Kate Creevy^5^, DVM, MS, DACVIM (SAIM)

#### ^1^University of Tennessee, Knoxville, TN, USA; ^2^Assistant Professor, Augusta University, Augusta, GA, USA; ^3^Assistant Professor, College of Veterinary Medicine & Biomedical Sciences, Texas A&M University, College Station, TX, USA; ^4^Veterinary Internist, Olympia Veterinary Specialists, Olympia, WA, USA; ^5^Professor, Texas A&M University, College Station, TX, USA

**Background:** The prevalence of gastrointestinal (GI) disorders in the general owned USA dog population is unknown.

**Objective:** To determine the occurrence and demographic features of GI disease in dogs enrolled in the Dog Aging Project (DAP) Pack.

**animals.**

33,172 dogs

**Methods:** Owner‐reported GI disorders were extracted from Health Life and Experience Survey (HLES) results for dogs enrolled in DAP between December 2019 and December 2021. Potential risk factors for various GI diseases were identified by means of multivariate logistic regression analysis. Age at diagnosis was used for dogs with GI diseases. Age at the time of HLES completion was used for control dogs.

**Results:** A total of 13,646 occurrences of GI disorders were reported with 29.2% (9,679) of dogs in the DAP Pack having at least one GI disorder reported. The most commonly reported disorders were infectious diseases (5344, 16.1% of DAP Pack), chronic diarrhea (954, 2.9%), anal sac impactions (829, 2.5%), foreign bodies (795, 2.4%), pancreatitis (680, 2.0%), chronic vomiting (389, 1.2%), acute hemorrhagic diarrhea syndrome (292, 0.9%), and inflammatory bowel disease (406, 1.2%). Dogs with GI disorders were more likely to be neutered (2.427, *P* < 0.0001), to have a history of being malnourished or underweight (1.598, *P* < 0.0001), to have received probiotics or over‐the‐counter GI medications (1.582 and 3.319, respectively, *P* < 0.0001), or to have a history of being fed primarily canned food (2.388, *P* < 0.0001).

**Clinical Relevance:** GI disorders are common owner‐reported diagnoses among dogs enrolled in the DAP.

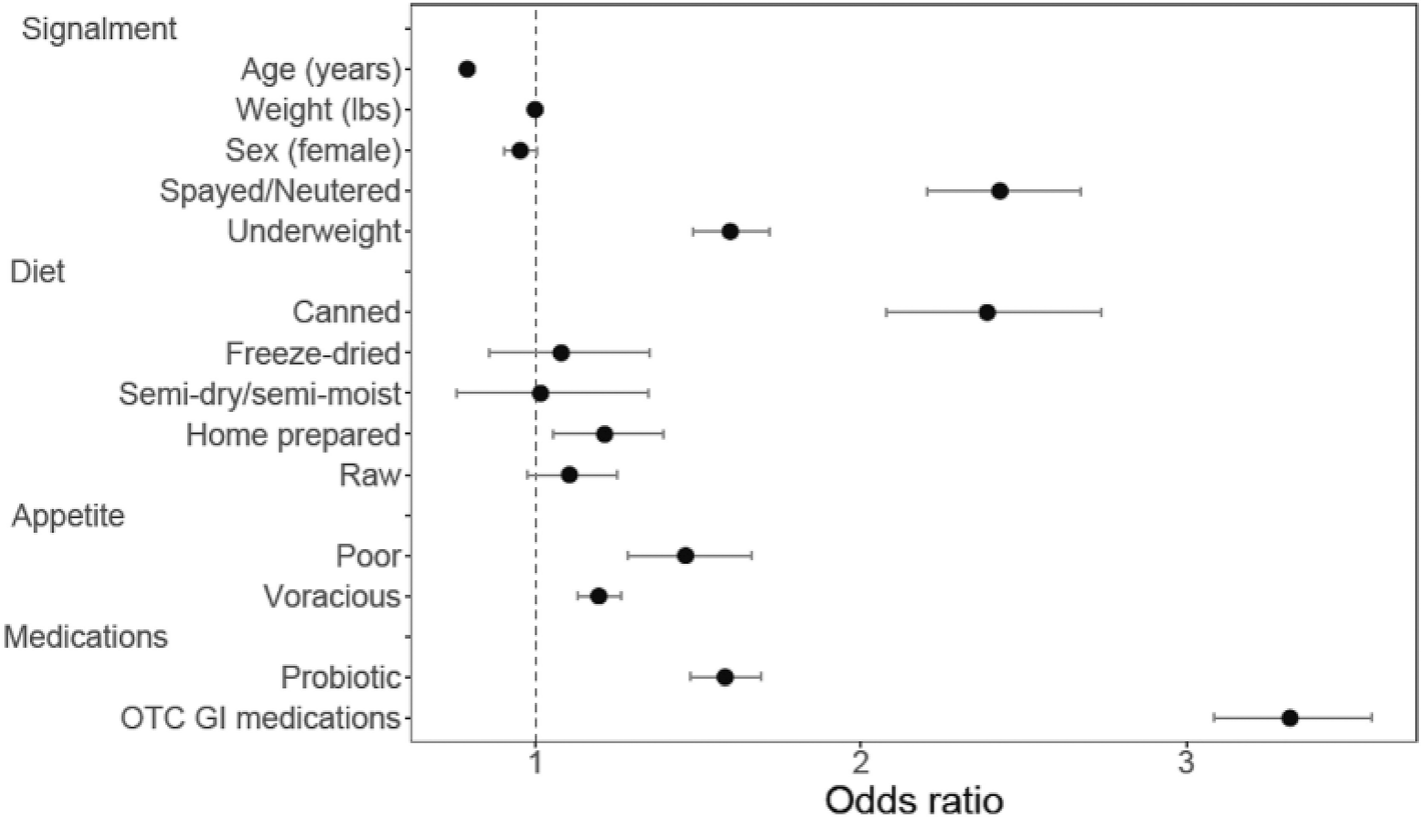

## Abstract GI28: Use of a Feline Dysbiosis Index to Monitor a Probiotic's Effects on Antibiotic‐Induced Dysbiosis

### **Megan Slaughter** ^1^; Jan Suchodoloski^2^; Chi‐Hsuan Sung^2^; Rachel Pilla^2^; Michael Lappin^3^

#### ^1^Colorado State University, Ft. Collins, CO, USA; ^2^College of Veterinary Medicine and Biomedical Sciences, Texas A&M University, College Station, TX, USA; ^3^College of Veterinary Medicine and Biomedical Sciences, Colorado State University, Ft. Collins, CO, USA

**Background:** In a previous study, clinical evidence showed that the probiotic *Enterococcus faecium* strain SF68 could lessen amoxicillin‐clavulanate associated diarrhea (1). A number of microbiome and metabolome assessments were made, but definitive explanations for the apparent clinical benefits were not apparent. Since that work, a new feline dysbiosis index (DI) was developed and further exploration of the effects of the probiotic on antibiotic‐induced dysbiosis is warranted (2).

**Hypothesis/Objectives:** To describe results of the feline DI in cats administered amoxicillin‐clavulanate with or without the probiotic.

**Animals:** 27 healthy young adult research cats.

**Methods:** Cats were randomized into the probiotic (13 cats) or placebo (14 cats) groups (1). The probiotic or placebo was fed 2 h before the morning dose of amoxicillin‐clavulanate with the second dose of antibiotic administered 10 h later. The probiotic or placebo was administered for 14 days and the antibiotic was administered for 7 days. The fecal microbiome was assessed by qPCR for specific bacterial taxa and the feline DI was calculated (2).

**Results:** Both groups developed a significant increase in DI by Day 7 and the DI improved significantly in both groups by Day 14 (Figure 1). However, the placebo group was still significantly increased compared to baseline (*P* = 0.028), while the probiotic group was not (*P* = 0.174).

**Conclusions and Clinical Importance:** The feline DI is useful to assess changes in fecal microbiota during and after the administration of this antibiotic. The probiotic led to faster recovery of the microbiome as assessed by this feline DI.

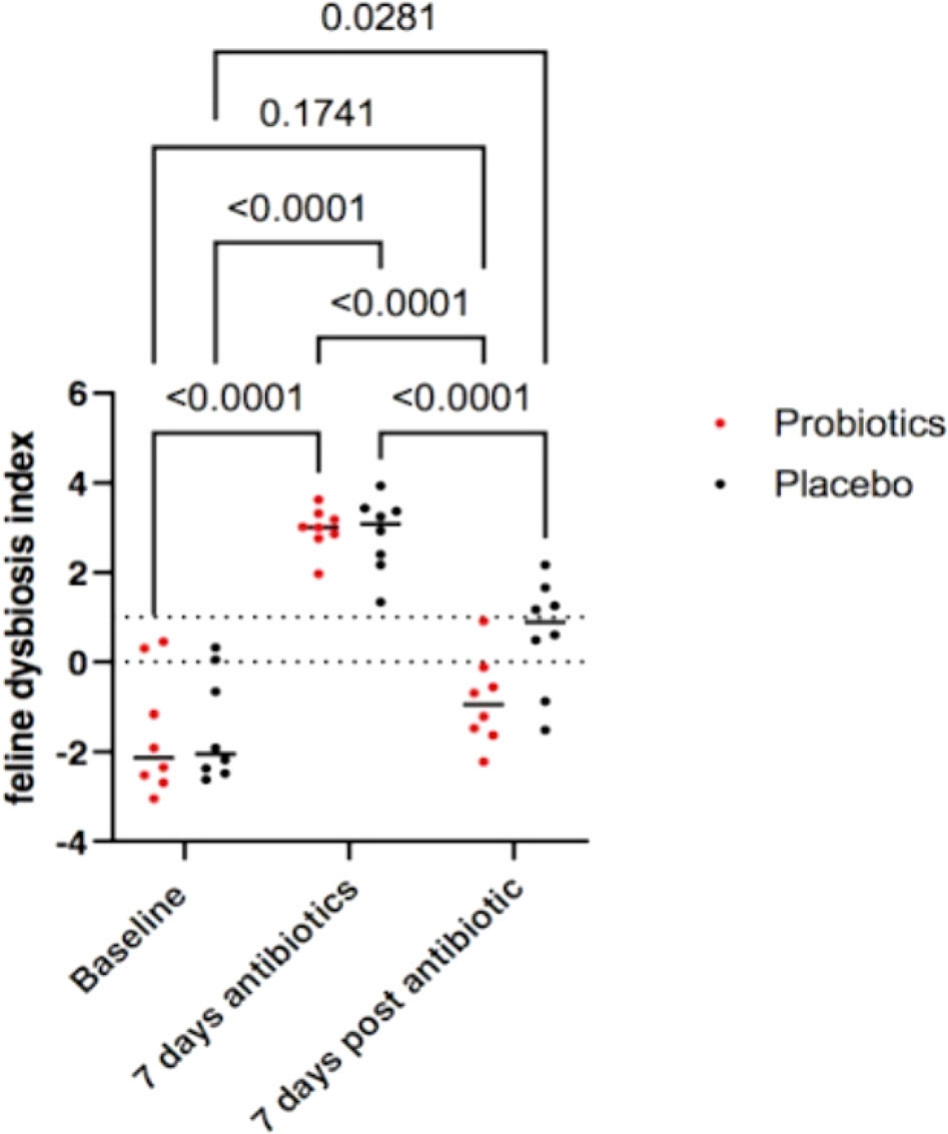

## Abstract GI29: Effect of Wheat Dextrin on Fecal Short‐Chain Fatty Acids in Dogs With Chronic Diarrhea

### **Stacie C. Summers** ^1^; Rachel Pilla^2^; Jan Suchodolski^2^

#### ^1^Oregon State University, Corvallis, OR, USA; ^2^Gastrointestinal Laboratory, Texas A&M University, College Station, TX, USA

**Background:** Wheat dextrin is a soluble fermentable fiber that has *in vitro* effects on the fecal microbiome and short‐chain fatty acid (SCFA) production.

**Objective:** To determine the prebiotic effect of wheat dextrin on major fecal bacterial taxa and SCFA concentrations in dogs with chronic diarrhea.

**Animals:** 17 client‐owned dogs with a history of chronic diarrhea

**Methods:** Randomized, blinded, placebo‐controlled clinical trial. Nine dogs received wheat dextrin powder and 8 dogs received maltodextrin powder (placebo) mixed with their food daily for 4 weeks (>20 kg body weight: 6 teaspoons/day; <20 kg: 3 teaspoons/day). Canine dysbiosis index (DI) was calculated using qPCR abundances of select bacterial taxa, and fecal SCFA concentrations (acetate, butyrate, propionate) were measured via gas chromatography/mass spectrometry before and 4‐weeks after supplementation. Fecal scores were recorded by pet owners for 7 days prior to starting the powder supplement and during the 4‐week supplementation period. Paired t‐test with false discovery rate correction was used for analysis.

**Results:** Dogs in the treatment group were supplemented a median dose of 0.4 g/kg/day of soluble fiber (range, 0.2–0.9 g/kg/day). The fecal DI, abundances of *Faecalibacterium*, *Turicibacter*, *Blautia*, *Fusobacterium*, and SCFA concentrations were not significantly different at week 4 when compared to baseline in either group (Table). Average fecal score was not significantly different after 4‐weeks of supplementation when compared to baseline.

**Conclusion:** Wheat dextrin was well tolerated when supplemented to dogs with chronic diarrhea, however, did not alter the fecal abundance of taxa known to ferment dietary fiber to SCFAs in the colon.

Table: Fecal short‐chain fatty acid concentrations, fecal qPCR abundances of select bacterial taxa, and dysbiosis index (mean and standard deviation) before and after supplementation with wheat dextrin or placebo powder. RI, reference interval

**Table:** 

	Wheat dextrin group (*n =* 9)			Placebo group (*n =* 8)		
	Baseline	Week 4	*P* value	Baseline	Week 4	*P* value
Fecal short‐chain fatty acids (μmol/g of fecal dry matter)						
Acetate	167 ± 67	171 ± 58	0.9	152 ± 111	122 ± 78	0.4
Propionate	103 ± 45	109 ± 34	0.8	73 ± 78	47 ± 49	0.2
Butyrate	35 ± 20	31 ± 22	0.7	28 ± 25	30 ± 31	0.9
Fecal qPCR abundance (Log DNA)						
*Faecalibacterium* (RI: 3.4–8.0)	5.8 ± 1.4	5.8 ± 1.3	1.0	5.0 ± 1.7	4.8 ± 1.5	0.8
*Turicibacter* (RI: 4.6–8.1)	5.6 ± 0.7	5.4 ± 0.6	0.4	5.4 ± 0.8	5.4 ± 0.9	1.0
*Blautia* (RI: 9.5–11.0)	9.9 ± 0.4	9.7 ± 0.3	0.4	9.4 ± 1.1	9.6 ± 0.9	0.7
*Fusobacterium* (RI: 7.0–10.3)	8.8 ± 0.7	8.5 ± 0.7	0.4	7.9 ± 0.8	8.3 ± 1.0	0.3
Dysbiosis index (normal <0)	−2.9 ± 2.7	−2.8 ± 2.5	1.0	1.1 ± 4.4	0.1 ± 4.5	0.4

## Abstract GI30: Correlation Between Targeted qPCR‐Assays and Untargeted DNA Shotgun Sequencing for Assessing Fecal Microbiota in Dogs

### **Chi‐Hsuan Sung** ^1^; Rachel Pilla^1^; Patricia Ishii^1^; Linda Toresson^2^; Karin Allenspach^3^; Albert Jergens^3^; Stacie Summers^4^; Kelly Swanson^5^; Holger Volk^6^; Teresa Schimidt^6^; Helene Stuebing^7^; Johanna Rieder^6^; Kathrin Busch^7^; Melanie Werner^8^; Anja Lisjak^9^; Frederic Gaschen^10^; M. Katherine Tobert^1^; Jonathan Lidbury^1^; Joerg Steiner^1^; Jan Suchodolski^1^

#### ^1^Texas A&M University, College Station, TX, USA; ^2^Evidensia Specialist Animal Hospital, Helsingborg, Sweden; ^3^Iowa State University, Ames, IA, USA; ^4^Oregon State University, Corvallis, OR, USA; ^5^University of Illinois at Urbana‐Champaign, Champaign, IL, USA; ^6^University of Veterinary Medicine, Hannover, Germany; ^7^Ludwig‐Maximilians University, Munich, Germany; ^8^Vetsuisse Faculty, Bern, Switzerland; ^9^University of Ljubljana, Ljubljana, Slovenia; ^10^Louisiana State University, Baton Rouge, LA, USA

**Background:** Shotgun DNA sequencing is an untargeted approach for identifying changes in relative microbiota abundances, while qPCR allows reproducible quantification of specific bacteria. The canine dysbiosis index (DI) is a mathematical model based on the obtained qPCR data designed to evaluate shifts in the global microbiota.

**Objective:** To correlate results between qPCR assays included in the DI and DNA shotgun sequencing.

**Animals:** Fecal samples from 296 dogs (112 healthy, 150 with various enteropathies, 34 dogs with non‐gastrointestinal diseases).

**Methods:** Correlations between relative proportions of bacteria obtained by sequencing or qPCR were evaluated by Spearman tests. Samples were classified into four groups based on the DI interpretation: normal (DI <0 with all evaluated bacterial taxa within the reference interval, RI), minor changes (DI <0 but with at least one bacterial taxa out of RI), mild‐moderate changes (02). Microbiota shifts were evaluated based on Bray‐Curtis distances and statistics were performed with ANOSIM.

**Results:** Significant correlations (*P* < 0.001) were found for all bacterial taxa quantified by qPCR and sequencing. A significantly lower alpha diversity (*P* < 0.001) was found in dogs with DI >2 (median[range]: 148[70–280]) than in dogs with a normal DI (192[89–329]). The DI classification correctly predicted microbiota shifts obtained by sequencing with increasing size effects (higher *R* values indicate larger size effects): minor changes (R =0.19), mild‐moderate changes (*R =* 0.24), and significant dysbiosis (*R =* 0.62), compared to dogs with a normal DI (*P* < 0.001 for all).

**Conclusions and Clinical Importance:** The DI correlates with results obtained by DNA shotgun sequencing in dogs.

## Abstract GI31: Improved Treatment Outcome & Reduced Recovery Time in Faecal Microbiota Transplanted Dogs With Parvovirus Disease

### **Nashwa E. Waly** ^1^; Rehab Sayed^2^, PhD; Maha Hamed^3^, PhD; Laila Ahmed^4^

#### ^1^Assiut University, Assiut, Egypt; ^2^Research Fellow, Animal Medicine, Assiut University, Assiut, Egypt; ^3^Professor of Infectious Diseases, Animal Medicine, Assiut University, Assiut, Egypt; ^4^Professor Emeritus of Veterinary Infectious Diseases, Animal Medicine, Assiut University, Assiut, Egypt

**Background:** Canine‐parvovirus (CPV) is a highly contagious disease of dogs that causes acute gastrointestinal illness. Treatment is supportive and symptomatic. Fecal microbiota transplantation (FMT) is method where stool of a healthy donor is transplanted into the intestinal tract of a diseased recipient with promising results.

**Objectives:** To examine FMT as an adjunct to treatment of CPV. The potential causes of variability in outcome of this procedure were evaluated.

**Animals:** Dogs with confirmed CPV were recruited (*n =* 50). Blood for CBC, and stool, for genetic characterization, were collected.

**Methods:** Cases were assigned to a conventional treatment group (CT) (*n =* 25) and another that received FMT+CT (*n =* 25). Outcome, days to symptom‐resolution, and days to complete recovery was recorded. Data were assessed for normality; parametric/non‐parametric tests were applied accordingly. Rotated component matrix analysis was conducted to detect any cumulative effects of variables.

**Results:** Presence of two or more risk factors increased the susceptibility to infection (*P* < 0.05). Young age and large breeds were over‐represented. Three antigenic variants (CPV‐2a, CPV‐2b, and CPV‐2c) were isolated.

For the CT group, 13 (52%) cases died; 12 cases (48%) took on average 4 days to resolution of symptoms and 5 to achieve full recovery (5.33 ± 1.97). For the FMT group, 3 cases died (12%); 22 cases (88%) took on average 2 days to symptom resolution and 3 to recover (2.86 ± 0.64). The difference between groups was significant (*P* < 0.001).

**Conclusions:** FMT is an effective adjunct to treatment for CPV with significant reduction in time required for treatment. Hospitalization of dogs isn’t required.

**Table:** Statistical analysis of time required for signs resolution and outcome in each group

Parameter	Groups		
	Conventional	FMT	*P* value
*Days to resolution of vomiting			
Mean ± SD	3.50 ± 1.73	1.41 ± 0.50	<0.001**
Median (Range)	3.0 (2.0–8.0)	1.0 (1.0–2.0)	
*Days to resolution of diarrhea			
Mean ± SD	4.67 ± 1.92	2.32 ± 0.84	<0.001**
Median (Range)	4.5 (2.0–9.0)	2.0 (1.0–4.0)	
*Days to complete recovery			
Mean ± SD	5.33 ± 1.97	2.86 ± 0.64	<0.001**
Median (Range)	5.5 (3.0–10.0)	3.0 (2.0–4.0)	
Outcome:			
Recovered	*n =* 12 (48%)	*n =* 22 (88)	0.002**
Died	*n =* 13 (52%)	*n =* 3 (12%)	

Recruited dogs assigned to one of two treatment groups; Conventional group received only the conventional treatment while FMT group received FMT+ conventional treatment.

* Three set parameters were recorded to help identify the success or failure of the treatment. The level of significance was set at 0.05.

## Abstract GI32: Immunohistochemical Characterization of Lymphocytic Infiltrates in Pancreata from Shelter Cats

### **Yu‐An Wu** ^1^; Shelley Newman^2^; Jonathan Lidbury^3^; Jörg Steiner^3^

#### ^1^Gastrointestinal Laboratory, Texas A&M University, College Station, TX, USA; ^2^Newman Specialty VetPath, Hicksville, NY, USA; ^3^Gastrointestinal Laboratory, Texas A&M University, College Station, TX, USA

**Background:** The phenotype and extent of lymphocytes infiltrating the feline pancreas are not well‐described.

**Objective:** To characterize lymphocytes infiltrating the pancreas from shelter cats.

**Animals:** Forty‐eight cats euthanized for population control.

**Methods:** Pancreata were collected within 1.5 h of death, sectioned every 1 cm, and H&E‐stained slides were prepared for histologic assessment. Sections containing lymphocytes were recut and stained with antibodies against CD3 and CD20 antigens. Distribution of T or B lymphocytes were recorded. When at least one cluster (group of at least 5 lymphocytes easily identifiable at low power) of lymphocytes was present, the percentage of T and B lymphocytes was estimated.

**Results:** Out of 48 cats, 13 (27%) showed clusters of lymphocytes in at least one H&E‐stained section, 7 of which had cluster(s) of lymphocytes present in the recut sections stained with CD3 or CD20 antibodies. For the 39 sections with lymphocytic clusters on H&E‐stained slides, 19 had clusters of T lymphocytes (median: 0%, range: 0%–50%), 16 of which were associated with clusters of B lymphocytes (median: 0%, range: 0%–75%). However, isolated T lymphocytes were commonly identified throughout the pancreatic parenchyma in many sections. T lymphocytes were most commonly periductal (85%), parenchymal (85%), or interstitial (67%); B lymphocytes were most commonly periductal (82%), interstitial (41%), or parenchymal (18%).

**Conclusions:** Clusters of lymphocytes were infrequently identified and were highly localized. These clusters contained both T‐ and B‐lymphocytes in most cases and were mostly periductal. The clinical significance of these lymphocytic clusters and of isolated T lymphocytes is unknown.

## Abstract GI33: Plasma Glucagon‐Like Peptide‐2 in Dogs With Chronic Enteropathies

### **Michelle D. Riehm** ^1^; Erin Mayhue^1^, BS, DVM Candidate Class of 2025; Maria Jugan^2^, DVM, MS, DACVIM (SAIM)

#### ^1^College of Veterinary Medicine, Kansas State University, Manhattan, KS, USA; ^2^Department of Clinical Sciences, College of Veterinary Medicine, Kansas State University, Manhattan, KS, USA

**Background:** Glucagon‐like peptide‐2 (GLP‐2) is an enteroendocrine hormone that promotes gastrointestinal mucosal growth and maintains the mucosal barrier. In humans with chronic gastrointestinal disease, mucosal pathology affects GLP‐2 secretion, which normalizes with disease remission.

**Hypothesis/Objectives:** Fasting and post‐prandial plasma GLP‐2 will be lower in dogs with chronic enteropathies (CE) compared to healthy dogs, and GLP‐2 will increase with clinical disease response.

**Animals:** 18 client‐owned dogs with uncontrolled CE, prior to directed gastrointestinal disease treatment; 17 client‐owned healthy control dogs.

**Methods:** This was a prospective study comparing plasma GLP‐2 in dogs with uncontrolled CE to healthy dogs. Fasted, 1‐, and 3‐h post‐prandial blood samples were collected into chilled EDTA tubes with proteinase inhibitors. Plasma GLP‐2 concentration was measured using a commercial canine ELISA. Procedures were repeated in CE dogs following 30 days of non‐standardized gastrointestinal disease therapy. Mixed effects models with repeated measures were used to assess effect of study day and dog group on GLP‐2 concentrations.

**Results:** Fasted and post‐prandial GLP‐2 concentrations were lower in uncontrolled CE dogs at enrollment (fasted, 424 ± 176 pg/mL; *P* < 0.0001) and follow‐up (fasted, 624 ± 314 pg/mL; *P* < 0.001) compared to healthy dogs (fasted, 1184 ± 435 pg/mL). One‐hour post‐prandial GLP‐2 concentrations were higher at recheck (720 ± 247 pg/mL) compared to enrollment (439 ± 169 pg/mL) in CE dogs (*P* = 0.02).

**Conclusions:** There is disrupted GLP‐2 secretion in dogs with CE. Treatment for gastrointestinal disease may normalize GLP‐2 secretion.

## Abstract GI34: Serum Vitamin D, Its Receptor, and Binding Protein Concentrations in Dogs With Acute Pancreatitis

### **Dohee Lee** ^1^; Taesik Yun^2^; Yoonhoi Koo^2^; Yeon Chae^2^; Byeong‐Teck Kang^2^; Mhan‐Pyo Yang^2^; Hakhyun Kim^2^

#### ^1^College of Veterinary Medicine, Chungbuk National University, Cheongju, Korea; ^2^Laboratory of Veterinary Internal Medicine, College of Veterinary Medicine, Chungbuk National University, Cheongju, Korea

**Background:** Previous studies have documented vitamin D insufficiency in dogs with acute pancreatitis (AP), but no studies have investigated serum vitamin D receptor (VDR) and vitamin D binding protein (VDBP) concentrations.

**Hypothesis/Objectives:** To compare the serum 25‐hydroxyvitamin D (25(OH)D), VDR, and VDBP concentrations in healthy dogs and dogs with AP and to identify the correlations of the concentrations with C‐reactive protein (CRP) and canine‐specific pancreatic lipase (Spec cPL) levels.

**Animals:** Twenty‐two dogs with AP and 20 healthy control dogs.

**Methods:** In this cross‐sectional study, serum 25(OH)D concentrations were measured using a chemiluminescence immunoassay, and VDR and VDBP were measured using a canine ELISA kit.

**Results:** Serum 25(OH)D concentrations were lower in dogs with AP (mean  ±  standard deviation, 66.1 ± 39.2 ng/mL) than in controls (96.8 ± 30.4 ng/mL; *P* = 0.01), and VDR concentration was lower in dogs with AP (5.3 ± 3.5 ng/mL) than in controls (7.4 ± 2.5 ng/mL; *P* = 0.03). No difference was observed in serum VDBP between the groups. Serum VDR concentrations differed between survivors (median [interquartile range] = 6.6 [4.3–8.2] ng/mL) and non‐survivors (2.7 [0.5–3.5] ng/mL; *P* = 0.01). Negative correlations were observed between serum VDR and CRP (rs = −0.55) and between serum VDR and Spec cPL (rs = −0.47) in dogs with AP.

**Conclusions and Clinical Importance:** Serum 25(OH)D and VDR concentrations decreased in dogs with AP, and VDR was associated with clinical outcomes. This study suggests a potential role of VDR expression in the inflammatory process of AP in dogs.

## Abstract GI35: The Risk of Hypomagnesemia With Long‐Term Use of Proton‐Pump Inhibitors in Dogs

### **Jeongmin Lee** ^1^,DVM; Ji‐Woong Her^4^, DVM, MS, DACVECC; Samantha Evans^4^, DVM, PhD, DACVP; Han Joon Lee^3^, DVM; Joong Hyun Song^2^, DVM, PhD; Kun Ho Song^2^, DVM, PhD

#### ^1^College of Veterinary Medicine, Chungnam National University, Daejeon, Korea; ^2^Professor, Internal Medicine, College of Veterinary Medicine, Chungnam National University, Daejeon, Korea; ^3^Internal Medicine, College of Veterinary Medicine, Chungnam National University, Daejeon, Korea; ^4^College of Veterinary Medicine, The Ohio State University, Columbus, Ohio, USA

**Background:** Hypomagnesemia induced by long‐term use of proton‐pump inhibitors (PPI) is a rare but increasingly recognized complication in human medicine. However, there is a lack of information regarding the risk of hypomagnesemia in dogs with long‐term use of PPI.

**Hypothesis/Objectives:** The objectives were to investigate the ionized magnesium (iMG) in dogs with long‐term use of PPI.

**Animals:** Ten client‐owned dogs with long‐term esomeprazole use and thirty healthy control dogs.

**Methods:** Retrospective study. Blood iMG concentrations were measured in each patient by ion‐selective electrodes methods. Inclusion criteria were a dog using esomeprazole more than 6 months. No patient received magnesium supplementation.

**Results:** Median duration of use for PPI was 25 months (range, 6 to 57). Mean iMg was 0.46 ± 0.05 mmol/L after long‐term use of PPI and 0.48 ± 0.05 mmol/L in healthy control dogs. iMg in dogs with long‐term use of PPI did not show a significant difference compared to normal control groups. Hypomagnesemia was not observed in all dogs, and clinical signs relating to hypomagnesemia were also not observed.

**Conclusions and Clinical Importance:** Dogs with long‐term use of PPI did not show hypomagnesemia compared to age‐matched healthy dogs. Further investigations could confirm these findings and evaluate the risk of hypomagnesemia with long‐term use of PPI.

## Abstract GI36: Esomeprazole With and Without Probiotics in Healthy Dogs

### **Rae McAtee** ^1^; Sarah Schmid^2^, DVM, DACVIM (SAIM); Katie Tolbert^3^, DVM, PhD, DACVIM (SAIM); Scott Hetzel^1^, MS; Jessica Pritchard^1^, VMD, DACVIM (SAIM), MS

#### ^1^University of Wisconsin–Madison, Madison, WI, USA; ^2^University of Tennessee, Knoxville, TN, USA; ^3^Texas A&M University, College Station, TX, USA

**Background:** Proton pump inhibitors can cause diarrhea and a transient increase in fecal dysbiosis index (CMDI) in dogs. It is unknown if concurrent probiotic administration mitigates these effects.

Objective/**Hypothesis:** To assess the fecal canine microbial dysbiosis index (CMDI), fecal short‐chain fatty acid (SCFA), and calprotectin concentrations in healthy dogs administered esomeprazole with and without a probiotic.

**Animals:** Sixteen healthy dogs.

**Methods:** Prospective, within‐subjects before and after study. Dogs received a 7‐day course of esomeprazole (1 mg/kg PO q 12 h) with and without a probiotic (15 billion CFU/kg). A 4‐week washout period was administered between treatments. Data were compared between phases using mixed effects ANOVA or generalized estimating equations with post‐hoc Holm adjustment for two‐way comparisons.

**Results:** Fecal CMDI increased with esomeprazole administration (mean CMDI −1.04, SD 3.21) compared to baseline (mean CMDI −2.99, SD 2.78, *P* = 0.017). The fecal CMDI also increased when probiotics were administered concurrently with esomeprazole (mean CMDI 0.39, SD 2.83, *P* < 0.001) compared to baseline. CMDI was not statistically different when esomeprazole was administered alone versus with probiotics (*P* = 0.059). Fecal calprotectin and SCFA concentrations did not differ between phases. The occurrence of vomiting (*P* = 0.003) and diarrhea (*P* = 0.008) increased with the administration of esomeprazole with or without a probiotic compared to baseline and washout phases.

**Conclusions and Clinical Importance:** In healthy dogs, short‐term esomeprazole administration with or without a probiotic induces a transient and mild increase in fecal CMDI with no changes to fecal concentrations of calprotectin or SCFA.

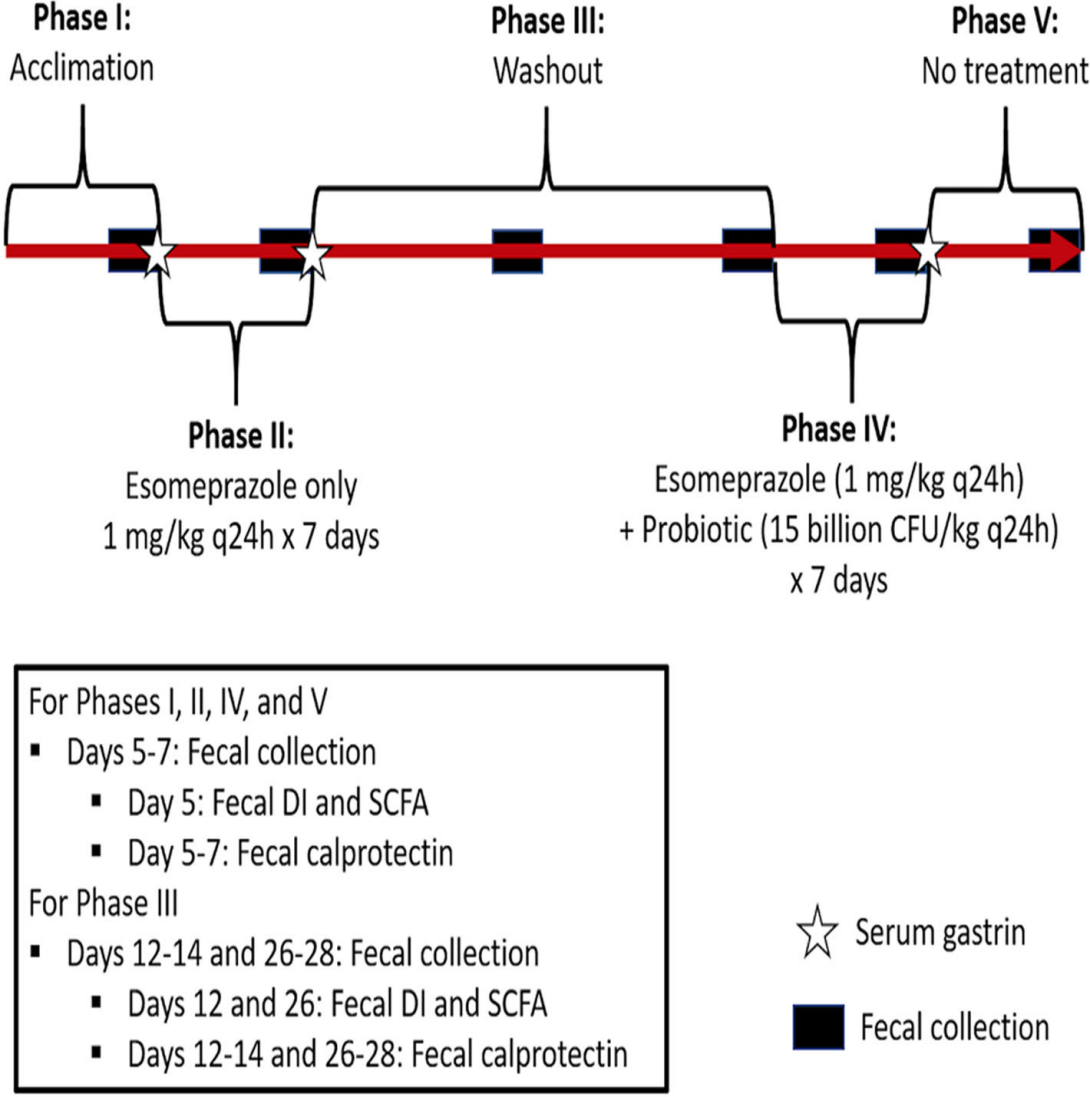

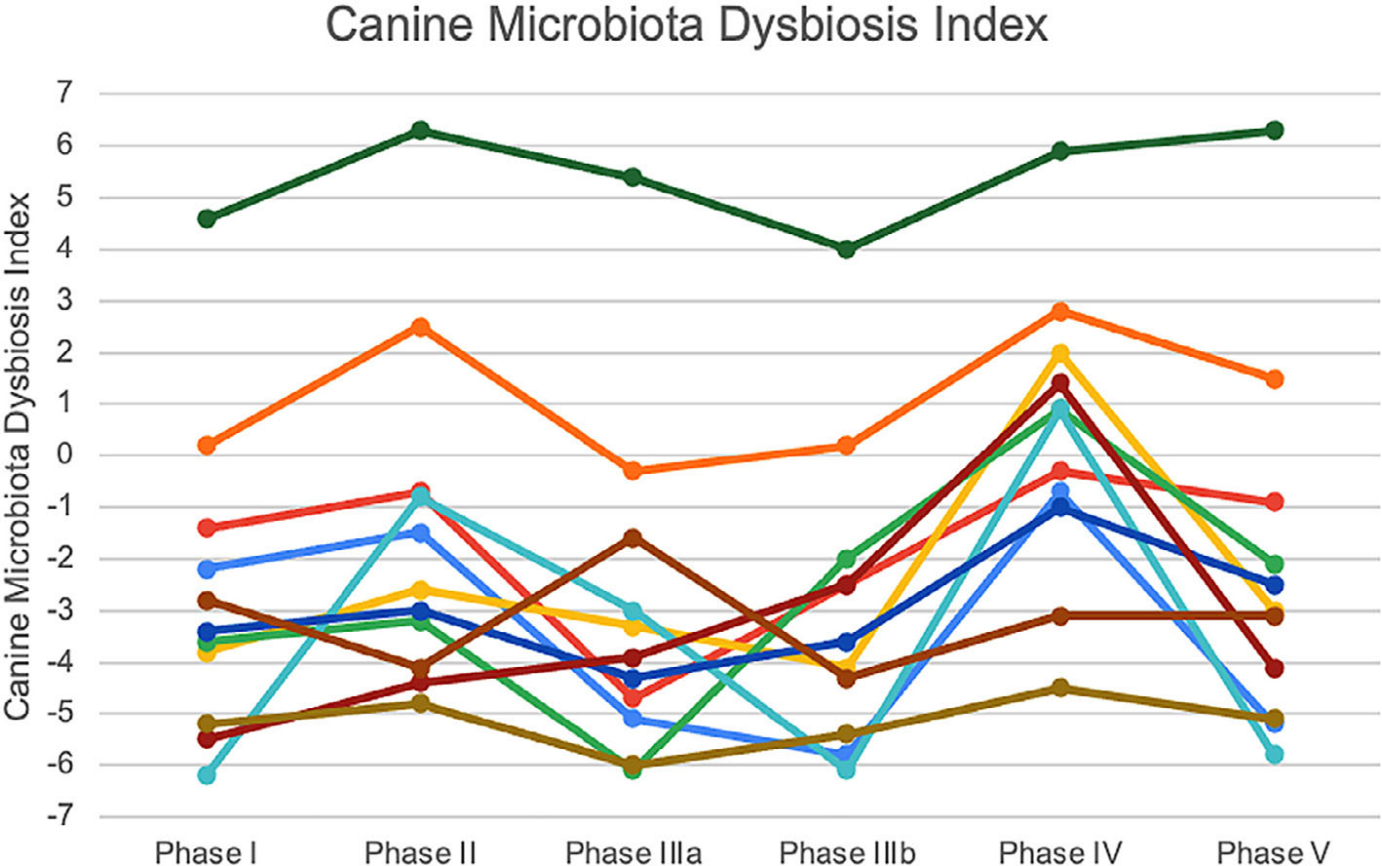

## Abstract GI37: Effect of Short‐Term Dietary Change on Anemia and Diarrhea in Dogs Previously Fed Inadequate Diet

### Hassan Saad Shehata^1^, BS; Hanan Elsayed^2^, PhD; **Nashwa Waly** ^2^, PhD

#### ^1^Assiut University, Assiut, Egypt; ^2^Professor of Veterinary Internal Medicine, Animal Medicine (Internal Medicine), Assiut University, Assiut, Egypt

**Background:** In Egypt, dogs are routinely fed home‐cooked food. Poor nutrition can result in nutritional deficiency disorders including anemia and dietary‐responsive diarrhea, which can result in abandonment of pets and psychological trauma to owners.

**Hypothesis/Objectives:** Correction of nutritional deficiency will result in reversal of anemia and diarrhea. This study aimed at investigating the effect of feeding a balanced diet on the dog's health condition previously kept on home‐prepared food.

**Animals:** A group of four‐month‐old native‐breed dogs (*n =* 9) were housed in our research facility; both males (*n =* 4) and females (*n =* 5) were included.

**Methods:** All dogs were previously fed a standard home‐prepared diet. For 30 days their diet was changed into a balanced commercial dry food. Blood was collected for CBC and stool was evaluated using a standard scoring sheet on days 1 and 30 of the experiment. Data was assessed for normality using Shapiro‐Wilk test. Parametric/non‐parametric tests were used accordingly using SPSS.

**Results:** The complete blood picture showed that eight dogs (89%) suffered from microcytic normochromic anemia. On day 30, 7 dogs (87.5%) recovered from anemia. There was a significant improvement in anemic parameters following change of diet. Among others, red cells (TRBCs) increased from 5.15 ± 0.335 to 5.63 ± 0.54 (mean ± SD). Hemoglobin concentration also changed from 10.9 ± 0.59 to 12.7 ± 1.2. All changes were significant (*P* < 0.05). Fecal consistency changed from unformed on day 7 to formed on day 30 (*P* = 0.01)

**Conclusions:** Dietary‐related anemia can be corrected in a month. Improvement of fecal quality will reduce traumatic conditions for owners.

**Figure:**
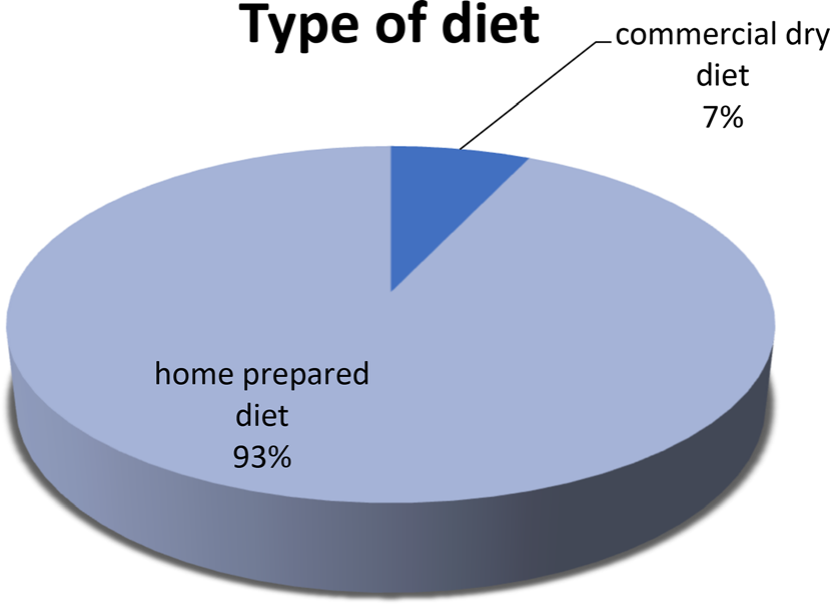
Figure 1. A poll was carried out in our clinic, where clients (dog owners) were questioned on the type of diet (commercial diet and/or home‐prepared diet) to dogs. A pie chart representing percentage of pet sample fed on home‐prepared diet vs. commercial dry diet in Assiut.

**Table:** Table 1. Fecal score, hemoglobin, total RBCs, MCV and MCH results on days 1 and 30 for experiment dogs

Day of experiment	Parameters				
	Fecal score (Royal Canin scoring system)	Hemoglobin (g/dl)	Total RBCs (10^12^/l)	MCV	MCH
D1	3 ± 0.26	10.9 ± 0.59	5.15 ± 0.335	56.7 ± 1.9	21.18 ± 0.7
D30	4 ± 0.6*	12.7 ± 1.2*	5.63 ± 0.54*	62 ± 1.9*	22.57 ± 0.6*

D1, day 1 of the experiment; D30, day 30 of the experiment; SD, standard deviation

Data are expressed as mean ± standard deviation of each variable.

## Abstract GI38: Evaluation of Variation in Fecal Microbiota and Bile Acids Over Time in Healthy Pet Cats

### **Jan S. Suchodolski** ^1^; Chi Sung^1^; Yu Wu^1^; Joao Cavasin^1^; Jonathan Lidbury^1^; Joerg Steiner^1^; Sina Marsilio^2^

#### ^1^Texas A&M University, College Station, TX, USA; ^2^University of California–Davis, Davis, CA, USA

**Background:** The fecal microbiota and its derived metabolites such as secondary bile acids are important for intestinal health and are often altered in chronic enteropathies. The feline dysbiosis index (fDI), a qPCR‐based assay, is a reproducible test developed to evaluate shifts in intestinal microbiota of cats with CE and after antibiotic administration. Little is known about variation of the microbiota and bile acid (BA) metabolism in healthy cats over time.

**Hypothesis/Objectives:** The aim of this study was to evaluate temporal variations in the fDI and unconjugated BA in fecal samples of healthy cats.

**Animals:** 16 healthy pet cats.

**Methods:** Naturally passed fecal samples from each cat were collected at least at 4 time points over two months, while additional fecal samples from 3 cats were collected between 9–34 times over the same period. The fDI was assessed by qPCR. Fecal unconjugated BAs were measured by mass spectrometry. Friedman tests were used to compare the DI and BAs across time points.

**Results:** Results of DI (*P* = 0.497), total BA (*P* = 0.645), and percentage of primary BA (*P* = 0.432) measured at TP 2, 3, and 4 did not differ from baseline. Although fDI and BA composition fluctuated in some cats, all evaluated parameters remained within the established reference intervals at all time points.

**Conclusions and Clinical Importance:** The fDI and BAs appear to be stable in healthy pet cats, as all parameters evaluated across time points over 2 months were within the respective reference intervals.

## Abstract HM01: Sample Collection Effect on Canine Fecal Occult Blood Testing and Utility of Hemoglobin Immunochemical Tests

### **Kelly Chappell** ^1^; Laura Van Vertloo^1^, DVM, MS, DACVIM (SAIM); Austin Viall^2^, DVM, DACVP; Jennifer Scaccianoce^1^, DVM; Dana LeVine^3^, DVM, PhD, DACVIM

#### ^1^Iowa State University, Ames, IA, USA; ^2^University of California Davis, Davis, CA, USA; ^3^Auburn University, Auburn, AL, USA

**Background:** The fecal occult blood test (FOBT) is important for diagnosing challenging anemias but voided fecal samples can be difficult to obtain. Sample collection method impact on FOBTs has not been assessed in veterinary medicine. In human medicine, fecal hemoglobin immunochemical tests (FITs) have largely replaced conventional FOBT methods due to their higher sensitivity and specificity. FITs have not been trialed in companion animals.

**Hypothesis/Objectives:** The primary objective was to determine if sample collection method (voided vs. digital rectal collection) affects FOBT results. The secondary objective was to assess human FITs’ capacity to detect canine and feline blood.

**Animals:** 308 privately owned dogs; healthy and sick.

**Methods:** Guaiac FOBTs were performed on paired voided and rectally obtained fecal samples. Two FITs (Hemosure One Step and OC‐Light S) were tested with serially diluted human, canine, and feline blood.

**Results:** Voided and rectally obtained samples showed good FOBT positivity agreement (*k* = 0.80), with 92.5% concordance and 13/308 dogs negative on void but positive on rectal. Multivariate analysis showed dogs with gastrointestinal disease (*P* = 0.0003 rectal; *P* = 0.0001 void) were more likely, and heavier dogs (*P* = 0.0037 rectal; *P* = 0.0022 void) less likely, to test positive. Health status, platelet count, fasting status, NSAID use, and age were associated with FOBT result on univariate, but not multivariate, analysis. FITs did not detect canine or feline blood.

**Conclusions and Clinical Importance:** Rectally obtained fecal samples can be reliably used for FOBTs. Human FITs may not be suitable for companion animals, but evaluation of further tests is needed.

## Abstract HM02: Leukocyte and Platelet Ratios in Dogs Diagnosed With Non‐associative Immune‐mediated Hemolytic Anemia

### Luke O’Sullivan^1^; **Antoine Duclos** ^1^, Dr Vét.; Ciara McPhedran^2^; Samuel Hocker^3^; Kevin LeBoedec^4^; Shauna Blois^5^; Benoît Cuq^6^, Dr Vét. Ph.D. DACVIM‐SAIM MRCVS

#### ^1^UCD School of Veterinary Medicine, Dublin, Ireland; ^2^Ontario Veterinary College, Guelph, ON, Canada; ^3^Evolution Veterinary Specialists, Lakewood, CO, USA; ^4^Centre Hospitalier Vétérinaire Frégis, Paris, France; ^5^Ontario Veterinary College, Guelph, ON, Canada; ^6^Assistant Professor in Small Animal Medicine, Small Animal Clinical Studies, UCD School of Veterinary Medicine, Dublin, Ireland

Canine immune mediated hemolytic anemia (IMHA) has a high mortality rate, especially in the immediate post‐diagnosis period. In human medicine, high neutrophil‐to‐lymphocyte (NLR) and platelet‐to‐lymphocyte ratios (PLR) are associated with a poor prognosis in cancer patients and in patients with pulmonary thromboembolism. In veterinary medicine, NLR has shown promising prognostic potential in dogs with solid tumors or sepsis, but limited data is available in dogs with IMHA.

Retrospective evaluation of 225 medical records of dogs diagnosed with IMHA was performed at two referral hospitals. The effects of hematological parameters, NLR, PLR, monocyte‐to‐lymphocyte ratio (MLR), lymphocyte‐to‐platelet ratio (LPR), monocyte‐to‐neutrophil ratio (MNR), band‐to‐neutrophil ratio (BNR), neutrophil‐to‐platelet ratio (NPR) and neutrophil‐to‐platelet and lymphocyte ratio (NPLR) on survival to discharge, 1 month and 6 months, on the number of blood transfusions required, and on erythrocyte regeneration were assessed. Survival rate was 80%, 70%, and 63% at discharge, 1 month, and 6 months, respectively. There was no significant association between any of the reported ratios and survival or number of blood transfusions. Elevated leukocyte (O*R =* 1.04 [1.01–1.08], *P* = 0.009,), neutrophil (O*R =* 1.06 [1.02–1.11], *P* = 0.005), band‐cell (O*R =* 1.24 [1.01–1.51], *P* = 0.037), and monocyte (O*R =* 1.70 [1.21–2.37], *P* = 0.002) counts as well as elevated NLR (O*R =* 1.04 [1.00–1.08], *P* = 0.044) and MLR (O*R =* 1.8 [1.18–2.60], *P* = 0.005) were significantly associated with erythrocyte regeneration at presentation.

The various ratios investigated failed to predict outcomes or requirement for transfusions in dogs diagnosed with IMHA. Regeneration was associated with various hematology variables, likely indicating bone marrow simultaneous stimulation of erythroid, lymphoid, and myeloid lines in IMHA patients.

## Abstract HM03: Neutrophil‐lymphocyte Ratio, Platelet‐lymphocyte Ratio and Mean Platelet Volume as Prognostic Biomarkers in Critically Ill Dogs

### **Francisco De Membiela** ^1^; Rachel Clifton^2^, VetMB PhD; Charlotte Dye^1^, BVM&S PhD DSAM (feline) DECVIM‐CA MRCVS

#### ^1^Pride Veterinary Referrals, Derby, UK; ^2^University of Nottingham, Nottingham, UK

**Background:** The ability to make outcome and mortality predictions is pivotal to clinical decision making in critically ill patients. Specific markers of inflammation and platelet activation derived from routine haematology profiles, such as neutrophil‐lymphocyte ratio (NLR), platelet‐lymphocyte ratio (PLR) and mean platelet volume (MPV), are widely accessible and low‐cost parameters that have been previously utilized as prognostic tools in dogs with a variety of medical conditions. In humans, these parameters have also been applied in critically ill patients.

**Hypothesis/Objectives:** To investigate whether NLR, PLR, and MPV can be used as prognostic biomarkers in a heterogeneous population of critically ill dogs.

**animals and methods:** The medical records of 200 dogs admitted to the ICU over a 3‐year period were retrospectively reviewed. Binomial regression models were used to investigate associations between NLR, PLR and MPV and survival. In dogs that survived, Kaplan Meier plots, log‐rank tests and cox proportional hazards models were used to investigate the association between NLR, PLR, and MPV and length of hospitalization.

**Results:** NLR values above normal were associated with longer hospitalization and higher mortality. When excluding dogs with neoplasia, dogs with higher MPV or PLR values were more likely to die or be euthanised than those with lower values.

**Conclusions and Clinical Importance:** The association of NLR on day of admission with length of hospitalization and mortality suggests the potential for its use as a prognostic biomarker. Further studies are required to confirm whether this parameter can be used to help predict outcome in critically ill dogs.

## Abstract HM04: β1‐tubulin Mutations in Non‐Cavalier King Charles Spaniel Dogs With Macrothrombocytopenia

### **Elizabeth A. Luciani** ^1^; Peter Christopherson^2^, DVM, PhD, DACVP; Jessica Pritchard^3^, VMD, MS, DACVIM (SAIM); Lisa Kim^4^; Nina Zitzer^5^, DVM, PhD, DACVP (Clinical Pathology); Erin Lashnits^6^, MS, DVM, PhD, DACVIM (SAIM)

#### ^1^UW Veterinary Care, University of Wisconsin‐Madison, Madison, WI, USA; ^2^Pathobiology, Auburn University College of Veterinary Medicine, Auburn, AL, USA; ^3^Medical Sciences, School of Veterinary Medicine, University of Wisconsin, Madison, WI, USA; ^4^Veterinary Student, School of Veterinary Medicine, University of Wisconsin, Madison, WI, USA; ^5^Pathobiological Sciences, School of Veterinary Medicine, University of Wisconsin, Madison, WI, USA; ^6^Medical Sciences, School of Veterinary Medicine, University of Wisconsin, Madison, WI, USA

**Background:** Thrombocytopenia is the most common canine hemostatic disorder. Non‐clinical congenital macrothrombocytopenia in Cavalier King Charles Spaniels (CKCS) is caused by a mutation in the β1‐tubulin gene. The prevalence of this gene mutation in non‐CKCS dogs has not been thoroughly investigated.

**Hypothesis/Objectives:** To determine the prevalence of the currently reported CKCS β1‐tubulin mutation associated with macrothrombocytopenia in non‐CKCS dogs with macrothrombocytopenia.

**Animals:** Sixty‐one client owned dogs with macrothrombocytopenia

**Methods:** This is a prospective observational descriptive study. Blood samples were banked from patients presenting to the UW Veterinary Care in which a thrombocytopenia was noted. The medical records of these dogs with a noted macrothrombocytopenia (platelet count <175 *10^3/uL, MPV > reference) between April 2021 to March 2022 were reviewed. Blood samples were submitted for DNA extraction and PCR testing to evaluate for the presence of the known mutations in the TUBB1 gene.

**Results:** 61 dogs were noted to have a macrothrombocytopenia with no documentation of normal values. There were 32 females (52.5%) and 29 males (47.5%), and the most common included breeds were German Shepherd Dogs (*n =* 5), Golden Retrievers (*n =* 5), and Boxers (*n =* 5). The median platelet count was 110 × 10^3^/μL (range 9–171). The number and proportion of dogs of each breed positive for the β1‐tubulin mutation will be reported, and the demographic and clinical histories for positive dogs will be presented.

**conclusions/clinical importance:** The presence of β1‐tubulin mutations in non‐CKCS dogs would indicate that testing for this mutation should be integrated into a diagnostic evaluation of macrothrombocytopenic dogs.

## Abstract HM05: Red Blood Cell Indices Performance to Detect Reticulocytosis in Anemic and Non‐anemic Dogs in Taiwan

### **Kimberly S. Yore** ^1^; Li‐Wen Chang^2^, DVM, PhD; Dennis DeNicola^3^, DVM, PhD, DACVP; Donald Szlosek^4^

#### ^1^IDEXX; ^2^Senior Medical Affairs Manager, IDEXX Taiwan, IDEXX Laboratory, Inc.; ^3^Veterinary Pathology Consultant, Laboratory Retrievers, LLC; ^4^Biostatistician, Data Insights, IDEXX Laboratory, Inc.

**Background:** Reticulocyte counts (RETIC) are considered the gold standard in detecting an erythroid bone marrow response (BMR) in anemic and non‐anemic dogs. Reticulocytosis without anemia (RWA) is a potential indicator of disease. Geographical and breed variations may render differing prevalence of anemia or RWA and differing performance of RBC indices to detect a BMR.

**Objective:** This study aimed to determine prevalence of anemia, performance of RBC indices to detect a BMR, and prevalence of RWA of dogs in Taiwan.

**Animals:** Final analysis population consisted of 149,076 dogs.

**Methods:** A cross sectional, retrospective analysis of CBC samples from ProCyte Dx hematology analyzers in Taiwan from December 1, 2018 through December 31, 2020, assessed sensitivity and specificity of increased MCV and decreased MCHC (MCV/MCHC) results to detect reticulocytosis.

**Results:** Among 149,076 dogs included, 11.8% (*n =* 17,600) had reticulocytosis (RETIC >110 K/μl) and 21.8% (*n =* 32,474) had anemia (HCT <37.3%). Of 32,474 anemic dogs, 17.8% (*n =* 5,789) had reticulocytosis. Of 116,602 dogs without anemia, 10.1% (*n =* 11,776) had reticulocytosis. Sensitivity/specificity of MCV/MCHC to detect BMR was 4.3% and 99.4%, respectively compared to RETIC across all dogs. Among anemic dogs, sensitivity/specificity of MCV/MCHC to detect BMR was 9.6% and 99.2%, respectively. Among non‐anemic dogs, sensitivity/specificity of MCV/MCHC to detect BMR was 1.6% and 99.5%, respectively.

**Conclusions and Clinical Importance:** Most regenerative anemias and cases of RWA did not have combined increased MCV and decreased MCHC. To avoid overlooking potential illness, RETIC should be evaluated regardless of whether patients have anemia or not.

## Abstract HM06: Heparin Induced VCM Tracing Changes Compared to Aptt and Anti‐xa Activity in *Ex Vivo* Canine Blood

### **Gina K. Dinallo** ^1^; Louisa Rahilly^2^, DVM, DACVECC; Anna Podlasek^3^, MD

#### ^1^Boundary Bay Veterinary Specialists, Bellingham, WA, USA; ^2^Chief of Staff, Emergency and Critical Care Specialist, ECC, Cape Cod Veterinary Specialists, Bourne, MA, USA; ^3^Radiology Fellow, Division of Radiological and Imaging Sciences, University of Nottingham, Nottingham, UK

**Background:** Heparin is a widely utilized treatment for thromboprophylaxis. Currently, no reliable cage‐side option is available to monitor heparin therapy. As many hypercoagulable disease states are dynamic, real‐time monitoring ability is crucial. The VCM offers a novel potential monitoring option.

**Hypothesis/Objectives:** To evaluate the impact of unfractionated heparin (UFH) on canine whole blood VCM tracings, and assess the relationship between VCM variables, aPTT, and anti‐Xa levels.

**Animals:** Six healthy adult dogs.

**Methods:** Prospective benchtop study. Untreated whole blood samples were collected from each dog into UFH‐free syringes and pre‐heparinized syringes at three *ex vivo* UFH doses (100 U/kg, 250 U/kg, and 600 U/kg equivalent concentrations) for analysis. Correlations aPTT, anti‐Xa levels, and viscoelastic (VCM) variables were assessed at each UFH dose.

**Results:** CT, CFT, α angle, α10, α20, and MCF changed significantly (*P* < 0.05) as UFH dose increased. However, there was no significant correlation between VCM variable values and either aPTT or anti‐Xa levels.

**Conclusions/clinical Importance:** This is the first known attempt to utilize the VCM to monitor the effect of UFH on canine blood. Statistically significant changes were appreciated between each UFH dose for multiple VCM variables. While anti‐Xa levels and VCM variables were not found to have a statistically significant correlation, visible changes in the tracings suggest a relationship between CT, CFT, α angle, MCF, and anti‐Xa levels. *In vivo* research in a larger population is warranted to solidify the clinical role of the VCM in cageside monitoring of heparin therapy.

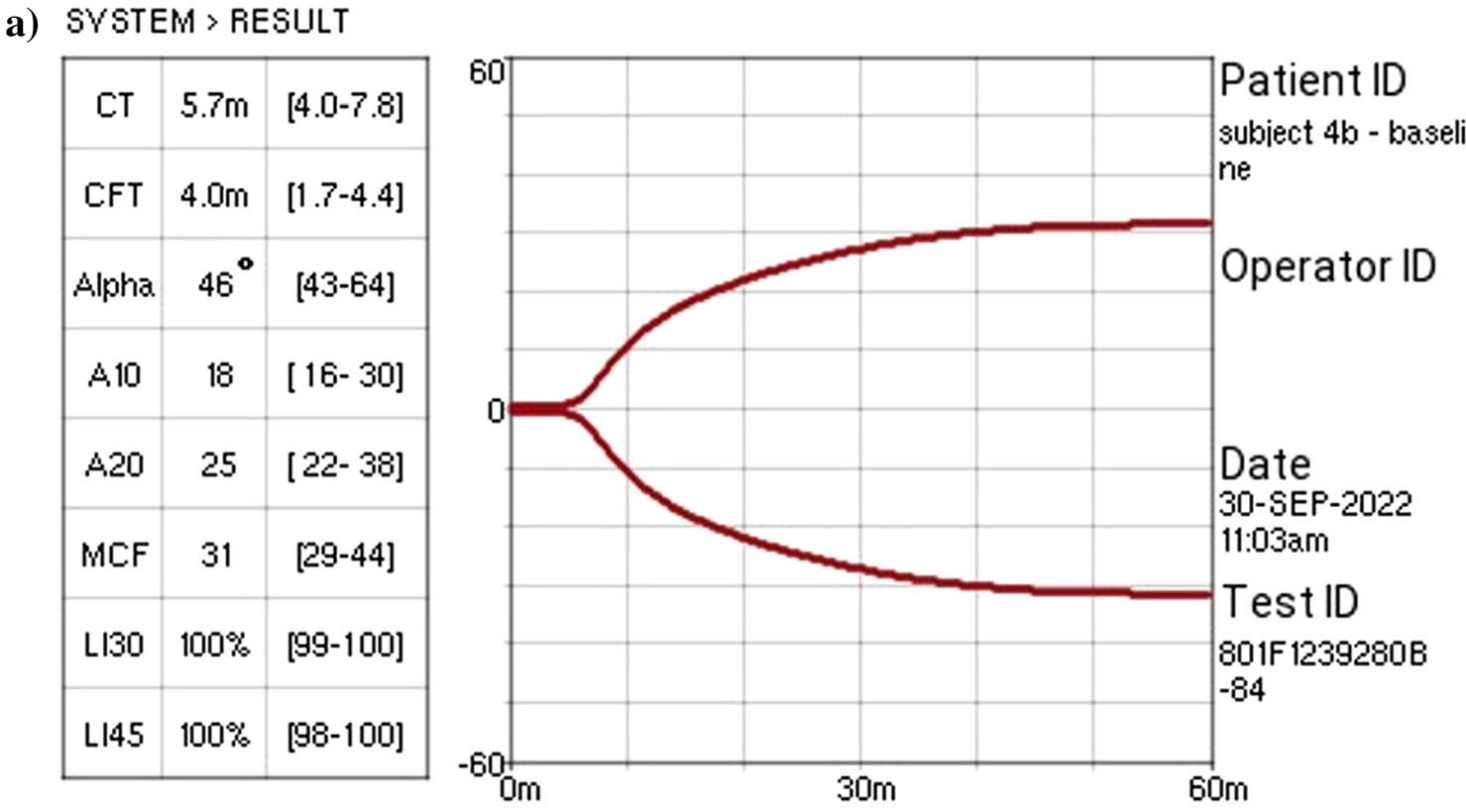

**Figure:**
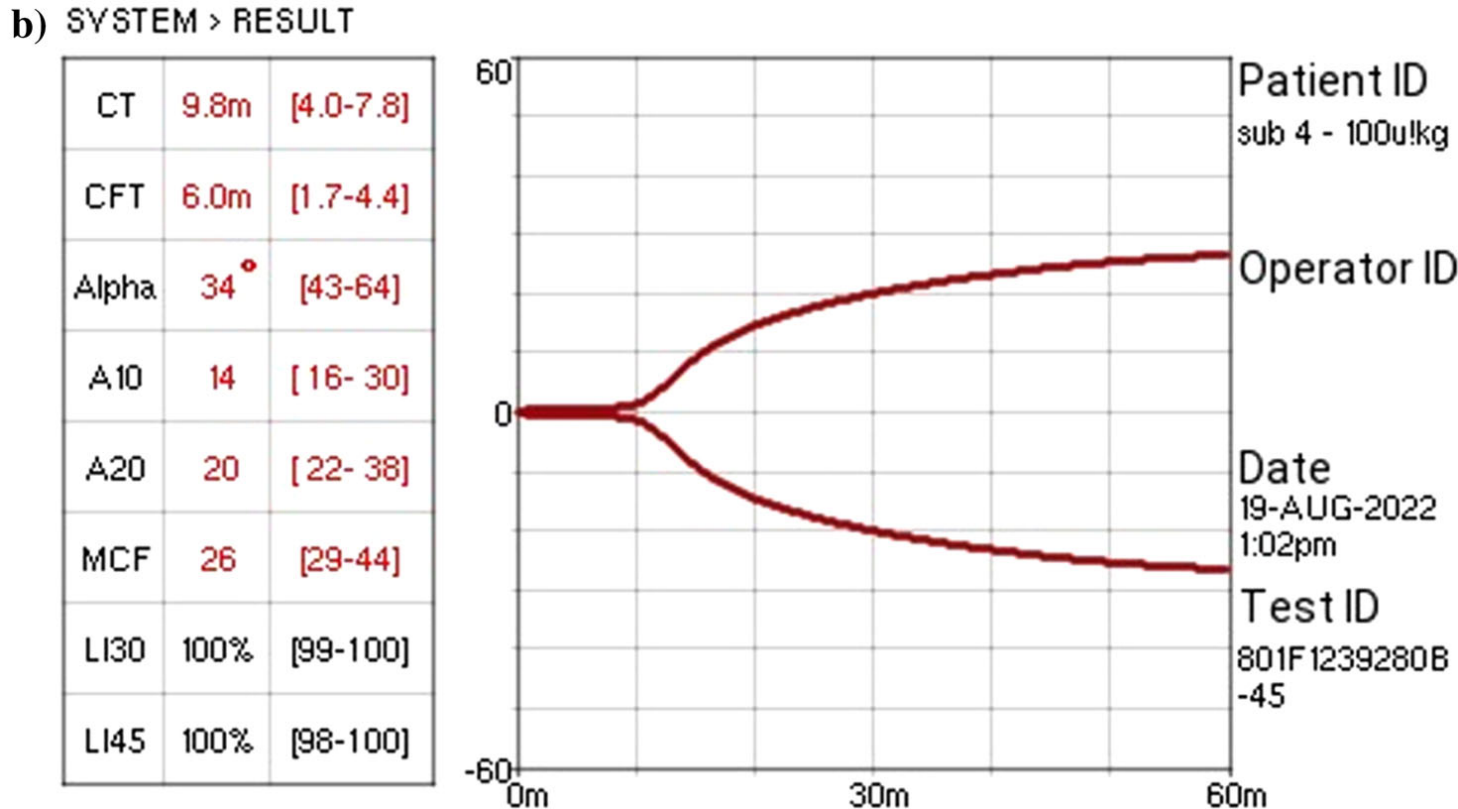
Figures a‐d: VCM tracings at 0 u/kg (a), 100 U/kg (b), 250 U/kg (c), and 600 U/kg (d) heparin from a single study subject. a) has normal values for all VCM variables. b) shows mild increase in CT and CFT, and mild decrease in α angle, α 10, α 20, and MCF. c) shows marked increase in CT and CFT, and marked decrease in α angle, α 10, α 20, and MCF. d) shows extreme prolongation of CT and an MCF of 0, with the machine unable to interpret the other variables, producing a flatline tracing.

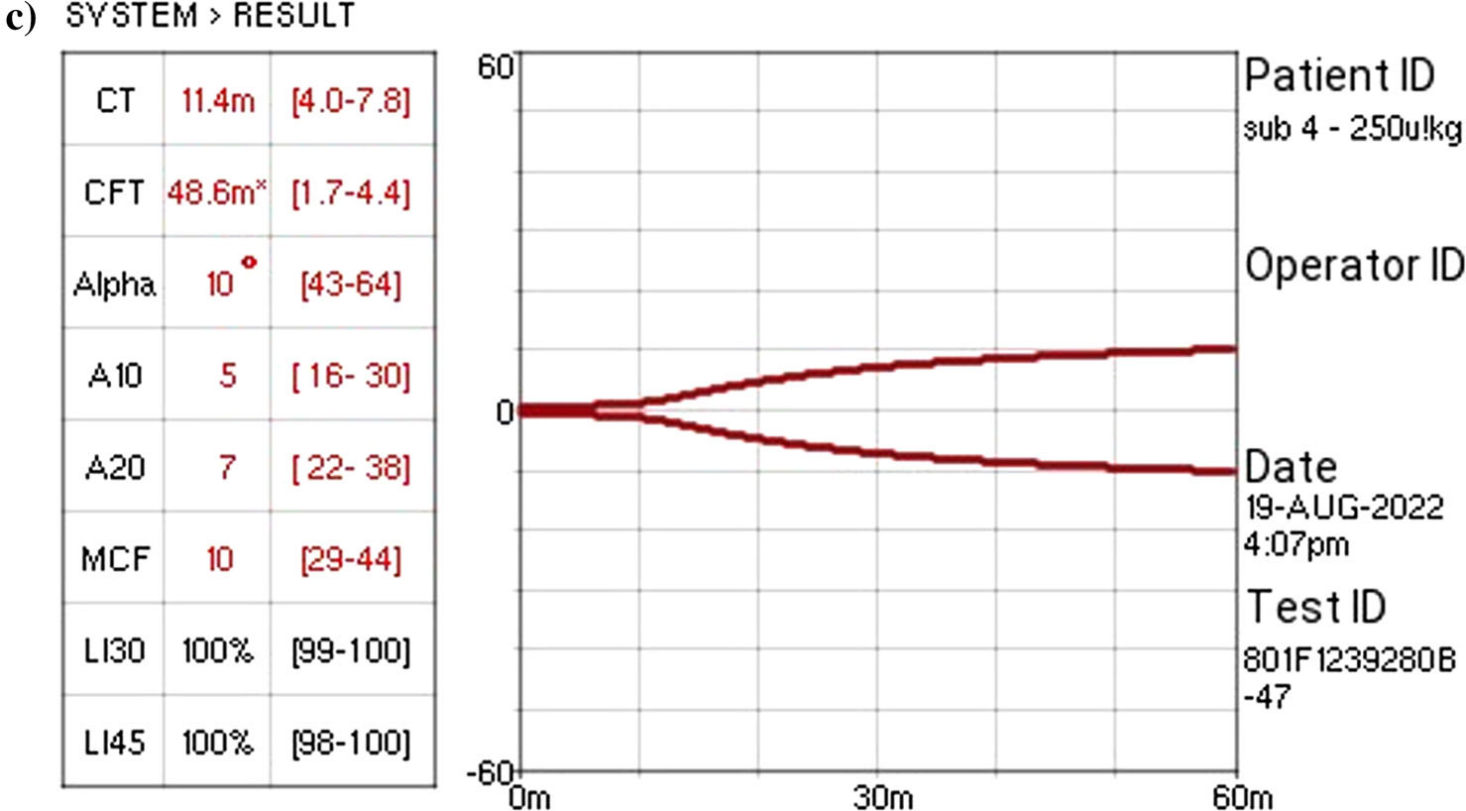

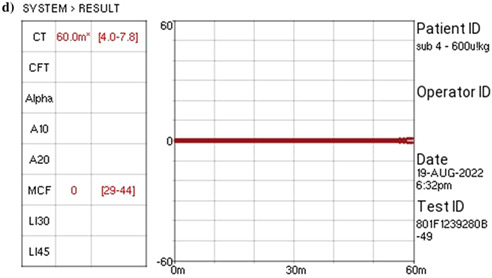

## Abstract HM07: Defining the Platelet Transcriptome in Dogs

### **Jennifer Weng**; Sarah Shropshire, DVM, PhD, DACVIM (SAIM); Dylan Ammons; Lyndah Chow, PhD; Steven Dow, DVM, PhD, DACVIM (SAIM)

#### Colorado State University, Fort Collins, CO, USA

**Background:** Little is known regarding the platelet transcriptome in health or disease, but application of next‐generation RNA sequencing technologies can provide unprecedented new insights into platelet function and dysfunction.

**Hypothesis/Objectives:** We hypothesized that platelet transcriptome in dogs could be sequenced using single‐cell and bulk RNA sequencing. Platelet samples from healthy adult dogs were subjected to transcriptomic analysis with the objective of creating a healthy platelet sequence database for analysis of platelets from dogs with diseases associated with high thromboembolic risk.

**Animals:** Blood samples were prospectively collected from 10 healthy dogs.

**Methods:** Platelet transcriptomes were sequenced using single‐cell RNA sequencing (SCS) (6/10 samples) or bulk RNA sequencing (4/10 samples). For SCS analysis, cells were prepared from blood leukocytes using a 10X Chromium instrument while bulk RNA sequence was done using purified platelets and ultra‐low‐pass RNA sequencing. Descriptive statistics were utilized.

**Results:** SCS analysis identified three unique subpopulations of platelets, and uniquely upregulated genes were identified to differentiate platelets from other leukocytes, including genes associated with expression of integrins, cytoskeletal proteins, coiled domain proteins and carboxylase proteins. These findings indicate that platelets remain transcriptionally active in circulation and that their transcriptomic profiles can be defined using RNA sequencing technologies.

**Conclusions and Clinical Importance:** Transcriptomic profiling of platelets can be used to define healthy platelet function and to identify unique gene expression profiles associated with dysfunctional platelets in prothrombotic diseases. This information can guide targeted therapies for prevention and treatment of thrombotic diseases.

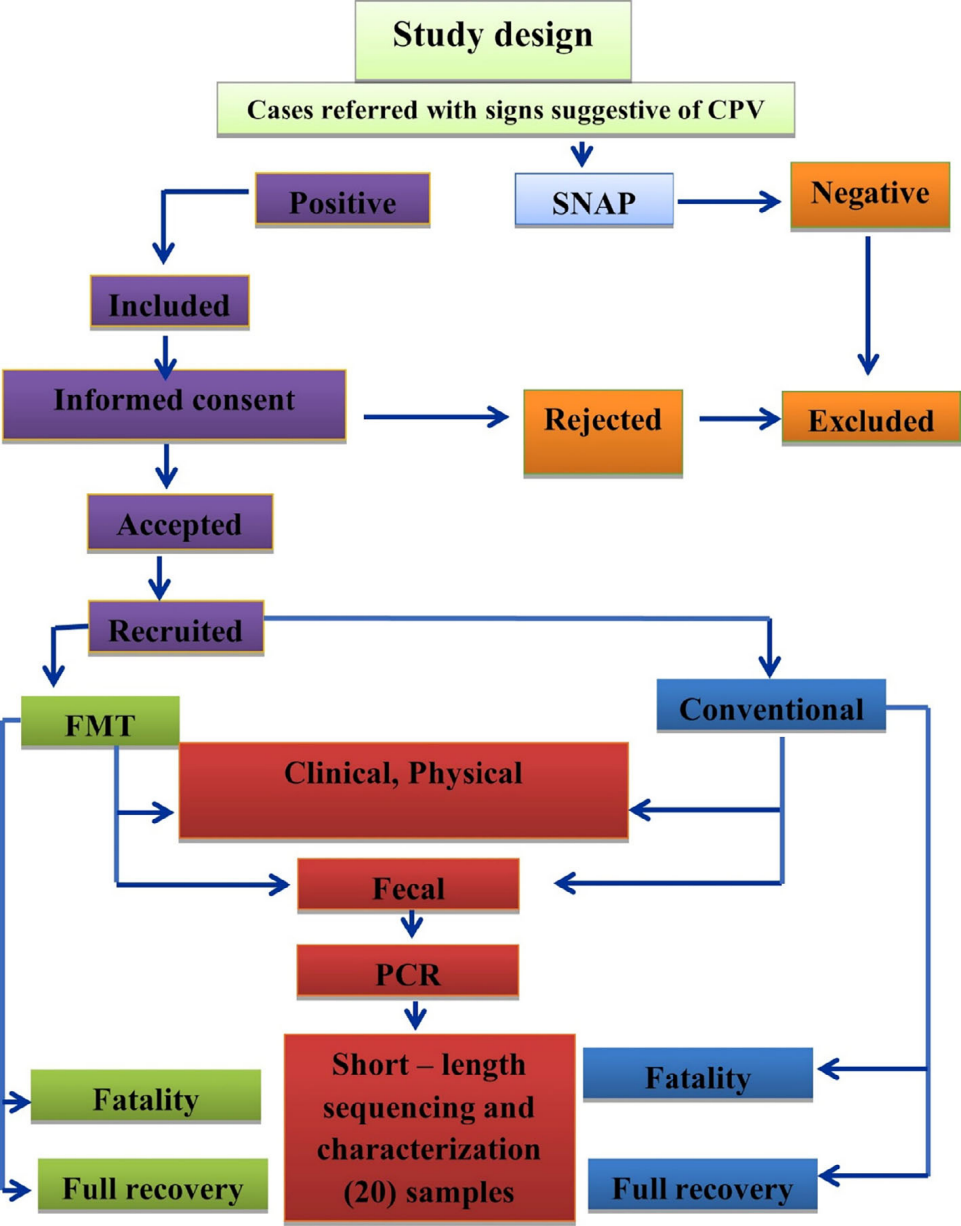

## Abstract HM08: Comparison of C‐Reactive Protein Measurement on Three Assays and Following Storage

### **Amanda D. Garrick** ^1^; Jennifer Hawley^2^, ACRP‐CP; Tracy Wangler^3^, BS, CVT, MLS (ASCP)^CM^; Russell Moore^4^, DVM, MS, DACVP; Michael Lappin^5^, DVM, PhD, DACVIM

#### ^1^Colorado State University, Fort Collins, CO, USA; ^2^Senior Research Associate, Colorado State University, Fort Collins, CO, USA; ^3^Medical Laboratory Scientist, Clinical Pathology, Colorado State University, Fort Collins, CO, USA; ^4^Faculty, CVMBS Microbiology, Immunology, and Pathology Department, Colorado State University, Fort Collins, CO, USA; ^5^Clinical Sciences Department Section Head, Professor, Small Animal Internal Medicine, Colorado State University, Fort Collins, CO, USA

**Background:** C‐reactive protein (CPR) serum concentrations are used to monitor systemic inflammatory disease syndromes. There are multiple methods of measuring CRP but no clear consensus on the optimal assay. CRP concentrations are believed to degrade even if sera are frozen, impacting retrospective studies.

**Objectives:** This study was intended to assess for degradation of CRP when stored at −80°C and to compare the results of three different commercially available CRP assays.

**Animals:** Sera (*n =* 19) collected from research beagles in other experiments with IACUC approval.

**Methods:** The 19 sera were originally assayed with a commercially available ELISA (Abcam—canine). The sera were stored for approximately 14 weeks at −80°C until assayed in this study. After thawing at room temperature, the sera were re‐assayed in the ELISA and 2 other assays (Randox Canine CRP and Gentian CRP‐G).

**Results:** Of 19 samples re‐tested in the same ELISA after storage at −80°C, 18 showed increased CRP concentrations, with changes ranging from 24 to 2000% (median *=* 43%.) While the Abcam and Gentian assays detected CRP in all 19 samples, 7 samples were negative in the Randox assay. CRP detection rates were similar in the Abcam (100%), Randox (83.3%) and Gentian (100%) assays using the 6 samples most likely to show inflammation.

**Conclusions and Clinical Importance:** If CRP measurement is used in management of clinical cases, the assay used in serial monitoring should be consistent. Using the Abcam kit, CRP concentrations generally increased after storage rather than decreased as expected.

## Abstract HM09: Validation of the Calibrated Automated Thrombogram Using Low Plasma Volumes in Dogs

### **Erin M. Phillips**; Anthony Abrams‐Ogg; Shauna Blois; Benoit Cuq; Darren Wood

#### Ontario Veterinary College, Guelph, ON, Canada

**Background:** Thrombin generation (TG) assessment using calibrated automated thrombogram (CAT) requires a standard volume of plasma (80 μL) and reagent (20 μL) run in triplicate. A low‐volume CAT method, using half the volume of both plasma and reagent, has been developed in humans and is appealing in veterinary medicine, as it lowers the sampling burden in patients.

**Objective:** Compare results of CAT in dogs using a standard method and method using low plasma and reagent volumes.

**Animals:** 30 client‐owned dogs with low, normal and high thrombin generation potential recruited from a tertiary referral hospital.

**Methods:** Blood samples were collected by direct jugular venipuncture in hypocoagulable (*n =* 10), hypercoagulable (*n =* 10) and normal (*n =* 10) dogs. Method comparison was performed between CAT using standard (80 μL plasma, 20 μL reagent—method 1) and low volume (40 μL plasma, 10 μL reagent—method 2) plasma and reagent, respectively. Four parameters of the TG curve were assessed: lag time (lag); endogenous thrombin potential (ETP); peak; and time to peak (ttpeak).

**Results:** There was excellent agreement between methods 1 and 2 for all parameters. Lin's concordance coefficients were 0.97, 0.94, 0.96 and 0.96 for lag time, ETP, ttpeak and peak, respectively. There was a small bias for all parameters (*P* < 0.05), resulting in a significant change for lag only. There was a significant predictive linear equation, that when modeled allowed conversion of method 2 to method 1 values.

**Conclusion and Clinical Importance:** Low volume CAT is a valid alternative to the standard testing method in dogs.

## Abstract HM10: Effectiveness of Clopidogrel in Preventing Post‐operative Hypercoagulability in Dogs Undergoing Splenectomy for Splenic Masses

### **Joong‐Hyun Song**; Guk‐Il Joung

#### Department of Veterinary Internal Medicine, College of Veterinary Medicine, Chungnam National University, Daejeon, Korea

**Background:** Dogs that had undergone splenectomy are predisposed to fatal thrombotic conditions. Marked thrombocytosis has been well‐known as a risk factor for post‐splenectomy hypercoagulability.

**Objective:** To determine the preventive effect of clopidogrel on post‐operative hypercoagulability during the first 2 weeks after splenectomy in dogs with splenic masses by platelet counts (PCs) and thromboelastographic (TEG) values.

**Animals:** Twelve dogs had undergone splenectomy. 7 dogs received no treatment (group A), and 5 dogs were treated with clopidogrel (group B).

**Methods:** Prospective study. Blood samples were collected on the day of surgery and 2, 7, and 14 days after splenectomy in both groups. In group B, TEG was performed on the same days. Clopidogrel was loaded at 10 mg/kg on day 2 and continued 2 mg/kg until day 14.

**Results:** In group A, there was a significant elevation of PCs on day 7 (*P* = 0.007) and day 14 (*P* = 0.001) compared to day 0. In group B, the PCs were significantly elevated on day 7 (*P* = 0.032) but no significant difference was found on day 14 compared to day 0. PCs on day 14 were significantly higher in group A than group B (*P* = 0.03). In TEG analysis, no significant differences were found in the K and α‐angle values at all postoperative assessment points compared to day 0.

**Conclusion and Clinical Importance:** Marked thrombocytosis was evident during the first 2 weeks after splenectomy. Clopidogrel may have preventive effects of thrombocytosis and related‐hypercoagulability in dogs undergoing splenectomy for splenic masses.

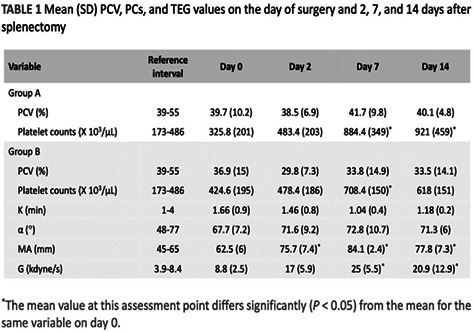

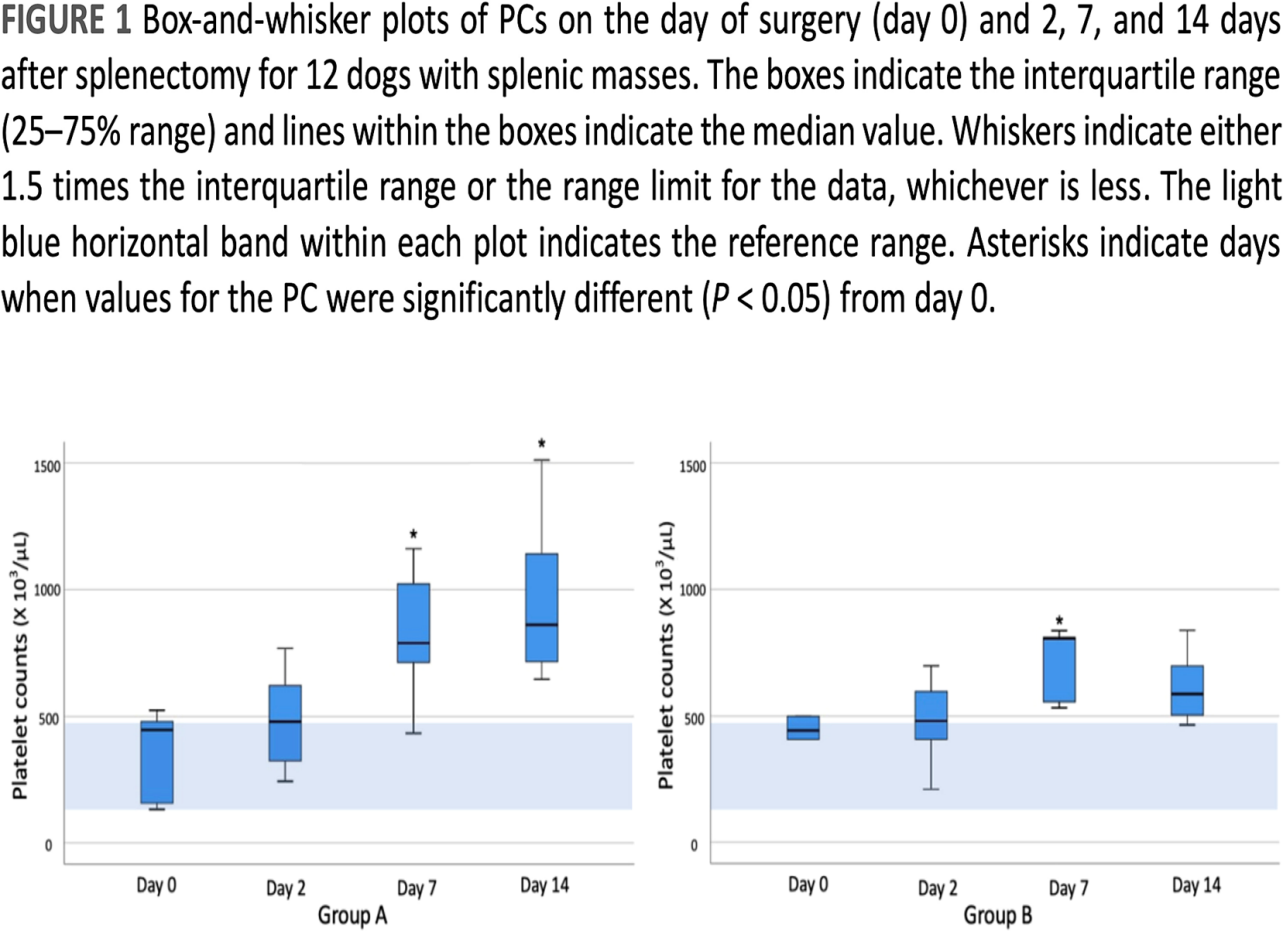

## Abstract HM11: Assessing the Reliability of Blood Samples Collected From Peripheral Intravenous Catheters

### **Chia‐Chen Wu**; Shang‐Lin Wang

#### National Taiwan University Veterinary Hospital, Taipei City, Taiwan

**Background:** Drawing blood from a peripheral intravenous catheter (PIVC) can be an efficient alternative to venipuncture, as it causes less traumatic entries to veins and minimizes patient discomfort, bruising and distress. There is a current research gap in comparing the effectiveness of blood sampling from PIVCs to venipuncture.

**Hypothesis/Objectives:** To investigate if blood sampling from a PIVC can be an alternative to direct venipuncture (DV). It is hypothesized that both sampling methods would lead to clinically interchangeable results.

**Animals:** Twenty‐three client‐owned dogs that required blood tests and insertions of PIVCs.

**Methods:** This was a randomized, prospective, method‐comparison study. Paired DV and PIVC samples were collected simultaneously from the contralateral cephalic veins. Pre‐samples (300% dead space) from PIVCs were discarded to minimize dilution effects from infusions. Hematological and biochemical analytes from different collection methods were analyzed for agreement and bias, using Bland‐Altman analysis, paired sample *t*‐test, Wilcoxon signed rank sum test and Passing‐Bablok regression analysis.

**Results:** A total of twenty‐four commonly measured laboratory analytes were evaluated respectively, including complete blood counts, leukocyte differential counts, biochemistry panels and electrolyte levels. Results showed that there was no significant difference between DV and PIVC sampling methods for the majority of analytes. Occasional differences were noted, but these were not clinically significant. Seven paired samples were lipemic; however, no statistically significant difference was identified between the two methods in these samples either.

**Conclusion:** Blood sampling from PIVCs can produce generally acceptable results and be an effective alternative to DV sampling.

## Abstract HM12: Nucleated RBCs as a Diagnostic and Prognostic Biomarker in Canine Systemic Inflammatory Response Syndrome

### **ARom Cho** ^1,2^; DoHyeon Yu^3^; Hyeona Bae^3^; Yeseul Jeon^3^; Youngju Kim^3^; Rankyung Jung^3^; Minji Kim^3^; Sumin Cha^3^; Minjeong Kang^3^

#### ^1^Gyeongsang National University, Jinju, Korea; ^2^VIP Animal Hospital, Seoul, Korea; ^3^College of Veterinary Medicine, Gyeongsang National University, Jinju, Korea

**Background:** Nucleated red blood cells (nRBCs) are immature erythrocytes that can be increased by pathologic processes as well as hematopoietic stress.

**Hypothesis/Objectives:** To evaluate the potency of nRBCs as a diagnostic and prognostic indicator in canine systemic inflammatory response syndrome (SIRS).

**Animals:** Sixty‐two client‐owned dogs met the criteria for SIRS without anemia.

**Methods:** Medical records were retrospectively reviewed. Dogs were classified into nRBC‐positive dogs (*n =* 32) and nRBC‐negative dogs (*n =* 30) based on blood films. Clinicopathological data, acute patient physiologic and laboratory evaluation (APPLEfast) score, and concentrations of cytokines including interleukin (IL)‐3, IL‐6, and erythropoietin were compared between two groups. Survival rate analysis and receiver operating characteristic curve were used to investigate the prognostic value of circulating nRBCs.

**Results:** nRBC‐positive dogs (*n =* 32) showed more severe inflammation compared to nRBC‐negative dogs (*n =* 30): higher white blood cells, neutrophils, and band neutrophils; higher plasma C‐reactive protein, and IL‐6; lower plasma albumin (all of the above, *P* < 0.05). The mean reticulocyte counts (*P* < 0.01) and concentrations of IL‐3 (*P* < 0.05) were also significantly greater in nRBC‐positive dogs. Survival time was considerably shorter in the nRBC‐positive dogs (*P* = 0.03). Circulating nRBCs at admission predicted negative outcome in dogs with SIRS at 4 weeks (area under the curve [AUC]: 0.73, confidence interval [CI]: 0.59–0.87).

**Conclusions and Clinical Importance:** Assessing peripheral nRBCs in canine SIRS is a rapid clinically‐applicable indices that reflect the severity of systemic inflammation.

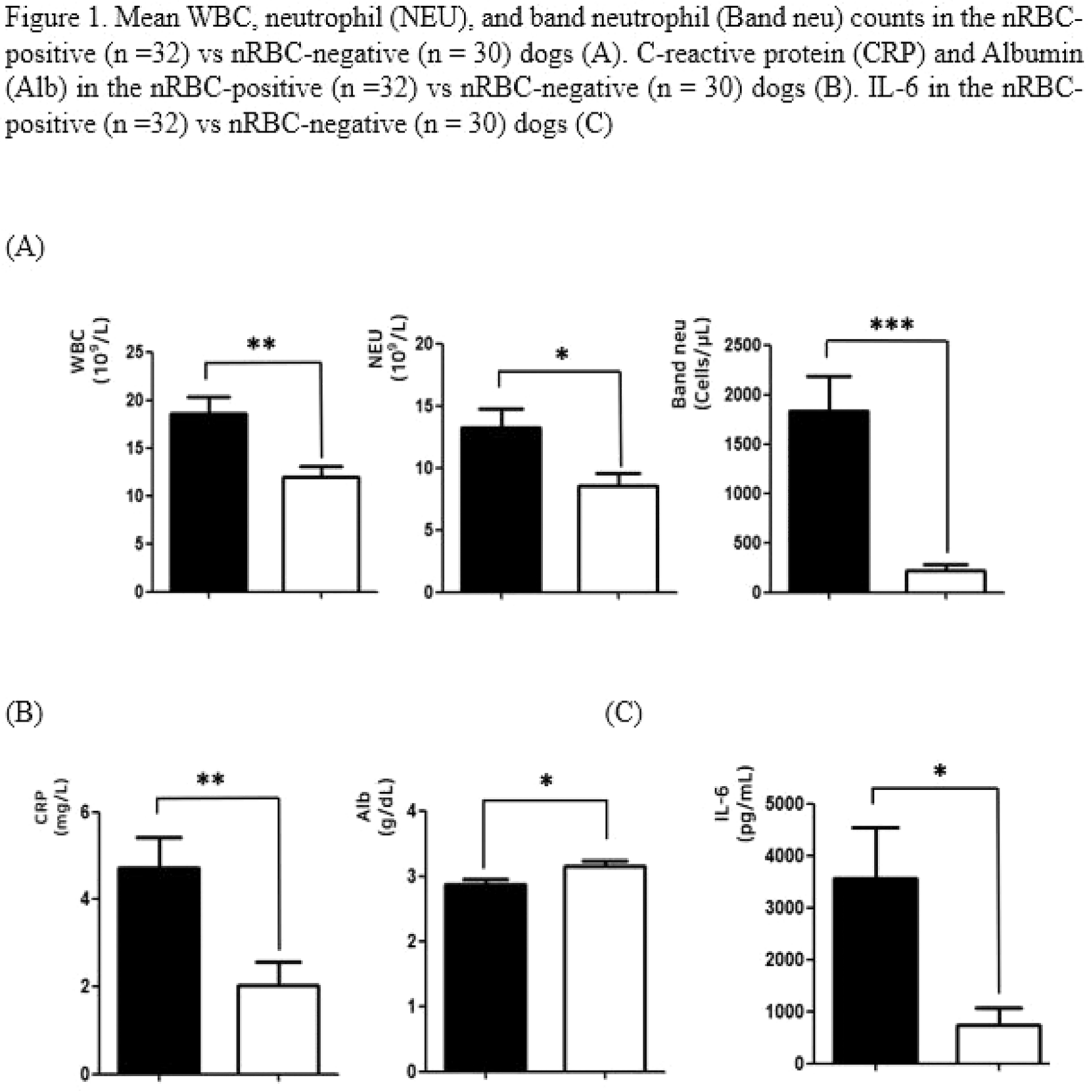

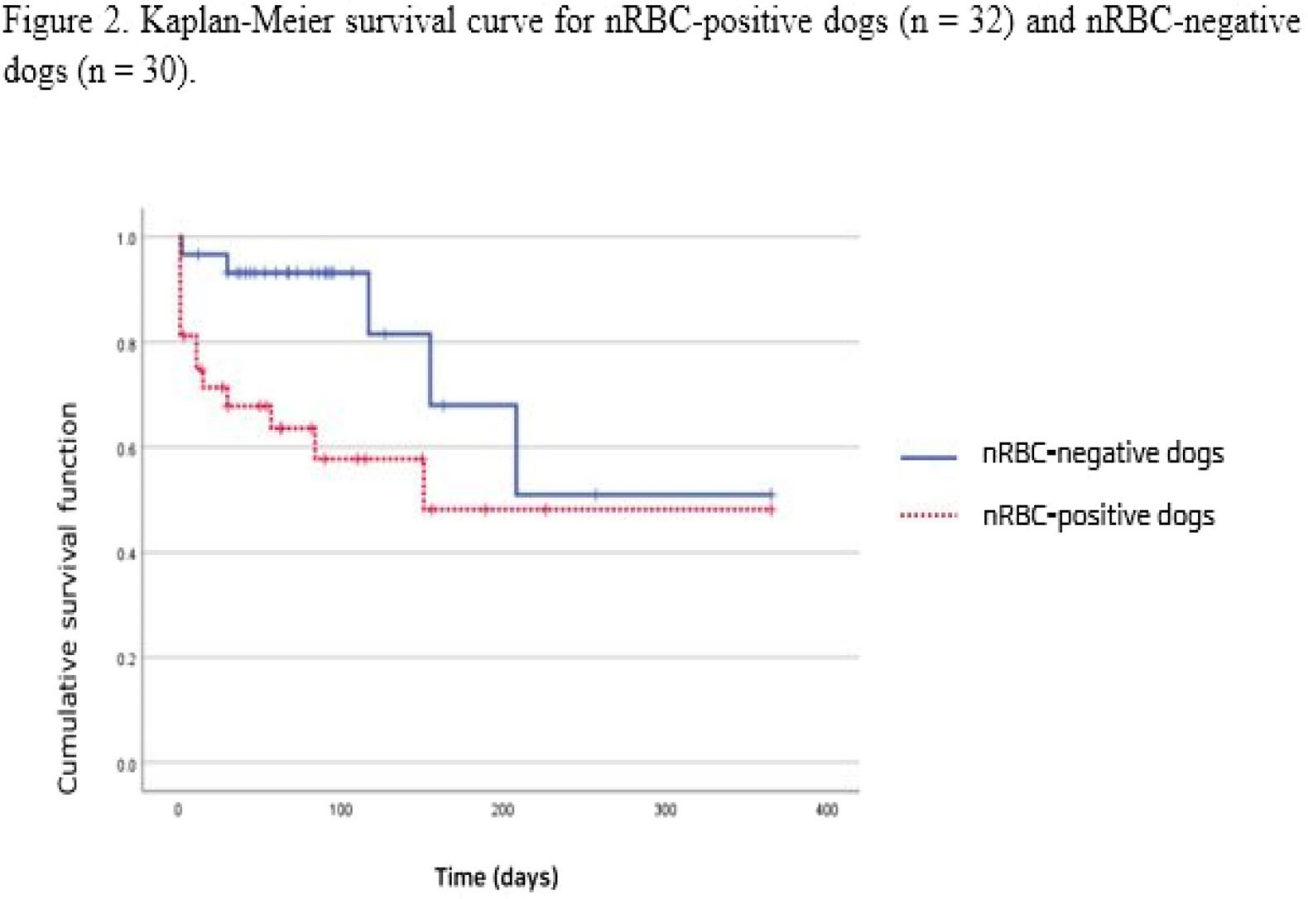

## Abstract HM13: The Evaluation of the VETSCAN IMAGYST Artificial Intelligence (AI) Blood Smear Polychromatophil Determination

### **Cory D. Penn** ^1^; Eric Morissette^2^, BSc, DVM, DACVP (Clinical); Austin Rhodes^3^, PhD

#### ^1^Medical Affairs, Global Diagnostics, Zoetis; ^2^Veterinary Clinical Pathologist, Vetscan Imagyst, Zoetis; ^3^Zoetis

**Background:** Peripheral blood film (PBF) analysis is a vital part of a comprehensive complete blood count (CBC). The VETSCAN IMAGYST AI Blood Smear Application (VS‐I) uses a deep learning artificial intelligence algorithm to evaluate blood smears and provide an estimated white blood cell count and differential, estimated platelet count, and estimated polychromatophil count for canine and feline patients.

**Hypothesis/Objectives:** The VETSCAN IMAGYST AI Blood Smear Application will correctly classify and quantify polychromatophils on blood smears, and agreement with reticulocyte counts will be appreciated in paired sample slides evaluated by American College of Veterinary Pathology (ACVP) board‐certified clinical pathologists (CP).

**Animals:** No animals were utilized in this study. Canine and feline EDTA blood samples submitted to Zoetis Reference Laboratories were used to create blood smears.

**Methods:** An incomplete block design randomly assigned PBF samples to CPs. Slides were created from each EDTA blood sample and stained by a quick‐Romanowsky type stain (Wright‐Giemsa/Diff‐Quik) and standard new methylene blue (NMB). CP‐reviewed PBF evaluations for reticulocytes using standard NMB served as gold standard and were combined statistically to provide the basis for results comparison to the VETSCAN IMAGYST AI Blood Smear Application.

**Results:** In slides stained with Wright‐Giemsa, 100% of the VS‐I evaluated slides where polychromatophils were counted were within the 95% prediction interval as calculated from the reticulocyte percentage from NMB‐stained slides.

**Conclusions and Clinical Importance:** The VETSCAN IMAGYST AI Blood Smear Application demonstrated accurate classification and quantification of polychromatophils vs. reticulocyte counts, comparable to CP confirmation of regeneration and WBC count.

## Abstract HP01: Hematological Parameters Do Not Correlate to Survival in Dogs With Multiple Acquired Portosystemic Shunts

### **Loni N. Albrecht** ^1^; Shannon Dehghanpir^2^, DVM, DACVP, MS; Andrea Johnston^3^, DVM, DACVIM (SAIM), PhD; Chin‐Chi Liu^4^, PhD, MS, MApStat

#### ^1^LSU SVM, Baton Rouge, LA, USA; ^2^Assistant Professor of Clinical Pathology, Veterinary Clinical Sciences, School of Veterinary Medicine, Louisiana State University, Baton Rouge, LA, USA; ^3^Assistant Professor, Veterinary Clinical Sciences, School of Veterinary Medicine, Louisiana State University, Baton Rouge, LA, USA; ^4^Instructor of Statistics, Veterinary Clinical Sciences, School of Veterinary Medicine, Louisiana State University, Baton Rouge, LA, USA

**Background:** Multiple acquired portosystemic shunts (MAPSS) develop secondary to portal hypertension (PH). In humans, PH has been associated with anemia, erythrocyte fragmentation, and thrombocytopenia, due to fragmentation. Hematologic abnormalities are more common and more severe in patients with advanced liver disease as opposed to patients with non‐hepatic causes of PH.

**Hypothesis/Objectives:** To determine if hematologic parameters in dogs with MAPSS are correlated to etiology or survival outcomes.

**Animals:** Thirty‐six client owned dogs diagnosed with MAPSS via ultrasound.

**Methods:** Retrospective study evaluating the hematologic data from dogs with MAPPS. The institutional Diagnostic Imaging databases were searched using key terms associated with MAPSS. Records were available from 2014 through 2022. CBC and serum chemistry results, diagnostic imaging findings, diagnosis, and survival time were recorded.

**Results:** The median age was 4 years (*n =* 36, range 0.42–13 years). The median hematocrit was 38.8% (range 16.3–64.1%), platelet count median was 172 × 103/μL (range 10–412 × 103/μL). Erythrocyte poikilocytosis included schistocytosis (15%), keratocytosis (12%), and acanthocytosis (6%). Six dogs had primary liver disease, with a median hematocrit of 35% (range 26.6–46.0%) and a median platelet count of 159 × 103/μL (range 44–275 × 103/μL). Morphologic changes of erythrocytes in this group included schistocytosis (2/6) and keratocytosis (1/6). Elevated BUN was correlated with an increased risk of death (*P* = 0.04).

**Conclusions and Clinical Importance:** Hematologic parameters were not correlated with survival and were not significantly different between dogs with and without primary liver disease.

## Abstract HP02: Liver Shear Wave Elastography Validated by Hepatic Histology in Normal Cats

### **Andrea N. Johnston** ^1^; Victoria Van^2^, DVM; Nathalie Rademacher^3^, Dr med. vet, DVM, DACVR, DECVDI; Chin‐Chi Liu^4^, PhD

#### ^1^School of Veterinary Medicine, Louisiana State University, Baton Rouge, LA, USA; ^2^Diagnostic Imaging Resident, VCS, School of Veterinary Medicine, Louisiana State University, Baton Rouge, LA, USA; ^3^Professor of Diagnostic Imaging, VCS, School of Veterinary Medicine, Louisiana State University, Baton Rouge, LA, USA; ^4^Instructor of Statistics, VCS, School of Veterinary Medicine, Louisiana State University, Baton Rouge, LA, USA

**Background:** 2D shear wave elastography (2D‐SWE) is a non‐invasive method to measure liver fibrosis.

**Hypothesis/Objectives:** The objectives of this study were trifold: (1) to assess the feasibility of performing 2D‐SWE in awake cats, (2) to determine whether anesthesia altered shear wave velocity (SWV) measurements, and (3) to correlate hepatic stiffness with histologically quantified hepatic fibrosis. We hypothesize that cats with normal hepatic histology will have a mean shear wave speed 1.197 ± 0.25 m/s, similar to humans.

**Animals:** Eleven healthy, research colony cats.

**Methods:** The study followed a prospective, observational, crossover design. Feline health status was based on physical examination, CBC, serum biochemistry, and abdominal ultrasound. Cats underwent 2D‐SWE in awake and anesthetized states. Ultrasound‐guided needle biopsies of the liver were collected during the anesthetic period. Histology samples were deemed acceptable if >15 portal tracts were present on H&E stained formalin fixed paraffin embedded (FFPE) histologic sections. Hepatic fibrosis was quantified using QuPath software on digitized Masson's trichrome stained FFPE histology slides.

**Results:** The mean velocities were not significantly different between awake (1.47 ± 0.18) and anesthetized cats (1.47 ± 0.24 m/s). There was a greater variability in the SWV values in the awake group. All cats had clinically insignificant fibrosis, and there was no correlation between the SWV measurements and the quantity of histological fibrosis.

**Conclusions and Clinical Importance:** 2D‐SWE is feasible in cats. Anesthesia did not impact mean SWV. 2D‐SWE cannot detect minimal differences in hepatic fibrosis in healthy cats.

## Abstract HP03: Canine Patient‐derived Gallbladder Organoids for Personalized Gallbladder Mucocele Modeling and Drug Screening

### **Itsuma Nagao** ^1,2^; Yoko Ambrosini^1^

#### ^1^Washington State University, Pullman, WA, USA; ^2^The University of Tokyo, Tokyo, Japan

**Background:** Gallbladder mucocele (GBM) is one of the most common diseases of the gallbladder in dogs. The pathogenesis of GBM is unclear due to lack of appropriate *in vitro* models. Recently, organoid technology has been applied in the gallbladder from humans, mice, and pigs as a novel *in vitro* disease model.

**Hypothesis/Objectives:** To develop, characterize, and assess the functions of a gallbladder organoid (GBO) in dogs with GBM.

**Animals:** GBOs were derived from gallbladder tissues isolated from one client‐owned dog undergoing cholecystectomy.

**Methods:** Surgically removed gallbladder was treated with trypsin to collect stem cells, which were then embedded in Matrigel to form GBOs. Phase‐contrast imaging and immunofluorescence (IF) were utilized to evaluate the size, morphology, and protein expression in GBOs. The function of cystic fibrosis transmembrane conductance regulator (CFTR), an ion channel mainly expressed in the gallbladder, was examined using Forskolin‐induced swelling assay. Results were evaluated by Mann–Whitney *U* test.

**Results:** Successful GBO development and maintenance were achieved. Phase‐contrast imaging demonstrated the budding structure of the organoids which were similar to what has been reported with the organoids from cystic fibrosis. IF staining revealed expression of epithelial cell marker and tight junction proteins. Furthermore, the Forskolin‐induced swelling assay demonstrated that GBOs hold functional CFTR (2.2‐fold swelling after 2 h; *P* < 0.01).

**Conclusions and Clinical Importance:** We demonstrated a successful development of GBOs from a dog with GBM. Our novel GBO model can serve as a useful *ex vivo* system for GBM pathophysiology investigation as well as pharmaceutical drug transport studies.

## Abstract HP04: Serial Quantification of Serum MicroRNA 122 in Dogs With Acute Liver Injury

### **Adrian Tinoco Najera**; Bruna Correa‐Lopes; Chi‐Hsuan Sung; Yuri Lawrence; Joerg Steiner; Jan Suchodolski; Jonathan Lidbury

#### Gastrointestinal Laboratory, Texas A&M University, College Station, TX, USA

**Background:** MicroRNA 122 (miR‐122) has been shown to be a potential biomarker for liver injury in dogs. However, changes in serum miR‐122 in dogs with acute liver injury (ALI) over time have not previously been reported.

**Objective:** To serially quantify miR‐122 in serum from dogs with ALI during the first 48 h of hospitalization.

**Animals:** Serum samples from 10 dogs with ALI were collected at baseline (T0), 24 h (T1), and 48 h (T2) after hospitalization. Serum samples from 10 healthy dogs were used as controls.

**Methods:** Prospective longitudinal study. MiR‐122 was quantified by RT‐qPCR. MicroRNA 26b was used as housekeeping gene. Serum ALT activities were concurrently measured. Kruskal‐Wallis tests were used to compare baseline miR‐122 gene‐fold expression and ALT activity in dogs with ALI versus those of healthy controls. Friedman tests were used to assess changes in miR‐122 and ALT over time. Spearman's correlation was used to evaluate the relationship between miR‐122 and ALT. Statistical significance was set at *P* < 0.05.

**Results:** At T0 miR‐122 expression (*P* < 0.001) and ALT activity (*P* < 0.001) of dogs with ALI were higher than those of healthy controls. MiR‐122 expression (*P* = 0.23) and ALT activity (*P* = 0.11) did not significantly change over time (Table 1). There was a positive correlation between miR‐122 and ALT (*P* < 0.001; *r =* 0.76).

**Conclusions and Clinical Importance:** Although, at the time of hospitalization serum miR‐122 expression was increased in dogs with ALI, studies of longer duration are needed to determine if this marker is useful for monitoring changes in liver injury over time.

**Table:** Table 1. Serum ALT concentrations and miR‐122 serum gene‐fold expressions of dogs with acute liver injury

Variable	Healthy controls median [range]	ALI ‐ T0 median [range]	ALI ‐ T1 median [range]	ALI ‐ T2 median [range]
ALT (U/L)	55 [38–103]	3,186 [358–8,250]	1,775 [341–7,500]	1,212 [253–7,837]
miR‐122 (GFE)	0.7 [0.2–11.6]	92 [4–1,969]	90 [1–1,969]	13 [1–145]

Footnotes: ALT = Alanine aminotransferase, ALI = Acute liver injury, GFE = Gene‐fold expression, T_0_ = baseline, *T*_1_ = 24 h, *T*_2_ = 48 h, U/L = Units per litre.

## Abstract HP05: Defining the Liver Transcriptome in Cats Using Total RNA Sequencing: A Pilot Study

### **Rommaneeya Leela‐arporn** ^1^; Lyndah Chow^2^, PhD; Sarah Shropshire^3^, DVM, PhD, DACVIM (SAIM); Steven Dow^4^, DVM, PhD, DACVIM (SAIM); Catriona MacPhail^4^, DVM, PhD, DACVS; Michael Lappin^4^, DVM, PhD, DACVIM (SAIM)

#### ^1^Colorado State University, Fort Collins, CO, USA; ^2^Research Scientist, Clinical Sciences, Colorado State University, Fort Collins, CO, USA; ^3^Assistant Professor, Clinical Sciences, Colorado State University, Fort Collins, CO, USA; ^4^Professor, Clinical Sciences, Colorado State University

**Background:** Cats develop a variety of idiopathic, inflammatory, and neoplastic diseases of the liver. Defining the liver transcriptome could be beneficial in identifying gene expression patterns that are associated with different diseases. The normal liver transcriptome in cats has not been previously investigated.

**Hypothesis/Objectives:** To define the hepatic transcriptome of cats using total RNA sequencing of liver biopsy samples from healthy animals.

**Animals:** Two healthy purpose‐bred adult cats.

**Methods:** Surgical liver biopsies were obtained during routine ovariectomy. RNA was extracted and subjected to Illumina sequencing. Sequence files were aligned with the reference cat genome and assembled using Partek flow software. The top 250 expressed genes were identified along with their function, and pathway analysis was performed using open‐source software.

**Results:** A total of 19,565 protein coding genes were sequenced. The most highly expressed genes included albumin, apolipoprotein, haptoglobin, and serotransferrin. Of the 100 most expressed genes, 27% were metabolite interconversion enzymes, with the second most abundant category being chromatin/chromatin‐binding, or ‐regulatory proteins (14.4%) followed by 8% of genes classified as protein‐modifying enzymes. Other highly expressed genes were classified as metabolite interconversion enzymes, protein‐modifying enzymes, and translational proteins. The most highly expressed pathways were those associated with metabolic processes.

**Conclusions and Clinical Importance:** These studies provided unique new insights into the normal hepatic transcriptome in cats, and the data provides an important resource for helping to identify abnormal pathways in cats with hepatic diseases. This database can also assist with the design of targeted gene expression analysis tools.

## Abstract HP06: Concurrent Hepatopathy in Dogs With Gallbladder Mucocele: Prevalence, Predictors, and Impact on Long‐Term Outcome

### **Sara Jablonski** ^1^; Polly Chen^1^; Jessica Kendziorski^1^; Jarod Williams^2^; Rebecca Smedley^1^

#### ^1^College of Veterinary Medicine, Michigan State University, East Lansing, MI, USA; ^2^Ozark Veterinary Specialty Care, Springdale, AR, USA

**Background:** Information is limited regarding the prevalence and importance of hepatic histologic abnormalities in dogs with gallbladder mucocele (GBM).

**Objectives:** To (1) report the prevalence of hepatic histologic abnormalities in GBM dogs, (2) evaluate for an association between hepatic abnormalities and outcome in GBM dogs, (3) evaluate whether the neutrophil‐to‐lymphocyte ratio (NLR) differs in GBM dogs with and without hepatic inflammatory lesions.

**Animals:** 52 dogs with grossly and histologically confirmed GBM.

**Methods:** Multicenter, retrospective study of GBM dogs undergoing cholecystectomy with concurrent liver biopsy. Archived histological sections of gallbladder and liver evaluated by a single blinded pathologist and pathologist‐in‐training. Proportions of dogs with each histologic abnormality alive vs. deceased at 1, 3 and 12 months post‐cholecystectomy were compared. Mann‐Whitney U was performed to determine if NLR differed in dogs with or without selected lesions.

**Results:** 51/52 (98%) dogs with GBM had at least one hepatic histologic abnormality. Hepatic fibrosis (30/52; 58%), biliary hyperplasia (29/52; 56%), and portal inflammation (25/52; 48%) were most common. Dogs with higher portal fibrosis scores were more likely to be deceased at 12 months compared to dogs with lower portal fibrosis scores (*P* = 0.03). Dogs with hepatic necrosis (*P* = 0.006) and cholangitis (*P* = 0.02) had higher NLRs compared to dogs without these lesions.

**Conclusions and Clinical Importance:** Histologic abnormalities of the liver are common in GBM dogs. A higher portal fibrosis score might be associated with decreased survival at 12 months post‐cholecystectomy for GBM. An increase in NLR might predict hepatic necrosis and cholangitis in GBM dogs.

## Abstract HP07: Correlation of Ultrasound Measures With Liver Volume Assessed by Water Displacement in Dogs

### **Fabio A. Teixeira** ^1,2^; Mariana Pacheco^1^; Mariana Queiroz^1^; Stefano Carlo Hagen^1^

#### ^1^School of Veterinary Medicine and Animal Science, University of Sao Paulo, Sao Paulo, SP, Brazil; ^2^Nutricare Vet

**Background:** Ultrasound evaluation of the liver volume can be an important information about dogs with suspected hepatopathy. There are few data regarding estimation of canine liver volume by ultrasonography.

**Objectives:** To obtain a method of measuring liver volume (mL) by ultrasonography.

**Animals:** 82 cadavers of dogs of various ages and breeds after recent natural death were used.

**Methods:** Morphological external measures [chest perimeter (PERI, cm); chest height (H, cm) and chest width (WC)], body weight (BW, kg) and two linear ultrasound liver measures [A (from the beginning of the hepatic parenchyma to the diaphragm tangent to the neck of the gallbladder) and E (from the caudal border of the liver to the diaphragm, measured on the midline)], both in cm, were performed three times by two veterinary sonographers. Liver volume was determined by water displacement method. Linear regression was used to correlate liver volume and measurements.

**Results:** The ultrasound measurements proved to be reproducible inter‐ and intra‐observer. Three equations were obtained: 639 + 10BW + 75A − 55H − 23PERI + 1.4H × PERI (adjusted *r* ^2^=0.81; *P* < 0.01); 822 + 14BW + 59E − 59H − 28PERI + 1.7H × PERI (adjusted *r* ^2^=0.83; *P* < 0.01); 30 + 0,3H × WC × A (adjusted *r* ^2^=0,88; *P* < 0,01).

**Conclusions and Clinical Importance:** The association of liver ultrasound measures with body weight, chest perimeter, chest height and chest width provided strong correlations with the liver volume and can be useful to clinical routine.

## Abstract HP08: Comparison of 2‐Dimensional Imaging to 3‐Dimensional Modeling of Intrahepatic Portosystemic Shunts Using Computed Tomography Angiography

### **Erin E. Jackson** ^1^; Lauren Markovic^2^, DVM, DACVIM (Cardiology); Michael Perlini^3^, DVM, DACVR, DACVR‐EDI

#### ^1^College of Veterinary Medicine, University of Georgia, Athens, GA, USA; ^2^Assistant Professor, Department of Small Animal Medicine and Surgery, College of Veterinary Medicine, University of Georgia, Athens, GA, USA; ^3^Assistant Professor, Department of Large Animal Medicine, College of Veterinary Medicine, University of Georgia, Athens, GA, USA

**Background:** Computed tomography angiography (CTA) is used to help diagnose intrahepatic portosystemic shunts (IHPSS). Caudal vena cava (CVC) measurements are typically obtained from 2‐dimensional (2D) imaging to aid in stent selection for transcatheter intervention.

**Hypothesis/Objectives:** 3‐dimensional (3D) vascular models can be generated for IHPSS, and CVC measurements will not differ significantly between 2D images and 3D models.

**Animals:** CTA datasets of IHPSS from client‐owned dogs at University of Georgia between 2016–2022.

**Methods:** Materialize Mimics 25.0 and 3‐matic 17.0 were used for 3D modeling. CVC diameters were measured in 2D coronal and axial planes 20 mm cranial and caudal from the shunt ostium and were compared to CVC diameters from 3D models. Length was measured in the 2D coronal plane between midpoints of each diameter and compared to 3D length. Data are presented as mean (standard deviation). Intra‐class correlation coefficients (ICC) were performed.

**Results:** 3D models were generated for 32 IHPSS (15 right‐, 12 left‐, and 5 central‐divisional). 2D coronal and axial diameter measurements were 16.7 mm (5.6) and 15.5 mm (4.2) cranial, 14.9 mm (4.2) and 14.3 mm (3.7) caudal. 3D diameter measurements were 15.3 mm (4.4) cranial and 14.0 mm (3.6) caudal. 2D length was 61.5 mm (7.1) compared to 3D 59.9 mm (7.2). ICCs comparing 2D and 3D diameters were all >0.80, indicating very good agreement, with good agreement between 2D and 3D length.

**Conclusions and Clinical Importance:** 3D IHPSS models can be generated using modeling software, and measurements from 3D models are consistent with 2D planar imaging. 3D IHPSS models are accurate representations of patient‐specific disease. Future directions include utilizing models for pre‐procedural planning.

## Abstract ID01: Canine Cholecystitis Secondary to *Hammondia* spp. Tachyzoite Infection

### **Skyler H. Caldwell**; Richard Gerhold, DVM, MS, PhD, DACVM (Parasitology); Ashley Hartley, DVM, PhD, DACVIM (SAIM); Lisa Neufang, DVM; Jake Salzman, DVM; Jillian Smith, DVM, DACVIM (SAIM); Nora Springer, DVM, PhD, DACVP; Chunlei Su, DVM, MS, PhD; Eliza Baker, DVM

#### College of Veterinary Medicine, University of Tennessee, Knoxville, TN, USA

**Background:** Biliary protozoal infections are rare in dogs. Sarcocystidae protozoal infections (i.e., *Toxoplasma*, *Neospora*, *Hammondia* spp., etc.) are uncommon and host‐specific. Diagnosis is traditionally dependent on tissue or serologic evaluation; sensitivity and specificity for these techniques can be variable. PCR amplification and genetic sequencing provide a technique to expedite diagnosis and speciation in clinical biliary protozoal infections.

**Objectives:** To characterize a series of canine protozoal cholecystitis cases.

**Animals:** Two dogs with biliary protozoal infections consistent with *Hammondia* spp.

**Methods:** Limited case series of two dogs presenting with mixed hepatopathies and hyperbilirubinemia. Biliary tachyzoites were identified via cholecystocentesis cytology during diagnostic investigations. Bile aerobic and anaerobic culture, Sarcocystidae serology, and protozoal gene PCR amplification were completed.

**Results:** Crescent‐shaped protozoal organisms (4–5 μm × 0.5 μm) consistent with tachyzoites were identified on bile cytology in both dogs. Bile aerobic and anaerobic cultures would have no growth. Sera were positive for anti‐*Toxoplasma* sp. (*n =* 1/2) and anti‐*Neospora* sp. (*n =* 2/2) antibodies. Genomic isolation from bile followed by pan‐Sarcocystidae (18S rRNA) PCR amplification were consistent with *Hammondia* spp. Subsequent cytochrome b gene‐specific amplification (384 bp fragment) and sequencing in one of the samples revealed 99.7% similarity to *Hammondia triffittae* versus 98.9% to *Hammondia heydorni*. Treatment with clindamycin and fluoroquinolone resolved clinical and clinicopathologic signs.

**Conclusions and Clinical Importance:** Molecular techniques are key adjunctive techniques for protozoal identification in rarer infections. This appears to be the first report of biliary infection with *Hammondia triffittae*, a protozoan with a definitive fox host and not dogs.

## Abstract ID02: Detection of Carbapenemase‐producing *E. coli* Through a Veterinary‐Public Health Surveillance Partnership—Minnesota, 2022

### **Jeff Bender** ^1^; Amanda Beaudoin^2^; Melissa Boyd^1^; Jennifer Granick^1^; Karen Olsen^1^; Kim Little^1^; Leslie Kollmann^3^; Paula Snippes Vagnone^3^

#### ^1^University of Minnesota, Minneapolis, MN, USA; ^2^College of Veterinary Medicine, University of Minnesota, Minneapolis, MN, USA; ^3^Minnesota Department of Health, St. Paul, MN, USA

**Background:** Carbapenemase‐producing Enterobacterales (CPE) are of urgent public health concern, with resistance to most antibiotics, including carbapenems. Major USA risk factors for human CPE colonization or infection include exposure to antibiotics and healthcare, especially hospitalization abroad. Detection of infection, colonization, and transmission in veterinary settings is challenging. Awareness is low and antibiotic susceptibility testing panels are often too narrow for detection.

**Objectives:** The Minnesota Department of Health (MDH) and University of Minnesota (UMN) conduct collaborative surveillance and response for CPE in companion animals.

**Animals:** UMN companion animal patients.

**Methods:** In 2020, MDH and UMN initiated collaborative surveillance to detect CPE in companion animal patients. Initially, carbapenem‐resistant Enterobacterales (CRE) isolates were sent by UMN to MDH for carbapenemase testing by modified carbapenem‐inhibition method (mCIM) and PCR. In March 2022, UMN implemented expanded susceptibility testing for Enterobacterales and *Pseudomonas*, with in‐house mCIM testing.

**Results:** In February 2022, carbapenemase‐producing *E. coli* was isolated from a dog of international origin. MDH and UMN worked together to investigate the potential for in‐hospital transmission, including review of epidemiologically linked patients for screening and assessment of infection prevention and control (IPC). During March–November 2022, 291 Enterobacterales and *Pseudomonas* isolates underwent enhanced susceptibility testing at UMN. Three isolates reflexed to mCIM testing, but no additional carbapenemase‐producing organisms have been detected.

**conclusions/clinical importance:** Collaborative surveillance allowed UMN to quickly detect CPE in a canine patient. Expansion of in‐house susceptibility, mCIM testing, and review of admission and IPC protocols has positioned UMN to respond quickly and prevent CPE transmission.

## Abstract ID03: Enterohemorrhagic *Escherichia coli* Reduces Mucus and Barrier Integrity in Translational Canine Colonoid‐Derived Monolayers

### **Meg Nakazawa**; Minae Kawasaki; Yoko Ambrosini

#### Washington State University, Pullman, WA, USA

**Background:** Enterohemorrhagic *E. coli* (EHEC) can cause acute colitis in dogs, which can lead to hemorrhagic diarrhea and a hemolytic uremic syndrome similar to those in humans. Understanding of the pathophysiology and treatment recommendations are hampered by the lack of translatable *in vitro* models which effectively reproduce canine and human clinical disease induced by EHEC.

**Objectives:** To investigate the effects of EHEC on canine intestinal epithelium using organoid‐derived monolayers.

Animals and **Methods:** Colonoid‐derived monolayers were developed using intestinal tissues biopsied from two healthy client‐owned dogs. The monolayers were challenged with EHEC at 1 × 106 CFU/ml for 24 h and impacts of EHEC on epithelial cells were evaluated by trans‐epithelial electrical resistance (TEER) measurement, immunofluorescence (IF) staining and scanning electron microscopy (SEM). The results were analyzed using Student t‐tests.

**Results:** The TEER declined significantly in EHEC‐infected monolayers after 12 h compared with that noted in the controls (‐24.2% vs. −6.3%; *P* < 0.05). An impact of EHEC on the cellular integrity was confirmed by 1.8‐fold increase in E‐cadherin expression per unit area with IF staining (*P* < 0.01). WGA stain confirmed significantly decreased mucus on the EHEC‐exposed epithelial surface (−18%, *P* < 0.05). Attaching/effacing lesions characteristic of EHEC infection were confirmed by SEM imaging.

**Conclusions and Clinical Importance:** The results recapitulated *in vivo* observations reported in dogs and humans with EHEC enteropathy, supporting that the canine colonoid‐derived monolayer system can serve as a useful translational model to assess the host‐pathogen interaction upon exposure to clinically important enteric pathogens such as EHEC.

## Abstract ID04: Flea‐Borne Pathogens in Fleas From Naturally Infested Dogs and Cats in Private Homes in Florida

### **Erin Lashnits** ^1^; Taylor Gin^2^; Amiah Gray^3^; Yiyao Li^1^; Kamilyah Miller^3^; Cameron Sutherland^3^; Trey Tomlinson^3^; Grace Wilson^3^; Michael Canfield^4^; Brian Herrin^3^

#### ^1^School of Veterinary Medicine, University of Wisconsin, Madison, WI, USA; ^2^North Carolina State University, Raleigh, NC, USA; ^3^College of Veterinary Medicine, Kansas State University, Manhattan, KS, USA; ^4^Animal Dermatology South, New Port Richey, FL, USA

**Background:** The cat flea, *Ctenocephalides felis*, is the most common ectoparasite of dogs and cats, and can transmit a variety of pathogens including zoonotic *Bartonella* and *Rickettsia* species. The risk factors underlying transmission of these pathogens are incompletely elucidated.

**Objective:** Describe the flea‐borne pathogens of fleas from owned cats and dogs and determine associations between flea pathogen carriage and pet and household characteristics.

**Animals:** 32 homes in west central Florida with flea infestations, including 40 cats and 8 dogs.

**Methods:** Fleas on each cat and dog were counted using a standardized procedure, then collected; fleas in the home were also counted using overnight intermittent light traps, then collected. A survey was used to gather demographic and household information as potential explanatory variables. Fleas were pooled by animal and tested using 16S‐rRNA next generation sequencing. Associations between the presence of *Bartonella* and *Rickettsia* spp. in fleas with potential explanatory variables were assessed using mixed effects modeling.

**Results:** There were 272 fleas collected from 40 cats in 31 homes, and 98 fleas from 8 dogs in 7 homes. Pathogens in fleas and the proportion of cats and dogs with fleas infected with each pathogen will be presented. Associations between the presence of *Bartonella* and *Rickettsia* spp. and potential explanatory variables, including host demographics, home characteristics, and geographic location, will be shown.

**Conclusions:** This study evaluated flea‐borne pathogens in fleas from owned pets in their homes, reflecting potential flea‐borne disease exposures for pets and pet owners with limited access to veterinary care.

## Abstract ID05: Frequency of Intestinal Parasites in Dogs and Cats Identified by Molecular Diagnostics

### **Christian M. Leutenegger**; Christian Savard; Michelle Evason; Jennifer Willcox; Haresh Rochani; Holly Richmond; Cecilia Lozoya; Jeffrey Tereski; Samantha Loo; Jan Andrews; Kelly Mitchell

#### Antech Diagnostics, Inc.

**Background:** Molecular diagnostic tests using real‐time PCR (qPCR) broaden the capacity for gastrointestinal (GI) parasite detection as compared to more traditional fecal testing. Additionally, the qPCR panel identifies genetic markers conferring anthelmintic resistance and identifies potentially zoonotic *Giardia* assemblages.

**Objective:** To determine regional and seasonal distribution of 20 intestinal parasites, benzimidazole resistance, and *Giardia* with zoonotic potential, from North America (NA).

**samples:** Sample set consisted of 315,953 fecals submitted to a reference laboratory for KeyScreen GI parasite PCR panel testing between March and December 2022.

**Methods:** Samples underwent nucleic acid extraction and qPCR analysis to identify 20 common GI parasites in cats and dogs, the genetic marker for *A. caninum* benzimidazole resistance (F167Y), and assemblage A/B *Giardia duodenalis* conferring zoonotic potential.

**Results:** The majority of submissions, 83.1% (262,641), were from dogs, with 16.9% (53,312) from cats. One or more parasites were detected in 25.4% (80,150/315,953); 26.3% (68,985/262,641) in dogs and 21.1% (11,232/53,312) in cats. Co‐infections were detected in 20.5% of samples with parasites (16,431/80,150). *A. caninum* benzimidazole resistance frequency was 11.2% (731/6,538) of canine samples, 11.3% in the USA (727/6,437), and 4.0% in Canada (4/101). *G. duodenalis* was detected in 13.4% of samples (42,238/315,953); 3.8% (1,610/42,238) of which belonged to the potentially zoonotic assemblages.

**Conclusions and Clinical Importance:** These data provide a parasite frequency update and emphasize the importance of routine GI parasite screening with a broad parasite panel. The presence of anthelmintic resistance and *G. duodenalis* assemblages with zoonotic potential highlight antimicrobial stewardship and One Health considerations.

## Abstract ID06: Sites of Feline Coronavirus Persistence in Cats

### **Beth N. Licitra**; Nicole Andre; Ximena Olarte Castillo; Gary Whittaker

#### Cornell University, Ithaca, NY, USA

**Background:** Feline Coronavirus (FCoV) infection is known to result in persistent infection and shedding in 10–15% of cats. However, the sites of persistent FCoV infection and their role in the pathogenesis of disease is poorly understood.

**Objectives:** This study seeks to identify reservoirs of coronavirus persistence in cats and to characterize the nature of the viral spike protein mutations that are linked to pathogenesis and transmission.

**Animals:** Six animals enrolled in a pilot study were included in this analysis. They were drawn from a population of client‐owned animals with a history of FCoV infection, and included classic cases of FIP as well as those with a less traditional course of disease; including cats on antiviral treatment, cats with chronic viral shedding and non‐specific disease signs, and a case of spontaneous FIP remission where the cat developed alimentary lymphoma later in life.

**Methods:** Feces, blood, effusion, and tissue was sampled ante‐mortem. RNA *in situ* hybridization of tissues collected at necropsy was used to identify viral reservoirs, together with amplicon‐based hybrid capture sequencing protocol for the spike gene.

**Results:** FCoV was identified in multiple tissues including in heart, eye, and intestine. Sequence analysis identified mutations associated with FIP in all cats in the study, including two that were diagnosed with alimentary lymphoma.

**Conclusions:** This case series sheds light on the nature of persistent coronavirus infection in cats. An understanding FCoV infection and its sites of persistence is important for viral testing and appropriate use of antiviral drugs.

## Abstract ID07: Outcomes of Naturally Occurring Canine Heartworm Infection

### **JoAnn Morrison** ^1^; Chris Adolph^2^; Kennedy Mwacalimba^2^, Kristine Smith^2^

#### ^1^Banfield Pet Hospital; ^2^Zoetis Animal Health

**Background:** Canine heartworm disease continues to be diagnosed in the United States, with positive tests numbering more than 100 000/year (according to the Companion Animal Parasite Council) nationally. To date, much of the peer reviewed literature on canine heartworm infection has focused on epidemiologic and prevalence data, severe clinical presentations, and parasite resistance.

**Hypothesis/Objectives:** The objective of this study was to examine longitudinal outcomes in dogs that tested positive and underwent therapy for heartworm infection within primary care hospitals.

**Animals:** Privately owned dogs presenting to Banfield Pet Hospital were included in the study. Dogs testing positive for heartworms (via antigen test) were identified as cases and a population of negative controls was identified.

**Methods:** Retrospective, case‐control study with dogs identified via medical record search. In this system, longitudinal data is available for individual dogs, allowing long‐term analysis. Analysis on structured diagnostic codes pertaining to right and left sided cardiac disease and relative risk of outcomes was performed.

**Results:** Dogs positive for heartworm infection were found to have increased relative risk (RR) for right heart failure (RR 3.59, 95% C.I. 2.64–4.86), left heart failure (1.83, 95% C.I. 1.51–2.22), and cardiomyopathy (2.79, 95% C.I. 1.71–4.57) compared to negative controls. All *P* values were significant at *P* < 0.0001 (Table 1).

**Conclusions and Clinical Importance:** This analysis describes the longitudinal clinical outcomes of dogs with heartworm infections and provides data driven evidence to demonstrate the importance of disease prevention.

**Table:** Table 1. Outcome statistics

	Exposure			Non‐exposure			Results			
Outcome of interest	Num	Denom	%	Num	Denom	%	RR	Lower Cl	Upper Cl	*P* value
Right heart failure	42	6,138	0.68%	7,639	4,022,752	0.19%	3.59	2.64	4.86	<0.0001
Left heart failure	105	6,138	1.71%	37,280	4,022,752	0.93%	1.83	1.51	2.22	<0.0001
Cardiomyopathy	16	6,138	0.26%	3,746	4,022,752	0.09%	2.79	1.71	4.57	<0.0001

Legend: Num = numerator; Deno*n =* denominator; R*R =* relative risk; CI = confidence interval

## Abstract ID08: Can We Predict Xanthinuria in Dogs with Leishmaniasis Undergoing Allopurinol Treatment? ‐ A Retrospective Study

### **Rodolfo Oliveira Leal** ^1,2^; Sara Oliveira^3^; Maria Joana Dias^1,2^; Eva Prosper^4^; Carolina Arenas^5^; Marina Domínguez‐Ruiz^6^

#### ^1^CIISA – Centre for Interdisciplinary Research in Animal Health, Faculty of Veterinary Medicine, University of Lisbon, Lisbon, Portugal; ^2^Associate Laboratory for Animal and Veterinary Sciences (AL4AnimalS), Caparica, Portugal; ^3^Veterinary Teaching Hospital, Faculty of Veterinary Medicine, University of Lisbon, Lisbon, Portugal; ^4^Anicura Hospital Veterinario Valencia, Valencia, Spain; ^5^Anicura Hospital Veterinario Valencia Sur, Valencia, Spain; ^6^Hospital Clinico Veterinario, Universidad Alfonso X El Sabio, Madrid, Spain

**Background:** Xanthinuria is a common but inconsistent side‐effect in dogs with leishmaniosis undergoing allopurinol treatment.

**Hypothesis/Objectives:** To evaluate potential predisposing factors to the development of xanthinuria in dogs with leishmaniosis treated with allopurinol.

**Animals:** Ninety dogs with leishmaniosis undergoing allopurinol treatment, recruited from three referral hospitals (one in Portugal and two in Spain) between 2012 and 2022.

**Methods:** A multicentric, retrospective, case‐control study was conducted. Dogs with leishmaniosis that developed xanthinuria (crystals and/or uroliths) undergoing allopurinol treatment were selected and grouped (Xgroup). A control group consisting of dogs with leishmaniosis without xanthinuria after at least five months of allopurinol treatment was concurrently recruited (NXgroup). Medical records were reviewed, and clinical and laboratorial variables were compared between groups. Descriptive and non‐parametric tests were used (*P* < 0.05).

**Results:** Forty‐five dogs were included in each group. Groups were homogeneous for gender, weight, and reproductive status. Only age and serum alpha‐1 globulins concentration were significantly different between groups. Dogs from Xgroup were younger than those from the NXgroup (median 4 years (range 1–10) versus 6 years (range 1–14), respectively; *P* = 0.002). Before allopurinol onset, a higher percentage of dogs from the NXgroup had decreased serum alpha‐1 globulin levels (40.5% versus 13.3% in the Xgroup, respectively; *P* = 0.016). In Xgroup, median time to xanthinuria development after starting allopurinol was 150 days (range 4–2190).

**Conclusions and Clinical Importance:** This study highlights that young‐adult age and normal serum alpha‐1 globulin levels (before treatment) might be predisposing factors for the development of xanthinuria in dogs with leishmaniosis undergoing allopurinol treatment.

## Abstract ID09: Trends and Risk Factors for Multidrug‐Resistant Canine Urinary Tract Infections (2009–2013; 2017–2021)

### **Víctor A. Oppenheimer Lúgaro** ^1^; Hsin‐Yi Weng, BVM, MPH, PhD^2^; Timothy Hui, DVM, DACVIM (SAIM)^3^; Lynn Guptill^4^, DVM, PhD, DACVIM (SAIM)

#### ^1^Purdue Veterinary Teaching Hospital, West Lafayette, IN, USA; ^2^Associate Professor/Clinical and Analytical Epidemiology, Department of Comparative Pathobiology, College of Veterinary Medicine, Purdue University, West Lafayette, IN, USA; ^3^Internist, Small Animal Internal Medicine, VCA Advanced Veterinary Care Center; ^4^Associate Professor/Internist, Department of Veterinary Clinical Sciences, College of Veterinary Medicine, Purdue University, West Lafayette, IN, USA

**Background:** Recent evidence suggests the proportion of multidrug‐resistant (MDR) urinary tract infections (UTIs) in dogs is increasing.

**Objective:** To characterize antimicrobial resistance trends in canine UTIs and determine risk factors associated with development of MDR UTIs.

**Animals:** 1,106 bacterial isolates from 759 dogs treated at a veterinary teaching hospital.

**Methods:** Isolate susceptibility data from urine cultures were retrospectively evaluated over 2 time periods (2009–2013; 2017–2021). Information extracted from medical records included signalment, urinalysis findings, classification of sporadic or recurring UTI, presence of lower urinary tract signs, urolithiasis, neoplasia, chronic kidney disease, endocrinopathies, urinary tract anatomical disorders, micturition disorders, previous antimicrobial or immunosuppressive drug use, and history of indwelling catheterization. Chi‐square tests and random‐effect logistic regression were used to assess temporal changes and risk factors.

**Results:** There were increases in MDR isolates between the two time periods (*P* < 0.001) and extensively‐drug resistant (XDR) isolates from 2017–2021 (*P* < 0.001). Common isolates included *Escherichia coli* (40.1%), *Staphylococcus* spp. (14.3%), and *Enterococcus* spp. (12.9%), all with an increasing trend in antimicrobial resistance from 2009–2021 (*P* < 0.001). Significant risk factors for MDR UTIs included previous antimicrobial and immunosuppressive therapy (*P* < 0.001 and *P* = 0.044 respectively), micturition disorders (*P* = 0.005), and previous catheterization (*P* < 0.001).

**Conclusions and Clinical Importance:** The increase in MDR/XDR UTIs emphasizes the urgent need for antimicrobial stewardship and the importance of urine culture and susceptibility, especially in dogs with previous antimicrobial or immunosuppressive drug use, micturition disorders, or previous catheterization.

## Abstract ID10: Use of an Online Questionnaire to Monitor the Success of a Hospital Antimicrobial Stewardship Program

### **Madeleine R. Stein** ^1^; Ian DeStefano^2^, DVM, DACVECC; Kirthana Beaulac^3^, PharmD; Shira Doron^4^, MD; Claire Fellman^5^, DVM, PhD, DACVIM, DACVCP

#### ^1^Cummings School of Veterinary Medicine, North Grafton, MA, USA; ^2^Clinical Sciences, Cummings School of Veterinary Medicine, North Grafton, MA, USA; ^3^Emerson Hospital, Concord, MA, USA; ^4^Tufts Medical Center and Tufts University School of Medicine, Boston, MA, USA; ^5^Department of Clinical Sciences, Cummings School of Veterinary Medicine, Tufts University, North Grafton, MA, USA

**Background:** A One Health approach is being used to develop antimicrobial stewardship programs (ASPs) in companion animal medicine. Success of these ASPs should be monitored using a variety of methods.

**Hypothesis/Objectives:** Objectives were to assess changes in veterinarian and veterinary student attitudes and self‐reported practices one year after the introduction of an ASP at a major tertiary care center.

**Animals:** None.

**Methods:** This was a prospective, survey‐based study. Data were collected via an online questionnaire at baseline (August 2021) and one year after implementation of a hospital ASP (September 2022). A mix of descriptive and analytic statistics were used.

**Results:** A total of 78 complete responses were received prior to the ASP, and 51 responses at one year. No significant changes in attitudes or self‐reported prescribing habits were noted, although the majority of participants (>80%) reported that stewardship was important to them and expressed a desire to improve their prescription habits at both timepoints. Successful aspects of the ASP included pre‐made packs for use in acute diarrhea, with 88% of participants finding them very or somewhat useful. Additionally, hospital specific prescription guidelines and mandatory reporting of indication on antimicrobial prescriptions were viewed as useful by 82% and 76% of participants, respectively.

**Conclusions and Clinical Importance:** Interest in antimicrobial stewardship was high at this hospital, but self‐reported habits had not significantly changed one year after an ASP was introduced. Practical efforts to aid judicious prescription were viewed as most beneficial and should be emphasized in future stewardship efforts.

## Abstract ID11: Transcriptome Analysis of Peritoneal Effusions in Healthy Cats and Cats with Feline Infectious Peritonitis

### **Petra Cerna**; Lyndah Chow; Catriona MacPhail; Steven Dow; Michael Lappin

#### Colorado State University, Fort Collins, CO, USA

**Background:** The pathogenesis of feline infectious peritonitis (FIP) is still poorly understood, and transcriptome analysis can help to identify new insights into immunological mechanisms in cats with effusive FIP.

**Hypothesis/Objectives:** To analyze the transcriptome of peritoneal cells of healthy cats and cats with effusive FIP. We hypothesize that there would be a significant difference between healthy cats and cats with effusive FIP.

**Animals:** Two healthy research colony cats and two client‐owned cats diagnosed with effusive FIP.

**Methods:** Peritoneal lavage samples were obtained from healthy cats during routine ovariectomy and from abdominocentesis in cats with FIP. The RNA was extracted and subjected to Illumina sequencing. Sequence files were aligned with the reference cat genome and assembled using Partek flow software. Gene set enrichment analysis (GSEA) was used for comparison.

**Results:** Expression of 558 genes was significantly upregulated, and expression of 732 genes was significantly downregulated in cats with FIP. Using GSEA, multiple pathways associated with Th1 T‐cell responses, including the interferon gamma, interferon alpha, TNF‐α, and STAT3 signaling pathways were identified. There was also a significant downregulation of the epithelial to mesenchymal transitional pathway.

**Conclusions and Clinical Importance:** This study provides new insights into the normal peritoneal fluid transcriptome in cats as well as transcriptome analysis of peritoneal fluid of cats with effusive FIP which shows the importance of T‐cell activation and signaling in these patients. These findings suggest additional immune targets in cats with FIP, including activation of Th1 pathway responses.

## Abstract ID12: Multi‐institutional Retrospective Study Investigating Blood Culture Protocols and Test Positivity in 701 Dogs

### **Andrzej Ogrodny** ^1^; Harry Cridge^2^, MVB, MS, PG Cert Vet Ed, DACVIM (SAIM), DECVIM‐CA, FHEA, MRCVS; Rinosh Mani^3^; Sarah Schmid^4^; Emily Gould^5^; Claire Fellman^6^; Ian DeStefano^6^; Sarah Shropshire^7^; Jillian Haines^8^; Timothy Bolton^9^; Sara Jablonski^1^; Nicole Jess^10^

#### ^1^College of Veterinary Medicine, Michigan State University, East Lansing, MI, USA; ^2^Assistant Professor, College of Veterinary Medicine, Michigan State University, East Lansing, MI, USA; ^3^Microbiologist, Veterinary Diagnostic Laboratory, College of Veterinary Medicine, Michigan State University, East Lansing, MI, USA; ^4^College of Veterinary Medicine, University of Tennessee, Knoxville, TN, USA; ^5^School of Veterinary Medicine and Biomedical Sciences, Texas A&M University, College Station, TX, USA; ^6^Cummings School of Veterinary Medicine, Tufts University, North Grafton, MA, USA; ^7^College of Veterinary Medicine and Biomedical Sciences, Colorado State University, Fort Collins, CO, USA; ^8^College of Veterinary Medicine, Washington State University, Pullman, WA, USA; ^9^Virginia‐Maryland College of Veterinary Medicine, Blacksburg, VA, USA; ^10^Center for Statistical Training and Consulting, Michigan State University, East Lansing, MI, USA

**Background:** Blood cultures (BCs) are used to identify bacteremia. No standardized protocols exist for the timing or number of samples.

**Hypothesis/Objectives:** Evaluate if BC positivity is impacted by i) the number or timing of blood samples, ii) antibiotic or immunosuppressive therapy, and iii) primary disease process. Secondary objectives were to document bacterial isolate and susceptibility data, and correlate clinicopathologic variables to culture positivity.

**Animals:** 701 dogs.

**Methods:** Multi‐institutional retrospective study. Mixed‐effect logistic regression was used to determine whether primary disease process, the number of BCs or the timing of sample collection influenced BC positivity. Prediction plots were generated, and correlations between minimum database and culture results were performed using two‐sided t‐tests.

**Results:** Dogs that had 3 samples had the greatest likelihood of obtaining a positive BC result (adjusted predictive value: 0.44, 95% CI: 0.21–0.70). The timing between sample collection had no significant effect on test positivity. Dogs with cardiac or urogenital disease had the greatest likelihood of a positive culture (adjusted predictive values: 0.33, 95% CI: 0.15–0.56 and CI: 0.16–0.56). Dogs with a positive BC were more likely to have a positive urine culture (OR: 4.36, 95% CI: 2.12–8.97, *P* = 0.003) and had higher mean cholesterol concentrations (279.5 mg/dL vs. 247.0 mg/dL, *P* = 0.008), and lower mean magnesium concentrations (1.88 mg/dL vs. 2.40 mg/dL, *P* = 0.003) than those with negative cultures.

**Conclusions and Clinical Importance:** Three BCs might be the optimal protocol. Urine culture, cholesterol, and magnesium results were associated with BC positivity in our population.

## Abstract ID13: Investigation of Anti‐*Leptospira* spp. Antibodies and Leptospiruria in Cats

### **Fernanda Vieira Amorim da Costa** ^1^; Mirela Paim^1^, DVM, MSc; Bárbara Rivas^1^, DVM, MSc; Daniela Lopes^1^, DVM, MSc; Aline Staudt^1^, DVM; Grasiele Sebastiao^1^, DVM; Kauana Kaefer^1^, DVM; Fabiana Mayer^2^, DVM, MSc, PhD; Luciana Nunes^3^, DVM, MSc, PhD; Rogério Rodrigues^2^, DVM, MSc, PhD

#### ^1^UFRGS, Porto Alegre, Rio Grande do Sul, Brazil; ^2^IPVDF, Sans Souci, Eldorado do Sul, Brazil; ^3^Associate Professor, Department of Statistics, UFRGS, Porto Alegre, Rio Grande do Sul, Brazil

Leptospirosis is a bacterial zoonosis that affects humans and animals worldwide. The transmission of the disease occurs from direct or indirect contact with urine, water, or tissues infected by *Leptospira* spp. Clinical signs may vary depending on the serovar and the host involved, besides the geographic location. Currently, it is known that felines can become infected and act as reservoirs for the disease.

The objectives of this study were to investigate the presence of anti‐*Leptospira* spp. antibodies and leptospiruria in cats, using microscopic agglutination test (MAT) and real‐time polymerase chain reaction (PCR) techniques, respectively. Cats were prospectively selected in a teaching hospital in Brazil from October 2020 to January 2021.

A total of 76 cats were included in the study, 33 with clinical signs previously associated with leptospirosis infection in the species and 43 asymptomatic ones. The frequency of anti‐*Leptospira* spp. in serum samples was 9.2% (7/76). The serovars Butembo, Icterohaemorrhagiae and Tarassovi were observed with antibody titers of 1:200 in the asymptomatic group, while in the symptomatic group the Pomona serovar showed the same titer. A positive sample of *Leptospira* spp. DNA in urine was found, representing a frequency of 1.3% (1/76) among the investigated animals. This cat was included in the group of symptomatic cats, and the main complaint was vomiting. The risk factors, clinical data and laboratory tests of positive animals showed no significant association with the results of serological and molecular diagnosis. Infection with *Leptospira* spp. and leptospiruria occurs in cats in Brazil.

## Abstract ID14: *Mycoplasma* Species and Immunosuppressive Viruses in Cats Affected by Oral Cavity Lesions

### **Fernanda Vieira Amorim da Costa** ^1^; Raquel Redaelli^2^, DVM, MSc; André Streck^3^, DVM, MSc, PhD; Carolina Braga^1^, DVM, MSc; Aline Kersting^4^, DVM; Deisy Lerner^2^, DVM, MSc; David Driemeier^1^, DVM, MSc, PhD

#### ^1^UFRGS, Porto Alegre, Rio Grande do Sul, Brazil; ^2^Gatices Veterinary Clinic; ^3^Institute of Biotechnology, UCS, Caxias do Sul, Rio Grande do Sul, Brazil; ^4^Smile Vet Veterinary Odontology

Oral cavity lesions in cats are relatively common and can influence their health and welfare. The main oral diseases observed in cats are periodontal disease, tooth resorption and chronic gingivostomatitis, and the latter still have uncertain etiology.

The purpose of this study was to investigate primary and secondary main causes of cats’ oral lesions, evaluating the role of *Mycoplasma* species isolation and its association with retrovirus infection. Selected patients included cats referred for oral prophylaxis and periodontal treatment by veterinary dentists, in which clinical and laboratorial evaluation was performed, including blood work and POC snap test for detection of feline immunodeficiency virus (FIV) antibody and feline leukemia virus (FeLV) antigen. Oral tissue samples were biopsied for histopathology and *Mycoplasma* species PCR during dental procedure.

A total of 78 patients with oral lesions were included in this study, and the most prevalent findings were periodontal disease Stage 3 to 4 in 57.7% of the cats, tooth resorption in 35.9% and chronic gingivostomatitis in 23%. Of all patients, 23 (29.5%) were positive for immunosuppressive virus infection (FIV and/or FeLV). There were 36 (46.2%) positive results for *Mycoplasma* species PCR. The association between chronic gingivostomatitis diagnosis, FIV or FeLV infection and *Mycoplasma* species detection was statistically significant (*P* = 0,003). This study identified that cats with oral cavity lesions, mainly with chronic gingivostomatitis, are commonly infected by retroviruses and *Mycoplasma* species. More studies should be performed to evaluate antimicrobial treatment against *Mycoplasma* species to control this disease in cats infected with FIV and FeLV.

## Abstract ID15: Field Evaluation of VETSCAN IMAGYST Artificial Intelligence (AI) Fecal Canine and Feline Parasite Detection

### **Yoko Nagamori** ^1^; Anonda Haskin^2^; Cory Penn^3^, DVM

#### ^1^Zoetis; ^2^Platform Lead, Global Diagnostics Medical Affairs, Zoetis; ^3^Senior Veterinary Diagnostic Platform Lead, Global Diagnostics Medical Affairs, Zoetis

**Background:** The VETSCAN IMAGYST AI Fecal application utilizes a deep learning artificial intelligence (AI) algorithm to detect parasite ova, cysts and oocysts in canine and feline feces. Deep learning AI algorithms evolve by learning from additional data sets provided by medical experts. Regular evaluation of algorithm performance and usability are essential to ensure the highest quality on‐market device.

**Hypothesis/Objectives:** The VETSCAN IMAGYST AI Fecal algorithm (v.8293) will correctly identify the diagnostic stages of *Ancylostoma*, *Cystoisospora*, *Giardia*, *Toxocara* and *Trichuris* in fecal samples of naturally infected dogs and cats previously scanned and manually evaluated by diagnostic parasitology experts from multiple geographies across the U.S.

**Animals:** 853 fecal samples from a mix of client‐owned animals undergoing routine fecal examinations (502 canine, 203 feline, and 148 *Giardia* (dog and cat)).

**Methods:** In previous studies, the 853 fecal samples were processed, manually read by diagnostic parasitology experts and digitally stored for future evaluation. In this study, those digitally scanned images were analyzed using the VETSCAN IMAGYST AI Fecal algorithm (v.8293) and compared against the original diagnostic parasitology expert manual reads.

**Results:** Combined diagnostic sensitivity and specificity for canine and feline samples for *Ancylostoma*, *Toxocara*, *Cystoisospora*, and *Giardia* ranged from 90.7–95.5% and 96.0–98.8%, respectively. Sensitivity and specificity for canine *Trichuris* was 93.1% and 99.7%, respectively.

**Conclusions and Clinical Importance:** The results of the VETSCAN IMAGYST AI Fecal algorithm tested in this study were comparable to those of diagnostic parasitology experts.

## Abstract ID17: Performance of C‐Reactive Protein and Haptoglobin Concentrations to Detect Remission in Dogs with Pulmonary Coccidioidomycosis

### **Aaren Glick** ^1^; Jared Jaffey^1^, DVM, MS, DACVIM (SAIM); Rachael Kreisler^2^, VMD, MSCE, DACVPM; Andrew Hanzlicek^3^, DVM, MS, DACVIM (SAIM); Randy Ringold^4^

#### ^1^College of Veterinary Medicine, Midwestern University, Glendale, AZ, USA; ^2^Associate Professor, College of Veterinary Medicine, Midwestern University, Glendale, AZ, USA; ^3^Director, Veterinary Medicine and Research, MiraVista Veterinary Diagnostics; ^4^Director, VDI Laboratory

**Background:** Treatment monitoring is subjective in dogs with pulmonary coccidioidomycosis. Biomarkers predictive of remission have not been explored.

**Hypothesis/Objectives:** To (i) to characterize changes in serum C‐reactive protein (CRP) and haptoglobin concentrations after starting antifungal therapy in dogs with pulmonary coccidioidomycosis, and (ii) evaluate the diagnostic performance of serum CRP and haptoglobin concentrations to detect remission.

**Animals:** Thirty‐one client‐owned dogs with newly diagnosed pulmonary coccidioidomycosis.

**Methods:** Prospective cohort study. Serum was obtained at baseline and once every 3 months after antifungal administration until either remission or 12 months. Remission required the following criteria: (i) anti‐*Coccidioides* spp. IgG titers ≤1:8, (ii) unremarkable or mild static thoracic radiographs, and (iii) an absence of related clinical signs. Serum CRP and haptoglobin were measured at a reference laboratory using ELISA assays.

**Results:** Twenty‐seven (87%) dogs achieved remission in the 12‐month study period. Median serum CRP and haptoglobin concentrations decreased from baseline (CRP: 56 mg/L; haptoglobin: 716 mg/dL) to the 3‐month (CRP: 17 mg/L; haptoglobin: 410 mg/dL) evaluation (both *P* < 0.0001); subsequent decreases were not significant. Eighteen (58%) and 16 (56%) dogs had normal serum CRP and haptoglobin concentrations, respectively, at the 3‐month visit. Serum CRP (sensitivity, 46%; specificity, 53%) and haptoglobin concentrations (sensitivity, 50%; specificity, 40%) at their optimal cutoffs were poor detectors of remission.

**Conclusions and Clinical Importance:** Serum CRP and haptoglobin concentrations decrease in the first 3 months of antifungal treatment in dogs with pulmonary coccidioidomycosis but are not useful adjunctive tests to facilitate detection of remission.

## Abstract ID18: Associations Between Physical and Mental Health and Exposure to *Bartonella henselae* in Small Animal Veterinarians

### **Haley C. Uustal** ^1^; Edward Breitschwerdt^2^, DVM; Chance Liedig^2^, MS; Erin Lashnits^3^, MS, DVM, PhD, DACVIM

#### ^1^School of Medicine and Public Health, University of Wisconsin–Madison, Madison, WI, USA; ^2^Intracellular Pathogens Research Laboratory, North Carolina State University, Raleigh, NC, USA; ^3^Assistant Clinical Professor, Department of Medical Sciences, School of Veterinary Medicine, University of Wisconsin–Madison, Madison, WI, USA

**Background:** Small animal veterinarians have a high risk of occupational exposure to *B. henselae*. This study will examine the association between *B. henselae* exposure and physical and mental health in this population.

**Hypothesis/Objectives:** Small animal veterinarians exposed to *B. henselae* will report higher prevalence and severity of neuromuscular symptoms, depression, and anxiety than those without exposure, and will report greater severity of fatigue and cognitive failure.

**Animals:** 385 practicing small animal veterinarians were recruited when seeking rabies titer testing at a large United States veterinary conference. Potential participants were blinded to the exposure of interest to not bias recruitment.

**Methods:** Case‐control study. Outcomes were self‐reported via an electronic questionnaire, including measures of depression and anxiety (evaluated with the Patient Health Questionnaire‐9 and the General Anxiety Disorder‐7, respectively), fatigue and cognitive failure (evaluated with the Checklist of Individual Strength and Cognitive Failures Breakdown), and clinical diagnoses. Exposure to *B. henselae* was detected using IFA serology and qPCR on whole blood.

**Results:** Blood, serum, and questionnaires were collected in January 2023 from 350 participants. Participants were 87.5% female and 12.5% male, with a mean age of 42 (27–69 years). The association between *B. henselae* exposure and the prevalence and severity of symptoms will be presented.

**Conclusions and Clinical Importance:** While *B. henselae* exposure is common in small animal veterinarians, there is little known about its impact on the health of this population. A greater understanding of *B. henselae* infections can increase detection and treatment of symptoms.

## Abstract IM01: Effect of Feeding *Enterococcus Faecium* Strain SF68 on Leukocyte Transcriptomic Responses in Healthy Cats

### **Michael R. Lappin**; Lyndah Chow; Alison Manchester; Steve Dow; Jennifer Hawley

#### Colorado State University, Ft Collins, CO, USA

**Background:** *Enterococcus faecium* strain SF68 (SF68) is the bacterium in probiotic and synbiotic products marketed for dogs and cats in many countries. There is published evidence in puppies (improved vaccine responses) and cats (lessened FHV‐1 reactivation during stress) that the probiotic may also support immune health.

**Hypothesis/Objectives:** The purpose of this study was to collect additional information concerning the immune modulatory effects of SF68 when fed as a synbiotic (combined with psyllium as the prebiotic) to healthy research cats.

**Methods:** Two groups of four young adult research cats were chosen for this IACUC approved protocol. All cats were acclimated to the facility, housed identically, and fed the same diet. The cats were randomized into the synbiotic group or the placebo group (canned food alone). On Day 28, polyA‐enriched RNA was extracted from blood leukocytes. RNA sequencing was performed using an Illumina‐based platform. Sequence files were aligned with the reference cat genome and assembled using Partek flow software. Gene set expression analysis (GSEA) was performed using open‐source software, with significance was set at FDR of <0.05 and *P* < 0.1.

**Results:** Group comparisons of leukocyte transcriptomic data from Day 28 revealed that cats fed SF68 had 45 significantly upregulated genes and 33 significantly downregulated genes. TNF‐a and TGF‐b signaling pathways were significantly upregulated while interferon‐alpha and interferon‐gamma pathways were significantly downregulated.

**Conclusions/Importance**

These findings indicate that feeding SF68 can elicit significant immune responses in circulating leukocytes. Correlative studies of GI responses to this dietary intervention may provide additional insights.

## Abstract IM02: Pilot Evaluation of Inflammatory Markers in Aging Dogs

### **Sarah M. Schmid** ^1^; Jessica Hoffman^2^; Jena Prescott^3^; Holley Ernst^3^; Daniel Promislow^4^; Kate Creevy^3^; DAP Consortium^3^

#### ^1^University of Tennessee, Knoxville, TN, USA; ^2^Augusta University, Augusta, GA, USA; ^3^Texas A&M University, College Station, TX, USA; ^4^University of Washington, Seattle, WA, USA

**Background:** In people, plasma inflammatory markers tumor necrosis factor‐α (TNF‐α), interluken‐6 (IL‐6), and C‐reactive protein (CRP) increase with age and are predictors of mortality.

**Objectives:** To characterize the effects of age, sex, weight, and body condition score (BCS) on serum S100A12, N‐methylhistamine (NMH), CRP, interleukins (ILs)‐2, 6, 8, and TNF‐α concentrations in healthy companion dogs.

**Animals:** 49 healthy dogs enrolled in the Dog Aging Project (DAP) Pack with banked serum samples.

**Methods:** Dogs were determined to be healthy on the basis of a lack of owner‐reported diseases, review of electronic medical records, and normal blood work. Serum ILs and TNF‐α concentrations were measured using a multiple electrochemiluminescence immunoassay validated in dogs. Serum S100A12 and NMH concentrations were determined using sandwich ELISA tests and gas chromatography‐mass spectrometry, respectively. BCS was assigned by a single observer reviewing owner‐provided photographs. Association of inflammatory markers with age, sex, weight, and BCS was evaluated by linear regression.

**Results:** There was no effect of BCS on any inflammatory measure. Positive associations were identified between age and several cytokines: IL‐2 (*P* = 0.0191), IL‐6 (*P* = 0.0005), IL‐8 (*P* = 0.0059), and TNF‐α (*P* = 0.00067), and between female sex and NMH (*P* = 0.0168). A negative association was identified between IL‐8 and weight, with smaller dogs having higher concentrations than large dogs (*P* = 0.0078).

**Conclusions and Clinical Importance:** Association of cytokines (IL‐2, IL‐6, IL‐8, and TNF‐α) with age and IL‐8 with dog size warrant further evaluation in a larger population.

## Abstract IM03: Role of CD8+ T Cells and Immune Activation in Pathogenesis of Inflammatory Bowel Disorder in Canines

### **Regina L. Hollar**; Selena Tavener; Kiran Panickar

#### Hill's Pet Nutrition

**Background:** Pathogenesis of inflammatory bowel disease (IBD) is multifactorial. Intestinal inflammation due to increased pro‐inflammatory cytokines and chemokines leads to mucosal barrier dysfunction and subsequent aberrant immune response.

**Hypothesis:** Tissue‐resident immune cells including excessive activation of T lymphocytes contribute to the pathogenesis of IBD.

**Animals:** Dogs, from Hill's colony, were diagnosed with IBD clinically by a veterinarian via endoscopy and re‐confirmed via pathology at necropsy at end of their natural life (*n =* 12; 5.3–15.3 yr). Control dogs exhibited no signs of IBD during life (*n =* 12, 6.0–15.0 yr)

**Methods:** We assessed gene expression, in a retrospective study, in transverse colon tissue collected at necropsy. RNA was extracted from tissues and gene expression investigated using Canine Immuno‐Oncology panel of the NanoString nCounter platform. Analysis performed using nSolver software.

**Results:** Significant increase in 24 genes in IBD compared to controls. Markers of CD8+ T cells were upregulated in IBD with CD8B increased 1.55‐fold (*P* < 0.05). CD8 antigen is a cell surface glycoprotein found on most cytotoxic T cells. KLRG1 (2.01‐fold, *P* < 0.05), receptor for E‐cadherin and expressed on CD8+ T cells; Granzyme B (1.86, *P* < 0.05), a protease secreted by CD8+ T cells and natural killer cells, and FLT3 (1.47, *P* < 0.05), a receptor expressed on CD103+ dendritic cells which can mediate CD8+ T‐cell proliferation and activation, were increased in IBD.

**Conclusions:** Upregulation of CD8+ T cells and KLRG1 is consistent with ulcerative colitis in humans. Results indicate an important role of CD8+ lymphocytes in IBD and diverse immune targets for reducing inflammation in canines with IBD.

## Abstract IM04: Comparison of Prednisolone Alone and Prednisolone Combined with Adjunctive Immunosuppressants for Dogs with Immune‐Mediated Polyarthritis

### **Jungil Kim**; Jeong‐Yeol Bae; Guk‐il Joung, DVM; Jin‐Young Kim, DVM; Joong‐Hyun Song, DVM, PhD, Professor

#### Veterinary Teaching Hospital, Chungnam National University, Daejeon, Korea

**Background:** Prednisolone (PDS) is commonly used for primary immune‐mediated polyarthritis (IMPA). The comparison of PDS monotherapy and PDS combined with adjunctive immunosuppressant for dogs with IMPA is scarce.

**Hypothesis/Objectives:** PDS combined with adjunctive immunosuppression is not superior to PDS alone in terms of efficacy and safety in dogs with IMPA.

**Animals:** Thirteen client‐owned dogs with primary IMPA. 6 dogs (group A) were treated with PDS alone, and 7 dogs (group B) were treated with PDS combined with the additional immunosuppressant.

**Methods:** Retrospective study. The data collected from the medical records included information regarding signalment, treatment response, and adverse effects.

**Results:** Complete response (CR) was achieved in both groups. The median time to CR was 19 days (range, 3 to 40) in group A and 17 days (range, 6 to 28) in group B. The median time of initial PDS tapering was 27.5 days (range, 18 to 41) in group A and 35 days (range, 11 to 84) in group B. The relapse was observed in 1 dog in group A and 3 dogs in group B. Adverse effects (AEs) were observed in 2 dogs (PDS‐related AEs) in group A and 3 dogs (sporadic infection, *n =* 2; PDS‐related AEs, *n =* 3) in group B.

**conclusions and clinical relevance:** The efficacy of PDS monotherapy is comparable to PDS combined with adjunctive immunosuppression in dogs with IMPA. Sporadic infections more frequently occurred in the dogs treated with the additional immunosuppressant. The overall findings help us extend our understanding of the treatment for IMPA.

## Abstract IM05: Prevention of Mortality by Canine Parvovirus Monoclonal Antibody Treatment After Experimental Challenge

### **Laurie J. Larson** ^1^; Lindy Miller^2^; Mary Margiasso^3^; Michael Piontkowski^4^; Barton Slagter^3^; Danielle Tremblay^3^; Stephanie Dykstra^3^; Jennifer Miller^3^; Debbie Champ^5^; Terri Wasmoen^6^

#### ^1^CAVIDS Titer Testing laboratory, School of Veterinary Medicine, University of Wisconsin–Madison, Madison, WI, USA; ^2^LFM Quality Research; ^3^Elanco Animal Health; ^4^Bighorn Veterinary Consulting; ^5^DAC Consulting; ^6^Innovative BioTech Consulting

**Background:** Canine parvovirus (CPV) remains a leading cause of death in young dogs.

**Objective:** Determine whether intravenous administration of canine parvovirus monoclonal antibody (CPMA) can prevent mortality after experimental CPV‐infection.

**Animals:** CPV seronegative 8‐week‐old beagles were randomized into groups: (1) Controls (saline; *n =* 7) and (2) CPMA (*n =* 21).

**Methods:** Susceptible dogs were intranasally challenged with CPV‐2b (Day 0). One dose of CPMA or saline was administered intravenously after dogs tested SNAP‐positive feces (IDEXX). Dogs were monitored for 14 days for clinical signs, seroconversion and viral shed. Staff were masked to group identity. Moribund dogs were euthanized (per IACUC) and no other supportive care was administered during the study.

**Results:** All dogs met CPV disease criteria and were treated on Day 4. By Day 5, CPMA‐treated group rapidly reached high anti‐CPV titers (μ HI = 3078) while controls remained seronegative. Controls developed severe disease: fever >103.4°F (71%), bloody diarrhea (100%), lymphopenia (100%), viral shed (100%), and mortality (57%). Resolution of clinical signs was significantly faster for vomiting (*P* = 0.0478), lethargy (*P* = 0.0185), and inappetence (*P* = 0.0478) in CPMA‐treated dogs compared to controls. The CPMA group had fewer severe signs overall including reduced diarrhea (Figure 1), fever (*P* < 0.0001), and lymphopenia (*P* = 0.0619). No CPMA‐treated dogs died due to challenge. The prevented fraction for mortality in CPMA‐treated dogs compared to controls was estimated at 1.00 (95% confidence interval 0.73, 1.00).

**Conclusions and Clinical Importance:** Intravenous treatment with CPMA statistically significantly decreased the duration of clinical disease and prevented mortality associated with canine parvovirus infection.

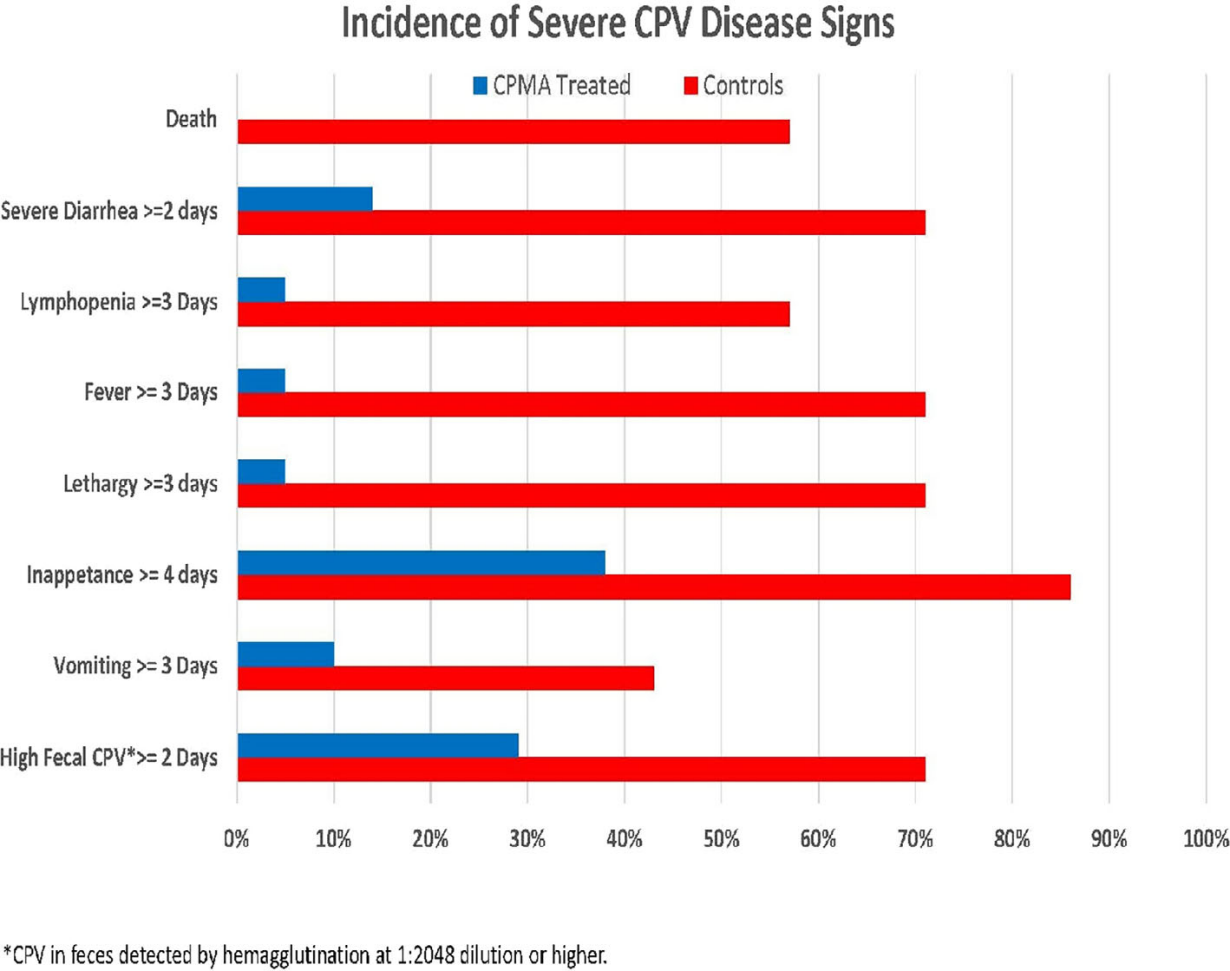

## Abstract IM06: Upregulation of SOCS1 and CXCL14 Can Contribute to the Pathogenesis of Hyperthyroidism in Cats

### **Selena K. Tavener** ^1^; Regina Hollar^2^, BS; Kiran Panickar^3^, PhD

#### ^1^Science & Technology Center, Hill's Pet Nutrition; ^2^Associate Scientist, Science & Technology Center, Hill's Pet Nutrition; ^3^Manager, Science & Technology Center, Hill's Pet Nutrition

**Background:** Feline hyperthyroidism is characterized by increased production of tetra‐iodothyronine (T4) and triiodothyronine (T3) and decreased levels of thyroid‐stimulating hormone.

**Hypothesis:** Inflammation, including increased cytokine levels, contributes to the pathogenesis of feline hyperthyroidism.

**Animals:** Cats clinically diagnosed as having hyperthyroidism by a veterinarian (*n =* 10, 12.1–18.9 yr). Circulating fT4 was significantly higher in hyperthyroid cats (*P* < 0.05). Control cats showed no signs of hyperthyroidism clinically (*n =* 10, 10.9–18.6 yr).

**Methods:** RNA was isolated from thyroid tissue collected from cats housed in the Hill's colony at the natural end‐of‐life after living full lives. Gene expression was investigated using NanoString nCounter and analysis performed using nSolver software. Histopathological analysis was performed on hematoxylin and eosin‐stained slides.

**Results:** Suppressor of cytokine signaling 1 (SOCS1) was significantly upregulated when compared to controls. There was a significant decrease in Janus Kinase 1 (JAK1) and a decrease in Signal Transducer and Activator of Transcription 1 (STAT1) (ns), IL7R (*P* < 0.05) and Cytotoxic T‐Lymphocyte Associated Protein 4 (CTLA4) (ns). An increase in SOCS1 and a decrease in the above signaling molecules may affect the production and maintenance of T regulatory cells, potentially leading to autoimmune disorders. There was a significant increase in CXCL14, a pro‐inflammatory chemokine and chemoattractant for monocytes and dendritic cells, in hyperthyroid cats. Histopathology from thyroid tissue showed increased follicular cells and evidence of colloid reabsorption in cats with hyperthyroidism compared to controls.

**Conclusions and Clinical Importance:** These biomarkers represent important targets for reducing inflammation‐associated signaling pathways in hyperthyroidism.

## Abstract NU01: Feline Urine Ammonia‐To‐Creatinine Ratio Reference Interval

### **Eleanor E. Brown** ^1^; Rebeca Castro^2^, BS; Francesca Griffin^2^, DVM, MS; Wendy Mandese^2^, DVM, DAVBP; Jenna Rooks^2^, DVM; Amy Stone^2^, DVM, PhD; Julia Wuerz^2^, DVM; Kirsten Cooke^2^, DVM, DACVIM (SAIM); Andrew Specht^2^, DVM, DACVIM (SAIM); Autumn Harris^2^, DVM, DACVIM (SAIM)

#### ^1^College of Veterinary Medicine, University of Florida, Gainesville, FL, USA; ^2^University of Florida, Gainesville, FL, USA

**Background:** Renal ammonia metabolism is critical to maintaining acid‐base homeostasis. Inadequate ammonia excretion drives development of metabolic acidosis in people with chronic kidney disease (CKD) and correlates with worse clinical outcomes. Metabolic acidosis is commonly recognized in cats with CKD, but there is limited information about ammonia excretion in these patients.

**Objectives:** The purpose of this study was to generate a reference interval (RI) for urine ammonia‐to‐creatinine ratio (UACR) in a population of healthy adult cats.

**Animals:** 92 adult, client‐owned cats were assessed via history, physical examination, serum chemistry, and urinalysis, and 20 were excluded. Urine samples (*n =* 72) were processed and stored under mineral oil at −80°C.

**Methods:** Ammonia and creatinine concentrations were measured using commercially available enzymatic assays. To establish RI for UACR, 2.5% and 97.5% limits were determined using nonparametric methods. The Dixon method was used to detect and exclude outliers. The Anderson‐Darling test was used to assess for normality, and statistical significance was set at *P* < 0.05 for all analyses.

**Results:** The RI for UACR was determined to be 3.4–20.7 with 90% confidence intervals of 3.0–3.7 and 16.0–23.7 for the lower and upper limits of detection respectively. General linear models did not detect significant relationships between UACR and serum bicarbonate, sex, weight, or age.

**Conclusions and Clinical Importance:** Having a RI for UACR for healthy cats will provide a foundation for future research to determine whether alterations in ammonia excretion are associated with specific disease states, which may provide prognostic information or influence clinical management strategies.

## Abstract NU02: Correlation of Ammonia Excretion with Renal Function and Serum Bicarbonate in Cats with Kidney Disease

### **Eleanor E. Brown** ^1^; Rebeca Castro^2^, BS; Kirsten Cooke^2^, DVM, DACVIM (SAIM); Andrew Specht^2^, DVM, DACVIM (SAIM); Autumn Harris^2^, DVM, DACVIM (SAIM)

#### ^1^College of Veterinary Medicine University of Florida, Gainesville, FL, USA; ^2^Department of Small Animal Clinical Science, College of Veterinary Medicine, University of Florida, Gainesville, FL, USA

**Background:** Metabolic acidosis is common in cats with chronic kidney disease (CKD). Inadequate ammonia excretion is thought to drive development of metabolic acidosis in people with CKD and correlates with worse outcomes. There is limited information about ammonia excretion in cats with CKD.

**Hypothesis/Objectives:** The primary aim was to determine if urine ammonia‐to‐creatinine ratio (UACR) correlated with renal function (serum creatinine) or serum bicarbonate in cats with CKD. A secondary aim was to evaluate if ammonia excretion was different in healthy (abstract: 1431493) and CKD cats.

**Animals:** Twenty‐one client‐owned cats with stable CKD IRIS stage 2–4 were assessed via history, physical examination, serum chemistry, PCV/TP, urinalysis, urine protein‐to‐creatinine ratio, and blood pressure measurement. Urine samples were processed and stored under mineral oil at −80°C.

**Methods:** Urinary ammonia and creatinine concentrations were measured using commercially available enzymatic assays and used to calculate UACR. Coefficient of determination (Pearson correlation coefficient squared) between UACR and serum creatinine, UACR and serum bicarbonate was calculated. Mean UACR of healthy versus CKD cats was compared using unpaired Student's T‐test.

**Results:** UACR weakly correlates with serum creatinine (*R* ^2^=0.22) and serum bicarbonate (*R* ^2^=0.10). There was a significant difference (*P* < 0.001) between UACR in healthy (8.9 ± 4.4) and CKD (2.8 ± 1.5) cats.

**Conclusions and Clinical Importance:** Clinical significance of the difference in ammonia excretion between healthy and CKD cats is unknown. Future research is warranted to determine if ammonia excretion alteration in CKD patients provides prognostic information or influences clinical management.

## Abstract NU03: Complications Associated With Percutaneous Ultrasound‐guided Renal Biopsy in 51 Dogs

### **Colleen Bourque** ^1^; Emmanuelle Butty^2^, med.vet., DACVIM (SAIM); Mary Labato^1^, DVM, DACVIM; Florian Wuillemin^1^, DED, DACVIM

#### ^1^Cummings School of Veterinary Medicine at Tufts University, North Grafton, MA, USA; ^2^Clinical Assistant Professor, Department of Clinical Sciences, Cummings School of Veterinary Medicine at Tufts University, North Grafton, MA, USA

**Background:** Renal biopsies are recommended to further investigate primary glomerular disease and/or acute kidney injury. Biopsy‐related complications are often a concern.

**Objectives:** To describe the incidence and severity of complications after percutaneous ultrasound‐guided renal biopsy (PURB) in dogs and to identify risk factors associated with these complications.

**Animals:** Fifty‐one client‐owned dogs that underwent PURB.

**Methods:** Complications of PURB were retrospectively reviewed, and their association with biopsy technique (needle gauge, number of biopsies, sedation, or anesthesia) and patient variables (weight, blood pressure, platelet count, PT/PTT, albumin level, and anticoagulant use) were compared. Creatinine, BUN, and PCV were assessed pre‐ and post‐biopsy, and absolute change of PCV (ΔPCV) was calculated as PCVpre‐PCVpost.

**Results:** Renal biopsies were collected using a 16‐ or 18‐gauge needle. Immediate bleeding following biopsy was visualized on ultrasound in 16/51 (31%) dogs. Forty‐one (80%) dogs had a decrease in PCV with a median ΔPCV of −7% (range, −2% to −17%). Only 1 dog required clinical intervention and received a blood transfusion. There was no association with risk of major bleeding (PCV decrease >6%) and biopsy technique or patient variables. There was no significant change in BUN (*P* = 0.241) or creatinine (*P* = 0.953) following biopsy.

**Conclusion and Clinical Importance:** Bleeding was the most common complication but was self‐limiting and did not require intervention in the majority of dogs that underwent PURB. Renal biopsies are an important diagnostic tool that can be safely collected percutaneously under ultrasound‐guidance using a 16 or 18‐gauge needle.

## Abstract NU04: Comprehensive Assessment of Hemostasis in Dogs With Acute Kidney Injury and Chronic Kidney Disease

### **Hilla Chen**; Itamar Aroch; Gilad Segev

#### Koret School of Veterinary Medicine, The Hebrew University of Jerusalem, Rehovot, Israel

**Background:** Bleeding tendencies and platelet dysfunction occurs with uremia. Impaired primary hemostatic function in dogs with acute kidney injury (AKI) and chronic kidney disease (CKD) has clinical implications, however, its comprehensive evaluation is limited.

**Objectives:** To assess hemostasis, including determination of platelet function and thromboelastometry of dogs with AKI and CKD.

**Animals:** Client‐owned dogs (*n =* 36; AKI, 18; CKD, 12; healthy controls, 6) presented to a teaching hospital.

**Methods:** Prospective study. PFA‐200 closure times using collagen‐ADP and collagen‐epinephrine agonists were determined. Hemostasis was evaluated by PT, aPTT, antithrombin activity, D‐dimer and fibrinogen concentrations, and thromboelastometry.

**Results:** Collagen‐ADP closure time of the AKI group [median, 139 seconds (range, 71–301)] was longer compared with the CKD [median, 77 seconds (range, 56–235), *P* = 0.042] and control [median, 70 seconds (range, 52–79), *P* = 0.003]. Additionally, the proportion of dogs with prolonged closure time was higher in the AKI compared with controls for both collagen‐ADP and collagen‐epinephrine (78% vs. 0%, *P* < 0.001 and 44% vs. 0%, *P* = 0.05, respectively). Collagen‐ADP closure time was correlated with creatinine (*r =* 0.61, *P* < 0.002) but not with platelet count or clotting times. The thromboelastometry clot formation time was lower while its α‐angel and maximal clot firmness were higher in the AKI and CKD groups compared with controls. Antithrombin activity was lower (*P* = 0.001), while fibrinogen was higher (*P* = 0.005) in dogs with AKI compared with controls.

**Conclusions and Clinical Importance:** Dogs with AKI show hemostatic abnormalities, including hypercoagulability in face of platelet dysfunction. Therefore, comprehensive assessment of hemostasis, including platelet function, is recommended prior to invasive interventions.

## Abstract NU05: The Impact of Nutrition on the Urobiome of 15 Clinically Healthy Adult Dogs

### **Emily Coffey** ^1^; Andres Gomez^1^, PhD; Aaron Ericsson^2^, DVM, PhD; Erin Burton^1^, DVM, DACVP; Jennifer Granick^1^, DVM, PhD, DACVIM (SAIM); Jody Lulich^1^, DVM, PhD, DACVIM (SAIM); Eva Furrow^1^, VMD, PhD, DACVIM (SAIM)

#### ^1^University of Minnesota, Minneapolis, MN, USA; ^2^University of Missouri, Columbia, MO, USA

**Background:** Nutrition plays an important role in shaping gut microbiome composition and might also impact the urobiome.

Hypothesis/**Objective:** The aim of this project was to determine if nutritional features affect diversity and composition of the urobiome in dogs.

**Animals:** Fifteen clinically healthy spayed/neutered adult dogs (client‐owned).

**Methods:** This was a cross‐sectional study. Dietary histories were obtained, and macronutrient intake (g/100 kcal protein, fat, and crude fiber), commercial diet brand, and dietary diversity (number of unique food sources) were recorded. Microbial DNA was extracted from urine collected via cystocentesis, and V4 amplicon sequencing of the bacterial 16S rRNA gene was performed. Data were analyzed to determine how variation in macronutrients and dietary sources contributed to urobiome alpha diversity, beta diversity, and individual taxa abundance.

**Results:** Macronutrient content was not found to affect alpha or beta diversity. Bray Curtis measure of beta diversity differed (*R* ^2^=0.10, *P* = 0.017) between dogs fed one commercial diet brand compared to dogs consuming any other brand, characterized by lower alpha diversity (*P* = 0.018) and lower relative abundances *Staphylococcus*, *Bacillus halodurans*, and *Paracoccus*. Beta diversity also differed (*R* ^2^=0.10, *P* = 0.019) between dogs consuming more diverse daily diets (≥3 unique food sources) as compared to those consuming less diverse diets.

**Conclusions and Clinical Importance:** Due to overlap between diet brand and dietary diversity groups, it is unclear whether one or both variables drove urobiome differences. Overall, the results of this study suggest that diet might impact the urobiome in dogs.

## Abstract NU06: Use of a Bipolar Resectoscope for Debulking of Obstructive Urethral Masses in Dogs

### **Karina J. Creighton**; JD Foster, VMD, MS, DACVIM (SAIM)

#### Friendship Hospital for Animals, Fort Collins, CO, USA

**Background:** Transurethral debulking (TUD) of obstructive urethral masses using a bipolar resectoscope is a novel alternative treatment to laser debulking, which is impractical for extensive tumors, and urethral stenting, which has a relatively frequent incidence of incontinence and persistent dysuria post‐procedure.

**Objective:** To evaluate procedural complications and post‐procedural urinary signs for patients undergoing TUD.

**Animals:** Five dogs presenting with lower urinary signs secondary to known or suspected urothelial carcinoma.

**Methods:** This is a retrospective study. Dogs who underwent TUD using a 5 mm bipolar resectoscope were included. Procedural complications, post‐procedural urinary signs, and time to reobstruction or death were recorded from the medical record.

**Results:** All dogs were female. Urothelial carcinoma was confirmed with cytology (1/5), BRAF test (2/5), and/or biopsy (3/5). Four dogs had 1 TUD, 1 dog had a second TUD six months after the first TUD. Iatrogenic full‐thickness urethral tear occurred in one TUD, no other procedural complications were noted. Improvement in lower urinary signs was noted after 5/6 TUD. One dog had persistent dysuria requiring urethral stent placement 18 days after TUD. Median time from TUD to reobstruction or death was 122 days (Range 16–285 at the time of submission). No incontinence was reported post TUD.

**Conclusions and Clinical Importance:** TUD using a bipolar resectoscope had a low procedural complication rate in this canine cohort and may present an effective alternative to urethral stent placement in the management of obstructive urothelial carcinoma.

## Abstract NU07: The Effect of Circumferential Tape Applied to the Cuff During Blood Pressure Measurement in Cats

### **Cailin Harro**; JD Foster, VMD, MS, DACVIM

#### Friendship Hospital for Animals, Fort Collins, CO, USA

**Background:** Doppler ultrasonic sphygmomanometry (Doppler) blood pressure measurement is commonly used to measure systolic blood pressure (SBP) in cats. It has been suggested that applying tape to hold the blood pressure cuff closed may affect Doppler results, however, this claim has not been directly investigated.

**Objectives:** To determine if applying circumferential white tape to a blood pressure cuff affects the Doppler SBP measurements in cats.

**Animals:** Client owned cats having SBP measured as part of their internal medicine evaluation.

**Methods:** Prospective crossover study with randomized order of SBP measurement methodology. Five serial SBP measurements were obtained with the first method (tape or untaped) and averaged according to ACVIM consensus guidelines. This was repeated with the alternate method. Average SBP was compared with a paired Student's t‐test.

**Results:** 35 cats were enrolled. There was no significant difference when SBP was measured untaped (mean 132.7 ± 29.33 mm Hg) or with tape (mean 135.9 ± 29.17 mm Hg) [*P* = 0.0873]. There was no difference in the standard deviation for each method (4.55 mm Hg vs. 5.09 mm Hg) [*P* = 0.41]. There was no significant difference between the first (mean 133.9 ± 29.29 mm Hg) or second method of SBP measurement (mean 134.7 ± 29.3 mm Hg) [*P* = 0.691]. The median difference between SBP measured with tape vs. untaped was +3.2 mm Hg (range −21.6 to 31.6 mm Hg).

**Conclusions and Clinical Importance:** Applying white tape to the cuff does not significantly alter Doppler SBP measurement in cats. Some cats had different results between the two methods; changing to the opposite method may be indicated when unexpected results occur.

## Abstract NU08: Evaluation of Urine Dipstick Protein:Urine Specific Gravity Ratio as a Test of Proteinuria in Dogs

### **David Haas**; JD Foster, VMD, MS, DACVIM (SAIM)

#### Friendship Hospital for Animals, Fort Collins, CO, USA

**Background:** The urine protein:creatinine ratio (UPC) is the gold standard for quantifying proteinuria. This is not always performed, limiting retrospective analysis of proteinuria. The urine dipstick protein:urine specific gravity (DP:USG) ratio has recently been used as marker for proteinuria, although this test has not been validated against UPC.

**Objective:** To investigate the correlation between DP:USG and UPC in dogs.

**Animals:** Dogs who had urinalysis and UPC performed for any reason.

**Methods:** Retrospective case study. The DP:USG was calculated and compared to UPC as continuous and categorical variables using a cutoff >1.5 as abnormal.

**Results:** 377 dogs were identified through retrospective medical record search. 216 dogs (58.7%) were excluded because the DP:USG could not be determined due to negative (*n =* 168, 43.1%) or trace (*n =* 61, 15.6%) dipstick urine protein results. 33 (15.3%) of the dogs with negative or trace dipstick proteinura that could not undergo DP:USG evaluation had UPC >0.5. DP:USG was significantly associated with UPC (*P* < 0.0001, *r =* 0.725). The DP:USG cutoff of >1.5 was 93.6% sensitive, 68.6% specific, and had 88% accuracy at identifying dogs with a UPC >0.5.

**Conclusions and Clinical Importance:** DP:USG is fairly reliable to predict UPC >0.5 only when ≥1+ protein is detectable via dipstick. DP:USG is not an appropriate substitute for UPC when quantifying the degree of proteinuria, nor should it be used to reliably quantify the magnitude of proteinuria. Absent and trace dipstick proteinuria precludes DP:USG measurement and fails to identify some proteinuric dogs.

## Abstract NU09: Proteinuria in Dogs and Cats With Presumed and Confirmed Urogenital Neoplasia

### **Jillian Yant** ^1^; Annette Folgueras^2^, VMD; JD Foster^1^

#### ^1^Friendship Hospital for Animals, Fort Collins, CO, USA; ^2^Rotating Intern, Friendship Hospital for Animals, Fort Collins, CO, USA

**Background:** The urine protein creatinine (UPC) ratio is the gold standard test for quantifying proteinuria. Sources of proteinuria may occur from pre‐renal, intrinsic renal, and post‐renal sites, and some patients may have multiple sources of proteinuria. While urogenital neoplasia is known to cause post‐renal proteinuria, the magnitude has not been evaluated.

**Hypothesis/Objectives:** To evaluate urinalysis and UPC findings in dogs with urogenital neoplasia.

**Methods:** A retrospective study identified patients seen at Friendship Hospital for Animals with confirmed or suspected urogenital neoplasia. Cancer was confirmed via urine cytology, positive BRAF test, or histopathology of the lesion. Animals were suspected to have urogenital neoplasia when ultrasound identified a mass‐lesion suggestive of urothelial carcinoma. Animals were eligible for enrollment if there was a urinalysis with UPC was available for review.

**Results:** A total of 122 samples from 6 felines and 47 dogs were evaluated. The median UPC was 1.04 with a range of 0.01–11.9. UPC was normal (<0.2) in 13 (10.6%) of samples and borderline proteinuric (0.2–0.5) in 16 (13.1%). 105 (71.4%) of samples were proteinuric (UPC >0.5), 33 (27.0%) had a UPC 1.0–2.0 and 29 (23.8%) had a UPC >2.0. Hypoalbuminemia (albumin <2.3) was identified in 2 (1.9%) samples. Serum albumin was not correlated to UPC. Animals with confirmed neoplasia, bacteriuria, or pyuria had higher UPCs than those without.

**Conclusions and Clinical Importance:** Proteinuria is common in animals with confirmed and suspected urogenital neoplasia. Further investigation is warranted to determine if there is glomerular disease or only post‐renal proteinuria.

## Abstract NU10: Urinary Syndecan‐1 in Dogs Anesthetized With Isoflurane or Sevoflurane: A Randomized, Prospective Study

### **Stephanie Harris** ^1^; Stuart Clark‐Price^2^; Kathy Gerken^3^; Wan‐Chu Hung^3^; Kendon Kuo^3^; Maureen McMichael^4^; Amy Yanke^3^

#### ^1^Auburn University, Auburn, AL, USA; ^2^Anesthesia/Pain Management, College of Veterinary Medicine, Auburn University, Auburn, AL, USA; ^3^College of Veterinary Medicine, Auburn University, Auburn, AL, USA; ^4^Emergency & Critical Care, College of Veterinary Medicine, Auburn University, Auburn, AL, USA

**Background:** Syndecan‐1 (SDC1) is an established marker of endothelial glycocalyx (EG) shedding. Most of the research on SDC1 has focused on plasma or serum, and little is known about urinary levels.

**Objectives:** To measure urinary SDC1 in dogs undergoing anesthesia with either sevoflurane or isoflurane; to compare urinary SDC1 to length of anesthesia and quantity of IV crystalloids.

**Animals:** Thirty‐one client‐owned dogs undergoing anesthesia for MRI +/‐ surgery for suspected intervertebral disc disease (IVDD).

**Methods:** Dogs with suspected IVDD were randomized to undergo anesthesia with either sevoflurane or isoflurane. Urine was collected prior to, and immediately after anesthesia for analysis of SDC1. Urinary creatinine levels were also measured and the ratio of urinary SDC1 to urinary creatinine (USCR) was used to account for dilution.

**Results:** Median (range) urinary SDC1 was significantly higher after anesthesia compared with baseline for all groups combined (*P* < 0.05). There was no significant difference between groups for age, sex, weight, and type of anesthesia. A multiple regression analysis of the effect of the independent variables inhalant type, age, weight, sex, anesthesia time, surgery, and quantity of IV fluids on the dependent variable, SDC1, found that only quantity of IV fluids significantly predicted a change (*P* < 0.0001).

**Conclusions and Clinical Importance:** Total volume of crystalloid fluids administered to anesthetized dogs may impact SDC1 in urine. Further investigations are warranted to evaluate the relationship between IV fluids and vascular/urinary SDC1.

## Abstract NU11: Effect of Iodinated Non‐ionic Contrast on Cultures of Canine Urine Inoculated With *E. coli*

### **Maxime G. Derré** ^1^; Sarah Steinbach^2^, DVM, DACVIM; Larry Adams^3^, DVM, PhD, DACVIM; Kenitra Hendrix^1^, DVM, PhD, DACVM; Meggan Stanton^1^, BS

#### ^1^Purdue University, West Lafayette, IN, USA; ^2^Uro‐Nephrology, Purdue University, West Lafayette, IN, USA; ^3^Professor, Uro‐Nephrology, Purdue University, West Lafayette, IN, USA

**Background:** The effect of iodinated contrast media on bacterial growth has been investigated in human medicine; however, results from scientific studies are conflicting and evidence in veterinary medicine is lacking.

**Objective:** To investigate the effect of iohexol on standardized quantitative urine culture results in dogs. The authors hypothesized that presence of iohexol in inoculated urine samples would result in lower bacterial concentrations (CFU/ml), and therefore decrease culture sensitivity.

**Samples:** Urine sample aseptically collected during cystoscopy from a single client‐owned dog untreated with antibiotics.

**Procedures:** Experimental controlled study. The urine sample was divided into 38 aliquots (0.5 ml each) which were used as negative controls or inoculated with an equal amount of *E. coli* (10^5^ CFU/ml). Different volumes (0.1 and 0.5 ml) of contrast or saline were added to the aliquots and quantitative culture results were compared. Two different incubation times between preparation of aliquots and culture were evaluated (15 min and 24 h).

**Results:** All aliquots from samples inoculated with *E. coli* (positive controls and iohexol group) had the same reported quantitative result (10^4^ CFU/ml). No growth was reported for the negative controls. Iohexol did not show any anti *E. coli* properties in canine urine cultures for dilutions up to 1:2 contrast:urine and concentrations up to 120 mgI/ml. No difference was reported when iohexol was incubated with inoculated urine for 15 min or 24 h.

**Clinical Relevance:** Based on the experimental *in vitro* conditions described, administration of iohexol prior to collection of urine during urologic procedures does not negatively impact the isolation and growth of *E. coli*.

**Table:** Table 1. Clinicopathologic findings of the client‐owned dog used in the study

Test	Result	Reference range	Units
Serum creatinine	1.1	<1.4	mg/dl
USG	1.023		
pH	8.0	6.0–8.0	
Protein	Negative	Negative	
Glucose	Negative	Negative	
Ketone	Negative	Negative	
Bilirubin	Negative	Negative to 1+	
RBC	3–4	0–5	/HPF
WBC	0–2	0–5	/HPF
Bacteria	Negative	Negative	
Urine culture	No growth	No growth	
Bladder wall culture	No growth	No growth	
*Mycoplasma* spp. PCR	Negative	Negative	

Blood and urine samples were collected (via venipuncture and cystocentesis, respectively) the day preceding cystoscopy. HPF: high‐power field, RBC: red blood cell, USG: urine specific gravity, WBC: white blood cell.

**Table:** Table 2. Characteristics of aliquots and concentration of *E. coli* (CFU/ml) obtained for each aliquot studied

Aliquot group	Composition	Inoculation‐to‐culture time	Purpose	Number of aliquots	Quantitative urine culture (CFU/ml)
**1**	Urine (0.5 ml)	15 min	Negative control	2	0
**2a**	Urine (0.5 ml) + saline (0.1 ml)		Negative controls used to confirm that addition of saline did not cause contamination	1	0
**2b**	Urine (0.5 ml) + saline (0.5 ml)			1	0
**3a**	Urine (0.5 ml) + iohexol (0.1 ml)		Negative controls used to confirm that addition of contrast did not cause contamination	1	0
**3b**	Urine (0.5 ml) + iohexol (0.5 ml)			1	0
**4**	Urine (0.5 ml) + *E. coli* (10^5^ CFU/ml)	15 min	Positive control	2	10^4^
**5a**	Urine (0.5 ml) + *E. coli* (10^5^ CFU/ml) + saline (0.1 ml)	15 min	Positive controls used to evaluate influence of dilution caused by addition of saline	5	10^4^
**5b**	Urine (0.5 ml) + *E. coli* (10^5^ CFU/ml) + saline (0.5 ml)			5	10^4^
**5c**	Urine (0.5 ml) + *E. coli* (10^5^ CFU/ml) + saline (0.5 ml)	24 h		5	10^4^
**6a**	Urine (0.5 ml) + *E. coli* (10^5^ CFU/ml) + iohexol (0.1 ml)	15 min	To evaluate effect of iohexol (1:6 dilution) on quantitative urine culture results	5	10^4^
**6b**	Urine (0.5 ml) + *E. coli* (10^5^ CFU/ml) + iohexol (0.5 ml)		To evaluate effect of iohexol (1:2 dilution) on quantitative urine culture results	5	10^4^
**6c**	Urine (0.5 ml) + *E. coli* (10^5^ CFU/ml) + iohexol (0.5 ml)	24 h	To evaluate effect of iohexol (1:2 dilution) contact time with *E. coli* on urine culture results	5	10^4^

CFU: Colony‐forming units

## Abstract NU12: Low‐Dose Radiation Therapy: A Novel Treatment for Feline Idiopathic/Interstitial Cystitis

### **Allison Kendall** ^1^; Shelly Vaden^2^; Tonya Harris^2^; Brittany Enders^2^; Michael Nolan^2^

#### ^1^North Carolina State University, Raleigh, NC, USA; ^2^College of Veterinary Medicine, North Carolina State University, Raleigh, NC, USA

**Background:** Feline idiopathic cystitis (FIC) accounts for the majority of feline lower urinary tract (LUT) disease. Severe morbidity and frequency of urethral obstruction (UO) often results in euthanasia. Despite many studies, severely affected cats remain difficult to manage. Low doses of radiation therapy (RT), which effectively reduce pain and inflammation in other disorders, has not been studied.

**Hypothesis/Objectives:** The purpose of this study is to determine the feasibility of using a single fraction of low‐dose RT to reduce rate of re‐obstruction and recurrence of clinical signs in cats with FIC.

**Animals:** Five client‐owned male cats with recent history of severe FIC and/or recurrent UO that had been managed with environmental modification and/or pharmacological management.

**Method:** Single‐arm clinical trial to establish proof‐of‐concept for low‐dose RT. All cats were anesthetized and received a total of 6 Gy in a single fraction to the urinary tract. A standardized questionnaire was administered to the owners at baseline and then every 30 days for up to one year.

**Results:** All cats tolerated RT with no adverse effects. Two cats completed one year of follow‐up. To date, none of the cats have had recurrence of LUT signs or UO.

**Conclusion and Clinical Importance:** This pilot study concludes that a single low‐dose fraction of RT to the urinary tract is a safe and viable treatment option for cats with FIC. In addition to environmental modification, low‐dose RT may reduce the severity and frequency of clinical signs associated with FIC in cats.

## Abstract NU13: Placement of an Artificial Urethral Sphincter in 6 Male Dogs With Urethral Diverticulum

### **Geoffrey Neumann** ^1^; Catherine Vachon^2^, DVM, DVSc, DACVIM; Carrie Palm^3^; William T.N. Culp^3^; Marilyn Dunn^2^, DVM, MSc, DACVIM

#### ^1^University of Montreal, Montreal, Quebec, Canada; ^2^Interventional Radiology, University of Montreal, Montreal, Quebec, Canada; ^3^School of Veterinary Medicine, University of California–Davis, Davis, CA, USA

**Background:** Urethral diverticulum (UD) is a rare anatomical anomaly defined as an outpouching of the urethra. Management without surgical resection has not been previously described in dogs.

**Hypothesis/Objectives:** The objective of this study was to report outcome of male dogs presented for urinary incontinence with UD treated with an artificial urethral sphincter (AUS).

**Animals:** Six client‐owned dogs with UD treated with AUS were identified.

**Methods:** Medical records from male dogs with urinary incontinence were reviewed. Inclusion criteria were retrograde cystourethrography and/or cystoscopy, abdominal ultrasonography or contrast CT demonstrating a urethral diverticulum, AUS placement and at least one follow‐up after AUS placement.

**Results:** Median age at time of diagnosis was 9 months and median weight was 32.9 kg. Median continence score at presentation was 1/5 (1/5 in 5/6 dogs and 2/5 in 1/6 dogs). A contrast cystourethrogram was performed and diagnostic in 5/5 dogs (fluoroscopic *n =* 4, CT cystourethrography *n =* 1). All diverticula were located in the proximal third of the urethra. A congenital origin was suspected in 5 dogs and acquired in 1. Concomitant anomalies included bilateral cryptorchidism (2/6) and ultrasound findings consistent with renal dysplasia or chronic pyelonephritis (3/6), persistent urachus (1/6,) ventrolateral insertion of the ureterovesicular junctions (1/6), and urethrocolonic fistula (1/6). All dogs were poorly/moderately responsive to phenylpropanolamine. AUS placement resulted in improvement in all dogs with a median continence score of 4/5 (5/5 in 2/6 dogs, 4/5 in 3/6 dogs, 3/5 in 1/6 dogs) post‐placement.

**Conclusion:** Placement of an AUS improved continence scores in male dogs with UD.

## Abstract NU14: Neutrophil Gelatinase‐Associated Lipocalin As Biomarker of Acute Kidney Injury in Dogs With Congestive Heart Failure

### **Maria Chiara Sabetti** ^1^; Francesca Fidanzio^1^; Serena Crosara^1^; Roberta Troia^2^; Cecilia Quintavalla^1^; Giovanni Romito^2^; Linda Perissinotto^2^; Chiara Mazzoldi^2^; Sabrina Fasoli^2^; Francesco Dondi^2^

#### ^1^University of Parma, Parma, Italy; ^2^University of Bologna, Bologna, Italy

**Background:** Dogs with acute congestive heart failure (CHF) due to myxomatous mitral valve disease (MMVD) can develop acute kidney injury (AKI); its characterization and prevalence have not been clearly defined.

**Hypothesis/Objectives:** To assess the occurrence of AKI in hospitalized dogs with acute CHF and the role of urinary neutrophil gelatinase‐associated lipocalin (uNGAL) as a predictive marker of AKI in this setting.

**Animals:** Thirty MMVD dogs with acute CHF.

**Methods:** Multicentric, prospective observational study. Type and dosage of diuretics administered, serum and urinary chemistry including uNGAL and uNGAL to urinary creatinine ratio (uNGALC) were determined at time of admission, after 24 and 48 h. Continuous variables were compared using Friedman test. Mann‐Whitney test was used to compare AKI vs. non‐AKI dogs (*P* < 0.05).

**Results:** Nineteen dogs (63%) developed AKI according to International Renal Interest Society grading: 10/19 (53%) grade I, 7/19 (37%) grade II and 2/19 dogs (10%) grade III. At admission, 16/30 dogs had increased uNGAL and 23/30 had increased uNGALC, of which 10/16 (62.5%) and 15/23 (65.2%) developed AKI, respectively. No significant differences were documented for creatinine, uNGAL, uNGALC at admission and diuretic dosage between dogs that developed AKI vs. dogs that had not developed AKI. Urinary NGAL and uNGALC were not different at the considered time points (Table 1, 2).

**Conclusions and Clinical Importance:** In our population of MMVD dogs with acute CHF, AKI is a frequent finding not correlated with diuretic dosage. The role of NGAL and its prognostic value needs further investigations.

**Table:** Table 1. Demographic and clinicopathological data in dogs that developed AKI or not during hospitalization

	AKI (*n =* 19)	Non‐AKI (*n =* 11)	*P* value
Age (years)	12.1 ± 1.8	11.9 ± 2.7	0.81
Number of dogs receiving PO furosemide at home	10/19	5/11	0.68
Dosage of furosemide (mg/kg/day)	4 (3–6)	4 (2.5–5.8)	0.8
Days of therapy	238 (24–563) (*n =* 5)	224 (26–459) (*n =* 8)	1.00
Number of dogs receiving PO torasemide at home	1/19	2/11	
Dosage of torasemide (mg/kg/day)	0.22	0.45 (0.4–0.5)	
Number of dogs receiving PO pimobendan at home	13/19	8/11	
Dosage of pimobendan (mg/kg/day)	0.6 (0.25–0.8)	0.5 (0.25–0.9)	0.66
Number of dogs receiving PO spironolactone at home	3/19	4/11	
Dosage of spironolactone (mg/kg/day)	2.3 (1.5–3)	2 (1.8–2.5)	0.72
Number of dogs receiving PO ACEi at home	9/19	2/11	
Dosage of ACEi (mg/kg/day)	0.7 (0.3–1.1)	0.47 (0.25–0.7)	0.28
			
Hours of hospitalization	50 (30–288)	50 (30–120)	0.72
Total dosage of furosemide administered in the first 24 h of hospitalization (mg/kg)	11.1 (6–22)	12 (8–12.4)	0.94
Total dosage of furosemide administered in the first 48 h of hospitalization (mg/kg)	18 (14–21)	18 (16–19)	0.74
Days of survival after discharge	188 (2–735)	50 (2–350)	0.14
			
Albumin (g/dL)	2.99 ± 0.31	3 ± 0.54	0.92
Cl (mEq/L)	109.8 ± 4.9	108.8 ± 4.9	0.64
Creatinine (mg/dL)	0.92 (0.32–2)	1.18 (0.74–2.14)	0.3
CRP (mg/dL)	3.8 (1–28.9)	3.14 (1.6–20)	0.78
K (mEq/L)	4.4 (2.9–5)	4.5 (3.4–5.8)	0.34
Na (mEq/L)	151 (138–156)	149 (147–156)	0.89
Total protein (g/dL)	6.37 ± 0.7	6.37 ± 0.42	0.99
UPC	0.28 (0.08–2.6)	0.31 (0.14–2.7)	0.34
Urea (mg/dL)	59 (28–200)	59.8 (28–125)	0.53
uNGAL (pg/mL) at admission	2774 (50–42565)	9216 (262–124994)	0.20
uNGALC (pg/mL) at admission	4243 (94–315514)	25361 (746–1413967)	0.43
uNGAL (pg/mL) at 24 h after admission	3779 (400–59685)	805 (27–148765)	0.57
uNGALC (pg/mL) at 24 h after admission	6672 (768–65372)	3203 (76.4–1565945)	0.9
uNGAL (pg/mL) at 48 h after admission	2987 (29.4–50537)	262 (67.7–51708)	0.23
uNGALC (pg/mL) at 48 h after admission	6338 (225–75181)	825 (326–193448)	0.35

*Note*: Data were reported as median and range (minimum–maximum value) or mean ± standard deviation (SD), based on their distribution.

Abbreviations: AKI, acute kidney injury; ACEi, angiotensin converting enzyme inhibitor; CRP, C‐reactive protein; K, potassium; Na, sodium; UPC, urinary proteins to urinary creatinine ratio; uNGAL, urinary neutrophil gelatinase associate lipocalin; uNGALC, uNGAL to urinary creatinine ratio.

**Table:** Table 2. Clinicopathological variables at time of admission (T0), after 24 h (T1) and 48 h (T2)

	T0 (admission)	T1 (24 h)	T2 (48 h)	*P* value
Serum creatinine (mg/dL)	0.92 (0.32–2)^a,b^	1.37 (0.9–3.5)	1.4 (0.75–4.49)	0.001
Urea (mg/dL)	59.4 (28–200)^a,b^	105.6 (40–235)	101 (49–159)	0.01
uNGAL (pg/mL)	3322 (50–124994)	2897 (27–149864)	1438.6 (29.4–51708)	0.24
uNGALC (pg/mL)	6578 (94.9–1413967)	4708 (76.4–1565945)	3600 (225–193448)	0.82

*Note*: Data were reported as median and range (minimum–maximum value).

Abbreviations: uNGAL, urinary neutrophil gelatinase associate lipocalin; uNGALC, uNGAL to urinary creatinine ratio.

^a^ Significantly different between T0 and T1

^b^ Significantly different between T0 and T2

## Abstract NU15: Urinary Incontinence in Male Dogs

### **Tanner Slead** ^1^; Shelly Vaden^2^, DVM, PhD, DACVIM (SAIM); Tonya Harris^2^; Allison Kendall^2^, DVM, MS, DACVIM (SAIM)

#### ^1^North Carolina State Veterinary Hospital, North Carolina State University, Raleigh, NC, USA; ^2^College of Veterinary Medicine, North Carolina State University, Raleigh, NC, USA

**Background:** Urinary incontinence in male dogs is a poorly characterized disorder. Incontinence patterns are often used to establish differential diagnoses in female dogs. However, in male dogs, this often results in inaccurate conclusions regarding which component of the lower urinary tract is affected, propagating inappropriate treatment recommendations.

**Objective:** To 1) determine the incidence of storage vs. voiding micturition disorders in male dogs presenting for urinary incontinence and 2) report the post‐void residual volume (PVR) measurements for dogs in each category.

**Animals:** 35 client‐owned male dogs presenting for urinary incontinence.

**Methods:** Retrospective case series. Signalment, presenting complaint, treatments, PVR measurements, and working diagnoses were recorded. PVR measurements were obtained via 3D ultrasound or urethral catheterization.

**Results:** Eighteen dogs were diagnosed with disorders of storage (urethral sphincter mechanism incompetence *n =* 16, ectopic ureters *n =* 3) with a median age of 3 years (range 6 m–10 y) and median weight of 28.2 kg (range 13.9–44.1 kg). Fifteen dogs were diagnosed with disorders of voiding (detrusor‐urethral dysfunction *n =* 13, lower motor neuron bladde*r =* 2), with a median age of 7 years (range 1.5–10 y) and median weight of 37.5 kg (range 9.5–72.2 kg). One dog was diagnosed with behavioral incontinence and one with partial urethral obstruction. All dogs with disorders of storage had PVR <1.1 ml/kg and all dogs with disorders of voiding had PVR >2.3 ml/kg (*P* = <0.001).

**Conclusions and Clinical Importance:** Disorders of both storage and voiding are common causes of urinary incontinence in male dogs. Differentiation can be facilitated via PVR measurement.

## Abstract NU16: A Pilot Study to Identify Plasma Calciprotein Particles in Cats With Chronic Kidney Disease

### **Pak Kan Tang** ^1^; Rebecca Geddes^2^; Rosanne Jepson^2^; Yu‐Mei Chang^3^; Makoto Kuro‐o^4^; Miki Tsuchida^4^; Jonathan Elliott^5^

#### ^1^Royal Veterinary College, London, England, UK; ^2^Department of Clinical Science and Services, Royal Veterinary College, London, England, UK; ^3^Research Support Office, Royal Veterinary College, London, England, UK; ^4^Division of Anti‐Aging Medicine, Center for Molecular Medicine, Jichi Medical University, Shimotsuke, Tochigi, Japan; ^5^Department of Comparative Biomedical Science, Royal Veterinary College, London, England, UK

**Background:** Aggregation of fetuin‐A with calcium‐phosphate mineral allows calciprotein monomers (low‐density calciprotein particles [L‐CPP]) to form. Integration with further protein consolidates L‐CPP to amorphous CPP which transform spontaneously into pro‐inflammatory crystalline CPP over time (high‐density CPP [H‐CPP]).

**Hypothesis/Objectives:** To identify different forms of CPP in feline plasma and evaluate their relationship with chronic kidney disease‐mineral and bone disorder (CKD‐MBD) parameters.

**Animals:** 15 euthyroid client‐owned cats with International Renal Interest Society (IRIS) stage 2–3 azotaemic CKD.

**Methods:** Retrospective cross‐sectional study. Frozen heparinized fasted plasma samples from CKD cats stabilized on a phosphate‐restricted diet (constituting ≥90% of diet consumed) were selected for the identification and quantification of CPP. Total CPP (T‐CPP), L‐CPP and H‐CPP were measured using an infrared fluorescent bisphosphonate (OsteoSense), in conjunction with a gel filtration method. Pearson correlation (r) was performed to evaluate the relationship of CPP with CKD‐MBD parameters.

**Results:** Low‐density CPP was identified as the predominant form of circulating CPP in feline plasma. Moderate to strong correlations were found among various forms of CPP (T‐CPP, L‐CPP and H‐CPP) with total calcium (tCa), ionized calcium (iCa), calcium‐phosphate product (CaPP) and fibroblast growth factor 23 (FGF23; Table 1); FGF23 was positively associated with tCa (*r =* 0.8; *P* = 0.005) and iCa (*r =* 0.77; *P* = 0.009).

**Conclusions and Clinical Importance:** Plasma tCa and iCa were found to be major predictors of CPP measured in fasted samples, supporting the importance of monitoring calcium status in CKD cats. The clinical implication and prognostic value associated with CPP in CKD cats remain to be investigated.

**Table:** Table 1. Pearson correlation (*r*) between the different forms of calciprotein particles (CPP) and various CKD‐MBD parameters in cats with CKD

	T‐CPP	L‐CPP	H‐CPP
Total calcium (tCa)	***r =* 0.86; *P* < 0.001**	***r =* 0.83; *P* < 0.001**	***r =* 0.93; *P* < 0.001**
Ionized calcium (iCa)	***r =* 0.87; *P* < 0.001**	***r =* 0.83 *P* < 0.001**	***r =* 0.92; *P* < 0.001**
Phosphate	*r =* 0.34; *P* = 0.237	*r =* 0.35; *P* = 0.222	*r =* 0.3; *P* = 0.305
Calcium‐phosphate product (CaPP)	***r =* 0.71; *P* = 0.004**	***r =* 0.7; *P* = 0.005**	***r =* 0.72; *P* = 0.004**
Creatinine	*r =* −0.38; *P* = 0.18	*r =* −0.4; *P* = 0.16	*r =* −0.32; *P* = 0.26
Total magnesium	*r =* −0.18; *P* = 0.734	*r =* −0.15; *P* = 0.783	*r =* −0.33; *P* = 0.524
Fibroblast growth factor (FGF23)	***r =* 0.85; *P* < 0.001**	***r =* 0.85; *P* = 0.001**	***r =* 0.87; *P* < 0.001**
Parathyroid hormone (PTH)	*r =* −0.27; *P* = 0.486	*r =* −0.27; *P* = 0.481	*r =* −0.26; *P* = 0.498

Significant correlations (*P* < 0.05) are highlighted in bold.

## Abstract NU17: Urinary Extracellular Vesicle Excretion Rate and Uromodulin Abundance in Cats With Calcium Oxalate Urolithiasis

### **Tim L. Williams**; Eve Price; Robin Antrobus; Fiona Karet Frankl

#### University of Cambridge, Cambridge, England, United Kingdom

**Background:** The pathogenesis of feline calcium oxalate urolithiasis remains enigmatic, although renal tubular injury secondary to endocytosis of intraluminal crystals is postulated to lead to formation of calcium oxalate uroliths in humans. Urinary extracellular vesicles (UEV) could act as a nidus for crystal formation, thus contributing to stone risk. Uromodulin is an inhibitor of crystal formation, hence reduced abundance of UEV‐associated uromodulin might increase heterogeneous nucleation and stone risk.

**Hypothesis:** UEV excretion rates are higher, and abundance of UEV‐associated uromodulin is reduced in cats with urolithiasis.

**Animals:** 7 cats with presumed calcium oxalate urolithiasis and 13 control cats.

**Methods:** UEVs were isolated from frozen, 0.5–3 mL urine samples by ultrafiltration and size exclusion chromatography. UEV concentrations were determined by nanoparticle tracking analysis and normalized to urine volume and urine creatinine concentration. UEV‐associated uromodulin abundance was determined by liquid chromatography tandem mass‐spectrometry. Data are presented as median [range].

**Results:** UEV excretion rates in female cats with uroliths were higher than in female controls (2.6 × 10^5^ [1.3 × 10^5^–7.3 × 10^5^] UEVs/mL:μmol/L, *n =* 5 vs. 5.13 × 10^4^ [1.1 × 10^4^–1.6×10^5^] UEVs/mL:μmol/L, *n =* 4; *P* = 0.03). This was not apparent in male cats (with uroliths [*n =* 2]: 3.2 × 10^4^ and 6.9 × 10^4^ UEVs/mL:μmol/L vs. without uroliths: 9.8 × 10^4^ [1.1 × 10^4^–6.0 × 10^5^] UEVs/mL:μmol/L [*n =* 9]). Uromodulin was detectable in UEVs of 5/10 control cats but in none of 4 cats with urolithiasis (*P* = 0.22).

**Conclusions and Clinical Importance:** These preliminary data suggest that UEV excretion rate and potentially the abundance of UEV‐associated uromodulin could contribute to the pathogenesis of calcium oxalate urolithiasis in cats, although larger studies are needed to confirm these findings.

## Abstract NU18: Use of a Pediatric Renal Replacement System (CARPEDIEM) for Extracorporeal Therapy in Dogs

### **Karina J. Creighton**; J.D. Foster, VMD, MS, DACVIM (SAIM)

#### Friendship Hospital for Animals, Washington, D.C., USA

**Background:** Small patients have a large proportion of their blood volume (BV) in the extracorporeal circuit (EC) when undergoing extracorporeal therapies using adult human systems. This leads to a much higher incidence of hemodynamic instability during treatment and increased use of blood products to prime the EC. The CARPEDIEM system is designed for children <10 kg and can be used to perform renal replacement therapy (RRT) and therapeutic plasma exchange (TPE).

**Objective:** To report the use of the CARPEDIEM system in small dogs.

**Animals:** Four dogs undergoing RRT and 2 dogs undergoing TPE using the CARPEDIEM system.

**Methods:** This is a retrospective study. Dogs who received RRT or TPE using the CARPEDIEM system were included. The following information was recorded from the medical record: patient weight, PCV before and after treatment, percentage of BV in EC, EC priming solution, hemodynamic parameters during treatment, and urea reduction ratio (URR) where applicable.

**Results:** Six RRT treatments were performed in 4 dogs. Two dogs had 1 TPE treatment each. Median body weight was 4.1 kg (range 2.89–9.9). Median BV in EC was 10.3% (range 3.7–17.9). Median delta PCV was 1% (range −11–7). Median URR for 5/6 RRT treatments was 0.58 (range 0.37–0.65). No patient required a blood prime. Fluid‐responsive hypotension was noted during 1 RRT treatment. Transient hypotension that resolved without intervention was noted in both TPE treatments.

**Conclusions and Clinical Importance:** The CARPEDIEM system can be used to provide safe RRT and TPE in small patients.

## Abstract NU19: Urinary Fractional Excretion of Calcium in Cats With Hypercalcemia or Normocalcemia

### **Fernanda C. Chacar** ^1^; Fabio Teixeira^2^, DVM, PhD; Clara Mori^2^; Bruna Ruberti^2^, DVM, MSc; Archivaldo Reche, Jr.^3^, DVM, PhD; Marcia Kogika^3^, DVM, PhD; Marcio Brunetto^4^, DVM, PhD

#### ^1^School of Veterinary Medicine and Animal Science, University of Sao Paulo, Sao Paulo, Brazil; ^2^University of Sao Paulo, Sao Paulo, Brazil; ^3^Professor, Internal Medicine, University of Sao Paulo, Sao Paulo, Brazil; ^4^Professor, University of Sao Paulo, Sao Paulo, Brazil

**Background:** Cats with hypercalcemia may have hypercalciuria, which may increase the risk to nephrolithiasis or renal mineralization.

**Objective:** To compare the urinary fractional excretion of calcium (uFECa) of cats with hypercalcemia or normocalcemia.

**Animals:** Thirty‐two cats were prospectively recruited from the Veterinary Teaching Hospital of the University of Sao Paulo (2016–2019) based on the levels of blood ionized calcium (iCa).

**Methods:** Case‐control study. Cats with iCa ≥1.4 mmol/L at three timepoints of evaluation were allocated into the hypercalcemic group (*n =* 13), and those with normal iCa levels (1.1 mmol/L ≤ iCa < 1.4 mmol/L) were assigned to the normocalcemic group (*n =* 19). uFECa was then assessed. After normality test (Shapiro‐Wilk), Mann‐Whitney and Spearman correlation were applied, considering *P* ≤ 0.05.

**Results:** Median iCa levels were (min–max) 1.46 (1.41–1.53) mmol/L and 1.34 (1.17–1.39) mmol/L in cats with hypercalcemia and normocalcemia, respectively (*P* < 0.0001). Hypercalcemic cats showed 0.42% (0.09–2.46) and normocalcemic cats had 0.20% (0.03–1.11) of uFECa (*P* = 0.06). No correlation was found between iCa and uFECa. Based on ultrasonographic results, nephrolithiasis was found in 14/19 (73.7%) of normocalcemic cats and 7/13 (53.8%) of hypercalcemic cats, and there were no differences in uFECa comparing cats with kidney stones with hypercalcemia or normocalcemia.

**Conclusions and Clinical Importance:** There were no differences in uFECa in cats with hypercalcemia or normocalcemia; further studies with larger sample sizes and prospective design are necessary to confirm this finding.

**Figure:**
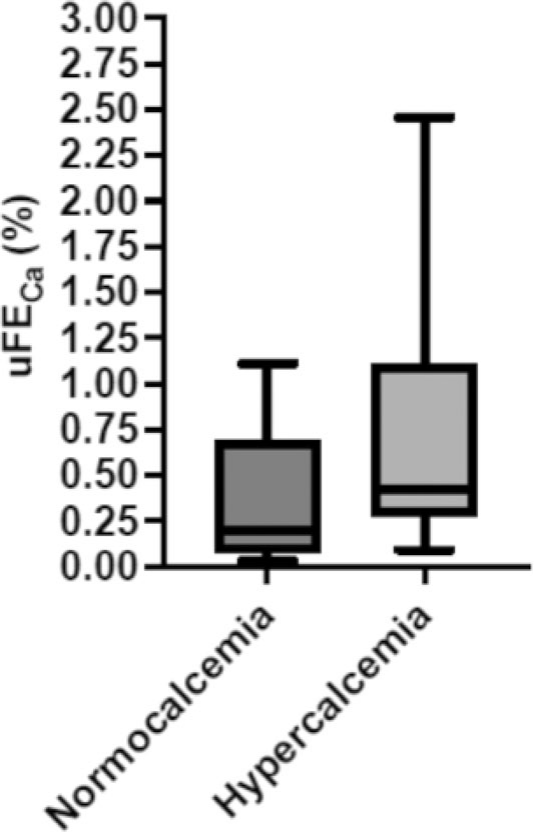
Figure 1. Urinary fractional excretion of calcium in cats with hypercalcemia or normocalcemia**.** Note: uFECa% urinary fractional excretion of calcium.

## Abstract NU20: Urinary Fractional Excretion of Phosphorus in Cats With Chronic Kidney Disease

### **Fernanda C. Chacar** ^1^; Fabio Teixeira^2^, DVM, PhD; Clara Mori^2^; Bruna Ruberti^2^; Archivaldo Reche, Jr.^2^; Marcia Kogika^2^; Marcio Brunetto^2^

#### ^1^School of Veterinary Medicine and Animal Science, University of Sao Paulo, Sao Paulo, Brazil; ^2^University of Sao Paulo, Sao Paulo, Brazil

**Background:** In addition to serum phosphate (sP) and FGF‐23, the urinary fractional excretion of phosphorus (uFEP) may be used to evaluate the mineral and bone disorders in chronic kidney disease (MBD‐CKD) in cats.

**Objective:** To evaluate the sP and uFEP in CKD cats (IRIS stages 2, 3 and 4).

**Animals:** Cats with CKD with stage 2 (*n =* 10), Stage 3 (*n =* 8) and stage 4 (*n =* 6) undergoing conservative treatment in accordance with IRIS guidelines at the Veterinary Teaching Hospital.

**Methods:** Serum and urine samples were collected at the same time‐point to sP and uFEP determinations, after 12‐h fasting. The normal cut off values of sP <4.6 mg/dL and uFEP <40% were considered to the analysis. ANOVA and Tukey‐Kramer test for multiple comparison were applied (*P* ≤ 0.05).

**Results:** CKD cats with stages 3 and 4 showed lower levels of uFEP (14.86 ± 7.5% and 11.58 ± 3.15%, respectively), with higher concentrations of sP (5.66 ± 2.27 mg/dL and 9.60 ± 3.94 mg/dL, respectively). Mean values of sP and uFEP in CKD cats with stage 2 were 4.41 ± 0.86 mg/dL and 8.71 ± 2.22%, probably due to the satisfactory phosphate control by feeding renal diets.

**Conclusions and Clinical Importance:** Cats in late stage of CKD had decreased phosphaturia and hyperphosphatemia. Prospective studies evaluating sP, FGF‐23 and uFEP may help to elucidate whether impaired effect of phosphatonins due to reduced renal mass may mediate hyperphosphatemia in cats with CKD.

**Figure:**
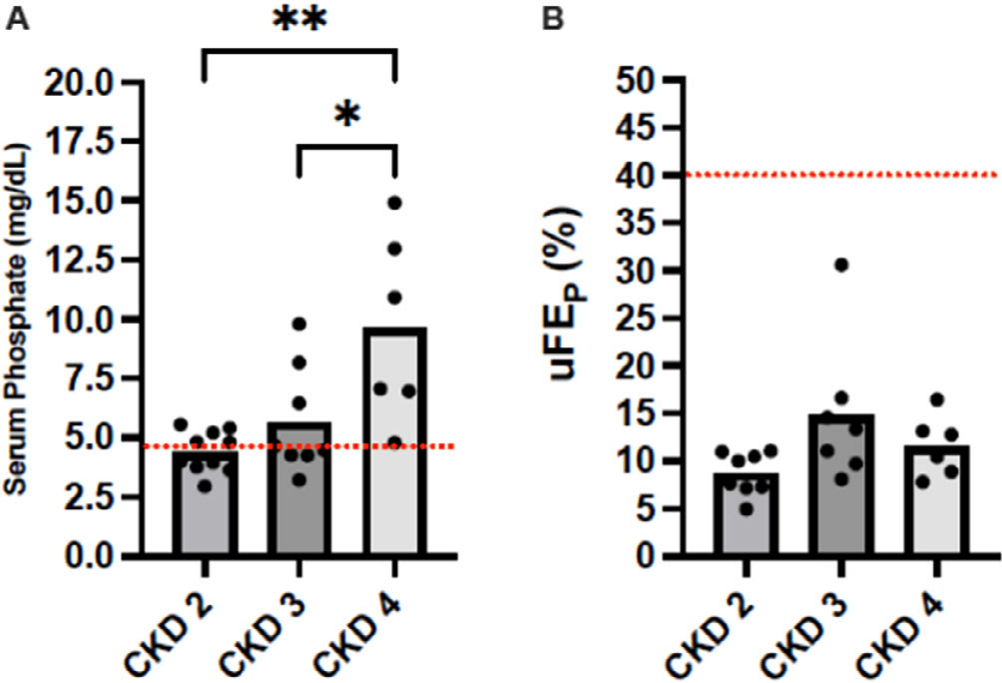
Figure 1. (A) Serum phosphate and (B) urinary fractional excretion of phosphorus in cats with CKD with stage 2, 3 and 4. * *P* < 0.01; ** *P* < 0.001; uFE_p_ (%) urinary fractional excretion of phosphorus. (A) Red line—sP <4.6 mg/dL (IRIS); (B) Red line— ^a^uFE_p_ <40% ^a^Finco et al *Am J Vet Res*. 1997;58:1184–1187.

## Abstract NU21: Traditional Approach Versus Stewart Approach for Acid‐Base Disorders in Cats With Chronic Kidney Disease

### **Fernanda C. Chacar** ^1^; Fabio Teixeira^2^, DVM, PhD; Bruna Coelho^2^, DVM; Marcia Kogika^2^, DVM, PhD

#### ^1^School of Veterinary Medicine and Animal Science, University of Sao Paulo, Sao Paulo, Brazil; ^2^University of Sao Paulo, Sao Paulo, Brazil

**Background:** Acid‐base disorders (ABDs) are common in cats with chronic kidney disease (CKD), leading to CKD progression and mortality.

**Objective:** To compare the traditional approach and the Stewart approach in the identification of ABDs in CKD cats.

**Animals:** CKD cats (IRIS stage 1, *n =* 6; stage 2, *n =* 16; Stage 3, *n =* 20; stage 4, *n =* 12) treated at the Veterinary Teaching Hospital.

**Methods:** Traditional approach (bicarbonate‐base approach and its modification using base excess, BE) and Stewart approach (strong ion difference, SID; total weak acids, ATOT; strong ion gap, SIG) were used to assess the metabolic and respiratory status in order to identify ABDs. Descriptive statistics and Spearman's correlation were performed (*P* ≤ 0.05).

**Results:** Traditional approach identified high anion gap metabolic acidosis in 10/54 (18.5%), hyperchloremic metabolic acidosis in 1/54 (1.8%), and metabolic alkalosis with respiratory compensation in 1/54 (1.8%) of cats with CKD. Stewart approach identified metabolic acidosis in 26/54 (48%) and metabolic alkalosis in 5/54 (9%) based on SID changes; metabolic acidosis based on ATOT abnormalities was found in 2/54 (3.7%). Both approaches concomitantly detected the presence of metabolic acidosis in 7/54 (13%) and metabolic alkalosis in 1/54 (1.8%) of the cases. Anion gap and SIG were positively correlated (rho = 0.58; *P* < 0.0001).

**Conclusions and Clinical Importance:** Stewart approach indicated the occurrence of ABDs more frequently than the traditional approach. However, it remains to be determined whether it could be relevant to guide clinical decisions.

**Figure:**
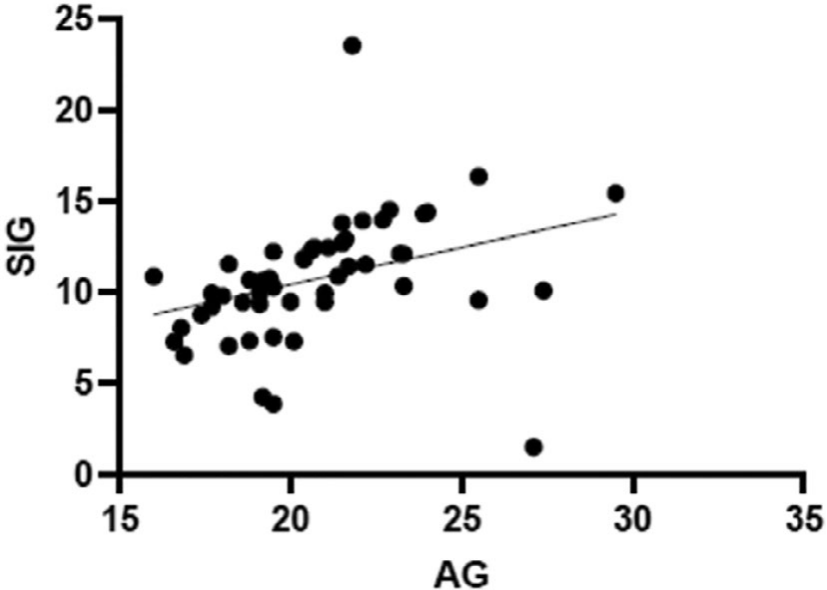
Figure 1. Spearman's correlation between anion gap and strong ion gap. **Note: AG (anion gap); SIG (strong ion gap); rho = 0.58; *P* < 0.0001.**

## Abstract NU22: Evaluation of Chronic Stress Status in Diseased Cats With Hair Cortisol Concentration and Psychometrics

### **Chien‐Hui Chen** ^1^; Ya‐Jane Lee, PhD^2^

#### ^1^National Taiwan University, Taipei City, Taiwan; ^2^Professor, Institute of Veterinary Clinical Science, School of Veterinary Medicine, College of Bio‐Resources and Agriculture, National Taiwan University, Taipei City, Taiwan

**Background:** Hair cortisol concentration (HCC) and psychometrics serve as indicators of chronic stress status and quality of life (QoL) respectively. To date, the application of both simultaneously was scarcely studied in diseased cats.

**Objectives:** To evaluate HCC and questionnaires in healthy, chronic kidney disease (CKD), and feline infectious peritonitis (FIP) cats. Meanwhile, to investigate their correlations in various aspects.

**Animals:** Client‐owned cats with healthy status (*n =* 20), CKD (*n =* 56), and suspected FIP (*n =* 20).

**Methods:** Cohort study. Hair was collected from cats’ abdominal region, and HCC was measured with a commercial enzyme‐linked immunosorbent assay (ELISA) kit. Questionnaires were fulfilled by owners, and average‐item‐weighted‐impact‐score (AWIS) was calculated.

**Results:** Suspected‐FIP (median [IQR]: 896.27 [444.05, 3921.19] pg/mg) and late‐stage‐CKD cats (271.00 [222.20, 441.36] pg/mg) had significantly higher HCC than healthy (149.94 [110.99, 198.16] pg/mg) and early‐stage‐CKD cats (158.35 [70.13, 186.12] pg/mg) (*P* < 0.05). Cats with suspected‐FIP and late‐stage‐CKD had significantly lower AWIS in general (suspected‐FIP: −2.06 [−3.41, −1.09]; healthy: 1.59 [1.18, 1.88]; early‐stage‐CKD: 0.41 [0.00, 1.12]), heath (suspected‐FIP: 2.27 [−3.82, −0.64]; healthy: 1.18 [0.73, 1.27]; early‐stage‐CKD: 0.45 [−0.18, 0.91]), behavioral (suspected‐FIP: −0.67 [−2.83, 1.17]; healthy: 7.00 [2.67, 7.00]), and medical (late‐stage‐CKD: −2.67 [−3.75, −2.42]; early‐stage‐CKD: −0.67 [−1.67, −0.33]) domains than healthy and early‐stage‐CKD cats (*P* < 0.05). HCC had significantly negative correlation with QoL in general (rho = −0.52), heath (rho = −0.50), and medical (rho = −0.53) domains (*P* < 0.05).

**Conclusions and Clinical Importance:** Cats’ chronic stress was proven significantly to affect QoL by the evaluation with objective HCC and subjective psychometrics.

**Figure:**
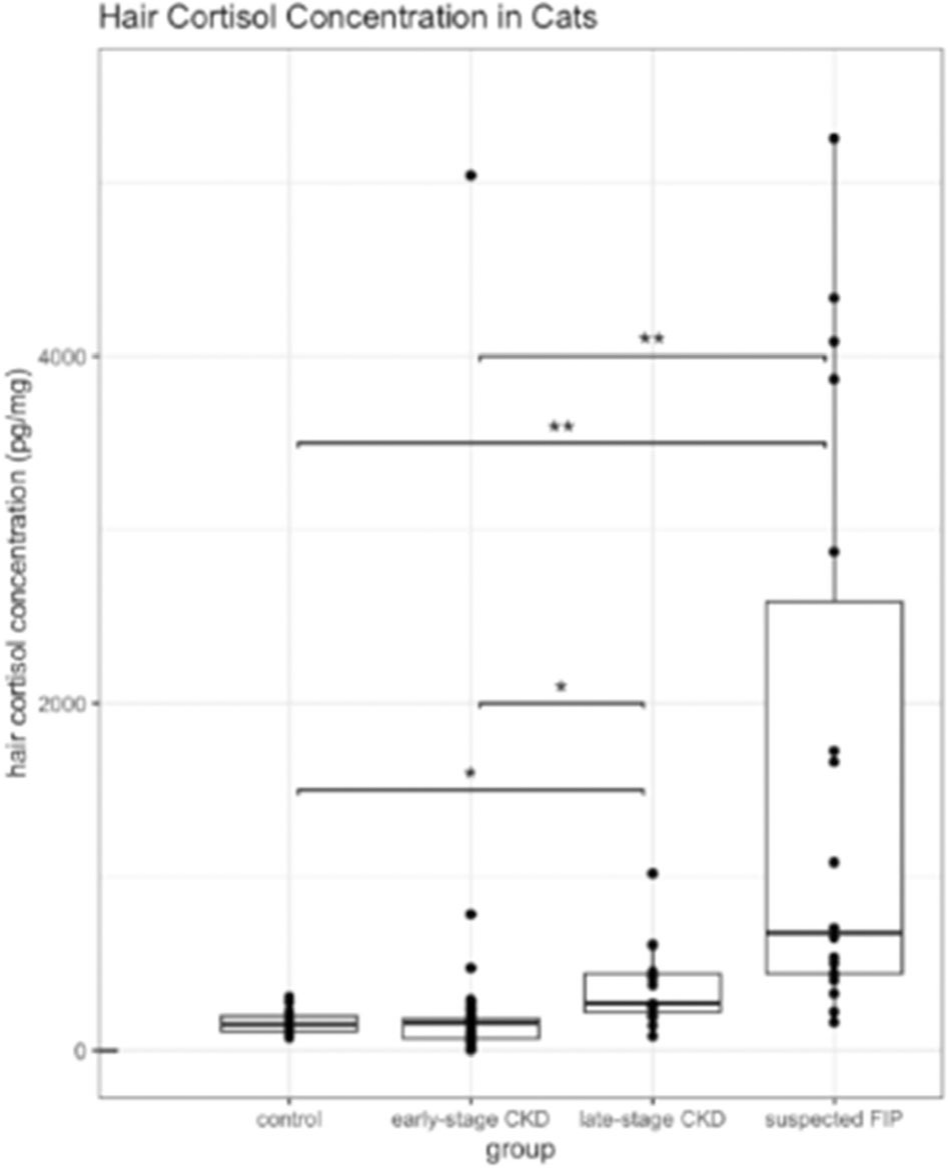
Figure 1. HCC in cats with different groups. Test was performed with Kruskal‐Wallis test. Post‐hoc analysis was performed with Dunn's test. * as *P* < 0.05; ** as *P* < 0.001. 2 outliers in suspected‐FIP group (HCC >10,000 pg/mg) were not shown in Figure 1.

**Table:** Table 1. HCC and questionnaire outcomes in cats with different groups

Parameters	Control		Early‐stage‐CKD^A^		Late‐stage‐CKD^B^		Suspected‐FIP		P
	Median (IQR)	*n*	Median (IQR)	*n*	Median (IQR)	*n*	Median (IQR)	*n*	
HCC (pg/mg)	149.94 (110.99, 198.16)	20	158.35 (70.13, 186.12)	41	271.00 (222.20, 441.36)^CD^	15	896.27 (444.05, 3921.19)^CD^	20	<0.001
AWIS in general domain	1.59 (1.18, 1.88)	5	0.41 (0.00, 1.12)	21	−0.12 (−0.75, 0.31)	10	−2.06 (−3.41, −1.09)^CD^	11	<0.001
AWIS in health domain	1.18 (0.73, 1.27)	5	0.45 (−0.18, 0.91)	21	−0.36 (−0.36, 0.61)	10	−2.27 (−3.82, −0.64)^CD^	11	0.001
AWIS in behavioral domain	7.00 (2.67, 7.00)	5	2.00 (0.67, 4.00)	21	2.83 (0.17, 4.75)	10	−0.67 (−2.83, 1.17)^C^	11	0.020
AWIS in medical domain	−0.67 (−0.67, −0.67)	5	−0.67 (−1.67, −0.33)	21	−2.67 (−3.75, −2.42)^D^	10	−2.67 (−4.00, −2.00)	11	0.004

*Note*: Test was performed with Kruskal‐Wallis test. *Post‐hoc* analysis was performed with Dunn's test. A: IRIS CKD stage I and II. B: IRIS CKD stage III and IV. C: achieved significance with control group. D: achieved significance with early‐stage‐CKD group.

## Abstract NU23: Urinary Fibroblast Growth Factor‐23 in Cats With Stable or Acute Decompensated Chronic Kidney Disease

### **Wei‐Che Chen** ^1^; Ya‐Jane Lee^2^, PhD

#### ^1^National Taiwan University, Taipei City, Taiwan; ^2^Professor, Institute of Veterinary Clinical Science, School of Veterinary Medicine, College of Bio‐Resources and Agriculture, National Taiwan University, Taipei City, Taiwan

**Background:** Elevated plasma fibroblast growth factor‐23 (FGF23) in cats with chronic kidney disease (CKD) has emerged as a prognostic biomarker. Whether increased urinary FGF23 (uFGF23) is associated with either renal diseases or the outcome is unknown.

**Objectives:** To evaluate uFGF23 in cats with renal diseases and its relationship with negative outcomes.

**Animals:** Forty CKD, 19 acute decompensated CKD (ACKD), and 14 healthy cats.

**Methods:** Case‐control study. Cats with International Renal Interest Society stage 1 and 2 and Stage 3 and 4 CKD were enrolled in the early, and late CKD groups, respectively, whereas ACKD was defined as an increase of >1 mg/dL and >20% baseline in serum creatinine concentration within 30 days. Urinary FGF23 was measured using commercial ELISA and expressed as uFGF23‐to‐creatinine ratio (uFGF23/cr).

**Results:** A significantly elevated uFGF23/cr (median [IQR]: 11.98 [7.09–19.15] pg/mg) was observed in the ACKD group compared to the control group (0.91 [0.66–1.01] pg/mg), early CKD group (2.57 [1.09–4.09] pg/mg), and late CKD group (7.01 [5.77–8.06] pg/mg, all *P* < 0.001). The odds of 30‐day mortality increased with an elevated uFGF23/cr (odds ratio = 1.57, 95% confidence interval = 1.02–2.41, *P* = 0.04) after adjusting for the CKD stage. Cats with ACKD in the high uFGF23/cr (cutoff = 17.9 pg/mg) group were associated with higher 30‐day mortality (6/6, 100%, versus 1/12, 8.3%, *P* < 0.001), and had higher BUN (116 [112–160] versus 60 [40.3–106.3] mg/dL, *P* = 0.037).

**Conclusions and Clinical Importance:** Urinary FGF23 is elevated with a decline in renal function and could be a potential biomarker to predict the prognosis of ACKD.

**Figure:**
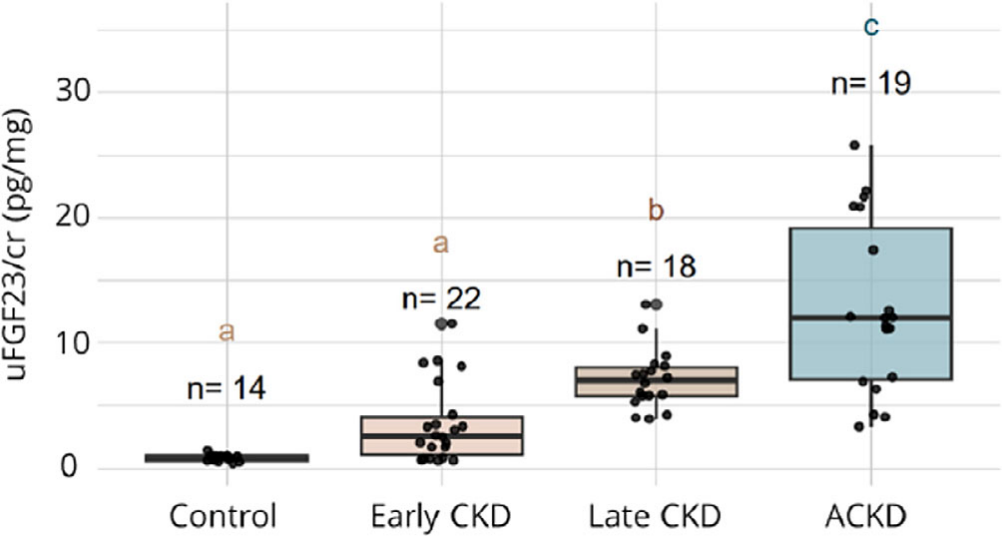
Figure 1. Urinary fibroblast growth factor‐23‐to‐creatinine ratio (uFGF23/cr) in the control group, early chronic kidney disease (CKD) group, late CKD group, and acute decompensated CKD (ACKD) group. *a: the control group and early CKD did not achieve a significant level (*P* = 0.22). *b: the late CKD group has a significantly higher uFGF23/cr compared to the control group (*P* < 0.001) and early CKD group (*P* = 0.04).*c: the ACKD group has a significantly higher uFGF23/cr compared to the other groups (*P* < 0.001 for all pairs).

**Figure:**
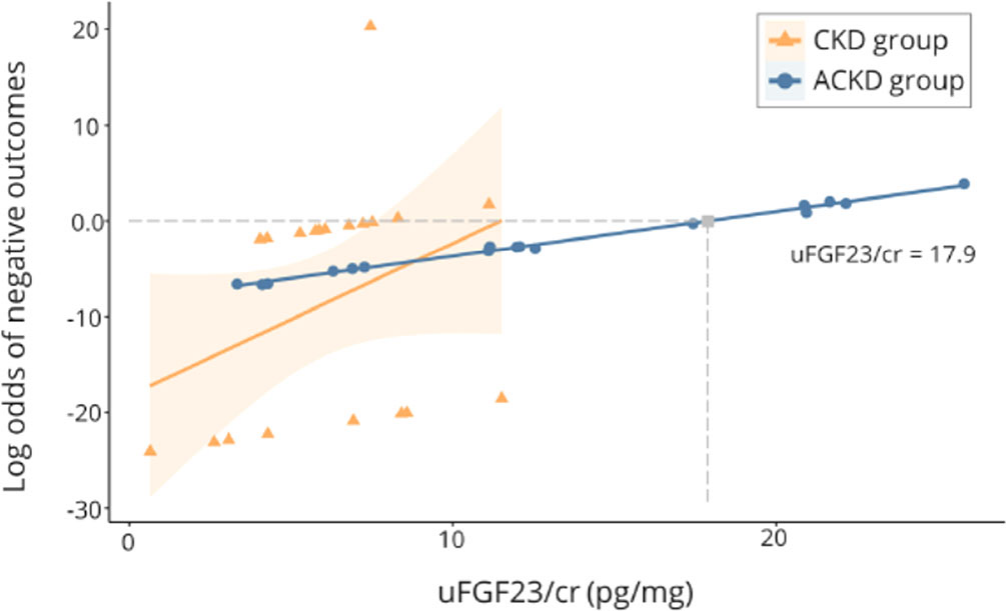
Figure 2. Relationship between uFGF23/cr and the logit to predict negative outcomes, which were defined as an increase in serum creatinine of >0.5mg/dL within 180 days in CKD, or 30‐day mortality in ACKD. After adjusting for the CKD stage, the odds of the negative outcomes increased significantly with an elevated uFGF23/cr in the ACKD group (odds ratio [OR]=1.57, 95% confidence interval [CI]=1.02–2.41, *P* = 0.04) (blue points) but not in cats with CKD (O*R =* 1.33, 95% CI = 0.87–2.03, *P* = 0.22) (orange points). The optimal cutoff was determined to be 17.9 pg/mg according to the estimated function when the logarithm of the odds was equal to zero. The orange band around the line represented the 95% CI. ACKD, acute decompensated chronic kidney disease; CKD, chronic kidney disease; uFGF23/cr, urine fibroblast growth factor‐23‐to‐creatinine ratio.

## Abstract OT01: Cross‐Sectional Investigation of Potential Biomarkers of Aging in Dogs

### **Ellen Ratcliff** ^1^; Brennen McKenzie^1^; Erin McCandless^2^, PhD; James McMahon^3^, BS; Michelle Nelson^4^, BS; Jessica Graves^5^, MS; Ashley Tovar^6^, MS, PhD; Matt Peloquin^7^, BS, MS

#### ^1^Cellular Longevity, Inc., dba Loyal; ^2^Head of Assay and Translational Biomarker Development, Research and Development, Cellular Longevity, Inc., dba Loyal; ^3^Senior Scientist, Preclinical Development, Research and Development, Cellular Longevity, Inc., dba Loyal; ^4^Operations Manager, Research and Development, Cellular Longevity, Inc., dba Loyal; ^5^Senior Data Scientist, Bioinformatics and Engineering, Cellular Longevity, Inc., dba Loyal; ^6^Scientist, Preclinical Development, Research and Development, Cellular Longevity, Inc., dba Loyal; ^7^Team Lead, Preclinical Development, Research and Development, Cellular Longevity, Inc., dba Loyal

**Background:** Hallmarks of aging in humans and rodents include unfavorable alterations to metabolic and immune system function. There is limited evidence characterizing if this is also true in dogs.

**Hypothesis/Objectives:** Determine if changes in insulin, adiponectin and lymphocytes are associated with age in dogs.

**Animals:** Study (1) 451 companion dogs ages 2–6 years and 7–17 years.

Study (2) 60 research beagles ages <4 years, 8–10 years, and >10 years.

**Methods:** Study (1) In this cross‐sectional observational field study, we measured complete blood counts, fasting insulin and adiponectin levels at a single time point. Associations between these blood‐based markers and age were assessed by Spearman correlation. Study (2) In this cross‐sectional observational study, single‐time point blood samples were collected for CBC and flow cytometry analyses. Differences between age groups were evaluated using one‐way analysis of variance.

**Results:** In Study 1, total lymphocyte counts decreased, insulin increased and adiponectin declined significantly with age, even after adjusting for the effect of weight and BCS (*P* < 0.001). In Study 2, total lymphocyte counts and specifically CD3+ T cells, CD3+/CD4+ T‐helper cells and the CD4+/CD8+ ratio were significantly lower in dogs >10 years old compared with dogs <4 years old (*P* < 0.001).

**Conclusions and Clinical Importance:** We demonstrate age‐related changes in biomarkers of immune and metabolic function in dogs. Importantly, these results mirror age‐related changes that are described in rodents and humans. These results support further investigation into these markers as tools to assess negative health outcomes associated with aging in dogs.

## Abstract OT02: Clinical Findings, Treatment Recommendations, and Outcome in Dogs with Known Trilostane Toxicity: 28 Cases (2005–2020)

### **Lindsey M. Summers** ^1^; Laura Stern^2^, DVM, DABVT

#### ^1^Angell Animal Medical Center, Boston, MA, USA; ^2^Director, Training and Quality Assurance, ASPCA Animal Poison Control Center

**Background:** Dogs have been shown to tolerate trilostane administration relatively well. Mild, self‐limiting side effects have been reported. Some patients administered trilostane have been reported to have significant clinical consequences such as lifelong hypoadrenocorticism. There is limited literature about single trilostane exposure events and the clinical outcome for the affected dog.

**Objectives:** Review the clinical findings and outcome in dogs exposed to various doses of inadvertent trilostane administration while emphasizing probable treatment recommendations and outcome.

**Animals:** Twenty‐eight cases were selected from a private toxicology database and specialty service. Data acquired included patient demographics, exposure history to trilostane, diagnostic findings, treatment recommendations and outcome.

**Methods:** Retrospective evaluation of canine trilostane exposure cases from 2005–2020. Cases were selected obtaining data from the ASPCA AnTox database.

**Results:** Median onset of clinical signs after trilostane exposure was 2.5 h (range 0.25–72 h). The most‐reported clinical sign was vomiting (15/28, 53.5%). Majority of cases (15/28, 53.5%) were recommended to seek veterinary care. Eight of the 28 cases included follow‐up information categorized by: dogs which had no clinical signs after 24 h with no veterinary intervention (7/8) vs. with veterinary treatment (1/8). No dog with follow‐up information had signs observed 24 h after exposure.

**Conclusions and Clinical Importance:** Dogs being inadvertently exposed to trilostane are likely to have mild clinical signs. Dogs exhibiting trilostane toxicosis should be managed individually; however, the need for extensive treatments is unlikely even with exposure doses significantly exceeding the published canine trilostane therapeutic dosages for initial hyperadrenocorticism treatment. Further studies are warranted.Table 1. Overview of case demographics

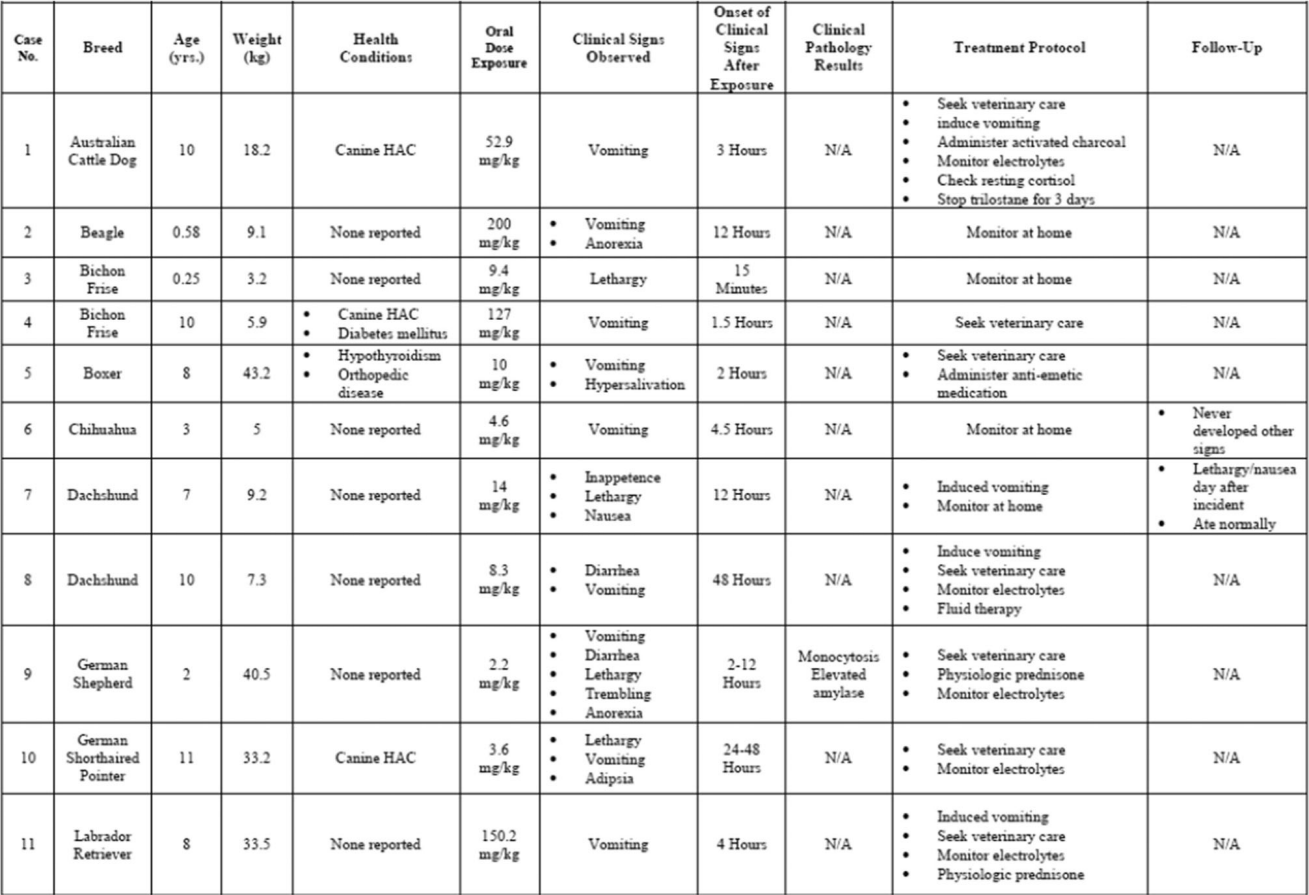

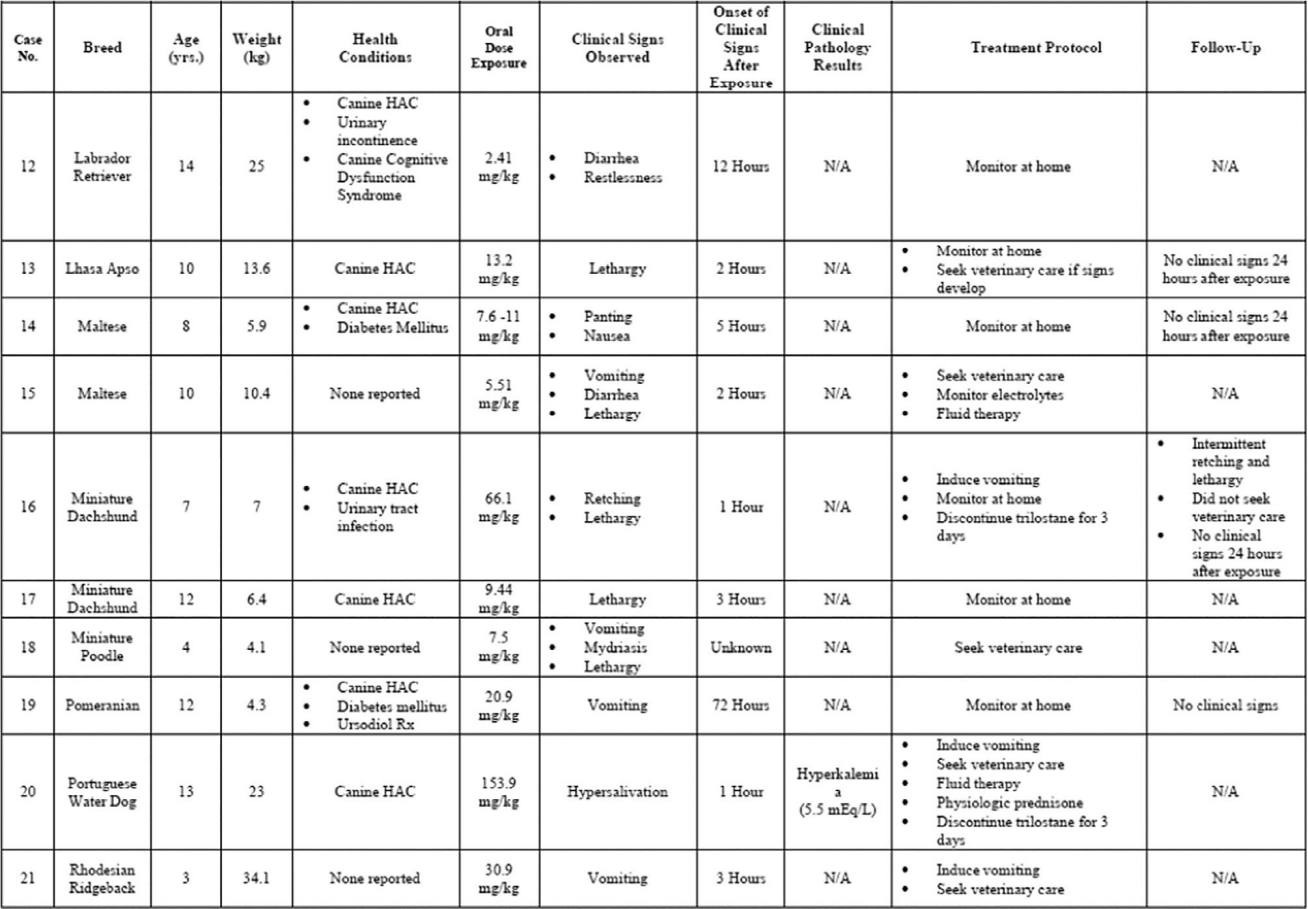

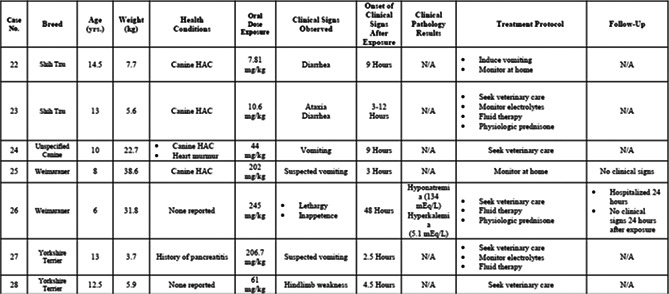

## Abstract OT03: Development, Implementation and Impact of an I‐PASS Based Patient Handoff Initiative

### **Jennifer Adler** ^1^; Richard Stone^1^; Melinda Larson^2^, DACVIM (SAIM)

#### ^1^BluePearl; ^2^Director of Medical Quality, BluePearl

**Background:** Patient handoff between caregivers presents opportunities for the incomplete transfer of information or responsibility of care. Employment of structured patient handoff tools has been shown to improve patient outcomes as well as increase rounding efficiency.

**Objective:** To describe the development, implementation and impact of an I‐PASS based patient handoff system in 100 emergency and specialty hospitals.

**Methods:** An organizational‐wide quality improvement initiative was developed based on TeamSTEPPS communication training utilizing the I‐PASS patient handoff tool. Both verbal as well as a written rounds document were recommended. Hospitals were asked to create champion teams to aid in implementation and self‐auditing processes.

**Results:** Over half of all hospitals (54/100) voluntarily adopted the use of I‐PASS for patient handoffs. The initiative took on a wide variety of forms based on hospital culture, hospital structure and schedule, and level of engagement. Challenges included the perceived redundancy of a written rounds document, pre‐established doctor schedules, and the required changes to workflow. Fifty‐one percent (51%) of survey respondents described their hospital's handoff format as moderately or extremely efficient post initiative. Seventy‐eight percent (78%) described rounds as extremely efficient when the same format was used each time. Vital information was omitted less often by hospitals employing I‐PASS than those that did not.

**Conclusions:** Structured patient handoffs that utilize a consistent format improve rounding efficiency and ensure vital information is not omitted. Adoption requires logistical and cultural shifts. Greater use of structured patient handoffs could positively impact patient safety.

## Abstract OT04: Development and Implementation of a WHO‐Derived Checklist Initiative

### **Jennifer Adler** ^1^; Richard Stone^1^; Melinda Larson^2^, DACVIM (SAIM)

#### ^1^BluePearl; ^2^Director of Medical Quality, BluePearl

**Background:** Surgical safety checklists are widely used in human healthcare and have been shown to decrease the rate of medical errors and improve teamwork. Their use in veterinary medicine is not well described.

**Objective:** To describe the development and implementation of a checklist initiative derived from the WHO Safe Surgical Checklist in 100 emergency and specialty veterinary hospitals.

**Methods:** An organization‐wide quality improvement initiative was developed based on the WHO Safe Surgery Saves Lives Initiative. Hospital teams were provided with WHO‐derived sedation and anesthesia checklists, as well as educational and implementation training. A perception survey was performed to determine baseline rate of use prior to initiative launch.

**Results:** Sixty‐four percent (64%) of respondents reporting using formal procedural and surgical checklists prior to the initiative. Of those, 71% felt comfortable speaking up about patient concerns during procedures. However, only 30% reported reliably discussing patient risk factors prior to procedure and 32% discussed contingency planning after anesthetic procedures. Checklist use was expanded to include contingency planning, and checklists were encouraged to be used by all hospital services. Veterinary technicians played a critical role in initiative success. Most teams adjusted the checklists provided to suit their unique needs, which was encouraged. Participants described verbally introducing themselves as the step they would most like to eliminate.

**Conclusions:** Anesthetic checklists are commonly used in specialty veterinary hospitals, but often without contingency planning. Further study is needed to determine the impact and sustained success of a large‐scale quality‐improvement checklist initiative.

## Abstract OT05: A Retrospective Study of Venom Animals Accidents in Cats in South Brazil

### **Fernanda Vieira Amorim da Costa**; Bianca Giacometti, DVM; Juliane Elisabeth Paz, DVM, MSc

#### UFRGS, Porto Alegre, Brazil

Accidents with venomous animals are uncommon in cats, but when they occur, they can lead to the patient's death if not handled correctly. There are few reports about these accidents in cats and knowing the frequency of the agents is very important.

The objectives of this study were to carry out a retrospective review of accidents with venom animals in cats in South Brazil between 2010 and 2020. Fifty‐one cases registered by the Rio Grande do Sul State Toxicology Information Center (CIT/RS) were included.

The number of cases and related agents was 13 accidents with snakes, where 11 cases involving *Bothrops* and 2 cases involving *Micrurus* genus, 13 accidents with centipedes; 9 cases of accidents with spiders, caused by the genera *Phoneutria* (5/9) and *Loxosceles* (4/9), 8 cases of accidents with insects of the order Hymenoptera; 4 involving bees (*Apis mellifera*), and 2 cases involving wasps (*Polistes* spp.). In two cases (2/8), it was not possible to identify the insect species of the Hymenoptera order. The rest of the accidents included: frogs of *Bufo* genus (4/51), scorpions (3/51), and a moth of *Hylesia* genus (1/51).

With this work, it can be observed that although less frequent, accidents with venomous and poisonous animals in cats occasionally occur, and more specific studies are necessary, since cats are animals with physiological and behavioral peculiarities that must be considered during toxicological emergency care.

## Abstract OT07: Deep Learning Artificial‐Intelligence (AI) Based Approach for Evaluation of Canine and Feline Dermatologic Cytology Samples

### **Kristin Owens**; Mary Lewis, MS, DVM, DACVP (Clinical); Eric Morissette, BSc, DVM, DACVP (Clinical); Cory Penn, DVM

#### Zoetis

**Background:** Dermatology is a top reason for veterinary visits for dogs/cats, and diagnostic testing is a vital part of case management. A digital tool for rapid, consistent evaluation of dermatologic cytology samples is currently lacking.

**Hypothesis/Objectives:** The VETSCAN IMAGYST AI Dermatology application will accurately identify inflammatory leukocytes, bacteria, and yeast on digital slides in agreement with ACVP‐boarded (ACVP) clinical pathologists (CPs).

**Animals:** No animals were utilized in this study. Slide specimens from general practice veterinarians and boarded dermatologists represented a mix of client‐owned canine and feline samples.

**Methods:** Slides (ear swabs, skin swabs, skin impressions; *n =* 218) were prepared in‐clinic, quick Romanowsky stained, coverslipped, and scanned by Grundium Ocus 40. Three ACVP CPs independently scored slides as positive or negative for presence or absence of object classes: inflammatory leukocytes (neutrophils, macrophages, lymphocytes, eosinophils), bacteria (cocci, bacilli), and yeast (*Malassezia* spp.). The VETSCAN IMAGYST AI Dermatology application analyzed these same 218 slides for presence/absence of each object class.

**Results:** The VETSCAN IMAGYST AI Dermatology application reliably identified bacteria (cocci and rods), yeast, and inflammatory leukocytes. Sensitivity for object classes ranged from 77–92%; specificity ranged from 73% to 92%.

**Conclusions and Clinical Importance:** Performance of the VETSCAN IMAGYST AI Dermatology application was comparable to expert ACVP CPs. In‐house utilization of the VETSCAN IMAGYST AI Dermatology application can provide a diagnostic tool for consistently identifying inflammatory cells, bacteria, and yeast.

## Abstract OT08: Evaluation of Factors Associated With Euthanasia versus Unassisted Death in the Dog Aging Project Pack

### **Elizabeth Pearson** ^1^; Jessica Hoffman^2^, PhD; Kate Creevy^3^, DVM, DACVIM; Kellyn McNulty^4^, DVM; Audrey Ruple^5^, DVM, MS, PhD, DACVPM

#### ^1^College of Veterinary Medicine, Texas A&M University, College Station, TX, USA; ^2^Department of Biological Sciences, College of Science and Mathematics, Augusta University, Augusta, GA, USA; ^3^College of Veterinary Medicine and Biomedical Sciences, Texas A&M University, College Station, TX, USA; ^4^Department of Small Animal Clinical Sciences, College of Veterinary Medicine and Biomedical Sciences, Texas A&M University, College Station, TX, USA; DAP Consortium, Dog Aging Project; ^5^Department of Population Health Sciences, Virginia‐Maryland College of Veterinary Medicine, Virginia Tech, Blacksburg, VA, USA

**Background:** The Dog Aging Project (DAP) is a nationwide, long‐term longitudinal study of aging in companion dogs. Each owner who reports the death of an enrolled dog is invited to complete the end of life survey (EOLS) to describe experiences surrounding the death of their dog.

**Objectives:** To examine the relationship between manner of death (unassisted vs. euthanasia) with predictor variables. We also seek to identify factors most likely to contribute to an owner's decision to euthanize.

**Animals:** Deceased DAP pack members whose owners filled out EOLS, first curated data release (*N =* 2570).

**Methods:** Linear and logistic regression models were used to determine factors associated with euthanasia.

**Results:** Demographics of the dogs represented in the survey results are reported in Table 1. Age was a significant predictor of euthanasia, with older dogs more likely to be euthanized than die unassisted (*P* = 8.73E−05). Dogs with ‘old age’ and illness as the owner‐reported cause of death were equally likely to experience euthanasia or unassisted death. Dogs with trauma, sedation complication, and sudden death as the owner‐reported cause of death were less likely to experience euthanasia than unassisted death (all *P* < 0.02). Presence (*P* < 3.61E−13) and number (*P* < 2.2E−10) of aging characteristics were also positively associated with euthanasia.

**Conclusions and Clinical Importance:** Discovery of factors associated with the choice of euthanasia can inform veterinarian communication with owners considering this option. Owner perception of certain characteristics in their dogs may influence their decision to euthanize. Continued analysis of this dataset will evaluate the relative weight of these factors.

**Table:** Table 1. Demographics of dogs for whom EOLS was submitted

	Euthanasia (*N =* 2195)	Unassisted death (*N =* 375)	Total dogs (*N =* 2570)
Age at death			
<1 year	0 (0.00%)	0 (0%)	0
1–<2 years	3 (0.12%)	4 (0.16%)	7
2 years–< 3 years	8 (0.31%)	6 (0.23%)	14
3 years–< 5 years	24 (0.93%)	10 (0.39%)	34
6 years–< 7 years	45 (1.75%)	7 (0.27%)	52
8 years–< 9 years	92 (3.58%)	23 (0.9%)	115
10 years–<11 years	244 (9.5%)	50 (1.95%)	294
11 years–<13 years	508 (19.78%)	79 (3.08%)	587
13 years–< 15 years	662 (25.76%)	112 (4.36%)	774
15 years–<17 years	467 (18.19%)	65 (2.53%)	532
17+ years	141 (5.49%)	18 (0.7%)	159
N/A	1 (0.04%)	1 (0.04%)	2
Sex			
Male, neutered	1063 (41.4%)	174 (6.8%)	1237
Male, intact	53 (2.1%)	21 (0.8%)	74
Female, spayed	1069 (40.7%)	168 (6.5%)	1237
Female, intact	10 (0.4%)	12 (0.5%)	22
Breed status			
Purebred	1166 (45.4%)	228 (8.9%)	1394
Mixed breed	1029 (40.0%)	147 (5.7%)	1176

	Euthanasia (*N =* 2195)	Unassisted death (*N =* 375)	Total dogs (*N =* 2570)
Dog weight class			
0–4.9 kg	117 (4.6%)	29 (1.1%)	146
5–9.9 kg	335 (13.0%)	87 (3.4%)	422
10–14.9 kg	203 (7.9%)	34 (1.3%)	237
15–19.9 kg	200 (7.8%)	26 (1.0%)	226
20–24.9 kg	343 (13.3%)	34 (1.3%)	377
25–29.9 kg	307 (11.9%)	56 (2.2%)	363
30–34.9 kg	306 (11.9%)	40 (1.6%)	346
35–39.9 kg	187 (7.3%)	32 (1.2%)	219
40–44.9 kg	84 (3.3%)	16 (0.6%)	100
45 kg+	113 (4.4%)	21 (0.8%)	134

## Abstract P01: Pharmacokinetics of Subcutaneous Maropitant Alone and Combined with a Subcutaneous Fluid Bolus in Healthy Dogs

### **Kristen Messenger** ^1^; Margaret Gruen^2^, DVM, MPH, PhD, DACVB; Heather Knych^3^, DVM, PhD, DACVCP; Tracey Deiss^4^, DVM

#### ^1^College of Veterinary Medicine, North Carolina State University, Raleigh, NC, USA; ^2^Associate Professor of Clinical Behavior, Department of Clinical Sciences, College of Veterinary Medicine, North Carolina State University, Raleigh, NC, USA; ^3^Professor, California Animal Health and Food Safety Lab, Molecular Biosciences, College of Veterinary Medicine, University of California–Davis, Davis, CA, USA; ^4^Medical Lead, Core Therapeutics, Zoetis

**Background:** Maropitant hydrochloride is labeled for the prevention and treatment of acute vomiting in dogs when administered by subcutaneous (SC) injection. Clinically, maropitant is often injected subcutaneously within a bolus of subcutaneous fluids; however, there is only anecdotal evidence this practice is effective.

**Hypothesis/Objectives:** The objective of this study was to describe the pharmacokinetics of maropitant in dogs when administered as a SC bolus and as a SC bolus combined with SC lactated Ringer's injection (LRS) fluids.

**Animals:** Six healthy, adult, purpose‐bred Beagle dogs.

**Methods:** Dogs received maropitant (1 mg/kg) as a SC injection alone and as a SC injection within a SC bolus of 15 mL/kg LRS in a masked, randomized crossover study with a minimum 4‐day washout period between treatments. Blood samples were collected at pre‐determined time points for 32 h for maropitant quantification using ultra‐high‐pressure liquid chromatography/mass spectrometry. Noncompartmental analysis was used to estimate pharmacokinetic parameters. Data were compared using the Wilcoxon matched pairs signed rank test and *P* < 0.05 was considered significant.

**Results:** Data are presented as median (range) in Table 1. Except for elimination half‐life, the pharmacokinetics of SC maropitant alone or within a bolus of LRS did not differ.

**Conclusions and Clinical Importance:** Elimination half‐life is longer when maropitant is administered with SC LRS. Further studies are needed to determine if there is pharmacodynamic benefit to maropitant administration within a SC LRS bolus in dogs.

**Table:** Table 1. Pharmacokinetic parameter estimates of subcutaneously administered maropitant (1 mg/kg) alone or when administered in a bolus of subcutaneously lactated Ringer's injection (LRS)

Parameter	Units	Median (range)	
		SC maropitant alone	Maropitant + SC LRS
Maximum concentration	ng/mL	37.05 (25.20–70.20)	30.40 (22.6–373.01)
Time to maximum concentration	hr	0.63 (0.25–4.00)	2.00 (0.25–4.00)
Elimination half‐life	hr	8.53 (5.70–12.55)	15.77 (11.17–29.97)*
Mean residence time	hr	13.72 (8.27–19.55)	12.36 (10.59–13.97)
Clearance per fraction absorbed	L/hr/kg	1.71 (1.43–2.42)	1.58 (1.16–2.32)
Volume of distribution per fraction absorbed	L/kg	25.74 (12.89–31.58)	38.11 (23.29–67.11)
Area under the curve time 0 to the last time point	ng*hr/mL	496.20 (375.08–648.52)	456.62 (349.48–700.54)
Area under the curve time 0 to infinity	ng*hr/mL	587.94 (412.61–699.40)	633.44 (430.30–863.67)
Area under the curve extrapolated to infinity	%	8.18 (2.15–18.01)	24.15 (11.65–45.77)
Relative bioavailability	%	N/A	88.71 (72.89–117.78)

*Note*: Legend: SC, subcutaneous; LRS, lactated Ringer's injection

* Denotes value significantly different (*P* < 0.05)

## Abstract P03: Pharmacokinetics of Oral Ondansetron in Hospitalized Dogs Exhibiting Clinical Signs of Nausea

### **Jessica M. Quimby** ^1^; Sarah Shropshire^2^, DVM, PhD, DACVIM (SAIM); Angela Molli^3^; Brooke Gallagher^4^; Samantha Fedotova^4^; Daniel Gustafson^4^; Jessica Quimby^1^; Kristin Zersen^5^

#### ^1^The Ohio State University, Columbus, OH, USA; ^2^Assistant Professor, Colorado State University, Fort Collins, CO, USA; ^3^Texas A&M University, College Station, TX, USA; ^4^Colorado State University, Fort Collins, CO, USA; ^5^Emergency and Critical Care, Colorado State University, Fort Collins, CO, USA

**Background:** Oral ondansetron is commonly prescribed to dogs but pharmacokinetics in clinical patients have not been evaluated.

**Hypothesis/Objectives:** To evaluate the pharmacokinetics of oral ondansetron in a population of hospitalized dogs exhibiting clinical signs of nausea.

**Animals:** Twenty‐four client‐owned dogs.

**Methods:** A prospective blinded study was performed. Dogs were randomly assigned to one of the following oral ondansetron protocols: 1 mg/kg q 12 h, 0.5 mg/kg q 12 h, mg/kg q 8 h, or 0.5 mg/kg q 8 h. Plasma was initially collected at 0, 0.25, 0.5, 1, 2, 4, 8, 16, and 24 h after the first dose in 7 dogs and was collected at 0, 2, 4, and 8 h in the remaining 17 dogs. Ondansetron concentrations were measured via high‐performance liquid chromatography coupled to tandem mass spectrometry. Below limit of quantification (BLOQ) values were arbitrarily set at 1 ng/mL for statistical analysis. Plasma ondansetron concentrations were compared between groups using a Mann‐Whitney test.

**Results:** Ondansetron concentrations were BLOQ in 18/36 (50%) samples in the 0.5 mg/kg groups and 14/36 (39%) samples in the 1 mg/kg groups. Plasma ondansetron concentrations were not significantly different between these groups [0.5 mg/kg group median 8.53 ng/mL; range 1–96.83 ng/mL and 1 mg/kg group median 7.43 ng/mL; range 1–278.67 ng/mL]. In six dogs, ondansetron was not detected at any timepoint.

**Conclusions and Clinical Importance:** Ondansetron was not detected in the plasma of multiple dogs after oral administration, raising concern for efficacy at currently recommended dosages.

## Abstract R01: Objective Assessment of Computed Tomographic Attenuation in Dogs With and Without Bronchomalacia

### **Charlotte Gerhard** ^1^; Isabelle Masseau^2^, DACVR, PhD; Aida Vientós‐Plotts^3^, DVM, PhD, DACVIM; Gregory Petroski^4^, PhD; Carol Reinero^5^, DVM, PhD, DACVIM

#### ^1^University of Missouri, Columbia, MO, USA; ^2^Associate Professor, Department of Clinical Sciences, Faculté de Médecine Vétérinaire, Université de Montréal, Montréal, QC, Canada; ^3^Assistant Professor, Small Animal Internal Medicine, College of Veterinary Medicine, University of Missouri, Columbia, MO, USA; ^4^Assistant Research Professor, Biostatistician, Biostatistics, School of Medicine, University of Missouri, Columbia, MO, USA; ^5^Professor, Small Animal Internal Medicine, College of Veterinary Medicine, University of Missouri, Columbia, MO, USA

**Background:** Paired inspiratory and expiratory breath‐hold computed tomographic scans (I:E‐BH CT) capture images with control of respiratory phase, important with dynamic airway collapse. CT attenuation histograms allow objective quantification of distribution of Hounsfield units (HU).

**Hypothesis/Objectives:** We hypothesize that dogs with bronchomalacia (BM) will demonstrate significant differences in objective metrics of attenuation on I:E‐BH CT, reflecting changes in airway caliber and impaired downstream parenchymal aeration compared to dogs without BM (NoBM). We aimed to document objective metrics of percentage low and high attenuating areas (LAA% and HAA%) and mean lung attenuation (MLA) on I:E‐BH CT in BM/NoBM using automated software.

**Animals:** Client‐owned dogs with respiratory signs and BM (*n =* 118) and NoBM (*n =* 25).

**Methods:** This retrospective study utilized 3D Slicer software to assess CT attenuation. Analysis used a Spearman correlation and a 2‐way ANOVA utilizing beta regression.

**Results:** Strong correlations were identified within 4 LAA and 4 HAA metrics; thus, the representative LAA%‐856 and HAA%‐700 were selected. BM had a significantly lower LAA%‐856 (19%) than NoBM (31%) (*P* = 0.013), independent of phase (I/E). Groups differed for HAA%‐700 with accentuation in expiration versus inspiration (*P* < 0.001). On expiration, BM versus NoBM had 61% and 35% of lung <–700 HU. MLA between groups was phase dependent with BM versus NoBM on inspiration being –708 HU and –776 HU and on expiration, –567 HU and –686 HU, respectively (*P* < 0.0001).

**Conclusion and Clinical Importance:** Higher lung attenuation in BM dogs supports downstream parenchymal aeration to segmental/subsegmental airway collapse. Quantitative imaging holds promise for objectively evaluating changes with BM.

## Abstract R02: Clinical, Imaging and Rhinoscopy Findings in 63 Dogs and 8 Cats with Nasal Foreign Body

### **Mireia Pascual Moreno** ^1^; Hannah Shing^2^, DVM; Ferran Valls Sanchez^3^, DECVIM‐CA (Internal Medicine)

#### ^1^Dick White Referrals; ^2^Resident EVCDI, Diagnostic Imaging, Dick White Referrals; ^3^Consultant and Head of Service, Internal Medicine, Dick White Referrals

**Background:** Nasal foreign bodies (FBs) have been reported as a common etiology of nasal disease.

**Objectives:** Description of clinical presentation and diagnostic findings in dogs and cats with confirmed nasal FBs.

**Animals:** Sixty‐three dogs and 8 cats were included.

**Methods:** Retrospective descriptive study. Clinical presentation, imaging and rhinoscopy findings of dogs and cats, between 2010 and 2022, were reviewed.

**Results:** Median length of clinical signs was 7 and 45 days in dogs and cats, respectively. Most common clinical signs were sneezing (46/71 cases) and nasal discharge (43/71 cases). Among patients with nasal discharge, 17/43 presented with epistaxis. The discharge was unilateral in 37/43 cases. Despite detection of a FB in only one nasal cavity, 6 cases had bilateral discharge. Imaging, predominantly computed tomography, was performed in 48/71 cases, with direct visualization of the foreign body in 5 dogs and 3 cats. Unilateral changes were described in 40/48 cases with turbinate destruction in 19/48 cases, being focal in 16/19 cases. Foreign body removal was achieved through rhinoscopy or nasal flushing in 66 and 4 cases, respectively. It was not possible to retrieve the FB in one case.

**Conclusions and Clinical Importance:** Based on the findings of this study, although unilateral nasal discharge was more common, nasal FBs should still be a differential diagnosis in bilateral cases, and cats seemed to have a more chronic presentation. Visualization of the nasal foreign body in advanced imaging was only reported in small minority of cases; consequently, inability to identify it does not exclude it.

## Abstract R03: Using Probiotics to Modulate the Respiratory Microbiome in Feline Allergic Asthma

### **Julia D. Remaks** ^1^; Aida Vientos‐Plotts^2^, DVM, PhD, DACVIM; Hansjorg Rindt^3^, PhD; Aaron Ericsson^4^, DVM, PhD, DACLAM; Carol Reinero^5^, DVM, PhD, DACVIM

#### ^1^Veterinary Health Center, University of Missouri, Columbia, MO, USA; ^2^Faculty, Small Animal Internal Medicine, Veterinary Health Center, University of Missouri, Columbia, MO, USA; ^3^Laboratory Manager, Comparative Internal Medicine Laboratory, University of Missouri, Columbia, MO, USA; ^4^Assistant Professor, Department of Veterinary Pathobiology, University of Missouri, Columbia, MO, USA; ^5^Faculty, Veterinary Health Center, University of Missouri, Columbia, MO, USA

**Background:** Feline asthma is associated with respiratory dysbiosis, correction of which could serve as a novel treatment.

**Hypothesis/Objectives:** We hypothesized that in asthmatic cats treated with anti‐inflammatory glucocorticoids, probiotics would attenuate the asthmatic phenotype and beneficially alter respiratory, blood and oropharyngeal (OP) microbial communities and immune parameters versus placebo.

**Animals:** 13 client‐owned asthmatic cats.

**Methods:** A randomized, blinded, placebo‐controlled clinical trial of asthmatic cats receiving anti‐inflammatory glucocorticoids with oral probiotics (PRO), or placebo (CONTROL) assessed airway eosinophilia and owner‐perceived improvement at baseline and after 2 weeks of treatment. Bronchoalveolar lavage fluid (BALF), blood, and OP microbial communities were compared using 16S rRNA amplicon sequencing. Real‐time PCR for transcription factors, activation markers and cytokines, and IgA ELISAs were used. Statistical analyses used two‐way RM‐ANOVA or PERMANOVA (significance, *P* < 0.05).

**Results:** There were no significant differences in mean ± SEM BALF eosinophils (PRO, 45.6 ± 7.51; CONTROL, 31.3 ± 5.9) or improvement in owner‐perceived clinical signs between groups. There was no significant difference in alpha‐ or beta‐diversity in BALF, blood, and OP samples between groups or over time. There were no significant differences in CD25, foxP3, GATA, Helios, IL‐4, IL‐5, IL‐10, IL‐13, IL‐17, IFN‐γ or serum or BALF IgA between groups or over time.

**Conclusions and Clinical Importance:** In asthmatic cats, oral probiotics failed to reduce airway eosinophilia, improve owner‐perceived signs, modify microbial community composition, or alter all assessed immune responses versus placebo or over time. Longer treatment, different composition or delivery (e.g., aerosolized) of probiotic, or larger number of cats would represent next stages of study.

## Abstract R04: Retrospective Evaluation of High‐Velocity Nasal Insufflation in Cats with Respiratory Disease

### **Ashlynne Wampfler** ^1^; Jessica Ward^1^; Melissa Tropf^1^; Allison Masters^2^; Meredith ‘t Hoen^1^; April Blong^1^; Rebecca Walton^1^

#### ^1^Iowa State University, Ames, IA, USA; ^2^University of Minnesota, Minneapolis, MN, USA

**Background:** Historically, mechanical ventilation has been the only therapeutic tool when failing traditional oxygen therapy. The goal of this study is to describe the use of high‐velocity nasal insufflation (HVNI) in cats.

**Hypothesis/Objectives:** We hypothesized that HVNI would be effective and well tolerated in cats with respiratory distress.

**Animals:** Eight cats admitted to a referral center with respiratory distress refractory to traditional oxygen therapy.

**Methods:** Retrospective descriptive study between the years 2019–2022. Medical records were retrospectively reviewed for signalment, indication for HVNI, complications while on HVNI, successful discontinuation of HVNI, and survival.

**Results:** A total of 8 cats had HVNI instituted between the years 2019–2022. Four cats underwent HVNI due to pneumonia, 1 secondary to acute respiratory distress syndrome, 1 post‐thoracotomy for peritoneal‐pericardial diaphragmatic hernia repair, and 2 were unknown respiratory disease. No cats underwent HVNI due to cardiac disease. The median time spent on HVNI was 17 h (range 2–76 h). The median flow rate was 1 L/kg (range 0.612–2.68). The median highest FiO_2_ recorded was 100% (range 50–100), and the median lowest FiO_2_ was 52.5% (range 40–100%). Three cats were successfully discontinued from HVNI and two cats survived to discharge. All cats tolerated HVNI without complication.

**Conclusions and Clinical Importance:** This is the first study to evaluate the use of HVNI for ventilatory support in cats. This study demonstrates that HVNI is a feasible option in cats requiring more aggressive treatment than traditional oxygen therapy without the use of mechanical ventilation.

## Abstract R05: Causes of Pleural Effusion in 67 Cats—A Retrospective Study

### **Fernanda Vieira Amorim da Costa**; Bianca Menezes, DVM; Mirela Paim, DVM, MSc; Aline Staudt, DVM; Izadora Zardo, DVM

#### UFRGS, Porto Alegre, Brazil

Pleural effusion is the abnormal accumulation of fluid in the pleural space. Data from the literature show that heart disease probably is the most frequent cause of pleural effusion in cats; however, it can vary due to the prevalence of infectious diseases and the epidemiological profile of affected cats. The underlying disease is defined through analysis of cavitary liquid, clinical data, and additional imaging and laboratory tests.

The objectives of this study were to carry out a retrospective review of cases of feline pleural effusion diagnosed in Brazil between 2016–2019. Our hypothesis was that mediastinal lymphoma associated with FeLV is the main cause of pleural effusions in this population. Cases were evaluated and information about sex, age, clinical signs, retrovirus status, and the definitive diagnosis were collected.

A total of 67 animals were included in the study, 35 were female (52,2%) and 32 males (47,8%). Age ranged from six months to 17 years. Dyspnea was the main sign in 39 cats (58,2%), followed by anorexia in 29 cats (43,3%). Cytologic analysis resulted in 36 neoplastic (53,7%), 8 modified transudates (11,9%), 9 aseptic exudates (11,9%), 4 pure transudates (6%), 4 septic exudates (6%), 4 hemorrhagic effusion (6%) and 3 chylothorax (4,5%). Mediastinal lymphoma was the most frequent cause of pleural effusion (53,7%), and all cats were positive in the POC FeLV antigen test. It can be concluded that the main cause of pleural effusion was mediastinal lymphoma in this population, which is associated with the high prevalence of FeLV in the region.

## Abstract R06: Chronic Rhinosinusitis in Cats—An Etiological Study

### **Fernanda Vieira Amorim da Costa** ^1^; Deisy Lerner^2^, DVM, MSc; Gabriela Sessegolo^1^, DVM, MSc; Marcele Bandinelli^1^, DVM, MSc, PhD; Andréia Spananberg^1^, DVM, MSc, PhD; Silvana Vidor^1^, DVM, MSc; Caroline de Andrade^1^, DVM, MSc, PhD; Laerte Ferreiro^3^, DVM, MSc, PhD; Carlos Afonso Beck^4^, DVM, MSc, PhD

#### ^1^UFRGS, Porto Alegre, Brazil; ^2^Gatices Veterinary Hospital; ^3^Full Professor, Department of Clinical Pathology, UFRGS, Porto Alegre, Brazil; ^4^Full Professor, Department of Animal Medicine, UFRGS, Porto Alegre, Brazil

Diseases that chronically affect the feline upper respiratory tract (URT) represent a frequent problem in feline practice and may represent a diagnostic and therapeutic challenge. The purpose of this study was to pursue a prospective investigation to determine the etiological causes for chronic rhinosinusitis in cats, and investigate the association between them and patient's history, clinical signs, and radiologic findings.

Cats presenting URT disease signs were selected to perform physical exam, bacterial and mycological cultures, cryptococcosis serology, skull radiographs, rhinoscopy and/or rhinotomy, histopathology, FHV‐1 and FCV immunohistochemistry and *Mycoplasma* spp. PCR.

Twenty‐one (58.3%) of 36 cats had idiopathic chronic rhinosinusitis (ICRS), 9 neoplasia (25.0%), 3 fungal rhinitis (8.3%), 2 structural changes, and 1 nasopharyngeal polyp. Most common signs were sneezing, nasal discharge, and stertorous breathing. Skull radiographs revealed mostly increased nasal cavity opacity, turbinate bones detail loss, and frontal sinus involvement. Significant statistical association was found between ICRS, mucopurulent discharge, and bilateral frontal sinus increased opacity in radiographs. Diagnosis of neoplasia and fungal rhinitis had significant association with appetite loss, sanguineous discharge, facial deformities, and nasal septum deviation on radiographs. All cases of ICRS had negative results for FHV‐1 and FCV immunohistochemistry.

We conclude that ICRS is the most prevalent chronic rhinosinusitis disease, but other important etiologies are also present and should be suspected, especially when there is a combination of clinical signs such as sanguineous discharge, facial deformity, and decreased appetite. Also, FHV‐1 and FCV are probably not important as being perpetuating pathogens of ICRS.

## Abstract R07: Comparing the Nasal Transcriptome and Microbiome in Healthy Dogs and Dogs With Chronic Rhinitis

### **Zhe (Alice) Wang** ^1^; Steven Dow^2^; Lyndad Chow^1^, PhD

#### ^1^Colorado State University, Fort Collins, CO, USA; ^2^Professor, Colorado State University, Fort Collins, CO, USA

**Background:** Little is known regarding nasal immune responses and how the nasal microbiome may be altered in dogs with chronic rhinitis (CR).

**objectives/hypothesis:** To compare the nasal transcriptome and microbiome in healthy dogs and dogs diagnosed with chronic idiopathic rhinitis using next gen sequencing approaches. We hypothesized that the nasal transcriptome and microbiome in dogs with CR were significantly different than in healthy dogs.

**Animals:** Prospective study enrolling six dogs with histologically confirmed chronic idiopathic lymphoplasmacytic rhinitis and six age‐matched healthy dogs.

**Methods:** Nasal swabs were collected under general anesthesia. For transcriptomic analysis, RNA was extracted from swabs and subjected to full RNA sequencing using Illumina sequencing with alignment to the CanFam3 database. Sequence files were analyzed using Partek Flow software. To evaluate the nasal microbiome, DNA was extracted from nasal swabs and subjected to 16S sequencing and analyzed using open‐source software. Correlations between the nasal transcriptome and the nasal microbiome were assessed using Pearson correlation analysis.

**Results:** Pathway analysis of transcriptome data revealed significant upregulation of pathways related to reactive oxygen and STAT3 signaling compared to healthy dogs. Notably absent were pathways related to allergic responses. The microbial composition appeared different in dogs with chronic rhinitis and healthy dogs. We identified significant correlations between certain overexpressed immune genes in dogs with CR and their microbiome phyla.

**Conclusion and Clinical Importance:** Marked immunological differences in the nasal transcriptomes of dogs with CR. These findings suggest important interactions between nasal immune responses and the nasal microbiome in dogs with CR.

